# Mitochondrial Genome Sequence of *Salvia officinalis* (Lamiales: Lamiaceae) Suggests Diverse Genome Structures in Cogeneric Species and Finds the Stop Gain of Genes through RNA Editing Events

**DOI:** 10.3390/ijms24065372

**Published:** 2023-03-11

**Authors:** Heyu Yang, Haimei Chen, Yang Ni, Jingling Li, Yisha Cai, Jiehua Wang, Chang Liu

**Affiliations:** 1 School of Environmental Science and Engineering, Tianjin University, Tianjin 300072, China; 2 Institute of Medicinal Plant Development, Chinese Academy of Medical Sciences, Peking Union Medical College, Beijing 100193, China

**Keywords:** *Salvia officinalis*, Lamiales, mitogenome, multi-chromosomal structure, introns

## Abstract

Our previous study was the first to confirm that the predominant conformation of mitochondrial genome (mitogenome) sequence of *Salvia* species contains two circular chromosomes. To further understand the organization, variation, and evolution of *Salvia* mitogenomes, we characterized the mitogenome of *Salvia officinalis*. The mitogenome of *S. officinalis* was sequenced using Illumina short reads and Nanopore long reads and assembled using a hybrid assembly strategy. We found that the predominant conformation of the *S. officinalis* mitogenome also had two circular chromosomes that were 268,341 bp (MC1) and 39,827 bp (MC2) in length. The *S. officinalis* mitogenome encoded an angiosperm-typical set of 24 core genes, 9 variable genes, 3 rRNA genes, and 16 tRNA genes. We found many rearrangements of the *Salvia* mitogenome through inter- and intra-specific comparisons. A phylogenetic analysis of the coding sequences (CDs) of 26 common protein-coding genes (PCGs) of 11 Lamiales species and 2 outgroup taxa strongly indicated that the *S. officinalis* was a sister taxon to *S. miltiorrhiza*, consistent with the results obtained using concatenated CDs of common plastid genes. The mapping of RNA-seq data to the CDs of PCGs led to the identification of 451 C-to-U RNA editing sites from 31 PCGs of the *S. officinalis* mitogenome. Using PCR amplification and Sanger sequencing methods, we successfully validated 113 of the 126 RNA editing sites from 11 PCGs. The results of this study suggest that the predominant conformation of the *S. officinalis* mitogenome are two circular chromosomes, and the stop gain of *rpl*5 was found through RNA editing events of the *Salvia* mitogenome.

## 1. Introduction

*Salvia officinalis* L., also known commonly as sage, is an economically important aromatic and medicinal plant. It belongs to the mint family of Lamiaceae within Lamiales [1]. *Salvia* means “to heal or save” in Latin, and its species name “officinalis” means “medicinal” [2,3]. Sage has a historical reputation for preventing and curing illnesses and was regarded as a sacred plant in Ancient Rome [3,4]. It has been extensively investigated for its antioxidant, antibacterial, hypoglycemic, and anti-inflammatory properties [5,6]. Moreover, sage can prevent and cure cardiovascular diseases, brain and nervous disorders, and diabetes [7]. The aerial parts of sage were used mostly for the extraction of active constituents [5,8]. Two principal types of secondary metabolites were found in *S. officinalis*: terpenoids and phenolics [6]. The most active constituents of *S. officinalis* are essential oils (1–2.8%), including 1,8-cineole, camphor, α-thujone, β-thujone, borneol, and viridiflorol [9].

The nuclear genome of *S. officinalis* was reported recently and revealed a genomic size of 480 Mb and seven chromosomes [10]. Two expression cascades have been identified as the regulating factors of the biosynthesis of shoot and root diterpenoids, particularly the diterpene biosynthesis gene cluster [10]. Furthermore, genetic and metabolomic studies have focused on discerning the genes and pathways that underpin volatile terpenoid biosynthesis [11,12] and the genetic diversity of seven *S. officinalis* populations in Greece [13].

Organelle genomes including the genomes of mitochondria and plastids [14,15] are resources for species identification, evolution, and phylogenetic studies [16,17,18]. They all evolved from bacterial endosymbionts [19,20]. Similar evolutionary histories lead to many common characteristics of the mitochondrial and plastid DNAs (mtDNAs and ptDNAs). However, mitochondrial genomes (mitogenome) present more complexity and more pronounced eccentricities than plastid genomes [21]. In particular, the mitogenomes of higher plants show enormous diversity in their genome sizes, structures, and gene contents [14,22].

The mitogenome size ranged from 11 Mb for *Silene conica* [23], to 66 kb for *Viscum scurruloideum* [24]. The size variations were reported to be extremely divergent even in the same genus *Silene* [25]. Different mechanisms contribute to these size variations of plant mitogenome including the proliferation of repeat elements, the incorporation of foreign DNA [22], and the gain or loss of whole chromosomes [26].

The structures of the mitochondrial DNA in angiosperm plants showed enormous diversity at three levels: inter-specific, intra-specific, and within individual levels. At the inter-specific level, different technologies have been used to observe the mitogenome structure diversity. For example, the in-gel fluorescence microscopy of *Lactuca sativa* mtDNA molecules showed the presence of linear, branched, and circular structures, with branched linear forms representing a prevailing presence [27]. In cultured tobacco cells, linear molecules and multibranched molecules in mtDNA were observed by the pulse-chase experiments with 3H-thymidine [28]. In cultured *Chenopodium album* (L.) cells, linear, circular, sigma-like, branched, and rosette-like structures were observed by electron microscopy [29]. The plant mitogenomes assembled from high-throughput DNA sequencing data are also found to have complex structures [30]. For example, the cucumber mitogenome was assembled into a single circular master chromosome and a collection of sub-master circular chromosomes [31]. The most prominent mechanism for structure diversity is likely to be the intra- and inter-molecular recombinations mediated by direct repetitive sequences [32,33]. 

In addition to the inter-specific level, the mitogenome structures were also found to be highly diverse at the intra-species level. For example, seven mitogenomes of sorghum from different cultivars and wild sources showed reticulated structures with multilinked relationships and were grouped into three types [34]. The different types of mitogenome structures could be associated with certain domesticated events.

Enormous diversity was also expected at the individual level, considering the fact that one cell has multiple copies of mitogenomes. Indeed, representing the mtDNAs as circular or linear chromosomes might not be appropriate, as these mtDNAs might exist as polymeric complexes. In this case, they are best represented with a graph. Recently, a graph-based sequence assembly toolkit (GSAT) was developed to assemble and obtain high-quality mitochondrial master graphs (MMGs) to represent the full spectrum of structural conformations of plant mitogenomes [30], demonstrating another level of mitogenomic structural diversity.

The mitogenome contents, in terms of the number of PCGs and introns, are also found to be highly diverse. The number of PCGs ranges from 19 in *Viscum scurruloideum* [24] to more than 50 in the liverwort *Marchantia polymorpha* [35,36]. Additionally, the number of introns varies from 4 in *Viscum scurruloideum* [24] to more than 40 in the hornwort *Anthoceros agrestis* [37]. In the angiosperm mitogenomes, 24 core protein-coding genes (PCGs) encode the respiratory proteins, and 17 variable PCGs encode the ribosomal proteins [38,39,40].

The diversity of the mitogenome contents was further increase by a post-transcriptional modification process, called RNA editing [41]. The number of RNA editing sites varies extensively among different mitogenomes, ranging from 8 RNA in the moss *Physcomitrella patens* to 2139 in *Selaginella* [42]. Previously, we identified 225 RNA editing sites in the *S. miltiorrhiza* mitogenomic mRNA. In particular, we found 115 symmetric RNA editing sites, characterized by being on two different strands at the same position [43]. Given the prevalent RNA editing events, the mitogenome sequences alone are insufficient to detect the mitochondrial proteome sequences. The identification of RNA editing sites is important to determine the final output of the functional mitochondrial proteins.

The plastid genomes of *S. officinalis* have been reported [12,44,45]. We also developed novel molecular markers for authenticating three *Salvia* species [45]. The nuclear genome of *S. officinalis* has also been reported, but the mitogenome of *S. officinalis* remains unknown, limiting our ability to understand the interaction of the three genomes in *S. officinalis.*

Our long-term goal is to understand the diversity and evolution of *Salvia* mitogenomes, thereby ultimately providing valuable information for molecular breeding studies on these economically important *Salvia* species. Recently, we confirmed for the first time that the predominant conformations of *Salvia* mitogenomes contain two circular molecules [46]. In the present study, we sequenced the mitogenome of *S. officinalis.* We ascertained the mitogenomic structure, gene content, repeat sequences, and RNA editing sites. We then compared the mitogenome and plastome to identify the mitochondrial plastid DNA (MTPT) and their evolutionary congruence. Lastly, we compared the mitogenome of *S. officinalis* with those of related species. The results obtained from this work lay a solid foundation for comprehending the diversity and evolution of *Salvia* mitogenomes.

## 2. Results

### 2.1. Structure of the S. officinalis Mitogenome

**Figure 1:**
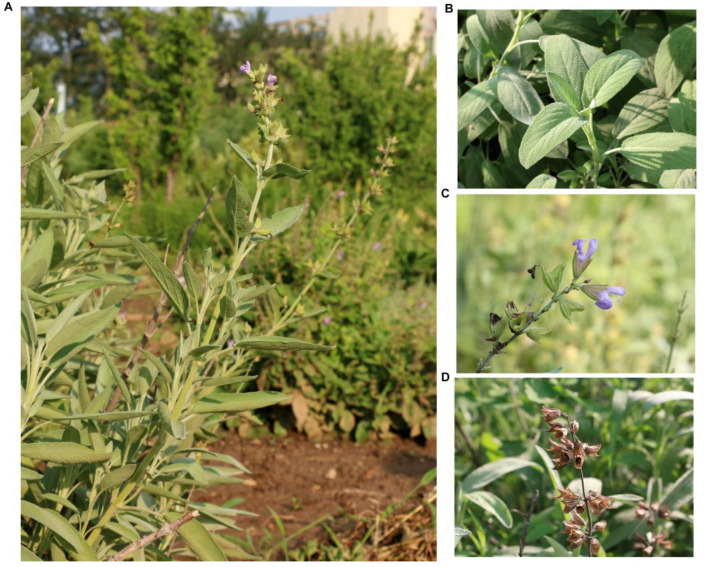
Photographs of the aerial parts of *S. officinalis*. The images were photographed by Dr. Xinlei Zhao from the Institute of Medicinal Plant Development, Chinese Academy of Medical Sciences, Beijing, China, and are used in this study with permission. (**A**) The entire plant; (**B**) the leaves; (**C**) the flowers; and (**D**) the fruits and seeds.

*S. officinalis* is a low (40–100 cm) shrub, and its leaves are oblong. The whole plants, aerial parts, leaves, flowers, and fruits of *S. officinalis* are shown in Figure 1A–D.

**Figure 2:**
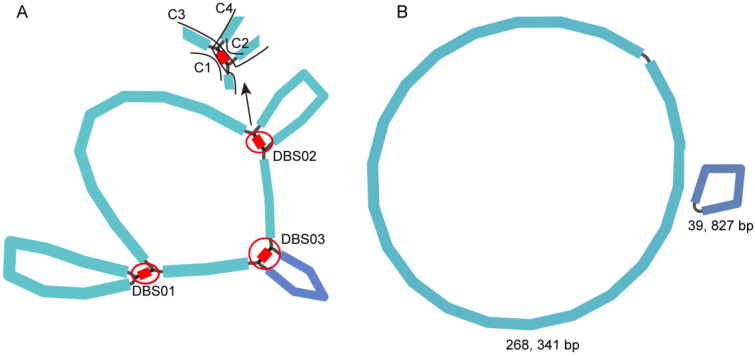
Schematic representation of the assembly steps of *S. officinalis* mitogenome. (**A**) Unitig graph of the *S. officinalis* mitogenome obtained from de novo assembly of Illumina reads with GetOrganelle. The unitig graph contained six contigs (blue and purple) that formed DBSs (DBS01–03, highlighted in the red circles). Each DBS has four conformations (c1, c2, c3, and c4), as shown in the top left corner, with DBS02 as an example. (**B**) The mitogenomic chromosomes MC1 (light blue circle) and MC2 (dark blue circle) of *S. officinalis* after the DBSs shown in (**A**) were resolved using Unicycler software.

We obtained 32 Gb of Illumina sequencing data and 22 Gb of Nanopore sequencing data. The statistics of the sequencing results can be found in Table S1. The mitochondrial reads were extended from the raw sequencing reads using the GetOrganelle toolkit and de novo assembled using Unicycler software. The assembly result from GetOrganelle is shown as a unitig graph in Figure 2A. Three double-bifurcation structures (DBS) are designated as DBS01–03 in Figure 2A. Each of the DBS structures contained four alternative conformations—1, 2, 3, and 4—correspondingly named c1–4.

Unicycler software was used to resolve these DBS structures, which resulted in two circular sequences (Figure 2B). These two sequences were considered mitogenomic chromosomes, named MC1 and MC2, and were 268,341 bp and 39,827 bp in length and had GC contents of 45.23 and 44.32, respectively. To confirm that these three DBS structures were resolved correctly, we manually mapped all long reads to the sequences corresponding to the four conformations c1, c2, c3, and c4 of each DBS (Figure S1). The percentages of each conformation were quantified by the ratio of the number of long reads mapped to its sequence and those mapped to all four conformations (Table 1). According to the calculation, the relative abundances of the less abundant conformations of DBS01, 02, and 03 were 8.93%, 15.00%, and 20.69%, respectively. The details for the HSP r01, r02, and r47 are provided in Section 2.3. 

To check the quality of the assemblies, we mapped the Nanopore and Illumina reads back to the MC1 and MC2 sequences. The mitogenome was mostly uniformly covered, with the Nanopore reads having average depths of coverage of 237.31 for MC1 (Figure S2a) and 53.72 for MC2 (Figure S2b). Similarly, the mitogenome was uniformly covered, with the Illumina short reads having average coverage depths of 285.93 for MC1 (Figure S2c) and 161.89 for MC2 (Figure S2d).

**Figure 3:**
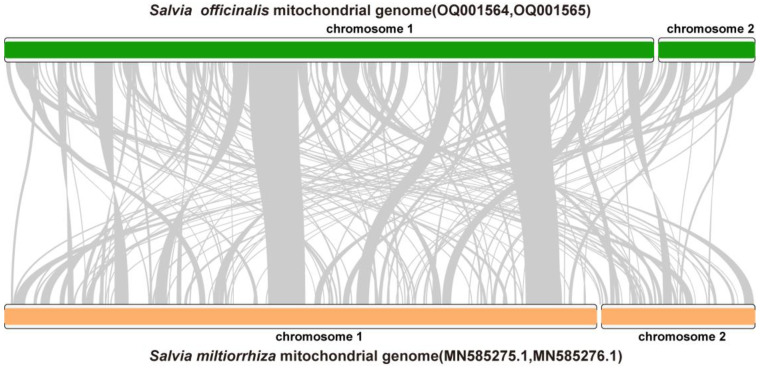
Syntenic analysis of the mitogenome sequences of *S. officinalis* and *S. miltiorrhiza*. The chromosomes of *S. officinalis* and *S. miltiorrhiza* are shown with green and orange blocks, respectively. DNA fragments having a similarity > 85% were connected with gray lines.

To determine the inter-specific variations of *Salvia* mitogenomes, we compared the mitogenome sequences of *S. officinalis* and *S. miltiorrhiza* [46] using Mummer (v3) [47] (Figure 3). As shown, there were many rearrangements between the two genomes (Figure 3). The lengths of the alignable regions were 177,342 bp (57.55%) for the *S. officinalis* mitogenome sequences and 176,363 bp (42.59%) for the *S. miltiorrhiza* mitogenome sequences, respectively (Figure 3).

### 2.2. Gene and Intron Content of S. officinalis and Other Related Lamiales Mitogenomes

**Figure 4:**
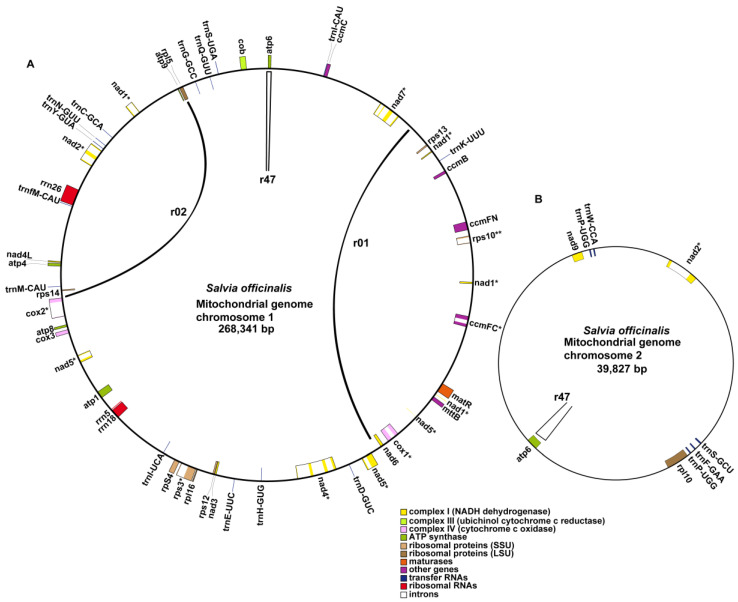
Schematic of the circular MC1 (**A**) and MC2 (**B**) of *S. officinalis*. The graph was drawn using PMGView (http://www.1kmpg.cn/pmgview (accessed on 28 October 2022)). Genes shown on the inside were on the negative strand, and those on the outside were on the positive strand. Genes with introns were highlighted using “*”. The colors indicate different functional categories. The locations of the three repetitive sequences (r01, r02, and r47) that could mediate homologous recombination in the *S. officinalis* mitogenome are highlighted inside the circles. The two repeat units of r01 and r02 are on chromosome 1 and are connected with arcs. In contrast, the two repeat units of r47 are distributed on chromosome 1 and 2.

The *S. officinalis* mitogenome encoded 33 PCGs, 3 rRNA genes, and 17 tRNA genes. The distribution of these genes, along with three repetitive sequences (r01, r02, and r47) that might mediate homologous recombinations are shown in Figure 4. Details for the repetitive sequences are provided in Section 2.3. The 33 PCGs included the 24 core PCGs found in most of the angiosperm plants and 9 variable PCGs (Table 2), consistent with prior descriptions for angiosperm mitogenomes [24]. The coding sequences (CDs) covered 12.5% of the mitogenome.

**Figure 5:**
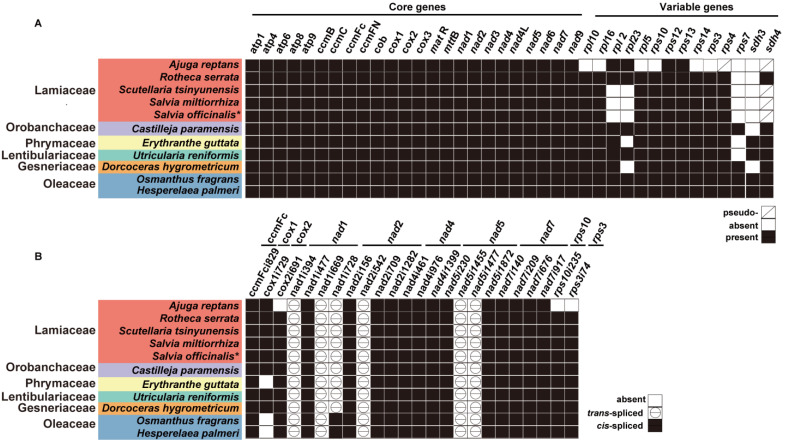
Heatmaps showing the PCG contents (**A**) and intron contents (**B**) in eleven sequenced Lamiales mitogenomes. Each column represents a gene in (**A**) and an intron in (**B**). Each row represents a species. The gene and intron names are shown above the heatmaps. The Latin names for each species and the family they belong to are shown to the left of the heatmap. The introns are named according to a prior scheme [52]. The mitogenome generated in this study was labeled with “*”. The different colored and patterned squares in the figures are explained below (**A**) and to the right of (**B**) the heatmaps.

Furthermore, we compared the gene contents of *S. officinalis* with all its Lamiales relatives (Figure 5A). We found that all the core genes were present. The *Salvia* mitogenomes lost four variable genes: *rpl2*, *rpl23*, *rps7*, and *sdh3*. In addition, the *sdh*4 was found to be a pseudogene as only partial sequences remained. 

The angiosperm mitogenomes contain group I and group II introns [48]. The two types of introns were defined according to their conserved folding structures and splicing mechanisms [49]. These introns were removed by *cis*- or *trans*-splicing to form continuous and functional transcripts [50,51]. In the *S. officinalis* mitogenome, 10 PCGs contained 18 *cis*-spliced introns (Figure 5B). Among them, *nad1*, *ccmFc*, *cox1*, *cox2*, *rps3*, and *rps10* contained one *cis*-spliced intron each. *nad5* contained two *cis*-spliced introns. *nad2* and *nad4* contained three *cis*-spliced introns each. *nad7* contained four *cis*-spliced introns. These introns were named according to a scheme described previously [38,52] and the names of the introns are shown in Figure 5B. Three PCGs (*nad1*, *nad2*, and *nad5*) contained six *trans*-spliced introns in total: *nad1*i394, *nad1*i669, *nad1*i728, *nad2i*542, *nad5i*455, and *nad5i*477 (Figure 5B).

We inferred intron gain/loss polymorphism in the *S. officinalis* mitogenome and the other ten Lamiales mitogenomes, which were introduced in the method of phylogenetic analysis (Section 4.7). The *cox1* intron (*cox1i729*) was detected in eight species, including *S. officinalis*, but was not detected in the three species: *E. guttata*, *O. fragrans,* and *H. palmeri* (Figure 5B). In the eight Lamiales species, the *cox1* intron was highly conserved in terms of its position on the *cox1* gene. The presence/absence polymorphism of *cis*-spliced intron *cox2i691* was also observed. Moreover, *nad1*i728 was *cis*-spliced in the two *Oleaceae* species. Its adjacent exons were *trans*-spliced in the nine Lamiales species (Figure 5B).

### 2.3. Homologous Recombination Mediated by Dispersed Repetitive Sequences

Angiosperm mitogenomes contain three types of dispersed repetitive sequences on the basis of the repeat unit length: larger repetitive sequences (LRs), intermediate repetitive sequences (IntRs), and short repetitive sequences (SRs), with repeat unit lengths of “>1 kb”, “100–1000 bp”, and “<100 bp”, respectively [53]. They are vastly distributed among different plants and contribute to homologous recombination and variable mitogenomic structures [54,55]. To identify the repetitive sequences that might mediate homologous recombination in the *S. officinalis* mitogenome, we conducted a self-to-self sequence comparison using BLASTn with an e-value < 1 × 10^−6^ and a word size of 7, as described previously [46]. The BLASTn results contained 52 high-scoring sequence pairs (HSPs). Prior research confirmed that the repeat unit sequences of the repetitive sequences mediating homologous recombination could be variable. Consequently, we considered all these HSPs as potential repetitive sequences and named them r01–r52 (Table 1 and Table S2).

These 52 repetitive sequences corresponded to 47 SRs and 5 IntRs, ranging from 28 to 892 bp in repeat unit length, with sequence identities ranging from 85.98% to 100% (Table S2). Among these 47 SRs and 5 IntRs, 30 SRs and 2 IntRs were intra-chromosomal, as both repeat units were on MC1 and MC2. By contrast, 20 SRs were inter-chromosomal and the two repeat units were located separately on MC1 and MC2.

**Figure 6:**
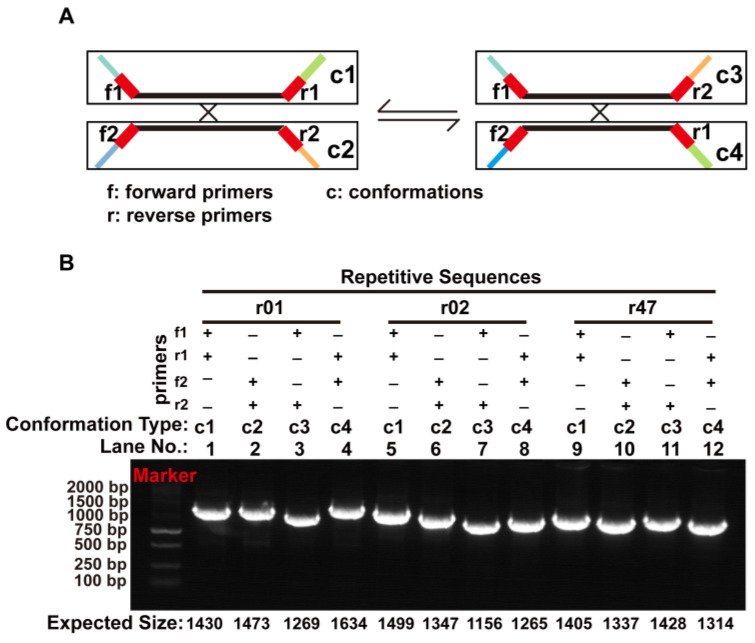
PCR verification of recombination products associated with the repetitive sequences on MC1 and MC2. (**A**) Schematic representation of the four conformations (c1–c4) associated with each repetitive sequence. The regions corresponding to the primers are shown as red blocks. f1 and f2: forward primers. r1 and r2: reverse primers. (**B**) Electrophoretic gel plot of PCR products amplified with various combinations of forward and reverse primers to amplify the DNA molecules corresponding to conformations c1–c4. The name of the repetitive sequence, combinations of forward and reverse primers, expected conformation to be amplified, and lane numbers are shown above the gel plot. The expected size of each PCR product includes the length of the repetitive sequence and the length of its 5′ and 3′ flanking regions (200–1000 bp). We used the length of the PCR products as a rough assessment of the successful amplification of the fragments representing the recombination products.

To investigate if these repetitive sequences can mediate homologous recombination, we extracted the repeat unit sequence along with its 1000 bp long flanking sequences and combined them to form the reference sequences representing the four conformations before and after recombination (Figure 6A). Then, we mapped the Nanopore long reads to the reference sequences representing those before and after recombination. The homologous recombination products of three repetitive sequences (r01, r02, and r47) were supported by the Nanopore long reads. Further examination of these three repetitive sequences showed that they corresponded to DBS01–03, respectively (Figure S1). 

To confirm the presence of the recombination products of r01, r02, and r47, we designed primers that can amplify DNA sequences corresponding to the c1–4 conformations (Table S3). The primers were then used to amplify the genomic DNA (gDNA), and the products were sequenced with the Sanger method. The gel electrophoresis results of the PCR products are shown in Figure 6B. The products corresponding to the c1–c4 recombination product conformations affiliated with r01, r02, and r47 are shown on lanes 1–4, 5–8, and 9–12, respectively. The PCR products were recovered and subjected to Sanger sequencing. The comparison of the Sanger sequencing results and those expected sequences revealed identical sequences (Figure S3).

**Figure 7:**
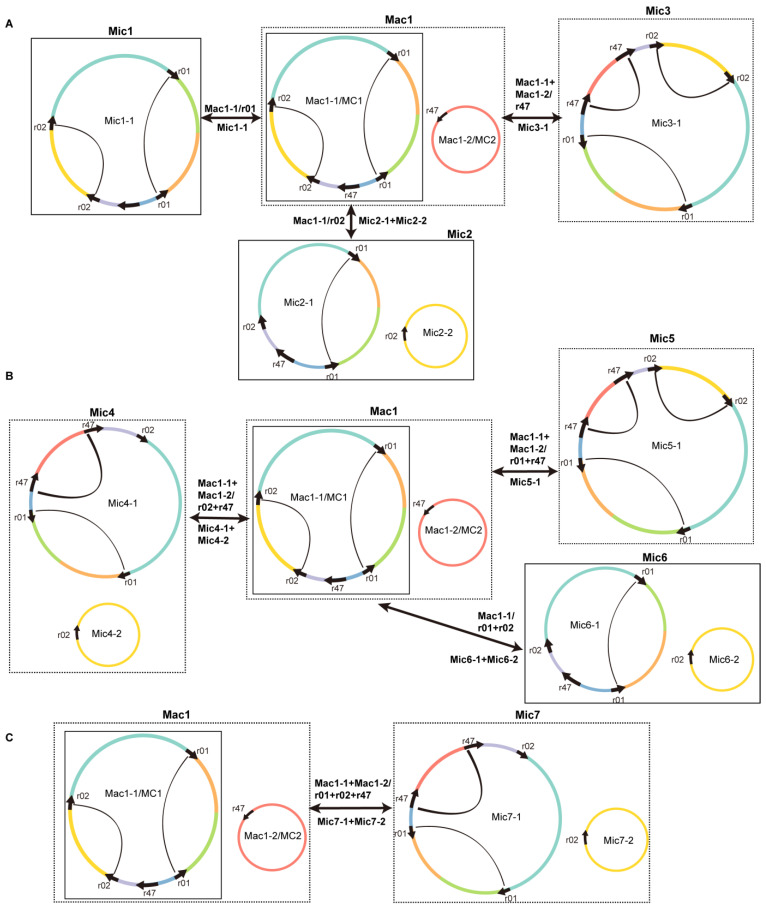
Products of homologous recombination mediated by one (**A**), two (**B**), and three (**C**) of the repetitive sequences r01, r02, and r47. The repeat units of r01, r02, and r47 are represented by arrows. The two repeat units are connected with arcs if they are on the same chromosome. Sequences around the repeat units are shown in different colors. The circles represent circular chromosomes. The genomic conformation is named “c” followed by the conformation number. In contrast, the circular chromosomes of a particular genomic conformation are named “c” followed by the conformation number, “-”, and the chromosome number. The double-headed arrows indicated the source circular chromosomes, the repetitive elements, and the product circular chromosomes, separated with “/”. The circular chromosomes before and after the recombination are shown with the same type of squares: solid or dashed. The genomic conformation name is prefixed with “Ma”, representing “major” if it is the most abundant conformation. Otherwise, the genomic conformation name is prefixed with “Mi”, representing “minor”. Mac1 is the genomic conformation containing chromosomes MC1 (Mac1-1) and MC2 (Mac1-2). (**Panel A**) Mac1-1 can undergo recombination mediated by r01 to form a circular chromosome Mic1-1. Similarly, Mac1-1 can undergo recombination mediated by r02 to form two circular chromosomes: Mic2-1 and Mic2-2. Mac1-1 and Mac1-2 can undergo recombination mediated by r47 to form Mic3-1. Mic3 only contains one circular chromosome Mic3-1. (**Panel B**) Mac1 can undergo recombinations mediated by two of the three repetitive sequences r01, r02, and r47 to form genomic conformations Mic4 to Mic6. (**Panel C**) Lastly, Mac1 can form Mic7 through recombinations mediated by r01, r02, and r47 together. Please note that we consider the Mac1 as the source conformation. Only newly formed circular chromosomes are shown for each newly formed genome conformation. By definition, it should also contain the circular chromosome in the source conformation that does not undergo recombination.

We then drew the structure of the circular molecules that might result from the recombination mediated by the repetitive sequences r01, r02, and r47 (Figure 7). We define genomic conformation as a set of chromosomes that can contain all the genomic contents. As shown in Figure 7A, we defined the genomic conformation containing both MC1 and MC2 as major conformation 1 (Mac1). Then, four circular molecules, namely, Mic1-1, Mic2-1, Mic2-2, and Mic3-1, can be formed after recombination, mediated by r01, r02, and r47 alone, repeatedly. Assuming recombinations mediated by the three repetitive sequences were independent, then various recombination products can be obtained after recombinations mediated by two and three of the repetitive sequences. In Figure 7B, we showed that Mac1 can form Mic4, Mic5, and Mic6 through recombinations mediated by r02 + r47, r01 + r47, and r01 + r02, respectively. In Figure 7C, we showed that Mac1 can form Mic7 through recombinations mediated by all three repetitive sequences. 

It should be noted that the genomic conformation Mic3 had only one circular chromosome, which was traditionally regarded as the master circle. Here, we found that its abundance was lower than that of the Mac1. Therefore, Mac1 was considered the predominant conformation. Note that multiple recombinations mediated by different combinations of repetitive sequences could occur and generate numerous molecules with different conformations.

### 2.4. Tandem Repeats in the Lamiales Mitogenomes

Tandem repeats, also called tandem repetitive sequences, are repetitive sequences whose repeat units are arranged in tandem. Simple sequence repeats (SSRs), or microsatellites, are short tandem repeat sequences in which the repeat units contain 1–6 nucleotides [56]. Tandem repeats in which the repeat units contain ≥7 nucleotides are designated as long tandem repeats. SSRs are involved in species authentication, genetic variation, construction of linkage maps, population phylogenetics, and evolution [57,58]. The importance of tandem repeats was reported in studies on the transcription, translation, and regulation of promoter activities [59,60]. Using the MISA web service and Linux version of the Tandem Repeats Finder (v4.09), we identified 78 SSRs and 9 long tandem repeats (Table S4).

Among the SSRs, 67 and 11 SSRs were located on MC1 and MC2, respectively. The 67 SSRs on MC1 consisted of 5 SSRs in the exonic regions of genes *atp6*, *cob*, *rps3*, and *nad1* (SSR17, SSR18, SSR42, SSR58, and SSR59), and 61 SSRs in the non-exonic regions of the mitogenome (Table S5). One SSR in the MC2 was also located in the exonic regions of gene *atp6* (SSR73). The *S. officinalis* mitogenome is rich in tetranucleotide repeats, which represented 43.28% and 36.36% of all SSRs on MC1 and MC2, respectively. By contrast, trinucleotide repeats were less frequent than their tetranucleotide counterparts and represent 19.40% of all SSRs on MC1. Trinucleotide and hexanucleotide repeats were not found in MC2. The *S. officinalis* mitogenome contained seven (TR1-7) and two (TR8-9) long tandem repetitive sequences on MC1 and MC2 (Table S6), respectively. The length of the repeat units ranged from 12 to 36 nt.

### 2.5. Identification of Mitochondrial Plastid DNA (MTPT)

MTPTs are mitochondrial DNA sequences that are derived from plastomes [61]. To identify MTPTs in the *S. officinalis* mitogenome, we compared the mitogenome and the plastome of *S. officinalis* using BLASTn with an e-value < 1 × 10^−6^ and a word size of 7 [46]. We identified 23 MTPTs in the *S. officinalis* mitogenome, with a total length of 14,495 bp accounting for 4.70% and 9.59% of the *S. officinalis* mitogenome and plastome sequences, respectively. The list of MTPTs is shown in Table S7. A schematic representation of the homologous sequence pairs from the mitogenome and plastome is shown in Figure S4.

The size of the MTPTs ranged from 41 to 4,261 bp. Twenty-one (mtpt01–21) and two MTPTs (mtpt22–23) were found in MC1 and MC2, respectively. The two largest MTPTs were mtpt01 (4261 bp) and mtpt11 (3598 bp), covering the regions of MC1 from positions 32,916 to 37,176 and from 167,076 to 170,673 (Table S7). The mtpt01 contained a fragment of the gene *ycf2* and the 198 bp long, complete CDs of gene *ycf15.* The mtpt11 contained the 468 bp long, complete CDs of the gene *rps7*.

Many complete plastid tRNA genes were also found in the MTPTs, including *trnI*-CAU, *trn*N-GUU, *trn*M-CAU, *trn*H-GUG, *trn*D-GUC, *trn*S-GGA, *trn*P-UGG, and *trn*W-CCA (Table S7). To confirm that these sequences homologous to the plastome sequences are indeed on the mitogenome, we extracted the MTPTs and 2 kb long flanking sequences on each side of the MTPTs as reference sequences. We also mapped the Nanopore long reads to these reference sequences. The presence of long reads spanning the homologous sequences would support the notion that these sequences were indeed MTPTs. The mapping results for mtpt01–23 (Figure S5) confirmed the presence of these MTPTs.

### 2.6. Phylogenetic Analysis

**Figure 8:**
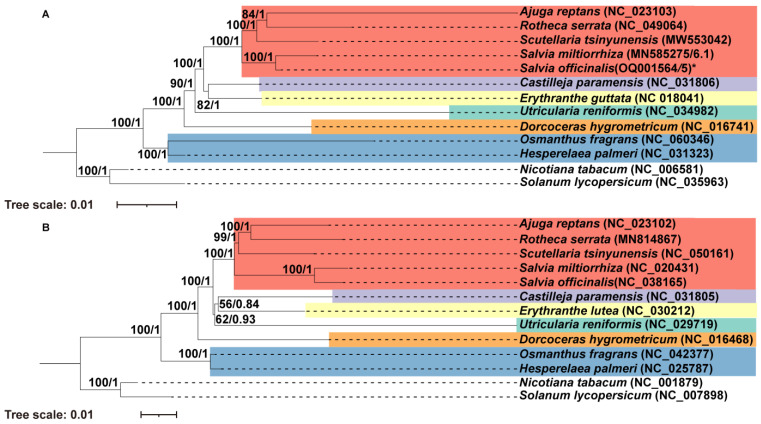
Molecular phylogenetic analysis of *Salvia officinalis* and its 10 close Lamiales relatives and two non-Lamiales species based on mitogenomes (**A**) and plastomes (**B**). The tree was constructed using concatenated conserved protein sequences from the mitogenomes of 11 Lamiales and 2 non-Lamiales species via the ML and BI methods. The bootstrap score was obtained by employing 1000 replicates. The ML bootstrap support values and BI posterior probabilities were labeled at the corresponding nodes. Two species from Solanaceae (*Nicotiana tabacum* and *Solanum lycopersicum*) were used as outgroups. The mitogenome assembled in this study was labeled with an asterisk.

To understand how mitochondrial genomes evolve, we conducted a phylogenetic analysis of 11 Lamiales species and 2 non-Lamiales species as outgroups. First, we extracted the CDs of 26 genes shared by these 13 species, including the 24 core genes (*atp*1, *atp*4, *atp*6, *atp*8, *atp*9, *ccm*B, *ccm*C, *ccm*Fc, *ccm*Fn, *cob*, *cox*1, *cox*2, *cox*3, *mat*R, *mtt*B, *nad*1, *nad*2, *nad*3, *nad*4, *nad*4L, *nad*5, *nad*6, *nad*7, and *nad*9) and 2 variable genes (*rps*12 and *rps*13). The CDs were concatenated and subject to multiple sequence alignment with MAFFT. We constructed a maximum likelihood (ML) tree using the RAxML (v8.2.4) and a Bayesian inference (BI) tree using the MrBayes version (3.2.7) [62]. ML and BI analyses revealed that *S. officinalis* is the sister taxon to *S. miltiorrhiza* with a bootstrap support of 100 and a posterior probability of 1.00 (Figure 8A). Furthermore, we conducted a parallel phylogenetic analysis of the same taxa using the CDs of 56 common plastid PCGs (Figure 8B). The two trees had identical topology, suggesting that the CDs of mitogenomes and plastomes underwent a similar evolutionary process in these Lamiales species.

### 2.7. RNA Editing Site Analysis

RNA editing is a post-transcriptional modification process frequently found in the organelle of high plants [63]. RNA editing plays important roles in protein sequence conservation in the plastids and protein sequence diversification in the mitochondria. RNA editing can affect the composition of the final mitochondrial proteome. Furthermore, RNA editing can create start and stop codons in the mitochondrial mRNA molecules [37] through the so-called start-gain or stop-gain processes [63]. To determine the extent of RNA editing in the *S. officinalis* mitogenome, we first detected the RNA editing sites using the RNA sequencing reads, and 452 sites in the CDs were obtained within the 31 PCGs (Table S8). To exclude sites that might have originated from polymorphic sites, we identified the single nucleotide polymorphism sites in the CDs of the 31 PCGs (Table S9). Comparing the predicted RNA editing sites and the single nucleotide polymorphism (SNP) sites revealed only one overlap which, in turn, was removed from the downstream analysis of RNA editing events.

For the remaining 451 sites, we first ascertained the discrepancies between the DNA and mRNA sequences. We mapped the RNA sequencing reads to the CDs of 31 PCGs and then visualizes the results using the IGV software (v 2.15.1). The results (Figure S6) confirm the RNA editing of all the detected sites. To further confirm the RNA editing of these sites, we randomly selected 11 genes that contained 126 detected RNA editing sites. We designed specific primers (Table S10) to amplify the corresponding gDNA and complementary DNA (cDNA) sequences. The amplicons were then sequenced using the Sanger method. The comparison of the sequences amplified from gDNA and cDNA was presented in Figure S7. In total, 113 (90%) sites were confirmed successfully (Figure S7 and Table S8).

**Figure 9:**
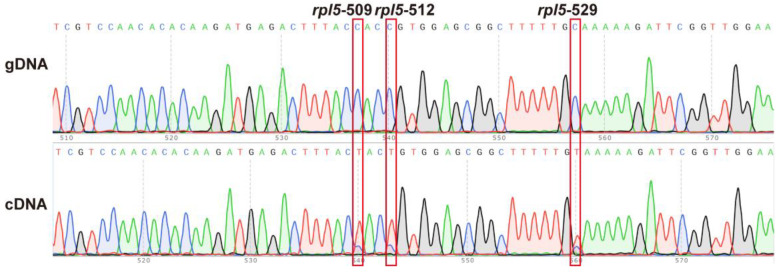
Validation of three predicted RNA editing sites (*rpl5*-509, *rpl5*-512, and *rpl5*-529) using PCR and Sanger sequencing. The top panel shows the sequences and chromatographs from the PCR products amplified using gDNA as the template. The bottom panel shows the sequences and chromatographs from the PCR products amplified using the cDNA. The gene names and the RNA editing sites’ positions are shown at the top of the figure, separated with “-”. The red squares highlight the focal RNA editing site. *rpl5*-529 created a new stop codon upstream from the stop codon found in the genomic sequence.

These 451 sites contained 404 non-synonymous editing sites, significantly more than the 47 synonymous sites in the *S. officinalis* mitogenome (Table S8). Moreover, 57.21% (258 sites), 32.37% (146 sites), and 10.42% (47 sites) editing sites occurred at the first, second, and third codon positions, respectively (Table S8). Specifically, we detected two stop-gain editing sites *rpl5*-529 and *rps10*-307, which created two new stop codons. The *rpl5*-529 editing changed the codon from CAA to the stop codon TAA (Table S8, Figure 9, Figure S6x, and Figure S7i). The *rps10*-307 editing changed the codon CGA to the stop codon TGA (Table S8 and Figure S6C).

## 3. Discussion

Recently, we reported on the mitogenome of *S. miltiorrhiza* on the basis of Pacbio long reads and Illumina short reads [46]. We were the first to confirm that the predominant conformations of the *Salvia* mitogenome are two circular chromosomes. To understand the diversity and evolution of the *Salvia* mitogenomes, we sequenced, assembled, and characterized the mitogenome of *S. officinalis* in detail in the current study. We characterized the gene contents, SSRs, tandem repeats sequences, dispersed repeat sequences, MTPTs, and RNA editing events in the *S. officinalis* mitogenome. Lastly, we used the common genes of 11 Lamiales species to identify the phylogenetic relationship and variation in gene and intron contents within Lamiales. The results from this work provide indispensable information for comprehending the diversity and evolution of *Salvia* mitogenomes.

### 3.1. Architecture of Two Major Circular Chromosomes and Multiple Variable forms for the S. officinalis Mitogenome

The *S. officinalis* mitogenome contains two circular chromosomes of 268,341 and 39,827 bps in length. The total length was 308,168 bps (Figure 1). These multichromosomal structures were also found in a close relative, *S. miltiorrhiza*, which also contained two chromosomes that were 328,915 and 85,199 bps in length. The total length was 414,114 bps [46]. These lines of data suggest that the dominant form of *Salvia* mitogenomes might be two circular chromosomes. With the availability of more *Salvia* mitogenomes, we will be able to test if this two-major-chromosome architecture is conserved.

The presence of alternative structures of plant mitogenomes created by repeat-mediated homologous recombination has been reported previously [54,55]. In the *S. miltiorrhiza* and *Scutellaria tsinyunensis* mitogenomes, repetitive sequences (numbered nine and one) could mediate homologous recombination, which resulted in alternative genomic conformations. We compared the sequences of these repeats and found no sequence similarity between them. Furthermore, the *Mimulus guttatus* mitogenome has eight alternative genomic conformations (C2–C8) predicted through the homologous recombination of three large repeats (R1, R2, and R3). In this study, we identified three repetitive sequences, r01, r02, and r47, in the *S. officinalis* mitogenome that mediated homologous recombination. Seven alternative genomic conformations Mic1–7 were predicted based on the recombination mediated by repeats independently. The results indicate that repeat-mediate recombination is a driving force in a plant mitogenome’s structural diversification.

It should be noted that there were 52 pairs of repetitive sequences in the *S. officinalis* mitogenome, but only 3 pairs were identified to be associated with homologous recombination. There are two putative explanations. Firstly, other repeats have low or no recombination activity. Secondly, due to the high sequencing error rate of Nanopore reads, it is difficult to identify the different copies of repeats, resulting in no observation of recombination products using the methods and parameters used in this study. Additional recombination products might be identified with an improvement in the accuracy of long-read sequencing. The specific observation of recombination products for these three repetitive sequences suggested that the recombination events are context-specific and that the recombination frequencies might result from the repeat sequences and their high-dimensional structures at or around the repeat sequences. However, we believe that we do not have sufficient data to draw further conclusions in the current paper.

The relative copy number of these alternative subgenomic conformations within plant mitochondria was differential [54] and was controlled by nuclear genes [64]. For instance, one nuclear gene (*CHM*) in *Arabidopsis* regulated the genomic shifting process and influenced the abundance levels of subgenomic conformations [64]. The possible involvement of the mechanism of this mitochondrial substoichiometric shifting should be studied in *S. officinalis* in the future.

### 3.2. Intron Contents of the Lamiales Mitogenomes

The intron contents of an angiosperm plant’s mitochondrial genes are conserved in each lineage but are variable among different lineages [38]. The introns’ positions, such as those of *nad7* introns of *Marchantia*, have been used as phylogeny markers [65]. In the present study, we observed the variety of intron contents among different Lamiales lineages. For example, intron loss of the *cox1* gene (*cox1i729*) occurred in two Oleaceae species and one Orobanchaceae species (Figure 5B). By contrast, the *cox1i729* introns were present in the other eight species. In these eight species, the positions of the *cox1i729* were highly conserved.

The origin of the intron *cox1i729* has also been examined in the literature. One study proposed that *cox1i729* was gained by a single horizontal transfer from fungi and subsequently transferred from one angiosperm to another [66]. Another report suggested that the *cox1i729* intron was gained in angiosperms once by largely or entirely vertical transmissions [67]. Five introns (*nad1i394*, *nad1i669*, *nad2i542*, *nad5i1455*, and *nad5i1477*) found in the angiosperm mitochondrial *trans*-spliced genes (*nad1*, *nad2,* and *nad5*) were also identified in the eleven Lamiales species [38]. Furthermore, these *trans*-spliced introns were postulated to have evolved from one common ancestor by fragmentation of a *cis*-spliced arrangement [50].

### 3.3. MTPTs in the S. officinalis Mitogenome

Foreign sequences are commonly found in the angiosperm plants’ mitogenome; in particular, plastome sequences are frequently found in the mitogenomes through intracellular gene transfer (IGT) [68]. In this study, we found 23 MTPTs in the *S. officinalis* mitogenome ranging from 41 to 4261 bp. The total length of MTPTs was 14,495 bp and represented 4.70% of the mitogenome. Previously, 16 MTPTs ranging from 115 to 4987 bp (totaling 12,583 bp) and 19 MTPTs ranging from 45 to 902 bp (totaling 3372 bp) were identified in the *S. miltiorrhiza* and *Scutellaria tsinyunensis* mitogenomes, covering 3.04% and 0.95% of the mitogenome, respectively [46,69]. A higher ratio of the MTPT sequences was gained in other plants, such as a total of 6.3% of plastid sequences in the rice mitogenome [70].

In addition, the largest MTPT of the *S. officinalis* mitogenome (4261 bp) was slightly shorter than the largest MTPT in the *S. miltiorrhiza* mitogenome (4987 bp) but was more than four times longer than the largest MTPT in the *Scutellaria tsinyunensis* mitogenome (902 bp). The long fragments that transferred from plastids were not unique to the *Salvia* mitogenomes as they were also found in the *Physochlaina orientalis* mitogenome, with the largest MTPT being 6593 bp. Most MTPTs in the *S. officinalis*, *S. miltiorrhiza,* and *Scutellaria tsinyunensis* mitogenomes might be genetically inserted and become functionless as fragments of the coding genes. This presumption was also given in other higher plants [68]. The plastome tRNA genes were always intact and present in the *S. officinalis* and *Scutellaria tsinyunensis* mitogenomes. Current data suggest that the MTPTs are quite diverse in terms of length and content within the *S. officinalis* mitogenome.

### 3.4. RNA Editing in the S. officinalis Mitogenome

We also compared the 451 RNA editing sites detected in this work to those in a previous study of *S. miltiorrhiza* and found 193 sites in the homologous sites of the PCGs (Table S8). The stop codon gain site of *rps10*-307 was also conserved in the two mitogenomes. In the future, more mitogenome sequences of *Salvia* are needed to obtain additional information about the conservation of and variation in RNA editing events, and the protein sequences and structures of *Salvia* mitogenomes.

RNA editing events modify transcript site-specific information post-transcriptionally, and mitochondrial RNA editing events have been extensively detected among land plants [63,71,72]. Additionally, RNA editing sites are somewhat conservative in the angiosperm plant mitochondrial genome [73]. In our work, 13 of the 126 RNA editing sites in PCGs were not validated successfully. This outcome might be caused by the following reasons. First, the frequency of RNA editing was low in some of these sites (eight sites had frequencies of RNA editing ranging from 0.13 to 0.44). Second, preferential amplification most likely occurs during PCR. 

Determining the RNA editing landscape is critical to annotate the mitogenome’s proteome. For example, annotation based on the stop codon on the DNA sequences predicted a CD of the gene *rpl5* in *S. officinalis* that is 786 bp in length. By contrast, the homologous *rpl*5 CD is 555 bp long in *S. miltiorrhiza* and 558 bp long in *Arabidopsis thaliana*. However, considering the stop gain, the CDs of the gene *rpl5* in *S. officinalis* became 529 bp in length and were more similar to their homologous sequences in *S. miltiorrhiza* and *Arabidopsis thaliana*. Furthermore, the sequence downstream from the stop gain codon had little similarity to sequences from *S. miltiorrhiza* and *Arabidopsis thaliana*. Taken together, DNA sequences alone are not sufficient to annotate PCG sequences, and RNA editing events must be taken into consideration.

## 4. Materials and Methods

### 4.1. Plant Materials and Nucleic Acid Preparation

Young leaves were collected from an *S. officinalis* line (named so-01) grown at the Institute of Medicinal Plant Development, Chinese Academy of Medical Sciences, Peking Union Medical College, Beijing City (E 116°25′, N 39°47′). *S. officinalis* is not an endangered or protected species, so no specific permissions were required for sample collection. The taxonomic identity of *S. officinalis* was confirmed by Dr. Yaodong Qi of the Institute of Medicinal Plant Development, Chinese Academy of Medical Sciences, Peking Union Medical College, Beijing. The *S. officinalis* plant was photographed by Dr. Xinlei Zhao, Institute of Medicinal Plant Development, Chinese Academy of Medical Sciences, Peking Union Medical College, Beijing (Figure 1). The voucher specimen of *S. officinalis* was deposited at the Institute of Medicinal Plant Development and assigned the accession number so-01-yhy. The *S. officinalis* leaves were kept at −80 °C until use.

For DNA sequencing, a plant genomic DNA kit (Tiangen Biotech, Beijing, Co., Ltd., Bejing, China) and an RNAprep Pure Plant Kit (Tiangen Biotech Beijing Co., Ltd) were used to isolate the total DNA and RNA from the fresh *S. officinalis* leaves, respectively. The DNA and RNA yield and purity were assessed using a Nanodrop spectrophotometer 2000 (Thermo Fisher Scientific, Waltham, MA, USA) and 1.0% agarose gel electrophoresis.

### 4.2. DNA and RNA Sequencing

For short read DNA sequencing, the DNA was first fragmented into 350 bp long fragments using the Covaris S2/E210 ultrasonicator (Covaris Inc., Woburn, MA, USA). The sequencing library was constructed with the NEBNext Ultra DNA Library Prep Kit for Illumina (NEB) according to the manufacturer’s instructions. The library was then subjected to pair-end sequencing (2 × 150 bp reads) on an Illumina HiSeq 2500 sequencer using the standard protocol (Illumina, Inc; San Diego, CA, USA) at Grandomics Biotechnology Co., Ltd. (Wuhan, China).

For long read DNA sequencing, the large DNA fragments (>10 kb) were selected and enriched by the Short Read Eliminator XS kit (Circulomics, Inc., Baltimore, MD, USA). The sequencing library was constructed using the Ligation Sequencing Kit SQK-LSK109 (Oxford Nanopore Technologies, Cambridge, UK) and sequenced on an R9.4.1 flow cell on the MinION sequencer at Grandomics Biotechnology Co., Ltd. The ONT MinKNOW v19.12.5 software was utilized for base calling.

For the total RNA sequencing, the rRNA in the total RNA was removed using a Ribo-Zero^TM^ Magnetic Kit (Epicenter, Madison, WI, USA). The sequencing library was prepared with a VAHTS Universal V8 RNA-seq Library Prep Kit (Vazyme, Nanjing, China) according to the manufacturer’s instructions. The prepared library was sequenced on an Illumina HiSeq 2500 sequencer (2 × 150 bp reads) at Grandomics Biotechnology Co., Ltd.

### 4.3. Mitogenome Assembly and Annotation

To acquire the complete sequence and uncover the possible structures of the mitogenome of *S. officinalis* (so-01), we performed a hybrid assembly strategy. We extended the mitochondrial short reads using the GetOrganelle (v1.6.4) toolkit. Subsequently, the GetOrganelle-extended reads were de novo assembled into a unitig graph using the SPAdes software packaged in the Unicycler (v0.4.9) [74]. Lastly, the DBSs in the unitig graph were resolved using the Unicycler with the Nanopore long reads.

To verify that the DBSs were resolved correctly, we extracted the repeat sequence and its 1000 bp long flanking sequences and combined them to form the reference sequences for the four conformations. The Nanopore long reads were then mapped to the DBSs of the reference sequences using the BWA (v0.7.12-r1039) [75] with default parameters.

Subsequently, the Nanopore long reads and the Illumina short reads were mapped back to the complete sequences of mitogenome using the BWA (v0.7.12-r1039) [75] with default parameters. The coverage depth of the long and short reads mapped to the *S. officinalis* mitogenome sequences was obtained using samtools (v1.3.1) [76]. 

For mitogenome annotation, we used a new custom program: MGA (http://www.1kmpg.cn/mga (accessed on 10 November 2022)). Finally, we carefully checked the start and stop codon positions and intron/exon boundaries of each gene using the program Apollo [77]. Maps of the circular *S. officinalis* mitogenome were drawn using PMGView (http://www.1kmpg.cn/pmgview (accessed on 10 November 2022)). The mitogenome sequences of *S. officinalis* were deposited in GenBank under the accession numbers OQ001564 and OQ001565.

For the collinear analysis of the *Salvia* mitogenomes, we used the Nucmer module in Mummer (v3) [47] with the parameters used in a previous report [78]. Briefly, the identity threshold was set to 85% and the alignment mode was many-to-many. The results of the collinear analysis were visualized using RIdeogram in the R package [79].

To identify the presence/absence of polymorphisms in the introns’ contents, we checked the intron contents of ten Lamiales species using MGA (http://www.1kmpg.cn/mga (accessed on 15 November 2022)). We named the introns following the scheme proposed by a previous report [52], which denoted each intron according to the positions of its orthologous genes in *Marchantia polymorpha*.

### 4.4. Analysis of the Homologous Recombination

To identify the repetitive sequences in the *S. officinalis* mitogenome that might mediate homologous recombination, we conducted a self-to-self comparison of the mitogenome using BLASTn (v 2.10.1+) with the parameters e-value < 1 × 10^−6^ and word_size = 7, as described previously [46]. To detect the possible recombination products of these homologous sequences, we extracted the homologous sequences and their 1000 bp long flanking sequences and combined them to form the reference sequences corresponding to the recombination products. Subsequently, we mapped the Nanopore long reads to these reference sequences and counted the numbers of reads spanning the homologous sequences.

To validate the presence of the homologous recombination products supported by the Nanopore long reads, we designed primers according to the corresponding reference sequences using Primer-BLAST [80]. PCR amplification was conducted for a total volume of 25 µL that contained 12 µL of 2 × Taq PCR Master Mix (TransGen Biotech, Beijing, China), 11 µL of water, 0.5 µL of each primer, and 1 µL of DNA. We performed the PCR reactions on a Pro-Flex PCR system (Applied Biosystems, Waltham, MA, USA) under the following conditions: denaturation at 94 °C for 2 min; then 35 cycles of 94 °C for 30 s, 57 °C for 30 s, and 72 °C for 60 s; and extension at 72 °C for 2 min. We visualized the PCR amplicons with 1.0% agarose gel electrophoresis and sequenced the PCR amplicons using the Sanger sequencing technology at SinoGenoMax Co., Ltd. (Beijing, China).

### 4.5. SSRs and Tandem Repeats Analysis

To analyze the SSRs in the *S. officinalis* mitogenome, we used the MISA web service [56], with 10 as the number of mononucleotide repeat units; 5 as the number of dinucleotide repeat units; 4 as the number of trinucleotide repeat units; and 3 as the number of tetra-, penta-, and hexanucleotide repeat units. The tandem repeats were determined using Tandem Repeats Finder (v 4.09) [81] with the command line “trf mitogenome.fasta 2 7 7 80 10 50 500”. The parameter settings were 2 for matches; 7 for mismatches and indels; and 50 and 500 for the minimum alignment score and maximum period size, respectively.

### 4.6. Identification of Mitochondrial Plastid DNA (MTPT)

We compared the sequences of the mitogenome and plastome (NC_038165.1) using BLASTn (v 2.10.1+) with an e-value < 1 × 10^−6^ and a word size of 7 [46]. All hits were annotated to check the genes located in the MTPTs. Graphic representations of MTPTs in the mitogenome and plastome were created using TBtools (v 1.076) [82]. To validate the presence of MTPTs, we extracted the MTPTs and their 2000 bp long flanking regions on each side as reference sequences. We then mapped the Nanopore long reads to these reference sequences with BWA-MEM [83]. The number of long reads that spanned the MTPTs was counted. We utilized Integrative Genomics Viewer (IGV) software (v 2.15.1) [84] to visualize the reads mapped to the reference sequences.

### 4.7. Phylogenetic Analysis of the 10 Lamiales Species Based on Common Mitochondrial Protein Sequences

For the phylogenetic analysis, we retrieved the mitogenome sequences from *S. officinalis* (OQ001564 and OQ001565), ten Lamiales species, and two outgroup species. The ten Lamiales mitogenomes include *Ajuga reptans* (NC_023103.1), *Rotheca serrata* (NC_049064.1), *Scutellaria tsinyunensis* (MW553042.1), *S. miltiorrhiza* (NC_023209.1), *Erythranthe lutea* (NC_018041.1), *Castilleja paramensis* (NC_031806.1), *Utricularia reniformis* (NC_034982.1), *Dorcoceras hygrometricum* (NC_016741.1), *Osmanthus fragrans* (NC_060346.1), and *Hesperelaea palmeri* (NC_031323.1). The two outgroup species were from the order Solanales: *Nicotiana tabacum* (NC_006581.1) and *Solanum lycopersicum* (NC_035963.1).

The CDs of the 26 PCGs (*atp*1, *atp*4, *atp*6, *atp*8, *atp*9, *ccm*B, *ccm*C, *ccm*Fc, *ccm*Fn, *cob*, *cox*1, *cox*2, *cox*3, *mat*R, *mtt*B, *nad*1, *nad*2, *nad*3, *nad*4, *nad*4L, *nad*5, *nad*6, *nad*7, *nad*9, *rps*12, and *rps*13) present in all these thirteen mitogenomes were extracted using PhyloSuite (v1.2.1) [85]. The sequences of each gene were aligned with MAFFT (v7.450) [86] and concatenated into a data matrix using PhyloSuite. The ML tree was constructed using RAxML (v8.2.4) [87] with the parameters “raxmlHPC-PTHREADS-SSE3-f a-N 1000-m PROTGAMMACPREV-x 551314260-p 551314260-o Nicotiana_tabacum, Solanum_lycopersicum-T 20.” The bootstrap support values of each branch in the phylogenetic tree were generated based on 1000 replicates. The BI of the phylogeny was performed using MrBayes (v3.2.7) [62] according to the appropriate model (TVM+I+G), and the parameters were determined by jMdoleTest (v2.1.0) [88]. All resulting trees were visualized with iTOL viewer (https://itol.embl.de (accessed on 20 November 2022)).

The CDs of the 56 common PCGs (*atpI, atpF, rps15, atpE, rpl22, rpl16, psbK, psbF, petD, rps3, petA, psaC, rpl14, clpP, rbcL, rps2, rps16, psbH, petL, atpA, rpoB, psbJ, petN, rpl20, rps11, ycf4, accD, rpl2, psbA, psbM, rps4, psbD, rpoC2, petG, matK, rpoA, petB, ycf1, rpl23, psbL, rps8, ycf3, psbC, psbN, rps18, ycf2, rps14, rpl33, atpH, psbE, rpl36, psaB, psbT, psaA, rps7,* and *rpoC1*) of plastomes of thirteen species—*Ajuga reptans* (NC_023102.1), *Rotheca serrata* (MN814867), *Scutellaria tsinyunensis* (NC_050161.1), *S. miltiorrhiza* (NC_023431.1), *S. officinalis* (NC_038165.1), *Erythranthe lutea* (NC_030212.1), *Castilleja paramensis* (NC_031805.1), *Utricularia reniformis* (NC_029719.1), *Dorcoceras hygrometricum* (NC_016468.1), *Osmanthus fragrans* (NC_042377.1), *Hesperelaea palmeri* (NC_025787.1), *Nicotiana tabacum* (NC_001879.1), and *Solanum lycopersicum* (NC_007898.1)—were subjected to phylogenetic analysis by utilizing the same methods employed for the mitogenome.

### 4.8. Detection of RNA Editing Sites

We extracted the CDs of each PCG with the 100 bp long flanking regions as reference sequences. We then mapped the strand-specific RNA-seq reads to these reference sequences using HISAT2 (v 2.2.1) [89] with the parameters “--rna-strandness RF--sensitive --no-mixed--no-discordant” [90]. We subsequently used REDItools (v 2.0) [91] with the parameters coverage ≥ 5 and frequency ≥ 0.1 to detect the RNA editing sites [43]. Lastly, we employed IGV software (v 2.15.1) [84] to visualize the mapping results at the RNA editing sites with the minor variant frequency ≥ 0.1.

To detect SNP sites, we mapped the DNA sequencing reads to the reference sequences extracted above using BWA (v0.7.12-r1039) [75] with default parameters, as described before [52,90]. The SNP sites were identified with REDItools (v 2.0) by employing the following parameters: coverage ≥ 5 and frequency ≥ 0.1.

To validate the RNA editing sites detected according to the RNA-seq data, we randomly selected eleven PCGs for PCR amplification and Sanger sequencing of the PCR products. The genomic DNA was the one extracted in the DNA sequencing experiment. To obtain the cDNA, the RNA extracted in the RNA sequencing experiment was subjected to reverse transcription reactions in a 20 μL reaction system containing 1 μg of total RNA, random primers, and 200 U of SuperScript III Reverse Transcriptase (TransGen Biotech, Beijing, China). We utilized the CDs of the selected PCGS as templates and designed primers using the Primer-BLAST. Then, the genomic DNA and cDNA of so-01 were used as templates for PCR amplification under the same conditions described above. The PCR products were subjected to Sanger sequencing at SinoGenoMax Co., Ltd. (Beijing, China).

## 5. Conclusions

We found that the predominant conformation of the *S. officinalis* mitogenome is two circular chromosomes. Recombination mediated by one of the three repetitive sequences r01, r02, and r47 could generate three minor conformations consisting of four circular chromosomes. The mitogenome structures are highly diverse between *S. officinalis* and *S. miltiorrhiza*. We identified more than 400 RNA editing sites, including two that created stop codons. A phylogenomic analysis using mitogenome and plastome CDs resulted in an identical phylogenetic relationship, suggesting that the genomes of the mitochondria and plastid of *Salvia* species underwent a similar evolutionary process.

## Figures and Tables

**Table 1:** Mapping results for Nanopore long reads to the four possible conformations associated with three DBS (DBS01–03), which correspond to high-scoring sequence pairs (HSPs) r01, r02, and r47 described in Section 2.3. DBS: double-bifurcation structure. MC1/2: mitogenome chromosome 1/2. The percentage of minor DBS conformations was calculated as the number of reads mapped to the conformations having fewer mapped reads divided by those mapped to all conformations. ^a^ Validated successfully with PCR; ^b^ all positions are based on MC1, except those marked with MC2.

The ID of the HSP	The ID of the DBS ^a^	Identity (%)	Alignment Length	No. of Mismatches	No. of Gap Openings	Positions of Repeat Copy 1 ^b^		Positions of Repeat Copy 2		E-Value	Type	Number of Long Reads Mapped to Each DBS Conformation				Percentage of Minor DBS Conformation (%)
						Start	End	Start	End			c1	c2	c3	c4	
r01 ^a^	DBS01	100	227	0	0	230,814	231,040	47,101	46,875	2.46 × 10^−116^	Inverted	23	33	4	1	8.93%
r02 ^a^	DBS02	100	200	0	0	137,561	137,760	85,306	85,505	2.52 × 10^−101^	Direct	14	26	3	3	15.00%
R47 ^a^	DBS03	100	892	0	0	66,042	66,933	25,340 (MC2)	24,449 (MC2)	0	Direct	10	19	3	3	20.69%

**Table 2:** Gene contents in the mitogenome of *S. officinalis*.

Group of Genes		Name of Genes
Core genes	ATP synthase	*atp*1, *atp*4, *atp*6 *, *atp*8, *atp*9
	Cytochrome c biogenesis	*ccm*B, *ccm*C, *ccmFc* ^a^, *ccmFn*
	Ubichinol cytochrome c reductase	*cob*
	Cytochrome c oxidase	*cox*1 ^a^, *cox*2 ^a^, *cox*3
	Maturases	*mat*R
	Transport membrane protein	*mtt*B
	NADH dehydrogenase	*nad*1 ^c^, *nad*2 ^c^, *nad*3, *nad*4 ^b^, *nad*4L, *nad*5 ^c^, *nad*6, *nad*7 ^b^, *nad*9
Variable genes	Ribosomal protein large subunit	*rpl*5, *rpl*10, *rpl*16
	Ribosomal protein small subunit	*rps*3 ^a^*, rps*4, *rps*10 ^a^, *rps*12, *rps*13, *rps*14
rRNA genes	Ribosomal RNA	*rrn*5, *rrn*18, *rrn*26
tRNA genes	Transfer RNA	*trn*C-GCA, *trn*D-GUC, *trn*E-UUC, *trn*F-GAA, *trn*G-GCC, *trn*H-GUG, *trn*I-CAU, *trn*K-UUU, *trn*M-CAU, *trn*N-GUU, *trn*P-UGG, *trn*P-UGG, *trn*Q-UUG, *trn*S-UGA, *trn*S-GCU, *trn*W-CCA, *trn*Y-GUA

“^a^”, “^b^”, and “^c^”: genes with two, four, and five exons, respectively. “*”: genes with two copies.

## Data Availability

The raw sequencing data from the Illumina and Nanopore platforms generated during this study are available in GenBank. The associated BioProject, BioSample, and SRA numbers are PRJNA908250, SAMN32033742, and SRR22727863 for the Illumina sequencing reads and SRR22728000 for the Nanopore sequencing reads. The mitogenome sequences were released into GenBank with the following accession numbers: OQ001564 and OQ001565. The plant samples are stored at the Herbarium of the Institute of Medicinal Plant Development, Beijing, China (voucher numbers: so-01-yhy).

## Supplementary Materials

The following supporting information can be downloaded at https://www.mdpi.com/article/10.3390/ijms24065372/s1.
