# Supplementary material for: Mitochondrial Genome Sequence of Salvia officinalis (Lamiales: Lamiaceae) Suggests Diverse Genome Structures in Cogeneric Species and Finds the Stop Gain of Genes through RNA Editing Events
Source: Int J Mol Sci. 2023 Mar 11;24(6):5372. doi: 10.3390/ijms24065372 (PMC10048906; doi:10.3390/ijms24065372)

SUPPLEMENTARY MATERIALS

Mitochondrial Genome Sequence of *Salvia officinalis* (Lamiales: Lamiaceae) Suggests Diverse Genome Structures in Cogeneric Species and Finds the Stop Gain of Genes through RNA Editing Events

Heyu Yang [1,2], Haimei Chen [2], Yang Ni [2], Jingling Li [2], Yisha Cai [2], Jiehua Wang [1,*] and Chang Liu [2,*]

1 School of Environmental Science and Engineering, Tianjin University, Tianjin 300072, China
2 Institute of Medicinal Plant Development, Chinese Academy of Medical Sciences, Peking Union Medical College, Beijing 100193, China
* Correspondence: jiehuawang@tju.edu.cn (J.W.); cliu@implad.ac.cn (C.L.); Tel.: +86-022-8740-2072 (J.W.); +86-10-5783-3111 (C.L.); Fax: +86-022-2740-7956 (J.W.); +86-10-62899715 (C.L.)

Table S1 Statistic summary of the sequencing data generated by Nanopore and Illumina platforms.

Sequencing descriptors	Sequencing platform	
	Nanopore	Illumina
Total number of nucleotides (raw data, bp)	11,326,908,802	16,380,908,800
Total Number of Reads	505,210	163,809,088
Mean Read Length (bp)	22,420	100
Total Number of Mapped Reads	23,959 (MC1), 13,431(MC2)	3,291,507 (MC1), 1,021,009(MC2)
Average Coverage Depth	327.77 (MC1), 396.13 (MC2)	969.556 (MC1), 903.15 (MC2)

Table S2 Results for mapping Nanopore long reads to the four possible conformations associated with three HSPs (r01–r52). “MC1/2”: mitogenome chromosome 1/2. The recombination frequency was calculated as the number of reads mapped to conformations having less number of mapped reads divided by that mapped to all four conformations.

ID of the HSP	Query sequence	Subject sequence	Identity (%)	Alignment Length	Numbers of Mismatches	Numbers of Gap openings	Positions of Repeat Copy 1		Positions of Repeat Copy 2		E-value	Type	Numbers of Long Reads Mapped to Each Conformation				Recombination frequency (%)
							Start	End	Start	End			c1	c2	c3	c4	
r03	MC1	MC1	87.861	173	15	6	190502	190671	94435	94266	1.26E-49	inverted	15	32	0	0	0
r04	MC1	MC1	100	80	0	0	226961	227040	28759	28838	1.28E-34	direct	23	26	0	0	0
r05	MC1	MC1	97.5	80	2	0	166752	166831	29701	29780	2.78E-31	direct	0	7	0	0	0
r06	MC1	MC1	94.937	79	1	1	221621	221696	28709	28631	2.8E-26	inverted	26	26	0	0	0
r07	MC1	MC1	98.507	67	1	0	181259	181325	82141	82075	1.01E-25	inverted	20	13	1	0	0
r08	MC1	MC1	100	61	0	0	253935	253995	237908	237848	4.68E-24	inverted	17	29	1	0	0
r09	MC1	MC1	98.387	62	1	0	222237	222298	183614	183675	6.06E-23	direct	17	19	0	0	0
r10	MC1	MC1	93.243	74	4	1	242683	242756	29375	29303	2.18E-22	inverted	15	26	0	0	0

r11	MC1	MC1	100	53	0	0	58352	58404	47046	47098	1.31E-19	direct	20	23	0	0	0
r12	MC1	MC1	100	53	0	0	230817	230869	58404	58352	1.31E-19	inverted	15	20	0	0	0
r13	MC1	MC1	100	52	0	0	81794	81845	38213	38162	4.71E-19	inverted	20	31	0	0	0
r14	MC1	MC1	100	46	0	0	234854	234899	45948	45993	1.02E-15	direct	25	37	0	0	0
r15	MC1	MC1	97.959	49	0	1	227788	227835	160305	160353	3.67E-15	direct	17	20	0	0	0
r16	MC1	MC1	91.379	58	5	0	138505	138562	65553	65610	4.75E-14	direct	18	28	0	0	0
r17	MC1	MC1	97.727	44	1	0	149621	149664	261610	261567	6.14E-13	inverted	25	28	0	0	0
r18	MC1	MC1	100	40	0	0	171082	171121	19296	19335	2.21E-12	direct	21	25	0	0	0
r19	MC1	MC1	100	40	0	0	182355	182394	91077	91116	2.21E-12	direct	12	22	0	0	0
r20	MC1	MC1	95.349	43	2	0	98250	98292	27395	27353	1.03E-10	inverted	18	26	0	0	0
r21	MC1	MC1	100	37	0	0	128825	128861	126675	126711	1.03E-10	direct	26	23	0	0	0
r22	MC1	MC1	91.667	48	3	1	179901	179948	68020	67974	1.33E-09	inverted	20	13	0	0	0
r23	MC1	MC1	100	35	0	0	86048	86082	85931	85965	1.33E-09	direct	3	16	0	0	0

r24	MC1	MC1	100	35	0	0	188677	188711	129203	129237	1.33E-09	direct	12	19	0	0	0
r25	MC1	MC1	90.196	51	4	1	67974	68023	179948	179898	1.33E-09	inverted	16	21	0	0	0
r26	MC1	MC1	100	34	0	0	186062	186095	59133	59100	4.78E-09	inverted	7	24	0	0	0
r27	MC1	MC1	97.222	36	1	0	110507	110542	27781	27746	1.72E-08	inverted	29	36	0	0	0
r28	MC1	MC1	97.222	36	1	0	135779	135814	126679	126644	1.72E-08	inverted	16	27	0	0	0
r29	MC1	MC1	94.872	39	2	0	236663	236701	166693	166731	1.72E-08	direct	26	2	0	0	0
r30	MC1	MC1	100	30	0	0	189917	189946	58633	58604	0.0000008	inverted	11	25	0	0	0
r31	MC1	MC1	100	29	0	0	96839	96867	95259	95287	0.00000288	direct	17	24	0	0	0
r32	MC1	MC2	100	90	0	0	28822	28911	6978	7067	3.54E-40	direct	23	18	0	0	0
r33	MC1	MC2	97.674	86	2	0	26796	26881	12509	12424	1.28E-34	direct	20	14	0	0	0
r34	MC1	MC2	98.611	72	1	0	138426	138497	12641	12570	1.67E-28	direct	20	15	0	0	0
r35	MC1	MC2	95.062	81	0	1	164744	164824	34518	34594	2.16E-27	direct	50	23	0	0	0
r36	MC1	MC2	85.981	107	11	2	26755	26857	7617	7511	1.68E-23	direct	25	19	0	0	0

r37	MC1	MC2	100	46	0	0	145108	145153	4177	4132	1.02E-15	direct	30	19	0	0	0
r38	MC1	MC2	97.959	49	1	0	96745	96793	5625	5577	1.02E-15	direct	33	7	0	0	0
r39	MC1	MC2	85.897	78	7	2	58387	58460	26434	26357	4.75E-14	direct	30	12	0	0	0
r40	MC1	MC2	100	40	0	0	116473	116512	35503	35464	2.21E-12	direct	28	14	0	0	0
r41	MC1	MC2	100	37	0	0	230706	230742	26330	26366	1.03E-10	direct	8	17	0	0	0
r42	MC1	MC2	95.349	43	1	1	143045	143086	8716	8674	3.7E-10	direct	21	17	0	0	0
r43	MC1	MC2	91.489	47	2	1	177500	177544	7245	7199	4.78E-09	direct	17	19	0	0	0
r44	MC1	MC2	100	31	0	0	107017	107047	36623	36653	0.000000222	direct	23	23	0	0	0
r45	MC1	MC2	100	29	0	0	65553	65581	12519	12491	0.00000288	direct	28	16	0	0	0
r46	MC1	MC2	90.244	41	4	0	203011	203051	20322	20362	0.00000288	direct	30	18	0	0	0
r48	MC1	MC2	95.833	48	2	0	138595	138548	7487	7534	2.53E-14	direct	11	7	0	0	0
r49	MC1	MC2	94.286	35	0	2	65592	65558	26415	26447	0.00000154	direct	14	24	0	0	0
r50	MC1	MC2	96.774	31	1	0	128321	128291	21798	21828	0.00000154	direct	20	22	0	0	0

r51	MC1	MC2	100	28	0	0	138544	138517	26415	26442	0.00000154	direct	13	18	0	0	0
r52	MC2	MC2	96.226	53	2	0	124448	125000	7511	7563	4.21E-17	direct	2	24	0	0	0

Table S3 PCR primers used to detect the homologous recombination of the three repeats in the *S. officinalis* mitogenome.

Primer Name	Corresponding DBS ID	Corresponding Repetitive sequence ID	Primer Sequence (5’->3’)
saof-r01-F1	DBS01	r01	TGGCAACAACCAGAATGAAA
saof-r01-R1	DBS01	r01	CGCTATTCAGGCCGTTACTC
saof-r01-F2	DBS01	r01	AATCAACCGAAGAGGGGACT
saof-r01-R2	DBS01	r01	CACCTCTTCTTTTGCCTTCG
saof-r02-F1	DBS02	r02	CGCTCTCTCAGACGTTTCCA
saof-r02-R1	DBS02	r02	CGATTTCGTTCCGCTGCTAC
saof-r02-F2	DBS02	r02	GGTTGGCATTGGGGAACTCT
saof-r02-R2	DBS02	r02	TTGGGCTTTGCTCTTACCGA
saof-r03-F1	DBS03	r47	AGATACCTTCCCCCGGACTT
saof-r03-R1	DBS03	r47	CAATGGACTATGCCTGCTGC
saof-r03-F2	DBS03	r47	CCCTTAGCTTTTGGCGTTCG
saof-r03-R2	DBS03	r47	GGAAGAGCGGAGCAGTCAAA

Table S4 Simple sequence repeats in the *S. officinalis* mitogenome. MC1/2: mitogenome chromosome 1/2.

Chromosomes	Repeat Unit	Number of Repeat Units										
		3	4	5	6	7	8	9	10	11	12	Total
MC1	A/T	-	-	-	-	-	-	-	2	1	1	4
MC1	AC/GT	-	-	1		-	-	-	-	-	-	1
MC1	AG/CT	-	-	6	2	-	-	-	-	-	-	8
MC1	AT/AT	-	-	2	-	-	-	-	-	-	-	2
MC1	AAG/CTT	-	5	1	-	-	-	-	-	-	-	6
MC1	AAT/ATT	-	2	2	-	-	-	-	-	-	-	4
MC1	ACT/AGT	-	3	-	-	-	-	-	-	-	-	3
MC1	AAAC/GTTT	1	-	-	-	-	-	-	-	-	-	1
MC1	AAAG/CTTT	14	-	-	-	-	-	-	-	-	-	14
MC1	AAAT/ATTT	1	-	-	-	-	-	-	-	-	-	1
MC1	AACC/GGTT	1	-	-	-	-	-	-	-	-	-	1
MC1	AAGC/CTTG	1	-	-	-	-	-	-	-	-	-	1
MC1	AATG/ATTC	3	-	-	-	-	-	-	-	-	-	3
MC1	ACCG/CGGT	1	-	-	-	-	-	-	-	-	-	1
MC1	AGAT/ATCT	1	-	-	-	-	-	-	-	-	-	1
MC1	AGCC/CTGG	1	-	-	-	-	-	-	-	-	-	1
MC1	AGCG/CGCT	1	-	-	-	-	-	-	-	-	-	1
MC1	AGCT/AGCT	1	-	-	-	-	-	-	-	-	-	1
MC1	AGGC/CCTG	1	-	-	-	-	-	-	-	-	-	1
MC1	AGGG/CCCT	1	-	-	-	-	-	-	-	-	-	1
MC1	CCGG/CCGG	1	-	-	-	-	-	-	-	-	-	1
MC1	AAAAG/CTTTT	1	-	-	-	-	-	-	-	-	-	1
MC1	AAAGG/CCTTT	1	-	-	-	-	-	-	-	-	-	1
MC1	AAGAG/CTCTT	1	-	-	-	-	-	-	-	-	-	1
MC1	AAGCT/AGCTT	1	-	-	-	-	-	-	-	-	-	1
MC1	AATAC/ATTGT	1	-	-	-	-	-	-	-	-	-	1
MC1	ACTAG/AGTCT	1	-	-	-	-	-	-	-	-	-	1
MC1	AAATAT/ATATTT	1	-	-	-	-	-	-	-	-	-	1
MC1	AAATGT/ACATTT	1	-	-	-	-	-	-	-	-	-	1
MC1	AACTCT/AGAGTT	1	-	-	-	-	-	-	-	-	-	1
MC1	AATAGT/ACTATT	1	-	-	-	-	-	-	-	-	-	1
MC2	A/T	-	-	-	-	-	-	-	3	-	-	3
MC2	AG/CT	-	-	2	-	-	-	-	-	-	-	2
MC2	AT/AT	-	-	1	-	-	-	-	-	-	-	1
MC2	AAAG/CTTT	1		-	-	-	-	-	-	-	-	1
MC2	AAAT/ATTT	1		-	-	-	-	-	-	-	-	1
MC2	ACTC/AGTG	1		-	-	-	-	-	-	-	-	1
MC2	AGAT/ATCT	1		-	-	-	-	-	-	-	-	1
MC2	AATAT/ATATT	1		-	-	-	-	-	-	-	-	1

Table S5 Detailed information of the simple sequence repeats in the *S. officinalis* mitogenome. MC1/2: mitogenome chromosome 1/2. The simple sequence repeats in the exonic regions of genes are highlighted with asterisks.

Chromosome	ID	Start	End	Repeat type	Repeat unit
MC1	SSR1	2062	2073	tetra-	(ATTC)3
MC1	SSR2	9248	9259	mono-	(A)12
MC1	SSR3	15954	15965	tetra-	(TTTC)3
MC1	SSR4	22901	22915	penta-	(AAGAG)3
MC1	SSR5	27147	27158	tetra-	(TGCC)3
MC1	SSR6	29637	29648	di-	(CT)6
MC1	SSR7	29887	29896	di-	(CT)5
MC1	SSR8	41762	41773	tetra-	(GGTC)3
MC1	SSR9	43093	43107	tri-	(AAT)5
MC1	SSR10	47505	47516	tetra-	(CTTT)3
MC1	SSR11	49986	49997	tri-	(AGA)4
MC1	SSR12	51577	51588	tetra-	(CCAA)3
MC1	SSR13	53213	53224	tetra-	(AAGA)3
MC1	SSR14	57328	57339	tri-	(GAA)4
MC1	SSR15	60765	60774	di-	(TG)5
MC1	SSR16	63084	63093	di-	(TA)5
MC1	SSR17*	66156	66167	tetra-	(TCTT)3
MC1	SSR18*	72382	72396	penta-	(CTTTT)3
MC1	SSR19	74363	74377	tri-	(TTC)5
MC1	SSR20	81956	81967	tetra-	(GCTA)3
MC1	SSR21	83825	83842	hexa-	(TTAGAG)3
MC1	SSR22	84906	84917	tri-	(GAA)4
MC1	SSR23	91667	91681	penta-	(GAAAG)3
MC1	SSR24	92866	92877	tetra-	(AAGA)3
MC1	SSR25	97264	97281	hexa-	(TTTATA)3
MC1	SSR26	104007	104018	tri-	(TTA)4
MC1	SSR27	105009	105018	di-	(GA)5
MC1	SSR28	113445	113459	penta-	(AGCTA)3
MC1	SSR29	124448	124459	tetra-	(GAAA)3
MC1	SSR30	132713	132724	tetra-	(CCTC)3
MC1	SSR31	135514	135525	tri-	(AGT)4
MC1	SSR32	138876	138893	hexa-	(CTATTA)3
MC1	SSR33	142826	142837	tetra-	(GCTC)3
MC1	SSR34	147535	147546	tri-	(CTT)4
MC1	SSR35	148000	148011	tetra-	(GAAA)3
MC1	SSR36	148634	148645	tetra-	(AAAG)3
MC1	SSR37	152086	152095	di-	(CT)5
MC1	SSR38	153889	153900	tri-	(AGA)4
MC1	SSR39	165316	165327	tetra-	(TTTC)3

MC1	SSR40	172992	173003	tetra-	(CATT)3
MC1	SSR41	178918	178932	tri-	(TAT)5
MC1	SSR42*	184739	184750	tetra-	(CATT)3
MC1	SSR43	194172	194182	mono-	(T)11
MC1	SSR44	202376	202387	di-	(AG)6
MC1	SSR45	204811	204822	tri-	(CTA)4
MC1	SSR46	205986	205997	tetra-	(TTTG)3
MC1	SSR47	209291	209302	tetra-	(TAAA)3
MC1	SSR48	212121	212132	tri-	(ACT)4
MC1	SSR49	214541	214550	di-	(AG)5
MC1	SSR50	215994	216008	penta-	(CAATA)3
MC1	SSR51	216372	216383	tetra-	(TTTC)3
MC1	SSR52	217234	217245	tetra-	(CTGG)3
MC1	SSR53	221348	221357	di-	(AG)5
MC1	SSR54	224542	224553	tetra-	(GCCG)3
MC1	SSR55	236633	236650	hexa-	(TTTACA)3
MC1	SSR56	236833	236842	mono-	(T)10
MC1	SSR57	241691	241700	di-	(CT)5
MC1	SSR58*	242042	242051	mono-	(T)10
MC1	SSR59*	244673	244687	penta-	(CTAGT)3
MC1	SSR60	247011	247022	tri-	(TAA)4
MC1	SSR61	247555	247566	tetra-	(TCTT)3
MC1	SSR62	249767	249778	tetra-	(AGAT)3
MC1	SSR63	252341	252350	di-	(AT)5
MC1	SSR64	255694	255705	tetra-	(AGAA)3
MC1	SSR65	261609	261620	tetra-	(TTCT)3
MC1	SSR66	263325	263336	tetra-	(AAGC)3
MC1	SSR67	265540	265551	tetra-	(AGAA)3
MC2	SSR68	830	839	di-	(TC)5
MC2	SSR69	5103	5112	di-	(AT)5
MC2	SSR70	9766	9775	di-	(TC)5
MC2	SSR71	18627	18641	penta-	(TATAA)3
MC2	SSR72	20392	20403	tetra-	(TCTA)3
MC2	SSR73*	25215	25226	tetra-	(AAGA)3
MC2	SSR74	25703	25714	tetra-	(AATA)3
MC2	SSR75	26570	26581	tetra-	(AGTG)3
MC2	SSR76	37406	37415	mono-	(A)10
MC2	SSR77	37494	37503	mono-	(A)10
MC2	SSR78	38492	38501	mono-	(A)10

Table S6 Long tandem repeats in the mitogenome of *S. officinalis*. The tandem repeats units in the exonic regions of genes were highlighted with the asterisks.

Chromosomes	ID	Indices	Period size(bp)	Copy Number	Consensus Size (bp)	Percent Matches	Percent Indels	Score	Bases number				Entropy
									A	G	C	T	(0–2)
MC1	TR1	34927-35003	18	4.3	18	90	6	102	33	6	25	33	1.82
MC1	TR2	34928-35003	36	2.1	36	90	0	116	34	6	25	34	1.82
MC1	TR3	57560-57594	17	2	18	88	5	54	25	20	31	22	1.98
MC1	TR4	66206-66278	33	2.3	31	84	11	87	28	15	8	47	1.73
MC1	TR5	167466-167500	17	2	18	94	5	63	48	22	0	28	1.51
MC1	TR6	167493-167538	23	2	23	86	0	65	60	6	6	26	1.46
MC1	TR7	190941-190979	20	2	20	84	0	51	69	7	17	5	1.32
MC2	TR8*	25104-25176	33	2.3	31	84	11	87	47	8	15	28	1.73
MC2	TR9	25792-25817	12	2.2	12	100	0	52	19	30	7	42	1.79

Table S7 List of mitochondrial plastid DNA (MTPT) in the *S. officinalis* mitogenome. MC1/2: mitogenomic chromosomes 1/2.

MTPT ID	Chromosome	Identity (%)	Alignment Length (bp)	Number of Mismatches	Number of Gap openings	Positions on the Chloroplast Genome		Positions on the Mitochondrial Genome		Genes Located in the MTPT Fragments	E value	Score
						Start	End	Start	End			
mtpt01	MC1	100	4261	0	0	141708	145968	37176	32916	*ycf2* fragment, *ycf15*	0	7869
mtpt02	MC1	100	1447	0	0	149035	150481	55242	56688	*rpl23*, *trnI*-CAU	0	2673
mtpt03	MC1	98.582	141	2	0	36259	36399	62423	62283		1.93E-65	250
mtpt04	MC1	98.485	66	1	0	54329	54394	64471	64536		2.04E-25	1.17E+02
mtpt05	MC1	94.118	85	4	1	126934	127017	105341	105425	*trn*N-GUU	9.42E-29	1.28E+02
mtpt06	MC1	92	50	3	1	134881	134930	130404	130452		5.79E-11	6.94E+01
mtpt07	MC1	93.75	80	5	0	51266	51345	136496	136575	*trn*M-CAU	1.58E-26	1.21E+02
mtpt08	MC1	100	386	0	0	43011	43396	159908	159523		0	7.13E+02
mtpt09	MC1	75.599	459	100	11	10034	10486	160245	160697		1.95E-55	2.17E+02
mtpt10	MC1	79.705	882	139	31	98634	99497	166520	165661		4.62E-171	6.01E+02
mtpt11	MC1	99.972	3598	1	0	135731	139328	167076	170673	*rps*7	0	6.64E+03
mtpt12	MC1	100	41	0	0	115938	115978	168353	168393		3.46E-13	7.68E+01
mtpt13	MC1	100	43	0	0	55410	55452	195019	195061		2.67E-14	8.05E+01
mtpt14	MC1	97.531	81	2	0	8	88	200038	200118	*trn*H-GUG	4.35E-32	1.39E+02
mtpt15	MC1	89.313	131	13	1	29441	29570	218815	218685	*trn*D-GUC	2.58E-39	1.63E+02
mtpt16	MC1	88.95	181	16	2	44071	44251	219338	219514		1.51E-56	2.20E+02
mtpt17	MC1	78.889	90	16	3	7827	7915	219501	219414	*trn*S-GGA	1.25E-07	5.84E+01

mtpt18	MC1	87.64	178	11	4	44790	44967	219520	219686		2.55E-49	1.96E+02
mtpt19	MC1	97.561	164	4	0	33546	33709	245207	245044		6.83E-75	2.81E+02
mtpt20	MC1	100	1698	0	0	31491	33188	246897	245200	*psbD*	0	3.14E+03
mtpt21	MC1	92.661	109	3	2	71222	71329	252451	252347		5.59E-36	1.52E+02
mtpt22	MC2	100	196	0	0	65279	65474	11286	11481	*trn*P-UGG	2.4E-99	363
mtpt23	MC2	100	128	0	0	65075	65202	11110	11237	*trn*W-CCA	1.5E-61	237

Table S8 Summary of RNA editing sites detected in the PCGs of the *S. officinalis* mitogenome. “Nt Pos” and “AA Pos” mean the positions of the RNA editing sites in the nucleotide and amino acid sequences of the PCGs. P: the sequence was successfully validated by both PCR amplification and Sanger sequencing experiments. N: the sequence was successfully amplified by PCR but the Sanger sequencing results differed from those expected. NA: the sequence was not validated by PCR and Sanger sequencing experiments. The 193 sites of *S. officinalis* mitogenome homologous to those of the *S. miltiorrhiza* mitogenome are underlined.

Gene	Nt Pos	AA Pos	Reference Nt	Nt coverage	Nt Base Count [A,C,G,T]	Nt allsubs	Nt Frequency	Effect	PCR Success	Codon Position
atp4	59	20	C	4474	[0, 31, 0, 4443]	CT	0.99	TCT (S) => TTT (F)	P	2
atp4	71	24	C	5250	[1, 27, 0, 5222]	CT CA	0.99	TCA (S) => TTA (L)	P	2
atp4	89	30	C	4834	[0, 107, 0, 4727]	CT	0.98	TCA (S) => TTA (L)	P	2
atp4	118	40	C	5023	[0, 18, 0, 5005]	CT	1	CGT (R) => TGT (C)	P	1
atp4	215	72	C	6007	[1, 39, 0, 5967]	CT CA	0.99	TCG (S) => TTG (L)	P	2
atp4	227	76	C	6076	[0, 84, 1, 5991]	CT CG	0.99	CCC (P) => CTC (L)	P	2
atp4	248	83	C	5278	[0, 27, 0, 5251]	CT	0.99	CCT (P) => CTT (L)	P	2
atp4	251	84	C	5577	[0, 37, 1, 5539]	CT CG	0.99	CCG (P) => CTG (L)	P	2
atp4	395	132	C	2771	[0, 9, 0, 2762]	CT	1	TCA (S) => TTA (L)	P	2
atp4	407	136	C	2590	[1, 5, 0, 2584]	CT CA	1	CCA (P) => CTA (L)	P	2
atp4	416	139	C	2323	[0, 23, 0, 2300]	CT	0.99	ACT (T) => ATT (I)	P	2
atp6	26	9	C	9490	[1, 84, 0, 9405]	CT CA	0.99	TCA (S) => TTA (L)	NA	2
atp6	77	26	C	10579	[0, 30, 1, 10548]	CT CG	1	CCG (P) => CTG (L)	NA	2
atp6	146	49	C	8603	[0, 53, 0, 8550]	CT	0.99	TCG (S) => TTG (L)	NA	2
atp6	164	55	C	8739	[0, 78, 1, 8660]	CT CG	0.99	TCG (S) => TTG (L)	NA	2
atp6	172	58	C	7290	[0, 14, 0, 7276]	CT	1	CGT (R) => TGT (C)	NA	1
atp6	311	104	C	10524	[0, 41, 0, 10483]	CT	1	TCA (S) => TTA (L)	NA	2

atp6	370	124	C	9843	[0, 32, 0, 9811]	CT	1	CCT (P) => TCT (S)	NA	1
atp6	373	125	C	9878	[0, 48, 0, 9830]	CT	1	CAT (H) => TAT (Y)	NA	1
atp8	30	16	C	3042	[0, 1434, 0, 1608]	CT	0.53	TCA (S) => TTA (L)	NA	2
atp8	58	20	C	2774	[0, 131, 1, 2642]	CT CG	0.95	CTC (L) => TTC (F)	P	1
atp9	212	71	C	1446	[0, 5, 0, 1441]	CT	1	TCA (S) => TTA (L)	NA	2
ccmB	28	10	C	57	[0, 29, 0, 28]	CT	0.49	CAT (H) => TAT (Y)	NA	1
ccmB	80	27	C	30	[0, 20, 0, 10]	CT	0.33	TCG (S) => TTG (L)	NA	2
ccmB	87	87	C	39	[0, 25, 0, 14]	CT	0.36	ATC (I) => ATT (I)	NA	3
ccmB	128	43	C	34	[4, 0, 30, 0]	CT	0.12	TCA (S) => TTA (L)	NA	2
ccmB	286	96	C	36	[0, 25, 0, 11]	CT	0.31	CGG (R) => TGG (W)	NA	1
ccmB	304	102	C	38	[0, 26, 0, 12]	CT	0.32	CGT (R) => TGT (C)	NA	1
ccmB	313	105	C	40	[0, 25, 0, 15]	CT	0.38	CGT (R) => TGT (C)	NA	1
ccmB	338	113	C	39	[0, 26, 0, 13]	CT	0.33	CCG (P) => CTG (L)	NA	2
ccmB	367	123	C	39	[0, 34, 0, 5]	CT	0.13	CGG (R) => TGG (W)	NA	1
ccmB	380	127	C	37	[0, 29, 0, 8]	CT	0.22	CCA (P) => CTA (L)	NA	2
ccmB	424	142	C	25	[0, 22, 0, 3]	CT	0.12	CGT (R) => TGT (C)	NA	1
ccmB	428	143	C	27	[0, 20, 0, 7]	CT	0.26	TCG (S) => TTG (L)	NA	2
ccmB	551	184	C	7	[1, 0, 6, 0]	CT	0.14	TCA (S) => TTA (L)	NA	2
ccmC	38	13	C	487	[0, 40, 0, 447]	CT	0.92	TCA (S) => TTA (L)	NA	2
ccmC	76	26	C	398	[0, 12, 0, 386]	CT	0.97	CGG (R) => TGG (W)	NA	1
ccmC	103	35	C	347	[0, 5, 0, 342]	CT	0.99	CAT (H) => TAT (Y)	NA	1
ccmC	115	39	C	305	[0, 16, 0, 289]	CT	0.95	CGG (R) => TGG (W)	NA	1
ccmC	133	45	C	310	[0, 23, 0, 287]	CT	0.93	CTT (L) => TTT (F)	NA	1
ccmC	161	54	C	235	[0, 6, 0, 229]	CT	0.97	CCC (P) => CTC (L)	NA	2
ccmC	179	60	C	194	[0, 2, 0, 192]	CT	0.99	GCG (A) => GTG (V)	NA	2
ccmC	184	62	C	195	[0, 4, 0, 191]	CT	0.98	CGG (R) => TGG (W)	NA	1

ccmC	253	85	C	139	[0, 124, 0, 15]	CT	0.11	CTT (L) => TTT (F)	NA	1
ccmC	281	94	C	65	[0, 8, 0, 57]	CT	0.88	ACA (T) => ATA (I)	NA	2
ccmC	299	100	C	41	[0, 21, 0, 20]	CT	0.49	TCT (S) => TTT (F)	NA	2
ccmC	331	111	C	27	[0, 11, 0, 16]	CT	0.59	CGG (R) => TGG (W)	NA	1
ccmC	395	132	C	18	[0, 14, 0, 4]	CT	0.22	TCG (S) => TTG (L)	NA	2
ccmC	399	133	C	18	[0, 10, 0, 8]	CT	0.44	TTC (F) => TTT (F)	NA	3
ccmC	400	134	C	18	[0, 14, 0, 4]	CT	0.22	CTT (L) => TTT (F)	NA	1
ccmC	436	146	C	16	[0, 13, 0, 3]	CT	0.19	CCT (P) => TCT (S)	NA	1
ccmC	446	149	C	15	[0, 13, 0, 2]	CT	0.13	CCG (P) => CTG (L)	NA	2
ccmC	458	153	C	14	[0, 12, 0, 2]	CT	0.14	TCA (S) => TTA (L)	NA	2
ccmC	548	183	C	4	[0, 3, 0, 1]	CT	0.25	TCT (S) => TTT (F)	NA	2
ccmC	568	190	C	4	[0, 3, 0, 1]	CT	0.25	CCT (P) => TCT (S)	NA	1
ccmC	575	192	C	4	[0, 3, 0, 1]	CT	0.25	CCC (P) => CTC (L)	NA	2
ccmC	605	202	C	4	[0, 3, 0, 1]	CT	0.25	TCC (S) => TTC (F)	NA	2
ccmC	608	203	C	4	[0, 0, 0, 4]	CT	1	CCC (P) => CTC (L)	NA	2
ccmC	614	205	C	4	[0, 1, 0, 3]	CT	0.75	TCA (S) => TTA (L)	NA	2
ccmC	650	217	C	4	[0, 0, 0, 4]	CT	1	CCT (P) => CTT (L)	NA	2
ccmC	673	225	C	4	[0, 1, 0, 3]	CT	0.75	CCT (P) => TCT (S)	NA	1
ccmFc	16	6	C	319	[0, 270, 0, 49]	CT	0.15	CAC (H) => TAC (Y)	NA	1
ccmFc	38	13	C	312	[0, 25, 0, 287]	CT	0.92	TCC (S) => TTC (F)	NA	2
ccmFc	50	17	C	247	[0, 15, 0, 232]	CT	0.94	CCT (P) => CTT (L)	NA	2
ccmFc	52	18	C	263	[0, 22, 0, 241]	CT	0.92	CGT (R) => TGT (C)	NA	1
ccmFc	103	35	C	288	[0, 5, 0, 283]	CT	0.98	CCC (P) => TCC (S)	NA	1
ccmFc	122	41	C	360	[0, 13, 0, 347]	CT	0.96	TCC (S) => TTC (F)	NA	2
ccmFc	146	49	C	363	[0, 2, 0, 361]	CT	0.99	CCT (P) => CTT (L)	NA	2
ccmFc	151	51	C	370	[0, 5, 0, 365]	CT	0.99	CCT (P) => TCT (S)	NA	1

ccmFc	155	52	C	370	[0, 15, 0, 355]	CT	0.96	TCA (S) => TTA (L)	NA	2
ccmFc	310	104	C	479	[0, 9, 0, 470]	CT	0.98	CGT (R) => TGT (C)	NA	1
ccmFc	321	107	C	503	[0, 422, 0, 81]	CT	0.16	CCC (P) => CCT (P)	NA	3
ccmFc	334	112	C	455	[0, 175, 0, 280]	CT	0.62	CTT (L) => TTT (F)	NA	1
ccmFc	378	126	C	397	[0, 131, 0, 266]	CT	0.67	TTC (F) => TTT (F)	NA	3
ccmFc	390	130	C	431	[0, 389, 0, 42]	CT	0.1	CTC (L) => CTT (L)	NA	3
ccmFc	406	136	C	437	[0, 15, 0, 422]	CT	0.97	CGT (R) => TGT (C)	NA	1
ccmFc	1228	410	C	64	[0, 2, 0, 62]	CT	0.97	CGG (R) => TGG (W)	NA	1
ccmFc	1233	411	C	61	[0, 55, 0, 6]	CT	0.1	ATC (I) => ATT (I)	NA	3
ccmFn	38	13	C	1934	[0, 8, 0, 1926]	CT	1	CCG (P) => CTG (L)	NA	2
ccmFn	98	33	C	1456	[0, 157, 0, 1299]	CT	0.89	CCT (P) => CTT (L)	NA	2
ccmFn	137	46	C	727	[0, 51, 0, 676]	CT	0.93	TCG (S) => TTG (L)	NA	2
ccmFn	142	48	C	772	[0, 70, 0, 702]	CT	0.91	CGT (R) => TGT (C)	NA	1
ccmFn	151	51	C	763	[0, 100, 0, 663]	CT	0.87	CCT (P) => TCT (S)	NA	1
ccmFn	165	55	C	750	[0, 609, 0, 141]	CT	0.19	TCC (S) => TCT (S)	NA	3
ccmFn	248	83	C	1184	[0, 60, 0, 1124]	CT	0.95	TCA (S) => TTA (L)	NA	2
ccmFn	256	86	C	1305	[0, 52, 0, 1253]	CT	0.96	CGG (R) => TGG (W)	NA	1
ccmFn	283	95	C	1315	[0, 42, 0, 1273]	CT	0.97	CTT (L) => TTT (F)	NA	1
ccmFn	371	124	C	1198	[0, 34, 0, 1164]	CT	0.97	TCG (S) => TTG (L)	NA	2
ccmFn	378	126	C	1173	[1, 179, 0, 993]	CT CA	0.85	TTC (F) => TTT (F)	NA	3
ccmFn	713	238	C	631	[1, 27, 0, 603]	CT CA	0.96	CCT (P) => CTT (L)	NA	2
ccmFn	722	241	C	612	[0, 60, 0, 552]	CT	0.9	TCA (S) => TTA (L)	NA	2
ccmFn	732	244	C	640	[0, 481, 0, 159]	CT	0.25	TCC (S) => TCT (S)	NA	3
ccmFn	760	254	C	761	[1, 25, 0, 735]	CT CA	0.97	CGT (R) => TGT (C)	NA	1
ccmFn	782	261	C	866	[0, 76, 0, 790]	CT	0.91	TCA (S) => TTA (L)	NA	2
ccmFn	794	265	C	983	[0, 45, 0, 938]	CT	0.95	CCA (P) => CTA (L)	NA	2

ccmFn	809	270	C	985	[0, 48, 0, 937]	CT	0.95	TCA (S) => TTA (L)	NA	2
ccmFn	867	289	C	1158	[0, 420, 0, 738]	CT	0.64	GGC (G) => GGT (G)	NA	3
ccmFn	958	320	C	1275	[0, 37, 0, 1238]	CT	0.97	CGC (R) => TGC (C)	NA	1
ccmFn	1276	426	C	886	[0, 62, 0, 824]	CT	0.93	CGG (R) => TGG (W)	NA	1
ccmFn	1304	435	C	892	[0, 56, 0, 836]	CT	0.94	CCA (P) => CTA (L)	NA	2
ccmFn	1321	441	C	970	[0, 60, 0, 910]	CT	0.94	CAT (H) => TAT (Y)	NA	1
ccmFn	1336	446	C	957	[0, 57, 0, 900]	CT	0.94	CGG (R) => TGG (W)	NA	1
ccmFn	1354	452	C	1161	[0, 71, 0, 1090]	CT	0.94	CGG (R) => TGG (W)	NA	1
ccmFn	1387	463	C	1292	[0, 64, 0, 1228]	CT	0.95	CGG (R) => TGG (W)	NA	1
ccmFn	1448	483	C	1704	[0, 19, 0, 1685]	CT	0.99	TCC (S) => TTC (F)	NA	2
ccmFn	1472	491	C	1660	[0, 27, 0, 1633]	CT	0.98	CCA (P) => CTA (L)	NA	2
ccmFn	1484	495	C	1597	[0, 30, 0, 1567]	CT	0.98	TCA (S) => TTA (L)	NA	2
ccmFn	1519	507	C	1212	[0, 28, 0, 1184]	CT	0.98	CCC (P) => TCC (S)	NA	1
cob	114	38	C	4575	[1, 3354, 0, 1220]	CT CA	0.27	TTC (F) => TTT (F)	NA	3
cob	180	60	C	4081	[0, 3256, 0, 825]	CT	0.2	TAC (Y) => TAT (Y)	NA	3
cob	298	100	C	3977	[0, 26, 0, 3951]	CT	0.99	CAC (H) => TAC (Y)	NA	1
cob	325	109	C	3603	[0, 35, 0, 3568]	CT	0.99	CAT (H) => TAT (Y)	NA	1
cob	358	120	C	3628	[0, 49, 1, 3578]	CT CG	0.99	CGG (R) => TGG (W)	NA	1
cob	568	190	C	3727	[0, 32, 0, 3695]	CT	0.99	CAT (H) => TAT (Y)	NA	1
cob	853	285	C	5247	[0, 36, 0, 5211]	CT	0.99	CAT (H) => TAT (Y)	NA	1
cob	908	303	C	4047	[0, 36, 0, 4011]	CT	0.99	TCA (S) => TTA (L)	NA	2
cob	982	328	C	2417	[0, 163, 0, 2254]	CT	0.93	CAC (H) => TAC (Y)	NA	1
cob	1015	339	C	1949	[0, 39, 0, 1910]	CT	0.98	CGC (R) => TGC (C)	NA	1
cob	1084	362	C	1072	[0, 19, 0, 1053]	CT	0.98	CCT (P) =>TCT (S)	NA	1
cob	1160	387	C	393	[0, 264, 0, 129]	CT	0.33	ACG (P) =>ATG (M)	NA	2
cox1	242	81	C	6994	[0, 27, 0, 6967]	CT	1	TCT (S) => TTT (F)	P	2

cox1	254	85	C	8482	[0, 31, 0, 8451]	CT	1	TCT (S) => TTT (F)	P	2
cox1	452	151	C	3351	[0, 11, 0, 3340]	CT	1	TCT (S) => TTT (F)	P	2
cox1	515	172	C	1820	[0, 5, 0, 1815]	CT	1	TCC (S) => TTC (F)	P	2
cox1	551	184	C	1139	[0, 5, 0, 1134]	CT	1	TCA (S) => TTA (L)	P	2
cox1	590	197	C	578	[0, 3, 0, 575]	CT	0.99	CCA (P) => CTA (L)	P	2
cox1	715	239	C	48	[0, 0, 0, 48]	CT	1	CGG (R) => TGG (W)	P	1
cox1	761	254	C	2262	[0, 48, 0, 2214]	CT	0.98	TCC (S) => TTC (F)	N	2
cox1	1078	360	C	8275	[0, 6261, 0, 2014]	CT	0.24	CTG (L) => TTG (L)	N	1
cox1	1186	396	C	9741	[0, 5, 0, 9736]	CT	1	CAC (H) => TAC (Y)	P	1
cox1	1405	469	C	4684	[0, 30, 0, 4654]	CT	0.99	CGT (R) => TGT (C)	P	1
cox1	1413	471	C	4508	[0, 2153, 0, 2355]	CT	0.52	TTC (F) => TTT (F)	N	3
cox1	1433	478	C	3399	[0, 14, 0, 3385]	CT	1	TCA (S) => TTA (L)	P	2
cox1	1489	497	C	1439	[0, 6, 0, 1433]	CT	1	CCA (P) => TCA (S)	N	1
cox1	1499	500	C	1238	[0, 11, 0, 1227]	CT	0.99	CCA (P) => CTA (L)	P	2
cox2	47	16	C	5613	[0, 63, 0, 5550]	CT	0.99	TCT (S) => TTT (F)	NA	2
cox2	419	140	C	3296	[0, 72, 0, 3224]	CT	0.98	ACG (P) =>ATG (M)	NA	2
cox2	437	146	C	2919	[0, 118, 0, 2801]	CT	0.96	CCA (P) => CTA (L)	NA	2
cox2	452	151	C	2567	[0, 131, 0, 2436]	CT	0.95	TCA (S) => TTA (L)	NA	2
cox2	520	174	C	872	[0, 31, 0, 841]	CT	0.96	CCT (P) => TCT (S)	NA	1
cox2	533	178	C	664	[0, 34, 0, 630]	CT	0.95	CCT (P) => CTT (L)	NA	2
cox2	595	199	C	228	[58, 170, 0, 0]	CA	0.25	CAG (Q) => AAG (K)	NA	1
cox2	608	203	C	161	[0, 2, 0, 159]	CT	0.99	TCG (S) => TTG (L)	NA	2
cox2	697	233	C	44	[0, 3, 0, 41]	CT	0.93	CCT (P) => TCT (S)	NA	1
cox2	718	240	C	53	[0, 5, 0, 48]	CT	0.91	CGG (R) => TGG (W)	NA	1
cox3	174	58	C	4400	[0, 3807, 0, 593]	CT	0.13	TTC (F) => TTT (F)	N	3
cox3	245	82	C	3733	[0, 32, 0, 3701]	CT	0.99	CCT (P) => CTT (L)	P	2

cox3	304	102	C	2928	[0, 32, 3, 2893]	CT CG	0.99	CGG (R) => TGG (W)	P	1
cox3	311	104	C	2931	[0, 120, 2, 2809]	CT CG	0.96	TCT (S) => TTT (F)	P	2
cox3	314	105	C	2893	[0, 56, 0, 2837]	CT	0.98	TCT (S) => TTT (F)	P	2
cox3	419	140	C	5644	[0, 50, 0, 5594]	CT	0.99	CCC (P) => CTC (L)	P	2
cox3	422	141	C	5327	[0, 93, 0, 5234]	CT	0.98	CCT (P) => CTT (L)	P	2
cox3	566	189	C	8247	[1, 110, 0, 8136]	CT CA	0.99	TCC (S) => TTC (F)	P	2
cox3	567	189	C	8377	[0, 4704, 0, 3673]	CT	0.44	TCC (S) => TTT (F)	N	3
cox3	754	252	C	697	[0, 7, 0, 690]	CT	0.99	CGG (R) => TGG (W)	P	1
cox3	764	255	C	627	[0, 16, 0, 611]	CT	0.97	CCA (P) => CTA (L)	P	2
matR	32	11	C	1325	[0, 31, 0, 1294]	CT	0.98	TCC (S) => TTC (F)	NA	2
matR	43	15	C	1270	[0, 54, 0, 1216]	CT	0.96	CCC (P) => TCC (S)	NA	1
matR	237	79	C	317	[0, 276, 0, 41]	CT	0.13	TCC (S) => TCT (S)	NA	3
matR	254	85	C	327	[0, 251, 0, 76]	CT	0.23	TCC (S) => TTC (F)	NA	2
matR	258	86	C	341	[0, 261, 0, 80]	CT	0.23	GCC (A) => GCT (A)	NA	3
matR	326	109	C	375	[0, 15, 0, 360]	CT	0.96	CCA (P) => CTA (L)	NA	2
matR	413	138	C	362	[0, 18, 0, 344]	CT	0.95	TCG (S) => TTG (L)	NA	2
matR	1400	467	C	939	[0, 799, 0, 140]	CT	0.15	TCG (S) => TTG (L)	NA	2
matR	1522	508	C	996	[0, 799, 0, 197]	CT	0.2	CCC (P) => TCT (S)	NA	1
matR	1524	508	C	1009	[0, 778, 0, 231]	CT	0.23	CCC (P) => TCT (S)	NA	3
matR	1658	553	C	1363	[0, 37, 0, 1326]	CT	0.97	TCC (S) => TTC (F)	NA	2
matR	1679	560	C	1133	[0, 59, 0, 1074]	CT	0.95	CCT (P) => CTT (L)	NA	2
matR	1699	567	C	1004	[0, 63, 0, 941]	CT	0.94	CGC (R) => TGC (C)	NA	1
matR	1713	571	C	1011	[0, 258, 0, 753]	CT	0.74	TAC (Y) => TAT (Y)	NA	3
matR	1735	579	C	937	[0, 49, 0, 888]	CT	0.95	CAC (H) => TAC (Y)	NA	1
matR	1766	589	C	793	[0, 29, 0, 764]	CT	0.96	CCG (P) => CTG (L)	NA	2
matR	1805	602	C	698	[0, 29, 0, 669]	CT	0.96	CCA (P) => CTA (L)	NA	2

matR	1823	608	C	586	[0, 30, 0, 556]	CT	0.95	TCA (S) => TTA (L)	NA	2
mttB	16	6	C	310	[0, 207, 0, 103]	CT	0.33	CAT (H) => TAT (Y)	P	1
mttB	26	9	C	339	[0, 73, 0, 266]	CT	0.78	CCG (P) => CTG (L)	P	2
mttB	64	22	C	319	[0, 28, 0, 291]	CT	0.91	CGG (R) => TGG (W)	P	1
mttB	100	34	C	176	[0, 75, 0, 101]	CT	0.57	CGT (R) => TGT (C)	P	1
mttB	112	38	C	154	[0, 46, 0, 108]	CT	0.7	CCG (P) => TCG (S)	P	1
mttB	128	43	C	115	[0, 72, 0, 43]	CT	0.37	TCT (S) => TTT (F)	P	2
mttB	131	44	C	108	[0, 50, 0, 58]	CT	0.54	CCA (P) => CTA (L)	P	2
mttB	178	60	C	158	[0, 20, 0, 138]	CT	0.87	CGT (R) => TGT (C)	P	1
mttB	188	63	C	160	[0, 26, 0, 134]	CT	0.84	TCA (S) => TTA (L)	P	2
mttB	201	67	C	173	[0, 150, 0, 23]	CT	0.13	TTC (F) => TTT (F)	N	3
mttB	202	68	C	172	[0, 51, 0, 121]	CT	0.7	CCG (P) => TCG (S)	P	1
mttB	236	79	C	184	[0, 93, 0, 91]	CT	0.49	TCT (S) => TTT (F)	P	2
mttB	262	88	C	192	[0, 16, 0, 176]	CT	0.92	CAT (H) => TAT (Y)	P	1
mttB	328	110	C	112	[0, 49, 0, 63]	CT	0.56	CTC (L) => TTC (F)	P	1
mttB	331	111	C	112	[0, 23, 0, 89]	CT	0.79	CAT (H) => TAT (Y)	P	1
mttB	344	115	C	98	[0, 58, 0, 40]	CT	0.41	TCT (S) => TTT (F)	P	2
mttB	346	116	C	102	[0, 34, 0, 68]	CT	0.67	CGC (R) => TGC (C)	P	1
mttB	373	125	C	80	[0, 56, 0, 24]	CT	0.3	CTT (L) => TTT (F)	P	1
mttB	376	126	C	74	[0, 21, 0, 53]	CT	0.72	CCC (P) => TCC (S)	P	1
mttB	379	127	C	84	[0, 56, 0, 28]	CT	0.33	CGG (R) => TGG (W)	P	1
mttB	407	136	C	347	[0, 10, 0, 337]	CT	0.97	CCA (P) => CTA (L)	P	2
mttB	472	158	C	573	[0, 16, 0, 557]	CT	0.97	CAT (H) => TAT (Y)	P	1
mttB	497	166	C	617	[0, 26, 0, 591]	CT	0.96	TCG (S) => TTG (L)	P	2
mttB	505	169	C	635	[0, 26, 0, 609]	CT	0.96	CCA (P) => TCA (S)	P	1
mttB	541	181	C	517	[0, 46, 0, 471]	CT	0.91	CGT (R) => TGT (C)	P	1

mttB	554	185	C	365	[0, 85, 0, 280]	CT	0.77	CCA (P) => CTA (L)	P	2
mttB	578	193	C	245	[0, 37, 0, 208]	CT	0.85	TCC (S) => TTC (F)	P	2
mttB	610	204	C	152	[0, 6, 0, 146]	CT	0.96	CCG (P) => TCG (S)	P	1
mttB	616	206	C	149	[0, 17, 0, 132]	CT	0.89	CTC (L) => TTC (F)	P	1
mttB	667	223	C	73	[0, 5, 0, 68]	CT	0.93	CGT (R) => TGT (C)	P	1
mttB	672	224	C	128	[0, 19, 0, 109]	CT	0.85	TTC (F) => TTT (F)	N	3
mttB	713	238	C	97	[0, 4, 0, 93]	CT	0.96	TCG (S) => TTG (L)	P	2
nad1	215	72	C	642	[0, 17, 0, 625]	CT	0.97	TCC (S) => TTC (F)	NA	2
nad1	265	89	C	324	[0, 13, 0, 311]	CT	0.96	CGG (R) => TGG (W)	NA	1
nad1	307	103	C	114	[0, 3, 0, 111]	CT	0.97	CCG (P) => TTG (S)	NA	1
nad1	308	103	C	108	[0, 1, 0, 107]	CT	0.99	CCG (P) => TTG (L)	NA	2
nad1	376	126	C	40	[0, 0, 0, 40]	CT	1	CGG (R) => TGG (W)	NA	1
nad1	401	134	C	43	[0, 11, 0, 32]	CT	0.74	TCT (S) => TTT (F)	NA	2
nad1	436	146	C	28	[0, 6, 0, 22]	CT	0.79	CCT (P) => TCT (S)	NA	1
nad1	490	164	C	48	[0, 22, 0, 26]	CT	0.54	CCC (P) => TCT (S)	NA	1
nad1	492	164	C	46	[0, 36, 0, 10]	CT	0.22	CCC (P) => TCT (S)	NA	3
nad1	493	165	C	43	[0, 11, 0, 32]	CT	0.74	CGT (R) => TGT (C)	NA	1
nad1	500	167	C	47	[0, 11, 0, 36]	CT	0.77	TCG (S) => TTG (L)	NA	2
nad1	536	179	C	47	[0, 6, 0, 41]	CT	0.87	TCC (S) => TTC (F)	NA	2
nad1	635	212	C	13	[0, 3, 0, 10]	CT	0.77	TCA (S) => TTA (L)	NA	2
nad1	725	242	C	258	[0, 30, 0, 228]	CT	0.88	CCA (P) => CTA (L)	NA	2
nad1	734	245	C	279	[0, 73, 0, 206]	CT	0.74	TCG (S) => TTG (L)	NA	2
nad1	740	247	C	271	[0, 67, 0, 204]	CT	0.75	TCT (S) => TTT (F)	NA	2
nad1	743	248	C	275	[0, 66, 0, 209]	CT	0.76	CCA (P) => CTA (L)	NA	2
nad1	755	252	C	587	[0, 160, 0, 427]	CT	0.73	CCG (P) => CTG (L)	NA	2
nad1	779	260	C	817	[0, 68, 0, 749]	CT	0.92	TCC (S) => TTC (F)	NA	2

nad1	792	264	C	956	[0, 455, 0, 501]	CT	0.52	CCC (P) => CCT (P)	NA	3
nad1	823	275	C	1072	[0, 18, 0, 1054]	CT	0.98	CTC (L) => TTC (F)	NA	1
nad1	898	300	C	1042	[0, 17, 0, 1025]	CT	0.98	CGG (R) => TGG (W)	NA	1
nad1	909	303	C	832	[0, 395, 0, 437]	CT	0.53	TTC (F) => TTT (F)	NA	3
nad1	928	310	C	613	[0, 12, 0, 601]	CT	0.98	CGG (R) => TGG (W)	NA	1
nad2	26	9	C	147	[0, 6, 0, 141]	CT	0.96	TCC (S) => TTC (F)	NA	2
nad2	223	75	C	550	[0, 14, 0, 536]	CT	0.97	CTT (L) => TTT (F)	NA	1
nad2	252	84	C	541	[0, 159, 0, 382]	CT	0.71	TTC (F) => TTT (F)	NA	3
nad2	303	101	C	301	[0, 117, 0, 184]	CT	0.61	TTC (F) => TTT (F)	NA	3
nad2	308	103	C	266	[0, 24, 0, 242]	CT	0.91	TCT (S) => TTT (F)	NA	2
nad2	311	104	C	265	[0, 38, 0, 227]	CT	0.86	TCC (S) => TTC (F)	NA	2
nad2	356	119	C	136	[0, 15, 0, 121]	CT	0.89	CCA (P) => CTA (L)	NA	2
nad2	361	121	C	130	[0, 15, 0, 115]	CT	0.88	CCT (P) => TCT (S)	NA	1
nad2	367	123	C	125	[0, 19, 0, 106]	CT	0.85	CGC (R) => TGC (C)	NA	1
nad2	401	134	C	105	[0, 19, 0, 86]	CT	0.82	TCA (S) => TTA (L)	NA	2
nad2	428	143	C	92	[0, 16, 0, 76]	CT	0.83	CCT (P) => CTT (L)	NA	2
nad2	497	166	C	39	[0, 6, 0, 33]	CT	0.85	TCG (S) => TTG (L)	NA	2
nad2	788	263	C	1447	[0, 26, 0, 1421]	CT	0.98	TCT (S) => TTT (F)	NA	2
nad2	800	267	C	1565	[0, 29, 0, 1536]	CT	0.98	TCA (S) => TTA (L)	NA	2
nad2	809	270	C	1591	[0, 37, 0, 1554]	CT	0.98	TCT (S) => TTT (F)	NA	2
nad2	928	310	C	1218	[0, 33, 0, 1185]	CT	0.97	CAT (H) => TAT (Y)	NA	1
nad2	958	320	C	1031	[0, 19, 0, 1012]	CT	0.98	CGT (R) => TGT (C)	NA	1
nad2	1028	343	C	498	[0, 9, 0, 489]	CT	0.98	TCA (S) => TTA (L)	NA	2
nad2	1058	353	C	356	[0, 17, 0, 339]	CT	0.95	TCA (S) => TTA (L)	NA	2
nad2	1298	433	C	199	[0, 4, 0, 195]	CT	0.98	GCG (A) => GTG (V)	NA	2
nad2	1400	467	C	320	[0, 7, 0, 313]	CT	0.98	TCA (S) => TTA (L)	NA	2

nad2	1408	470	C	315	[0, 10, 0, 305]	CT	0.97	CCA (P) => TTA (S)	NA	1
nad2	1409	470	C	315	[0, 4, 0, 311]	CT	0.99	CCA (P) => TTA (L)	NA	2
nad2	1416	472	C	315	[0, 21, 0, 294]	CT	0.93	CCC (P) => CCT (P)	NA	3
nad2	1457	486	C	68	[0, 0, 0, 68]	CT	1	TCA (S) => TTA (L)	NA	2
nad3	44	15	C	333	[0, 19, 0, 314]	CT	0.94	TCG (S) => TTG (L)	P	2
nad3	62	21	C	290	[0, 20, 0, 270]	CT	0.93	CCA (P) => CTA (L)	P	2
nad3	79	27	C	220	[0, 49, 0, 171]	CT	0.78	CCA (P) => TTA (S)	P	1
nad3	80	27	C	219	[0, 15, 0, 204]	CT	0.93	CCA (P) => TTA (L)	P	2
nad3	124	42	C	154	[0, 13, 0, 141]	CT	0.92	CAC (H) => TAC (Y)	P	1
nad3	146	49	C	111	[0, 12, 0, 99]	CT	0.89	TCC (S) => TTC (F)	P	2
nad3	208	70	C	22	[0, 5, 0, 17]	CT	0.77	CCT (P) => TTT (F)	P	1
nad3	209	70	C	26	[0, 6, 0, 20]	CT	0.77	CCT (P) => TTT (F)	P	2
nad3	215	72	C	25	[0, 7, 0, 18]	CT	0.72	CCG (P) => CTG (L)	P	2
nad3	230	77	C	18	[0, 5, 0, 13]	CT	0.72	TCC (S) => TTC (F)	P	2
nad3	247	83	C	4	[0, 3, 0, 1]	CT	0.25	CCT (P) => TCT (S)	P	1
nad3	251	84	C	4	[0, 3, 0, 1]	CT	0.25	CCC (P) => CTC (L)	P	2
nad3	266	89	C	10	[0, 3, 0, 7]	CT	0.7	CCC (P) => CTC (L)	P	2
nad3	275	92	C	12	[0, 3, 0, 9]	CT	0.75	TCT (S) => TTT (F)	P	2
nad3	317	106	C	10	[0, 1, 0, 9]	CT	0.9	TCT (S) => TTT (F)	P	2
nad3	344	115	C	7	[0, 0, 0, 7]	CT	1	TCG (S) => TTG (L)	P	2
nad3	349	117	C	9	[0, 0, 0, 9]	CT	1	CGG (R) => TGG (W)	P	1
nad4	65	22	C	1284	[0, 138, 0, 1146]	CT	0.89	ACT (T) => ATT (I)	NA	2
nad4	68	23	C	1305	[0, 8, 0, 1297]	CT	0.99	CCT (P) => CTT (L)	NA	2
nad4	75	25	C	1342	[0, 1031, 0, 311]	CT	0.23	TTC (F) => TTT (F)	NA	3
nad4	98	33	C	1094	[0, 10, 0, 1084]	CT	0.99	CCG (P) => CTG (L)	NA	2
nad4	149	50	C	659	[0, 4, 0, 655]	CT	0.99	CCT (P) => CTT (L)	NA	2

nad4	157	53	C	722	[0, 2, 0, 720]	CT	1	CGG (R) => TGG (W)	NA	1
nad4	188	63	C	516	[0, 37, 0, 479]	CT	0.93	TCT (S) => TTT (F)	NA	2
nad4	353	118	C	51	[0, 0, 0, 51]	CT	1	ACA (T) => ATA (I)	NA	2
nad4	359	120	C	47	[0, 4, 0, 43]	CT	0.91	TCT (S) => TTT (F)	NA	2
nad4	367	123	C	46	[0, 1, 0, 45]	CT	0.98	CGT (R) => TGT (C)	NA	1
nad4	407	136	C	16	[0, 1, 0, 15]	CT	0.94	CCT (P) => CTT (L)	NA	2
nad4	424	142	C	12	[0, 1, 0, 11]	CT	0.92	CTT (L) => TTT (F)	NA	1
nad4	427	143	C	9	[0, 6, 0, 3]	CT	0.33	CCC (P) => TTC (S)	NA	1
nad4	428	143	C	11	[0, 8, 0, 3]	CT	0.27	CCC (P) => TTC (F)	NA	2
nad4	440	147	C	12	[0, 0, 0, 12]	CT	1	CCA (P) => CTA (L)	NA	2
nad4	599	200	C	2433	[1, 4, 0, 2428]	CT CA	1	TCA (S) => TTA (L)	NA	2
nad4	637	213	C	1726	[0, 1435, 0, 291]	CT	0.17	CTA (L) => TTA (L)	NA	1
nad4	650	217	C	1683	[0, 10, 0, 1673]	CT	0.99	TCT (S) => TTT (F)	NA	2
nad4	758	253	C	385	[0, 3, 0, 382]	CT	0.99	CCT (P) => CTT (L)	NA	2
nad4	810	270	C	223	[0, 170, 0, 53]	CT	0.24	CCC (P) => CCT (P)	NA	3
nad4	847	283	C	118	[0, 91, 0, 27]	CT	0.23	CCA (P) => TTA (S)	NA	1
nad4	848	283	C	110	[0, 13, 0, 97]	CT	0.88	CCA (P) => TTA (L)	NA	2
nad4	878	293	C	79	[0, 4, 0, 75]	CT	0.95	TCG (S) => TTG (L)	NA	2
nad4	997	333	C	628	[0, 113, 0, 515]	CT	0.82	CTA (L) => TTA (L)	NA	1
nad4	1001	334	C	585	[0, 47, 0, 538]	CT	0.92	CCG (P) => CTG (L)	NA	2
nad4	1007	336	C	632	[0, 50, 0, 582]	CT	0.92	TCA (S) => TTA (L)	NA	2
nad4	1100	367	C	1068	[0, 19, 0, 1049]	CT	0.98	TCA (S) => TTA (L)	NA	2
nad4	1120	374	C	821	[0, 39, 0, 782]	CT	0.95	CTC (L) => TTC (F)	NA	1
nad4	1123	375	C	842	[0, 46, 0, 796]	CT	0.95	CCT (P) => TCT (S)	NA	1
nad4	1142	381	C	759	[0, 39, 0, 720]	CT	0.95	TCC (S) => TTC (F)	NA	2
nad4	1163	388	C	689	[0, 7, 0, 682]	CT	0.99	TCA (S) => TTA (L)	NA	2

nad4	1298	433	C	103	[0, 7, 0, 96]	CT	0.93	GCG (A) => GTG (V)	NA	2
nad4	1346	449	C	8	[0, 0, 0, 8]	CT	1	CCA (P) => CTA (L)	NA	2
nad4	1364	455	C	3	[0, 0, 0, 3]	CT	1	TCC (S) => TTC (F)	NA	2
nad4	1408	470	C	9	[0, 1, 0, 8]	CT	0.89	CAC (H) => TAC (Y)	NA	1
nad4	1424	475	C	7	[0, 3, 0, 4]	CT	0.57	CCG (P) => CTG (L)	NA	2
nad4L	11	4	C	269	[0, 13, 0, 256]	CT	0.95	TCT (S) => TTT (F)	P	2
nad4L	17	6	C	286	[0, 29, 0, 257]	CT	0.9	TCA (S) => TTA (L)	P	2
nad4L	25	9	C	283	[0, 16, 0, 267]	CT	0.94	CGG (R) => TGG (W)	P	1
nad4L	56	19	C	59	[0, 11, 0, 48]	CT	0.81	CCT (P) => CTT (L)	P	2
nad4L	65	22	C	27	[0, 22, 0, 5]	CT	0.19	TCA (S) => TTA (L)	P	2
nad4L	70	24	C	30	[0, 23, 0, 7]	CT	0.23	CCA (P) => TCA (S)	P	1
nad4L	80	27	C	37	[0, 16, 0, 21]	CT	0.57	TCA (S) => TTA (L)	P	2
nad4L	101	34	C	240	[0, 15, 0, 225]	CT	0.94	TCG (S) => TTG (L)	P	2
nad4L	128	43	C	377	[0, 13, 0, 364]	CT	0.97	TCG (S) => TTG (L)	P	2
nad4L	149	50	C	476	[0, 8, 0, 468]	CT	0.98	TCA (S) => TTA (L)	P	2
nad4L	158	53	C	539	[0, 23, 0, 516]	CT	0.96	TCA (S) => TTA (L)	P	2
nad4L	167	56	C	567	[0, 25, 0, 542]	CT	0.96	TCA (S) => TTA (L)	P	2
nad4L	222	74	C	641	[0, 567, 0, 74]	CT	0.12	TTC (F) => TTT (F)	P	3
nad4L	251	84	C	377	[0, 5, 0, 372]	CT	0.99	TCT (S) => TTT (F)	P	2
nad5	155	52	C	48	[0, 11, 0, 37]	CT	0.77	CCG (P) => CTG (L)	NA	2
nad5	242	81	C	239	[0, 21, 0, 218]	CT	0.91	CCG (P) => CTG (L)	NA	2
nad5	359	120	C	440	[0, 12, 0, 428]	CT	0.97	TCT (S) => TTT (F)	NA	2
nad5	374	125	C	896	[0, 26, 0, 870]	CT	0.97	CCA (P) => CTA (L)	NA	2
nad5	398	133	C	942	[0, 17, 0, 925]	CT	0.98	TCT (S) => TTT (F)	NA	2
nad5	539	180	C	1232	[0, 35, 0, 1197]	CT	0.97	CCT (P) => CTT (L)	NA	2
nad5	548	183	C	1280	[0, 33, 0, 1247]	CT	0.97	TCG (S) => TTG (L)	NA	2

nad5	608	203	C	1179	[0, 32, 0, 1147]	CT	0.97	GCC (A) => GTC (V)	NA	2
nad5	609	203	C	1139	[0, 906, 0, 233]	CT	0.2	GCC (A) => GTT (V)	NA	2
nad5	629	210	C	1189	[0, 27, 0, 1162]	CT	0.98	TCT (S) => TTT (F)	NA	2
nad5	676	226	C	1044	[0, 19, 0, 1025]	CT	0.98	CTT (L) => TTT (F)	NA	1
nad5	713	238	C	1203	[0, 16, 0, 1187]	CT	0.99	TCG (S) => TTG (L)	NA	2
nad5	725	242	C	1279	[0, 16, 0, 1263]	CT	0.99	TCA (S) => TTA (L)	NA	2
nad5	835	279	C	1166	[0, 41, 0, 1125]	CT	0.96	CCG (P) => TCG (S)	NA	1
nad5	1310	437	C	103	[0, 5, 0, 98]	CT	0.95	TCA (S) => TTA (L)	NA	2
nad5	1490	497	C	758	[0, 68, 0, 690]	CT	0.91	CCC (P) => CTC (L)	NA	2
nad5	1550	517	C	1199	[0, 95, 0, 1104]	CT	0.92	ACC (T) => ATC (I)	NA	2
nad5	1568	523	C	1197	[0, 80, 0, 1117]	CT	0.93	CCG (P) => CTG (L)	NA	2
nad5	1580	527	C	1191	[0, 84, 0, 1107]	CT	0.93	TCA (S) => TTA (L)	NA	2
nad5	1589	530	C	1103	[0, 110, 0, 993]	CT	0.9	TCT (S) => TTT (F)	NA	2
nad5	1610	537	C	1351	[0, 223, 0, 1128]	CT	0.83	TCC (S) => TTC (F)	NA	2
nad5	1695	565	C	684	[0, 586, 0, 98]	CT	0.14	TTC (F) => TTT (F)	NA	3
nad5	1895	632	C	78	[0, 14, 0, 64]	CT	0.82	TCA (S) => TTA (L)	NA	2
nad5	1916	639	C	54	[0, 12, 0, 42]	CT	0.78	TCT (S) => TTT (F)	NA	2
nad5	1918	640	C	56	[0, 14, 0, 42]	CT	0.75	CGT (R) => TGT (C)	NA	1
nad5	1958	653	C	33	[0, 4, 0, 29]	CT	0.88	TCG (S) => TTG (L)	NA	2
nad5	1981	661	C	30	[0, 3, 0, 27]	CT	0.9	CGT (R) => TGT (C)	NA	1
nad6	26	9	C	2037	[0, 114, 1, 1922]	CT CG	0.94	CCT (P) => CTT (L)	NA	2
nad6	88	30	C	1440	[0, 25, 0, 1415]	CT	0.98	CCC (P) => TTC (F)	NA	1
nad6	89	30	C	1457	[0, 31, 0, 1426]	CT	0.98	CCC (P) => TTC (F)	NA	2
nad6	95	32	C	1467	[0, 95, 0, 1372]	CT	0.94	CCA (P) => CTA (L)	NA	2
nad6	103	35	C	1668	[0, 32, 0, 1636]	CT	0.98	CGC (R) => TGC (C)	NA	1
nad6	161	54	C	2957	[0, 62, 0, 2895]	CT	0.98	CCA (P) => CTA (L)	NA	2

nad6	169	57	C	3157	[0, 48, 0, 3109]	CT	0.98	CAT (H) => TAT (Y)	NA	1
nad6	191	64	C	3983	[0, 27, 0, 3956]	CT	0.99	TCA (S) => TTA (L)	NA	2
nad6	306	102	C	6191	[1, 4510, 0, 1680]	CT CA	0.27	TTC (F) => TTT (F)	NA	3
nad6	463	155	C	1391	[0, 14, 0, 1377]	CT	0.99	CCT (P) => TCT (S)	NA	1
nad6	569	190	C	101	[0, 2, 0, 99]	CT	0.98	TCT (S) => TTT (F)	NA	2
nad7	45	15	C	147	[0, 115, 0, 32]	CT	0.22	TTC (F) => TTT (F)	NA	3
nad7	77	26	C	70	[0, 13, 0, 57]	CT	0.81	TCA (S) => TTA (L)	NA	2
nad7	137	46	C	18	[0, 5, 0, 13]	CT	0.72	TCA (S) => TTA (L)	NA	2
nad7	200	67	C	17	[0, 3, 0, 14]	CT	0.82	TCT (S) => TTT (F)	NA	2
nad7	209	70	C	17	[0, 9, 0, 8]	CT	0.47	TCA (S) => TTA (L)	NA	2
nad7	244	82	C	1731	[0, 48, 0, 1683]	CT	0.97	CAT (H) => TAT (Y)	NA	1
nad7	251	84	C	1743	[0, 59, 0, 1684]	CT	0.97	TCA (S) => TTA (L)	NA	2
nad7	316	106	C	3061	[0, 28, 0, 3033]	CT	0.99	CGT (R) => TGT (C)	NA	1
nad7	335	112	C	3450	[0, 21, 0, 3429]	CT	0.99	TCA (S) => TTA (L)	NA	2
nad7	344	115	C	3572	[0, 68, 0, 3504]	CT	0.98	TCA (S) => TTA (L)	NA	2
nad7	383	128	C	3372	[0, 34, 0, 3338]	CT	0.99	TCA (S) => TTA (L)	NA	2
nad7	531	177	C	2232	[0, 1905, 0, 327]	CT	0.15	TCC (S) => TCT (S)	NA	3
nad7	534	178	C	2263	[0, 1936, 0, 327]	CT	0.14	TTC (F) => TTT (F)	NA	3
nad7	578	193	C	1586	[0, 17, 0, 1569]	CT	0.99	TCA (S) => TTA (L)	NA	2
nad7	724	242	C	744	[0, 24, 0, 720]	CT	0.97	CAT (H) => TAT (Y)	NA	1
nad7	739	247	C	599	[0, 26, 0, 573]	CT	0.96	CCT (P) => TTT (F)	NA	1
nad7	740	247	C	609	[0, 24, 0, 585]	CT	0.96	CCT (P) => TTT (F)	NA	2
nad7	769	257	C	487	[0, 17, 0, 470]	CT	0.97	CGC (R) => TGC (C)	NA	1
nad7	926	309	C	1810	[0, 1545, 0, 265]	CT	0.15	TCA (S) => TTA (L)	NA	2
nad7	944	315	C	2402	[0, 51, 0, 2351]	CT	0.98	CCT (P) => CTT (L)	NA	2
nad7	1050	350	C	1882	[0, 44, 0, 1838]	CT	0.98	CCC (P) => CCT (P)	NA	3

nad7	1057	353	C	1854	[0, 110, 0, 1744]	CT	0.94	CGT (R) => TGT (C)	NA	1
nad7	1103	368	C	745	[0, 5, 0, 740]	CT	0.99	TCT (S) => TTT (F)	NA	2
nad7	1124	375	C	558	[0, 0, 0, 558]	CT	1	CCA (P) => CTA (L)	NA	2
nad7	1137	379	C	473	[0, 362, 0, 111]	CT	0.23	GTC (V) => GTT (V)	NA	3
nad7	1166	389	C	149	[0, 10, 0, 139]	CT	0.93	TCT (S) => TTT (F)	NA	2
nad9	15	5	C	4345	[0, 95, 0, 4250]	CT	0.98	TTC (F) => TTT (F)	P	3
nad9	92	31	C	4365	[0, 37, 0, 4328]	CT	0.99	TCT (S) => TTT (F)	P	2
nad9	113	38	C	5111	[0, 253, 0, 4858]	CT	0.95	CCA (P) => CTA (L)	P	2
nad9	167	56	C	6302	[0, 63, 0, 6239]	CT	0.99	TCG (S) => TTG (L)	P	2
nad9	298	100	C	9255	[0, 68, 1, 9186]	CT CG	0.99	CCG (P) => TCG (S)	P	1
nad9	328	110	C	7221	[1, 52, 1, 7167]	CT CA CG	0.99	CGG (R) => TGG (W)	P	1
nad9	368	123	C	5797	[0, 28, 0, 5769]	CT	1	TCC (S) => TTC (F)	P	2
nad9	398	133	C	3982	[0, 14, 0, 3968]	CT	1	TCA (S) => TTA (L)	P	2
nad9	439	147	C	2185	[0, 66, 0, 2119]	CT	0.97	CTT (L) => TTT (F)	P	1
rpl5	8	3	C	550	[0, 411, 0, 139]	CT	0.25	CCA (P) => CTA (L)	N	2
rpl5	35	12	C	596	[0, 71, 0, 525]	CT	0.88	TCA (S) => TTA (L)	P	2
rpl5	47	16	C	636	[0, 121, 0, 515]	CT	0.81	CCG (P) => CTG (L)	P	2
rpl5	64	22	C	692	[0, 390, 0, 302]	CT	0.44	CAC (H) => TAC (Y)	N	1
rpl5	160	54	C	769	[0, 90, 0, 679]	CT	0.88	CCG (P) => TTG (L)	P	1
rpl5	161	54	C	795	[0, 658, 0, 137]	CT	0.17	CCG (P) => TTG (L)	N	2
rpl5	441	147	C	549	[0, 178, 0, 371]	CT	0.68	ATC (I) => ATT (I)	N	3
rpl5	509	170	C	396	[0, 31, 0, 365]	CT	0.92	CCA (P) => CTA (L)	P	2
rpl5	512	171	C	382	[0, 27, 0, 355]	CT	0.93	CCG (P) => CTG (L)	P	2
rpl5	529	177	C	398	[0, 29, 0, 369]	CT	0.93	CAA (Q) => TAA (*)	P	1
rpl5	666	222	C	195	[0, 159, 0, 36]	CT	0.18	ATC (I) => ATT (I)	N	3

rpl10	101	34	C	361	[0, 64, 0, 297]	CT	0.82	TCA (S) => TTA (L)	NA	2
rpl10	134	45	C	342	[0, 170, 0, 172]	CT	0.5	CCA (P) => CTA (L)	NA	2
rpl16	221	74	C	414	[0, 1, 0, 413]	CT	1	TCG (S) => TTG (L)	P	2
rps3	92	31	C	484	[0, 13, 0, 471]	CT	0.97	TCA (S) => TTA (L)	NA	2
rps3	512	171	C	1912	[0, 89, 0, 1823]	CT	0.95	TCA (S) => TTA (L)	NA	2
rps3	713	238	C	1950	[0, 60, 0, 1890]	CT	0.97	TCG (S) => TTG (L)	NA	2
rps3	986	329	C	1603	[0, 1299, 0, 304]	CT	0.19	TCG (S) => TTG (L)	NA	2
rps3	1022	341	C	1013	[1, 9, 0, 1003]	CT CA	0.99	CCA (P) => CTA (L)	NA	2
rps3	1355	452	C	2246	[0, 18, 0, 2228]	CT	0.99	CCG (P) => CTG (L)	NA	2
rps3	1496	499	C	979	[0, 20, 0, 959]	CT	0.98	TCA (S) => TTA (L)	NA	2
rps3	1582	528	C	584	[0, 9, 0, 575]	CT	0.98	CCT (P) => TCT (S)	NA	1
rps4	176	59	C	882	[0, 37, 0, 845]	CT	0.96	TCA (S) => TTA (L)	NA	2
rps4	205	69	C	745	[0, 30, 0, 715]	CT	0.96	CAT (H) => TAT (Y)	NA	1
rps4	219	73	C	736	[0, 148, 0, 588]	CT	0.8	CCC (P) => CCT (P)	NA	3
rps4	275	92	C	691	[0, 20, 0, 671]	CT	0.97	CCA (P) => CTA (L)	NA	2
rps4	287	96	C	661	[0, 13, 0, 648]	CT	0.98	TCG (S) => TTG (L)	NA	2
rps4	299	100	C	727	[0, 17, 0, 710]	CT	0.98	CCG (P) => CTG (L)	NA	2
rps4	316	106	C	726	[0, 102, 0, 624]	CT	0.86	CGT (R) => TGT (C)	NA	1
rps4	344	115	C	730	[0, 14, 0, 716]	CT	0.98	CCG (P) => CTG (L)	NA	2
rps4	443	148	C	901	[0, 770, 0, 131]	CT	0.15	GCG (A) => GTG (V)	NA	2
rps4	483	161	C	1072	[0, 924, 0, 148]	CT	0.14	ATC (I) => ATT (I)	NA	3
rps4	491	164	C	1038	[1, 37, 0, 1000]	CT CA	0.96	TCA (S) => TTA (L)	NA	2
rps4	592	198	C	1889	[0, 1421, 0, 468]	CT	0.25	CGC (R) => TGC (C)	NA	1
rps4	848	283	C	495	[0, 443, 0, 52]	CT	0.11	TCG (S) => TTG (L)	NA	2
rps4	852	284	C	484	[0, 371, 0, 113]	CT	0.23	ACC (T) => ACT (T)	NA	3
rps4	941	314	C	237	[0, 3, 0, 234]	CT	0.99	TCG (S) => TTG (L)	NA	2

rps4	952	318	C	223	[0, 8, 0, 215]	CT	0.96	CAT (H) => TAT (Y)	NA	1
rps4	962	321	C	182	[0, 15, 0, 167]	CT	0.92	CCA (P) => CTA (L)	NA	2
rps4	977	326	C	165	[0, 8, 0, 157]	CT	0.95	TCT (S) => TTT (F)	NA	2
rps4	1028	343	C	60	[0, 1, 0, 59]	CT	0.98	CCA (P) => CTA (L)	NA	2
rps4	1042	348	C	46	[0, 2, 0, 44]	CT	0.96	CGG (R) => TGG (W)	NA	1
rps10	102	34	C	186	[0, 13, 0, 173]	CT	0.93	GTC (V) => GTT (V)	NA	3
rps10	132	44	C	120	[0, 107, 0, 13]	CT	0.11	TCC (S) => TCT (S)	NA	3
rps10	210	70	C	44	[0, 32, 0, 12]	CT	0.27	TTC (F) => TTT (F)	NA	3
rps10	214	72	C	38	[0, 0, 0, 38]	CT	1	CGG (R) => TGG (W)	NA	1
rps10	278	93	C	19	[0, 0, 0, 19]	CT	1	TCG (S) => TTG (L)	NA	2
rps10	307	103	C	9	[0, 0, 0, 9]	CT	1	CGA (O) => TGA (*)	NA	1
rps12	104	35	C	1162	[0, 20, 0, 1142]	CT	0.98	CCG (P) => CTG (L)	NA	2
rps12	159	53	C	571	[0, 474, 0, 97]	CT	0.17	GCC (A) => GCT (A)	NA	3
rps12	196	66	C	366	[0, 4, 0, 362]	CT	0.99	CAC (H) => TAC (Y)	NA	1
rps12	221	74	C	239	[0, 10, 0, 229]	CT	0.96	TCG (S) => TTG (L)	NA	2
rps12	284	95	C	73	[0, 1, 0, 72]	CT	0.99	TCC (S) => TTC (F)	NA	2
rps13	56	19	C	1136	[0, 41, 0, 1095]	CT	0.96	TCA (S) => TTA (L)	P	2
rps13	100	34	C	901	[0, 34, 0, 867]	CT	0.96	CGT (R) => TGT (C)	P	1
rps13	287	96	C	140	[0, 1, 0, 139]	CT	0.99	TCG (S) => TTG (L)	P	2

Table S9 Summary of single nucleotide polymorphisms (SNPs) detected in the PCGs of *S. officinalis* mitogenome. “Nt Pos” means the nucleotide position of a SNP in a PCG.

Gene	Nt Pos	Nt reference	Nt coverage	Nt Base Count [A,C,G,T]	Nt allsubs	Nt Frequency
ccmFn	1417	G	152	[15, 0, 137, 0]	GA	0.1
ccmFn	1448	C	161	[0, 131, 0, 30]	CT	0.19
ccmFn	1449	C	157	[10, 126, 21, 0]	CG CA	0.14
ccmFn	1457	A	154	[135, 0, 19, 0]	AG	0.12
ccmFn	1471	C	156	[0, 136, 0, 20]	CT	0.13
ccmFn	1486	G	157	[29, 0, 128, 0]	GA	0.18
ccmFn	1496	C	153	[0, 137, 0, 16]	CT	0.1
ccmFn	1818	G	113	[21, 0, 92, 0]	GA	0.19
ccmc	45	C	122	[0, 110, 0, 12]	CT	0.1
ccmc	179	C	165	[19, 146, 0, 0]	CA	0.12
ccmc	190	A	167	[151, 0, 16, 0]	AG	0.1
rpl5	413	C	279	[0, 251, 0, 28]	CT	0.1
atp9	35	C	132	[13, 119, 0, 0]	CA	0.1
atp9	125	C	145	[0, 128, 0, 17]	CT	0.12
cox2	149	C	56	[0, 49, 0, 7]	CT	0.12
cox2	153	C	49	[0, 43, 0, 6]	CT	0.12
atp1	906	C	739	[0, 188, 0, 551]	CT	0.75
atp1	912	A	712	[191, 0, 521, 0]	AG	0.73
atp1	918	C	719	[533, 186, 0, 0]	CA	0.74
atp1	921	T	715	[0, 537, 0, 178]	TC	0.75
atp1	924	C	614	[0, 186, 0, 428]	CT	0.7
atp1	936	G	666	[0, 480, 186, 0]	GC	0.72
atp1	1002	A	186	[158, 0, 28, 0]	AG	0.15
atp1	1023	T	1096	[0, 919, 0, 177]	TC	0.84
atp1	1590	C	99	[0, 88, 11, 0]	CG	0.11
atp1	1604	T	435	[0, 83, 0, 352]	TC	0.19
rps3	21	A	118	[94, 0, 24, 0]	AG	0.2
rps3	27	G	121	[22, 0, 99, 0]	GA	0.18
rpl16	15	C	149	[0, 133, 0, 16]	CT	0.11
nad3	12	T	184	[0, 43, 0, 141]	TC	0.23
nad3	29	A	137	[123, 14, 0, 0]	AC	0.1
nad3	30	T	142	[14, 0, 0, 128]	TA	0.1
nad3	45	G	144	[0, 14, 130, 0]	GC	0.1
nad3	48	A	137	[123, 0, 14, 0]	AG	0.1
nad3	51	T	139	[0, 0, 14, 125]	TG	0.1
nad3	60	T	172	[0, 40, 0, 132]	TC	0.23
nad3	62	C	170	[0, 131, 0, 39]	CT	0.23
nad3	99	G	183	[46, 0, 137, 0]	GA	0.25
matR	1496	G	164	[16, 0, 148, 0]	GA	0.1
matR	1526	T	129	[0, 17, 0, 112]	TC	0.13
ccmFc	583	A	217	[184, 33, 0, 0]	AC	0.15
ccmFc	620	C	218	[0, 192, 26, 0]	CG	0.12
ccmFc	405	G	163	[19, 0, 144, 0]	GA	0.12

Table S10 PCR primers used to validate the RNA editing sites from eleven PCGs in the *S. officinalis* mitogenome.

Primer Name	PCG name	Primer Sequence
so-atp4-F1	atp4	CCACTTCCCGTTCAGTTGCT
so-atp4-R1	atp4	TGGATCCGCTCTTCTGTGAAC
so-atp8-F1	atp8	ACACAATTCTTCTGGTCATGCCT
so-atp8-R1	atp8	GCTCTTCCGAACGACCAAGT
so-cox1-F1	cox1	GGGGAGCGGAACCTTTTTAC
so-cox1-R1	cox1	ACAGTCCCAGCATATAGCGCA
so-cox1-F2	cox1	AGTAGAAGAGAGAAGCACCCTG
so-cox1-R2	cox1	AGTAGCAATCGGCGACCTTT
so-cox3-F1	cox3	ACAACCGAGGCAAAGTGGTT
so-cox3-R1	cox3	GGAGTTCTCTTTGTCTTCGAGC
so-mttB-F1	mttB	TAAAGATGCGCCGGTCGTAG
so-mttB-R1	mttB	TGTTGGTTGAGAATTGCTCTGG
so-nad3-F1	nad3	TTTCCGGTATGTAGCTCCGC
so-nad3-R1	nad3	GCATCGCTCTTTCCTTTGTCC
so-nad4L-F1	nad4L	ATTCTACGTTCCCGACACGG
so-nad4L-R1	nad4L	AAGAAAACGAAAGGAGAAATTCGT
so-nad9-F1	nad9	CTTCATTTTTAGAGGAAGAAGCGGA
so-nad9-R1	nad9	TTCCTATTGATTTGTCTCCTGGACT
so-rpl5-F1	rpl5	CTTTTTGAAGGCGATAGTTCACAGT
so-rpl5-R1	rpl5	TGGATCAATAGAAAAGGGGCTCT
so-rpl16-F1	rpl16	CTTTCATATCGAGCCATTGAAGCA
so-rpl16-R1	rpl16	TTTTGAATCCCGGTCCGGTT
so-rps13-F1	rps13	ACAGCCCATTTCTGATTCCAGC
so-rps13-R1	rps13	TCTTCAGCAGGTAGGCTGCAC

Figure S1 The alignment of the Nanopore reads to the four conformations of the three double bifurcating structures (DBS), named DBS01-DBS03, found in the unitig graph. The unitig graph was generated using Unicycler from Illumina reads that were filtered with GetOrganelle for mitochondrial reads. The major conformations (Mac) refer to the conformations in the DBS structure supported by more long reads. The minor conformations (Mic) refer to the conformations in the DBS structure supported by less long reads. It should be emphasized that the repetitive sequences affiliated with DBS01, DBS02, and DBS03 are identical to the high-scoring pairs (HSPs): r01, r02, and r47, respectively. Each figure can be divided into three parts from top to bottom. The top part shows the reference sequences with their coordinates. The middle part shows the read coverage bar chart in blue. The name and length of the repeat and the name of the conformation are shown above the coverage plot. The bottom part shows the reads' alignment to the reference sequences. The repeat region is shown as a double-headed arrow. The boundaries of the repeat regions of the DBS structure are indicated with red vertical lines.

a Mapping of long reads to the major conformation Mac1 of the DBS01/r01.

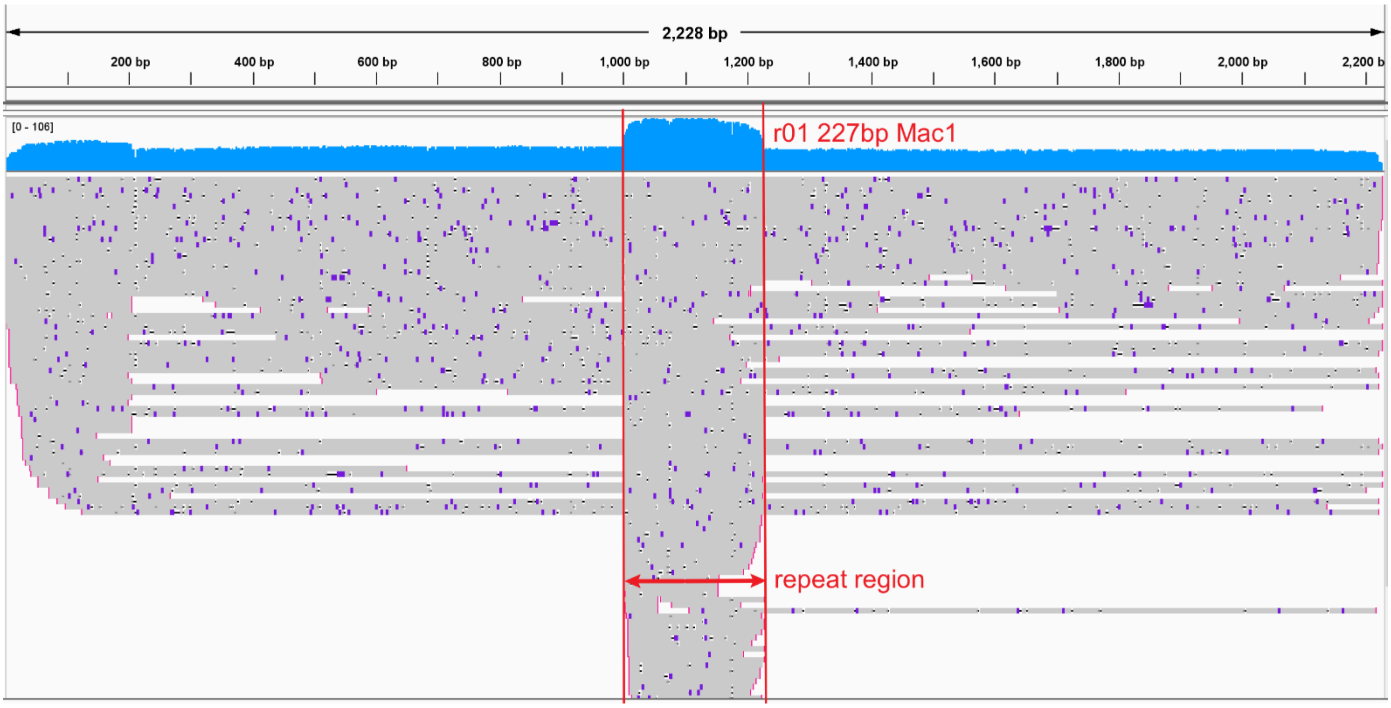

b Mapping of long reads to the major conformation Mac2 of the DBS01/r01.

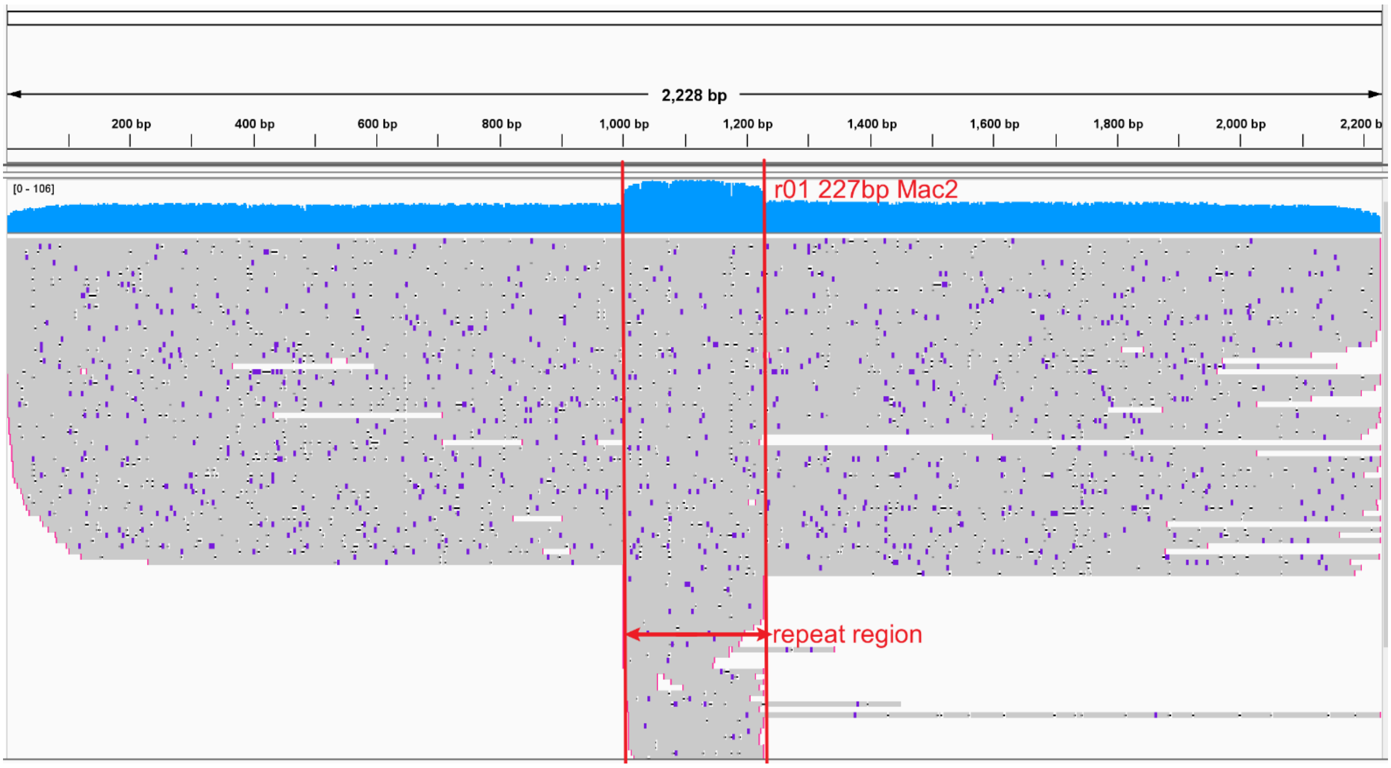

c Mapping of long reads to the minor conformation Mic1 of the DBS01/r01.

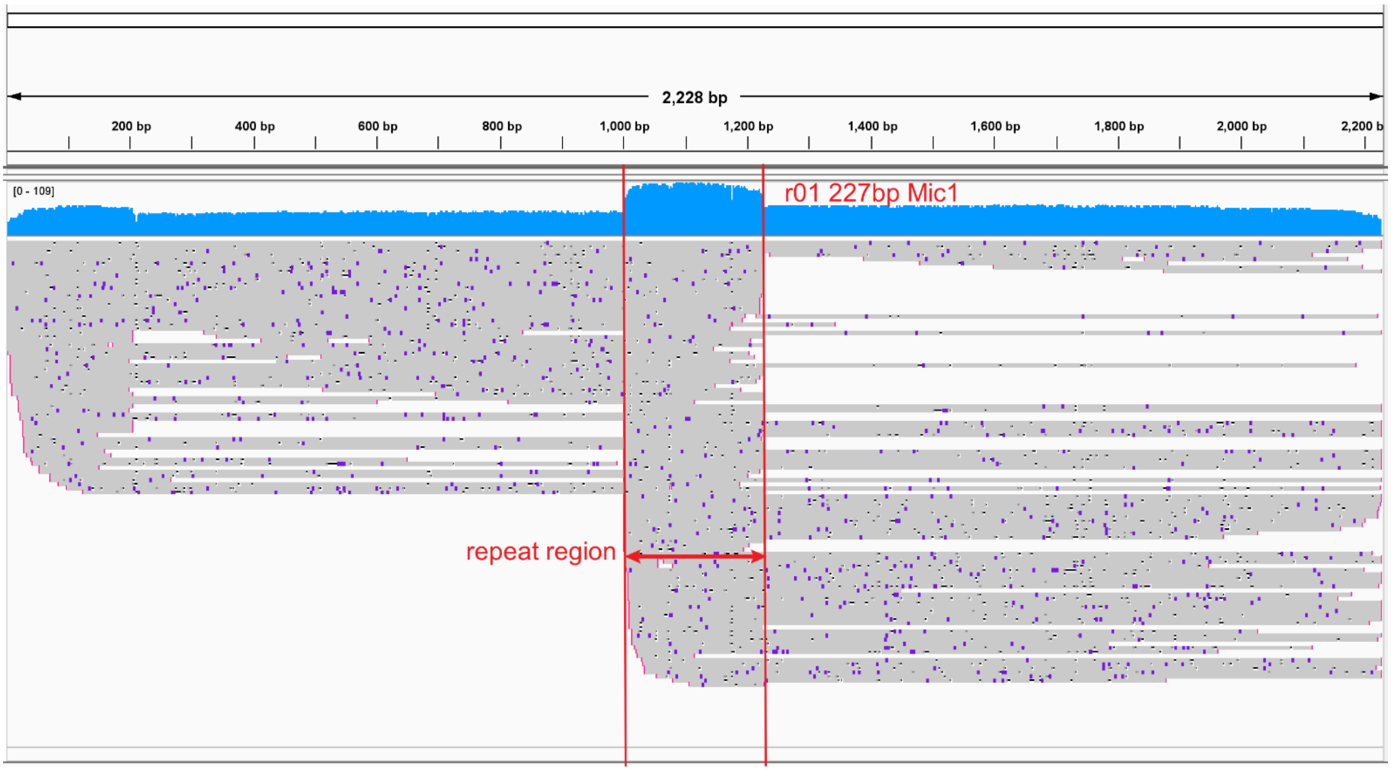

d Mapping of long reads to the minor conformation Mic2 of the DBS01/r01.

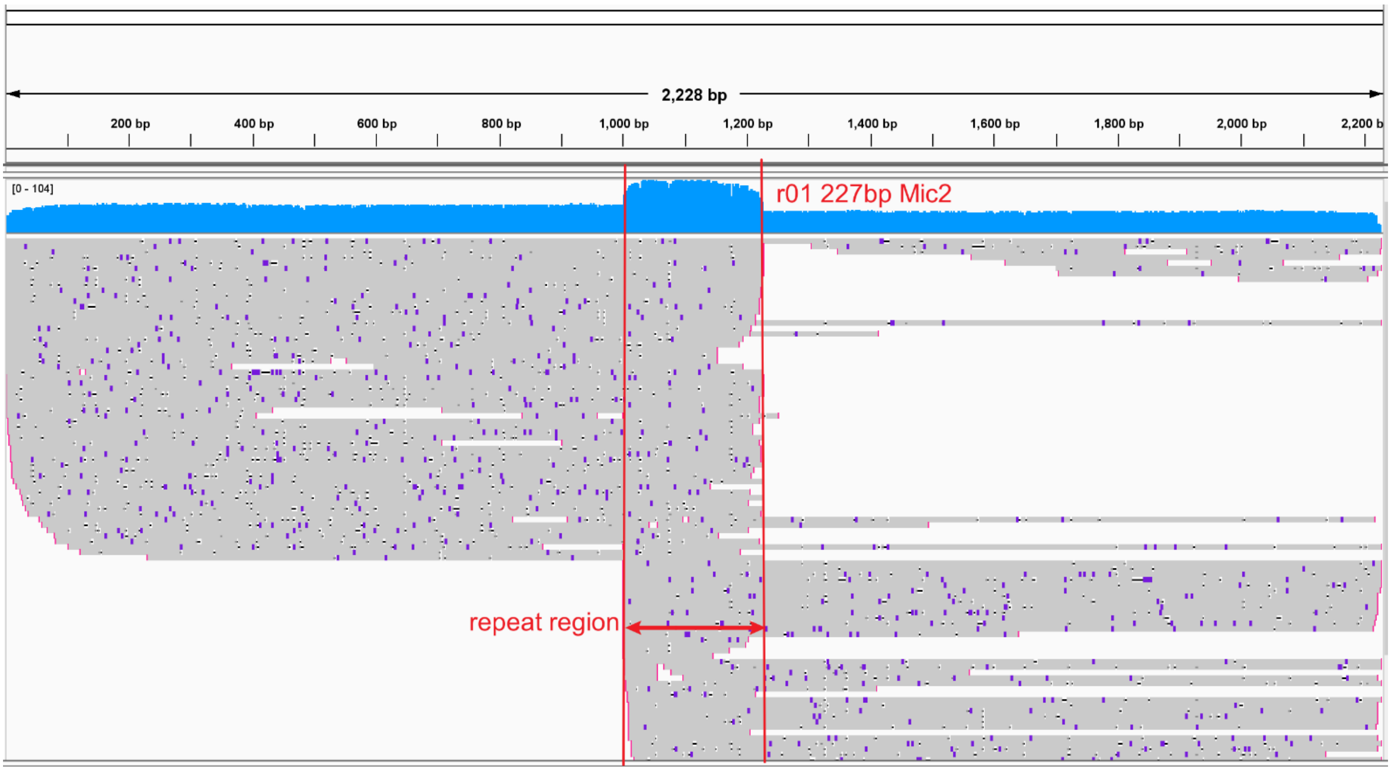
2,228 bp
200 bp
400 bp
600 bp
800 bp
1,000 bp
1,200 bp
1,400 bp
1,600 bp
1,800 bp
2,000 bp
[0 - 104]
r01 227bp Mic2
repeat region

e Mapping of long reads to the major conformation Mac1 of the DBS02/r02.

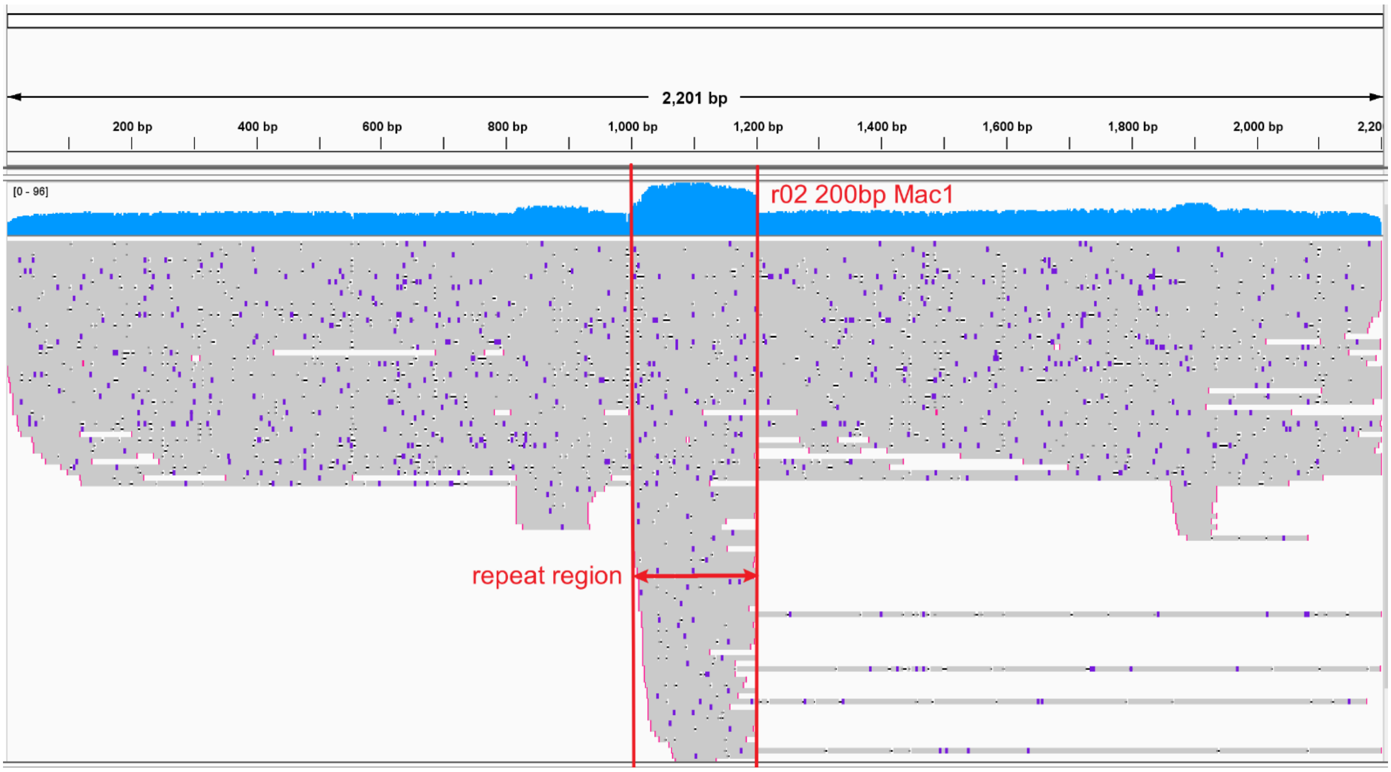

f Mapping of long reads to the major conformation Mac2 of the DBS02/r02.

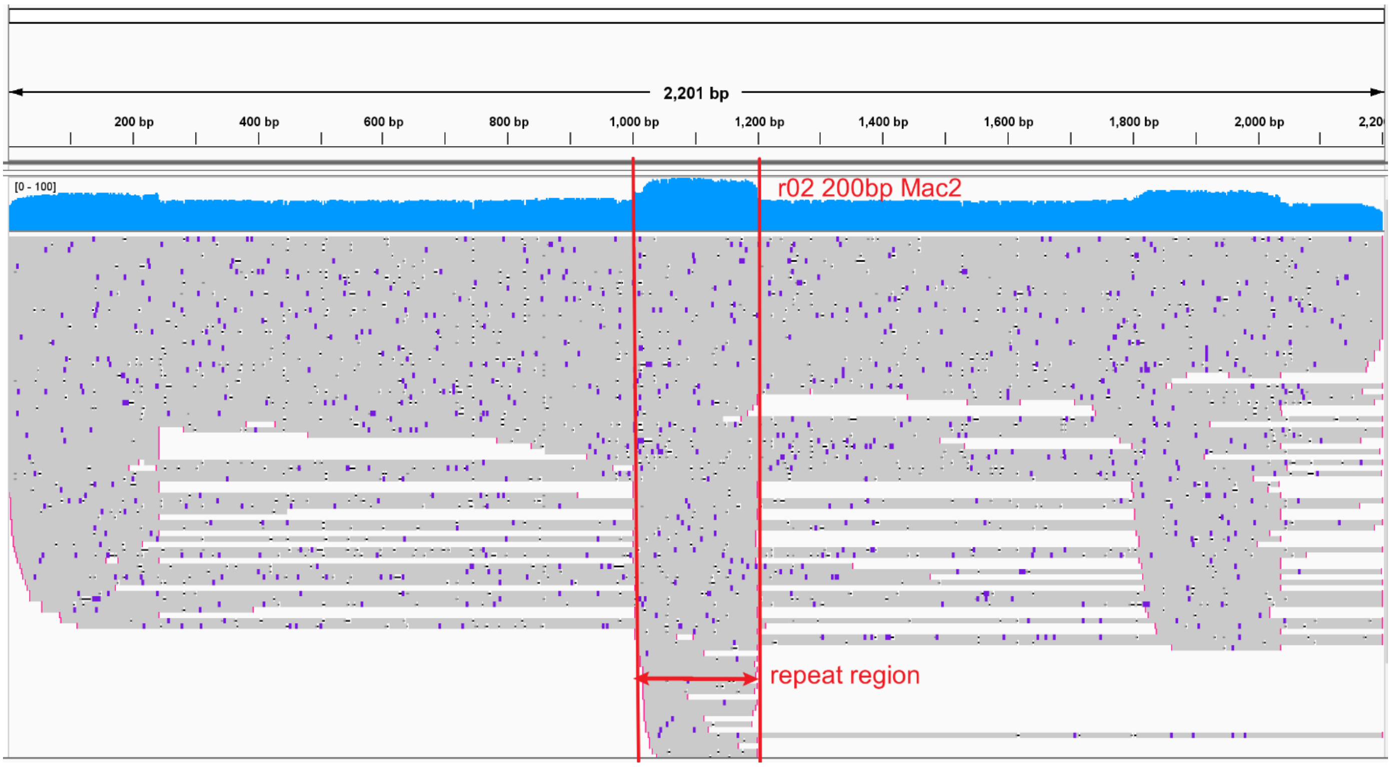

g Mapping of long reads to the minor conformation Mic1 of the DBS02/r02.

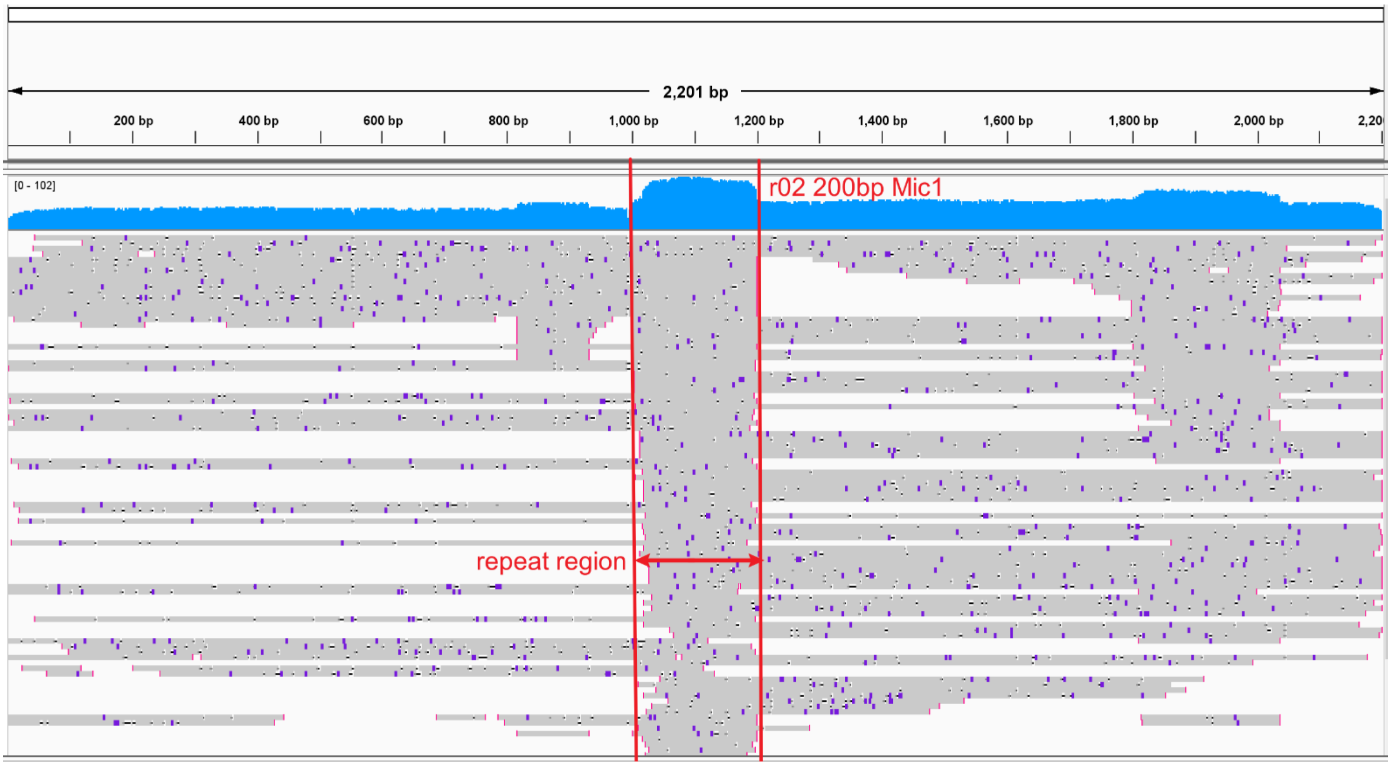

h Mapping of long reads to the minor conformation Mic1 of the DBS02/r02.

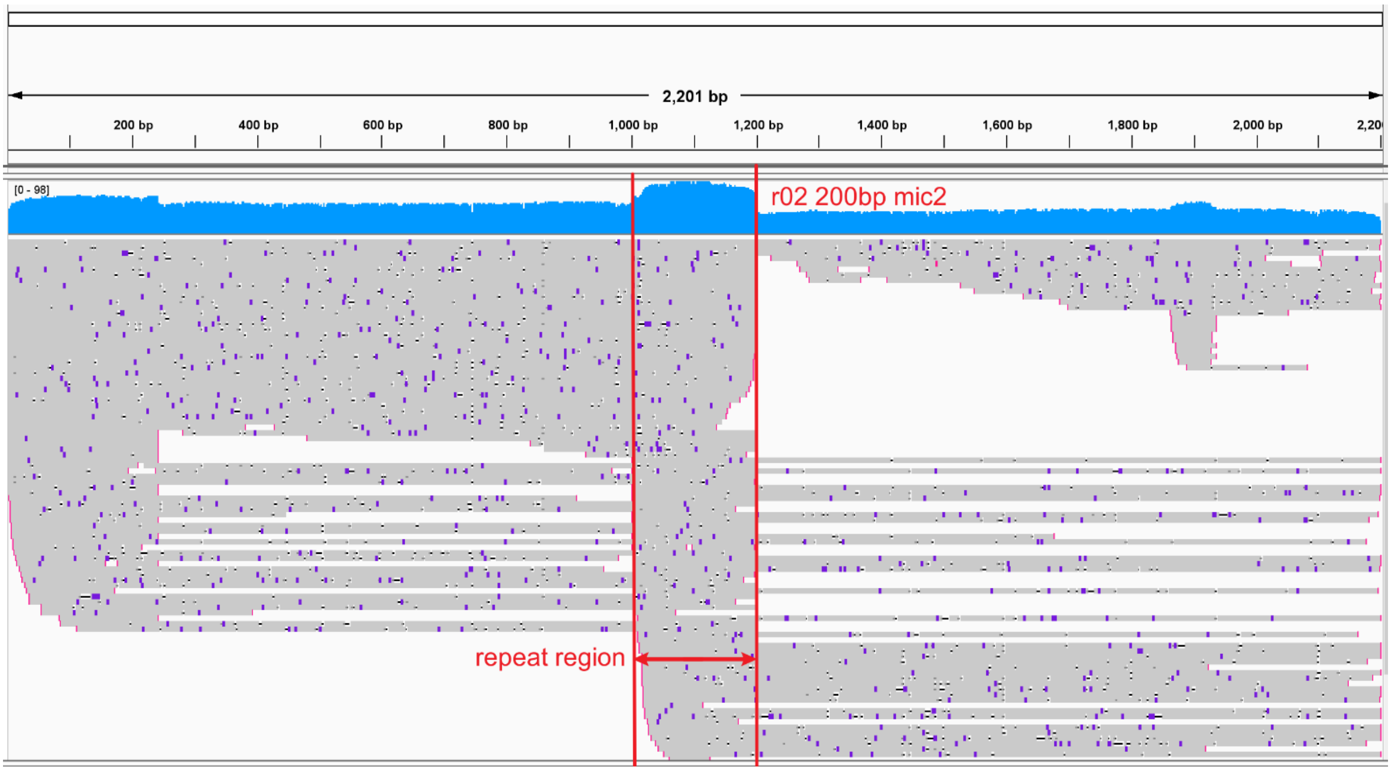

i Mapping of long reads to the major conformation Mac1 of the DBS03/r47.

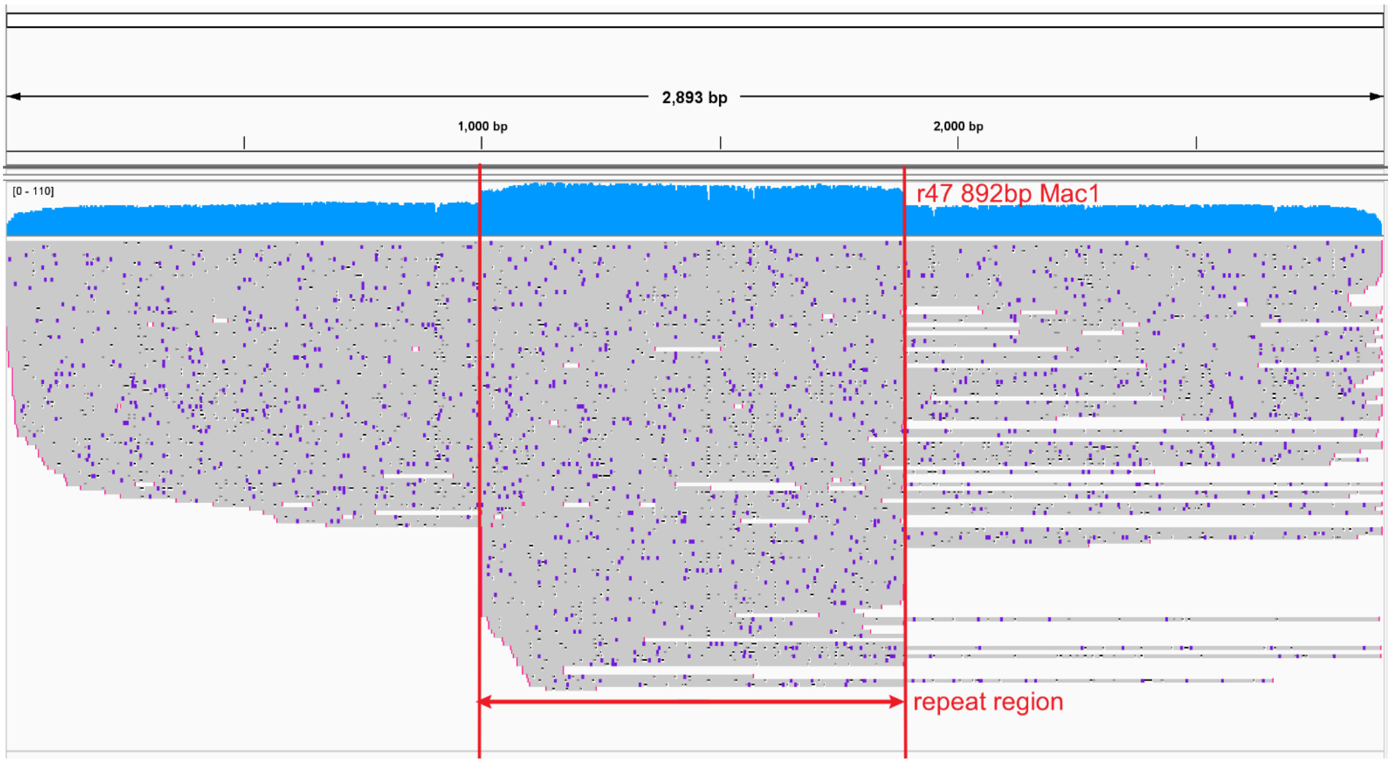

j Mapping of long reads to the major conformation Mac2 of the DBS03/r47.

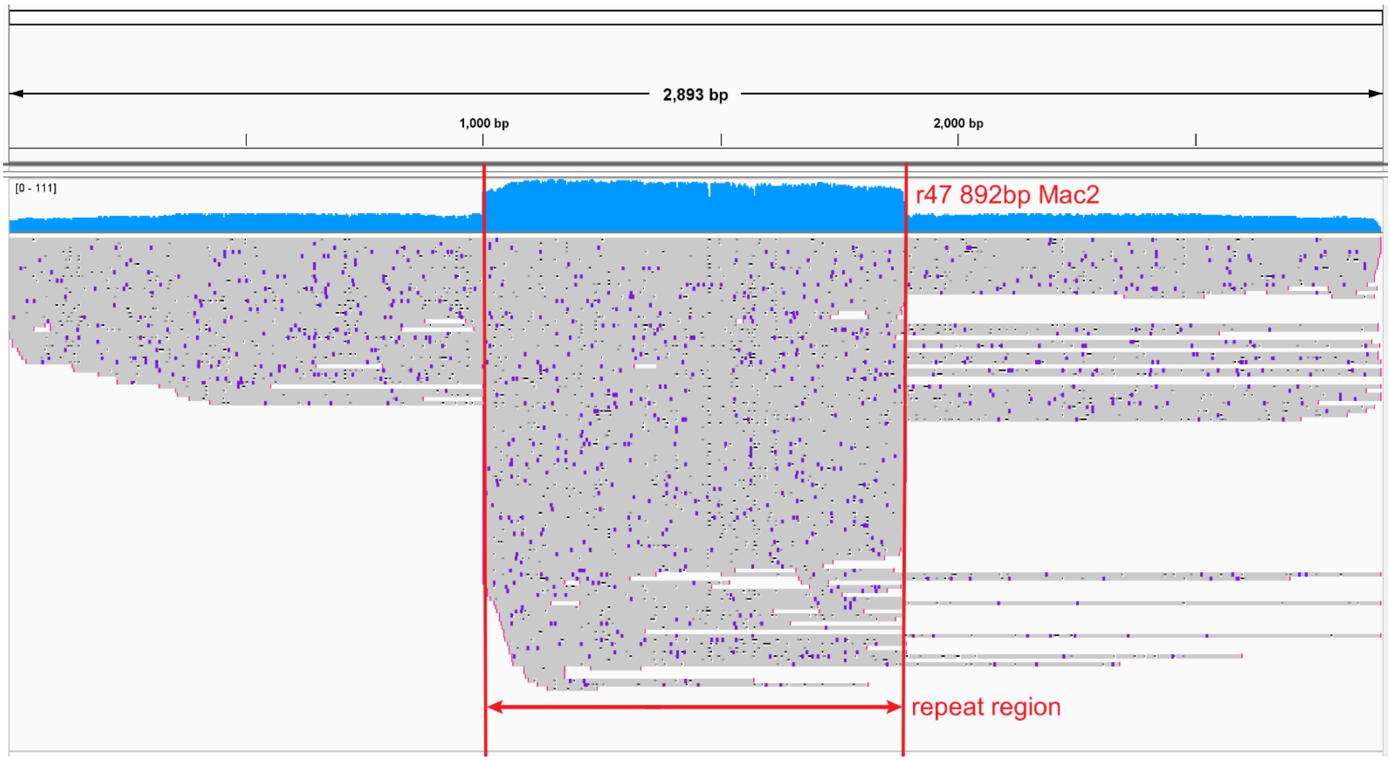

k Mapping of long reads to the minor conformation Mic1 of the DBS03//r47.

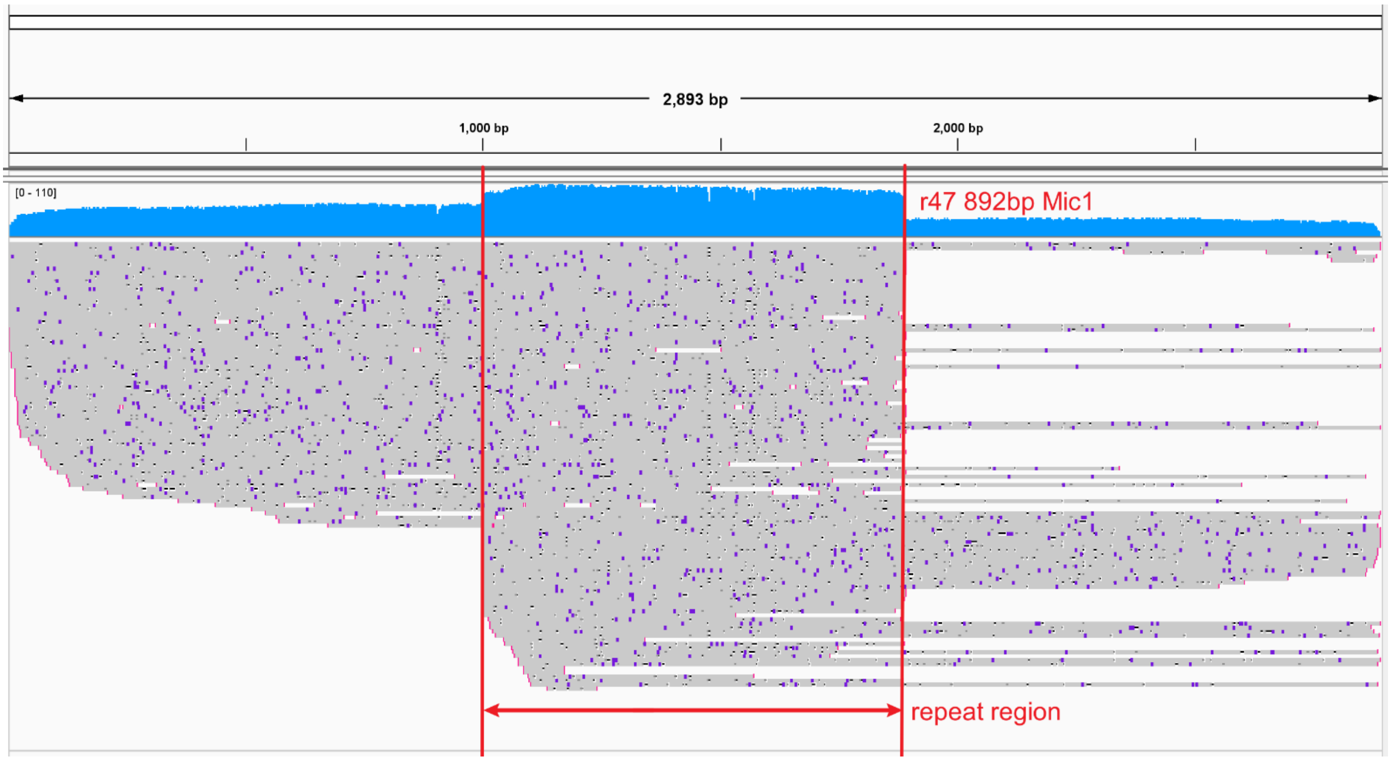

l Mapping of long reads to the minor conformation Mic1 of the DBS03/r47.

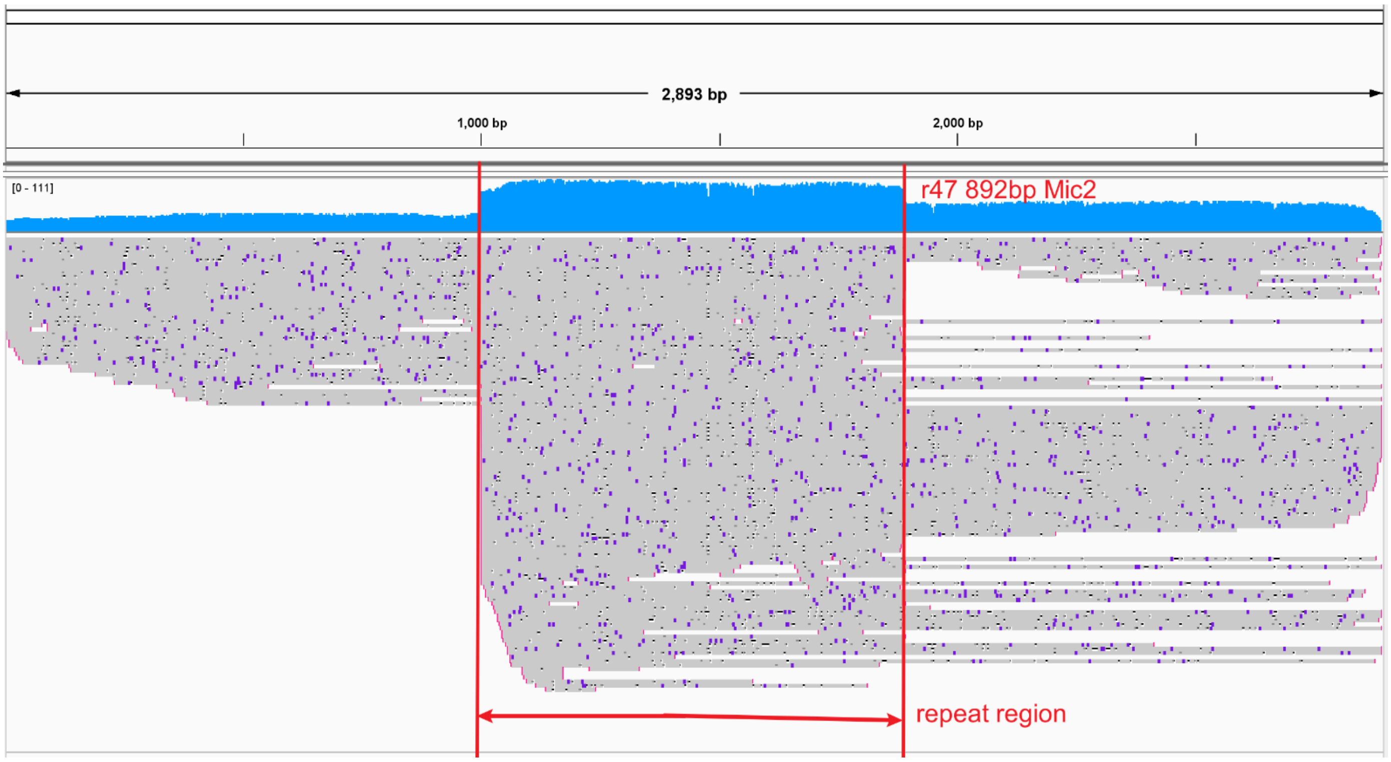

Figure S2 Mapping results of Nanopore and Illumina reads to the mitochondrial chromosomes 1 (MC1) and 2 (MC2) of the *S. officinalis*. Panels a and b show the mapping results of the Nanopore reads to MC1 and MC2, respectively. Panels c and d show the mapping results of all Illumina reads to the MC1 and MC2, respectively. The X-axis shows the nucleotide position, and the Y-axis shows the corresponding coverage depth.

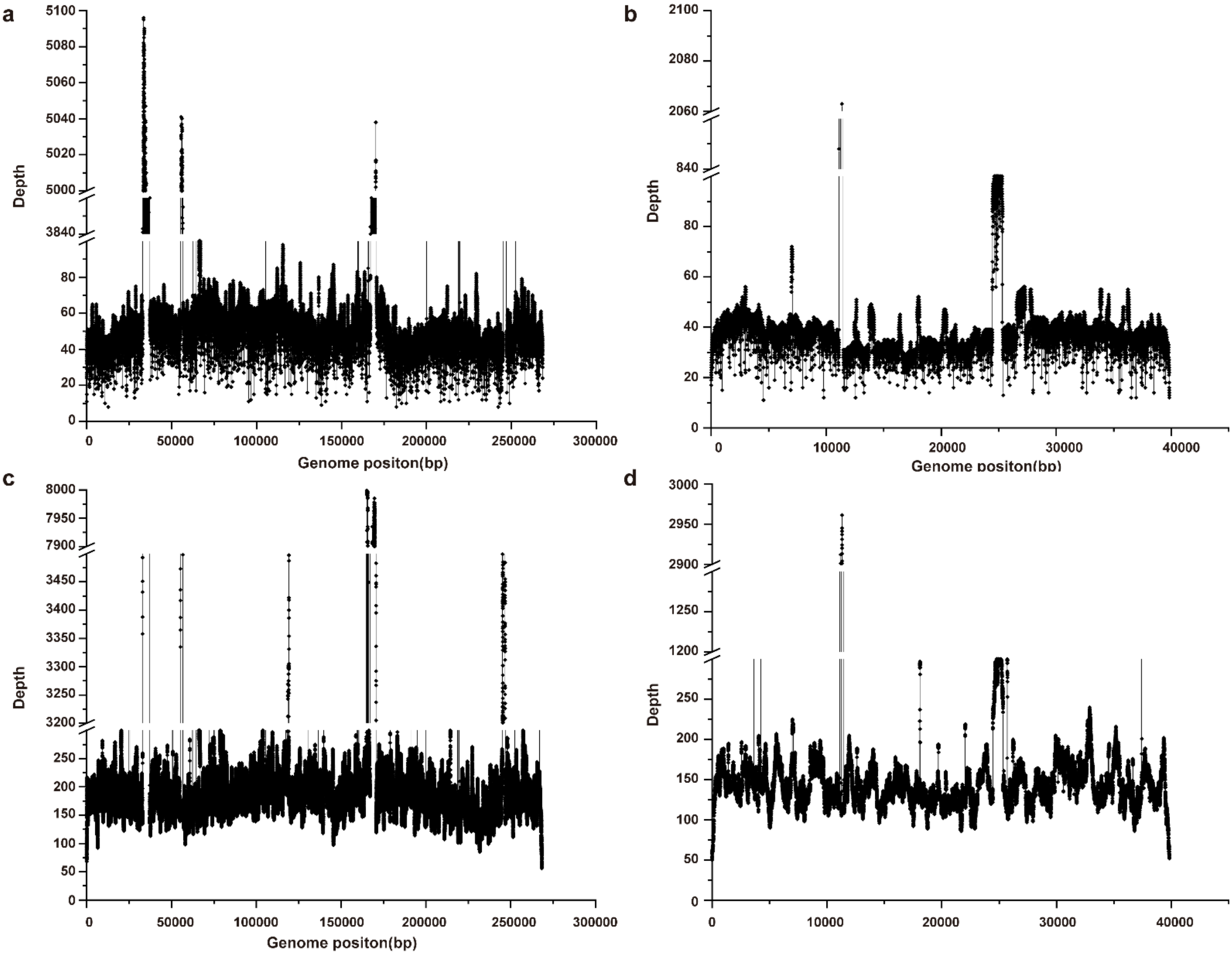

Figure S3 Validation of the presence of the four conformations c1-4 of DBS01, DBS02, and DBS03, corresponding to the recombination products mediated by the three repeats R01, R02, and R47. PCR primers were designed based on the four conformations. The genomic DNA were then amplified by PCR, and the PCR products were subject to Sanger sequencing. The sequencing chromatograms, the Sanger sequencing results (labeled with "PCR" and conformation number), the expected sequences (labeled with repeat id and conformation number), and the consensus sequences are shown at the top of chromatograms and below the chromatograms. Panels a-i correspond to DBS01, DBS02, and DBS03, affiliated with the repetitive sequences r01, r02, and r47. The sequences of r01, r02, and r047 were high-lighted in bold red.

a comparison of the sequence obtained from PCR amplification and Sanger sequencing (R01-C1) and the expected sequence.

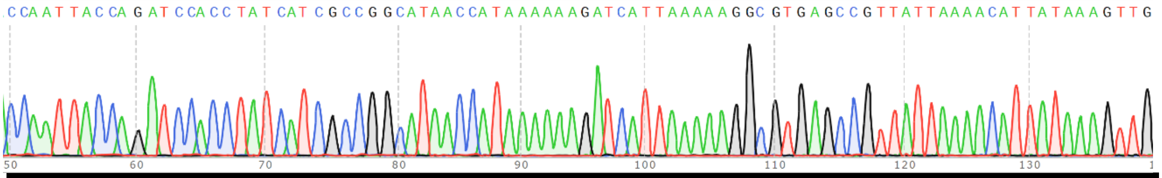

PCR_C1 : CCAATTACCAGATCCACCTATCATCGCCGGCATAACCATAAAAAAGATCATTAAAAAGGCGTGAGCCGTTATTAAAACATTATAAAGTTG : 90
R01_C1 : CCAATTACCAGATCCACCTATCATCGCCGGCATAACCATAAAAAAGATCATTAAAAAGGCGTGAGCCGTTATTAAAACATTATAAAGTTG : 90
CCAATTACCAGATCCACCTATCATCGCCGGCATAACCATAAAAAAGATCATTAAAAAGGCGTGAGCCGTTATTAAAACATTATAAAGTTG

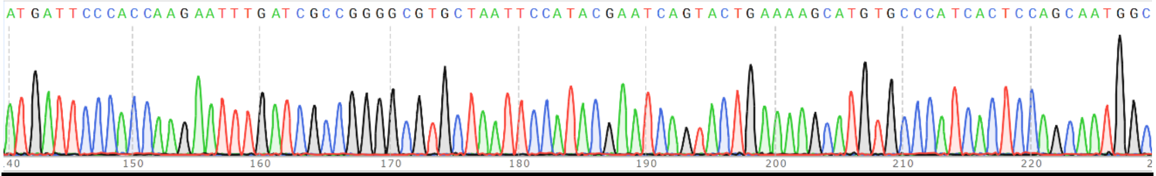

PCR_C1 : ATGATTCCCACCAAGAATTTGATCGCCGGGGCGTGCTAATTCCATACGAATCAGTACTGAAAAGCATGTGCCCATCACTCCAGCAATGGC : 180
R01_C1 : ATGATTCCCACCAAGAATTTGATCGCCGGGGCGTGCTAATTCCATACGAATCAGTACTGAAAAGCATGTGCCCATCACTCCAGCAATGGC : 180
ATGATTCCCACCAAGAATTTGATCGCCGGGGCGTGCTAATTCCATACGAATCAGTACTGAAAAGCATGTGCCCATCACTCCAGCAATGGC

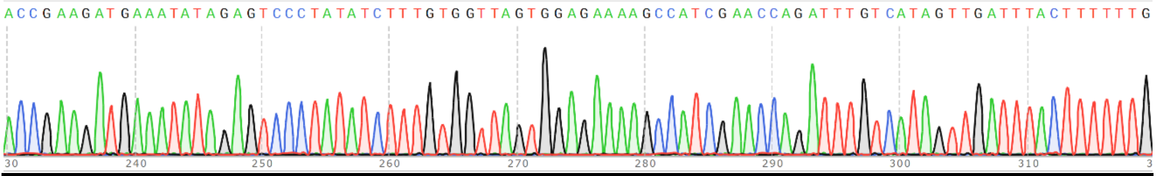

PCR_C1 : ACCGAAGATGAAATATAGAGTCCCTATATCTTTGTGGTTAGTGGAGAAAAGCCATCGAACCAGATTTGTCATAGTTGATTTACTTTTTTG : 270
R01_C1 : ACCGAAGATGAAATATAGAGTCCCTATATCTTTGTGGTTAGTGGAGAAAAGCCATCGAACCAGATTTGTCATAGTTGATTTACTTTTTTG : 270
ACCGAAGATGAAATATAGAGTCCCTATATCTTTGTGGTTAGTGGAGAAAAGCCATCGAACCAGATTTGTCATAGTTGATTTACTTTTTTG

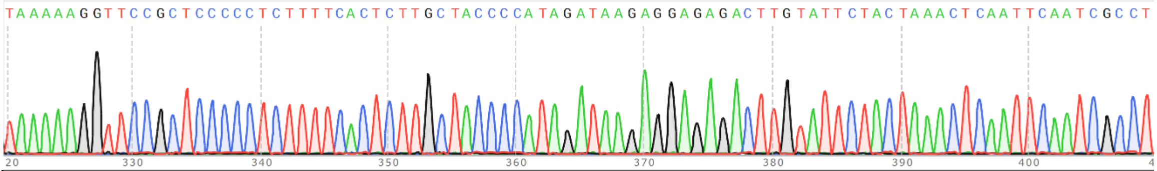

PCR_C1 : TAAAAAGGTTCCGCTCCCCCTCTTTTCACTCTTGCTACCCCATAGATAAGAGGAGAGACTTGTATTCTACTAAACTCAATTCAATCGCCT : 360
R01_C1 : TAAAAAGGTTCCGCTCCCCCTCTTTTCACTCTTGCTACCCCATAGATAAGAGGAGAGACTTGTATTCTACTAAACTCAATTCAATCGCCT : 360
TAAAAAGGTTCCGCTCCCCCTCTTTTCACTCTTGCTACCCCATAGATAAGAGGAGAGACTTGTATTCTACTAAACTCAATTCAATC**GCCT**

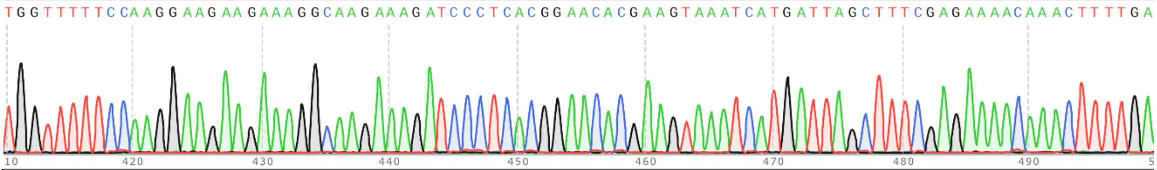

PCR_C1 : TGGTTTTTCCAAGGAAGAAGAGAAAGGCAAGAAAGATCCCTCACGGAACACGAAGTAAATCATGATTAGCTTTCGAGAAAACAAACTTTTGA : 450
R01_C1 : TGGTTTTTCCAAGGAAGAAGAGAAAGGCAAGAAAGATCCCTCACGGAACACGAAGTAAATCATGATTAGCTTTCGAGAAAACAAACTTTTGA : 450
TGGTTTTTCCAAGGAAGAAGAGAAAGGCAAGAAAGATCCCTCACGGAACACGAAGTAAATCATGATTAGCTTTCGAGAAAACAAACTTTTGA

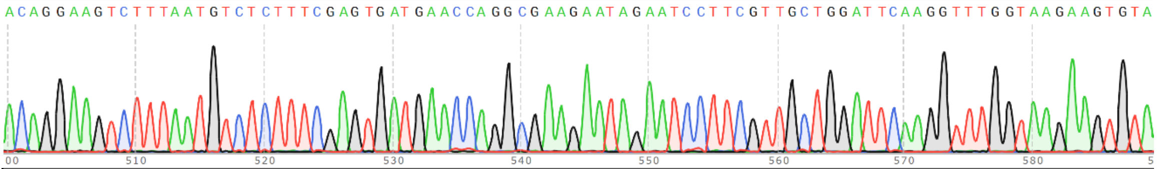

PCR_C1 : ACAGGAAGTCTTTAATGTCTCTTTCGAGTGATGAACCAGGCGAAGAATAGAATCCTTCGTTGCTGGATTCAAGGTTTGGTAAGAAGTGTA : 540
R01_C1 : ACAGGAAGTCTTTAATGTCTCTTTCGAGTGATGAACCAGGCGAAGAATAGAATCCTTCGTTGCTGGATTCAAGGTTTGGTAAGAAGTGTA : 540
ACAGGAAGTCTTTAATGTCTCTTTCGAGTGATGAACCAGGCGAAGAATAGAATCCTTCGTTGCTGGATTCAAGGTTTGGTAAGAAGTGTA

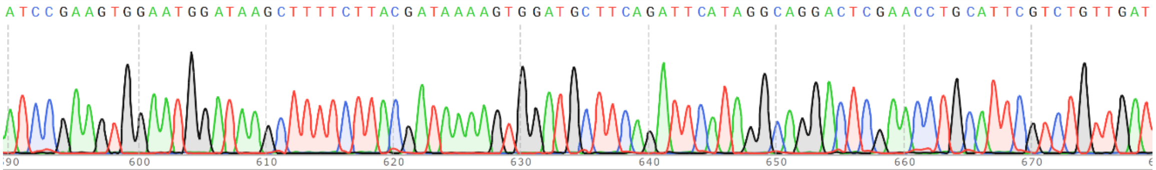

PCR_C1 : ATCCGAAGTGGAATGGATAAGCTTTTCTTACGATAAAAGTGGATGCTTCAGATTCATAGGCAGGACTCGAACCTGCATTCGTCTGTTGAT : 630

R01_C1 : ATCCGAAGTGGAATGGATAAGCTTTTCTTACGATAAAAGTGGATGCTTCAGATTCATAGGCAGGACTCGAACCTGCATTCGTCTGTTGAT : 630

ATCCGAAGTGGAATGGATAAGCTTTTCTTACGATAAAAGTGGATGCTTCAGATTCATAGGCAGGACTCGAACCTGCATTCGTCTGTTGAT

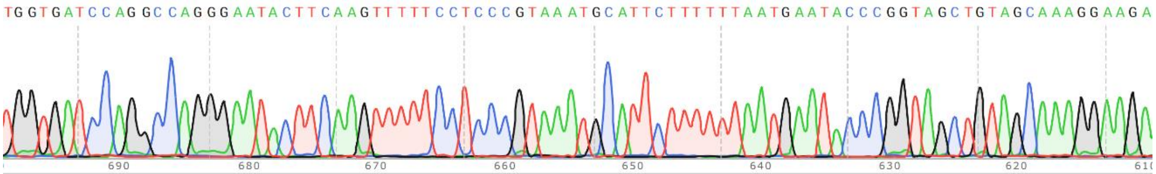

PCR_C1 : TGGTGATCCAGGCCAGGGAATACTTCAAGTTTTTCCTCCCGTAAATGCATTCTTTTTTAATGAATACCCGGTAGCTGTAGCAAAGGAAGA : 720

R01_C1 : TGGTGATCCAGGCCAGGGAATACTTCAAGTTTTTCCTCCCGTAAATGCATTCTTTTTTAATGAATACCCGGTAGCTGTAGCAAAGGAAGA : 720

TGGTGATCCAGGCCAGGGAATACTTCAAGTTTTTCCTCCCGTAAATGCATTCTTTTTTAATGAATACCCGGTAGCTGTAGCAAAGGAAGA

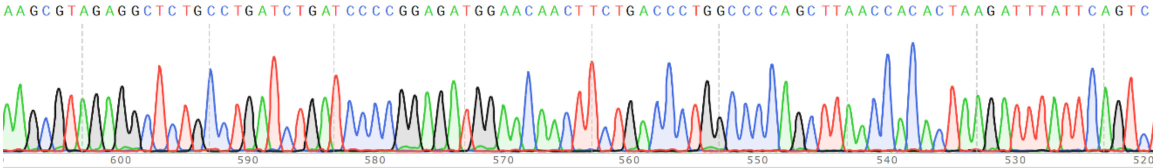

PCR_C1 : AAGCGTAGAGGCTCTGCCTGATCTGATCCCCGGAGATGGAACAACTTCTGACCCTGGCCCCAGCTTAACCACACTAAGATTTATTCAGTC : 810

R01_C1 : AAGCGTAGAGGCTCTGCCTGATCTGATCCCCGGAGATGGAACAACTTCTGACCCTGGCCCCAGCTTAACCACACTAAGATTTATTCAGTC : 810

AAGCGTAGAGGCTCTGCCTGATCTGATCCCCGGAGATGGAACAACTTCTGACCCTGGCCCCAGCTTAACCACACTAAGATTTATTCAGTC

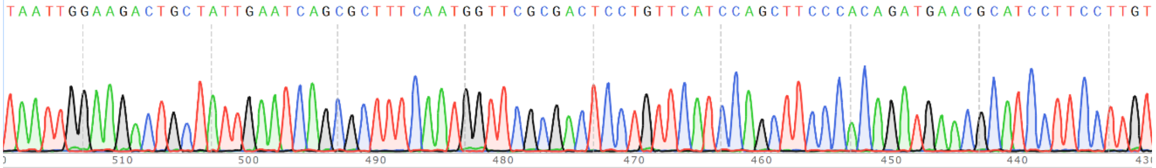

PCR_C1 : TAATTGGAAGACTGCTATTGAATCAGCGCTTTCAATGGTTCGCGACTCCTGTTCATCCAGCTTCCCACAGATGAACGCATCCTTCCTTGT : 900

R01_C1 : TAATTGGAAGACTGCTATTGAATCAGCGCTTTCAATGGTTCGCGACTCCTGTTCATCCAGCTTCCCACAGATGAACGCATCCTTCCTTGT : 900

TAATTGGAAGACTGCTATTGAATCAGCGCTTTCAATGGTTCGCGACTCCTGTTCATCCAGCTTCCCACAGATGAACGCATCCTTCCTTGT

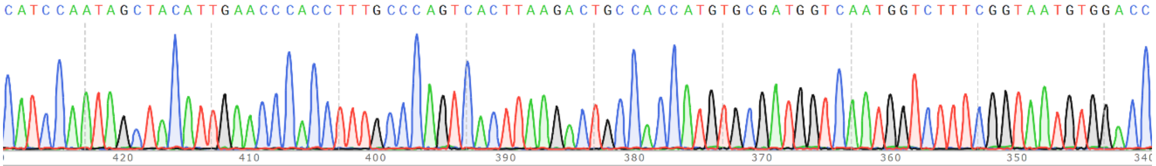

PCR_C1 : CATCCAATAGCTACATTGAACCCACCTTTGCCCAGTCACTTAAGACTGCCACCATGTGCGATGGTCAATGGTCTTTCGGTAATGTGGACC : 990

R01_C1 : CATCCAATAGCTACATTGAACCCACCTTTGCCCAGTCACTTAAGACTGCCACCATGTGCGATGGTCAATGGTCTTTCGGTAATGTGGACC : 990

CATCCAATAGCTACATTGAACCCACCTTTGCCCAGTCACTTAAGACTGCCACCATGTGCGATGGTCAATGGTCTTTCGGTAATGTGGACC

b comparison of the sequence obtained from PCR amplification and Sanger sequencing (R01-C2) and the expected sequence.

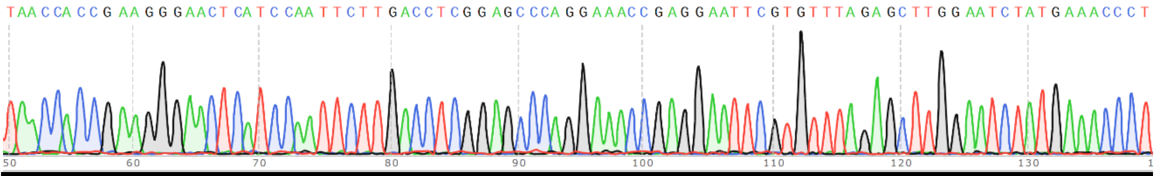

PCR_C2 : taaccaccgaagggaactcatccaattcttgacctcggagcccaggaaaccgaggaattcgtgtttagagcttggaatctatgaaaccct : 90

R01_C2 : taaccaccgaagggaactcatccaattcttgacctcggagcccaggaaaccgaggaattcgtgtttagagcttggaatctatgaaaccct : 90

TAACCACCGAAGGGAACTCATCCAATTCTTGACCTCGGAGCCCAGGAAACCGAGGAATTCGTGTTTAGAGCTTGGAATCTATGAAACCCT

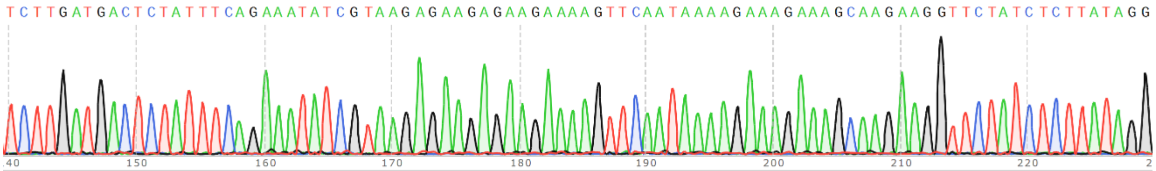

PCR_C2 : tcttgatgactctatttcagaaatatcgtaagagaagagaagaaaagttcaataaaagaaagaaagcaagaaggttctatctcttatagg : 180

R01_C2 : tcttgatgactctatttcagaaatatcgtaagagaagagaagaaaagttcaataaaagaaagaaagcaagaaggttctatctcttatagg : 180

TCTTGATGACTCTATTTCAGAAATATCGTAAGAGAAGAGAAGAAAAGTTCAATAAAAGAAAGAAAGCAAGAAGGTTCTATCTCTTATAGG

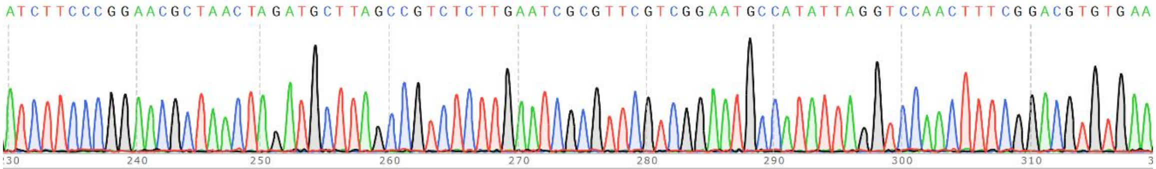

PCR_C2 : atcttcccggaacgctaactagatgcttagccgtctcttgaatcgcgttcgtcggaatgccatattaggtccaactttcggacgtgtgaa : 270

R01_C2 : atcttcccggaacgctaactagatgcttagccgtctcttgaatcgcgttcgtcggaatgccatattaggtccaactttcggacgtgtgaa : 270

ATCTTCCCGGAACGCTAACTAGATGCTTAGCCGTCTCTTGAATCGCGTTCGTCGGAATGCCATATTAGGTCCAACTTTCGGACGTGTGAA

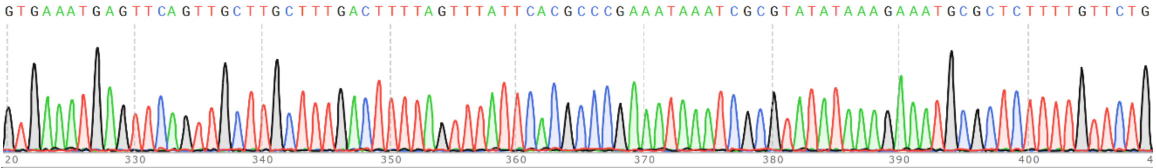

PCR_C2 : gtgaaatgagttcagttgcttgctttgacttttagtttattcacgcccgaaataaatcgcgtatataaagaaatgcgctcttttgttctg : 360

R01_C2 : gtgaaatgagttcagttgcttgctttgacttttagtttattcacgcccgaaataaatcgcgtatataaagaaatgcgctcttttgttctg : 360

GTGAAATGAGTTCAGTTGCTTGCTTTGACTTTTAGTTTATTCACGCCCGAAATAAATCGCGTATATAAAGAAATGCGCTCTTTTGTTCTG

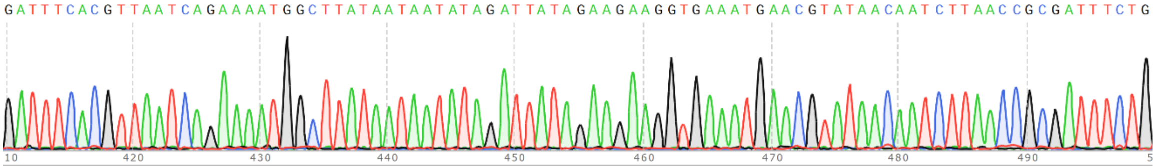

PCR_C2 : gatttcacgttaatcagaaaatggcttataataatatagattatagaagaaggtgaaatgaacgtataacaatcttaaccgcgatttctg : 450

R01_C2 : gatttcacgttaatcagaaaatggcttataataatatagattatagaagaaggtgaaatgaacgtataacaatcttaaccgcgatttctg : 450

GATTTCACGTTAATCAGAAAATGGCTTATAATAATATAGATTATAGAAGAAGGTGAAATGAACGTATAACAATCTTAACCGCGATTTCTG

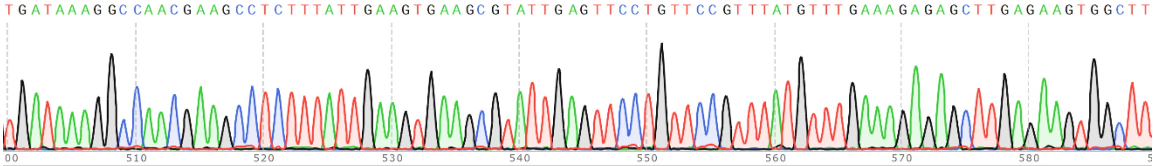

PCR_C2 : tgataaaggccaacgaagcctctttattgaagtgaagcgtattgagttcctgttccgtttatgtttgaaagagagcttgagaagtggctt : 540

R01_C2 : tgataaaggccaacgaagcctctttattgaagtgaagcgtattgagttcctgttccgtttatgtttgaaagagagcttgagaagtggctt : 540

TGATAAAGGCCAACGAAGCCTCTTTATTGAAGTGAAGCGTATTGAGTTCCTGTTCCGTTTATGTTTGAAAGAGAGCTTGAGAAGTGGCTT

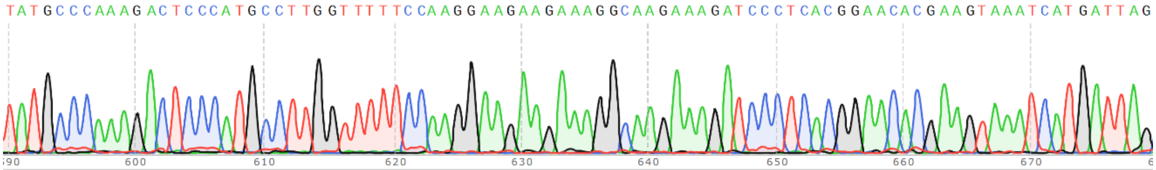

PCR_C2 : tatgcccaaagactcccatgccttggtttttccaaggaagaagaaaggcaagaaagatccctcacggaacacgaagtaaatcatgattag : 630

R01_C2 : tatgcccaaagactcccatgccttggtttttccaaggaagaagaaaggcaagaaagatccctcacggaacacgaagtaaatcatgattag : 630

TATGCCCAAAGACTCCCAT**GCCTTGGTTTTTCCAAGGAAGAAGAAAGGCAAGAAAGATCCCTCACGGAACACGAAGTAAATCATGATTAG**

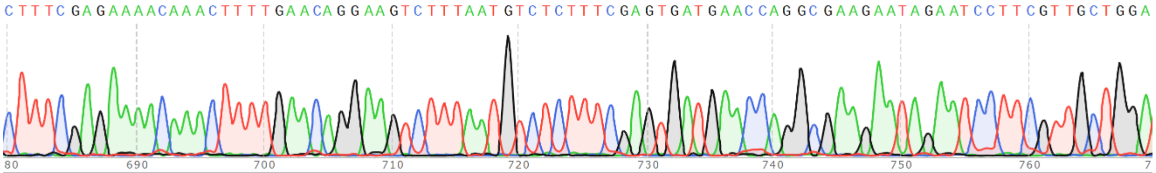

PCR_C2 : ctttcgagaaaacaaactttttgaacaggaagtctttaatgtctctttcgagtgatgaaccaggcgaagaatagaatccttcgttgctgga : 720

R01_C2 : ctttcgagaaaacaaactttttgaacaggaagtctttaatgtctctttcgagtgatgaaccaggcgaagaatagaatccttcgttgctgga : 720

CTTTCGAGAAAACAAACTTTTTGAACAGGAAGTCTTTAATGTCTCTTTCGAGTGATGAACCAGGCGAAGAATAGAATCCTTCGTTGCTGGA

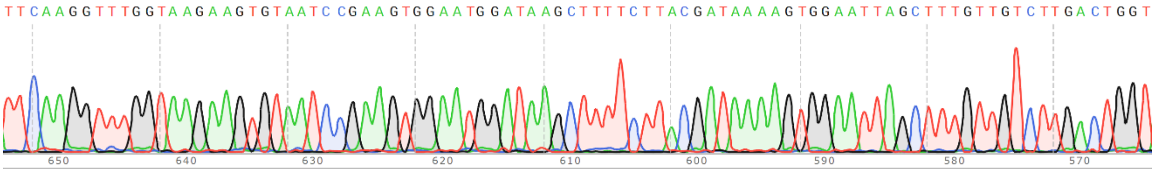

PCR_C2 : ttcaaggtttggtaagaagtgtaatccgaagtggaatggataagcttttcttacgataaaagtggaattagctttgttgtcttgactggt : 810

R01_C2 : ttcaaggtttggtaagaagtgtaatccgaagtggaatggataagcttttcttacgataaaagtggaattagctttgttgtcttgactggt : 810

TTCAAGGTTTGGTAAGAAGTGTAATCCGAAGTGGAATGGATAAGCTTTTCTTACGATAAAAGTGGAATTAGCTTTGTTGTCTTGACTGGT

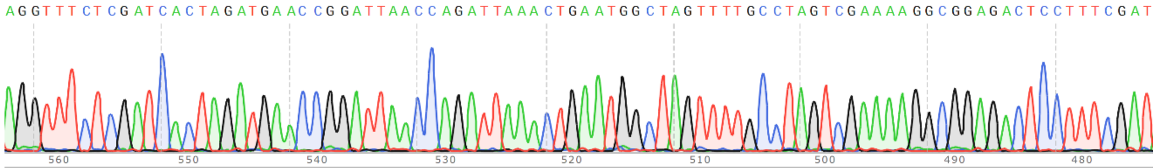

```
PCR_C2 : aggtttctcgatcactagatgaaccggattaaccagattaaactgaatggctagttttgcctagtcgaaaaggcggagactcctttcgat : 900
R01_C2 : aggtttctcgatcactagatgaaccggattaaccagattaaactgaatggctagttttgcctagtcgaaaaggcggagactcctttcgat : 900
         AGGTTTCTCGATCACTAGATGAACCGGATTAACCAGATTAAACTGAATGGCTAGTTTTGCCTAGTCGAAAAGGCGGAGACTCCTTTCGAT
```

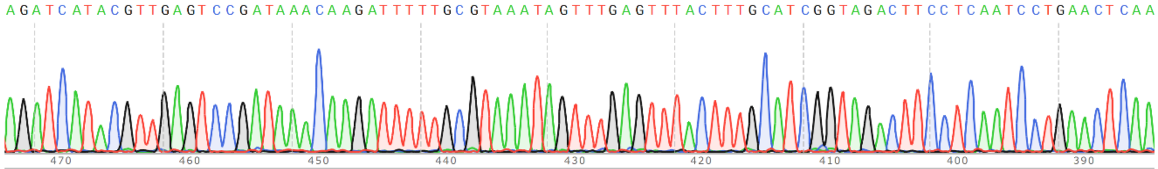

```
PCR_C2 : agatcatacgttgagtccgataaacaagattttttgcgtaaatagtttgagtttactttgcatcggtagacttcctcaatcctgaactcaa : 990
R01_C2 : agatcatacgttgagtccgataaacaagattttttgcgtaaatagtttgagtttactttgcatcggtagacttcctcaatcctgaactcaa : 990
         AGATCATACGTTGAGTCCGATAAACAAGATTTTTTGCGTAAATAGTTTGAGTTTACTTTGCATCGGTAGACTTCCTCAATCCTGAACTCAA
```

c comparison of the sequence obtained from PCR amplification and Sanger sequencing (R01-C3) and the expected sequence.

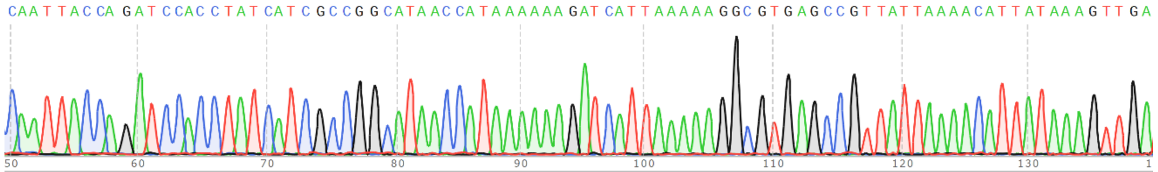

```
PCR_C3 : caattaccagatccacctatcatcgccggcataaccataaaaaagatcattaaaaaggcgtgagccgttattaaaacattataaagttga : 90
R01_C3 : caattaccagatccacctatcatcgccggcataaccataaaaaagatcattaaaaaggcgtgagccgttattaaaacattataaagttga : 90
         CAATTACCAGATCCACCTATCATCGCCGGCATAACCATAAAAAAGATCATTAAAAAGGCGTGAGCCGTTATTAAAACATTATAAAGTTGA
```

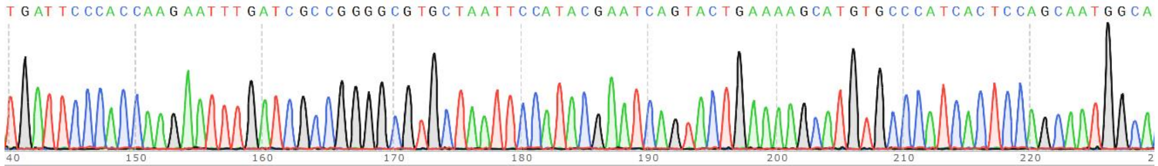

```
PCR_C3 : tgattcccaccaagaatttgatcgccggggcgtgctaattccatacgaatcagtactgaaaagcatgtgcccatcactccagcaatggca : 180
R01_C3 : tgattcccaccaagaatttgatcgccggggcgtgctaattccatacgaatcagtactgaaaagcatgtgcccatcactccagcaatggca : 180
         TGATTCCCACCAAGAATTTGATCGCCGGGGCGTGCTAATTCCATACGAATCAGTACTGAAAAGCATGTGCCCATCACTCCAGCAATGGCA
```

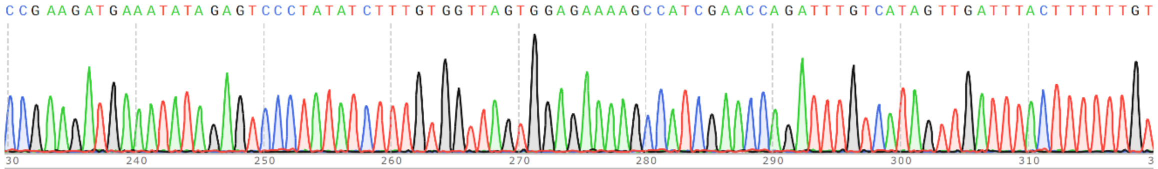

```
PCR_C3 : ccgaagatgaaatatagagtccctatatctttgtggttagtggagaaaagccatcgaaccagatttgtcatagttgatttactttttttgt : 270
R01_C3 : ccgaagatgaaatatagagtccctatatctttgtggttagtggagaaaagccatcgaaccagatttgtcatagttgatttactttttttgt : 270
         CCGAAGATGAAATATAGAGTCCCTATATCTTTGTGGTTAGTGGAGAAAAGCCATCGAACCAGATTTGTCATAGTTGATTTACTTTTTTTGT
```

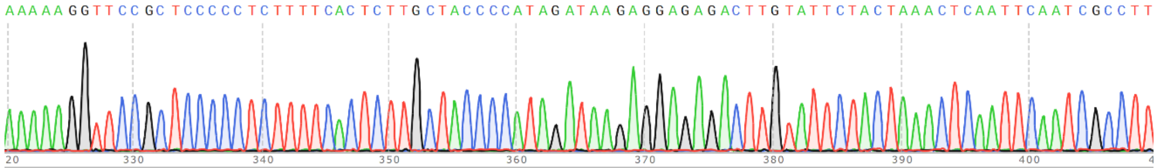

```
PCR_C3 : aaaaaggttccgctccccctcttttcactcttgctaccccatagataagaggagagacttgtattctactaaactcaattcaatcgcctt : 360
R01_C3 : aaaaaggttccgctccccctcttttcactcttgctaccccatagataagaggagagacttgtattctactaaactcaattcaatcgcctt : 360
         AAAAAGGTTCCGCTCCCCCTCTTTTCACTCTTGCTACCCCATAGATAAGAGGAGAGACTTGTATTCTACTAAACTCAATTCAATCGCCTT
```

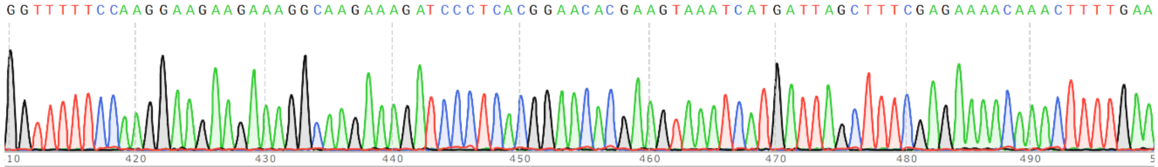

```
PCR_C3 : ggtttttccaaggaagaagaaaggcaagaaagatccctcacggaacacgaagtaaatcatgattagctttcgagaaaacaaactttttgaa : 450
R01_C3 : ggtttttccaaggaagaagaaaggcaagaaagatccctcacggaacacgaagtaaatcatgattagctttcgagaaaacaaactttttgaa : 450
```

```
GGTTTTTCCAAGGAAGAAGAAAGGCAAGAAAGATCCCTCACGGAACACGAAGTAAATCATGATTAGCTTTCGAGAAAACAAACTTTTGAA
```

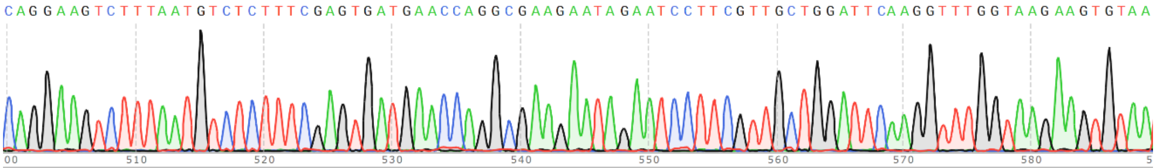

```
PCR_C3 : caggaagtctttaatgtctctttcgagtgatgaaccaggcgaagaatagaatccttcgttgctggattcaaggtttggtaagaagtgtaa : 540
R01_C3 : caggaagtctttaatgtctctttcgagtgatgaaccaggcgaagaatagaatccttcgttgctggattcaaggtttggtaagaagtgtaa : 540
         CAGGAAGTCTTTAATGTCTCTTTCGAGTGATGAACCAGGCGAAGAATAGAATCCTTCGTTGCTGGATTCAAGGTTTGGTAAGAAGTGTAA
```

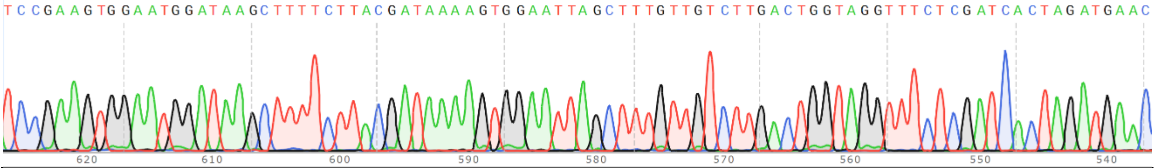

```
PCR_C3 : tccgaagtggaatggataagcttttcttacgataaaagtggaattagctttgttgtcttgactggtaggtttctcgatcactagatgaac : 630
R01_C3 : tccgaagtggaatggataagcttttcttacgataaaagtggaattagctttgttgtcttgactggtaggtttctcgatcactagatgaac : 630
         TCCGAAGTGGAATGGATAAGCTTTTCTTACGATAAAAGTGGAATTAGCTTTGTTGTCTTGACTGGTAGGTTTCTCGATCACTAGATGAAC
```

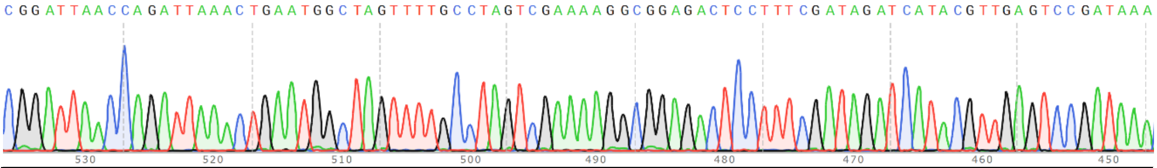

```
PCR_C3 : cggattaaccagattaaactgaatggctagttttgcctagtcgaaaaggcggagactcctttcgatagatcatacgttgagtccgataaa : 720
R01_C3 : cggattaaccagattaaactgaatggctagttttgcctagtcgaaaaggcggagactcctttcgatagatcatacgttgagtccgataaa : 720
         CGGATTAACCAGATTAAACTGAATGGCTAGTTTTGCCTAGTCGAAAAGGCGGAGACTCCTTTCGATAGATCATACGTTGAGTCCGATAAA
```

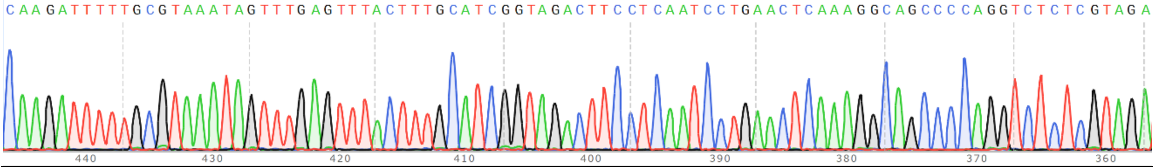

```
PCR_C3 : caagattttttgcgtaaatagtttgagtttactttgcatcggtagacttcctcaatcctgaactcaaaggcagcccccaggtctctcgtaga : 810
R01_C3 : caagattttttgcgtaaatagtttgagtttactttgcatcggtagacttcctcaatcctgaactcaaaggcagcccccaggtctctcgtaga : 810
         CAAGATTTTTGCGTAAATAGTTTGAGTTTACTTTGCATCGGTAGACTTCCTCAATCCTGAACTCAAAGGCAGCCCCAGGTCTCTCGTAGA
```

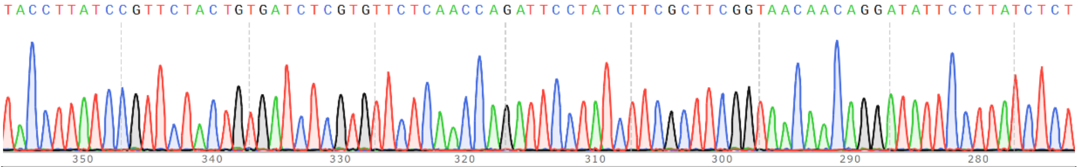

```
PCR_C3 : taccttatccgttctactgtgatctcgtgttctcaaccagattcctatcttcgcttcggtaacaacaggatattccttatctct : 894
R01_C3 : taccttatccgttctactgtgatctcgtgttctcaaccagattcctatcttcgcttcggtaacaacaggatattccttatctct : 894
         TACCTTATCCGTTCTACTGTGATCTCGTGTTCTCAACCAGATTCCTATCTTCGCTTCGGTAACAACAGGATATTCCTTATCTCT
```

d comparison of the sequence obtained from PCR amplification and Sanger sequencing (R01-C4) and the expected sequence.

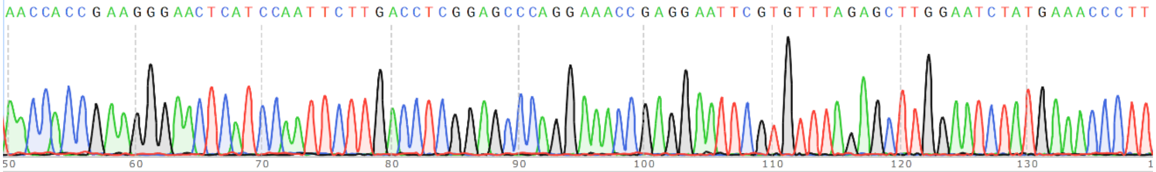

```
PCR_C4 : aaccaccgaagggaactcatccaattcttgacctcggagcccaggaaaccgaggaattcgtgtttagagcttggaatctatgaaaccctt : 90
R01_C4 : aaccaccgaagggaactcatccaattcttgacctcggagcccaggaaaccgaggaattcgtgtttagagcttggaatctatgaaaccctt : 90
         AACCACCGAAGGGAACTCATCCAATTCTTGACCTCGGAGCCCAGGAAACCGAGGAATTCGTGTTTAGAGCTTGGAATCTATGAAACCCTT
```

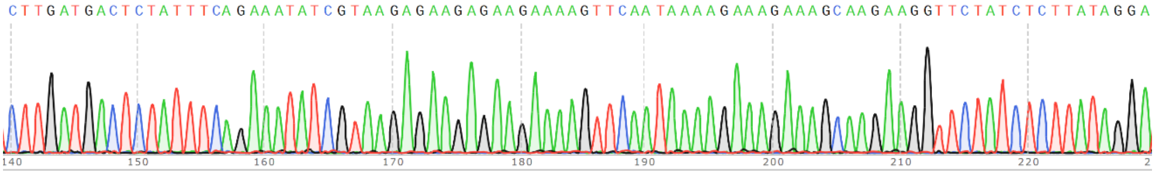

PCR_C4 : cttgatgactctatttcagaaatatcgtaagagaagagaagaaaagttcaataaaagaaagaaagcaagaaggttctatctcttatagga : 180
R01_C4 : cttgatgactctatttcagaaatatcgtaagagaagagaagaaaagttcaataaaagaaagaaagcaagaaggttctatctcttatagga : 180
CTTGATGACTCTATTTCAGAAATATCGTAAGAGAAGAGAAGAAAAGTTCAATAAAAGAAAGAAAGCAAGAAGGTTCTATCTCTTATAGGA

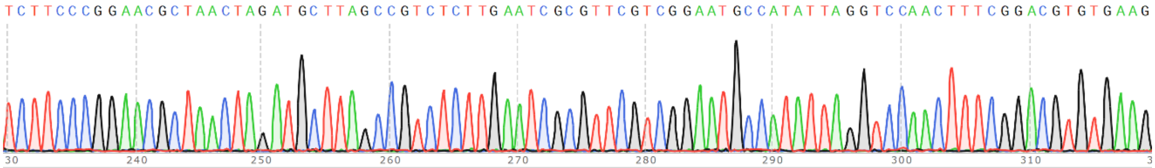

PCR_C4 : tcttcccggaacgctaactagatgcttagccgtctcttgaatcgcgttcgtcggaatgccatattaggtccaactttcggacgtgtgaag : 270
R01_C4 : tcttcccggaacgctaactagatgcttagccgtctcttgaatcgcgttcgtcggaatgccatattaggtccaactttcggacgtgtgaag : 270
TCTTCCCGGAACGCTAACTAGATGCTTAGCCGTCTCTTGAATCGCGTTCGTCGGAATGCCATATTAGGTCCAACTTTCGGACGTGTGAAG

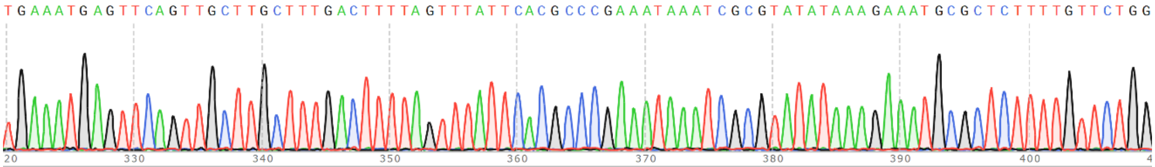

PCR_C4 : tgaaatgagttcagttgcttgctttgacttttagtttattcacgcccgaaataaatcgcgtatataaagaaatgcgctcttttgttctgg : 360
R01_C4 : tgaaatgagttcagttgcttgctttgacttttagtttattcacgcccgaaataaatcgcgtatataaagaaatgcgctcttttgttctgg : 360
TGAAATGAGTTCAGTTGCTTGCTTTGACTTTTAGTTTATTCACGCCCGAAATAAATCGCGTATATAAAGAAATGCGCTCTTTTGTTCTGG

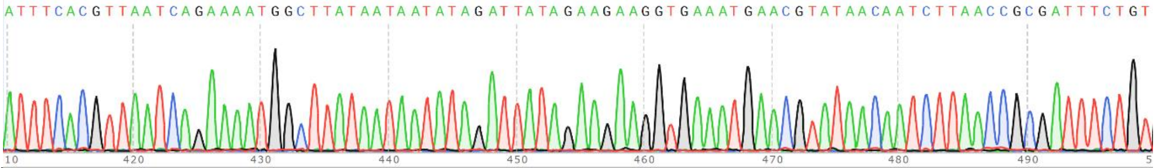

PCR_C4 : atttcacgttaatcagaaaatggcttataataatatagattatagaagaaggtgaaatgaacgtataacaatcttaaccgcgatttctgt : 450
R01_C4 : atttcacgttaatcagaaaatggcttataataatatagattatagaagaaggtgaaatgaacgtataacaatcttaaccgcgatttctgt : 450
ATTTCACGTTAATCAGAAAATGGCTTATAATAATATAGATTATAGAAGAAGGTGAAATGAACGTATAACAATCTTAACCGCGATTTCTGT

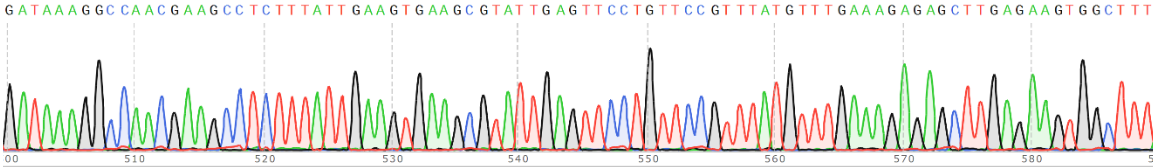

PCR_C4 : gataaaggccaacgaagcctctttattgaagtgaagcgtattgagttcctgttccgtttatgtttgaaagagagcttgagaagtggcttt : 540
R01_C4 : gataaaggccaacgaagcctctttattgaagtgaagcgtattgagttcctgttccgtttatgtttgaaagagagcttgagaagtggcttt : 540
GATAAAGGCCAACGAAGCCTCTTTATTGAAGTGAAGCGTATTGAGTTCCTGTTCCGTTTATGTTTGAAAGAGAGCTTGAGAAGTGGCTTT

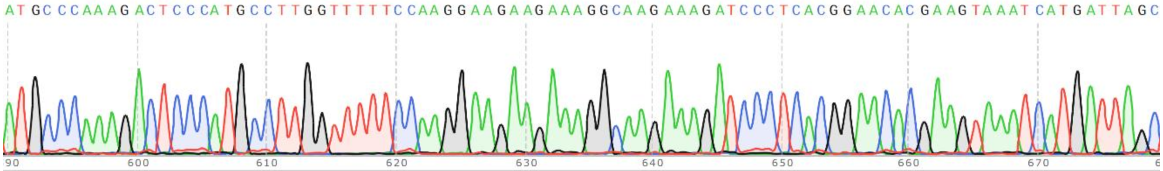

PCR_C4 : atgcccaaagactcccatgccttggtttttccaaggaagaagaaaggcaagaaagatccctcacggaacacgaagtaaatcatgattagc : 630
R01_C4 : atgcccaaagactcccatgccttggtttttccaaggaagaagaaaggcaagaaagatccctcacggaacacgaagtaaatcatgattagc : 630
ATGCCCAAAGACTCCCAT**GCCTTGGTTTTTCCAAGGAAGAAGAAAGGCAAGAAAGATCCCTCACGGAACACGAAGTAAATCATGATTAGC**

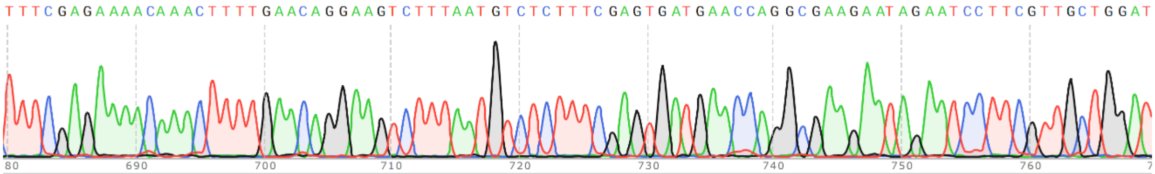

PCR_C4 : tttcgagaaaacaaacttttgaacaggaagtctttaatgtctctttcgagtgatgaaccaggcgaagaatagaatccttcgttgctggat : 720
R01_C4 : tttcgagaaaacaaacttttgaacaggaagtctttaatgtctctttcgagtgatgaaccaggcgaagaatagaatccttcgttgctggat : 720
TTTCGAGAAAACAAACTTTTGAACAGGAAGTCTTTAATGTCTCTTTCGAGTGATGAACCAGGCGAAGAATAGAATCCTTCGTTGCTGGAT

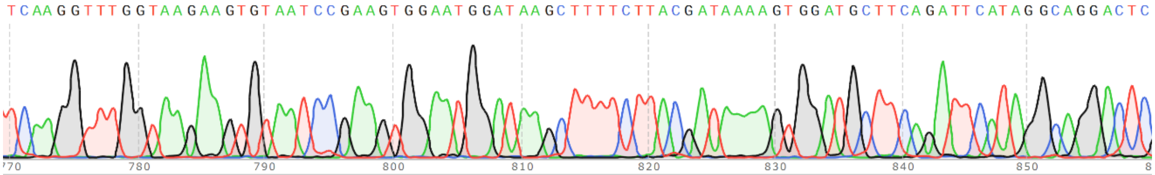

PCR_C4 : tcaaggtttggtaagaagtgtaatccgaagtggaatggataagcttttcttacgataaaagtggatgcttcagattcataggcaggactc : 810
R01_C4 : tcaaggtttggtaagaagtgtaatccgaagtggaatggataagcttttcttacgataaaagtggatgcttcagattcataggcaggactc : 810
TCAAGGTTTGGTAAGAAGTGTAATCCGAAGTGGAATGGATAAGCTTTTCTTACGATAAAAGTGGATGCTTCAGATTCATAGGCAGGACTC

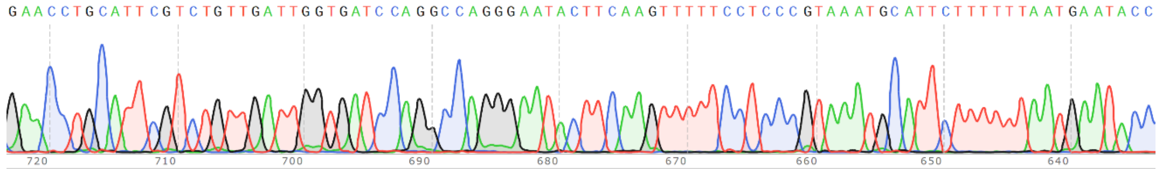

```
PCR_C4 : gaacctgcattcgtctgttgattggtgatccaggccagggaatacttcaagtttttcctcccgtaaatgcattctttttttaatgaatacc : 900
R01_C4 : gaacctgcattcgtctgttgattggtgatccaggccagggaatacttcaagtttttcctcccgtaaatgcattctttttttaatgaatacc : 900
         GAACCTGCATTCGTCTGTTGATTGGTGATCCAGGCCAGGGAATACTTCAAGTTTTTCCTCCCGTAAATGCATTCTTTTTTTAATGAATACC
```

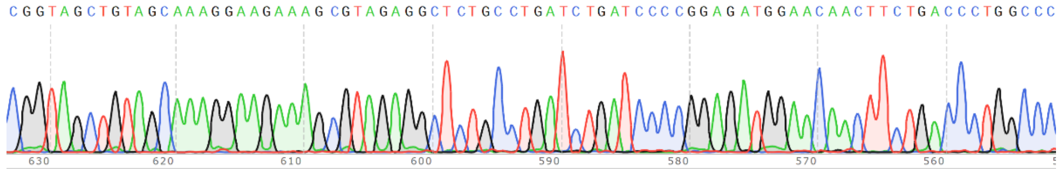

```
PCR_C4 : cggtagctgtagcaaaggaagaaagcgtagaggctctgcctgatctgatccccggagatggaacaacttctgaccctggccc : 982
R01_C4 : cggtagctgtagcaaaggaagaaagcgtagaggctctgcctgatctgatccccggagatggaacaacttctgaccctggccc : 982
         CGGTAGCTGTAGCAAAGGAAGAAAGCGTAGAGGCTCTGCCTGATCTGATCCCCGGAGATGGAACAACTTCTGACCCTGGCCC
```

e comparison of the sequence obtained from PCR amplification and Sanger sequencing (R02-C1) and the expected sequence.

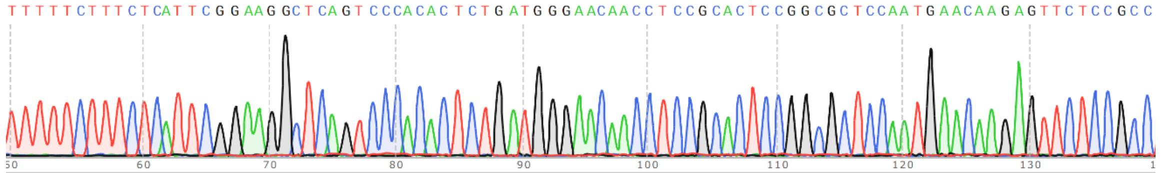

```
PCR_C1 : tttttctttctcattcggaaggctcagtcccacactctgatgggaacaacctccgcactccggcgctccaatgaacaagagttctccgcc :  90
R02_C1 : tttttctttctcattcggaaggctcagtcccacactctgatgggaacaacctccgcactccggcgctccaatgaacaagagttctccgcc :  90
         TTTTTCTTTCTCATTCGGAAGGCTCAGTCCCACACTCTGATGGGAACAACCTCCGCACTCCGGCGCTCCAATGAACAAGAGTTCTCCGCC
```

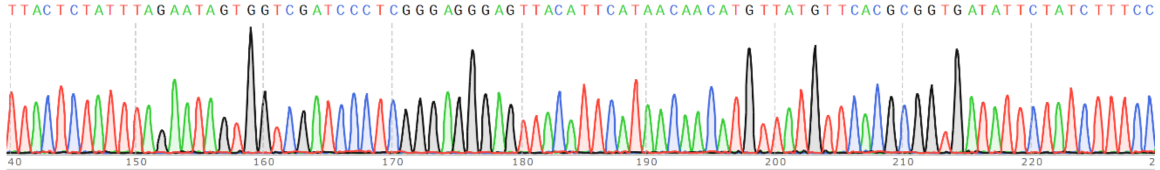

```
PCR_C1 : ttactctatttagaatagtggtcgatccctcgggagggagttacattcataacaacatgttatgttcacgcggtgatattctatctttcc : 180
R02_C1 : ttactctatttagaatagtggtcgatccctcgggagggagttacattcataacaacatgttatgttcacgcggtgatattctatctttcc : 180
         TTACTCTATTTAGAATAGTGGTCGATCCCTCGGGAGGGAGTTACATTCATAACAACATGTTATGTTCACGCGGTGATATTCTATCTTTCC
```

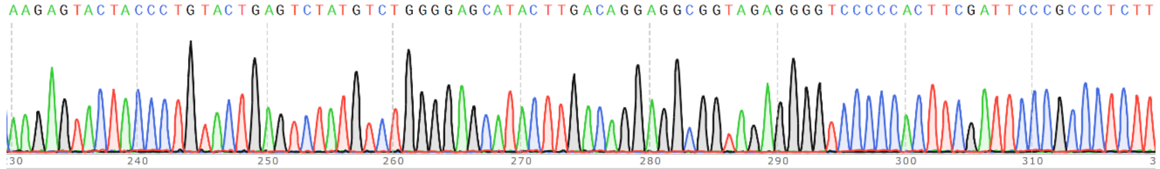

```
PCR_C1 : aagagtactaccctgtactgagtctatgtctggggagcatacttgacaggaggcggtagaggggtcccccacttcgattcccgccctctt : 270
R02_C1 : aagagtactaccctgtactgagtctatgtctggggagcatacttgacaggaggcggtagaggggtcccccacttcgattcccgccctctt : 270
         AAGAGTACTACCCTGTACTGAGTCTATGTCTGGGGAGCATACTTGACAGGAGGCGGTAGAGGGGTCCCCCACTTCGATTCCCGCCCTCTT
```

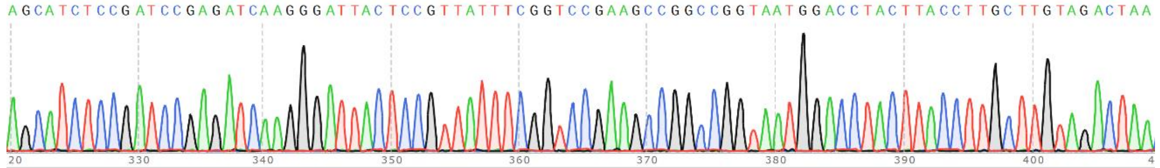

```
PCR_C1 : agcatctccgatccgagatcaagggattactccgttatttcggtccgaagccggccggtaatggacctacttaccttgcttgtagactaa : 360
R02_C1 : agcatctccgatccgagatcaagggattactccgttatttcggtccgaagccggccggtaatggacctacttaccttgcttgtagactaa : 360
         AGCATCTCCGATCCGAGATCAAGGGATTACTCCGTTATTTCGGTCCGAAGCCGGCCGGTAATGGACCTACTTACCTTGCTTGTAGACTAA
```

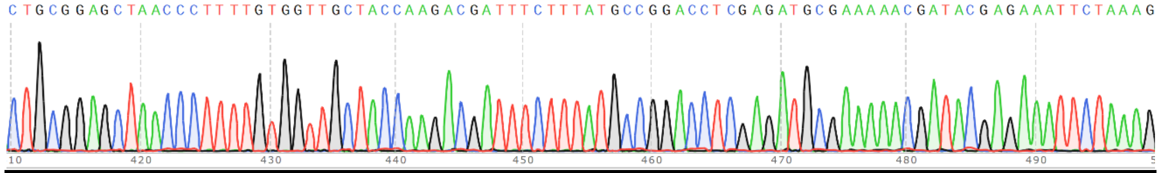

```
PCR_C1 : ctgcggagctaaccccttttgtggttgctaccaagacgatttctttatgccggacctcgagatgcgaaaaacgatacgagaaattctaaag : 450
```

R02_C1 : ctgcggagctaacccttttgtggttgctaccaagacgatttctttatgccggacctcgagatgcgaaaaacgatacgagaaattctaaag : 450

CTGCGGAGCTAACCCTTTTGTGGTTGCTACCAAGACGATTTCTTTATGCCGGACCTCGAGATGCGAAAAACGATACGAGAAATTCTAAAG

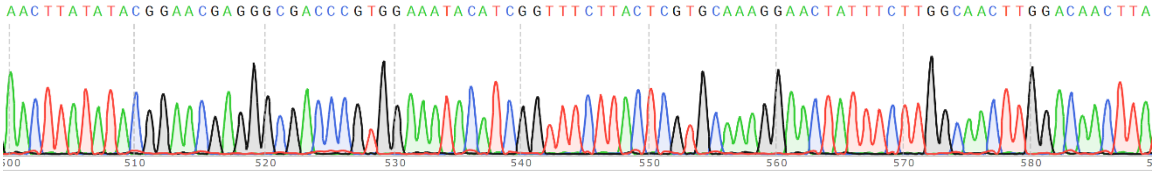

PCR_C1 : aacttatatacggaacgagggcgacccgtggaaatacatcggtttcttactcgtgcaaaggaactatttcttggcaacttggacaactta : 540

R02_C1 : aacttatatacggaacgagggcgacccgtggaaatacatcggtttcttactcgtgcaaaggaactatttcttggcaacttggacaactta : 540

AACTTATATACGGAACGAGGGCGACCCGTGGAAATACATCGGTTTCTTACTCGTGCAAAGGAACTATTTCTTGGCAACTTGGACAACTTA

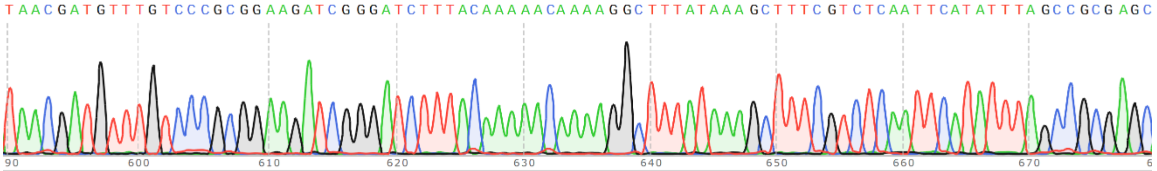

PCR_C1 : taacgatgtttgtcccgcggaagatcgggatctttacaaaaacaaaaggctttataaagctttcgtctcaattcatatttagccgcgagc : 630

R02_C1 : taacgatgtttgtcccgcggaagatcgggatctttacaaaaacaaaaggctttataaagctttcgtctcaattcatatttagccgcgagc : 630

TAACGATGTTTGTCCCGCGGAAGATCGGGATCTTTACAAAAACAAAAGGCTTTATAAAGCTTTCGTCTCAATTCATATTTAGCCGCGAGC

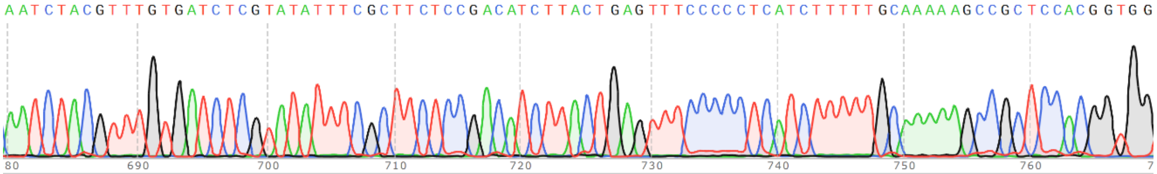

PCR_C1 : aatctacgtttgtgatctcgtatatttcgcttctccgacatcttactgagtttccccctcatctttttgcaaaaagccgctccacggtgg : 720

R02_C1 : aatctacgtttgtgatctcgtatatttcgcttctccgacatcttactgagtttccccctcatctttttgcaaaaagccgctccacggtgg : 720

AATCTACGTTTGTGATCTCGTATATTTCGCTTCTCCGACATCTTACTGAGTTTCCCCCTC**ATCTTTTTGCAAAAAGCCGCTCCACGGTGG**

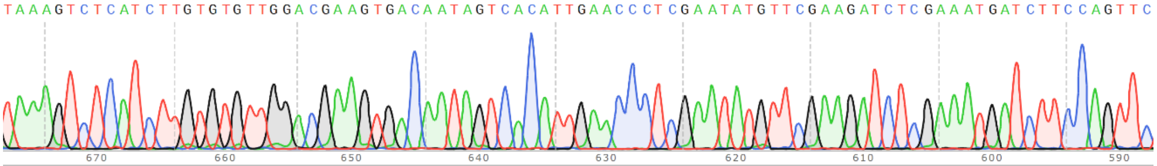

PCR_C1 : taaagtctcatcttgtgtgttggacgaagtgacaatagtcacattgaaccctcgaatatgttcgaagatctcgaaatgatcttccagttc : 810

R02_C1 : taaagtctcatcttgtgtgttggacgaagtgacaatagtcacattgaaccctcgaatatgttcgaagatctcgaaatgatcttccagttc : 810

TAAAGTCTCATCTTGTGTGTTGGACGAAGTGACAATAGTCACATTGAACCCTCGAATATGTTCGAAGATCTCGAAATGATCTTCCAGTTC

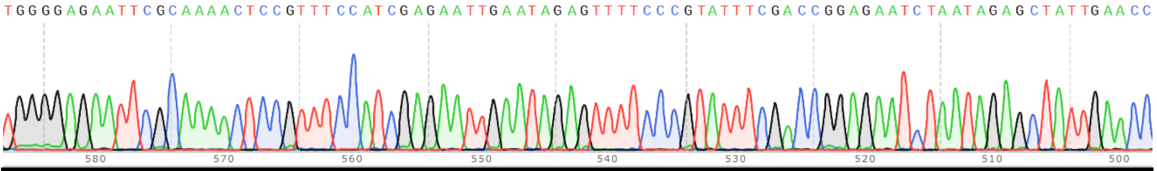

PCR_C1 : tggggagaattcgcaaaactccgtttccatcgagaattgaatagagttttcccgtatttcgaccggagaatctaatagagctattgaacc : 900

R02_C1 : tggggagaattcgcaaaactccgtttccatcgagaattgaatagagttttcccgtatttcgaccggagaatctaatagagctattgaacc : 900

TGGGGAGAATTCGCAAAACTCCGTTTCCATCGAGAATTGAATAGAGTTTTCCCGTATTTCGACCGGAGAATCTAATAGAGCTATTGAACC

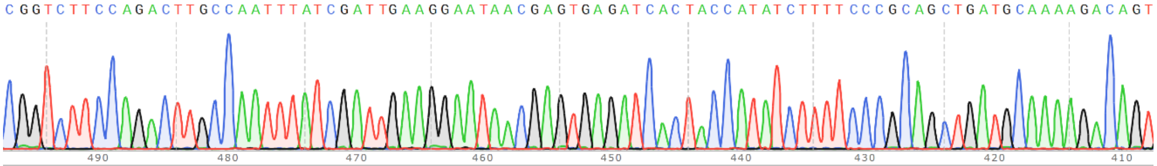

PCR_C1 : cggtcttccagacttgccaatttatcgattgaaggaataacgagtgagatcactaccatatcttttcccgcagctgatgcaaaagacagt : 990

R02_C1 : cggtcttccagacttgccaatttatcgattgaaggaataacgagtgagatcactaccatatcttttcccgcagctgatgcaaaagacagt : 990

CGGTCTTCCAGACTTGCCAATTTATCGATTGAAGGAATAACGAGTGAGATCACTACCATATCTTTTCCCGCAGCTGATGCAAAAGACAGT

f comparison of the sequence obtained from PCR amplification and Sanger sequencing (R02-C2) and the expected sequence.

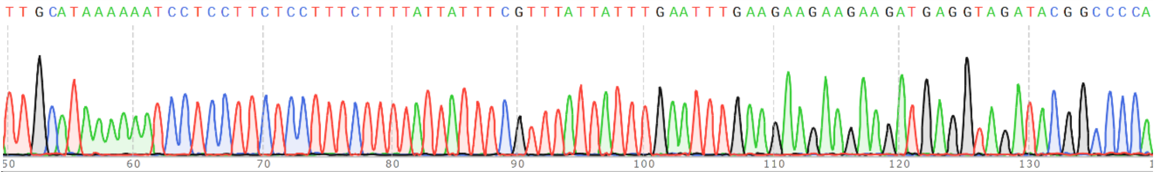

PCR_C2 : ttgcataaaaaatcctccttctcctttcttttattatttcgtttattatttgaatttgaagaagaagaagatgaggtagatacggcccca : 90
R02_C2 : ttgcataaaaaatcctccttctcctttcttttattatttcgtttattatttgaatttgaagaagaagaagatgaggtagatacggcccca : 90
TTGCATAAAAAATCCTCCTTCTCCTTTCTTTTATTATTTCGTTTATTATTTGAATTTGAAGAAGAAGAAGATGAGGTAGATACGGCCCCA

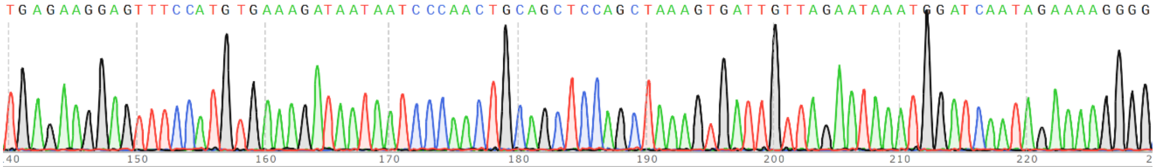

PCR_C2 : tgagaaggagtttccatgtgaaagataataatcccaactgcagctccagctaaagtgattgttagaataaatggatcaatagaaaagggg : 180
R02_C2 : tgagaaggagtttccatgtgaaagataataatcccaactgcagctccagctaaagtgattgttagaataaatggatcaatagaaaagggg : 180
TGAGAAGGAGTTTCCATGTGAAAGATAATAATCCCAACTGCAGCTCCAGCTAAAGTGATTGTTAGAATAAATGGATCAATAGAAAAGGGG

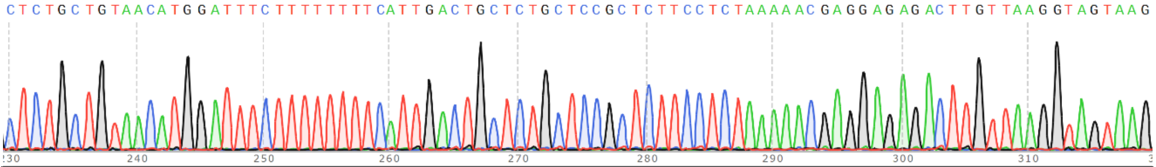

PCR_C2 : ctctgctgtaacatggatttcttttttttcattgactgctctgctccgctcttcctctaaaaacgaggagagacttgttaaggtagtaag : 270
R02_C2 : ctctgctgtaacatggatttcttttttttcattgactgctctgctccgctcttcctctaaaaacgaggagagacttgttaaggtagtaag : 270
CTCTGCTGTAACATGGATTTCTTTTTTTTCATTGACTGCTCTGCTCCGCTCTTCCTCTAAAAACGAGGAGAGACTTGTTAAGGTAGTAAG

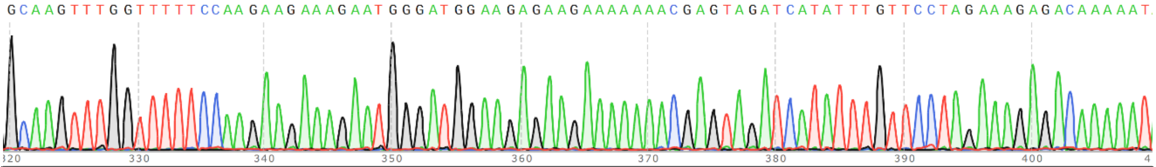

PCR_C2 : gcaagtttggtttttccaagaagaaagaatgggatggaagagaagaaaaaaacgagtagatcatatttgttcctagaaagagacaaaaat : 360
R02_C2 : gcaagtttggtttttccaagaagaaagaatgggatggaagagaagaaaaaaacgagtagatcatatttgttcctagaaagagacaaaaat : 360
GCAAGTTTGGTTTTTCCAAGAAGAAAGAATGGGATGGAAGAGAAGAAAAAAACGAGTAGATCATATTTGTTCCTAGAAAGAGACAAAAAT

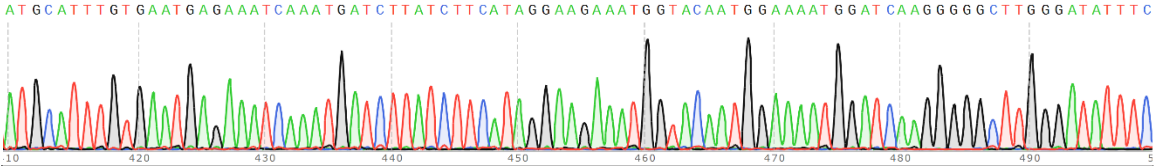

PCR_C2 : atgcatttgtgaatgagaaatcaaatgatcttatcttcataggaagaaatggtacaatggaaaatggatcaagggggcttgggatatttc : 450
R02_C2 : atgcatttgtgaatgagaaatcaaatgatcttatcttcataggaagaaatggtacaatggaaaatggatcaagggggcttgggatatttc : 450
ATGCATTTGTGAATGAGAAATCAAATGATCTTATCTTCATAGGAAGAAATGGTACAATGGAAAATGGATCAAGGGGGCTTGGGATATTTC

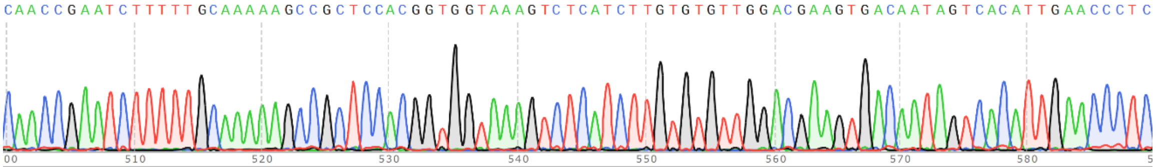

PCR_C2 : caaccgaatctttttgcaaaaagccgctccacggtggtaaagtctcatcttgtgtgttggacgaagtgacaatagtcacattgaaccctc : 540
R02_C2 : caaccgaatctttttgcaaaaagccgctccacggtggtaaagtctcatcttgtgtgttggacgaagtgacaatagtcacattgaaccctc : 540
CAACCGA**ATCTTTTTGCAAAAAGCCGCTCCACGGTGGTAAAGTCTCATCTTGTGTGTTGGACGAAGTGACAATAGTCACATTGAACCCTC**

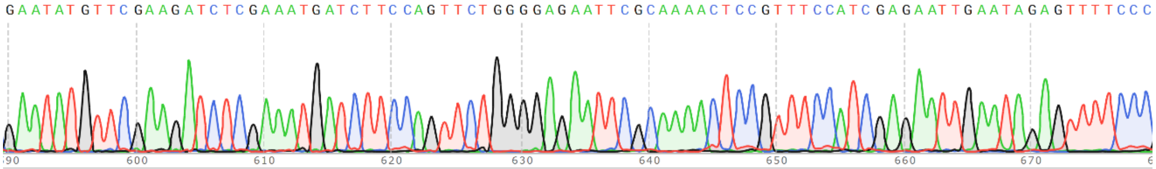

PCR_C2 : gaatatgttcgaagatctcgaaatgatcttccagttctggggagaattcgcaaaactccgtttccatcgagaattgaatagagttttccc : 630
R02_C2 : gaatatgttcgaagatctcgaaatgatcttccagttctggggagaattcgcaaaactccgtttccatcgagaattgaatagagttttccc : 630
GAATATGTTCGAAGATCTCGAAATGATCTTCCAGTTCTGGGGAGAATTCGCAAAACTCCGTTTCCATCGAGAATTGAATAGAGTTTTCCC

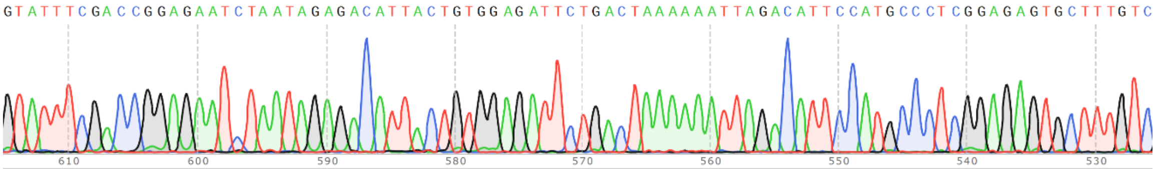

PCR_C2 : gtatttcgaccggagaatctaatagagacattactgtggagattctgactaaaaaattagacattccatgccctcggagagtgctttgtc : 720
R02_C2 : gtatttcgaccggagaatctaatagagacattactgtggagattctgactaaaaaattagacattccatgccctcggagagtgctttgtc : 720
GTATTTCGACCGGAGAATCTAATAGAGACATTACTGTGGAGATTCTGACTAAAAAATTAGACATTCCATGCCCTCGGAGAGTGCTTTGTC

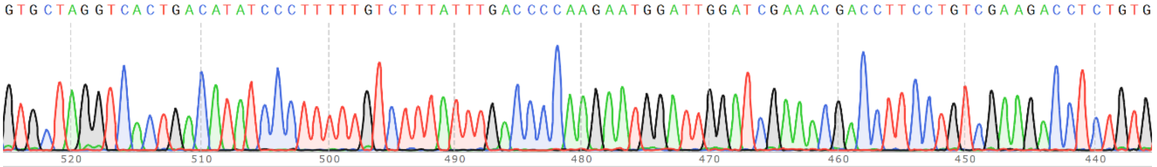

PCR_C2 : gtgctaggtcactgacatatccctttttgtctttatttgaccccaagaatggattggatcgaaacgaccttcctgtcgaagacctctgtg : 810
R02_C2 : gtgctaggtcactgacatatccctttttgtctttatttgaccccaagaatggattggatcgaaacgaccttcctgtcgaagacctctgtg : 810
GTGCTAGGTCACTGACATATCCCTTTTTGTCTTTATTTGACCCCAAGAATGGATTGGATCGAAACGACCTTCCTGTCGAAGACCTCTGTG

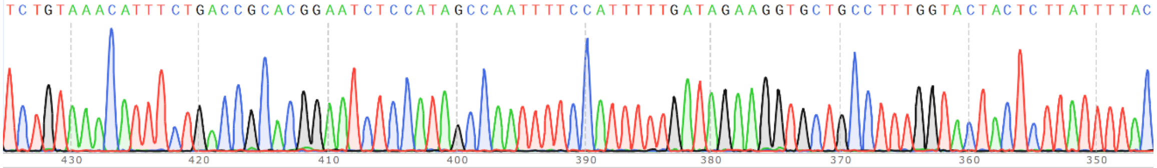

PCR_C2 : tctgtaaacatttctgaccgcacggaatctccatagccaattttccatttttgatagaaggtgctgcctttggtactactcttattttac : 900
R02_C2 : tctgtaaacatttctgaccgcacggaatctccatagccaattttccatttttgatagaaggtgctgcctttggtactactcttattttac : 900
TCTGTAAACATTTCTGACCGCACGGAATCTCCATAGCCAATTTTCCATTTTTGATAGAAGGTGCTGCCTTTGGTACTACTCTTATTTTAC

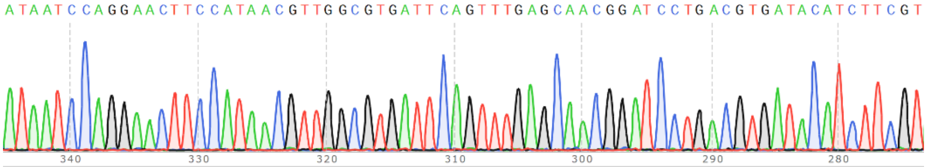

PCR_C2 : ataatccaggaacttccataacgttggcgtgattcagtttgagcaacggatcctgacgtgatacatcttcgt : 972
R02_C2 : ataatccaggaacttccataacgttggcgtgattcagtttgagcaacggatcctgacgtgatacatcttcgt : 972
ATAATCCAGGAACTTCCATAACGTTGGCGTGATTCAGTTTGAGCAACGGATCCTGACGTGATACATCTTCGT

g comparison of the sequence obtained from PCR amplification and Sanger sequencing (R02-C3) and the expected sequence.

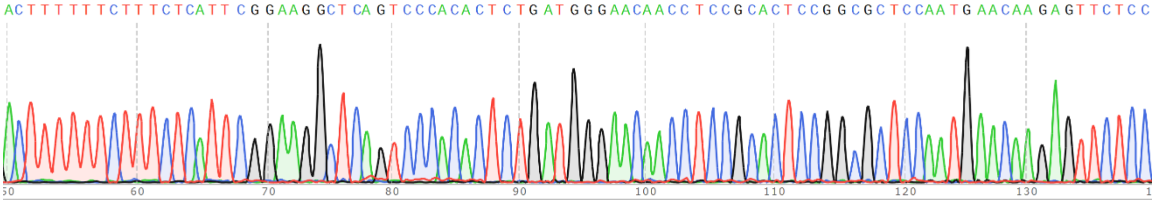

PCR_C3 : acttttttctttctcattcggaaggctcagtcccacactctgatgggaacaacctccgcactccggcgctccaatgaacaagagttctcc : 90
R02_C3 : acttttttctttctcattcggaaggctcagtcccacactctgatgggaacaacctccgcactccggcgctccaatgaacaagagttctcc : 90
ACTTTTTTCTTTCTCATTCGGAAGGCTCAGTCCCACACTCTGATGGGAACAACCTCCGCACTCCGGCGCTCCAATGAACAAGAGTTCTCC

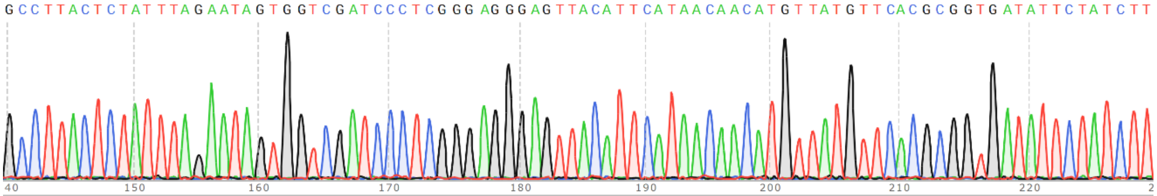

PCR_C3 : gccttactctatttagaatagtggtcgatccctcgggagggagttacattcataacaacatgttatgttcacgcggtgatattctatctt : 180
R02_C3 : gccttactctatttagaatagtggtcgatccctcgggagggagttacattcataacaacatgttatgttcacgcggtgatattctatctt : 180
GCCTTACTCTATTTAGAATAGTGGTCGATCCCTCGGGAGGGAGTTACATTCATAACAACATGTTATGTTCACGCGGTGATATTCTATCTT

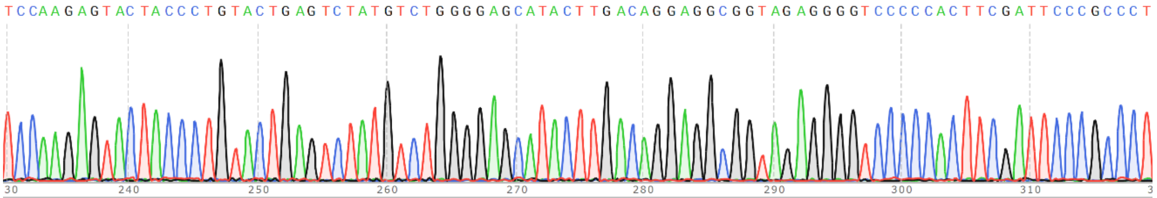


```
PCR_C3 : tccaagagtactaccctgtactgagtctatgtctggggagcatacttgacaggaggcggtagaggggtcccccacttcgattcccgccct : 270
R02_C3 : tccaagagtactaccctgtactgagtctatgtctggggagcatacttgacaggaggcggtagaggggtcccccacttcgattcccgccct : 270
         TCCAAGAGTACTACCCTGTACTGAGTCTATGTCTGGGGAGCATACTTGACAGGAGGCGGTAGAGGGGTCCCCCACTTCGATTCCCGCCCT
```

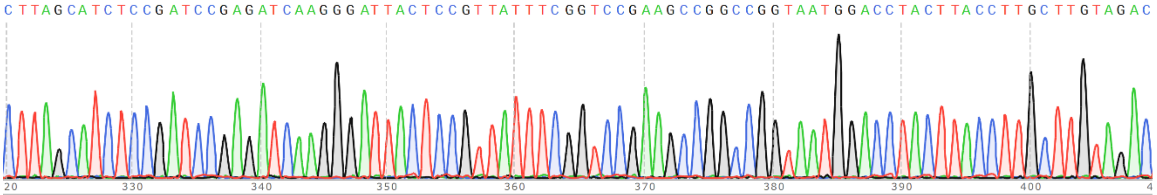


```
PCR_C3 : cttagcatctccgatccgagatcaagggattactccgttatttcggtccgaagccggccggtaatggacctacttaccttgcttgtagac : 360
R02_C3 : cttagcatctccgatccgagatcaagggattactccgttatttcggtccgaagccggccggtaatggacctacttaccttgcttgtagac : 360
         CTTAGCATCTCCGATCCGAGATCAAGGGATTACTCCGTTATTTCGGTCCGAAGCCGGCCGGTAATGGACCTACTTACCTTGCTTGTAGAC
```

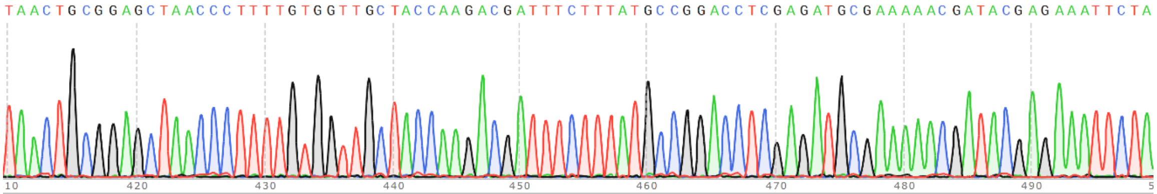


```
PCR_C3 : taactgcggagctaaccctttttgtggttgctaccaagacgatttctttatgccggacctcgagatgcgaaaaacgatacgagaaattcta : 450
R02_C3 : taactgcggagctaaccctttttgtggttgctaccaagacgatttctttatgccggacctcgagatgcgaaaaacgatacgagaaattcta : 450
         TAACTGCGGAGCTAACCCTTTTGTGGTTGCTACCAAGACGATTTCTTTATGCCGGACCTCGAGATGCGAAAAACGATACGAGAAATTCTA
```

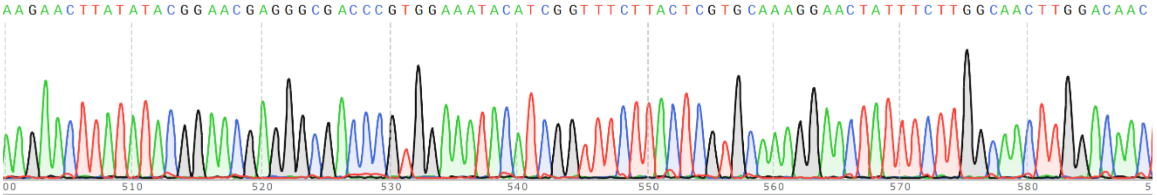


```
PCR_C3 : aagaacttatatacggaacgagggcgacccgtggaaatacatcggtttcttactcgtgcaaaggaactatttcttggcaacttggacaac : 540
R02_C3 : aagaacttatatacggaacgagggcgacccgtggaaatacatcggtttcttactcgtgcaaaggaactatttcttggcaacttggacaac : 540
         AAGAACTTATATACGGAACGAGGGCGACCCGTGGAAATACATCGGTTTCTTACTCGTGCAAAGGAACTATTTCTTGGCAACTTGGACAAC
```

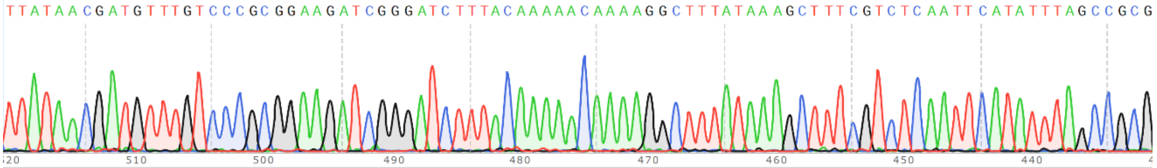


```
PCR_C3 : ttataacgatgtttgtcccgcggaagatcgggatctttacaaaaacaaaaggctttataaagctttcgtctcaattcatatttagccgcg : 630
R02_C3 : ttataacgatgtttgtcccgcggaagatcgggatctttacaaaaacaaaaggctttataaagctttcgtctcaattcatatttagccgcg : 630
         TTATAACGATGTTTGTCCCGCGGAAGATCGGGATCTTTACAAAAACAAAAGGCTTTATAAAGCTTTCGTCTCAATTCATATTTAGCCGCG
```

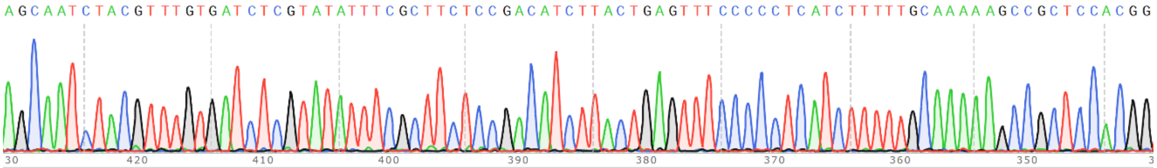


```
PCR_C3 : agcaatctacgtttgtgatctcgtatatttcgcttctccgacatcttactgagtttcccccctcatctttttgcaaaaagccgctccacgg : 720
R02_C3 : agcaatctacgtttgtgatctcgtatatttcgcttctccgacatcttactgagtttcccccctcatctttttgcaaaaagccgctccacgg : 720
         AGCAATCTACGTTTGTGATCTCGTATATTTCGCTTCTCCGACATCTTACTGAGTTTCCCCCTCATCTTTTTGCAAAAAGCCGCTCCACGG
```

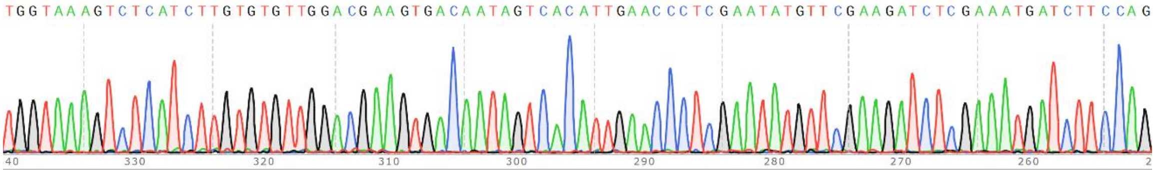


```
PCR_C3 : tggtaaagtctcatcttgtgtgttggacgaagtgacaatagtcacattgaaccctcgaatatgttcgaagatctcgaaatgatcttccag : 810
R02_C3 : tggtaaagtctcatcttgtgtgttggacgaagtgacaatagtcacattgaaccctcgaatatgttcgaagatctcgaaatgatcttccag : 810
         TGGTAAAGTCTCATCTTGTGTGTTGGACGAAGTGACAATAGTCACATTGAACCCTCGAATATGTTCGAAGATCTCGAAATGATCTTCCAG
```

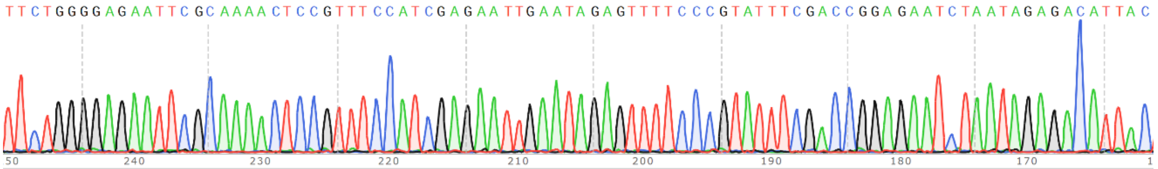

PCR_C3 : ttctggggagaattcgcaaaactccgtttccatcgagaattgaatagagttttcccgtatttcgaccggagaatctaatagagacattac : 900

R02_C3 : ttctggggagaattcgcaaaactccgtttccatcgagaattgaatagagttttcccgtatttcgaccggagaatctaatagagacattac : 900

TTCTGGGGAGAATTCGCAAAACTCCGTTTCCATCGAGAATTGAATAGAGTTTTCCCGTATTTCGACCGGAGAATCTAATAGAGACATTAC

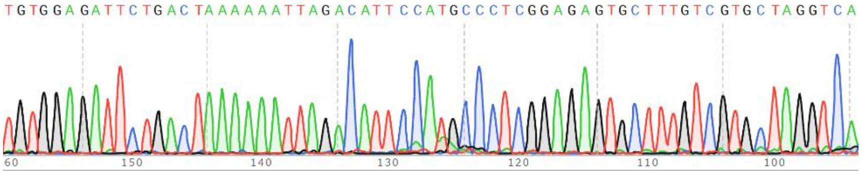

PCR_C3 : tgtggagattctgactaaaaaattagacattccatgccctcggagagtgctttgtcgtgctaggtca : 967

R02_C3 : tgtggagattctgactaaaaaattagacattccatgccctcggagagtgctttgtcgtgctaggtca : 967

TGTGGAGATTCTGACTAAAAAATTAGACATTCCATGCCCTCGGAGAGTGCTTTGTCGTGCTAGGTCA

h comparison of the sequence obtained from PCR amplification and Sanger sequencing (R01-C4) and the expected sequence.

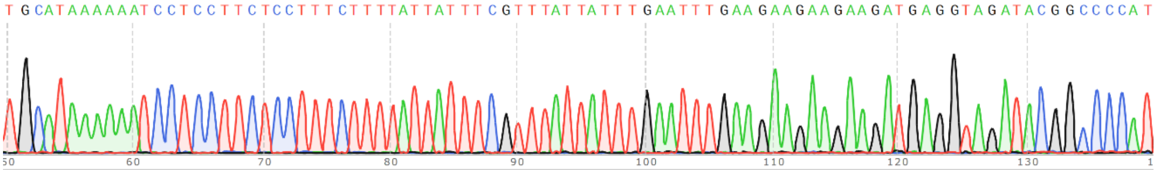

PCR_C4 : tgcataaaaaatcctccttctcctttcttttattatttcgtttattatttgaatttgaagaagaagaagatgaggtagatacggccccat : 90

R02_C4 : tgcataaaaaatcctccttctcctttcttttattatttcgtttattatttgaatttgaagaagaagaagatgaggtagatacggccccat : 90

TGCATAAAAAATCCTCCTTCTCCTTTCTTTTATTATTTCGTTTATTATTTGAATTTGAAGAAGAAGAAGATGAGGTAGATACGGCCCCAT

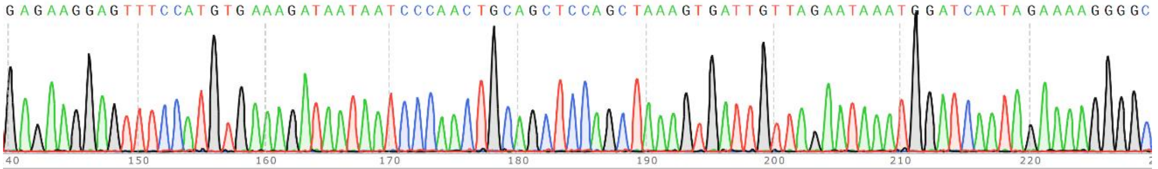

PCR_C4 : gagaaggagtttccatgtgaaagataataatcccaactgcagctccagctaaagtgattgttagaataaatggatcaatagaaaagggggc : 180

R02_C4 : gagaaggagtttccatgtgaaagataataatcccaactgcagctccagctaaagtgattgttagaataaatggatcaatagaaaagggggc : 180

GAGAAGGAGTTTCCATGTGAAAGATAATAATCCCAACTGCAGCTCCAGCTAAAGTGATTGTTAGAATAAATGGATCAATAGAAAAGGGGC

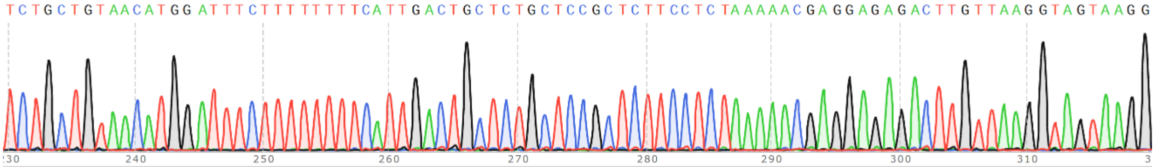

PCR_C4 : tctgctgtaacatggatttcttttttttcattgactgctctgctccgctcttcctctaaaaacgaggagagacttgttaaggtagtaagg : 270

R02_C4 : tctgctgtaacatggatttcttttttttcattgactgctctgctccgctcttcctctaaaaacgaggagagacttgttaaggtagtaagg : 270

TCTGCTGTAACATGGATTTCTTTTTTTTCATTGACTGCTCTGCTCCGCTCTTCCTCTAAAAACGAGGAGAGACTTGTTAAGGTAGTAAGG

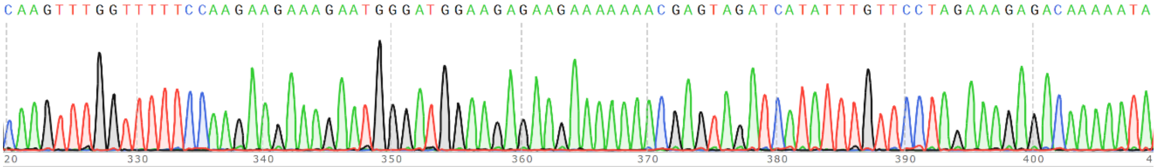

PCR_C4 : caagtttggtttttccaagaagaaagaatgggatggaagagaagaaaaaaacgagtagatcatatttgttcctagaaagagacaaaaata : 360

R02_C4 : caagtttggtttttccaagaagaaagaatgggatggaagagaagaaaaaaacgagtagatcatatttgttcctagaaagagacaaaaata : 360

CAAGTTTGGTTTTTCCAAGAAGAAAGAATGGGATGGAAGAGAAGAAAAAAACGAGTAGATCATATTTGTTCCTAGAAAGAGACAAAAATA

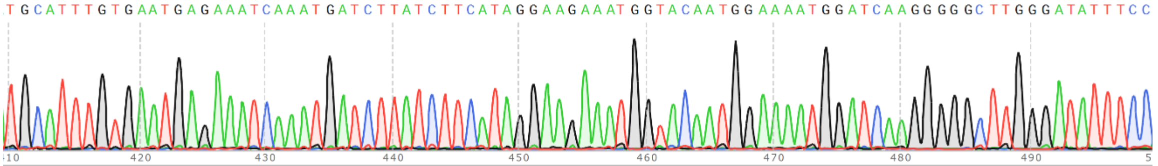

PCR_C4 : tgcatttgtgaatgagaaatcaaatgatcttatcttcataggaagaaatggtacaatggaaaatggatcaaggggggcttgggatatttcc : 450

R02_C4 : tgcatttgtgaatgagaaatcaaatgatcttatcttcataggaagaaatggtacaatggaaaatggatcaaggggggcttgggatatttcc : 450

TGCATTTGTGAATGAGAAATCAAATGATCTTATCTTCATAGGAAGAAATGGTACAATGGAAAATGGATCAAGGGGGCTTGGGATATTTCC

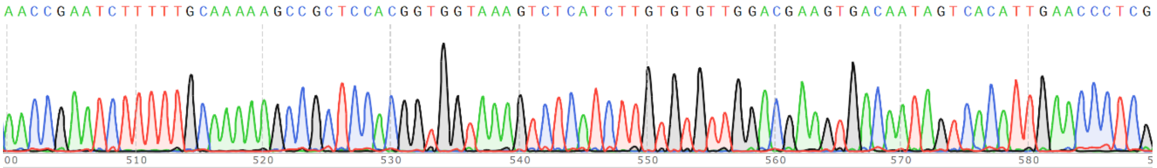

PCR_C4 : aaccgaatctttttgcaaaaagccgctccacggtggtaaagtctcatcttgtgtgttggacgaagtgacaatagtcacattgaaccctcg : 540

R02_C4 : aaccgaatctttttgcaaaaagccgctccacggtggtaaagtctcatcttgtgtgttggacgaagtgacaatagtcacattgaaccctcg : 540

AACCGA**ATCTTTTTGCAAAAAGCCGCTCCACGGTGGTAAAGTCTCATCTTGTGTGTTGGACGAAGTGACAATAGTCACATTGAACCCTCG**

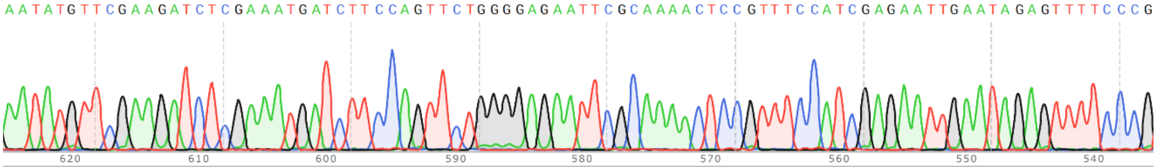

PCR_C4 : aatatgttcgaagatctcgaaatgatcttccagttctggggagaattcgcaaaactccgtttccatcgagaattgaatagagttttcccg : 630

R02_C4 : aatatgttcgaagatctcgaaatgatcttccagttctggggagaattcgcaaaactccgtttccatcgagaattgaatagagttttcccg : 630

AATATGTTCGAAGATCTCGAAATGATCTTCCAGTTCTGGGGAGAATTCGCAAAACTCCGTTTCCATCGAGAATTGAATAGAGTTTTCCCG

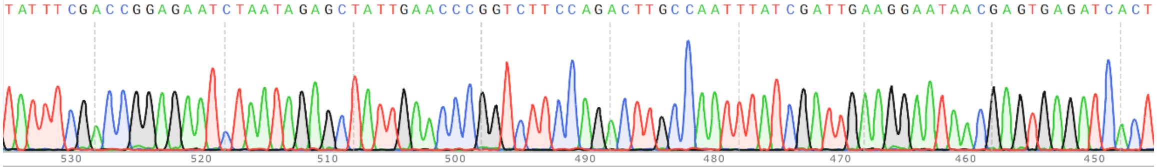

PCR_C4 : tatttcgaccggagaatctaatagagctattgaacccggtcttccagacttgccaatttatcgattgaaggaataacgagtgagatcact : 720

R02_C4 : tatttcgaccggagaatctaatagagctattgaacccggtcttccagacttgccaatttatcgattgaaggaataacgagtgagatcact : 720

TATTTCGACCGGAGAATCTAATAGAGCTATTGAACCCGGTCTTCCAGACTTGCCAATTTATCGATTGAAGGAATAACGAGTGAGATCACT

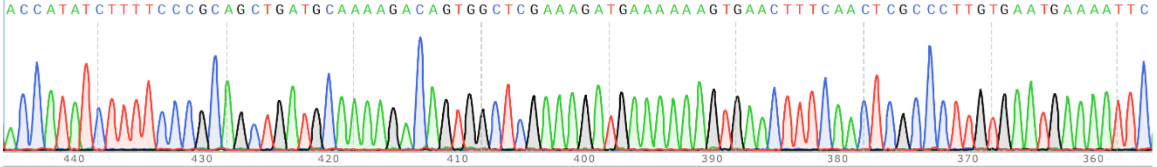

PCR_C4 : accatatcttttcccgcagctgatgcaaaagacagtggctcgaaagatgaaaaaagtgaactttcaactcgcccttgtgaatgaaaattc : 810

R02_C4 : accatatcttttcccgcagctgatgcaaaagacagtggctcgaaagatgaaaaaagtgaactttcaactcgcccttgtgaatgaaaattc : 810

ACCATATCTTTTCCCGCAGCTGATGCAAAAGACAGTGGCTCGAAAGATGAAAAAAGTGAACTTTCAACTCGCCCTTGTGAATGAAAATTC

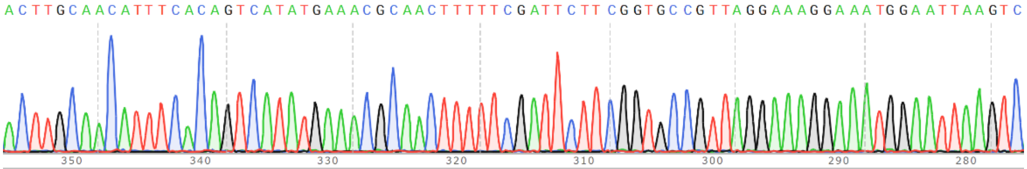

PCR_C4 : acttgcaacatttcacagtcatatgaaacgcaactttttcgattcttcggtgccgttaggaaaggaaatggaattaagtc : 890

R02_C4 : acttgcaacatttcacagtcatatgaaacgcaactttttcgattcttcggtgccgttaggaaaggaaatggaattaagtc : 890

ACTTGCAACATTTCACAGTCATATGAAACGCAACTTTTTCGATTCTTCGGTGCCGTTAGGAAAGGAAATGGAATTAAGTC

i comparison of the sequence obtained from PCR amplification and Sanger sequencing (R47-C1) and the expected sequence.

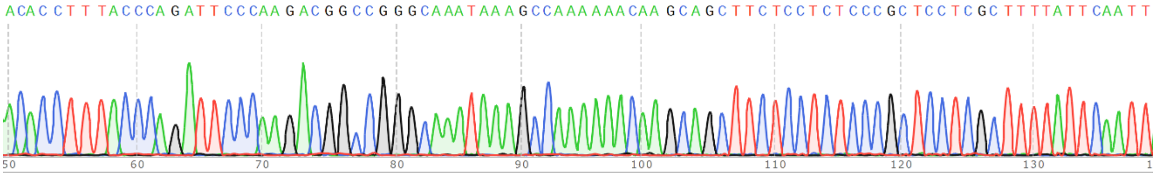

PCR_C1 : acacctttacccagattcccaagacggccgggcaaataaagccaaaaaaacaagcagcttctcctctcccgctcctcgcttttattcaatt : 90

R47_C1 : acacctttacccagattcccaagacggccgggcaaataaagccaaaaaaacaagcagcttctcctctcccgctcctcgcttttattcaatt : 90

ACACCTTTACCCAGATTCCCAAGACGGCCGGGCAAATAAAGCCAAAAAAACAAGCAGCTTCTCCTCTCCCGCTC**CTCGCTTTTATTCAATT**

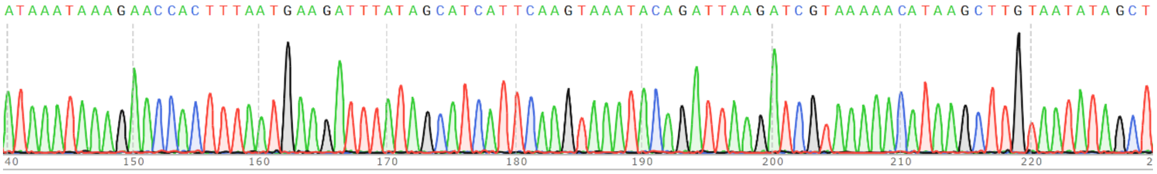

```
PCR_C1 : ataaataaagaaccactttaatgaagatttatagcatcattcaagtaaatacagattaagatcgtaaaaacataagcttgtaatatagct : 180
R47_C1 : ataaataaagaaccactttaatgaagatttatagcatcattcaagtaaatacagattaagatcgtaaaaacataagcttgtaatatagct : 180
         ATAAATAAAGAACCACTTTAATGAAGATTTATAGCATCATTCAAGTAAATACAGATTAAGATCGTAAAAACATAAGCTTGTAATATAGCT
```

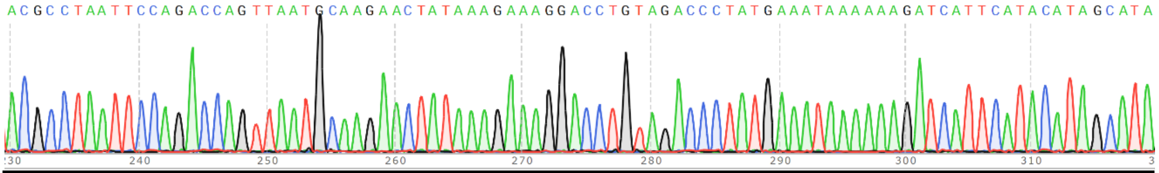


```
PCR_C1 : acgcctaattccagaccagttaatgcaagaactataaagaaaggacctgtagaccctatgaaataaaaaagatcattcatacatagcata : 270
R47_C1 : acgcctaattccagaccagttaatgcaagaactataaagaaaggacctgtagaccctatgaaataaaaaagatcattcatacatagcata : 270
         ACGCCTAATTCCAGACCAGTTAATGCAAGAACTATAAAGAAAGGACCTGTAGACCCTATGAAATAAAAAAGATCATTCATACATAGCATA
```

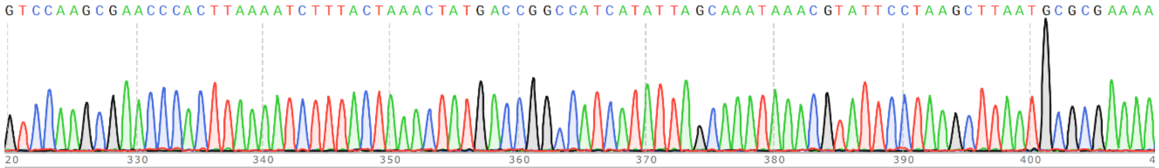


```
PCR_C1 : gtccaagcgaacccacttaaaatctttactaaactatgaccggccatcatattagcaaataaacgtattcctaagcttaatgcgcgaaaa : 360
R47_C1 : gtccaagcgaacccacttaaaatctttactaaactatgaccggccatcatattagcaaataaacgtattcctaagcttaatgcgcgaaaa : 360
         GTCCAAGCGAACCCACTTAAAATCTTTACTAAACTATGACCGGCCATCATATTAGCAAATAAACGTATTCCTAAGCTTAATGCGCGAAAA
```

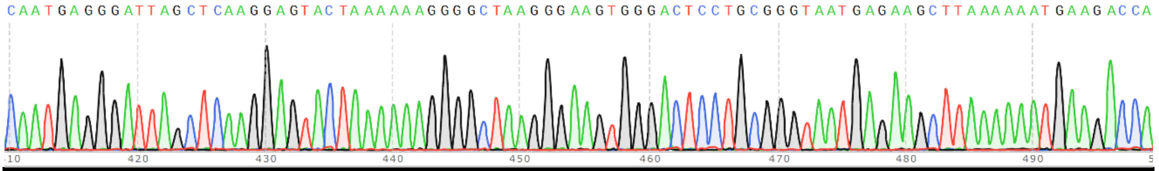


```
PCR_C1 : caatgagggattagctcaaggagtactaaaaaaggggctaagggaagtgggactcctgcgggtaatgagaagcttaaaaaatgaagacca : 450
R47_C1 : caatgagggattagctcaaggagtactaaaaaaggggctaagggaagtgggactcctgcgggtaatgagaagcttaaaaaatgaagacca : 450
         CAATGAGGGATTAGCTCAAGGAGTACTAAAAAAGGGGCTAAGGGAAGTGGGACTCCTGCGGGTAATGAGAAGCTTAAAAAATGAAGACCA
```

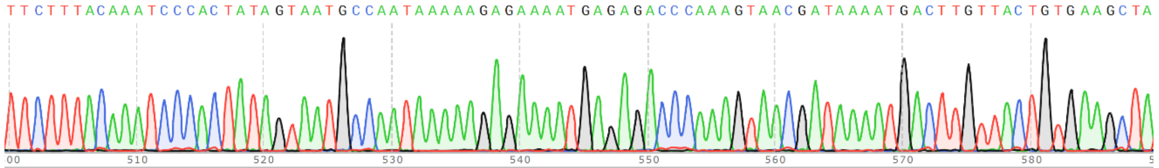


```
PCR_C1 : ttctttacaaatcccactatagtaatgccaataaaaagagaaaatgagagacccaaagtaacgataaaatgacttgttactgtgaagcta : 540
R47_C1 : ttctttacaaatcccactatagtaatgccaataaaaagagaaaatgagagacccaaagtaacgataaaatgacttgttactgtgaagcta : 540
         TTCTTTACAAATCCCACTATAGTAATGCCAATAAAAAGAGAAAATGAGAGACCCAAAGTAACGATAAAATGACTTGTTACTGTGAAGCTA
```

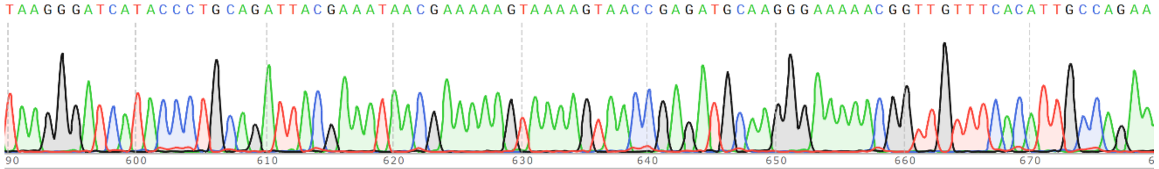


```
PCR_C1 : taagggatcatacccctgcagattacgaaataacgaaaaagtaaaagtaaccgagatgcaagggaaaaacggttgtttcacattgccagaa : 630
R47_C1 : taagggatcatacccctgcagattacgaaataacgaaaaagtaaaagtaaccgagatgcaagggaaaaacggttgtttcacattgccagaa : 630
         TAAGGGATCATACCCTGCAGATTACGAAATAACGAAAAAGTAAAAGTAACCGAGATGCAAGGGAAAAACGGTTGTTTCACATTGCCAGAA
```

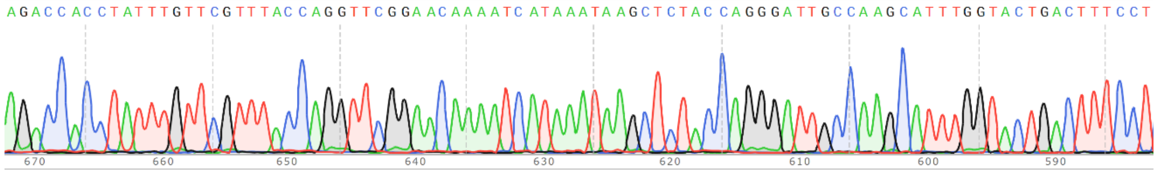


```
PCR_C1 : agaccacctatttgttcgtttaccaggttcggaacaaaatcataaataagctctaccagggattgccaagcatttggtactgactttcct : 720
R47_C1 : agaccacctatttgttcgtttaccaggttcggaacaaaatcataaataagctctaccagggattgccaagcatttggtactgactttcct : 720
         AGACCACCTATTTGTTCGTTTACCAGGTTCGGAACAAAATCATAAATAAGCTCTACCAGGGATTGCCAAGCATTTGGTACTGACTTTCCT
```

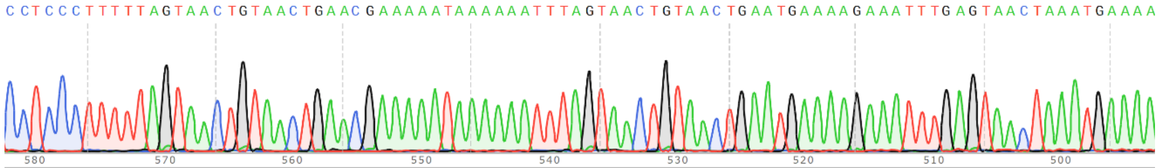


```
PCR_C1 : cctcccttttttagtaactgtaactgaacgaaaaataaaaaaatttagtaactgtaactgaatgaaaagaaatttgagtaactaaatgaaaa : 810
R47_C1 : cctcccttttttagtaactgtaactgaacgaaaaataaaaaaatttagtaactgtaactgaatgaaaagaaatttgagtaactaaatgaaaa : 810
         CCTCCCTTTTTTAGTAACTGTAACTGAACGAAAAATAAAAAAATTTAGTAACTGTAACTGAATGAAAAGAAATTTGAGTAACTAAATGAAAA
```

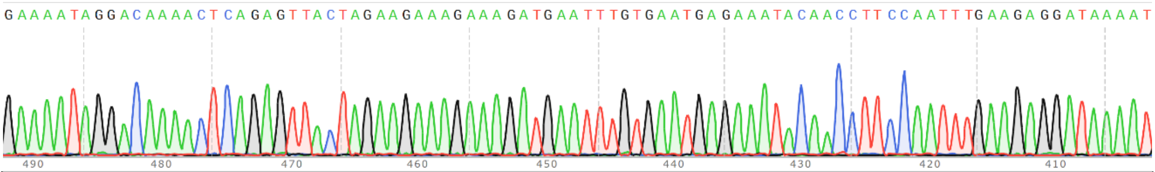

PCR_C1 : gaaaataggacaaaactcagagttactagaagaaagaaagatgaatttgtgaatgagaaatacaaccttccaatttgaagaggataaaat : 900

R47_C1 : gaaaataggacaaaactcagagttactagaagaaagaaagatgaatttgtgaatgagaaatacaaccttccaatttgaagaggataaaat : 900

GAAAATAGGACAAAACTCAGAGTTACTAGAAGAAAGAAAGATGAATTTGTGAATGAGAAATACAACCTTCCAATTTGAAGAGGATAAAAT

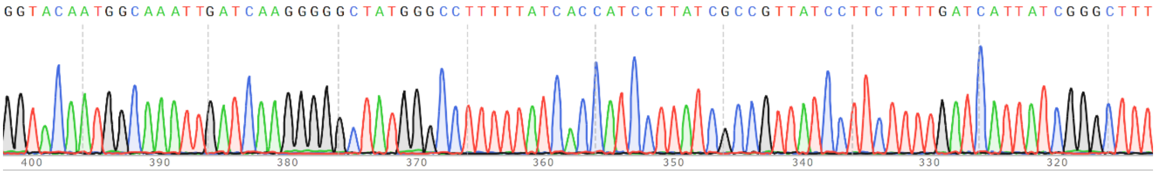

PCR_C1 : ggtacaatggcaaattgatcaagggggctatgggcctttttatcaccatccttatcgccgttatccttcttttgatcattatcgggcttt : 990

R47_C1 : ggtacaatggcaaattgatcaagggggctatgggcctttttatcaccatccttatcgccgttatccttcttttgatcattatcgggcttt : 990

GGTACAATGGCAAATTGATCAAGGGGGCTATGGGCCTTTTTATCACCATCCTTATCGCCGTTATCCTTCTTTTGATCATTATCGGGCTTT

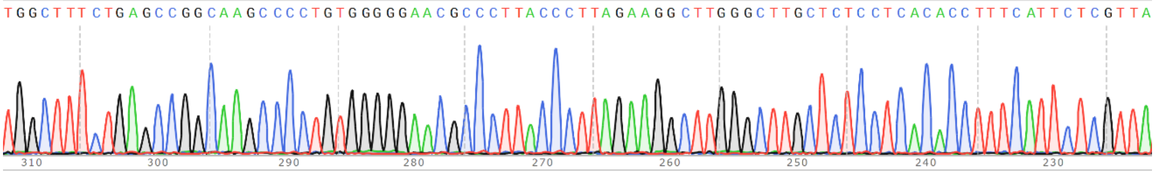

PCR_C1 : tggctttctgagccggcaagcccctgtgggggaacgcccttacccttagaaggcttgggcttgctctcctcacacctttcattctcgtta : 1080

R47_C1 : tggctttctgagccggcaagcccctgtgggggaacgcccttacccttagaaggcttgggcttgctctcctcacacctttcattctcgtta : 1080

TGGCTTTCTGAGCCGGCAAGCCCCTGTGGGGGAACGCCCTTACCCTTAGAAGGCTTGGGCTTGCTCTCCTCACACCTTTCATTCTCGTTA

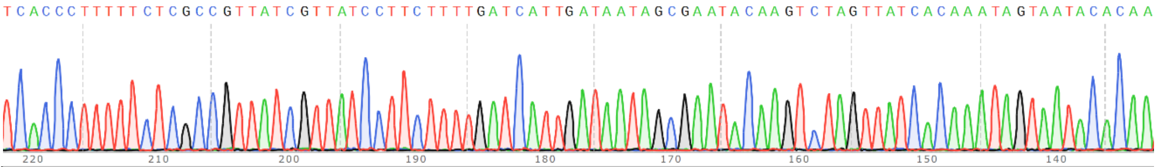

PCR_C1 : tcaccctttttctcgccgttatcgttatccttcttttgatcattgataatagcgaatacaagtctagttatcacaaatagtaatacacaa : 1170

R47_C1 : tcaccctttttctcgccgttatcgttatccttcttttgatcattgataatagcgaatacaagtctagttatcacaaatagtaatacacaa : 1170

TCACCCTTTTTCTCGCCGTTATCGTTATCCTTCTTTTGATCATTGATAATAGCGAATACAAGTCTAGTTATCACAAATAGTAATACACAA

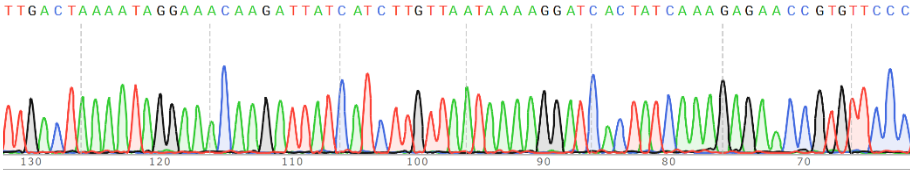

PCR_C1 : ttgactaaaataggaaacaagattatcatcttgttaataaaaggatcactatcaaagagaaccgtgttccc : 1241

R47_C1 : ttgactaaaataggaaacaagattatcatcttgttaataaaaggatcactatcaaagagaaccgtgttccc : 1241

TTGACTAAAATAGGAAACAAGATTATCATCTTGTTAATAAAAGGATCACTATCAAAGAGAACCGTGTTCCC

j comparison of the sequence obtained from PCR amplification and Sanger sequencing (R47-C2) and the expected sequence.

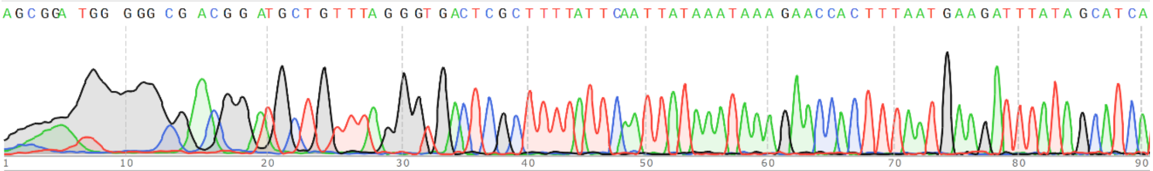

PCR_C2 : agcggatgggggcgacggatgctgtttagggtgactcgcttttattcaattataaataaagaaccactttaatgaagatttatagcatca : 90

R47_C2 : agcggatgggggcgacggatgctgtttagggtgactcgcttttattcaattataaataaagaaccactttaatgaagatttatagcatca : 90

AGCGGATGGGGGCGACGGATGCTGTTTAGGGTGA**CTCGCTTTTATTCAATTATAAATAAAGAACCACTTTAATGAAGATTTATAGCATCA**

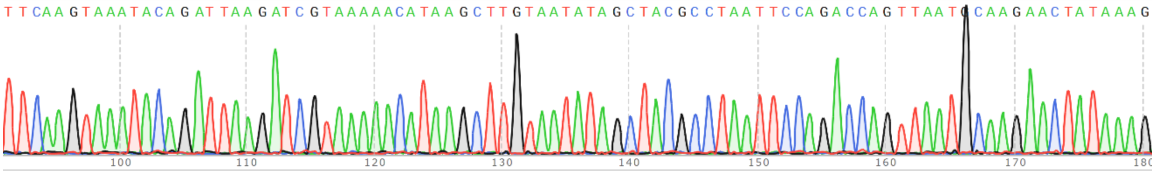

PCR_C2 : ttcaagtaaatacagattaagatcgtaaaaacataagcttgtaatatagctacgcctaattccagaccagttaatgcaagaactataaag : 180

R47_C2 : ttcaagtaaatacagattaagatcgtaaaaacataagcttgtaatatagctacgcctaattccagaccagttaatgcaagaactataaag : 180

TTCAAGTAAATACAGATTAAGATCGTAAAAACATAAGCTTGTAATATAGCTACGCCTAATTCCAGACCAGTTAATGCAAGAACTATAAAG

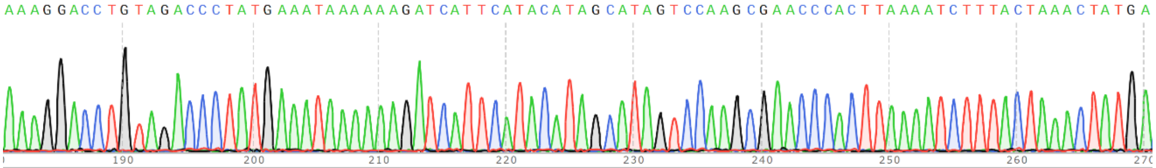

```
PCR_C2 : aaaggacctgtagaccctatgaaataaaaaagatcattcatacatagcatagtccaagcgaacccacttaaaatctttactaaactatga : 270
R47_C2 : aaaggacctgtagaccctatgaaataaaaaagatcattcatacatagcatagtccaagcgaacccacttaaaatctttactaaactatga : 270
         AAAGGACCTGTAGACCCTATGAAATAAAAAAGATCATTCATACATAGCATAGTCCAAGCGAACCCACTTAAAATCTTTACTAAACTATGA
```

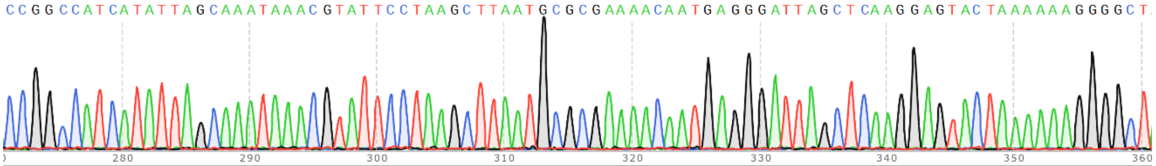

```
PCR_C2 : ccggccatcatattagcaaataaacgtattcctaagcttaatgcgcgaaaacaatgagggattagctcaaggagtactaaaaaaggggct : 360
R47_C2 : ccggccatcatattagcaaataaacgtattcctaagcttaatgcgcgaaaacaatgagggattagctcaaggagtactaaaaaaggggct : 360
         CCGGCCATCATATTAGCAAATAAACGTATTCCTAAGCTTAATGCGCGAAAACAATGAGGGATTAGCTCAAGGAGTACTAAAAAAGGGGCT
```

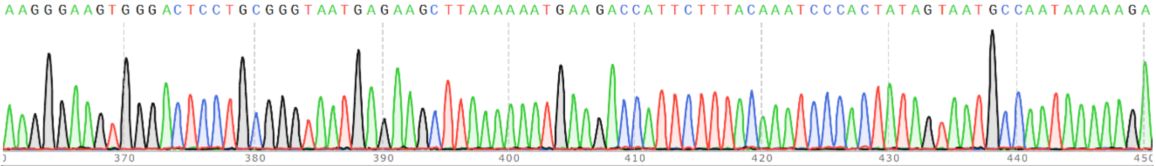

```
PCR_C2 : aagggaagtgggactcctgcgggtaatgagaagcttaaaaaaatgaagaccattctttacaaatcccactatagtaatgccaataaaaaga : 450
R47_C2 : aagggaagtgggactcctgcgggtaatgagaagcttaaaaaaatgaagaccattctttacaaatcccactatagtaatgccaataaaaaga : 450
         AAGGGAAGTGGGACTCCTGCGGGTAATGAGAAGCTTAAAAAAATGAAGACCATTCTTTACAAATCCCACTATAGTAATGCCAATAAAAAGA
```

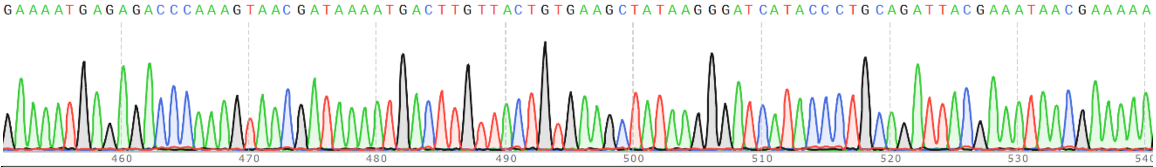

```
PCR_C2 : gaaaatgagagacccaaagtaacgataaaatgacttgttactgtgaagctataagggatcataccctgcagattacgaaataacgaaaaa : 540
R47_C2 : gaaaatgagagacccaaagtaacgataaaatgacttgttactgtgaagctataagggatcataccctgcagattacgaaataacgaaaaa : 540
         GAAAATGAGAGACCCAAAGTAACGATAAAATGACTTGTTACTGTGAAGCTATAAGGGATCATACCCTGCAGATTACGAAATAACGAAAAA
```

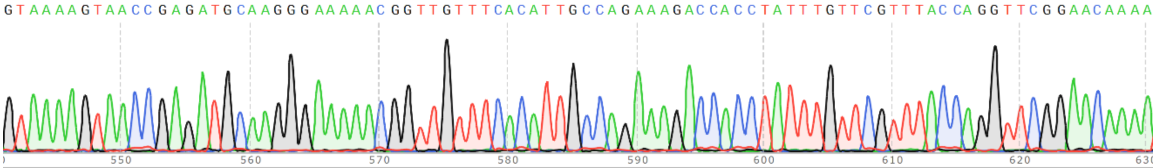

```
PCR_C2 : gtaaaagtaaccgagatgcaagggaaaaacggttgtttcacattgccagaaagaccacctatttgttcgtttaccaggttcggaacaaaa : 630
R47_C2 : gtaaaagtaaccgagatgcaagggaaaaacggttgtttcacattgccagaaagaccacctatttgttcgtttaccaggttcggaacaaaa : 630
         GTAAAAGTAACCGAGATGCAAGGGAAAAACGGTTGTTTCACATTGCCAGAAAGACCACCTATTTGTTCGTTTACCAGGTTCGGAACAAAA
```

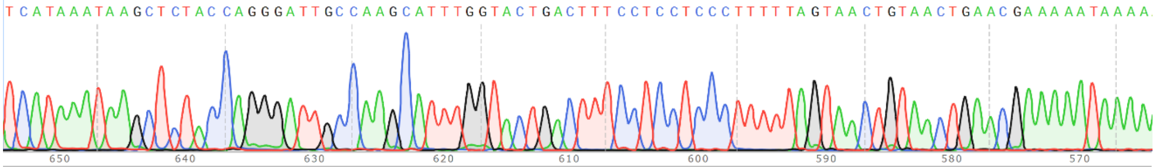

```
PCR_C2 : tcataaataagctctaccagggattgccaagcatttggtactgactttcctcctcccttttttagtaactgtaactgaacgaaaaaataaaa : 720
R47_C2 : tcataaataagctctaccagggattgccaagcatttggtactgactttcctcctcccttttttagtaactgtaactgaacgaaaaaataaaa : 720
         TCATAAATAAGCTCTACCAGGGATTGCCAAGCATTTGGTACTGACTTTCCTCCTCCCTTTTTTAGTAACTGTAACTGAACGAAAAAATAAAA
```

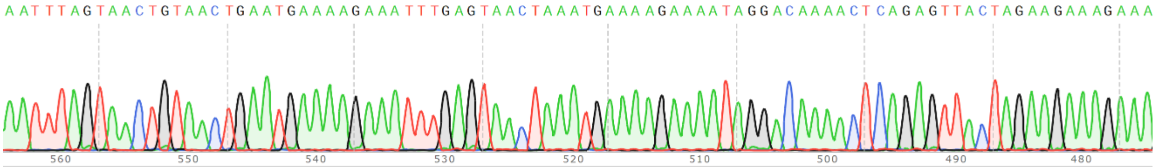

```
PCR_C2 : aatttagtaactgtaactgaatgaaaagaaatttgagtaactaaatgaaaagaaaataggacaaaactcagagttactagaagaaagaaa : 810
R47_C2 : aatttagtaactgtaactgaatgaaaagaaatttgagtaactaaatgaaaagaaaataggacaaaactcagagttactagaagaaagaaa : 810
         AATTTAGTAACTGTAACTGAATGAAAAGAAATTTGAGTAACTAAATGAAAAGAAAATAGGACAAAACTCAGAGTTACTAGAAGAAAGAAA
```

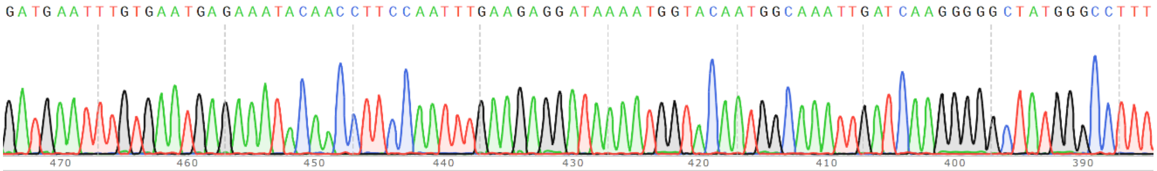

PCR_C2 : gatgaatttgtgaatgagaaatacaaccttccaatttgaagaggataaaatggtacaatggcaaattgatcaagggggctatgggccttt : 900
R47_C2 : gatgaatttgtgaatgagaaatacaaccttccaatttgaagaggataaaatggtacaatggcaaattgatcaagggggctatgggccttt : 900
GATGAATTTGTGAATGAGAAATACAACCTTCCAATTTGAAGAGGATAAAATGGTACAATGGCAAATTGATCAAGGGGGCTATGGGCCTTT

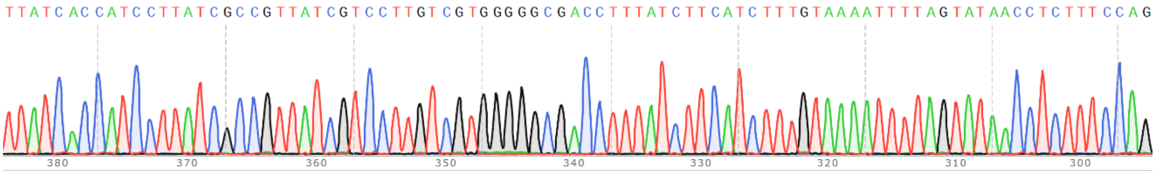

PCR_C2 : ttatcaccatccttatcgccgttatcgtccttgtcgtgggggcgacctttatcttcatctttgtaaaattttagtataacctctttccag : 990
R47_C2 : ttatcaccatccttatcgccgttatcgtccttgtcgtgggggcgacctttatcttcatctttgtaaaattttagtataacctctttccag : 990
TTATCACCATCCTTATCGCCGTTATCGTCCTTGTCGTGGGGGCGACCTTTATCTTCATCTTTGTAAAATTTTAGTATAACCTCTTTCCAG

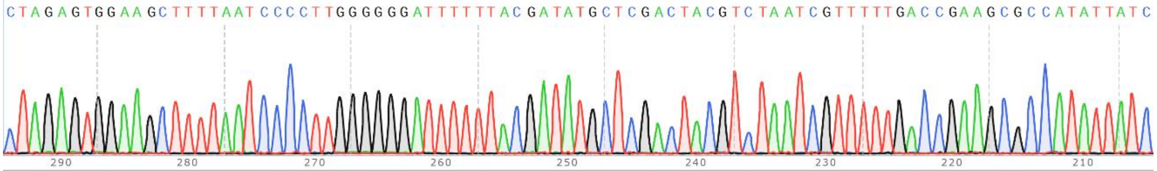

PCR_C2 : ctagagtggaagcttttaatccccttggggggattttttacgatatgctcgactacgtctaatcgtttttgaccgaagcgccatattatc : 1080
R47_C2 : ctagagtggaagcttttaatccccttggggggattttttacgatatgctcgactacgtctaatcgtttttgaccgaagcgccatattatc : 1080
CTAGAGTGGAAGCTTTTAATCCCCTTGGGGGGATTTTTTACGATATGCTCGACTACGTCTAATCGTTTTTGACCGAAGCGCCATATTATC

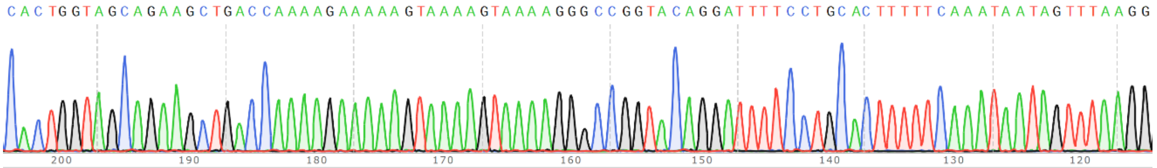

PCR_C2 : cactggtagcagaagctgaccaaaagaaaaagtaaaagtaaaagggccggtacaggattttcctgcactttttcaaataatagtttaagg : 1170
R47_C2 : cactggtagcagaagctgaccaaaagaaaaagtaaaagtaaaagggccggtacaggattttcctgcactttttcaaataatagtttaagg : 1170
CACTGGTAGCAGAAGCTGACCAAAAGAAAAAGTAAAAGTAAAAGGGCCGGTACAGGATTTTCCTGCACTTTTTCAAATAATAGTTTAAGG

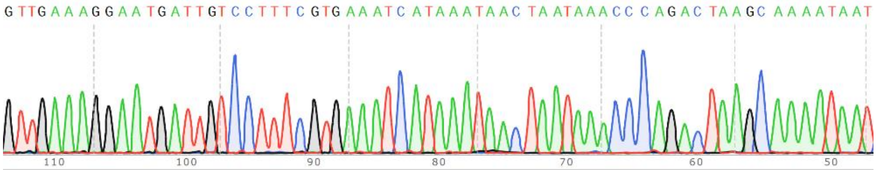

PCR_C2 : gttgaaaggaatgattgtcctttcgtgaaatcataaataactaataaacccagactaagcaaaataat : 1238
R47_C2 : gttgaaaggaatgattgtcctttcgtgaaatcataaataactaataaacccagactaagcaaaataat : 1238
GTTGAAAGGAATGATTGTCCTTTCGTGAAATCATAAATAACTAATAAACCCAGACTAAGCAAAATAAT

k comparison of the sequence obtained from PCR amplification and Sanger sequencing (R47-C3) and the expected sequence.

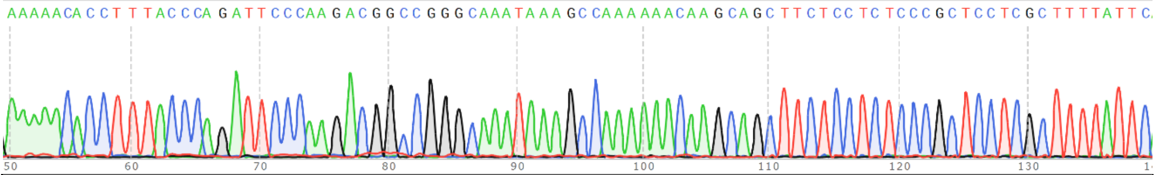

PCR_C3 : aaaaacacctttacccagattcccaagacggccgggcaaataaagccaaaaaacaagcagcttctcctctcccgctcctcgcttttattc : 90
R47_C3 : aaaaacacctttacccagattcccaagacggccgggcaaataaagccaaaaaacaagcagcttctcctctcccgctcctcgcttttattc : 90
AAAAACACCTTTACCCAGATTCCCAAGACGGCCGGGCAAATAAAGCCAAAAAACAAGCAGCTTCTCCTCTCCCGCTC**CTCGCTTTTATTC**

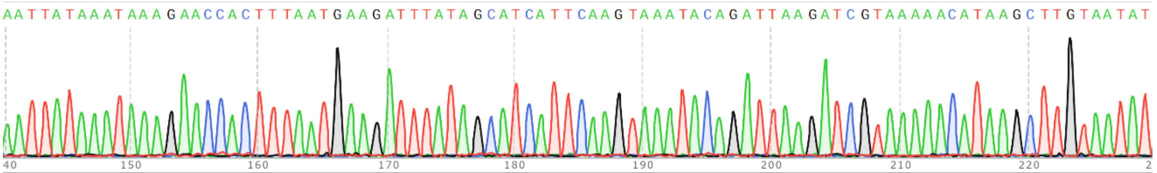

PCR_C3 : aattataaataaagaaccactttaatgaagatttatagcatcattcaagtaaatacagattaagatcgtaaaaacataagcttgtaatat : 180

R47_C3 : aattataaataaagaaccactttaatgaagatttatagcatcattcaagtaaatacagattaagatcgtaaaaacataagcttgtaatat : 180
AATTATAAATAAAGAACCACTTTAATGAAGATTTATAGCATCATTCAAGTAAATACAGATTAAGATCGTAAAAACATAAGCTTGTAATAT

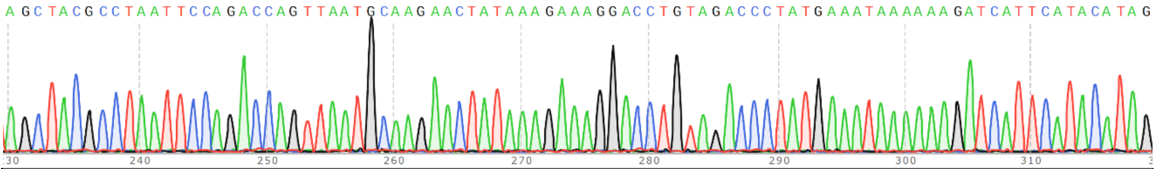

PCR_C3 : agctacgcctaattccagaccagttaatgcaagaactataaagaaaggacctgtagaccctatgaaataaaaaagatcattcatacatag : 270
R47_C3 : agctacgcctaattccagaccagttaatgcaagaactataaagaaaggacctgtagaccctatgaaataaaaaagatcattcatacatag : 270
AGCTACGCCTAATTCCAGACCAGTTAATGCAAGAACTATAAAGAAAGGACCTGTAGACCCTATGAAATAAAAAAGATCATTCATACATAG

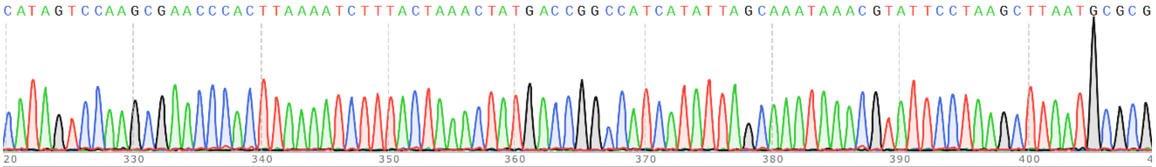

PCR_C3 : catagtccaagcgaacccacttaaaatctttactaaactatgaccggccatcatattagcaaataaacgtattcctaagcttaatgcgcg : 360
R47_C3 : catagtccaagcgaacccacttaaaatctttactaaactatgaccggccatcatattagcaaataaacgtattcctaagcttaatgcgcg : 360
CATAGTCCAAGCGAACCCACTTAAAATCTTTACTAAACTATGACCGGCCATCATATTAGCAAATAAACGTATTCCTAAGCTTAATGCGCG

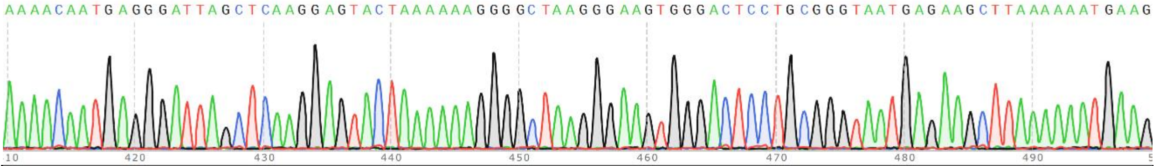

PCR_C3 : aaaacaatgagggattagctcaaggagtactaaaaaaggggctaagggaagtgggactcctgcgggtaatgagaagcttaaaaaatgaag : 450
R47_C3 : aaaacaatgagggattagctcaaggagtactaaaaaaggggctaagggaagtgggactcctgcgggtaatgagaagcttaaaaaatgaag : 450
AAAACAATGAGGGATTAGCTCAAGGAGTACTAAAAAAGGGGCTAAGGGAAGTGGGACTCCTGCGGGTAATGAGAAGCTTAAAAAATGAAG

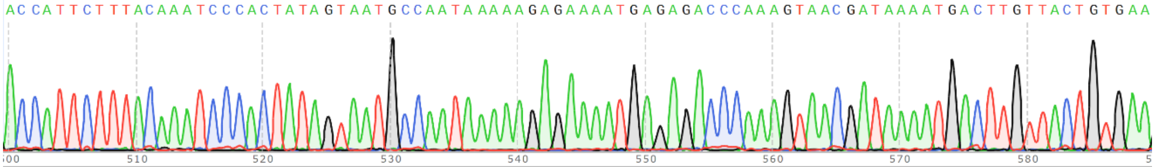

PCR_C3 : accattctttacaaatcccactatagtaatgccaataaaaagagaaaatgagagacccaaagtaacgataaaatgacttgttactgtgaa : 540
R47_C3 : accattctttacaaatcccactatagtaatgccaataaaaagagaaaatgagagacccaaagtaacgataaaatgacttgttactgtgaa : 540
ACCATTCTTTACAAATCCCACTATAGTAATGCCAATAAAAAGAGAAAATGAGAGACCCAAAGTAACGATAAAATGACTTGTTACTGTGAA

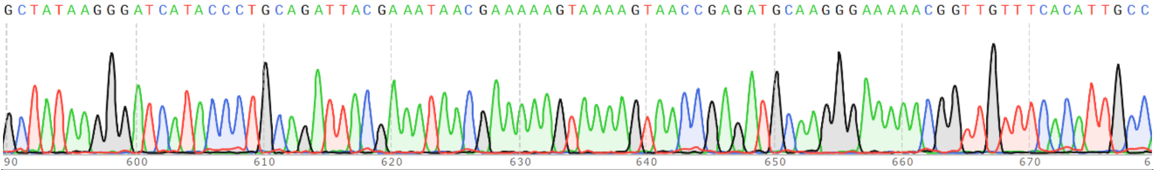

PCR_C3 : gctataagggatcataccctgcagattacgaaataacgaaaaagtaaaagtaaccgagatgcaagggaaaaacggttgtttcacattgcc : 630
R47_C3 : gctataagggatcataccctgcagattacgaaataacgaaaaagtaaaagtaaccgagatgcaagggaaaaacggttgtttcacattgcc : 630
GCTATAAGGGATCATACCCTGCAGATTACGAAATAACGAAAAAGTAAAAGTAACCGAGATGCAAGGGAAAAACGGTTGTTTCACATTGCC

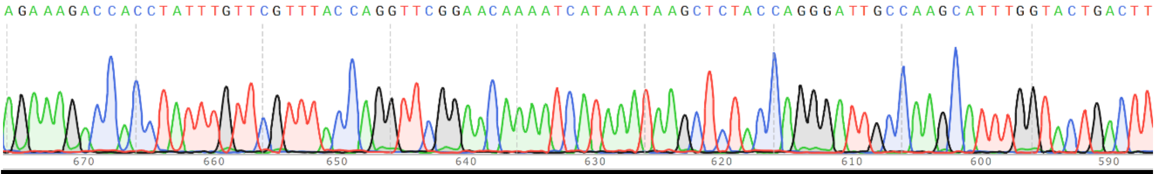

PCR_C3 : agaaagaccacctatttgttcgtttaccaggttcggaacaaaatcataaataagctctaccagggattgccaagcatttggtactgactt : 720
R47_C3 : agaaagaccacctatttgttcgtttaccaggttcggaacaaaatcataaataagctctaccagggattgccaagcatttggtactgactt : 720
AGAAAGACCACCTATTTGTTCGTTTACCAGGTTCGGAACAAAATCATAAATAAGCTCTACCAGGGATTGCCAAGCATTTGGTACTGACTT

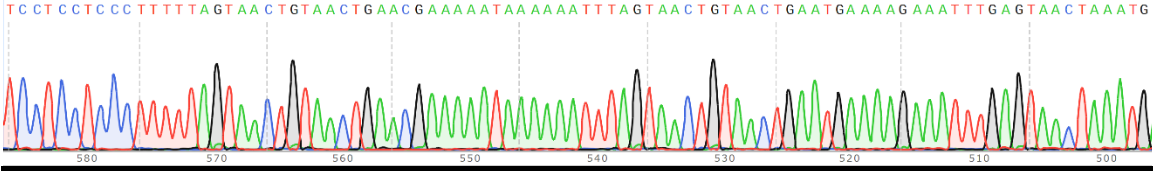

PCR_C3 : tcctcctcccttttttagtaactgtaactgaacgaaaaataaaaaaatttagtaactgtaactgaatgaaaagaaatttgagtaactaaatg : 810
R47_C3 : tcctcctcccttttttagtaactgtaactgaacgaaaaataaaaaaatttagtaactgtaactgaatgaaaagaaatttgagtaactaaatg : 810
TCCTCCTCCCTTTTTTAGTAACTGTAACTGAACGAAAAATAAAAAAATTTAGTAACTGTAACTGAATGAAAAGAAATTTGAGTAACTAAATG

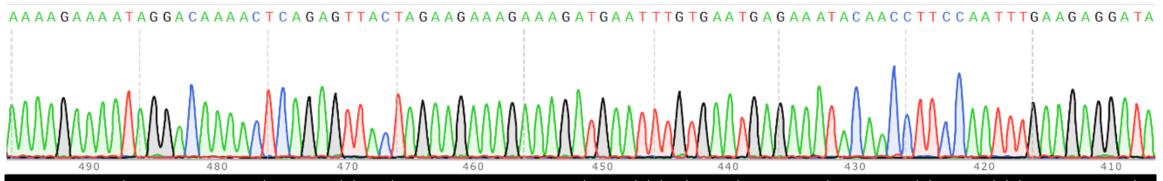

PCR_C3 : aaaagaaaataggacaaaactcagagttactagaagaaagaaagatgaatttgtgaatgagaaatacaaccttccaatttgaagaggata : 900
R47_C3 : aaaagaaaataggacaaaactcagagttactagaagaaagaaagatgaatttgtgaatgagaaatacaaccttccaatttgaagaggata : 900
AAAAGAAAATAGGACAAAACTCAGAGTTACTAGAAGAAAGAAAGATGAATTTGTGAATGAGAAATACAACCTTCCAATTTGAAGAGGATA

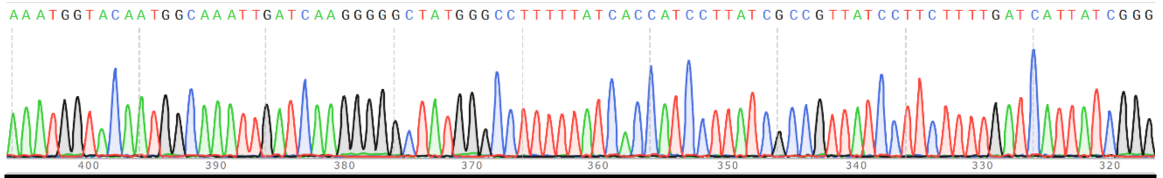

PCR_C3 : aaatggtacaatggcaaattgatcaaggggggctatgggcctttttatcaccatccttatcgccgttatccttcttttgatcattatcggg : 990
R47_C3 : aaatggtacaatggcaaattgatcaaggggggctatgggcctttttatcaccatccttatcgccgttatccttcttttgatcattatcggg : 990
AAATGGTACAATGGCAAATTGATCAAGGGGGGCTATGGGCCTTTTTATCACCATCCTTATCGCCGTTATCCTTCTTTTGATCATTATCGGG

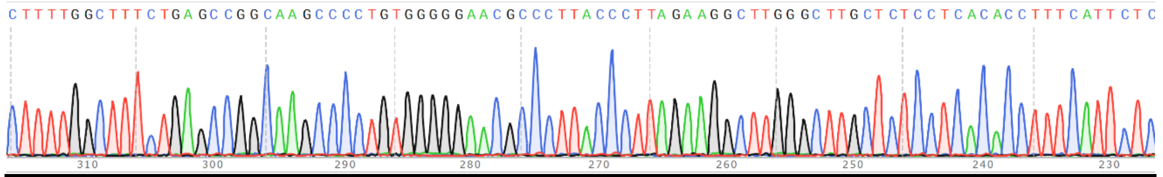

PCR_C3 : ctttggctttctgagccggcaagcccctgtgggggaacgcccttacccttagaaggcttgggcttgctctcctcacacctttcattctc : 1080
R47_C3 : ctttggctttctgagccggcaagcccctgtgggggaacgcccttacccttagaaggcttgggcttgctctcctcacacctttcattctc : 1080
CTTTTGGCTTTCTGAGCCGGCAAGCCCCTGTGGGGGAACGCCCTTACCCTTAGAAGGCTTGGGCTTGCTCTCCTCACACCTTTCATTCTC

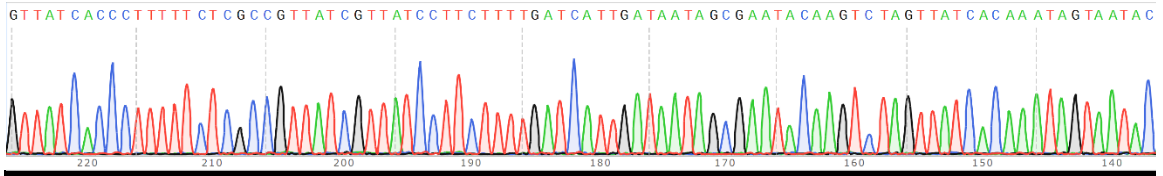

PCR_C3 : gttatcacccttttttctcgccgttatcgttatccttcttttgatcattgataatagcgaatacaagtctagttatcacaaatagtaatac : 1170
R47_C3 : gttatcacccttttttctcgccgttatcgttatccttcttttgatcattgataatagcgaatacaagtctagttatcacaaatagtaatac : 1170
GTTATCACCCTTTTTTCTCGCCGTTATCGTTATCCTTCTTTTGATCATTGATAATAGCGAATACAAGTCTAGTTATCACAAATAGTAATAC

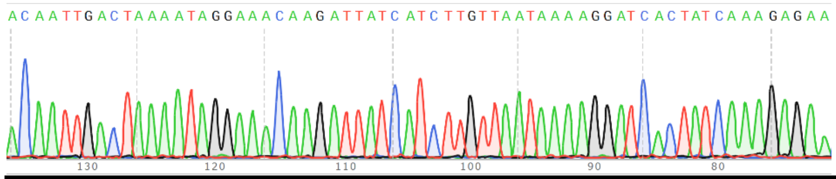

PCR_C3 : acaattgactaaaataggaaacaagattatcatcttgttaataaaaggatcactatcaaagagaa : 1235
R47_C3 : acaattgactaaaataggaaacaagattatcatcttgttaataaaaggatcactatcaaagagaa : 1235
ACAATTGACTAAAATAGGAAACAAGATTATCATCTTGTTAATAAAAGGATCACTATCAAAGAGAA

1 comparison of the sequence obtained from PCR amplification and Sanger sequencing (R47-C4) and the expected sequence.

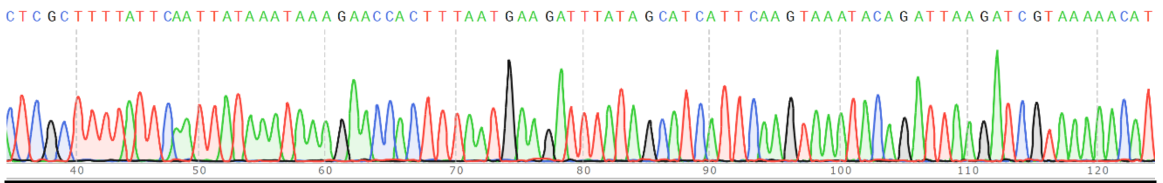

PCR_C4 : ctcgcttttattcaattataaataaagaaccactttaatgaagatttatagcatcattcaagtaaatacagattaagatcgtaaaaacat : 90
R47_C4 : ctcgcttttattcaattataaataaagaaccactttaatgaagatttatagcatcattcaagtaaatacagattaagatcgtaaaaacat : 90
CTCGCTTTTATTCAATTATAAATAAAGAACCACTTTAATGAAGATTTATAGCATCATTCAAGTAAATACAGATTAAGATCGTAAAAACAT

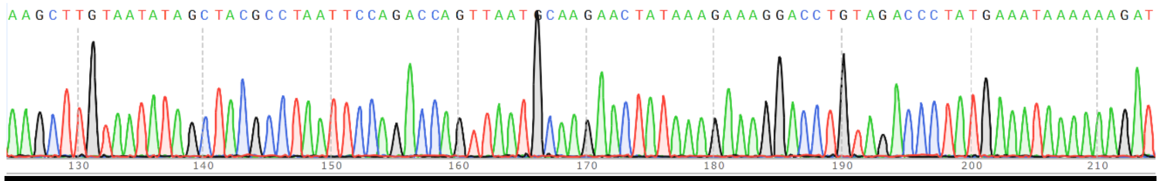

PCR_C4 : aagcttgtaatatagctacgcctaattccagaccagttaatgcaagaactataaagaaaggacctgtagaccctatgaaataaaaaagat : 180

R47_C4 : aagcttgtaatatagctacgcctaattccagaccagttaatgcaagaactataaagaaaggacctgtagaccctatgaaataaaaaagat : 180
AAGCTTGTAATATAGCTACGCCTAATTCCAGACCAGTTAATGCAAGAACTATAAAGAAAGGACCTGTAGACCCTATGAAATAAAAAAGAT

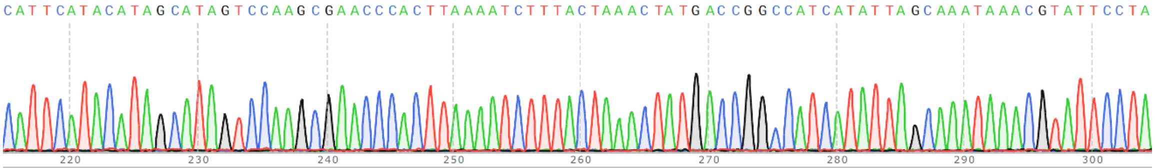

PCR_C4 : cattcatacatagcatagtccaagcgaacccacttaaaatctttactaaactatgaccggccatcatattagcaaataaacgtattccta : 270
R47_C4 : cattcatacatagcatagtccaagcgaacccacttaaaatctttactaaactatgaccggccatcatattagcaaataaacgtattccta : 270
CATTCATACATAGCATAGTCCAAGCGAACCCACTTAAAATCTTTACTAAACTATGACCGGCCATCATATTAGCAAATAAACGTATTCCTA

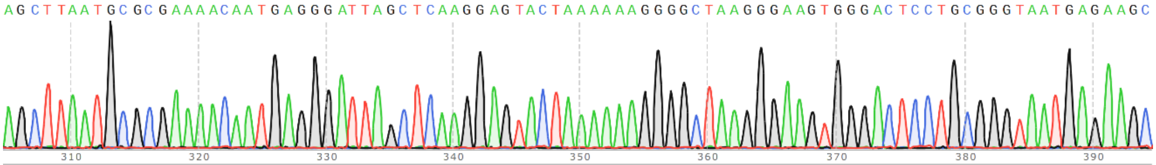

PCR_C4 : agcttaatgcgcgaaaacaatgagggattagctcaaggagtactaaaaaaggggctaagggaagtgggactcctgcgggtaatgagaagc : 360
R47_C4 : agcttaatgcgcgaaaacaatgagggattagctcaaggagtactaaaaaaggggctaagggaagtgggactcctgcgggtaatgagaagc : 360
AGCTTAATGCGCGAAAACAATGAGGGATTAGCTCAAGGAGTACTAAAAAAGGGGCTAAGGGAAGTGGGACTCCTGCGGGTAATGAGAAGC

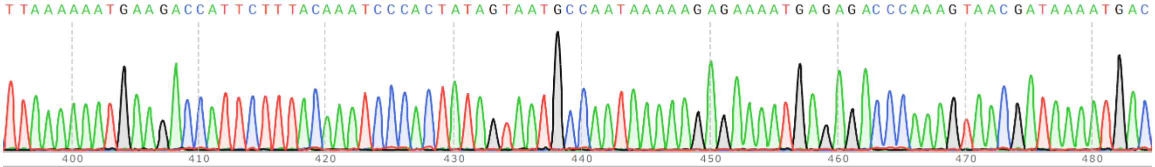

PCR_C4 : ttaaaaaatgaagaccattctttacaaatcccactatagtaatgccaataaaaagagaaaatgagagacccaaagtaacgataaaatgac : 450
R47_C4 : ttaaaaaatgaagaccattctttacaaatcccactatagtaatgccaataaaaagagaaaatgagagacccaaagtaacgataaaatgac : 450
TTAAAAAATGAAGACCATTCTTTACAAATCCCACTATAGTAATGCCAATAAAAAGAGAAAATGAGAGACCCAAAGTAACGATAAAATGAC

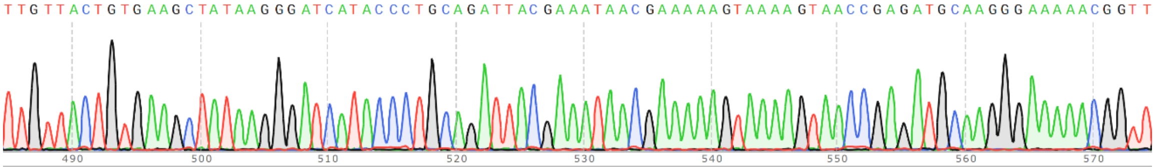

PCR_C4 : ttgttactgtgaagctataagggatcataccctgcagattacgaaataacgaaaaagtaaaagtaaccgagatgcaagggaaaaacggtt : 540
R47_C4 : ttgttactgtgaagctataagggatcataccctgcagattacgaaataacgaaaaagtaaaagtaaccgagatgcaagggaaaaacggtt : 540
TTGTTACTGTGAAGCTATAAGGGATCATACCCTGCAGATTACGAAATAACGAAAAAGTAAAAGTAACCGAGATGCAAGGGAAAAACGGTT

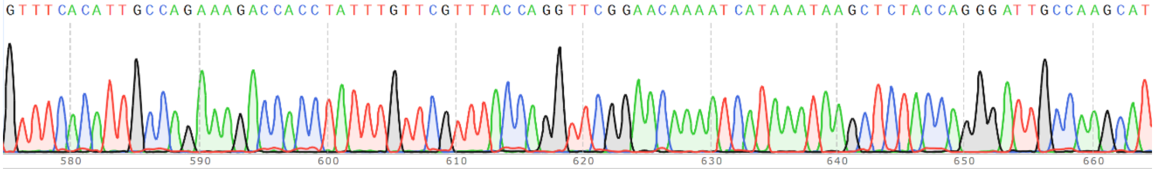

PCR_C4 : gtttcacattgccagaaagaccacctatttgttcgtttaccaggttcggaacaaaatcataaataagctctaccagggattgccaagcat : 630
R47_C4 : gtttcacattgccagaaagaccacctatttgttcgtttaccaggttcggaacaaaatcataaataagctctaccagggattgccaagcat : 630
GTTTCACATTGCCAGAAAGACCACCTATTTGTTCGTTTACCAGGTTCGGAACAAAATCATAAATAAGCTCTACCAGGGATTGCCAAGCAT

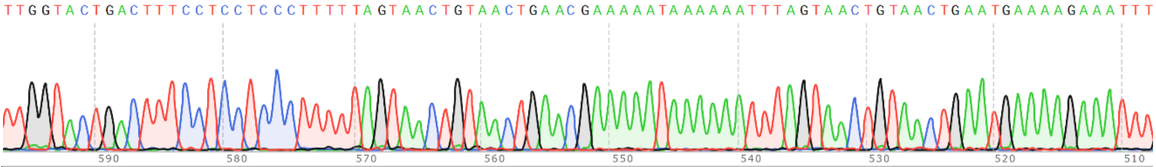

PCR_C4 : ttggtactgactttcctcctcccttttttagtaactgtaactgaacgaaaaaataaaaaatttagtaactgtaactgaatgaaaagaaattt : 720
R47_C4 : ttggtactgactttcctcctcccttttttagtaactgtaactgaacgaaaaaataaaaaatttagtaactgtaactgaatgaaaagaaattt : 720
TTGGTACTGACTTTCCTCCTCCCTTTTTTAGTAACTGTAACTGAACGAAAAAATAAAAAATTTAGTAACTGTAACTGAATGAAAAGAAATTT

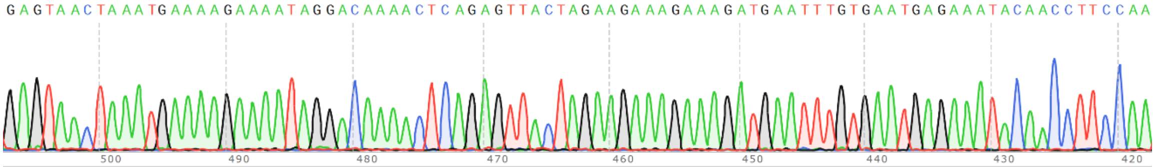

PCR_C4 : gagtaactaaatgaaaagaaaataggacaaaactcagagttactagaagaaagaaagatgaatttgtgaatgagaaatacaaccttccaa : 810
R47_C4 : gagtaactaaatgaaaagaaaataggacaaaactcagagttactagaagaaagaaagatgaatttgtgaatgagaaatacaaccttccaa : 810
GAGTAACTAAATGAAAAGAAAATAGGACAAAACTCAGAGTTACTAGAAGAAAGAAAGATGAATTTGTGAATGAGAAATACAACCTTCCAA

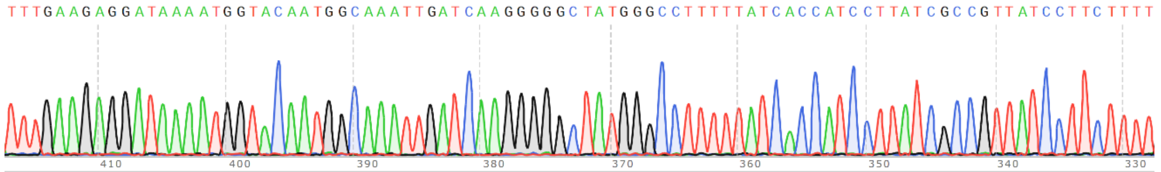

PCR_C4 : tttgaagaggataaaatggtacaatggcaaattgatcaagggggctatgggcctttttatcaccatccttatcgccgttatccttctttt : 900
R47_C4 : tttgaagaggataaaatggtacaatggcaaattgatcaagggggctatgggcctttttatcaccatccttatcgccgttatccttctttt : 900
TTTGAAGAGGATAAAATGGTACAATGGCAAATTGATCAAGGGGGCTATGGGCCTTTTTATCACCATCCTTATCGCCGTTATCCTTCTTTT

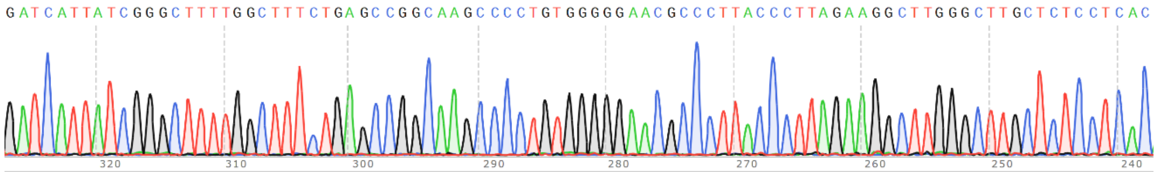

PCR_C4 : gatcattatcgggcttttggctttctgagccggcaagcccctgtgggggaacgcccttacccttagaaggcttgggcttgctctcctcac : 990
R47_C4 : gatcattatcgggcttttggctttctgagccggcaagcccctgtgggggaacgcccttacccttagaaggcttgggcttgctctcctcac : 990
GATCATTATCGGGCTTTTGGCTTTCTGAGCCGGCAAGCCCCTGTGGGGGAACGCCCTTACCCTTAGAAGGCTTGGGCTTGCTCTCCTCAC

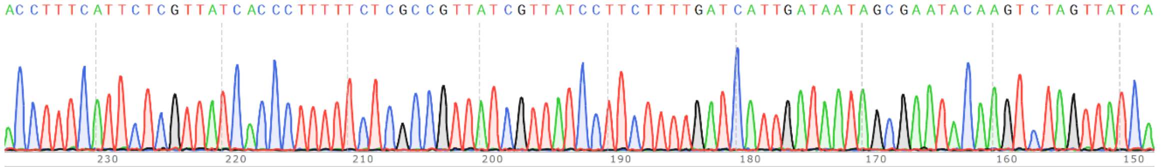

PCR_C4 : acctttcattctcgttatcacccttttttctcgccgttatcgttatccttcttttgatcattgataatagcgaatacaagtctagttatca : 1080
R47_C4 : acctttcattctcgttatcacccttttttctcgccgttatcgttatccttcttttgatcattgataatagcgaatacaagtctagttatca : 1080
ACCTTTCATTCTCGTTATCACCCTTTTTCTCGCCGTTATCGTTATCCTTCTTTTGATCATTGATAATAGCGAATACAAGTCTAGTTATCA

Figure S4 Circular map showing the distribution of MTPTs on MC1 and MC2 of *S. officinalis* mitogenome. The light and dark blue lines of the outer circle represent MC1 and MC2 of the *S. officinalis* mitogenome, respectively. The green line of the outer circle is the plastome of *S. officinalis*. The blue and yellow links in the inner circle represent MTPTs on MC1 and MC2 of the *S. officinalis* mitogenome, respectively. The plastid-derived PCGs and tRNA are marked in the plastome and mitogenome, respectively.

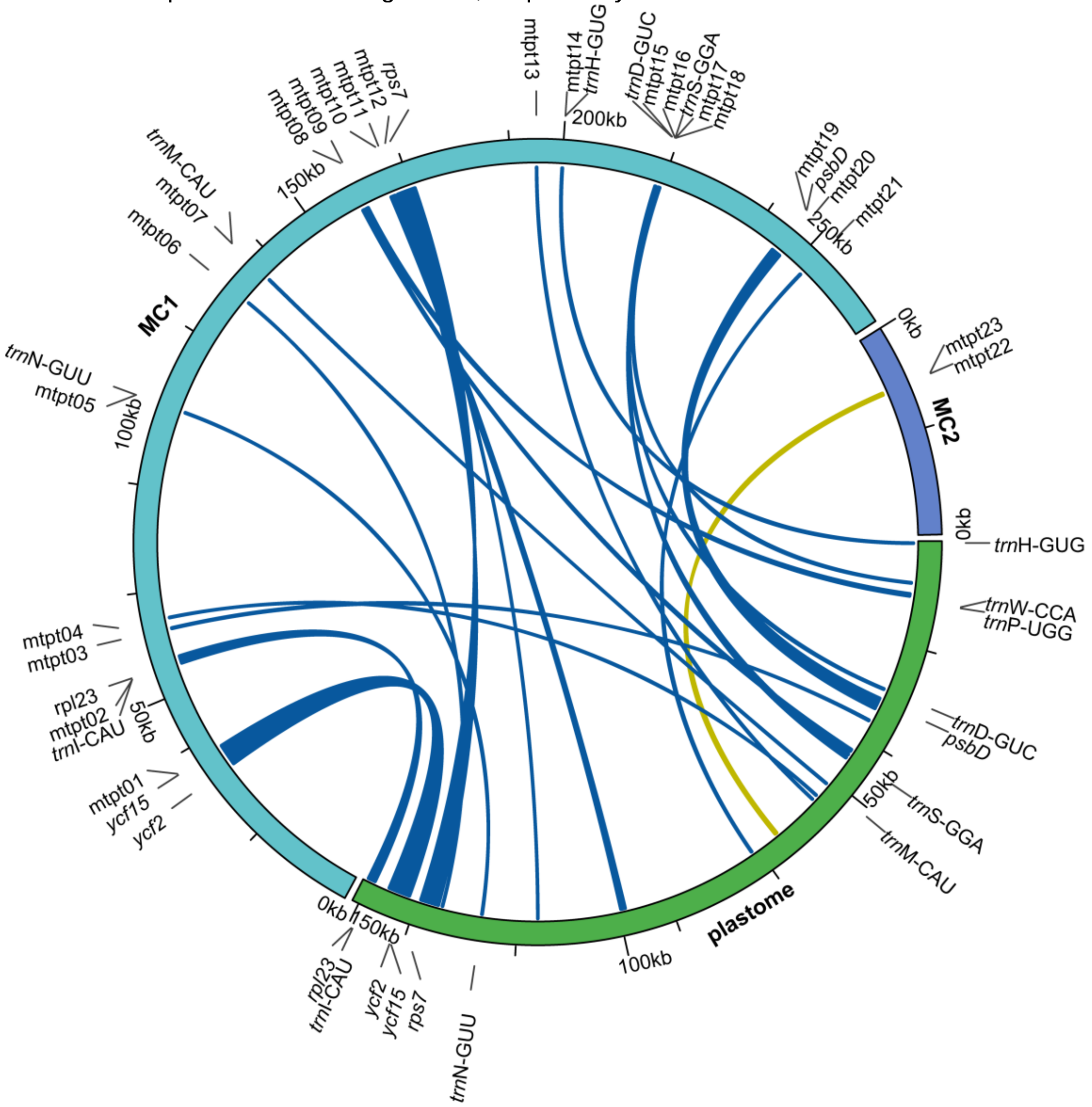

Figure S5 Alignment of the Nanopore long reads to the MTPTs and their flanking sequences in the mitogenome of *S. officinalis*. Panels a-w show the alignment of the Nanopore long reads to the 21 MTPT sequences on MC1 (mtpt01 to mtpt21) and 2 MTPT sequences on MC2 (mtpt22 to mtpt23). The figures were generated using IGV. The MTPT sequence was shown on the top of each figure with its coordinates. The coverage depth was shown with a blue bar chart. The reads were represented with gray lines. The regions in gray represent a match. The purple regions represent small indels. Regions shown with other colors represent mismatched bases. The MTPT sequences are indicated with red lines that have arrowheads at each end. The flanking sequences that are 2000 bp long are indicated with red lines without arrowheads.

a alignment of Nanopore long reads to mtpt01 and its flanking sequences.

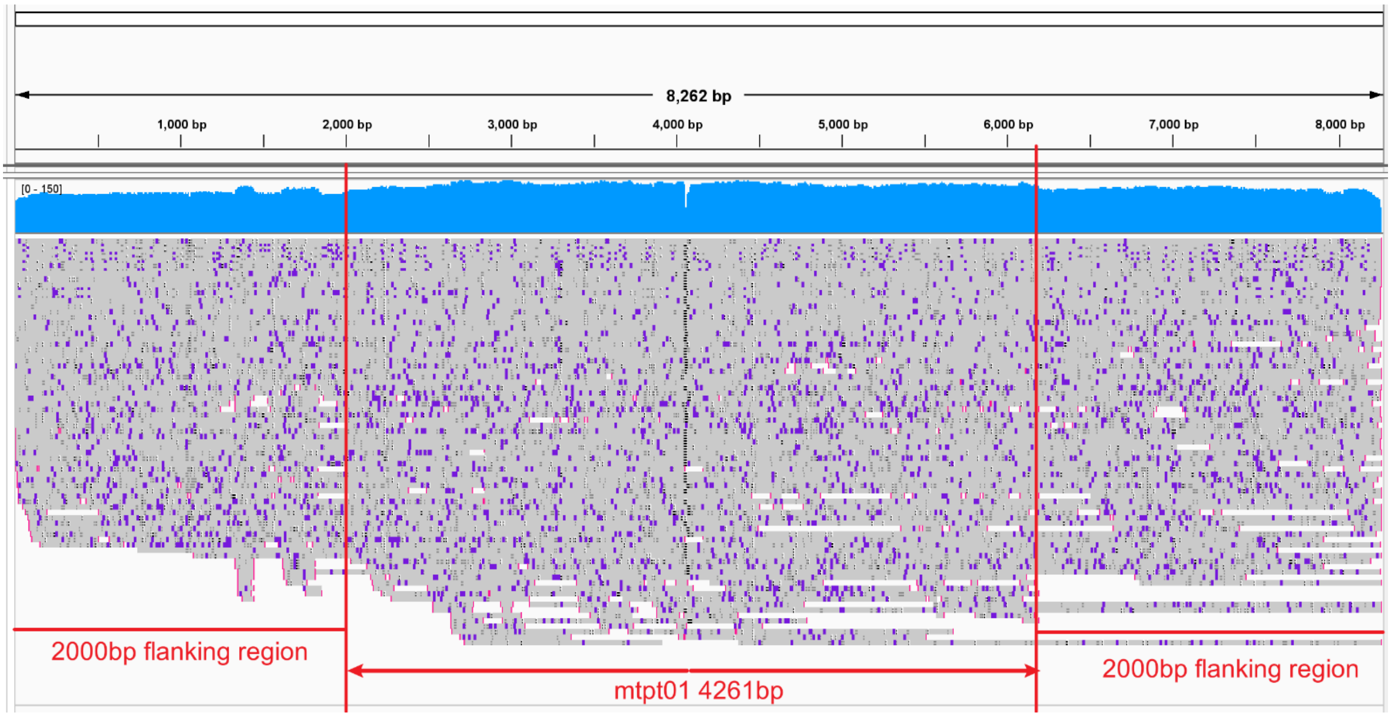

b alignment of Nanopore long reads to mtpt02 and its flanking sequences.

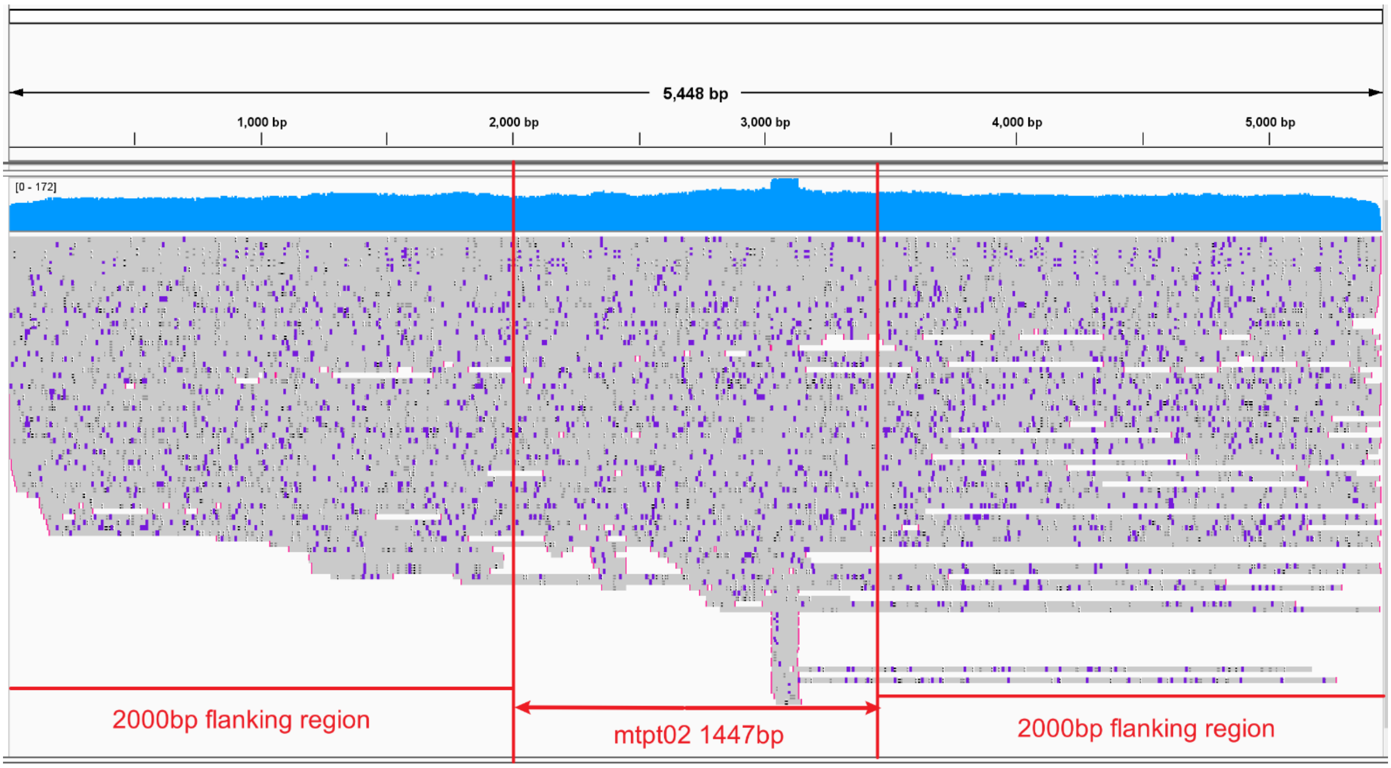

c alignment of Nanopore long reads to mtpt03 and its flanking sequences.

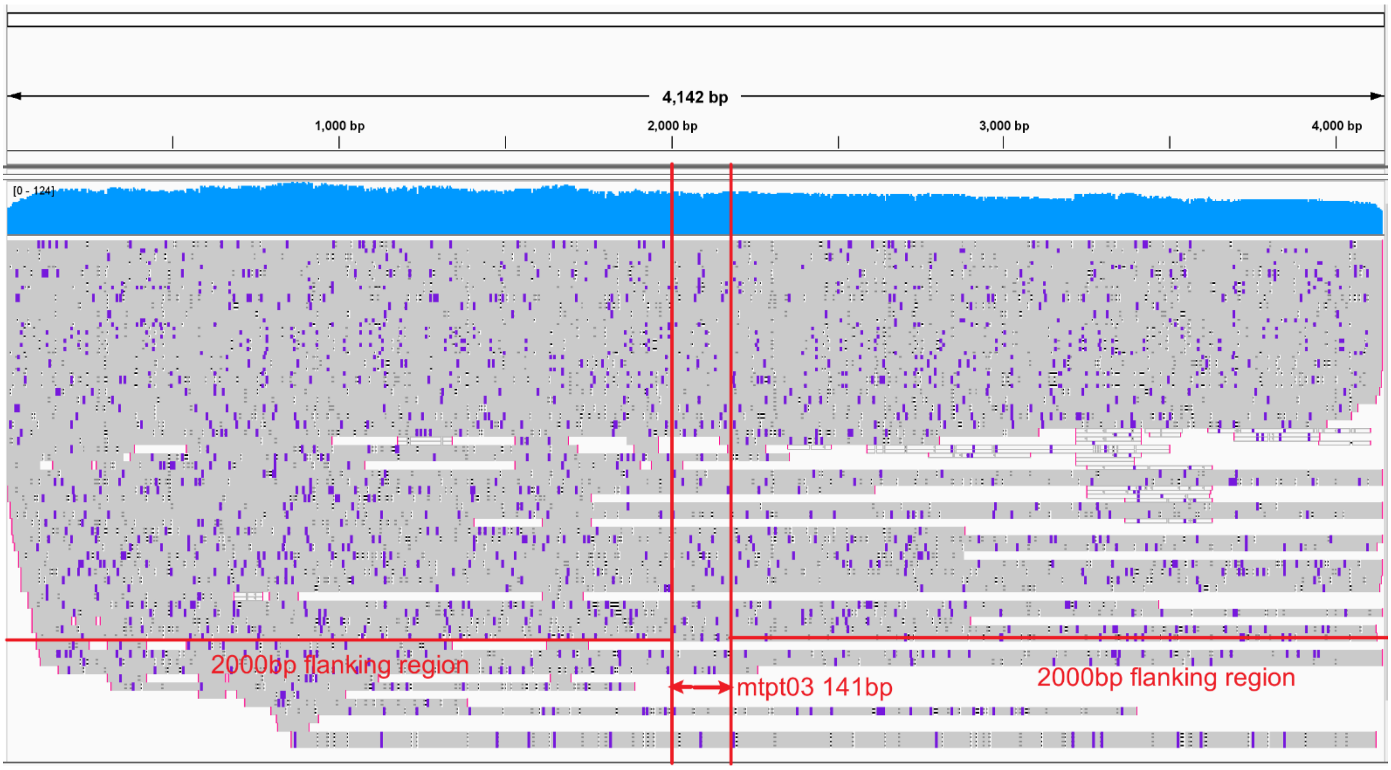

d alignment of Nanopore long reads to mtpt04 and its flanking sequences.

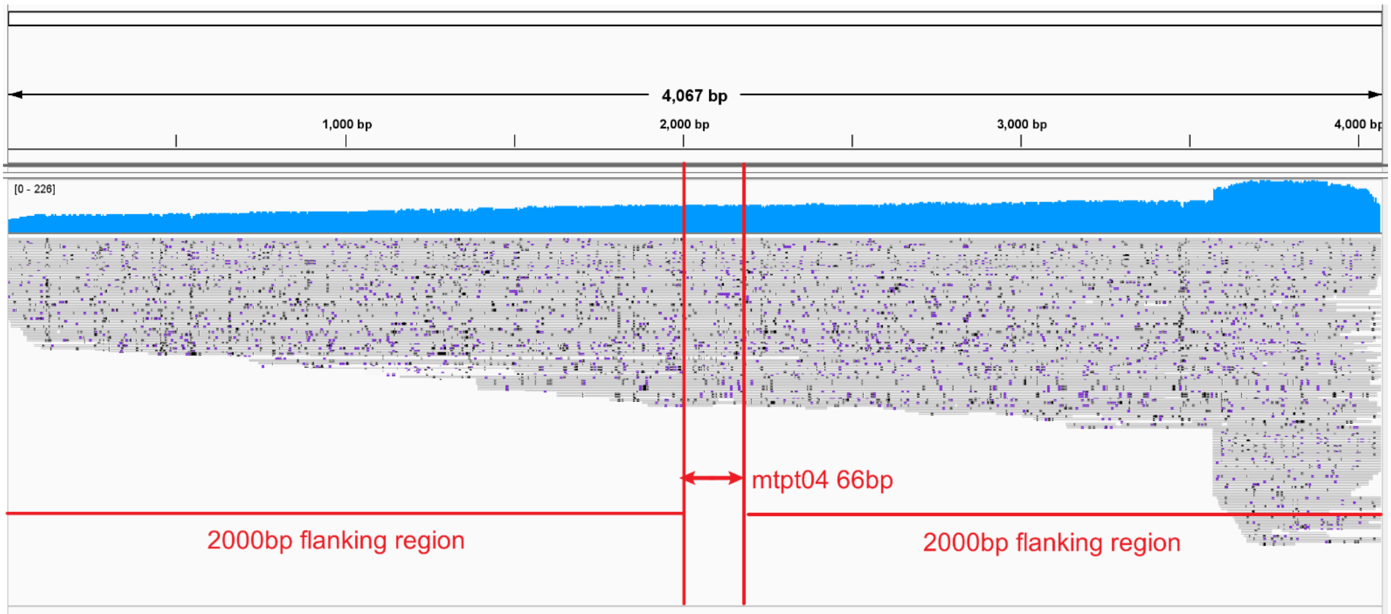

e alignment of Nanopore long reads to mtpt05 and its flanking sequences.

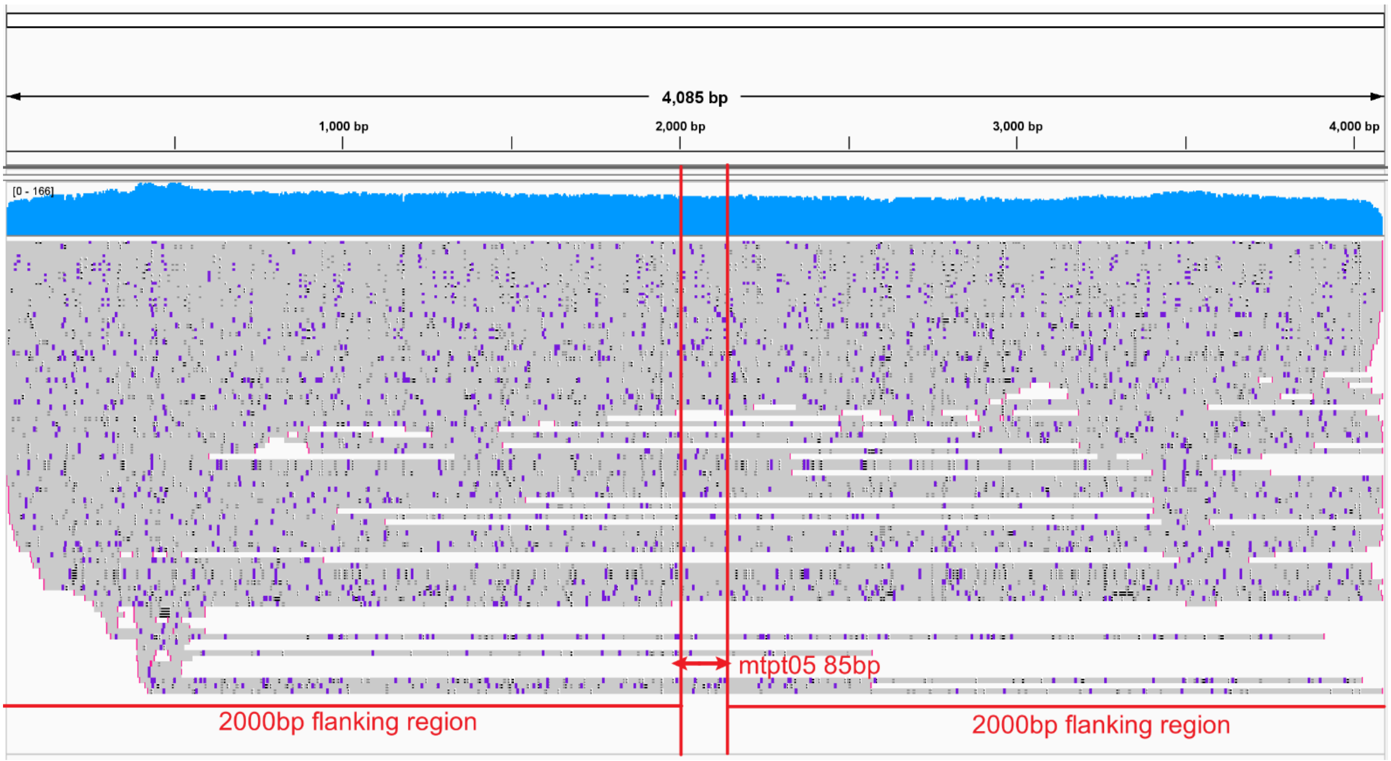

f alignment of Nanopore long reads to mtpt06 and its flanking sequences.

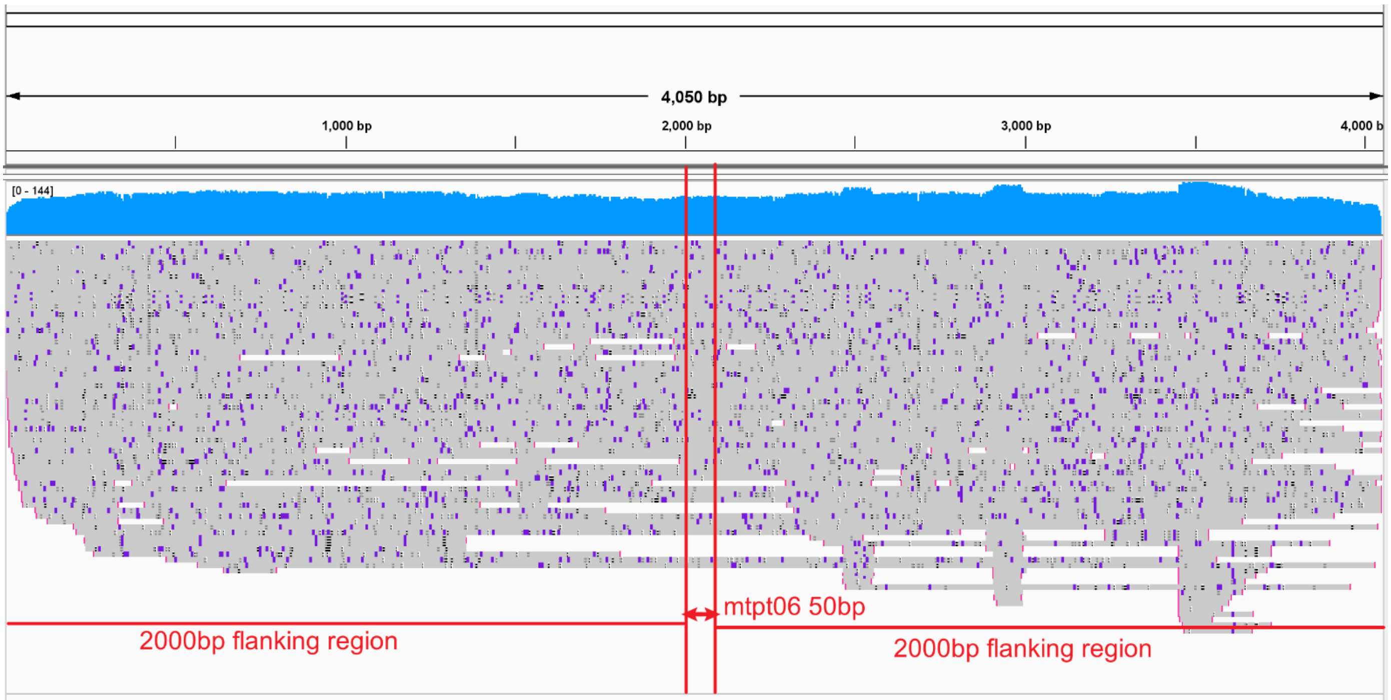

g alignment of Nanopore long reads to mtpt07 and its flanking sequences.

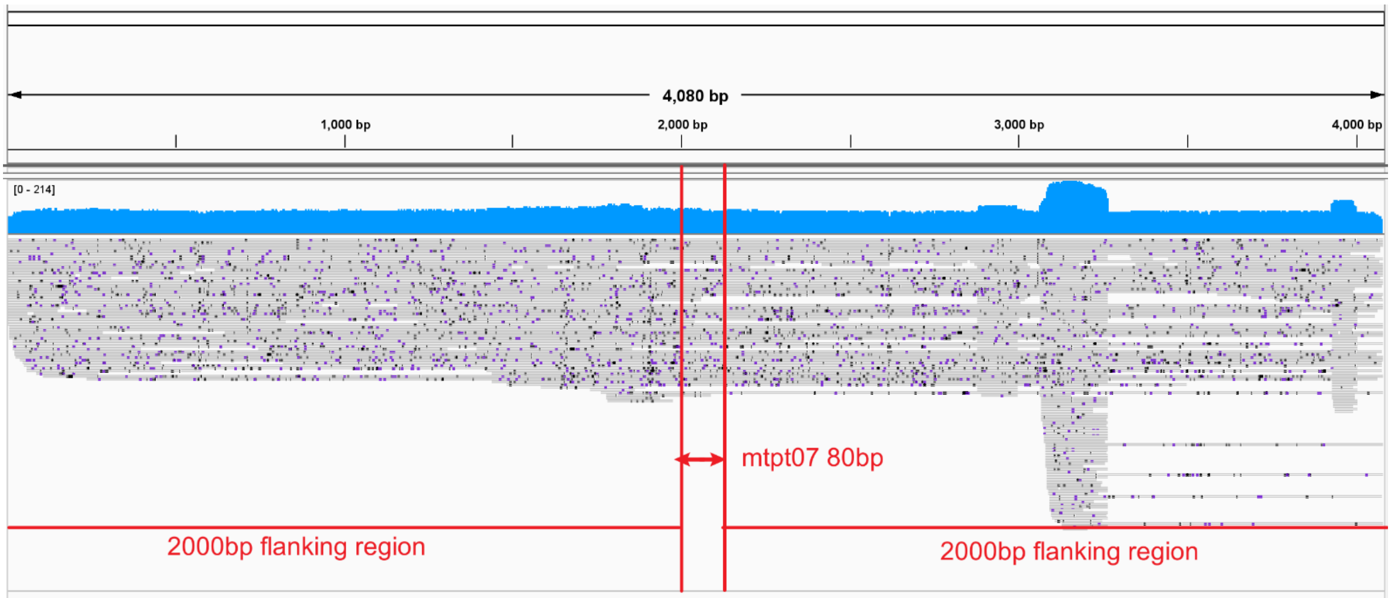

h alignment of Nanopore long reads to mtpt08 and its flanking sequences.

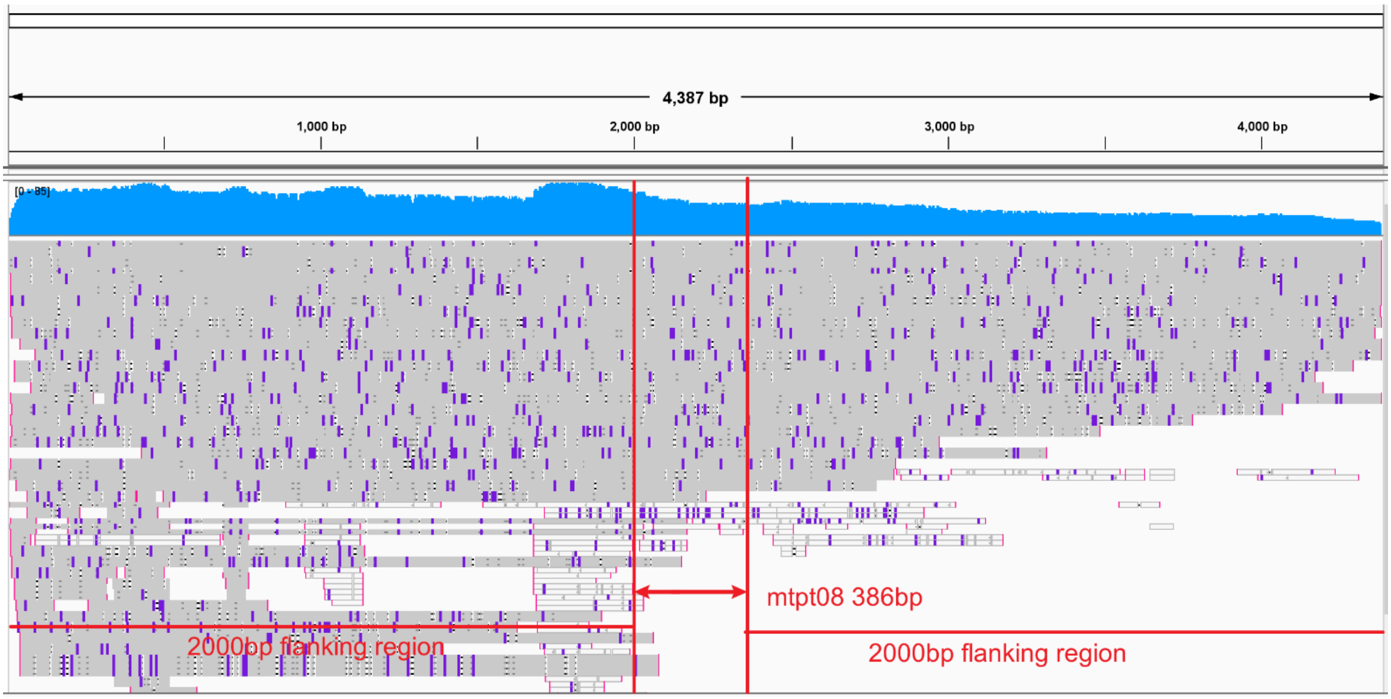

i alignment of Nanopore long reads to mtpt09 and its flanking sequences.

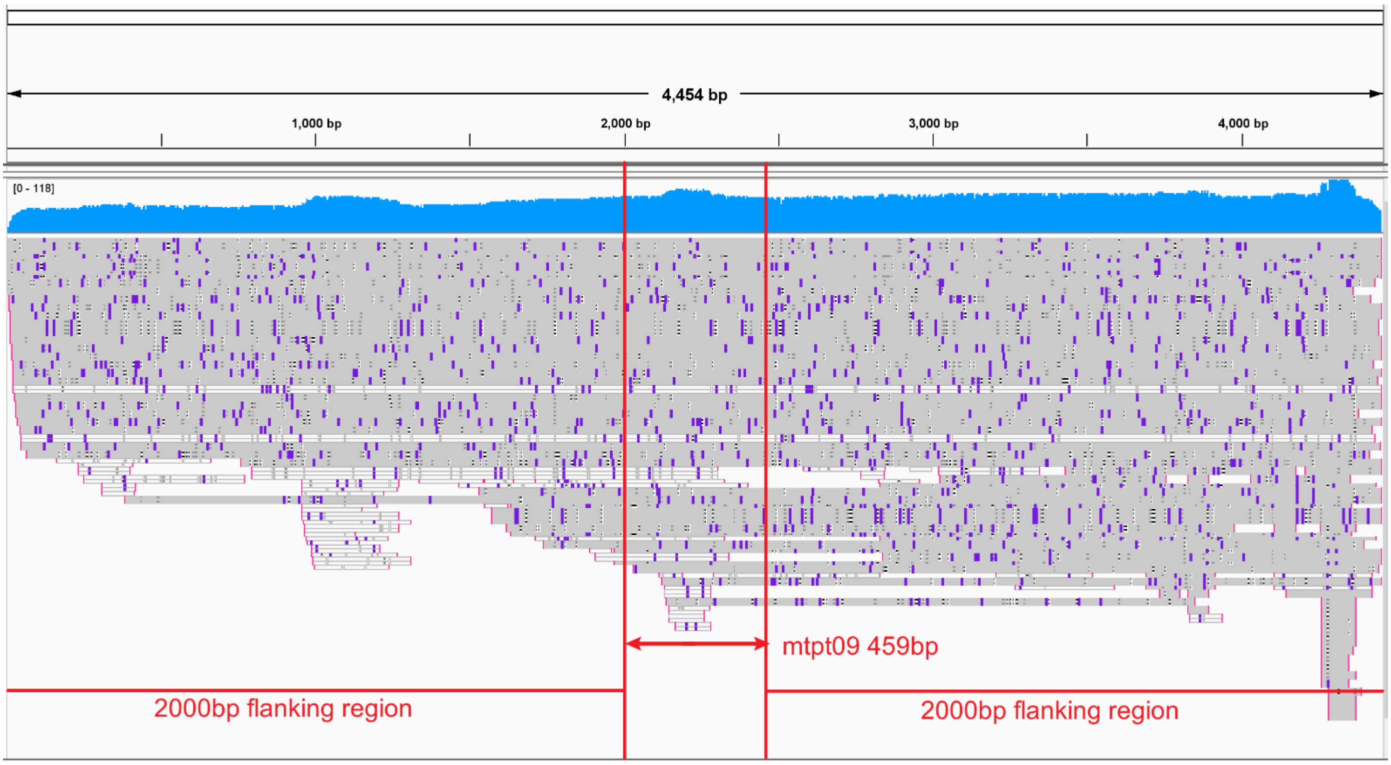

j alignment of Nanopore long reads to mtpt10 and its flanking sequences.

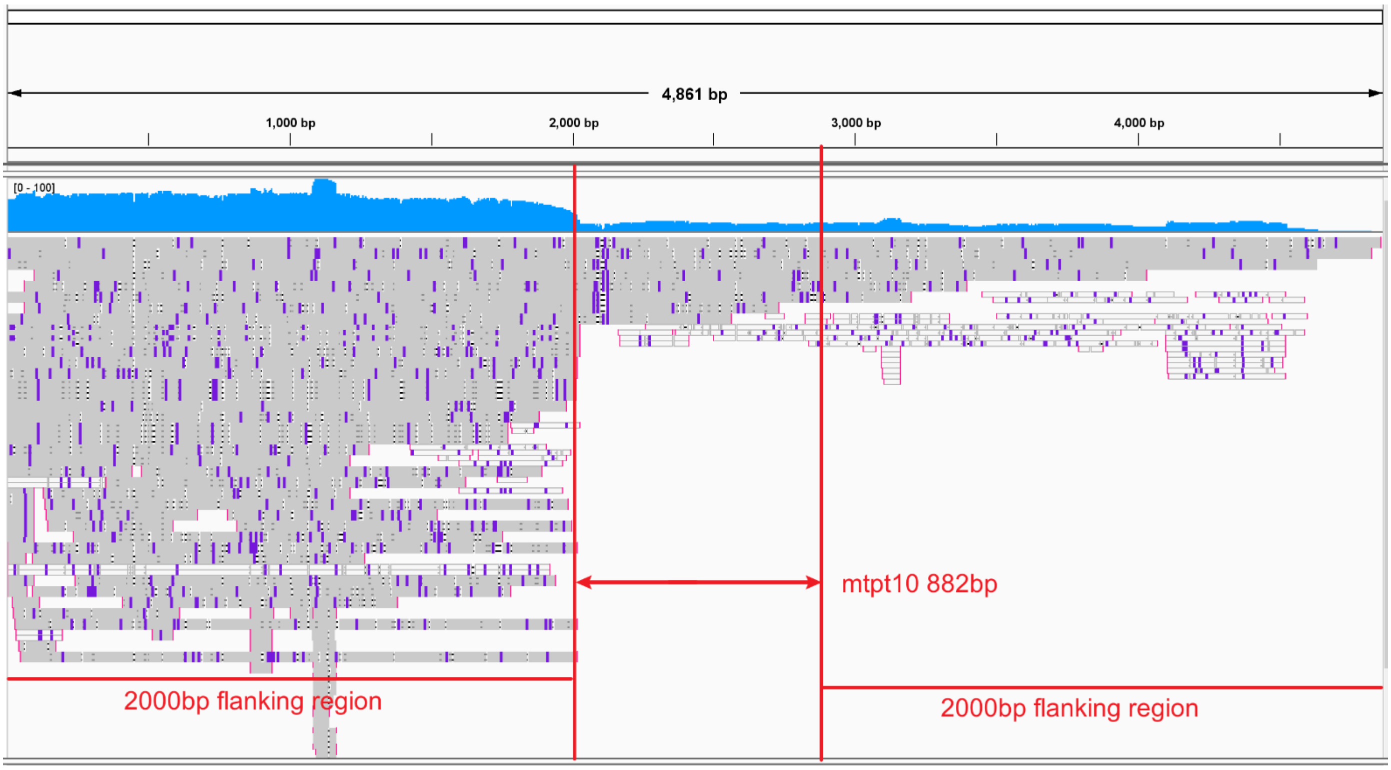

k alignment of Nanopore long reads to mtpt11 and its flanking sequences.

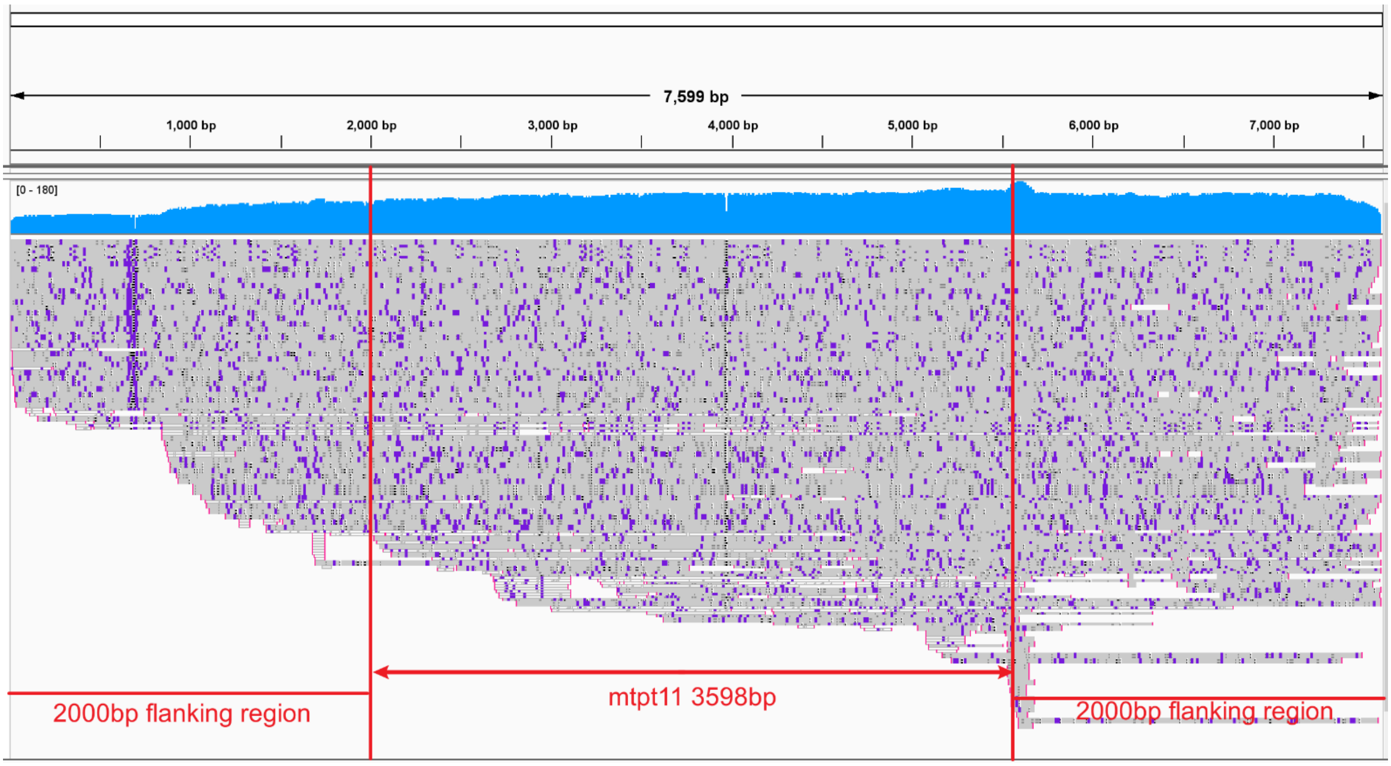

I alignment of Nanopore long reads to mtpt12 and its flanking sequences.

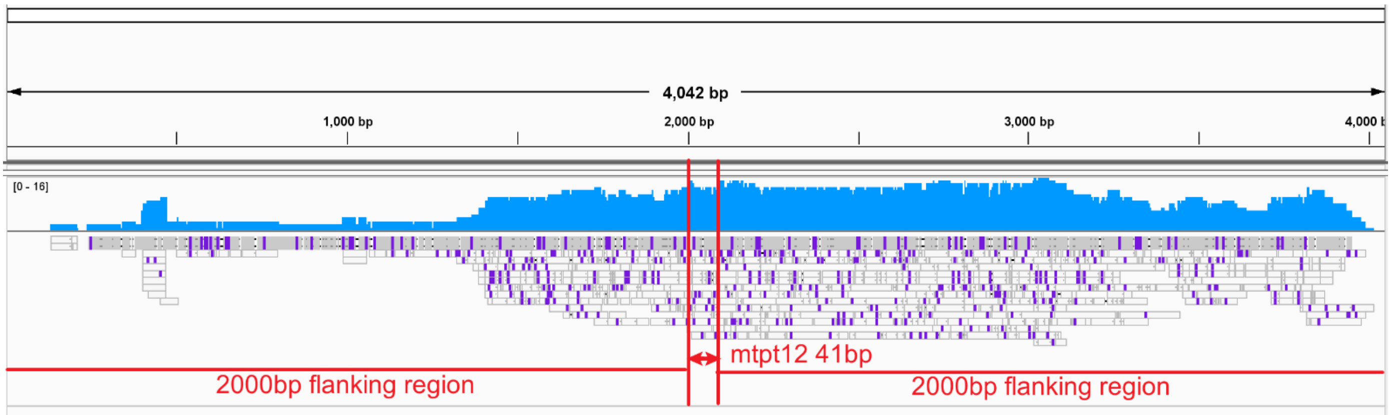

m alignment of Nanopore long reads to mtpt13 and its flanking sequences.

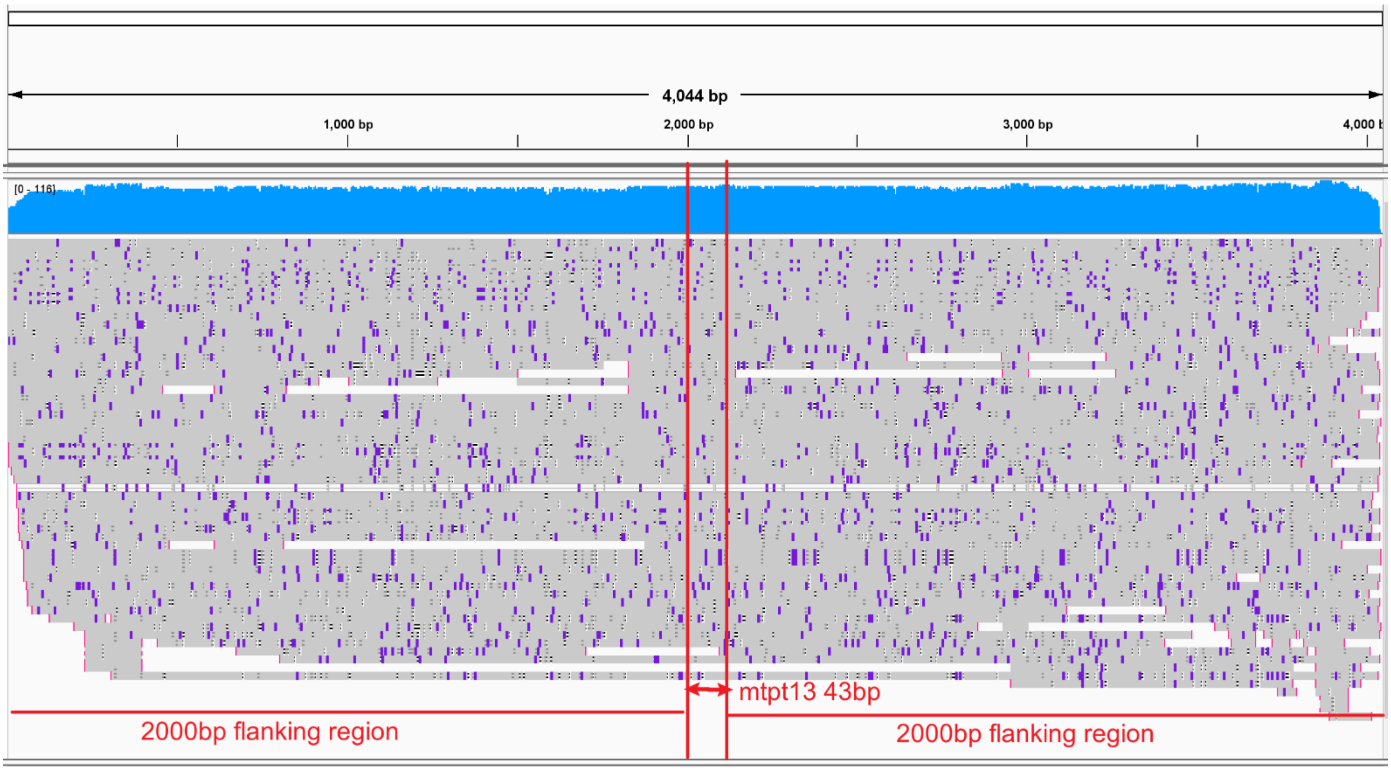

n alignment of Nanopore long reads to mtpt14 and its flanking sequences.

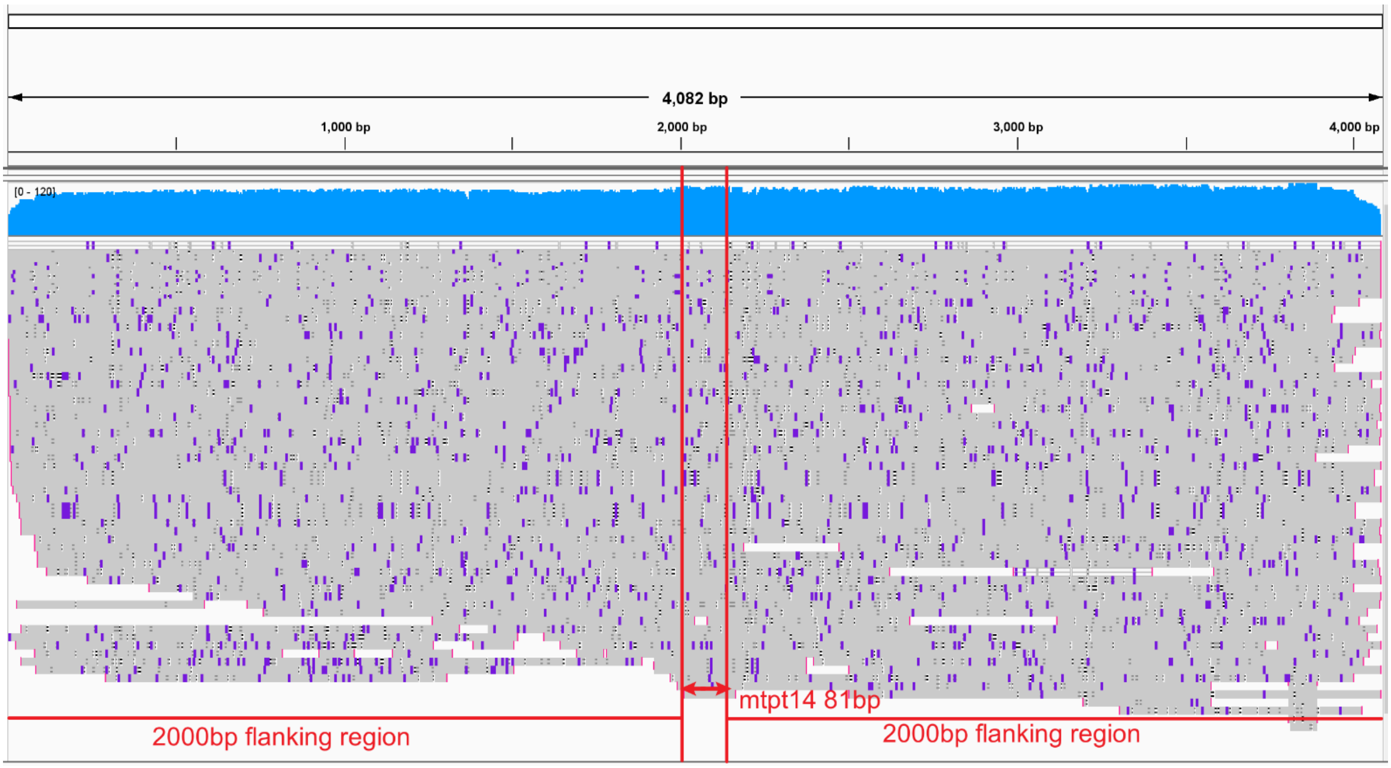

o alignment of Nanopore long reads to mtpt15 and its flanking sequences.

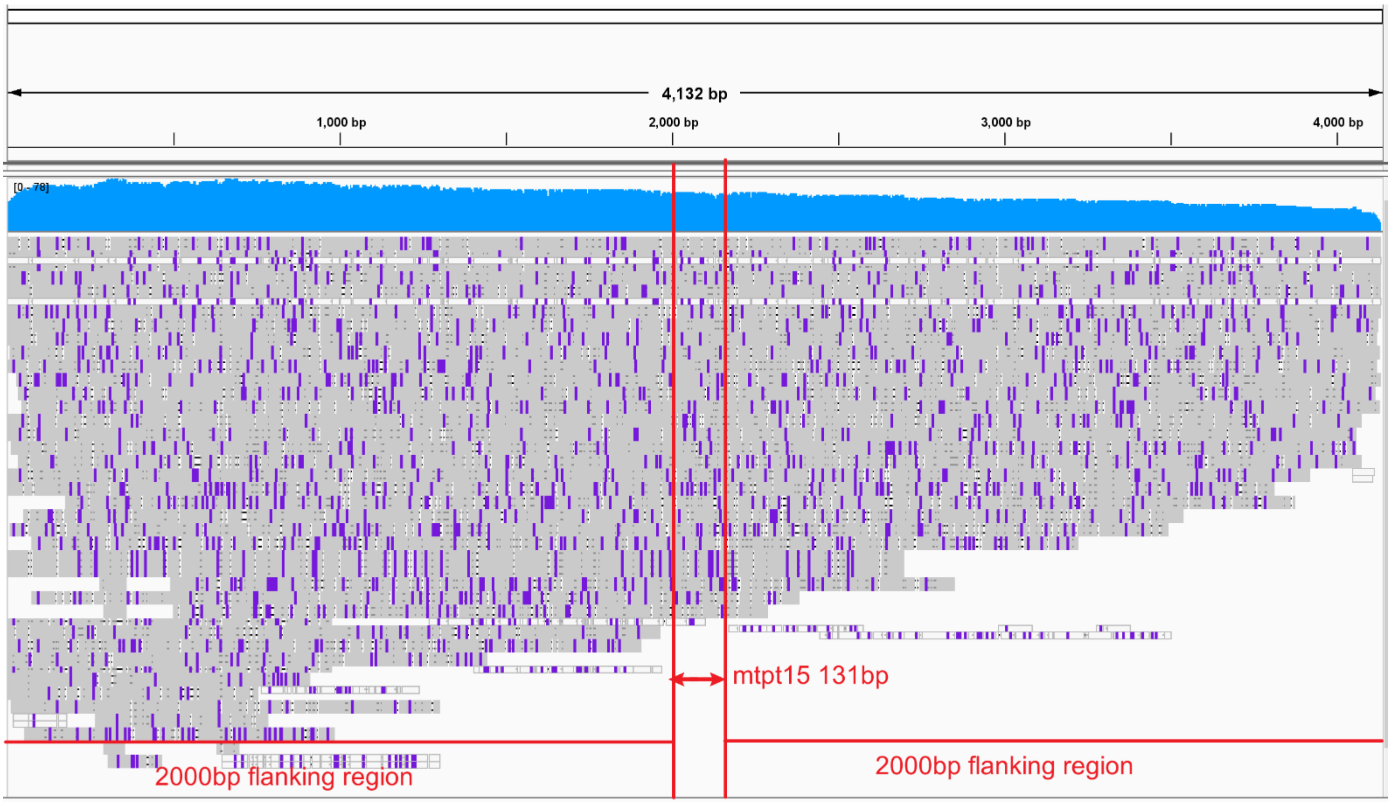

p alignment of Nanopore long reads to mtpt16 and its flanking sequences.

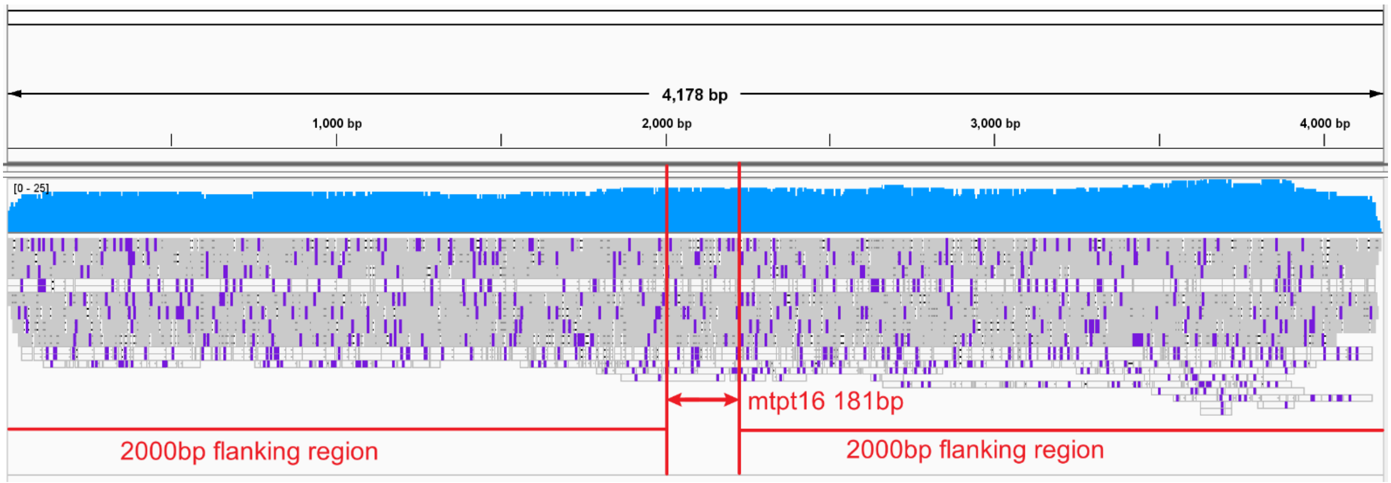

q alignment of Nanopore long reads to mtpt17 and its flanking sequences.

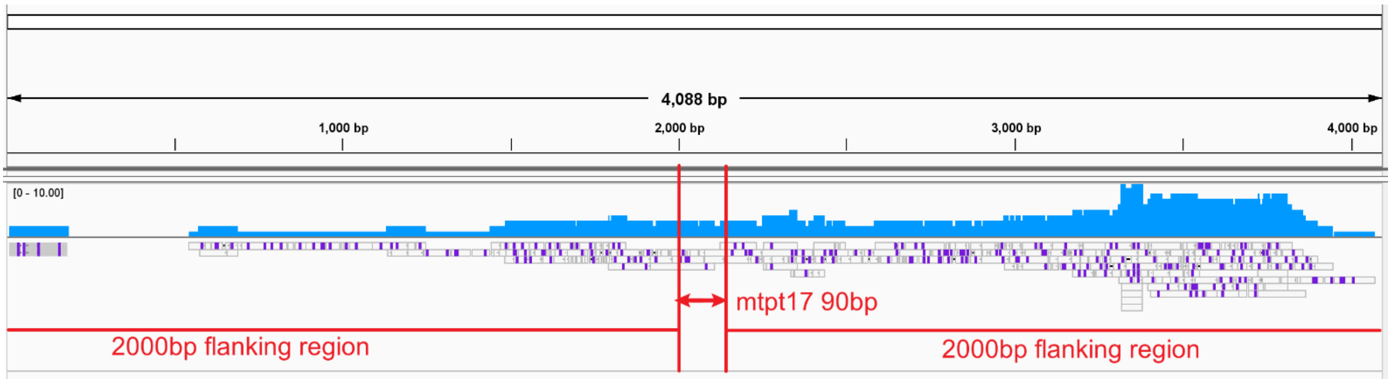

r alignment of Nanopore long reads to mtpt18 and its flanking sequences.

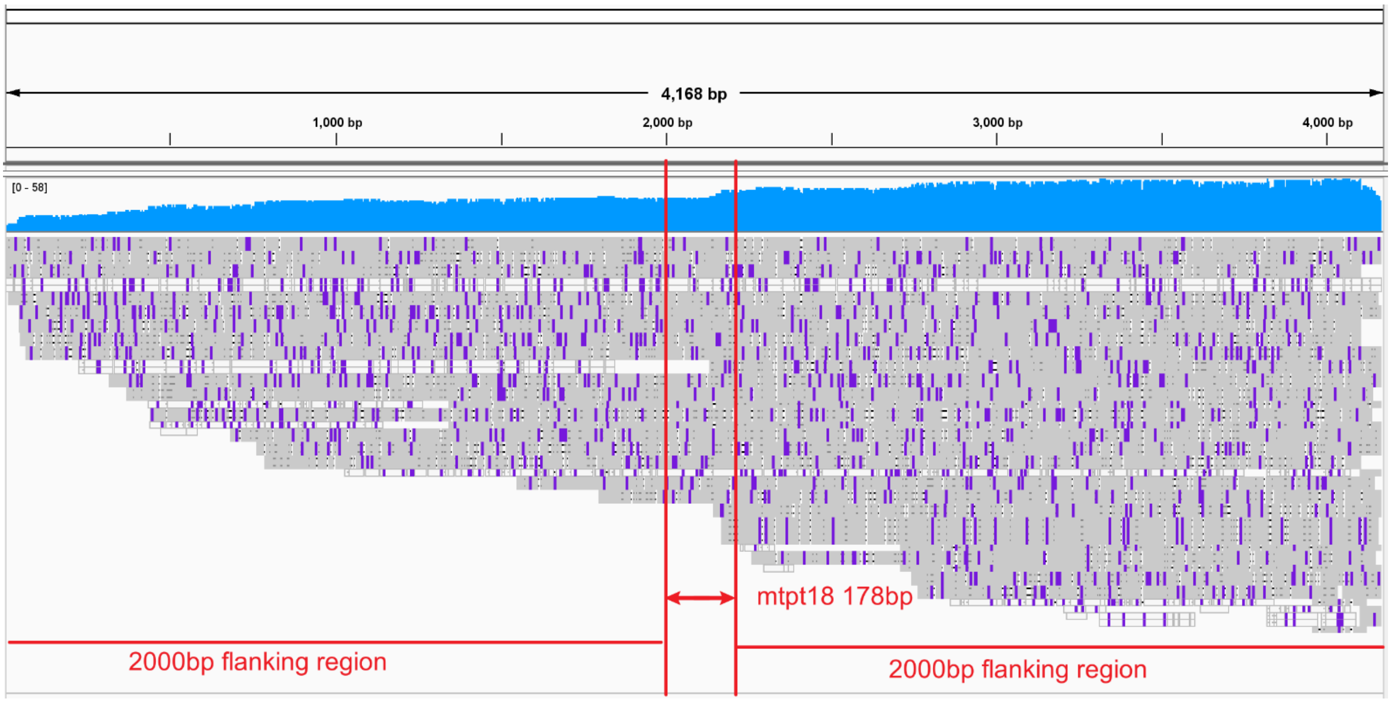

s alignment of Nanopore long reads to mtpt19 and its flanking sequences.

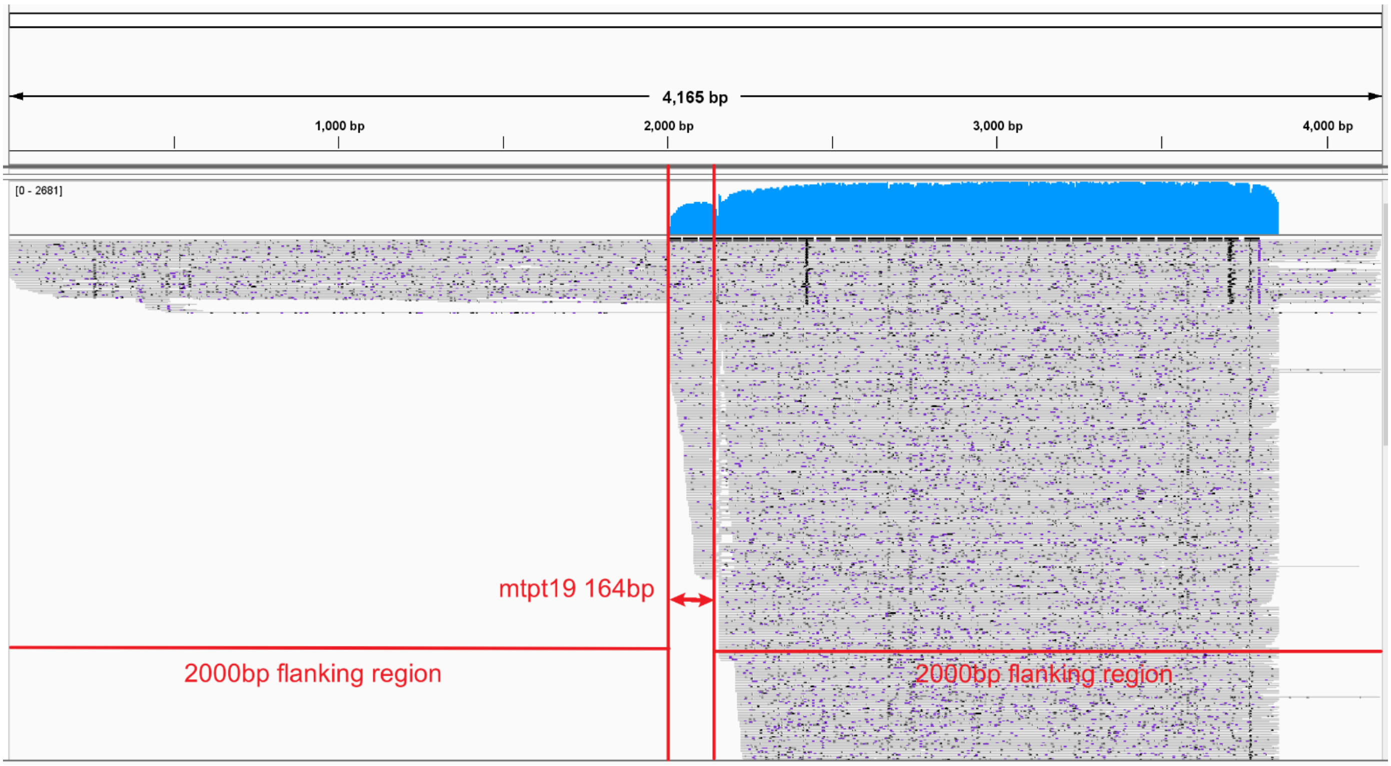

t alignment of Nanopore long reads to mtpt20 and its flanking sequences.

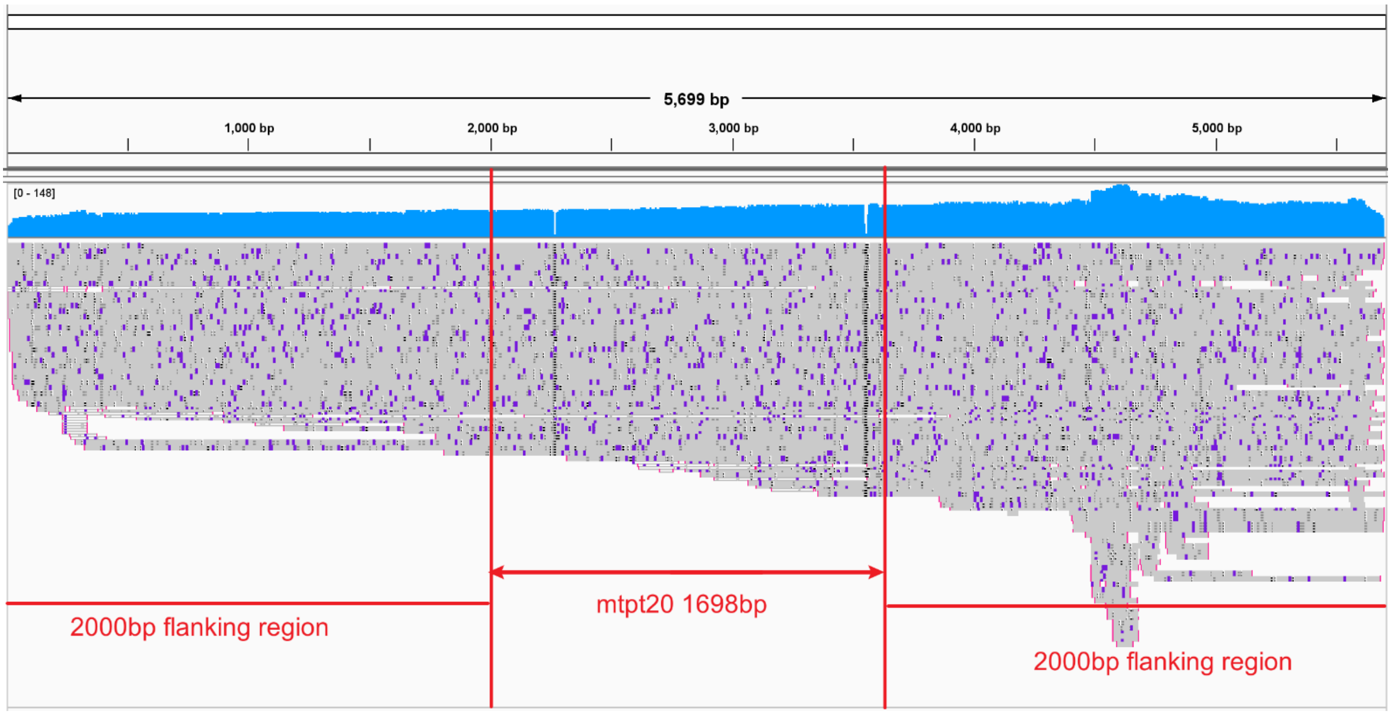

u alignment of Nanopore long reads to mtpt21 and its flanking sequences.

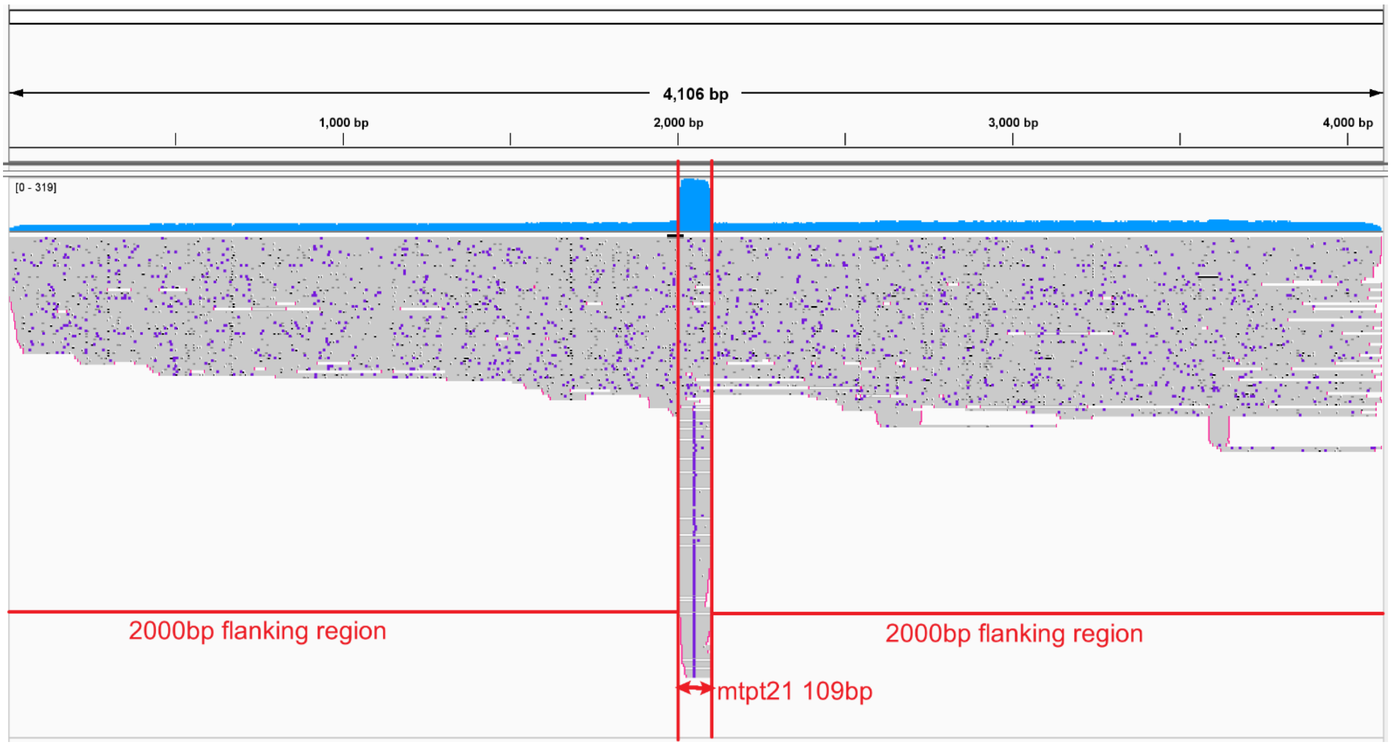

v alignment of Nanopore long reads to mtpt22 and its flanking sequences.

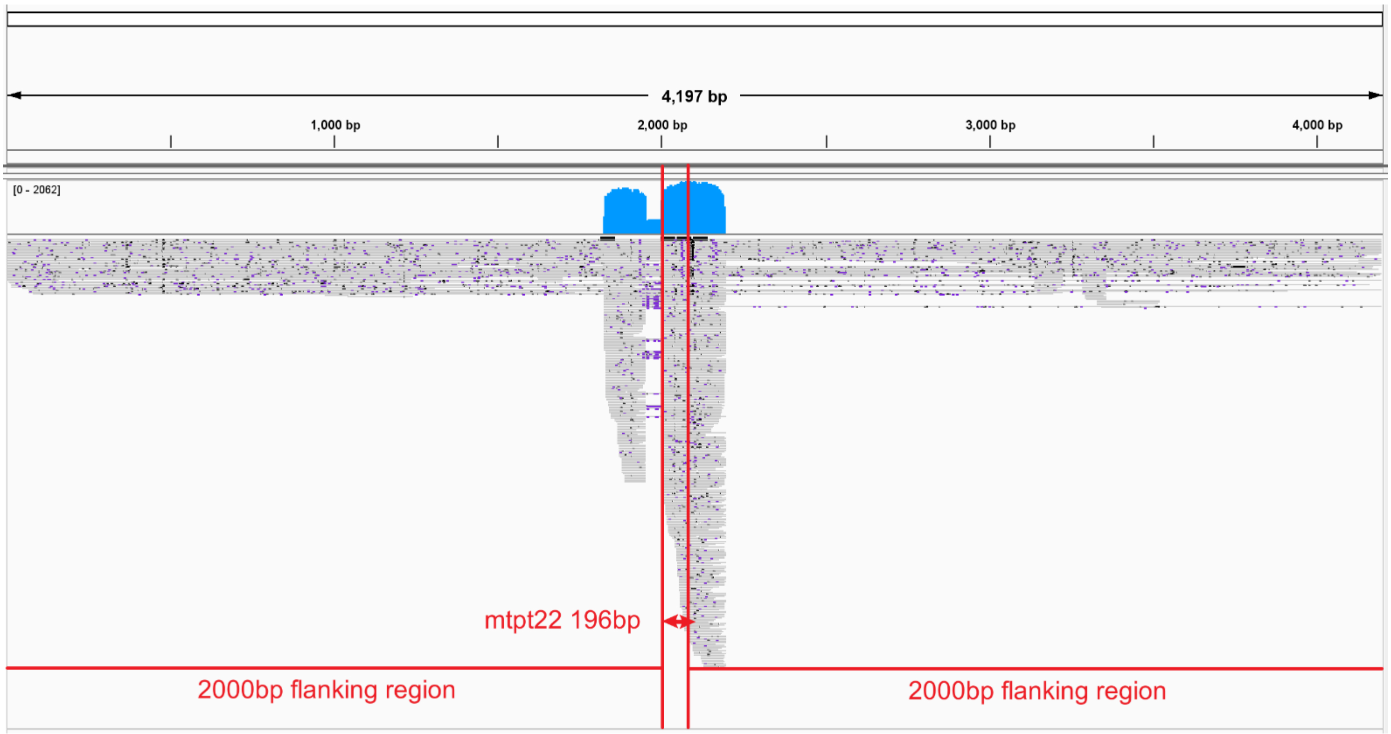

w alignment of Nanopore long reads to mtpt23 and its flanking sequences.

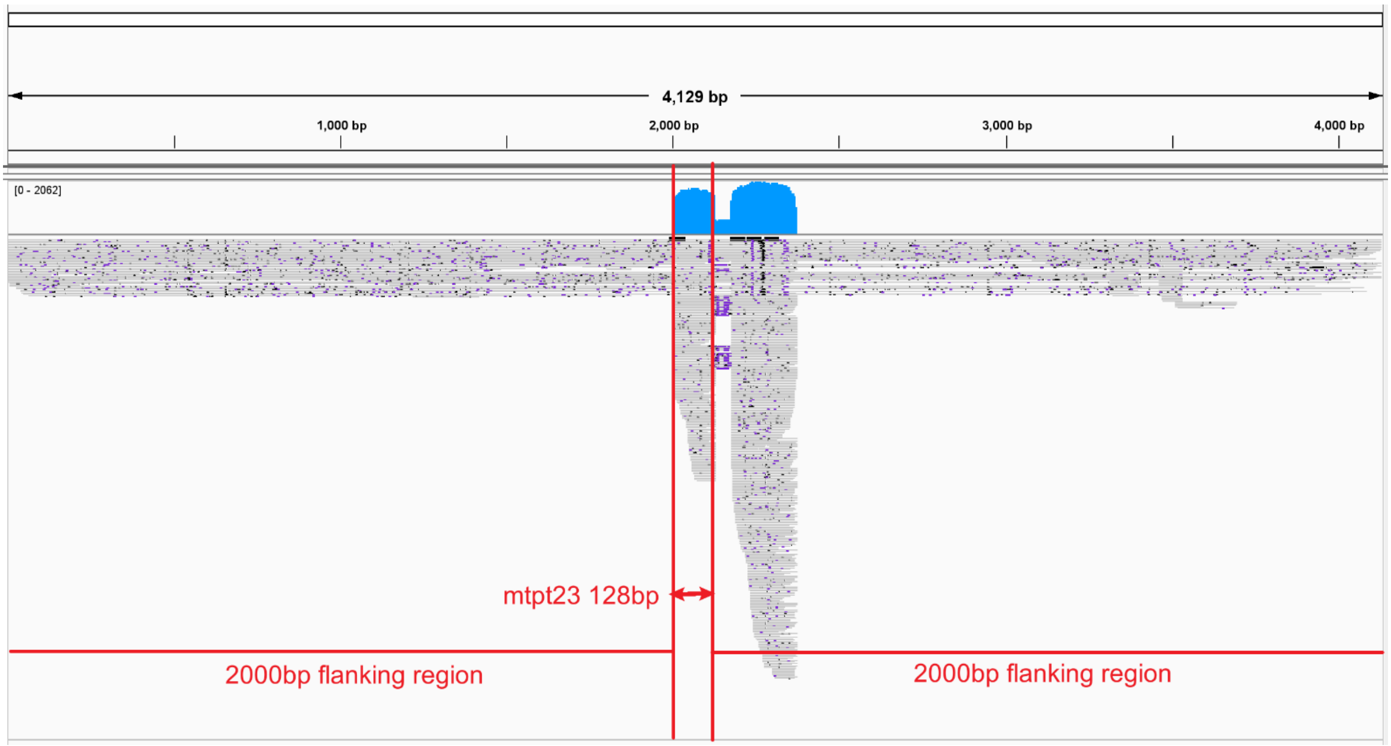

Figure S6 Alignments of the RNA sequencing reads to the coding sequences of twenty PCGs in the mitogenome of *S. officinalis*. The reference sequences are shown at the bottom. The gray lines above represent the reads mapped to the reference sequence. Bases matching those in the reference sequences are shaded in gray. Bases not matching those in the reference sequences are shown in green for “A”, brown for “G”, blue for “C”, and red for “T”. The RNA editing sites are shown in the red squares.

a alignment of RNA-seq reads to the coding sequence of *atp4*. 11 RNA-seq editing sites: atp4-59, 71, 89, 118, 215, 227, 248, 251, 395, 407, and 416 were highlighted in red squares.

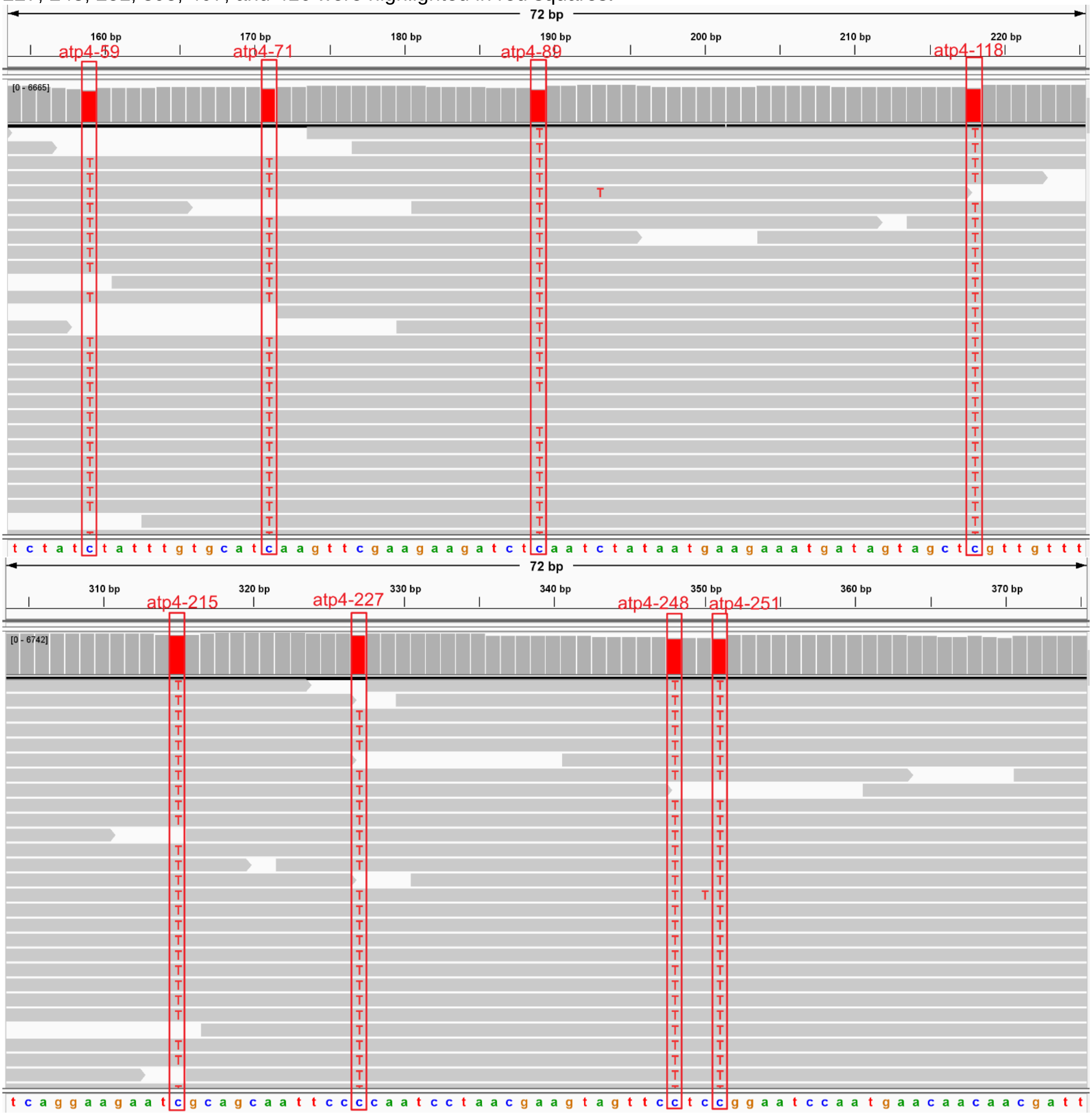

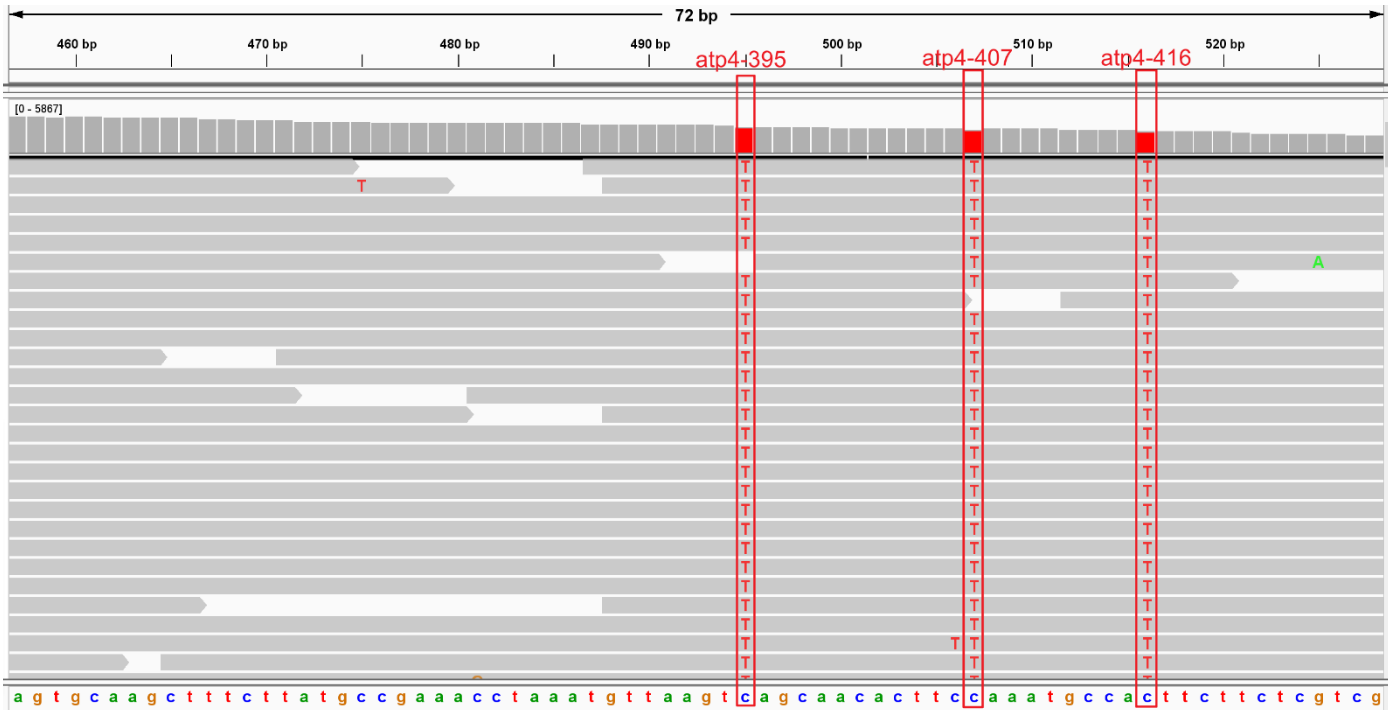
72 bp
460 bp
470 bp
480 bp
490 bp
500 bp
510 bp
520 bp
atp4-395
atp4-407
atp4-416
[0 - 5867]
a g t g c a a g c t t t c t t a t g c c g a a a c c t a a a t g t t a a g t c a g c a a c a c t t c c a a a t g c c a c t t c t t c t c g t c g

b alignment of RNA-seq reads to the coding sequence of *atp6*. Eight RNA-seq editing sites: atp6-26, 77, 146, 164, 172, 311, 370, and 372 were highlighted in red squares.

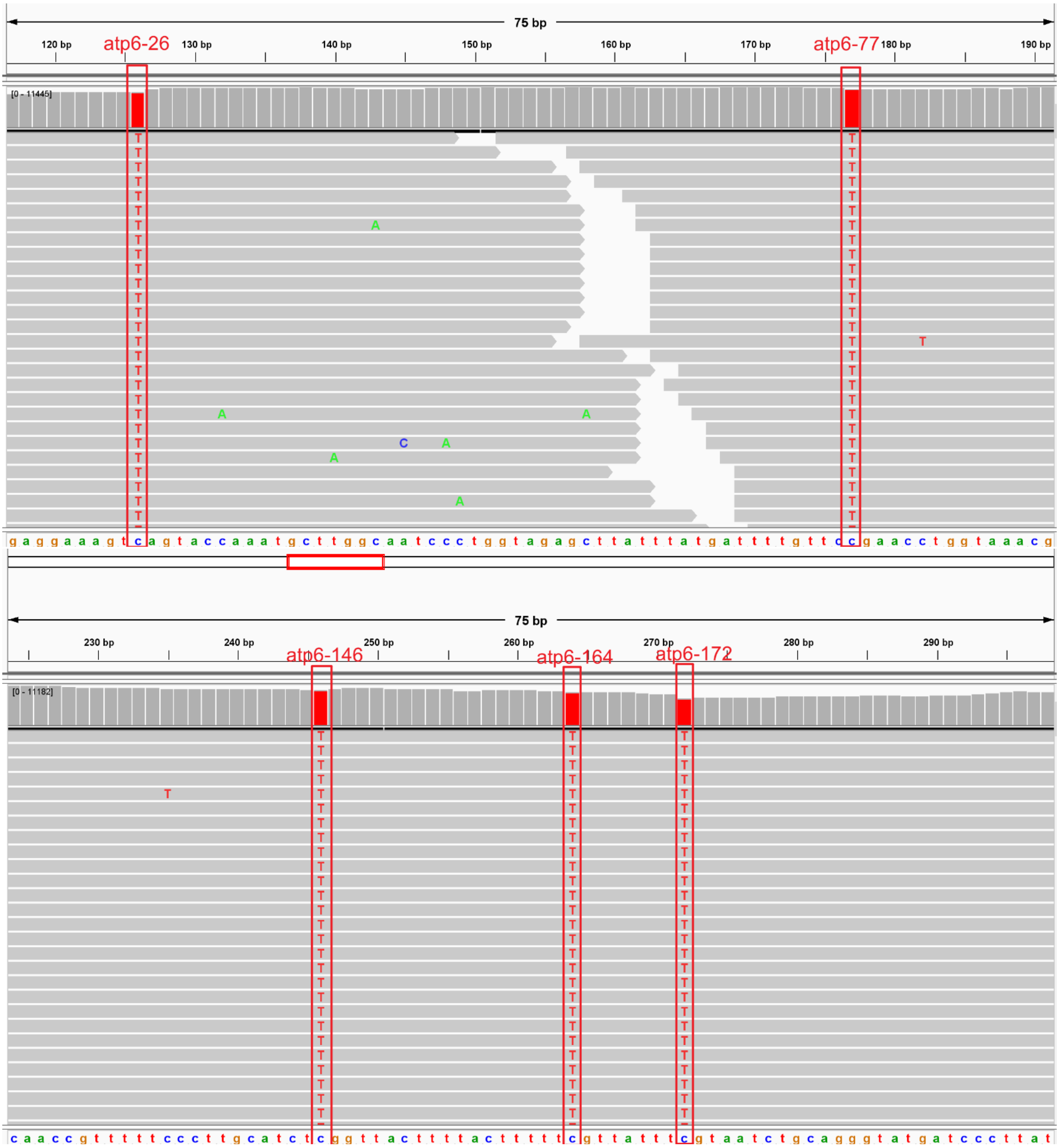

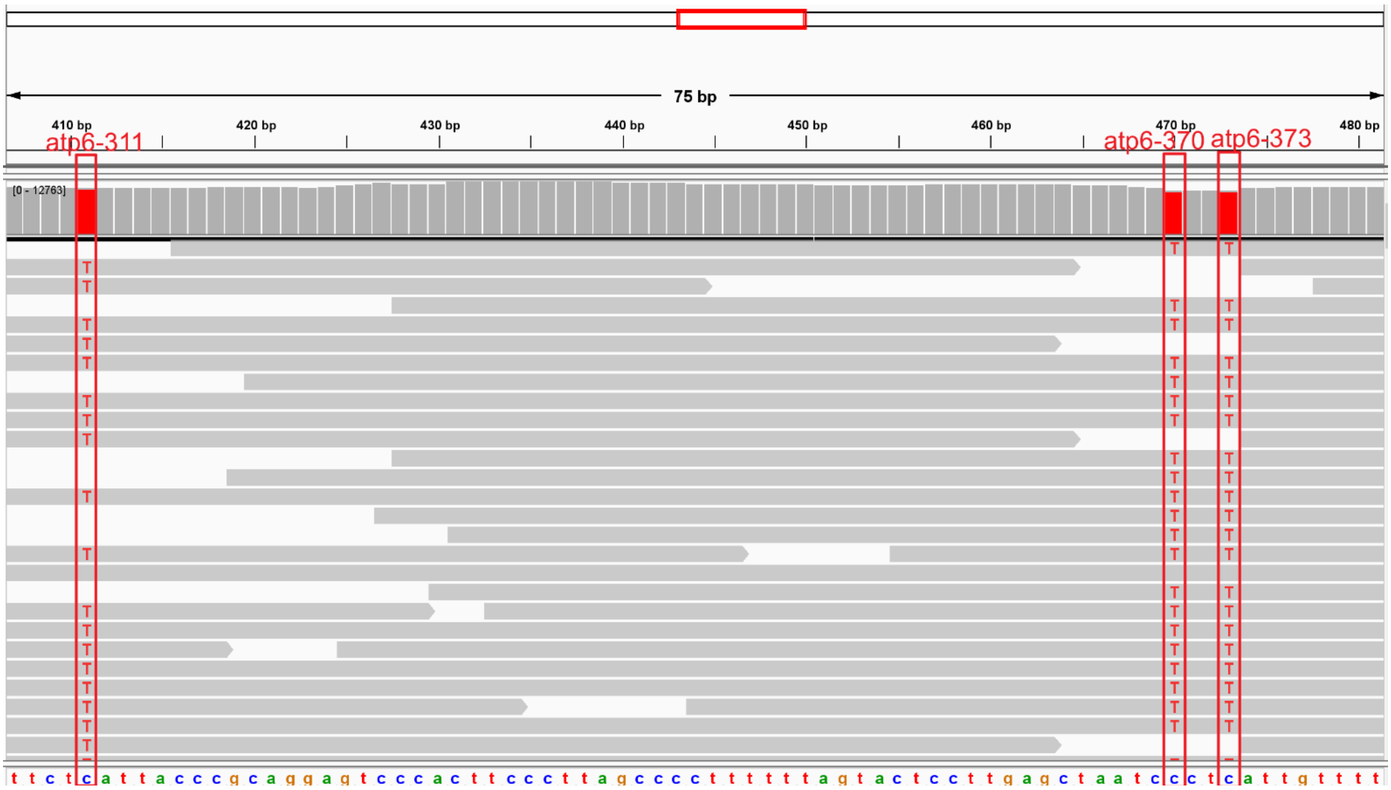

c alignment of RNA-seq reads to the coding sequence of *atp8*. Two RNA-seq editing sites: atp8-30 and 58 were highlighted in red squares.

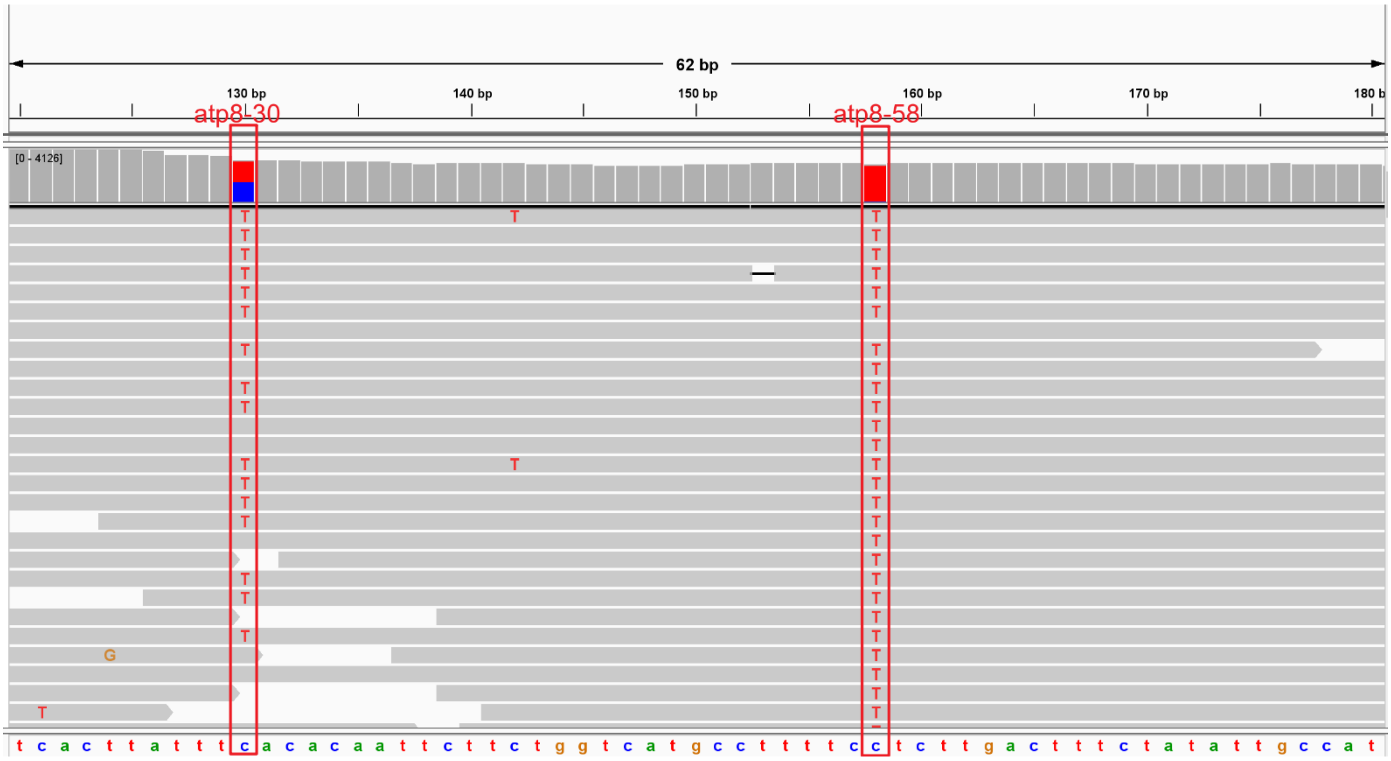

d alignment of RNA-seq reads to the coding sequence of *atp9*. One RNA-seq editing site: atp9-212 was highlighted in red squares.

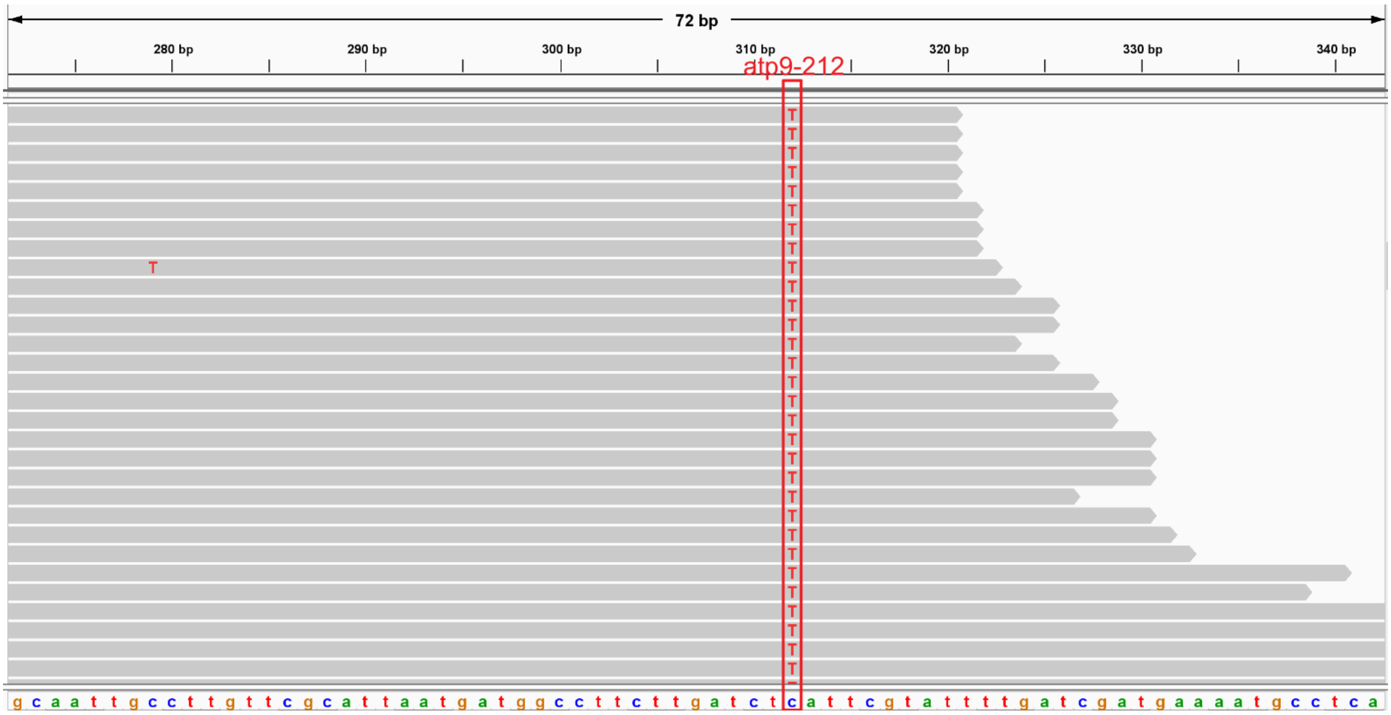

e alignment of RNA-seq reads to the coding sequence of *ccmB*. 13 RNA-seq editing sites: ccmB-28, 80, 87, 128, 286, 304, 313, 338, 367, 380, 424, 428, and 551 were highlighted in red squares.

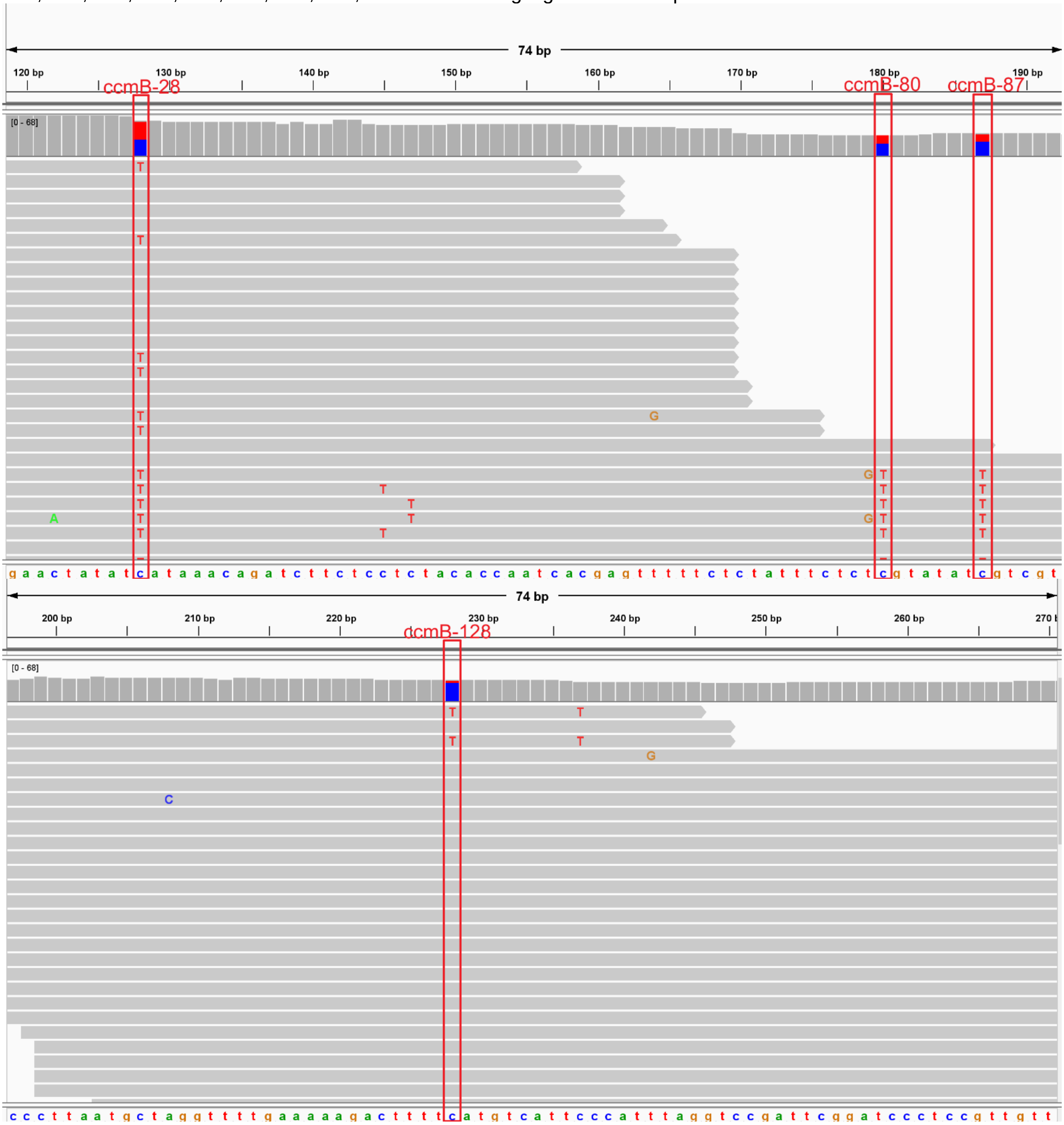

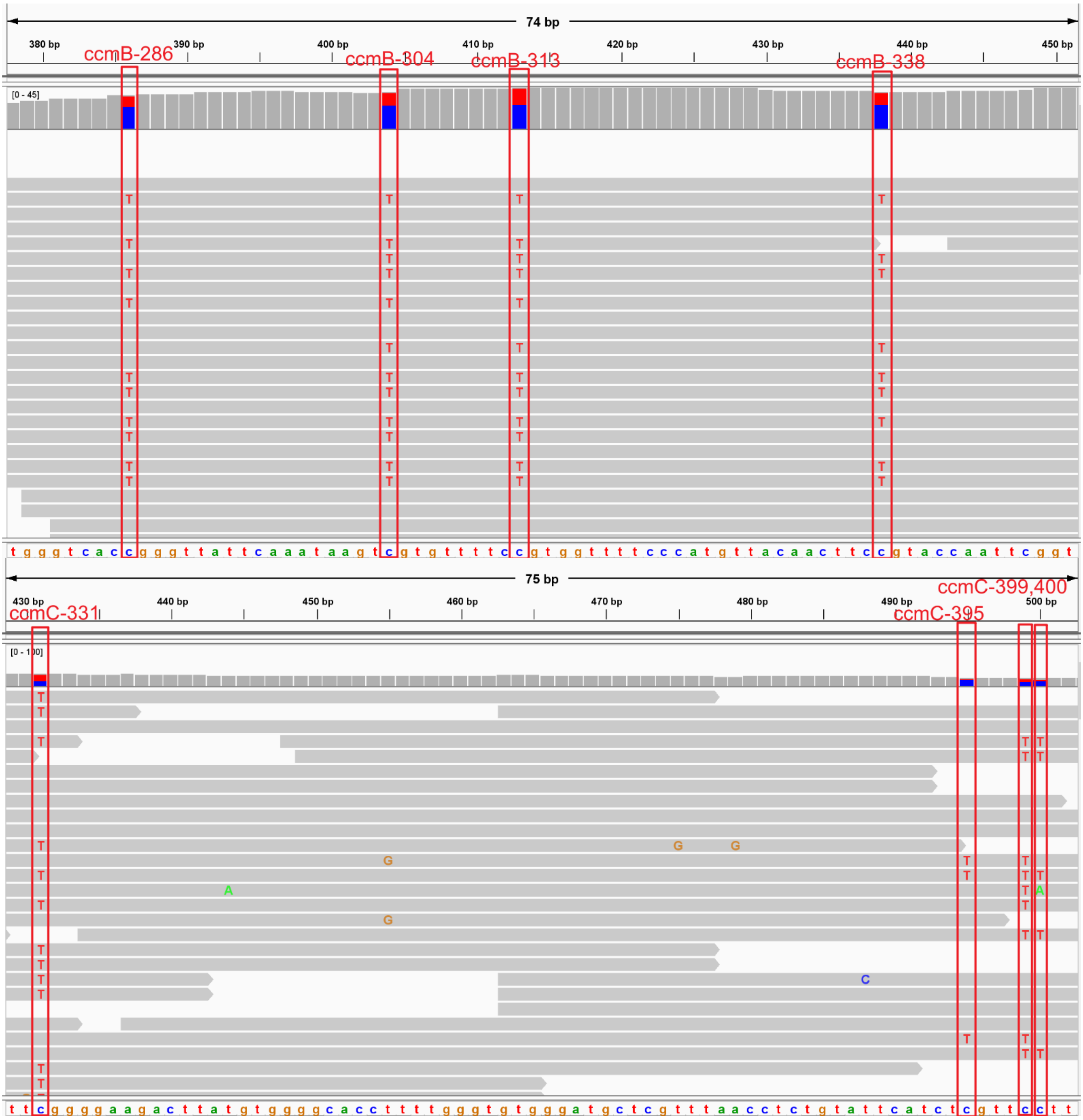
74 bp
380 bp
390 bp
400 bp
410 bp
420 bp
430 bp
440 bp
450 bp
ccmB-286
ccmB-304
ccmB-313
ccmB-338
[0 - 45]
75 bp
430 bp
440 bp
450 bp
460 bp
470 bp
480 bp
490 bp
500 bp
ccmC-331
ccmC-395
ccmC-399,400
[0 - 100]

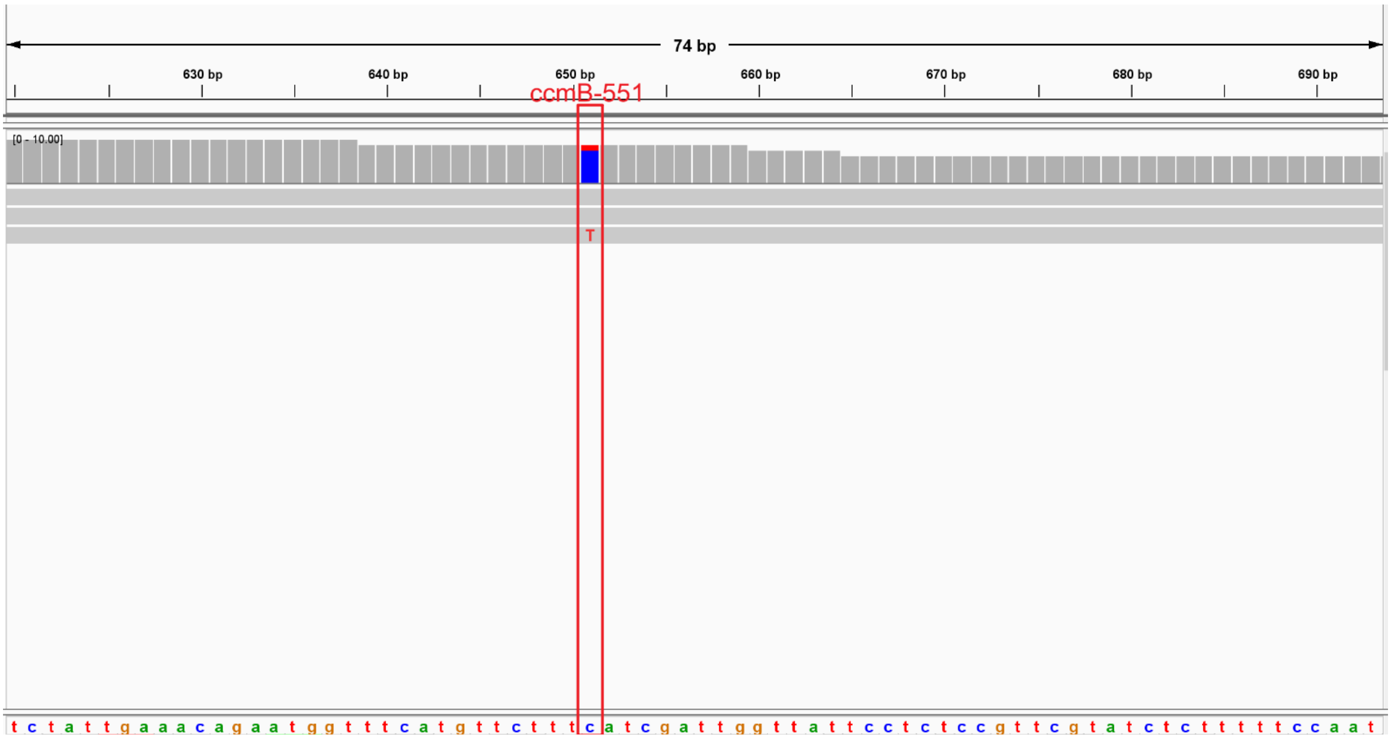
74 bp
630 bp
640 bp
650 bp
660 bp
670 bp
680 bp
690 bp
ccmB-551
[0 - 10.00]
T
t c t a t t g a a a c a g a a t g g t t t c a t g t t c t t t c a t c g a t t g g t t a t t c c t c t c c g t t c g t a t c t c t t t t t c c a a t

f alignment of RNA-seq reads to the coding sequence of *ccmC*. 26 RNA-seq editing sites: *ccmC*-38, 76, 103, 115, 133, 161, 179, 184, 253, 281, 299, 331, 395, 399, 400, 436, 446, 458, 548, 568, 575, 605, 608, 614, 650, and 673 were highlighted in red squares.

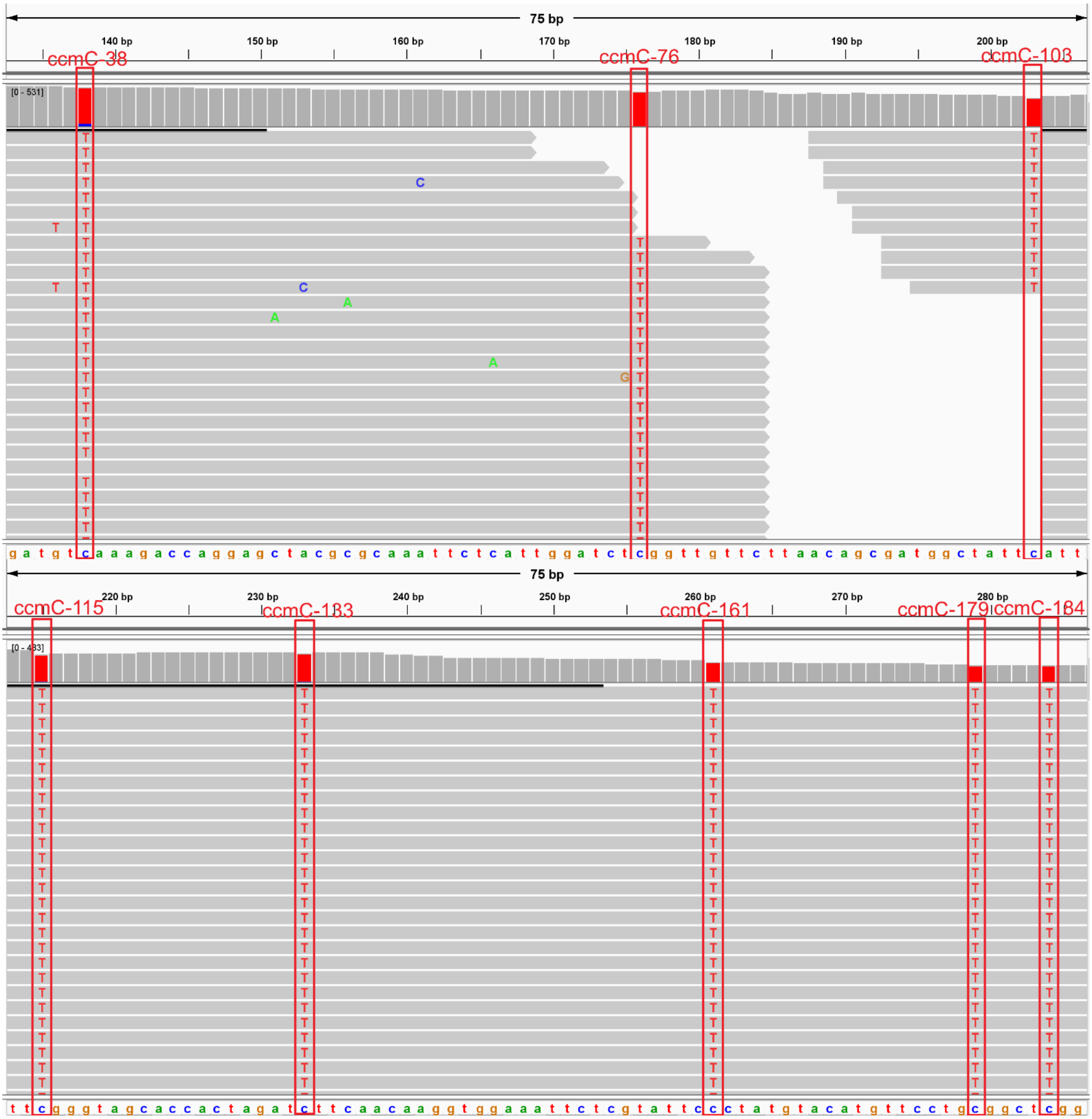

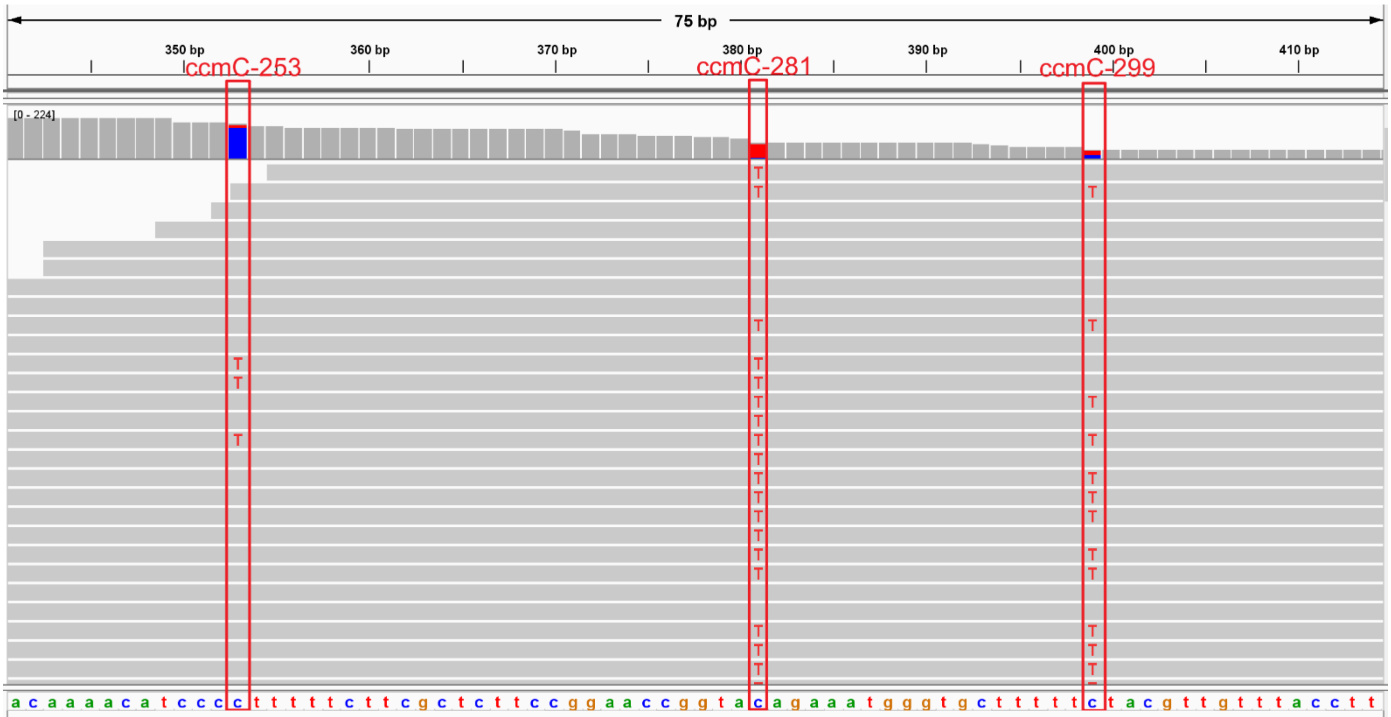
75 bp
350 bp
360 bp
370 bp
380 bp
390 bp
400 bp
410 bp
ccmC-253
ccmC-281
ccmC-299
[0 - 224]

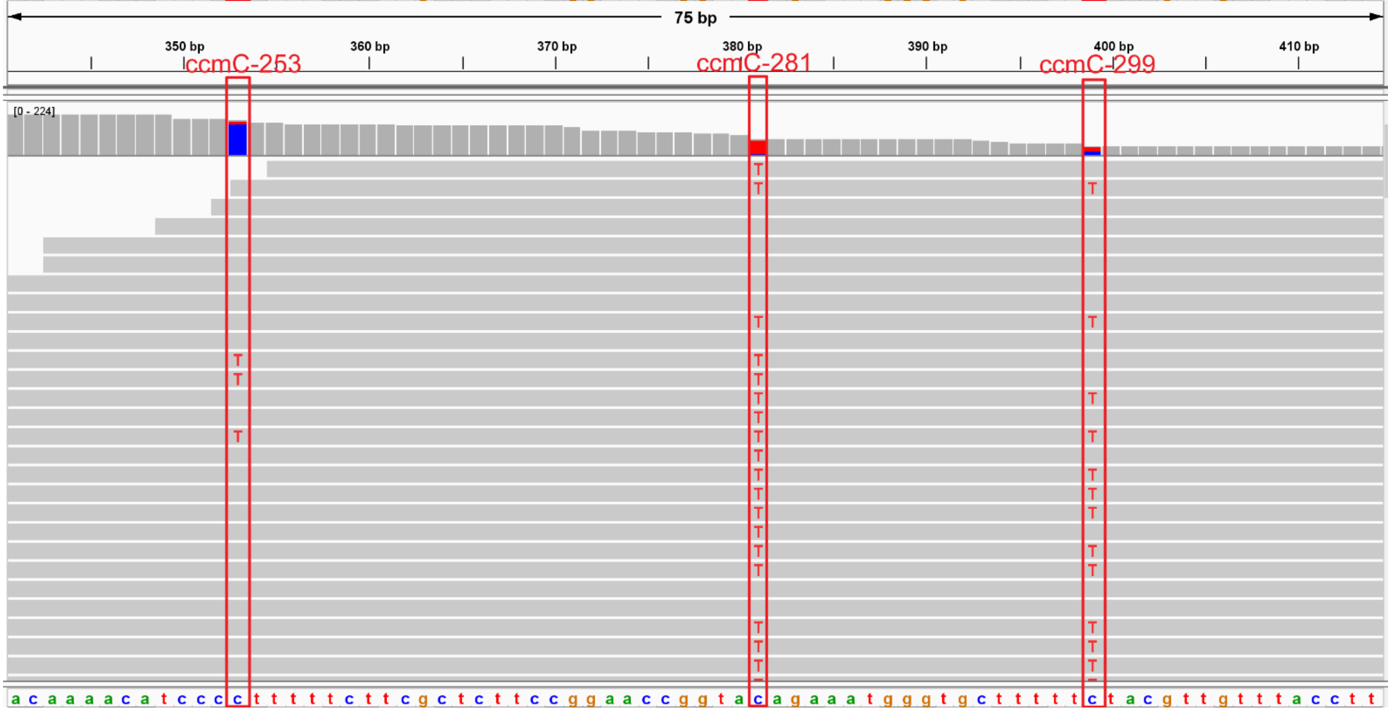
75 bp
350 bp
360 bp
370 bp
380 bp
390 bp
400 bp
410 bp
ccmC-253
ccmC-281
ccmC-299
[0 - 224]

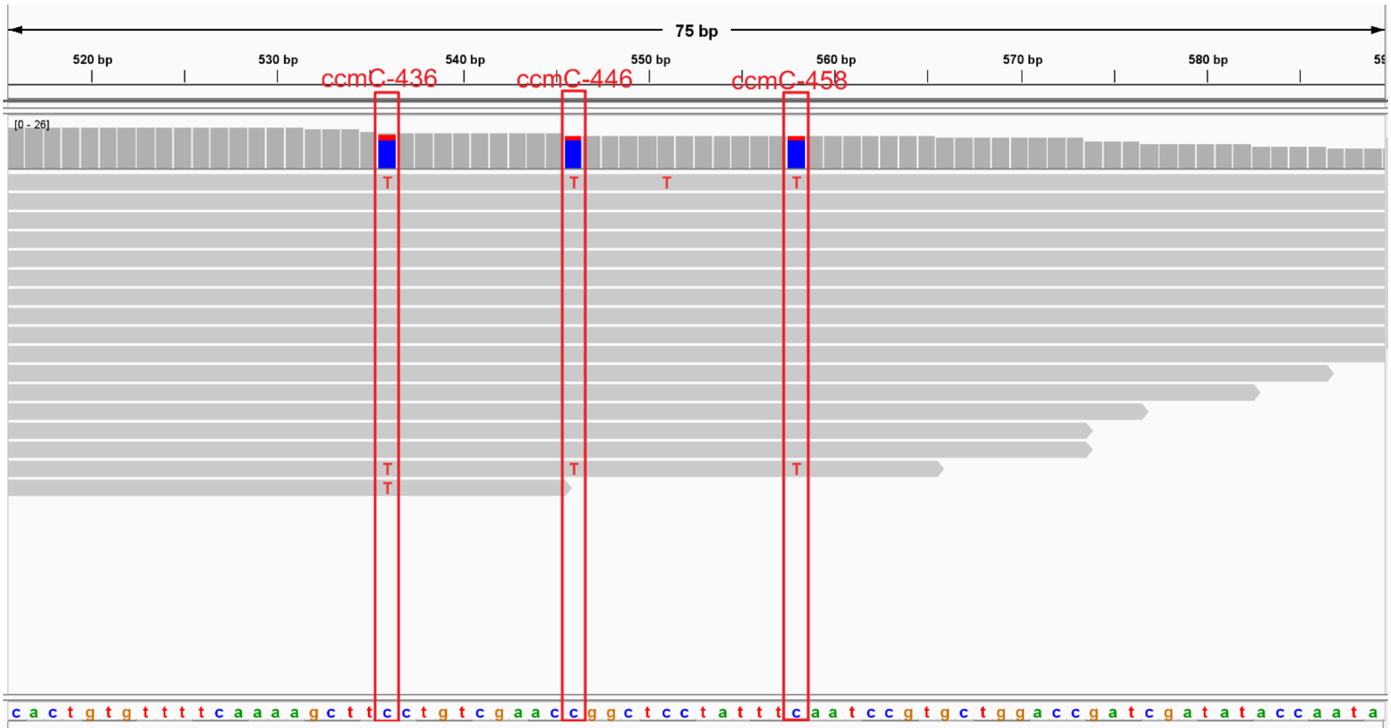

75 bp
520 bp
530 bp
540 bp
550 bp
560 bp
570 bp
580 bp
ccmC-436
ccmC-446
ccmC-458
[0 - 26]

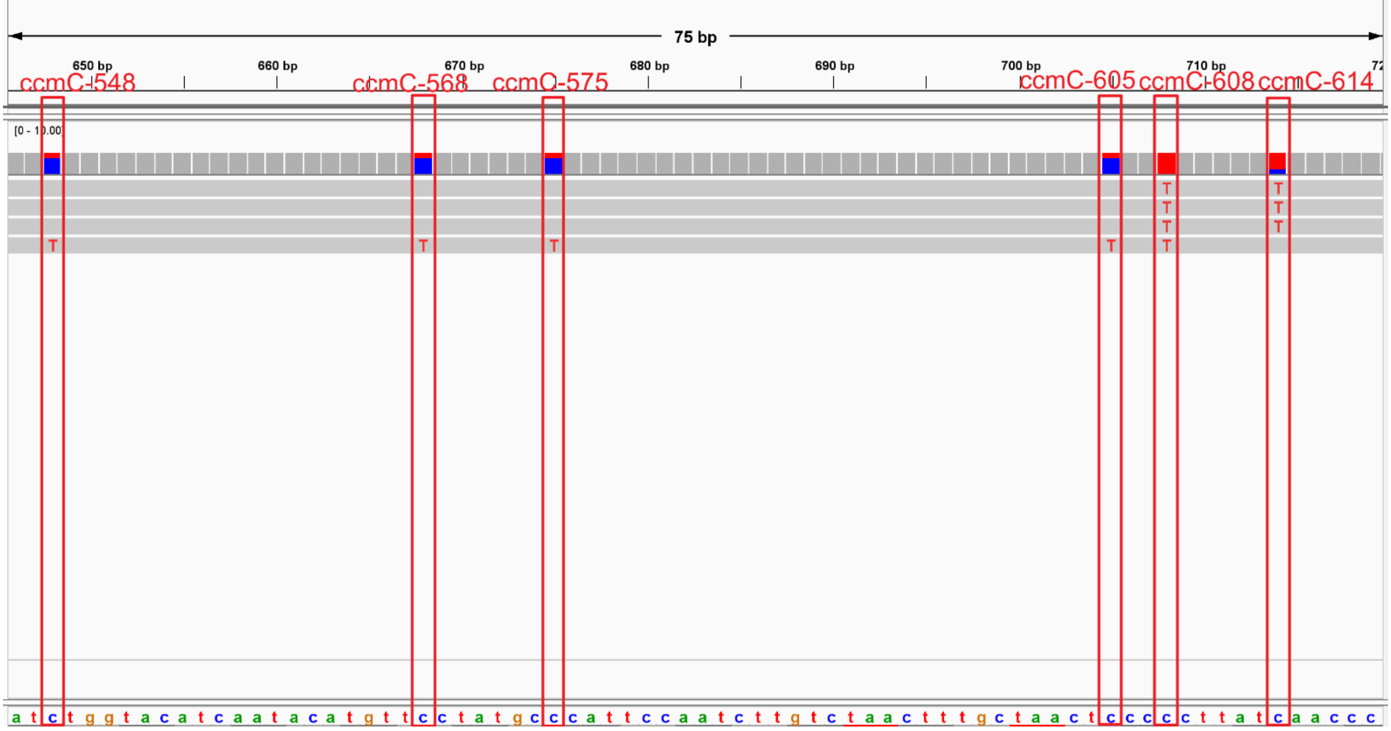

75 bp
650 bp
660 bp
670 bp
680 bp
690 bp
700 bp
710 bp
ccmC-548
ccmC-568
ccmC-575
ccmC-605
ccmC-608
ccmC-614

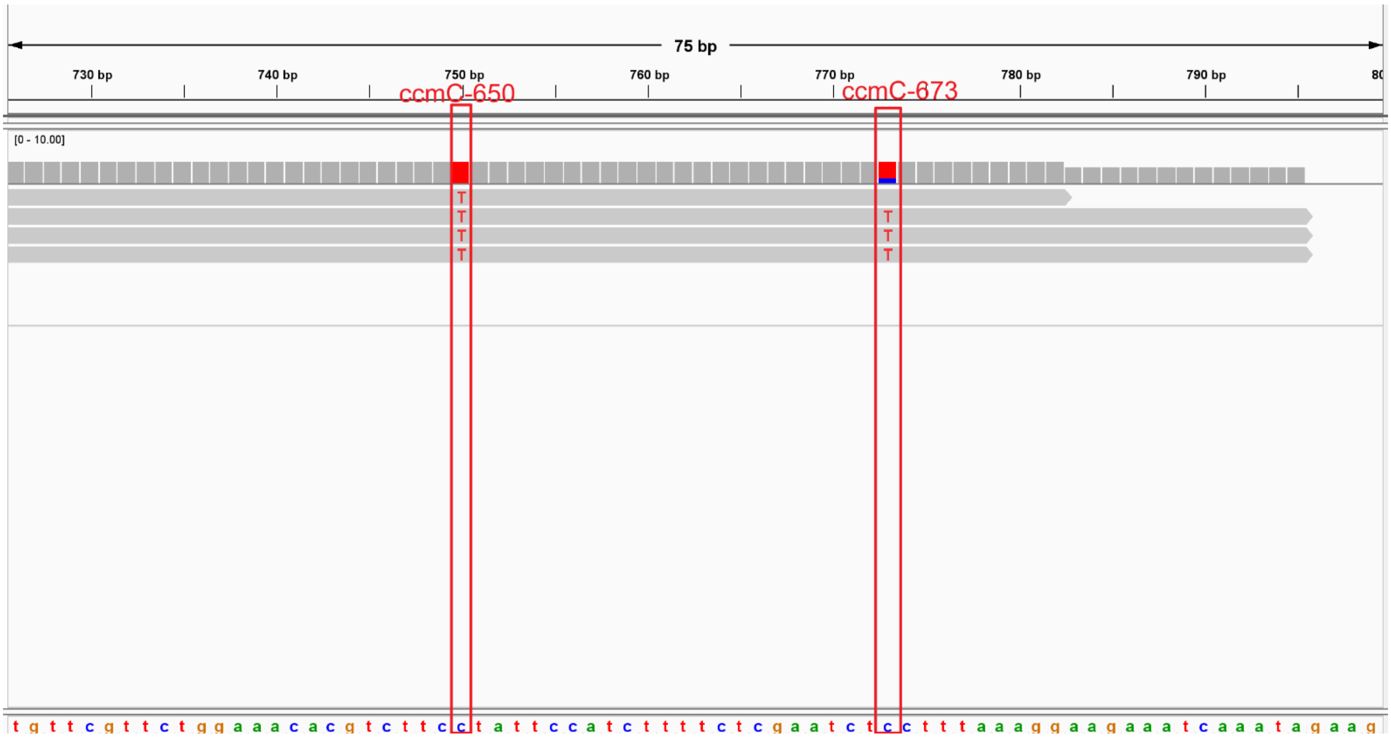
75 bp
730 bp
740 bp
750 bp
760 bp
770 bp
780 bp
790 bp
ccmC-650
ccmC-673
[0 - 10.00]
T
T
T
T
T
T
T
t g t t c g t t c t g g a a a c a c g t c t t c c t a t t c c a t c t t t t c t c g a a t c t c c t t t a a a g g a a g a a a t c a a a t a g a a g

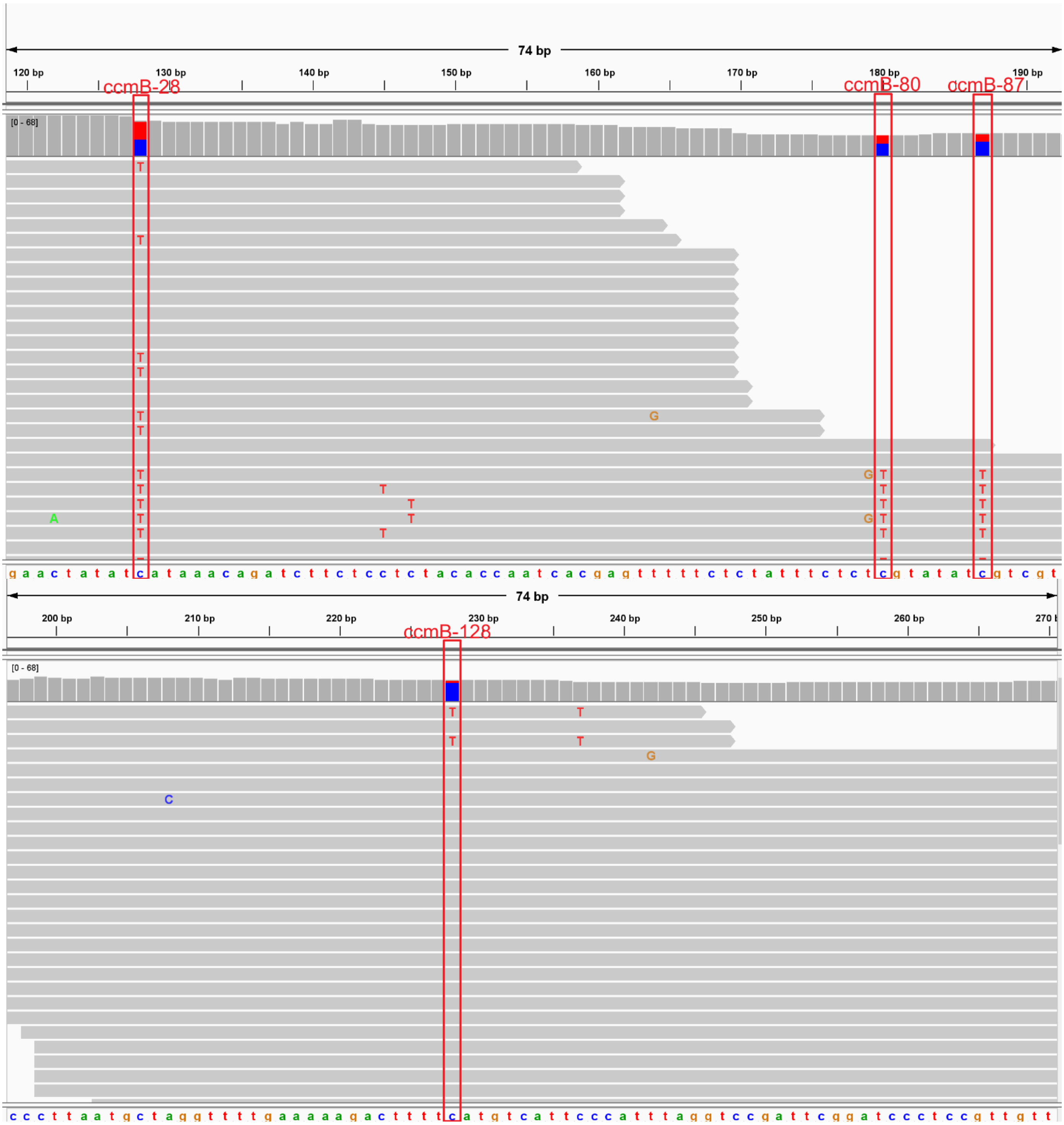
74 bp
120 bp
130 bp
140 bp
150 bp
160 bp
170 bp
180 bp
190 bp
ccmB-28
ccmB-80
ccmB-87
[0 - 68]
74 bp
200 bp
210 bp
220 bp
230 bp
240 bp
250 bp
260 bp
ccmB-128
[0 - 68]

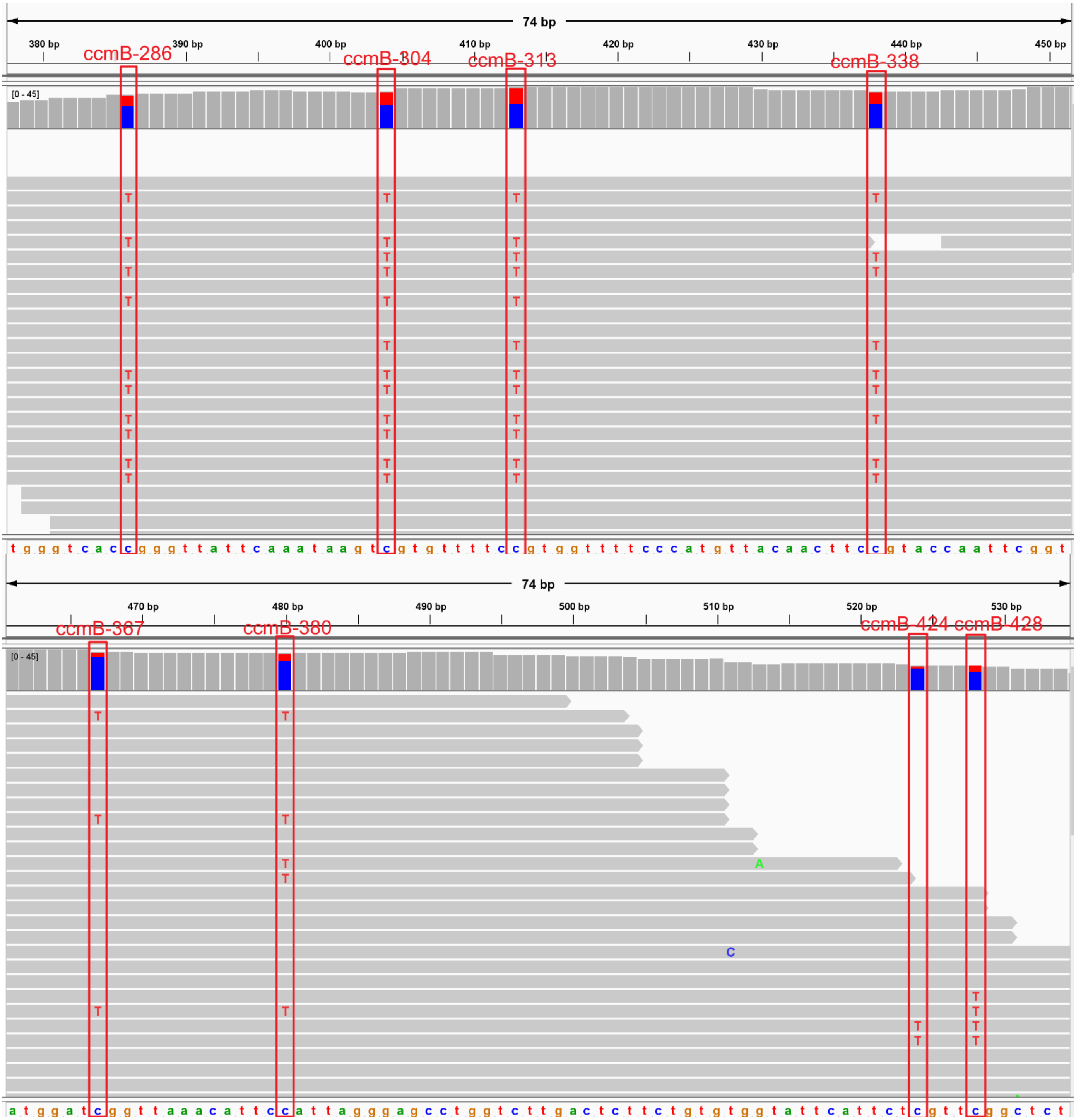

74 bp
380 bp
390 bp
400 bp
410 bp
420 bp
430 bp
440 bp
450 bp
[0 - 45]
ccmB-286
ccmB-304
ccmB-313
ccmB-338
74 bp
470 bp
480 bp
490 bp
500 bp
510 bp
520 bp
530 bp
[0 - 45]
ccmB-367
ccmB-380
ccmB-424
ccmB-428

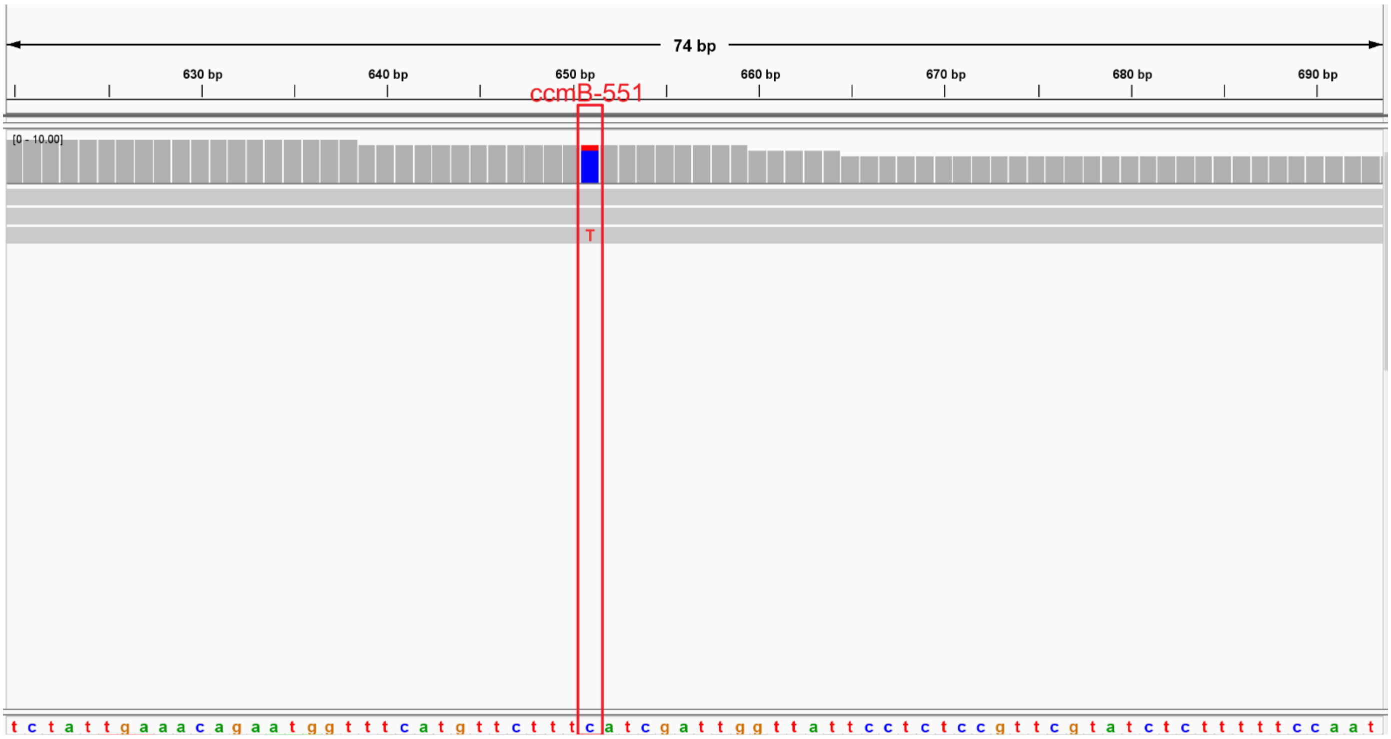
74 bp
630 bp
640 bp
650 bp
660 bp
670 bp
680 bp
690 bp
ccmB-551
[0 - 10.00]
T
t c t a t t g a a a c a g a a t g g t t t c a t g t t c t t t c a t c g a t t g g t t a t t c c t c t c c g t t c g t a t c t c t t t t t c c a a t

g alignment of RNA-seq reads to the coding sequence of *ccmFc*. 17 RNA-seq editing sites: ccmFc-16, 38, 50, 52, 103, 122, 146, 151, 155, 310, 321, 334, 378, 390, 406, 1228, 1233 were highlighted in red squares.

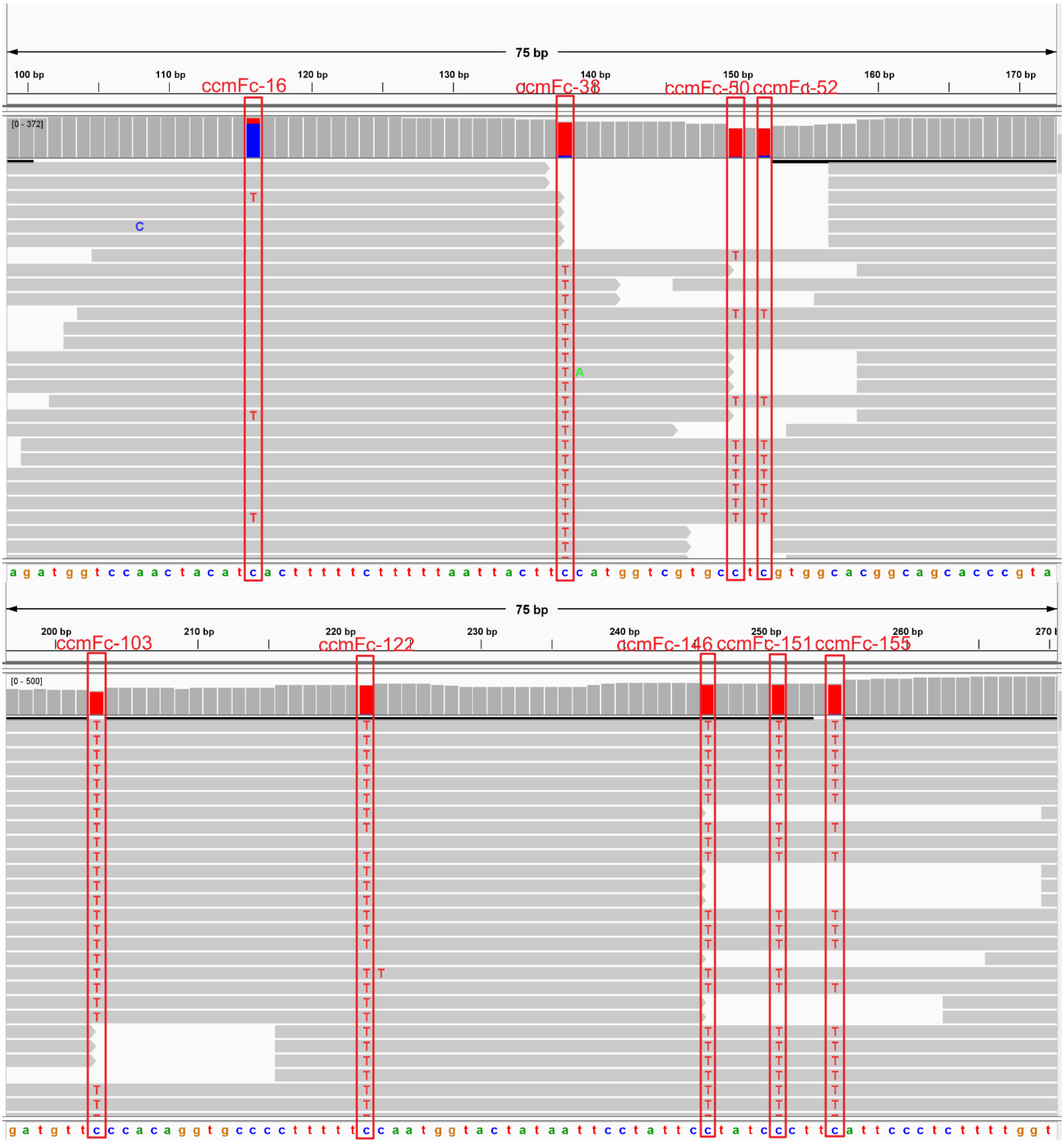

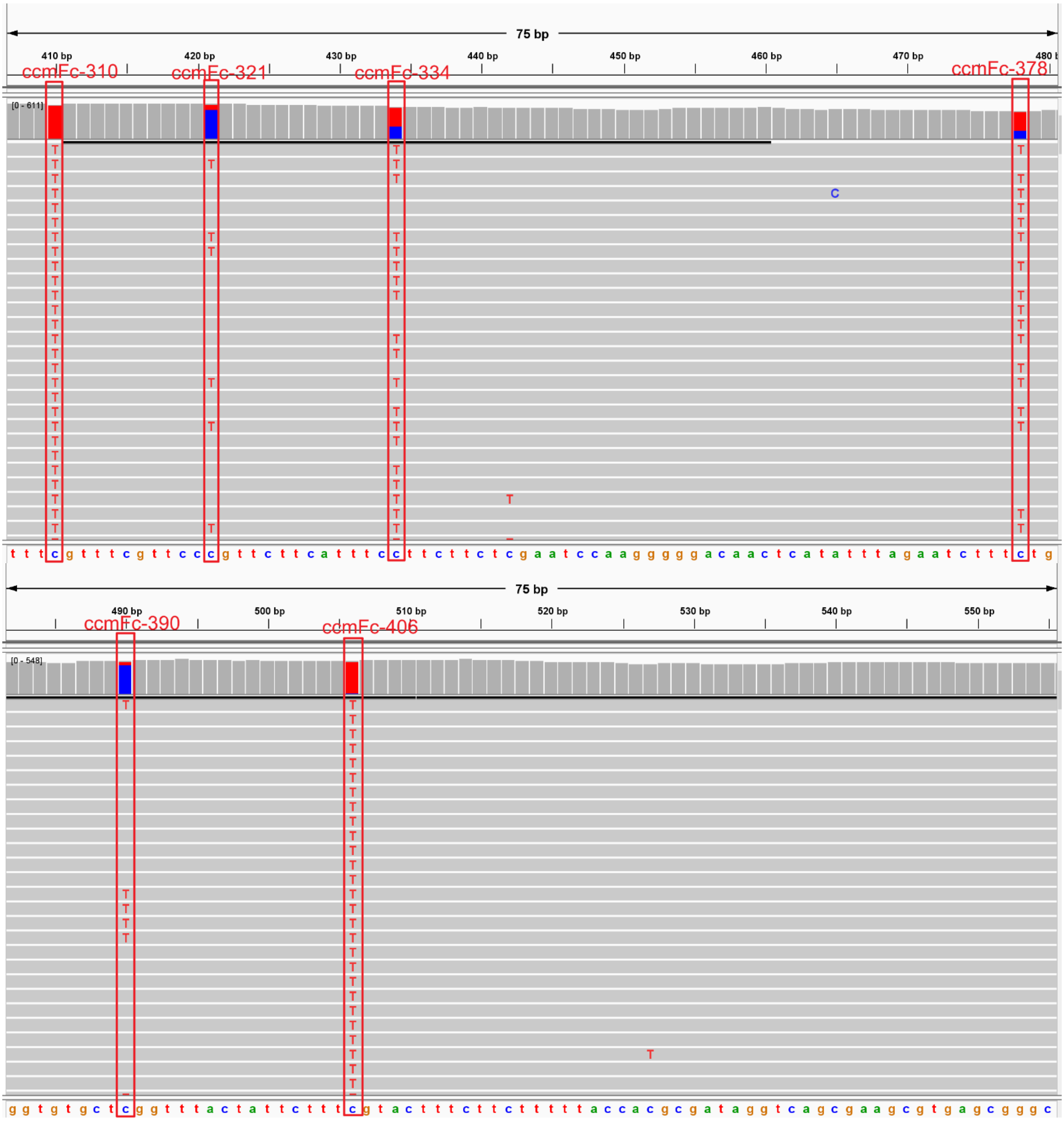
75 bp
410 bp
420 bp
430 bp
440 bp
450 bp
460 bp
470 bp
ccmFc-310
ccmFc-321
ccmFc-334
ccmFc-378
[0 - 611]
75 bp
490 bp
500 bp
510 bp
520 bp
530 bp
540 bp
550 bp
ccmFc-390
ccmFc-406
[0 - 548]

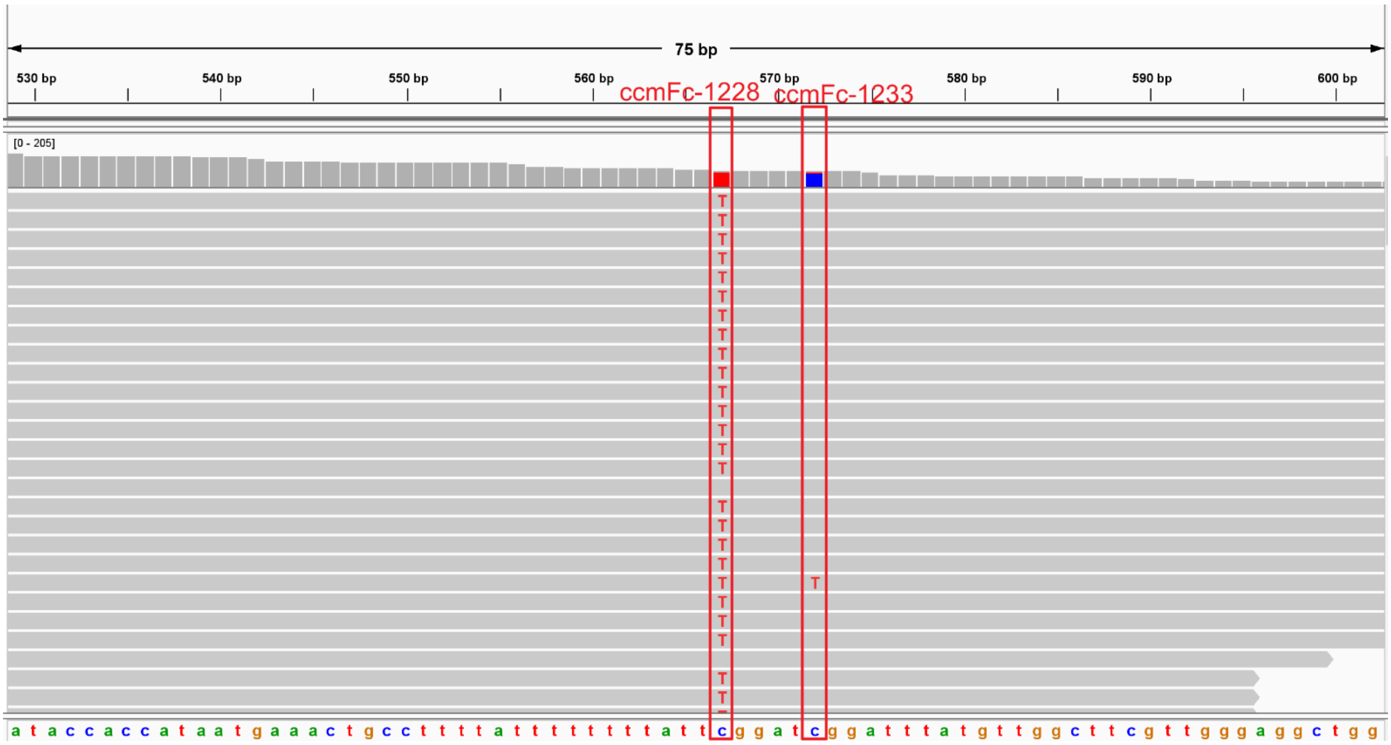
75 bp
530 bp
540 bp
550 bp
560 bp
570 bp
580 bp
590 bp
600 bp
ccmFc-1228
ccmFc-1233
[0 - 205]
a t a c c a c c a t a a t g a a a c t g c c t t t t a t t t t t t t t a t t c g g a t c g g a t t t a t g t t g g c t t c g t t g g g a g g c t g g

h alignment of RNA-seq reads to the coding sequence of *ccmFn*. 30 RNA-seq editing sites: ccmFn-38, 98, 137, 142, 151, 165, 248, 256, 283, 371, 378, 713, 722, 732, 760, 782, 794, 809, 867, 958, 1276, 1304, 1321, 1336, 1354, 1387, 1448, 1472, 1484, 1519 were highlighted in red squares.

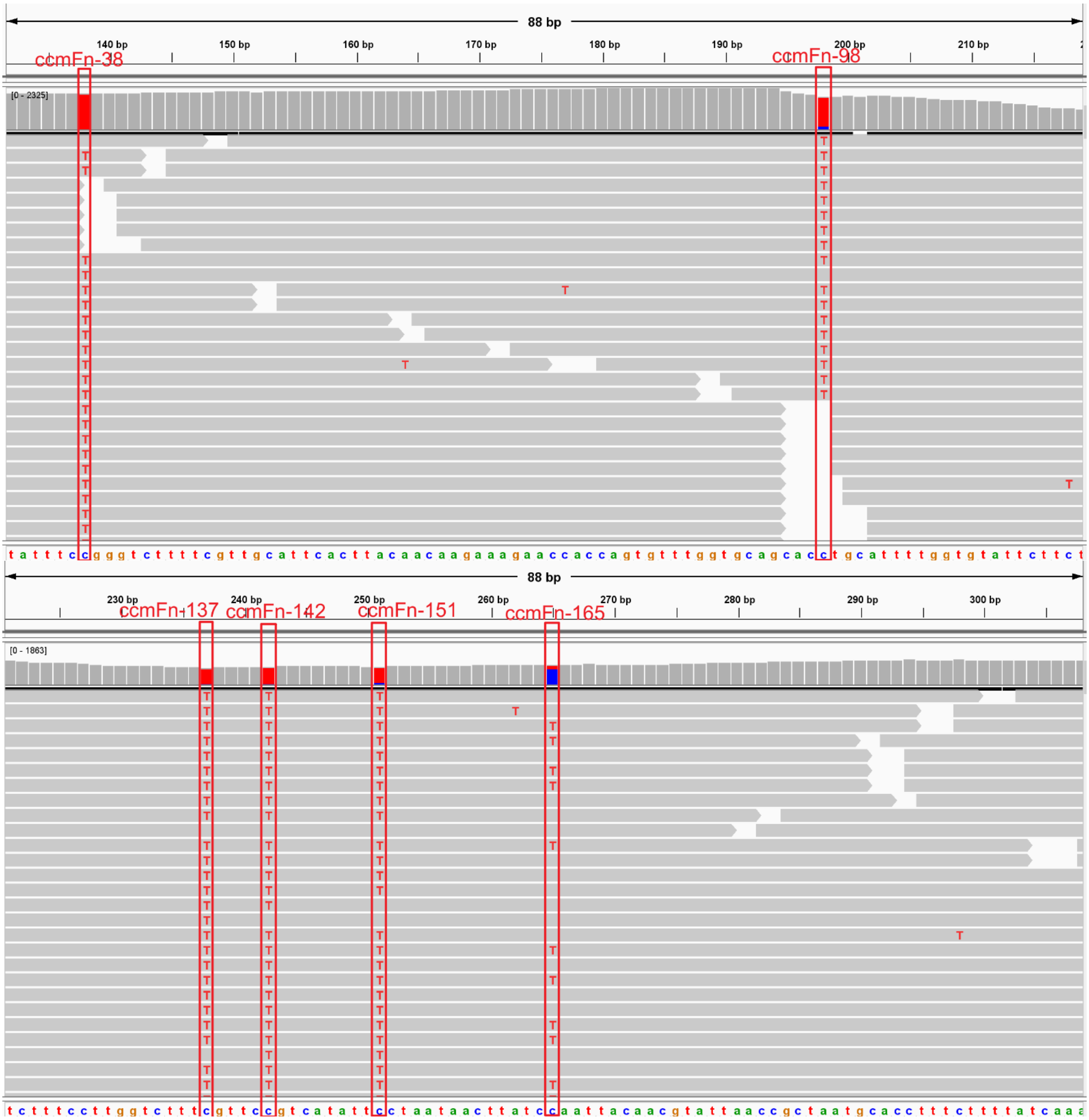

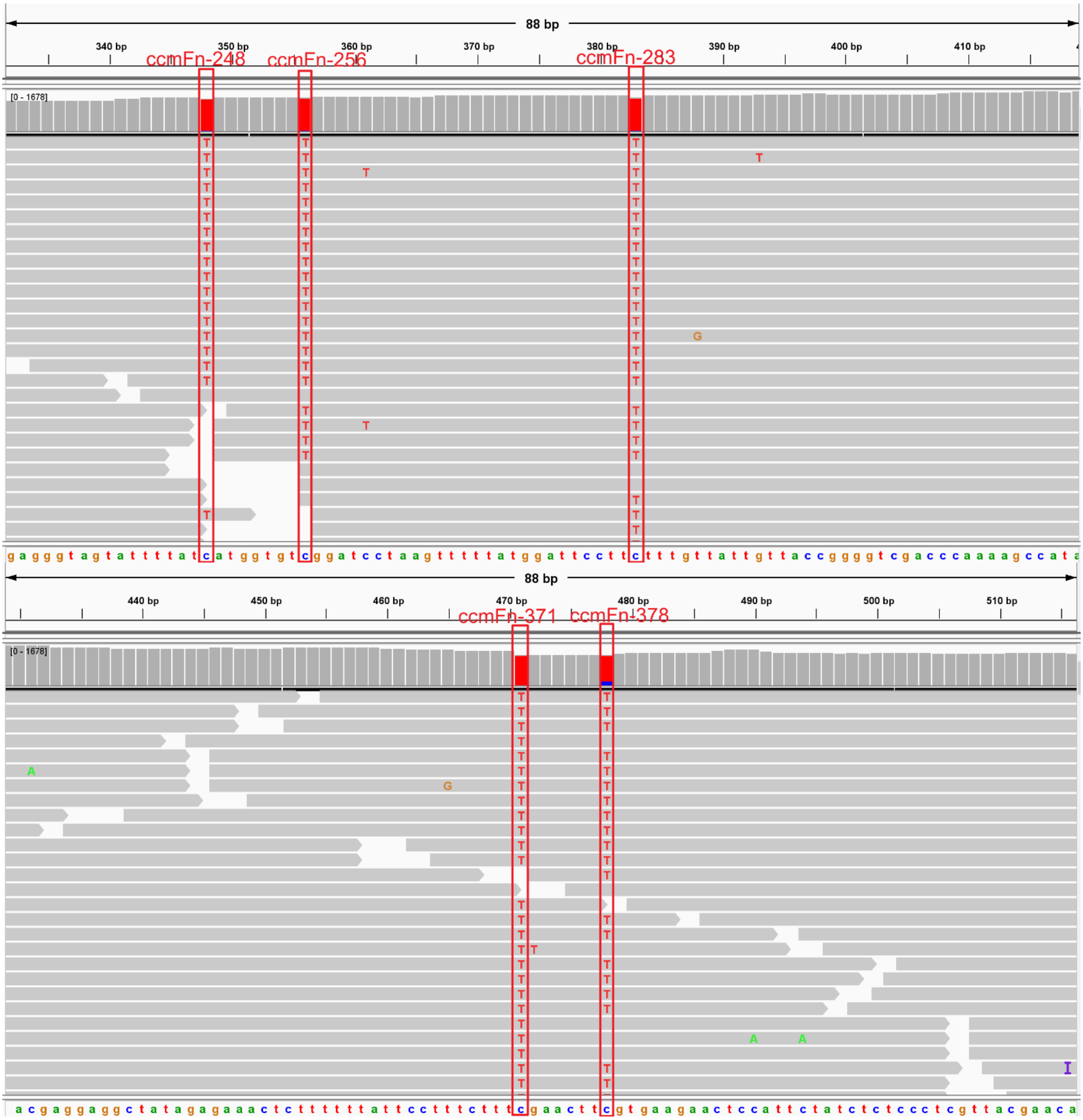

88 bp
340 bp
350 bp
360 bp
370 bp
380 bp
390 bp
400 bp
410 bp
ccmFn-248
ccmFn-256
ccmFn-283
[0 - 1678]
88 bp
440 bp
450 bp
460 bp
470 bp
480 bp
490 bp
500 bp
510 bp
ccmFn-371
ccmFn-378
[0 - 1678]

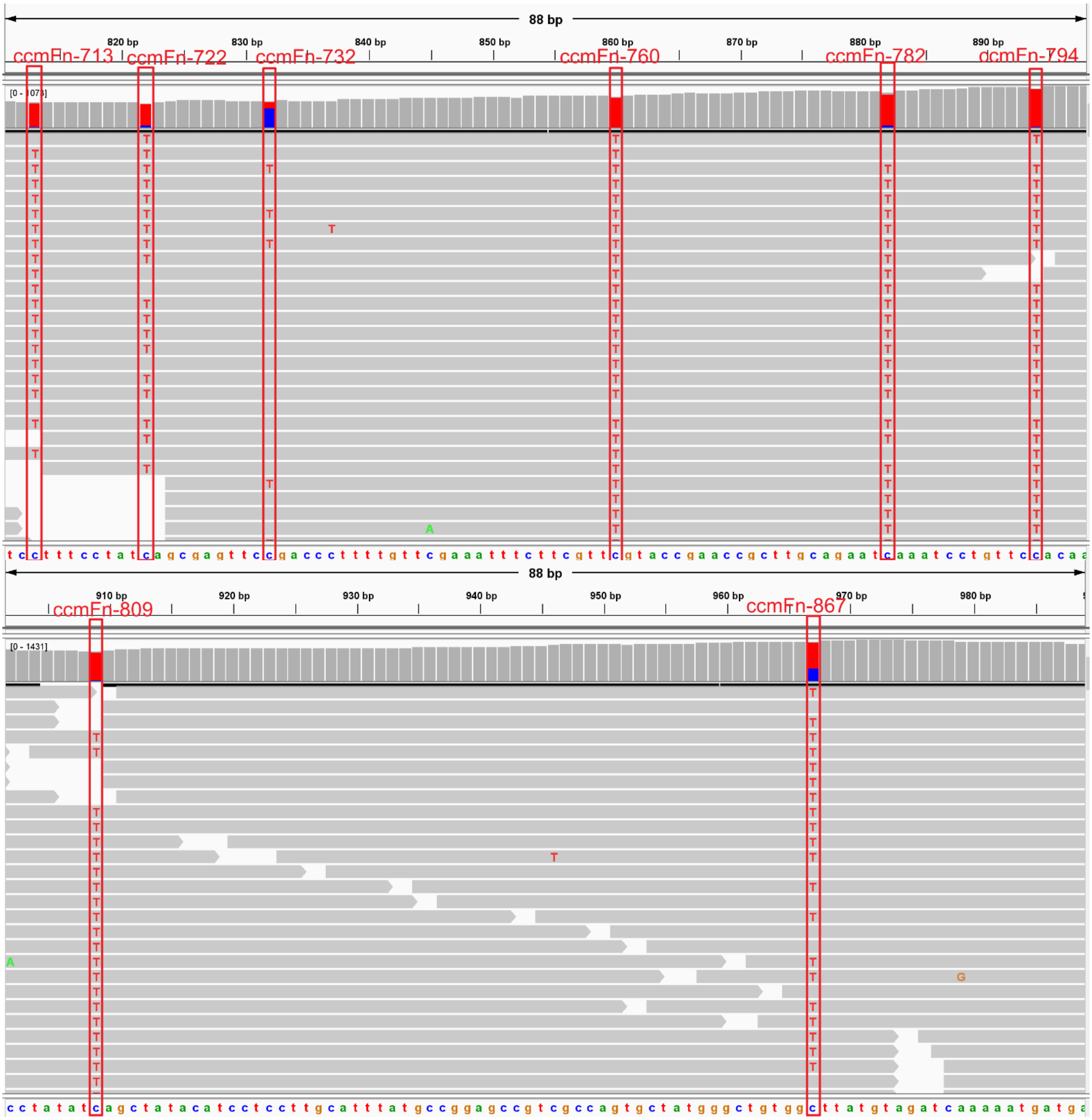
88 bp
820 bp
830 bp
840 bp
850 bp
860 bp
870 bp
880 bp
890 bp
ccmFn-713
ccmFn-722
ccmFn-732
ccmFn-760
ccmFn-782
ccmFn-794
88 bp
910 bp
920 bp
930 bp
940 bp
950 bp
960 bp
970 bp
980 bp
ccmFn-809
ccmFn-867
[0 - 1431]

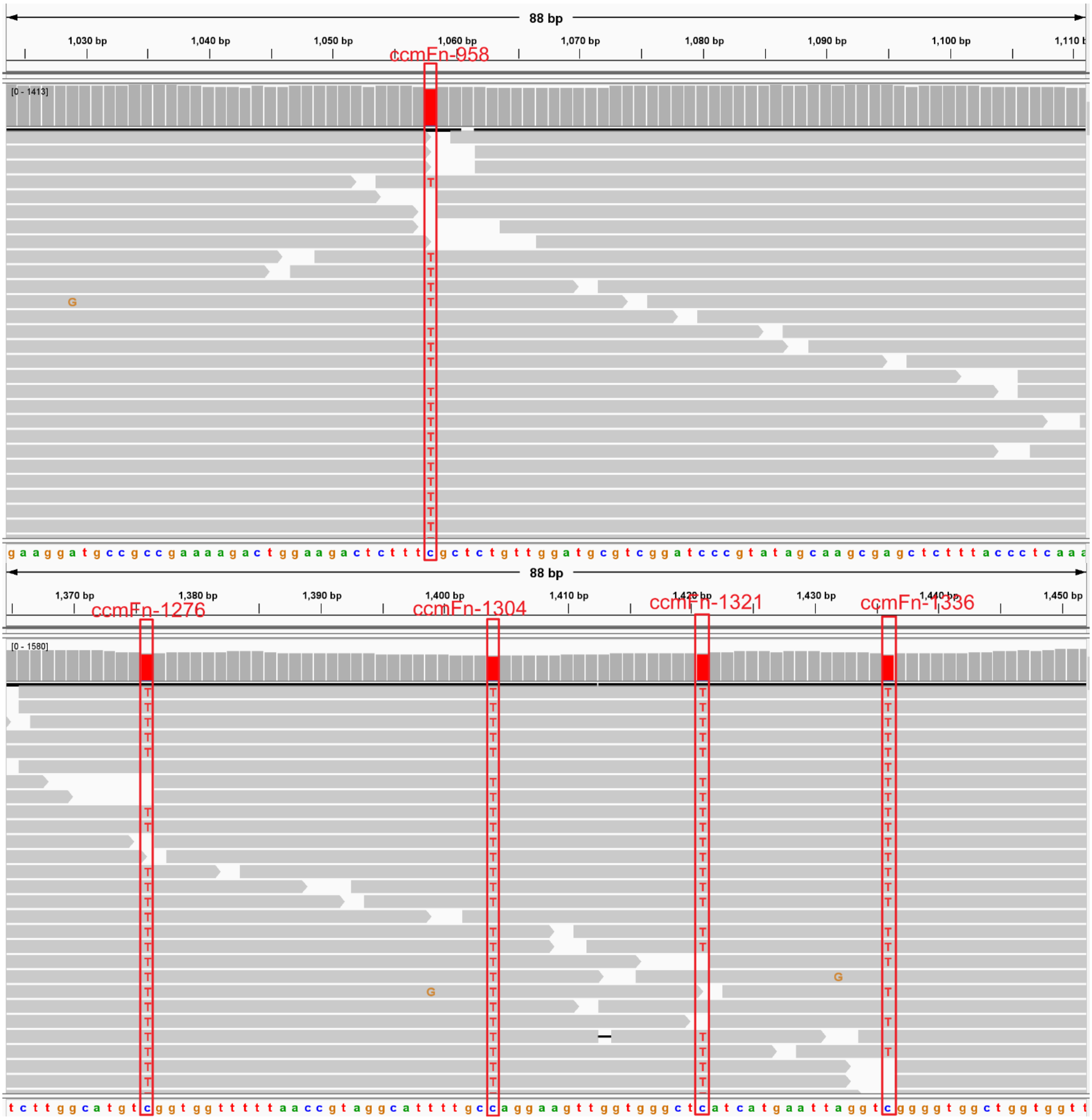
88 bp
1,030 bp
1,040 bp
1,050 bp
1,060 bp
1,070 bp
1,080 bp
1,090 bp
1,100 bp
ccmFn-958
[0 - 1413]
88 bp
1,370 bp
1,380 bp
1,390 bp
1,400 bp
1,410 bp
1,430 bp
1,450 bp
ccmFn-1276
ccmFn-1304
ccmFn-1321
ccmFn-1336
[0 - 1580]

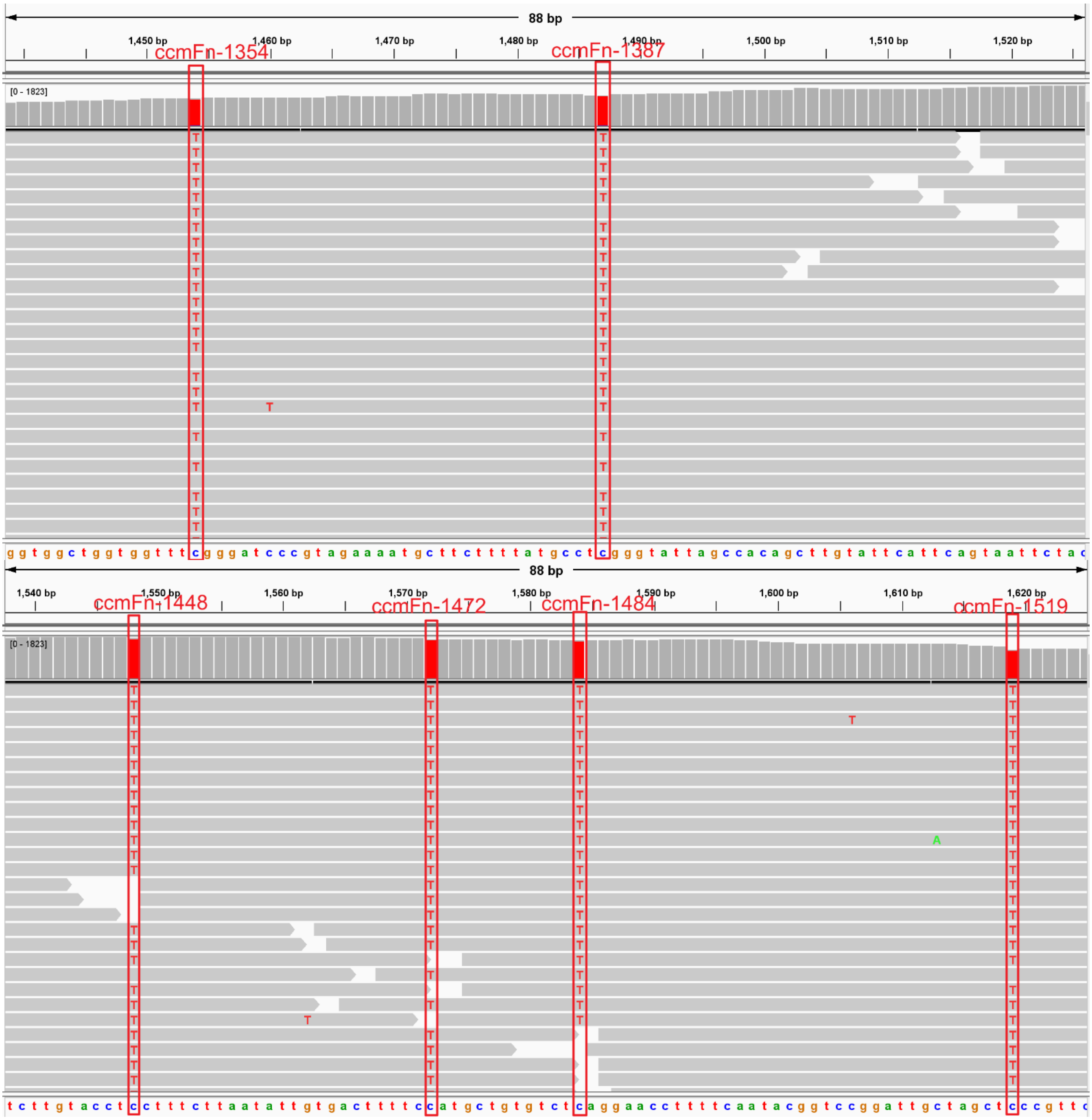

88 bp
1,450 bp
1,460 bp
1,470 bp
1,480 bp
1,490 bp
1,500 bp
1,510 bp
1,520 bp
ccmFn-1354
ccmFn-1387
[0 - 1823]
88 bp
1,540 bp
1,550 bp
1,560 bp
1,570 bp
1,580 bp
1,590 bp
1,600 bp
1,610 bp
1,620 bp
ccmFn-1448
ccmFn-1472
ccmFn-1484
ccmFn-1519
[0 - 1823]

i alignment of RNA-seq reads to the coding sequence of *cob.* 12 RNA-seq editing sites: *cob*-114, 180, 298, 325, 358, 568, 853, 908, 982, 1015, 1084, and 1160 were highlighted in red squares.

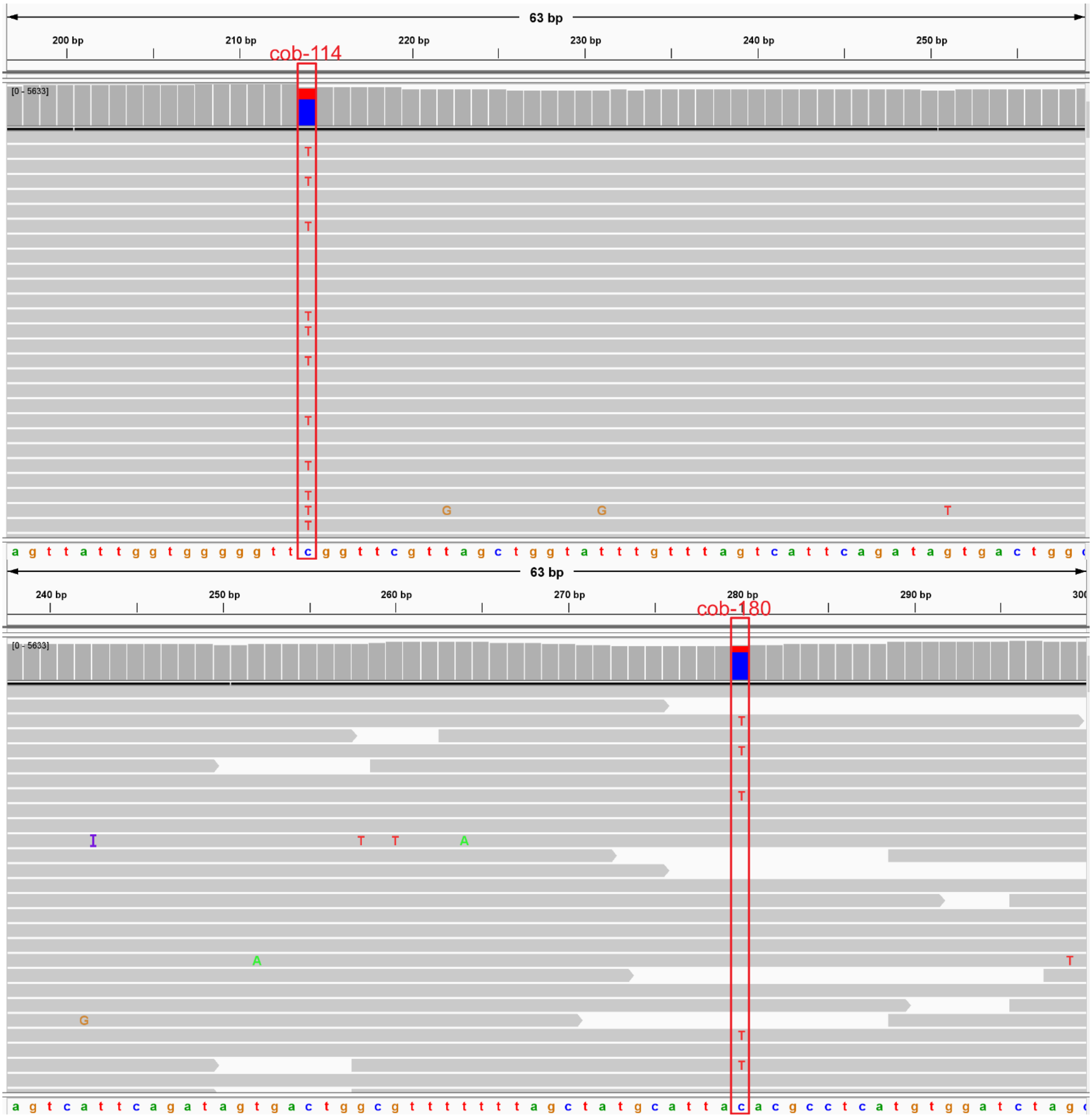

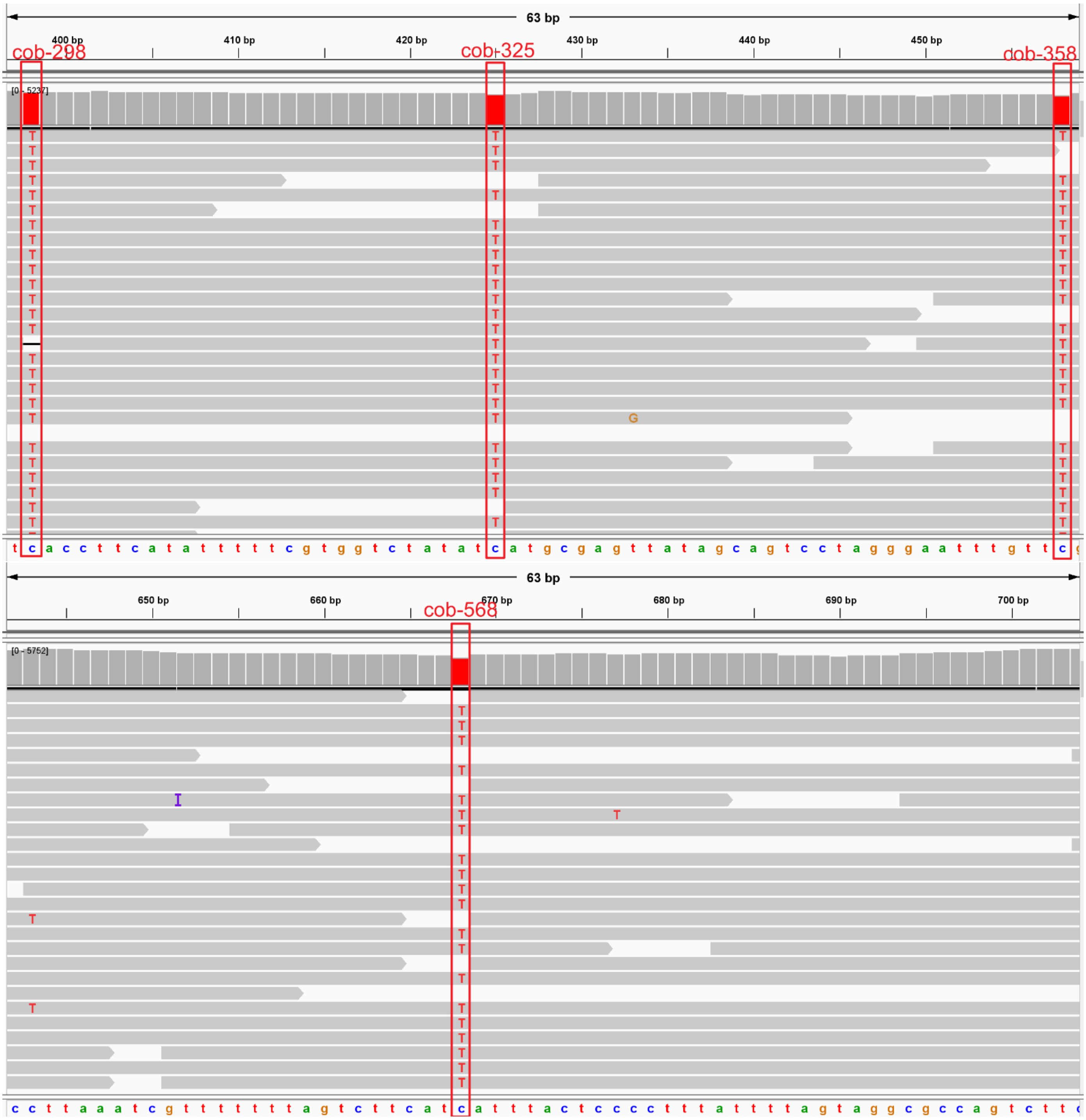
63 bp
400 bp
410 bp
420 bp
430 bp
440 bp
450 bp
cob-298
cob-325
cob-358
[0 - 5237]
63 bp
650 bp
660 bp
670 bp
680 bp
690 bp
700 bp
cob-568
[0 - 5752]

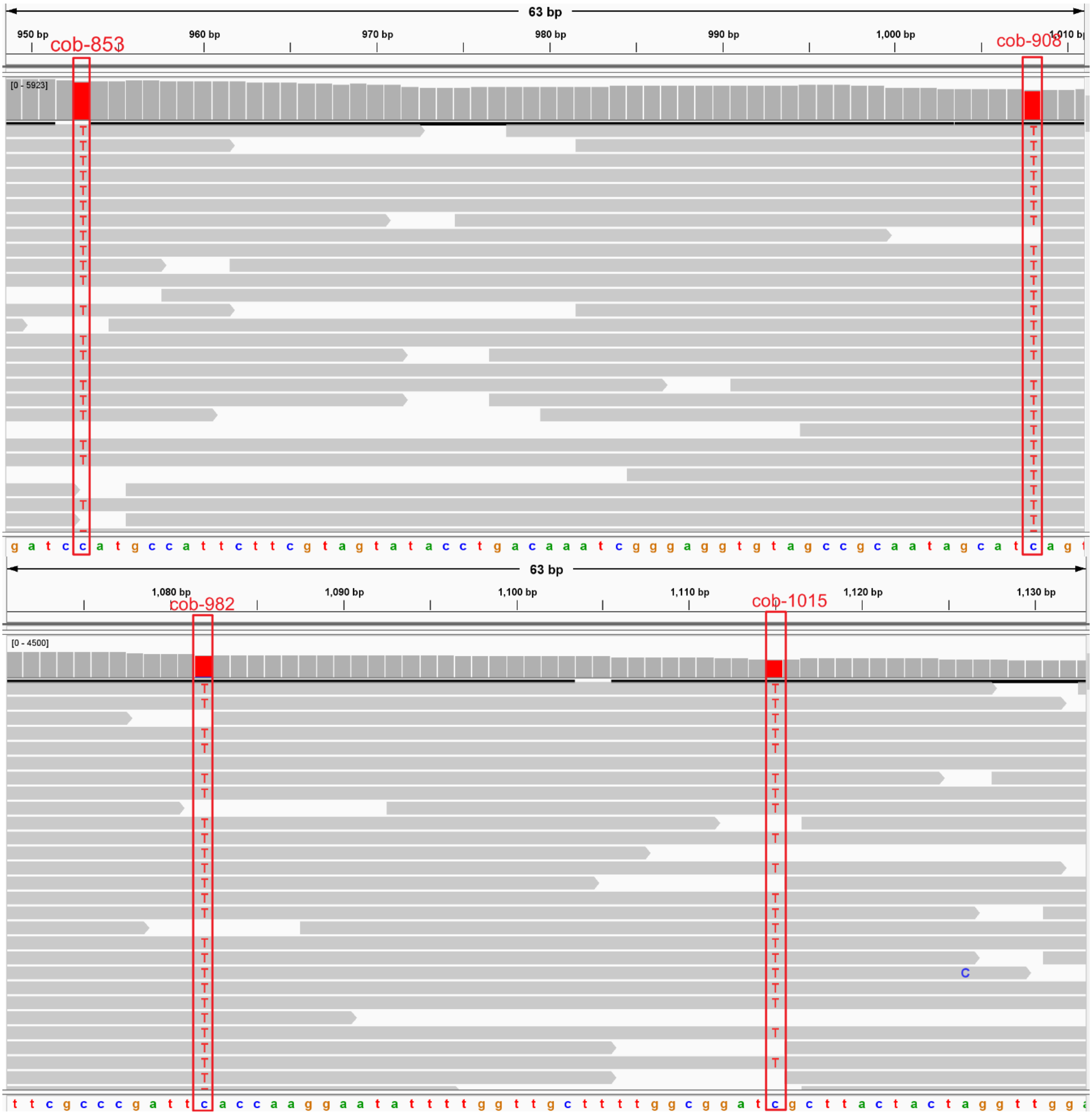

63 bp
950 bp
960 bp
970 bp
980 bp
990 bp
1,000 bp
cob-853
cob-908
[0 - 5923]
63 bp
1,080 bp
1,090 bp
1,100 bp
1,110 bp
1,120 bp
1,130 bp
cob-982
cob-1015
[0 - 4500]

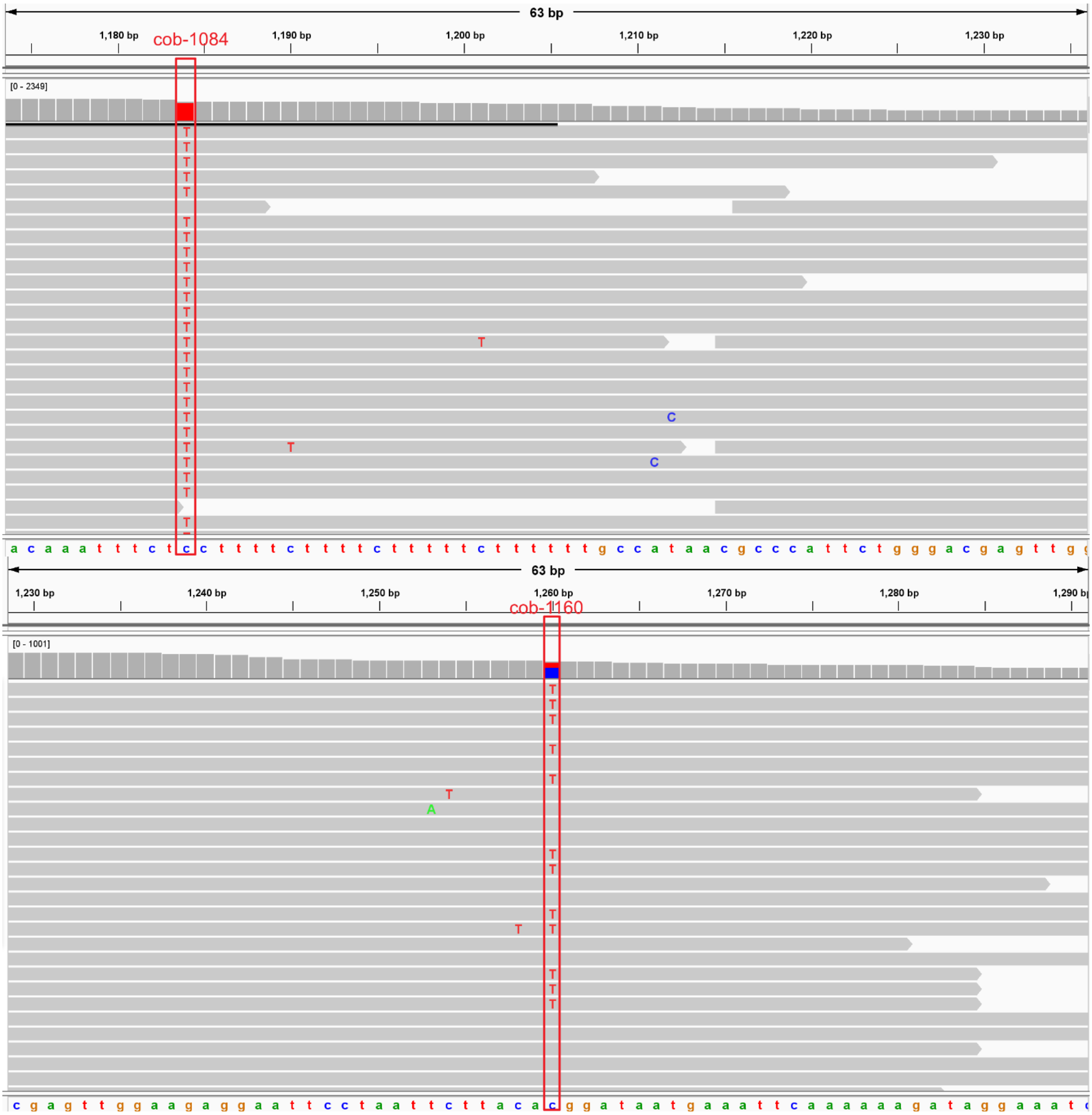
63 bp
1,180 bp
cob-1084
1,190 bp
1,200 bp
1,210 bp
1,220 bp
1,230 bp
[0 - 2349]
63 bp
1,230 bp
1,240 bp
1,250 bp
1,260 bp
cob-1160
1,270 bp
1,280 bp
[0 - 1001]

j alignment of RNA-seq reads to the coding sequence of *cox1*. 15 RNA-seq editing sites: *cox1*-242, 254, 452, 515, 551, 590, 715, 761, 1078, 1186, 1405, 1413, 1433, 1489, and 1499 were highlighted in red squares.

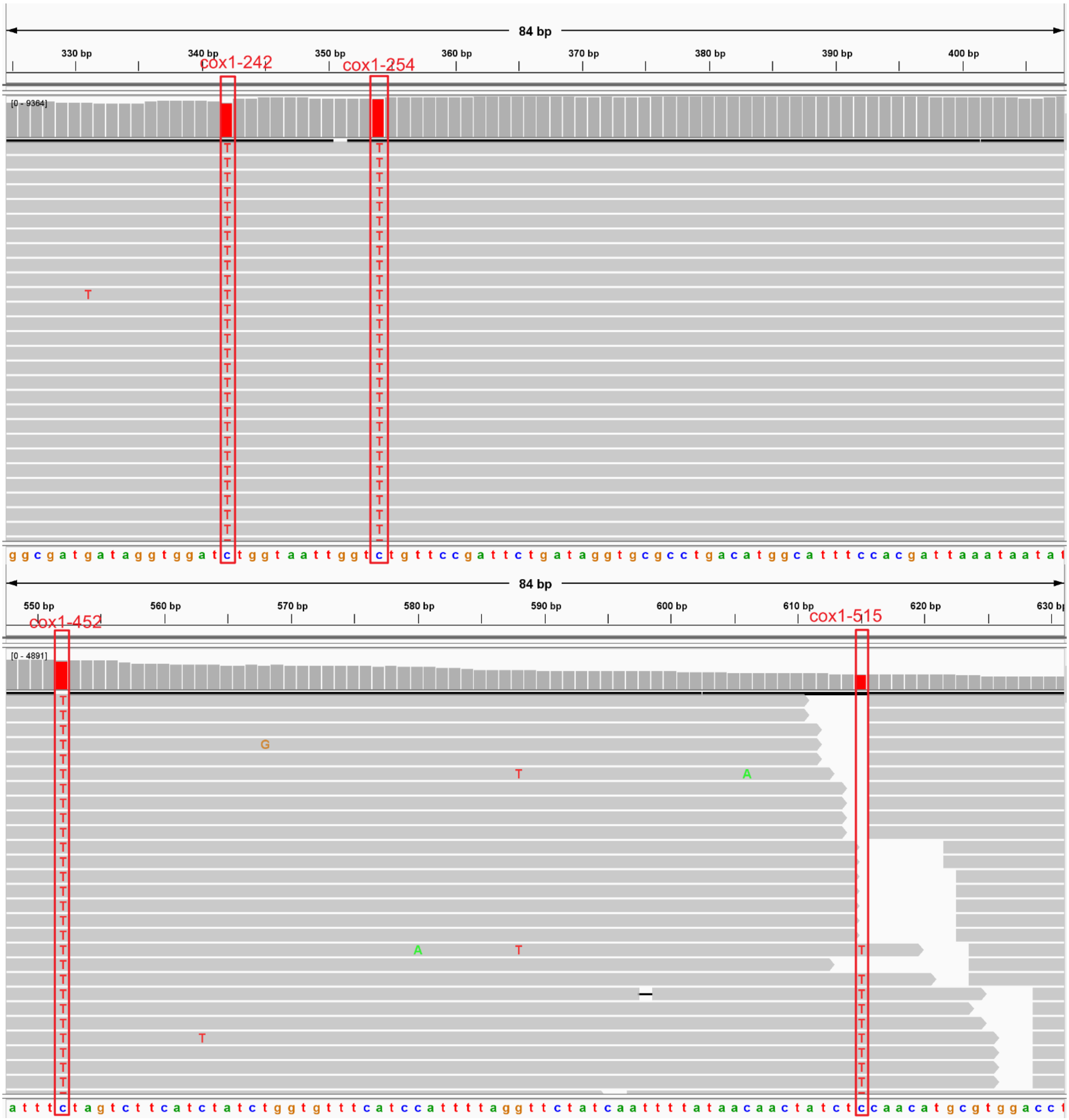

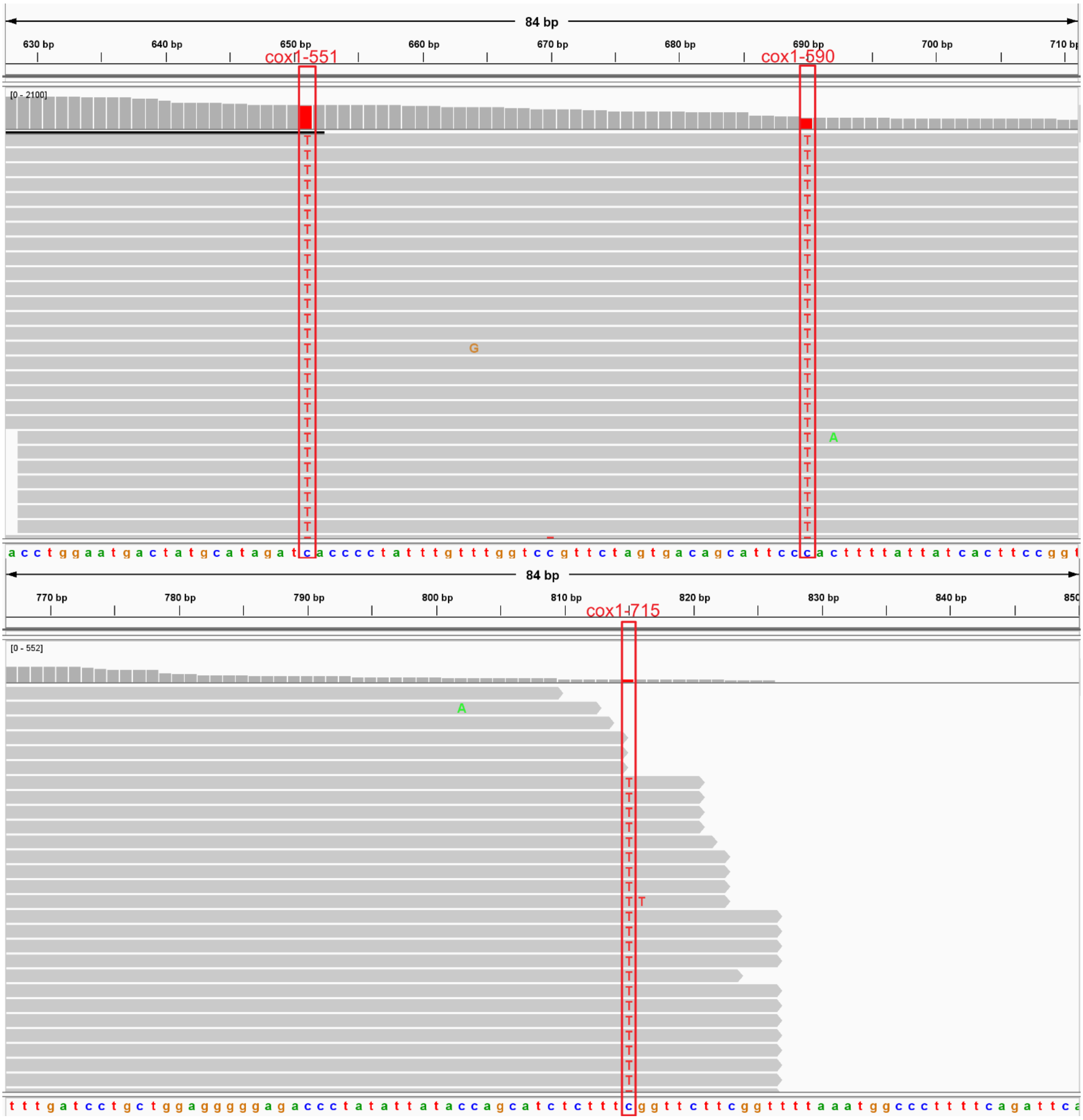
84 bp
630 bp
640 bp
650 bp
660 bp
670 bp
680 bp
690 bp
700 bp
cox1-551
cox1-590
[0 - 2100]
84 bp
770 bp
780 bp
790 bp
800 bp
810 bp
820 bp
830 bp
840 bp
cox1-715
[0 - 552]

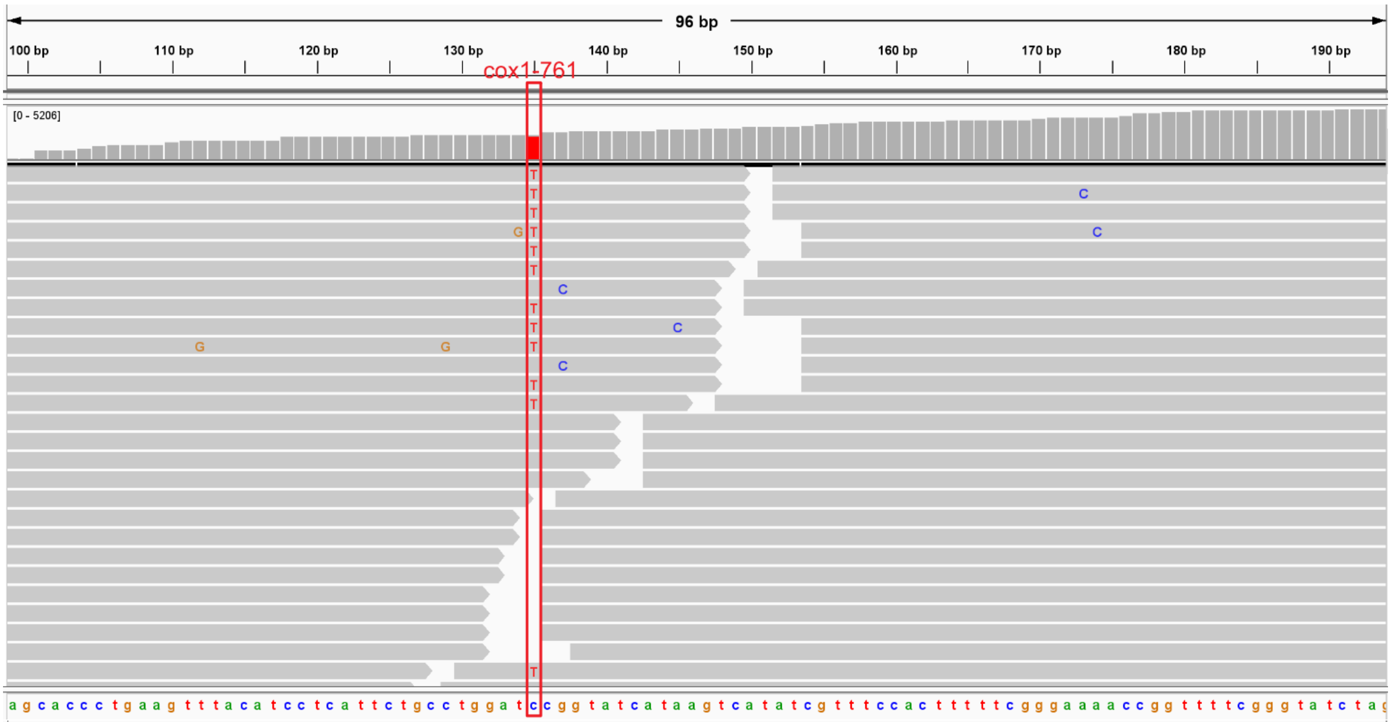
96 bp
100 bp
110 bp
120 bp
130 bp
140 bp
150 bp
160 bp
170 bp
180 bp
190 bp
cox1-1761
[0 - 5206]

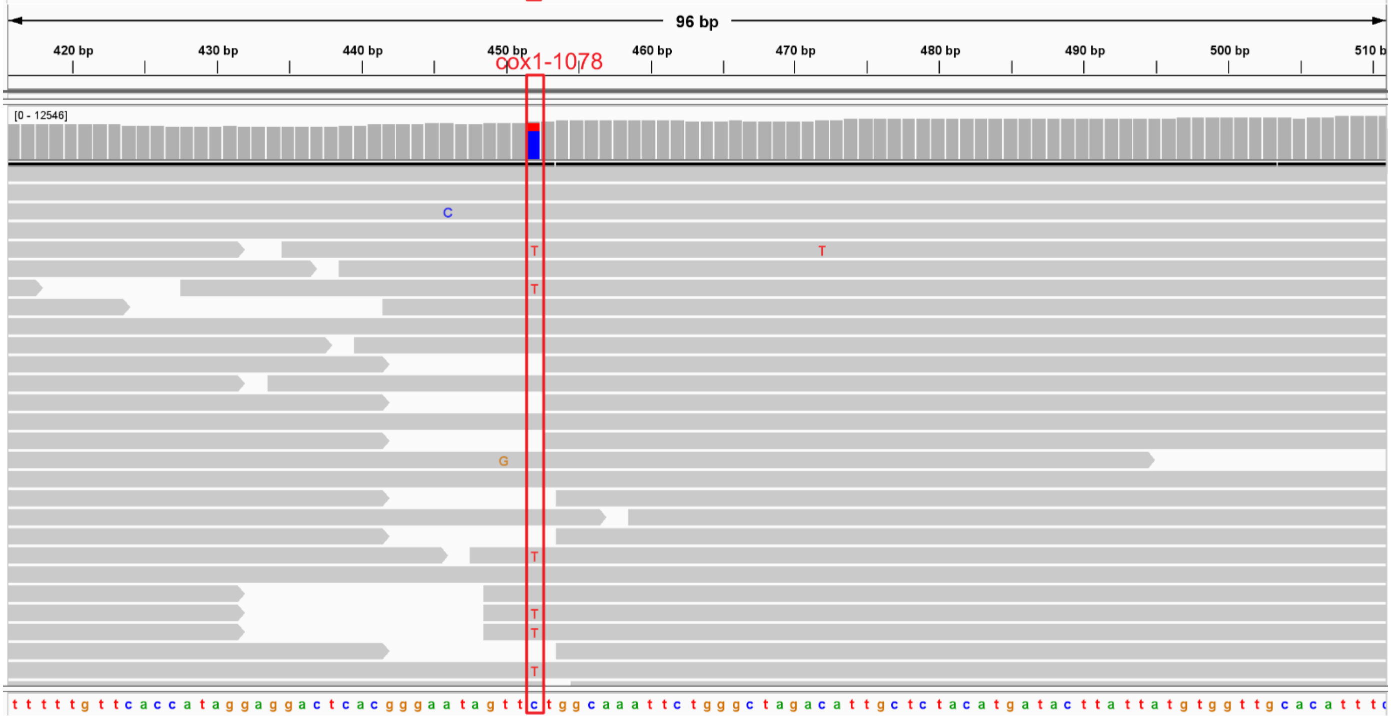
96 bp
420 bp
430 bp
440 bp
450 bp
460 bp
470 bp
480 bp
490 bp
500 bp
cox1-1078
[0 - 12546]

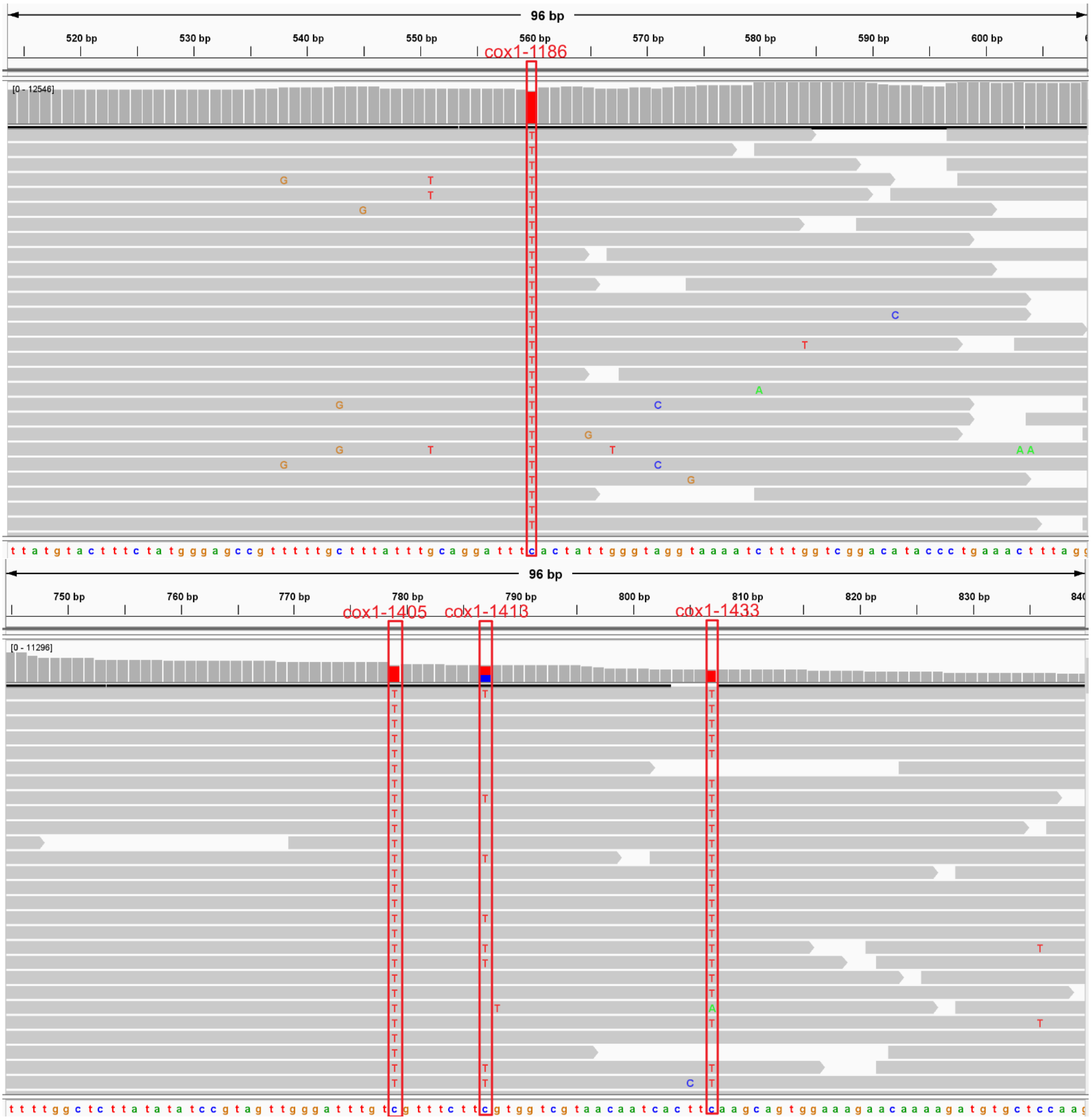
96 bp
520 bp
530 bp
540 bp
550 bp
560 bp
570 bp
580 bp
590 bp
600 bp
cox1-1186
[0 - 12546]
96 bp
750 bp
760 bp
770 bp
780 bp
790 bp
800 bp
810 bp
820 bp
830 bp
cox1-1405
cox1-1413
cox1-1433
[0 - 11296]

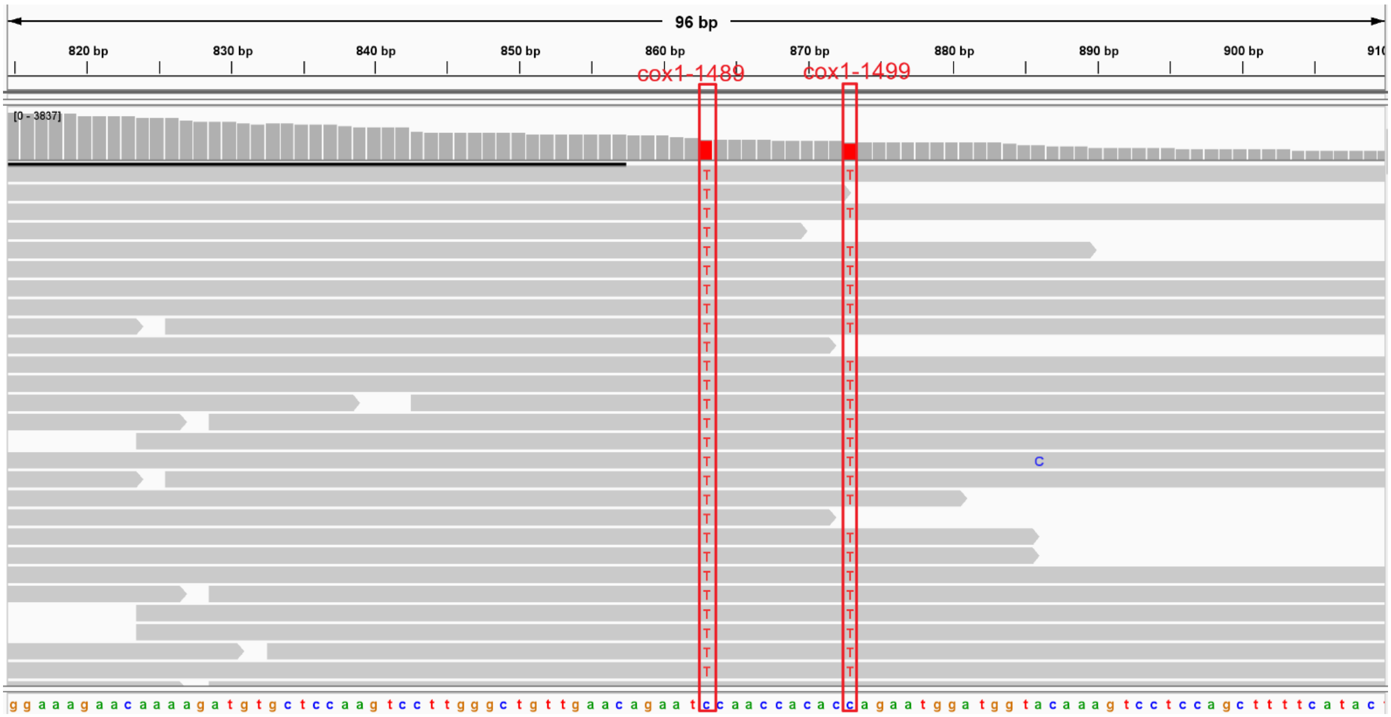
96 bp
820 bp
830 bp
840 bp
850 bp
860 bp
870 bp
880 bp
890 bp
900 bp
cox1-1489
cox1-1499
[0 - 3837]

k alignment of RNA-seq reads to the coding sequence of *cox2.* 10 RNA-seq editing sites: *cox2*-47, 419, 437, 452, 520, 533, 595, 608, 697, and 718 were highlighted in red squares.

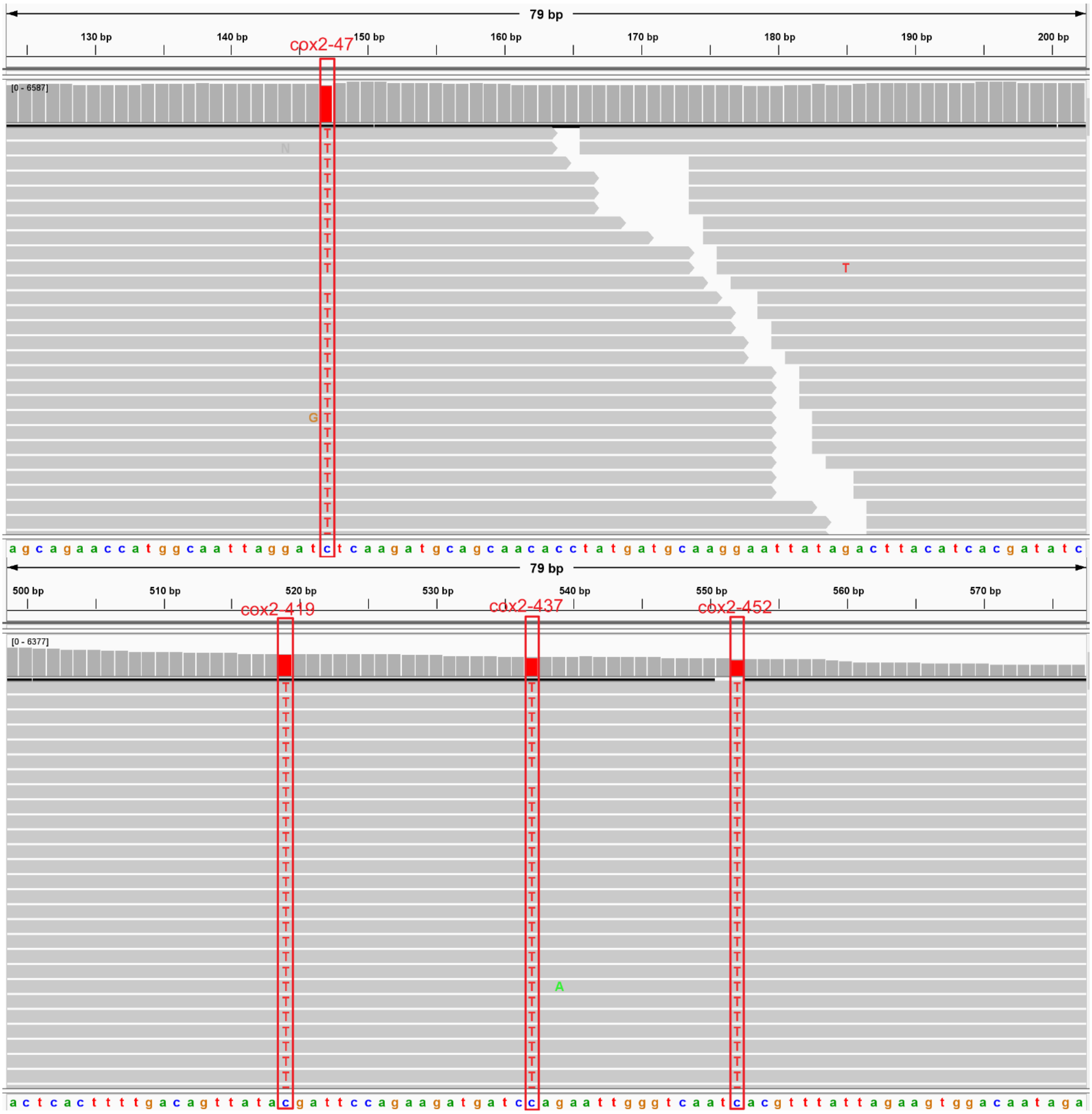

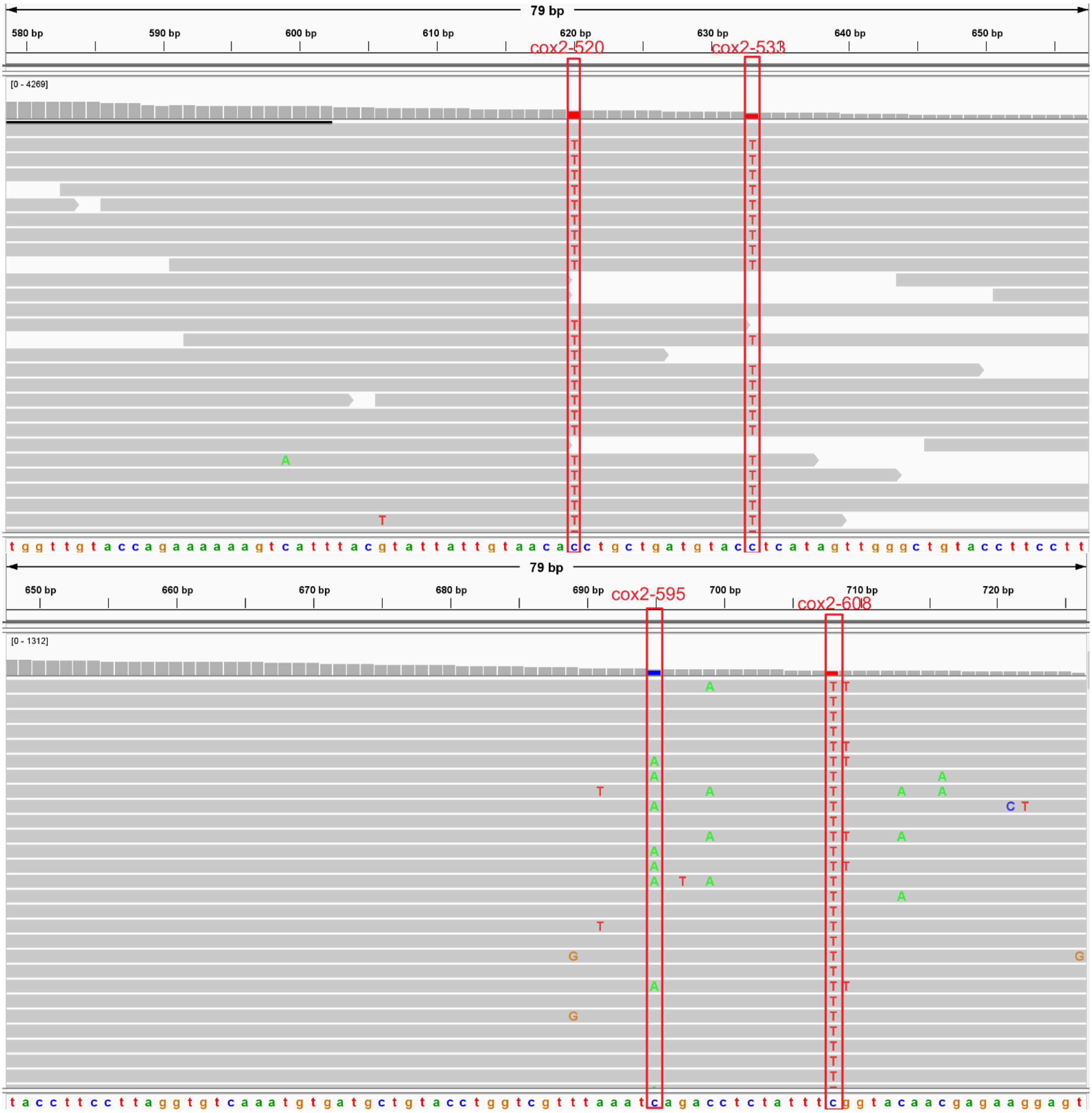
79 bp
580 bp
590 bp
600 bp
610 bp
620 bp
630 bp
640 bp
650 bp
cox2-520
cox2-533
[0 - 4269]
79 bp
650 bp
660 bp
670 bp
680 bp
690 bp
700 bp
710 bp
720 bp
cox2-595
cox2-608
[0 - 1312]

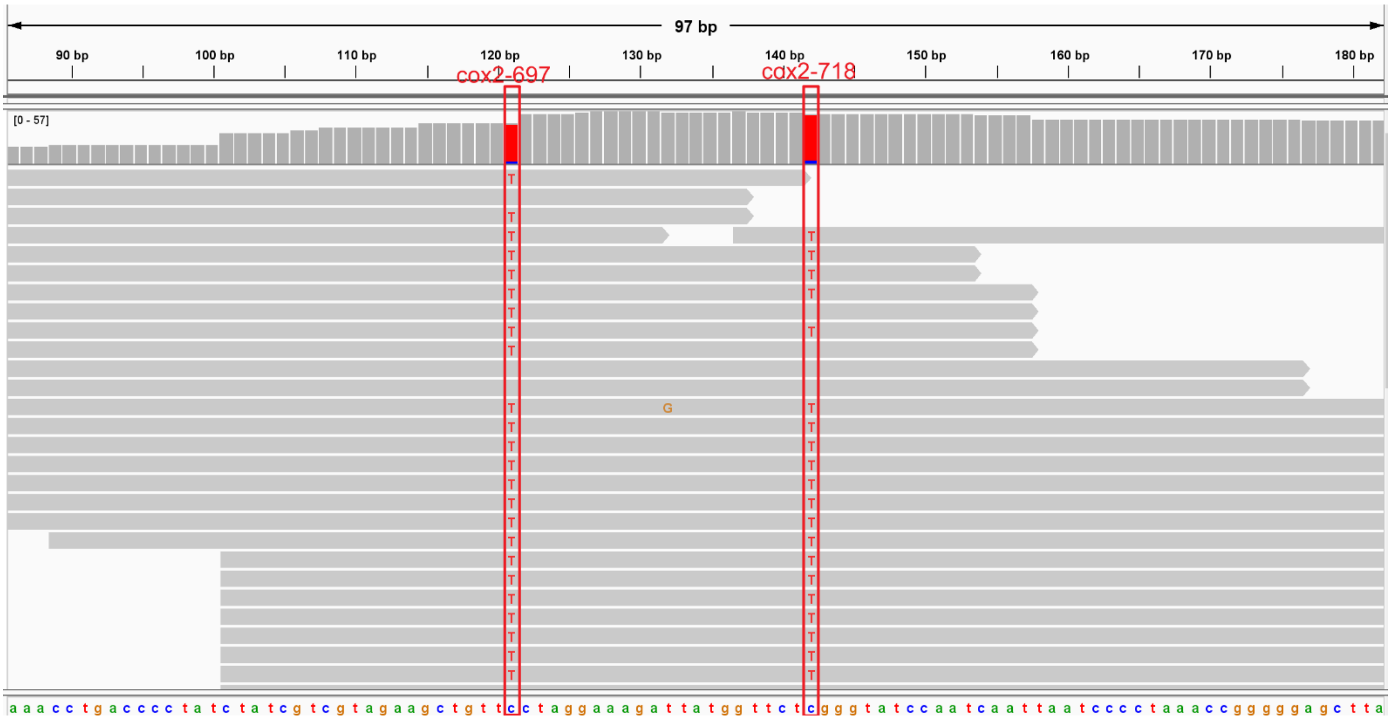
97 bp
90 bp
100 bp
110 bp
120 bp
130 bp
140 bp
150 bp
160 bp
170 bp
180 bp
cox2-697
cox2-718
[0 - 57]

l alignment of RNA-seq reads to the coding sequence of *cox3.* 11 RNA-seq editing sites: *cox3*-174, 245, 304, 311, 314, 419, 422, 566, 567, 754, and 764 were highlighted in red squares.

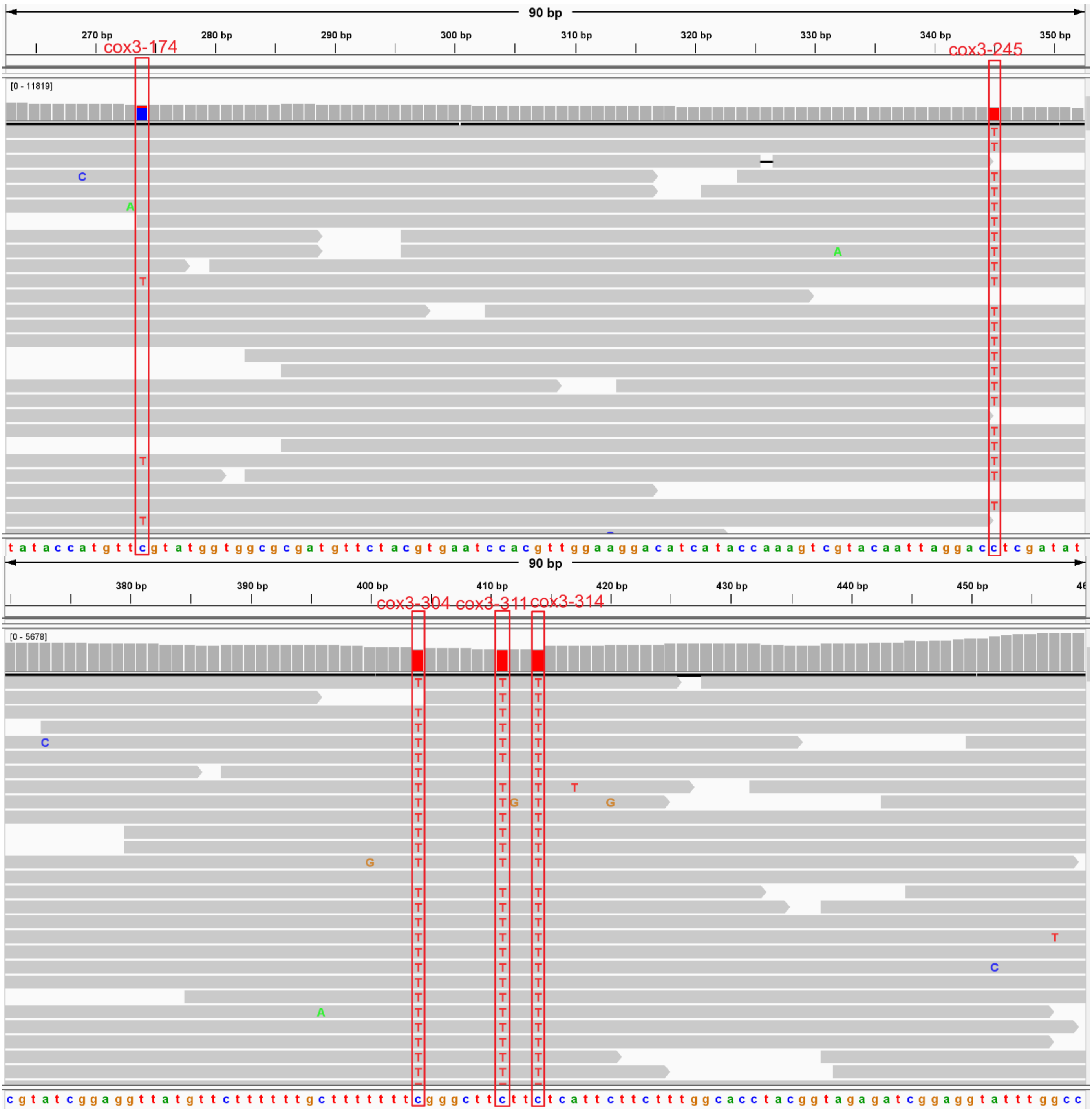

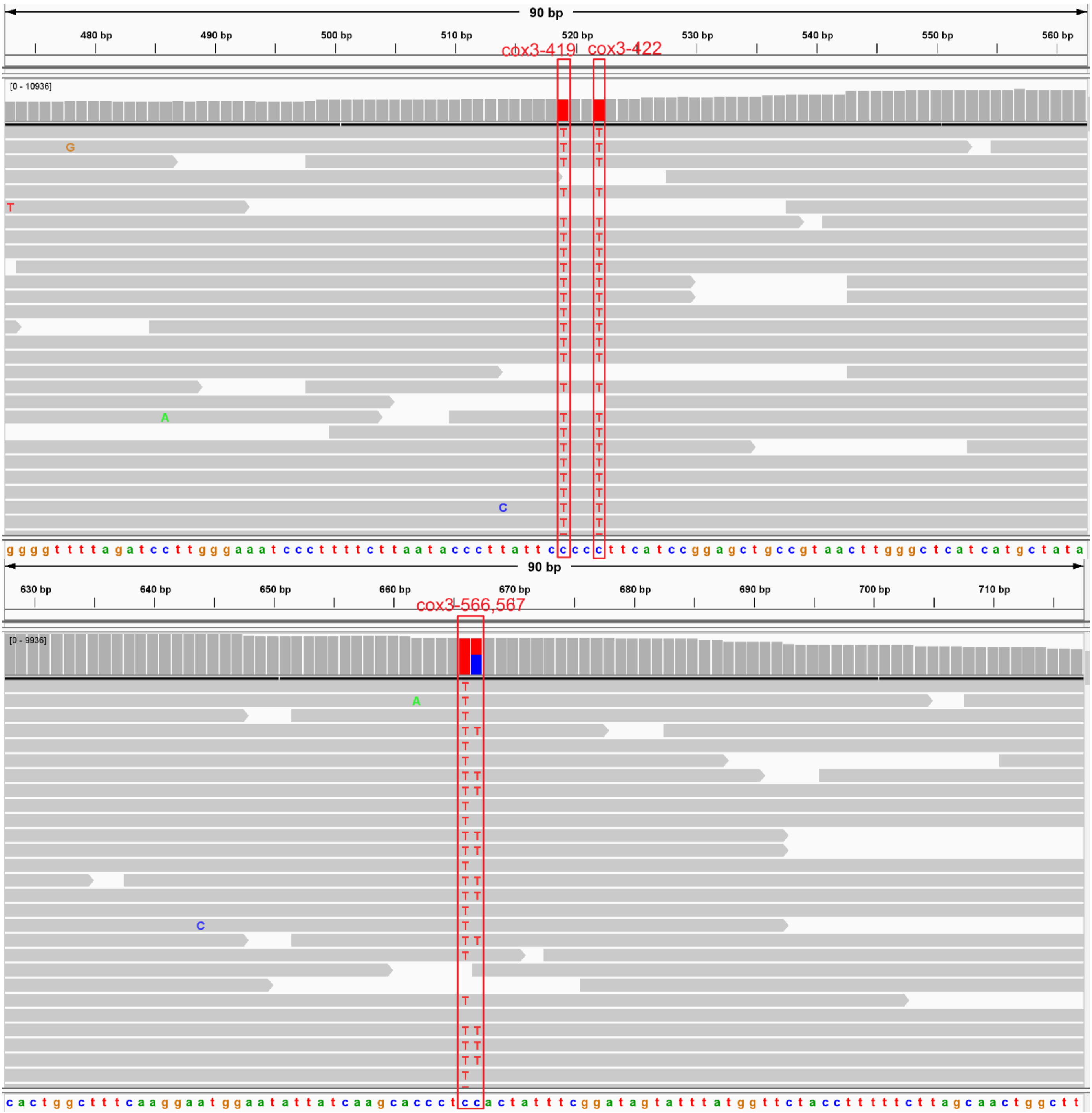
90 bp
480 bp
490 bp
500 bp
510 bp
520 bp
530 bp
540 bp
550 bp
560 bp
cox3-419
cox3-422
[0 - 10936]
90 bp
630 bp
640 bp
650 bp
660 bp
670 bp
680 bp
690 bp
700 bp
710 bp
cox3-566,567
[0 - 9936]

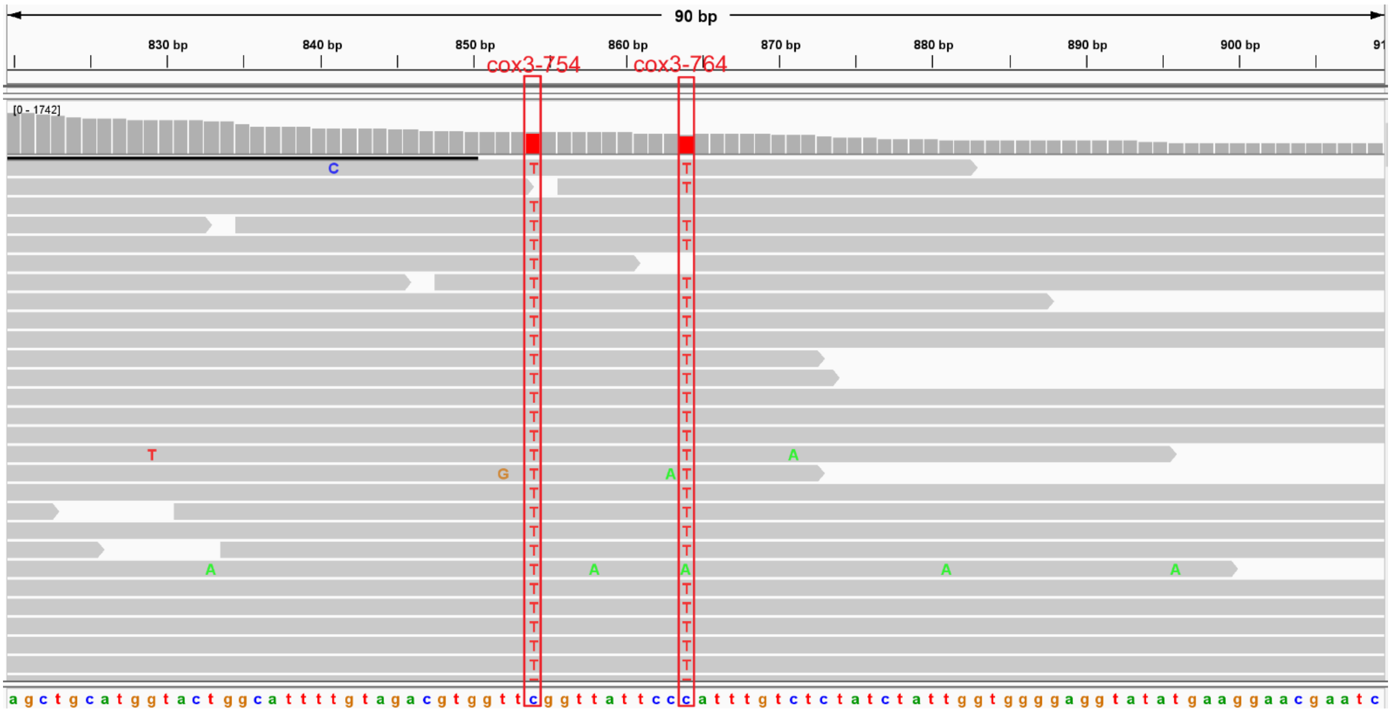
90 bp
830 bp
840 bp
850 bp
860 bp
870 bp
880 bp
890 bp
900 bp
cox3-754
cox3-764
[0 - 1742]

m alignment of RNA-seq reads to the coding sequence of *matR.* 18 RNA-seq editing sites: *matR*-32, 43, 237, 254, 258, 326, 413, 1400, 1522, 1524, 1658, 1679, 1699, 1713, 1735, 1766, 1805, and1823 were highlighted in red squares.

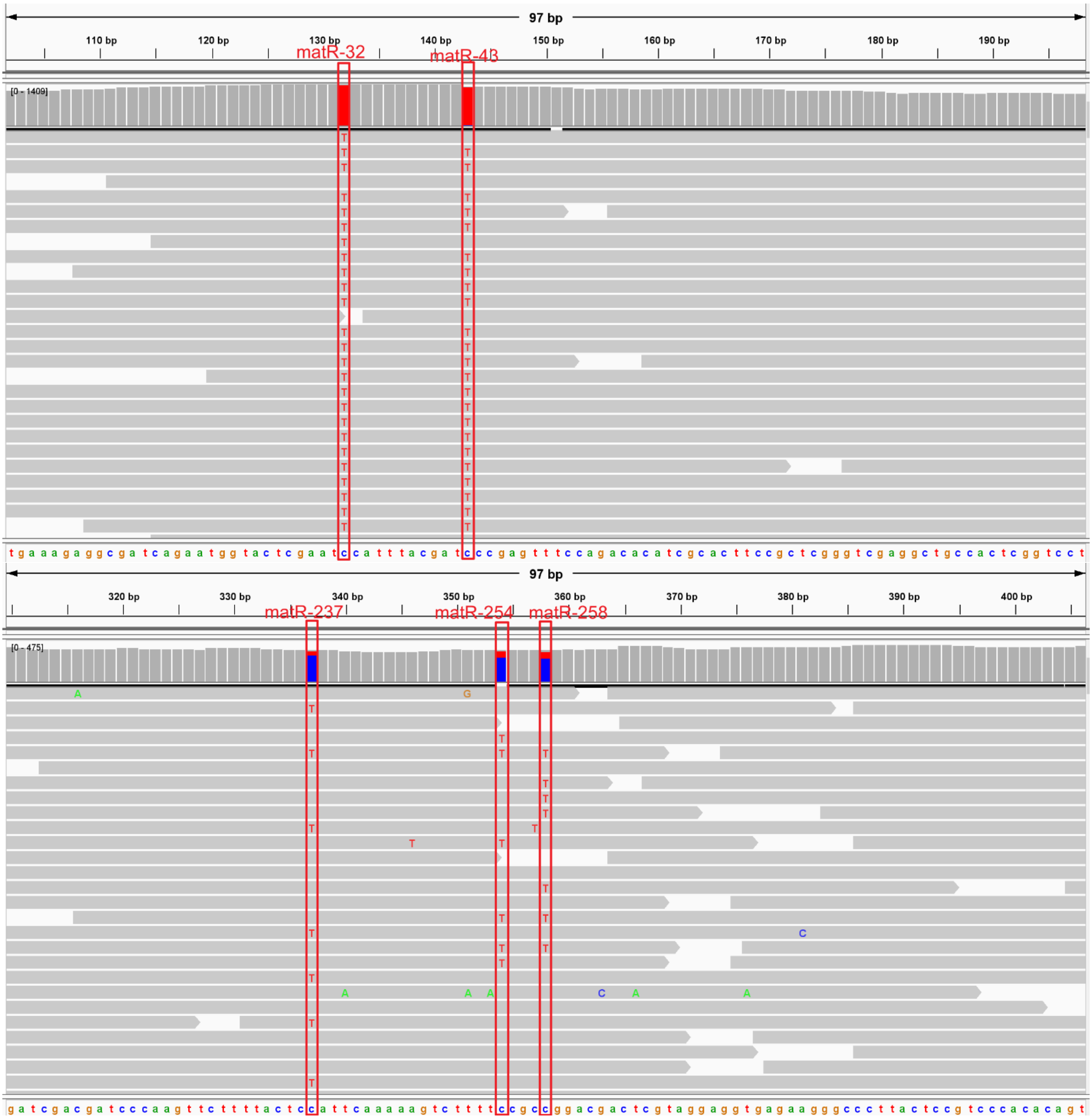

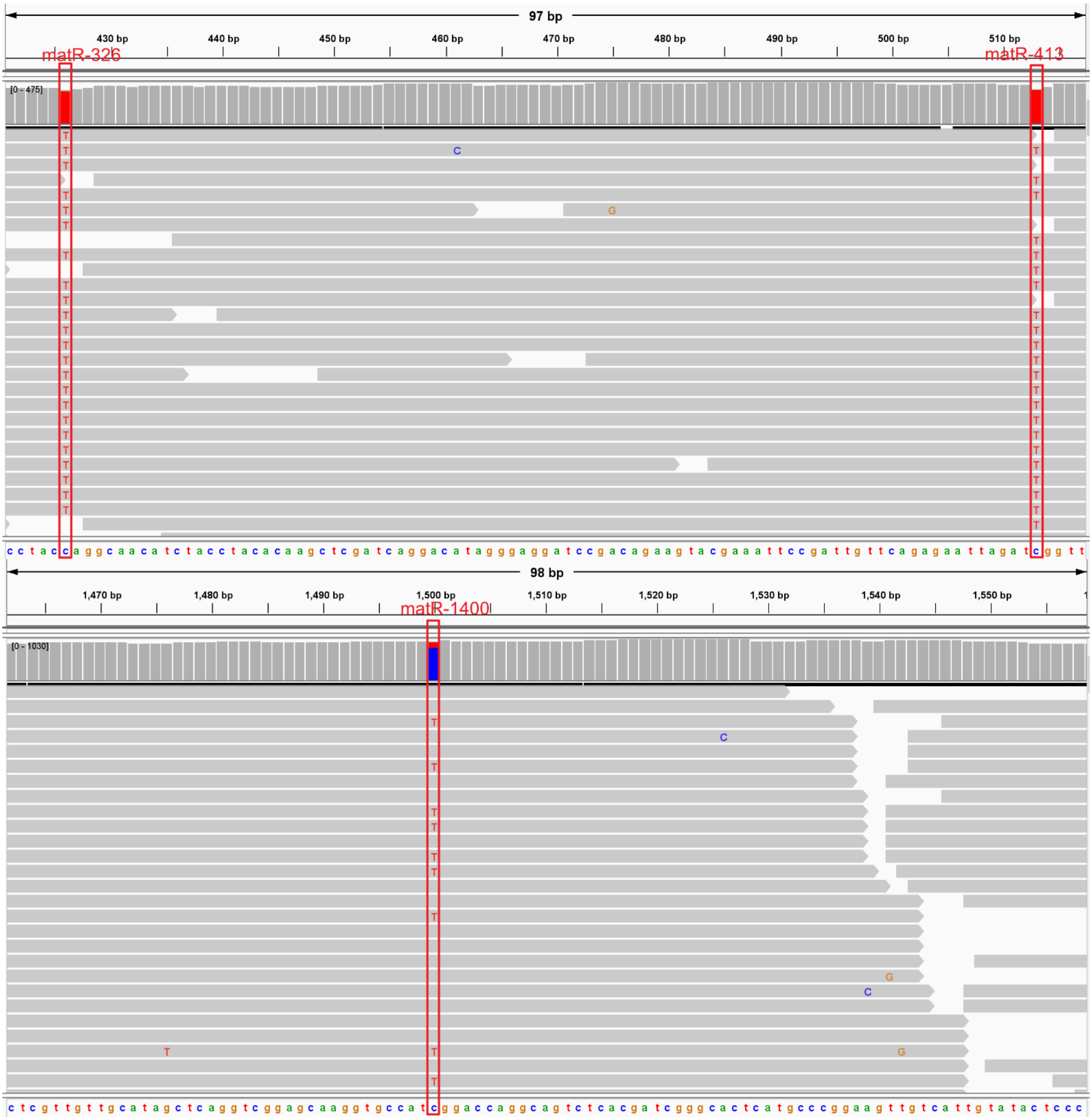
97 bp
430 bp
440 bp
450 bp
460 bp
470 bp
480 bp
490 bp
500 bp
510 bp
matR-326
matR-413
[0 - 475]
98 bp
1,470 bp
1,480 bp
1,490 bp
1,500 bp
1,510 bp
1,520 bp
1,530 bp
1,540 bp
1,550 bp
matR-1400
[0 - 1030]

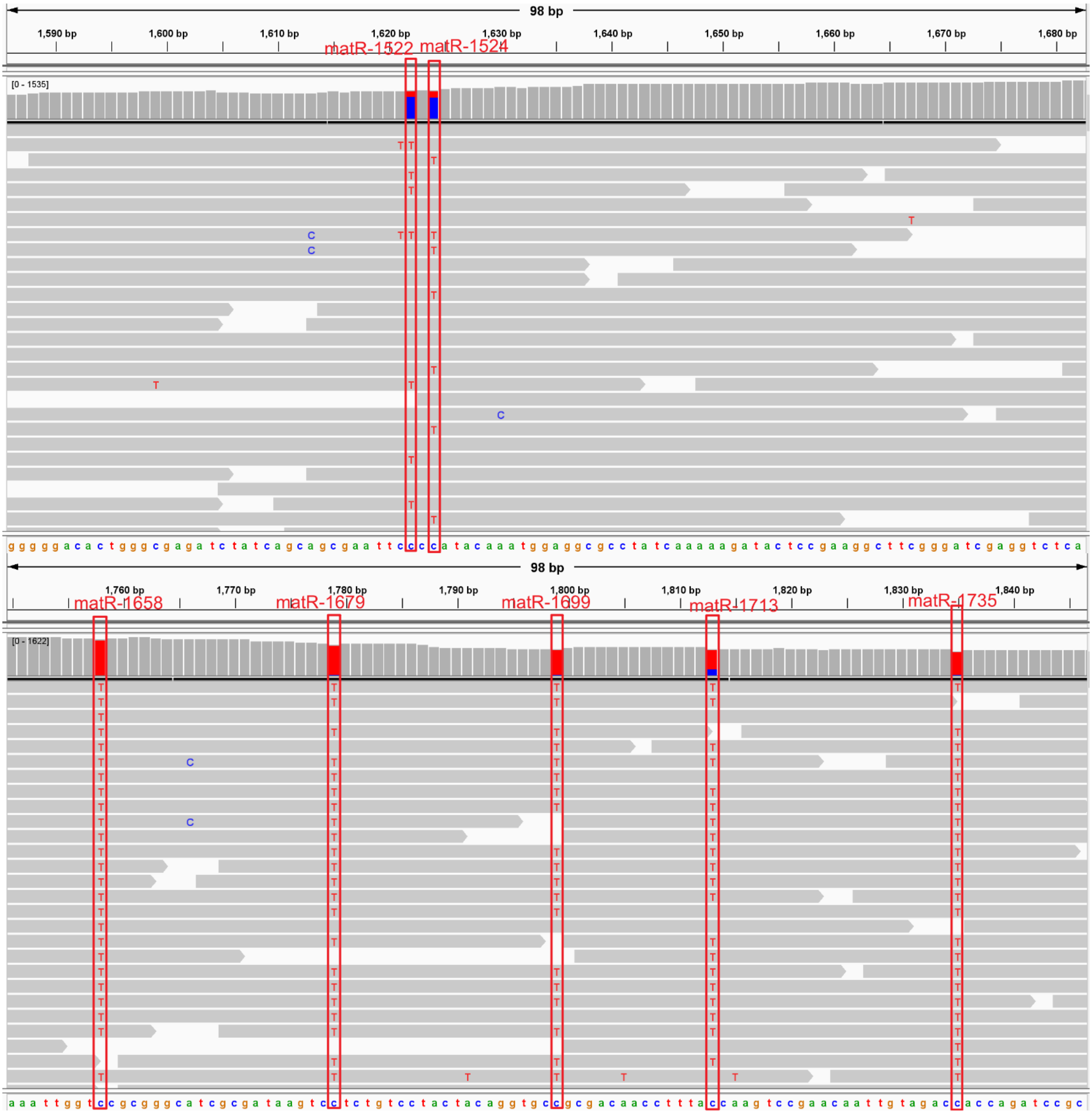

98 bp
1,590 bp
1,600 bp
1,610 bp
1,620 bp
1,630 bp
1,640 bp
1,650 bp
1,660 bp
1,670 bp
1,680 bp
matR-1522
matR-1524
[0 - 1535]
98 bp
1,760 bp
1,770 bp
1,780 bp
1,790 bp
1,800 bp
1,810 bp
1,820 bp
1,830 bp
1,840 bp
matR-1658
matR-1679
matR-1699
matR-1713
matR-1735
[0 - 1622]

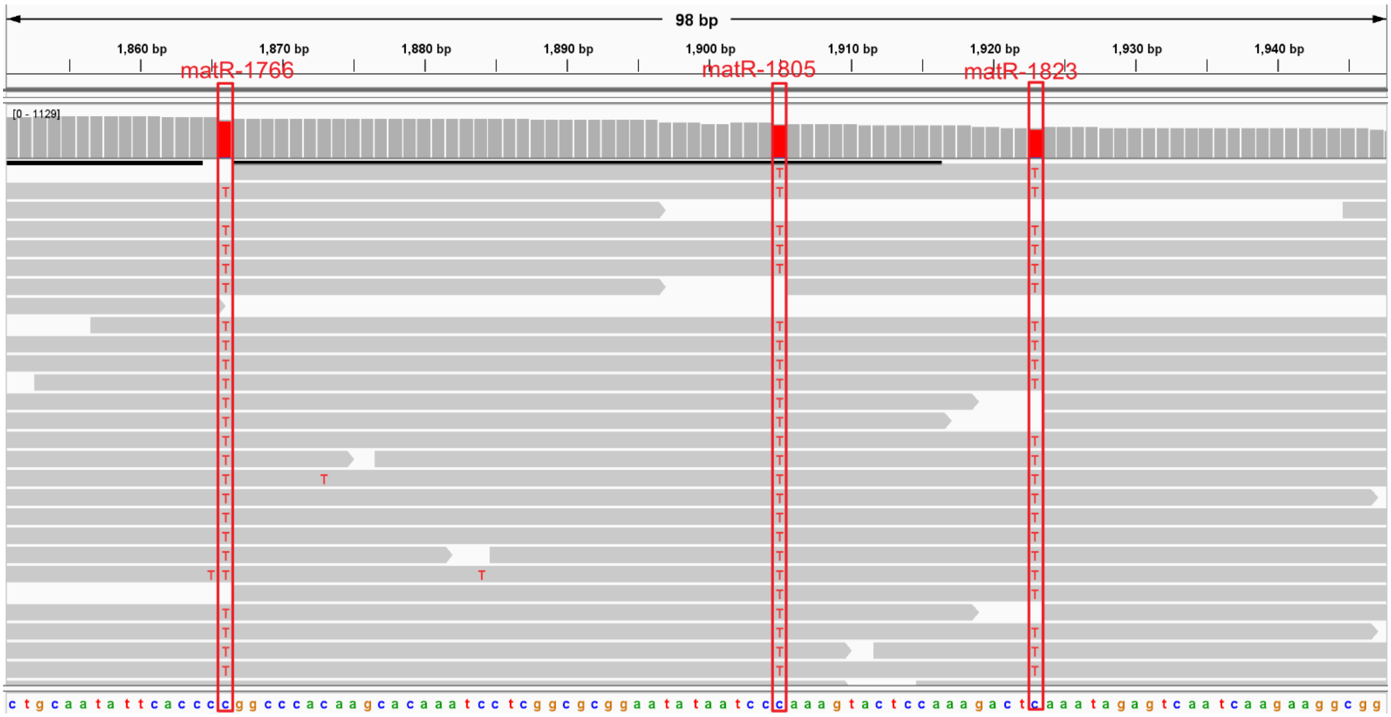
98 bp
1,860 bp
1,870 bp
1,880 bp
1,890 bp
1,900 bp
1,910 bp
1,920 bp
1,930 bp
1,940 bp
matR-1766
matR-1805
matR-1823
[0 - 1129]
c t g c a a t a t t c a c c c c g g c c c a c a a g c a c a a a t c c t c g g c g c g g a a t a t a a t c c c a a a g t a c t c c a a a g a c t c a a a t a g a g t c a a t c a a g a a g g c g g

n alignment of RNA-seq reads to the coding sequence of *mttB.* 32 RNA-seq editing sites: *mttB*-16, 26, 64, 100, 112, 128, 131, 178, 188, 201, 202, 236, 262, 328, 331, 344, 346, 373, 376, 379, 407, 472, 497, 505, 541, 554, 578, 610, 616, 667, 672, and 713 were highlighted in red squares.

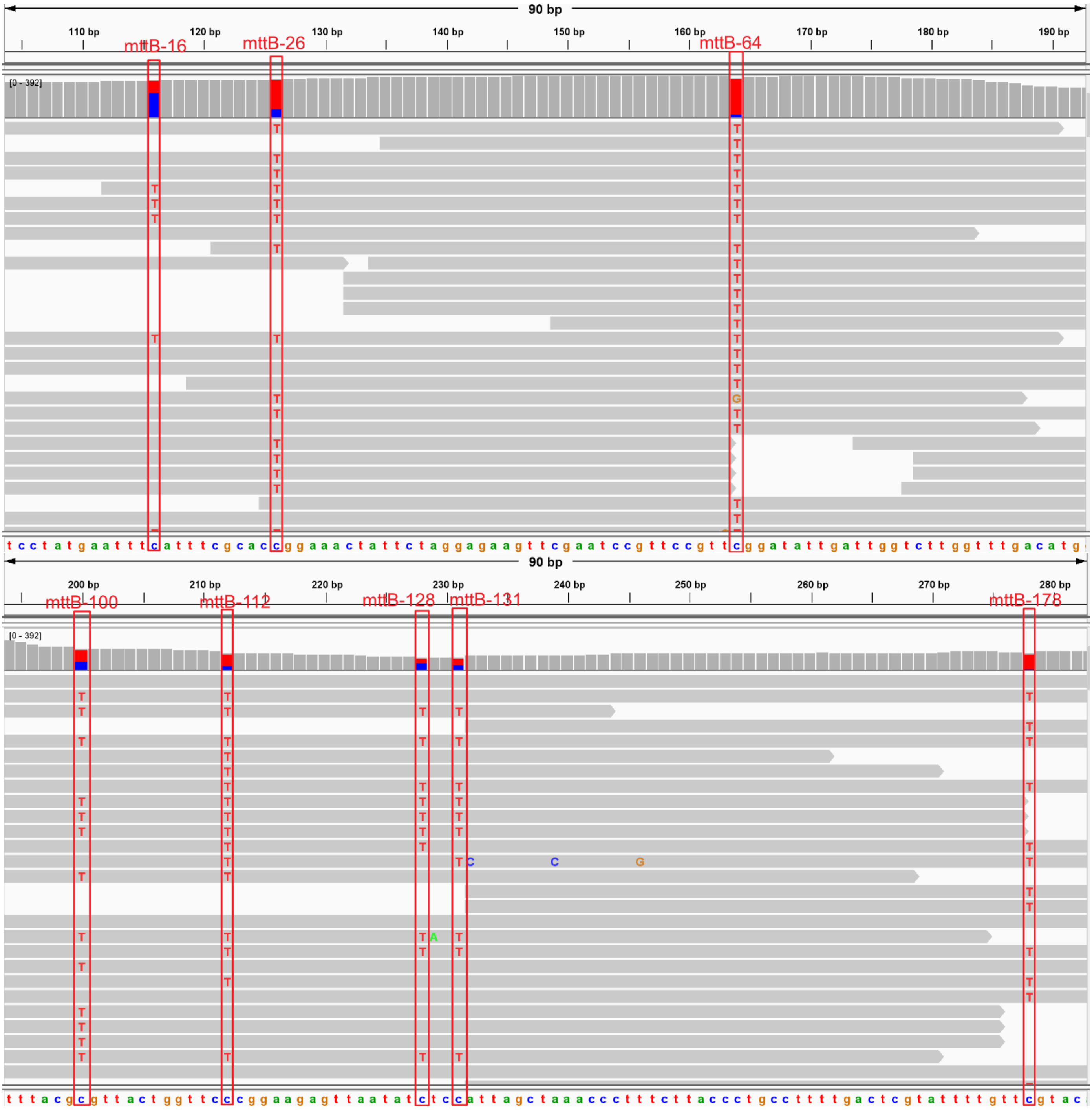

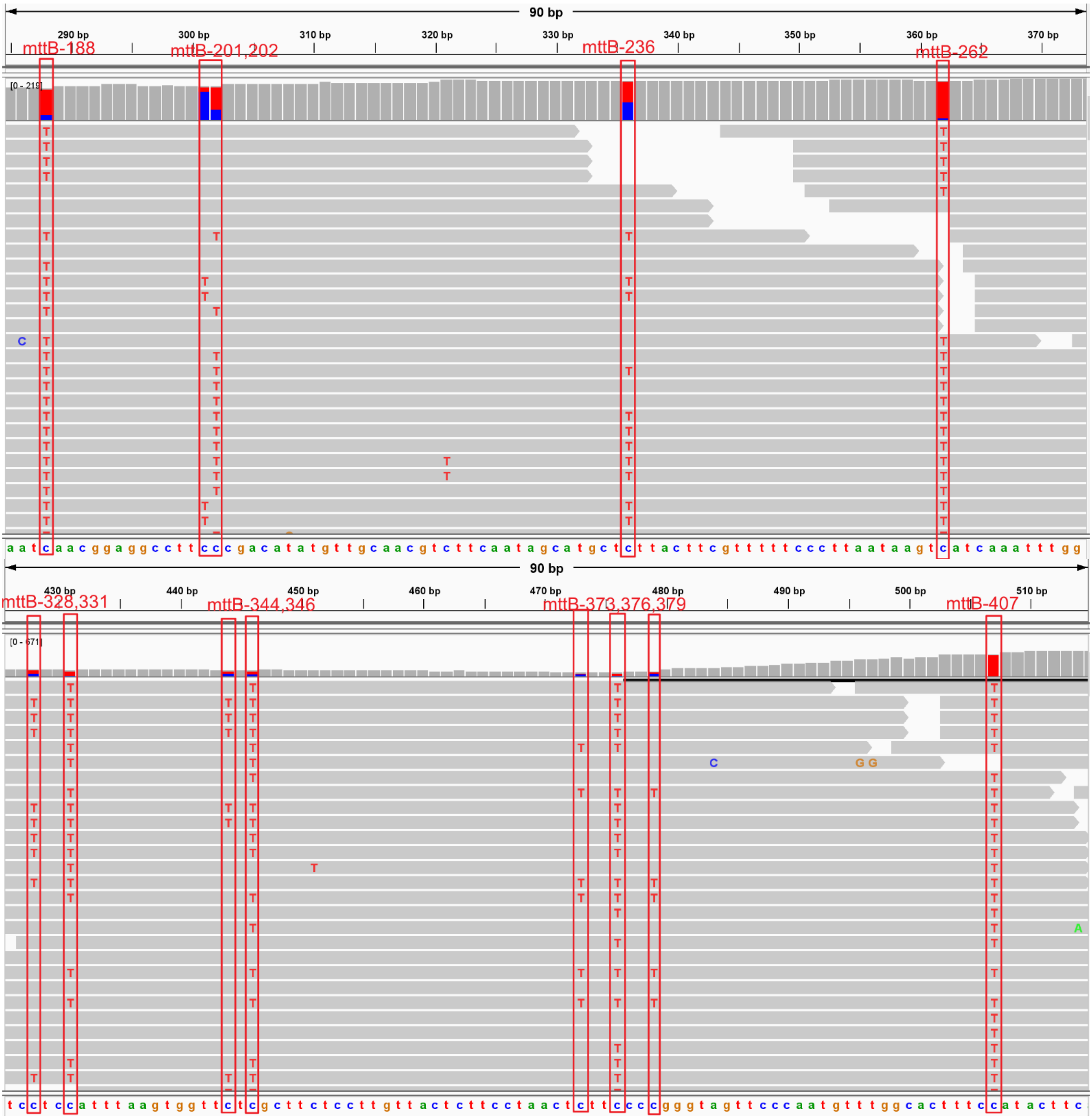
90 bp
290 bp
300 bp
310 bp
320 bp
330 bp
340 bp
350 bp
360 bp
370 bp
mttB-188
mttB-201,202
mttB-236
mttB-262
90 bp
430 bp
440 bp
450 bp
460 bp
470 bp
480 bp
490 bp
500 bp
510 bp
mttB-328,331
mttB-344,346
mttB-373,376,379
mttB-407

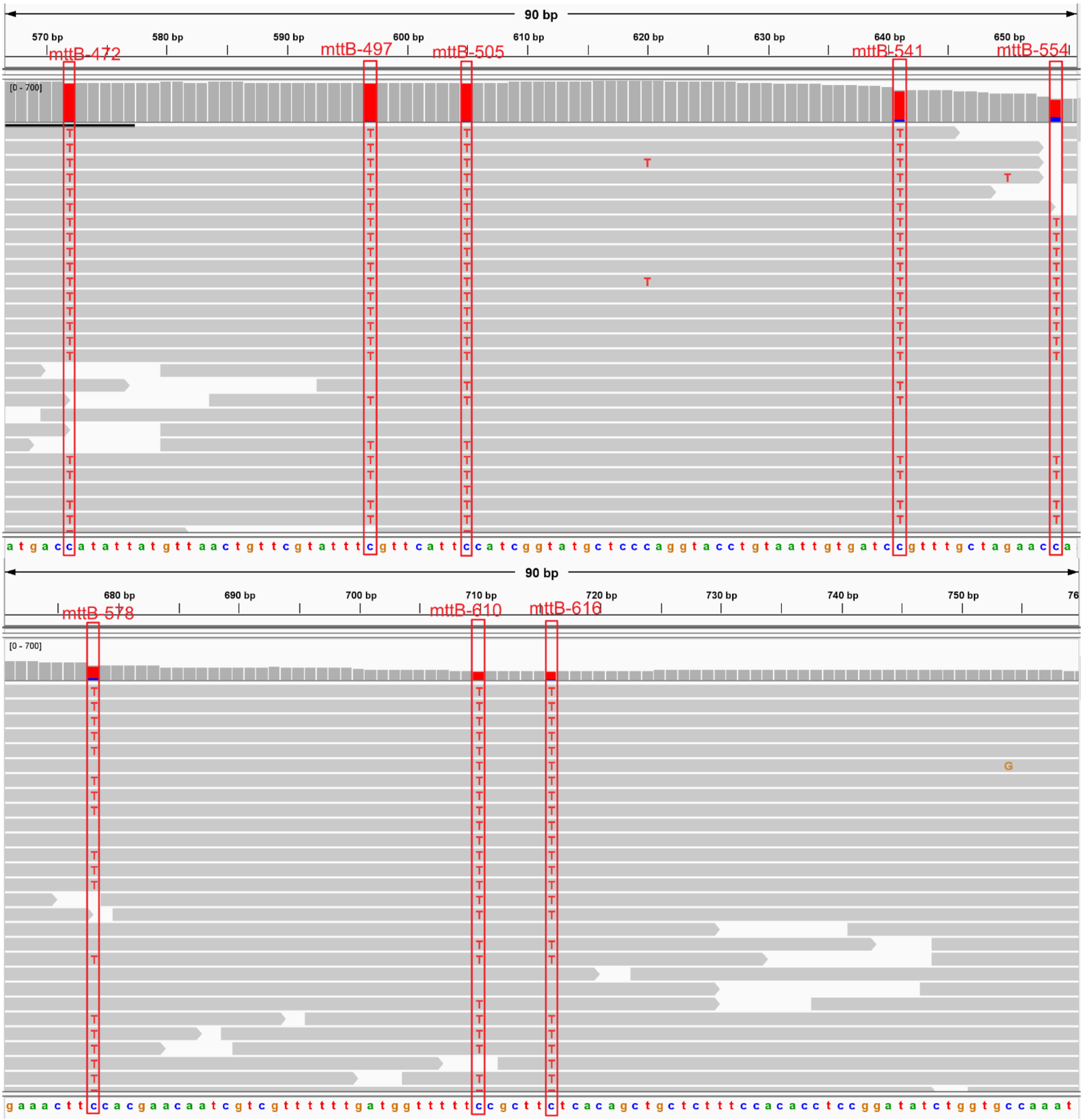
90 bp
570 bp
580 bp
590 bp
600 bp
610 bp
620 bp
630 bp
640 bp
650 bp
mttB-472
mttB-497
mttB-505
mttB-541
mttB-554
[0 - 700]
a t g a c c a t a t t a t g t t a a c t g t t c g t a t t t c g t t c a t t c c a t c g g t a t g c t c c c a g g t a c c t g t a a t t g t g a t c c g t t t g c t a g a a c c a
90 bp
680 bp
690 bp
700 bp
710 bp
720 bp
730 bp
740 bp
750 bp
mttB-578
mttB-610
mttB-616
[0 - 700]
g a a a c t t c c a c g a a c a a t c g t c g t t t t t g a t g g t t t t t c c g c t t c t c a c a g c t g c t c t t t c c a c a c c t c c g g a t a t c t g g t g c c a a a t

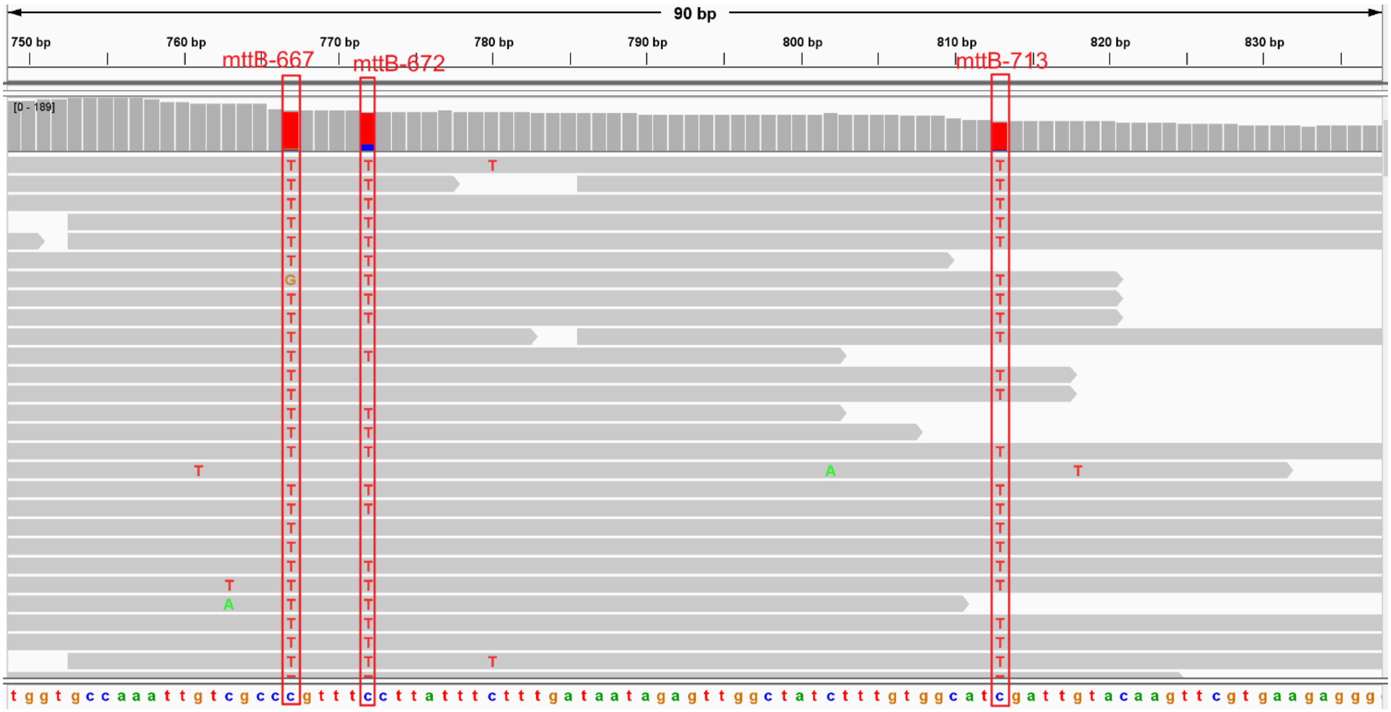
90 bp
750 bp
760 bp
770 bp
780 bp
790 bp
800 bp
810 bp
820 bp
830 bp
mttB-667
mttB-672
mttB-713
[0 - 189]

o alignment of RNA-seq reads to the coding sequence of *nad1.* 24 RNA-seq editing sites: *nad1*-215, 265, 307, 308, 376, 401, 436, 490, 492, 493, 500, 536, 635, 725, 734, 740, 743, 755, 779, 792, 823, 898, 909, 928 were highlighted in red squares.

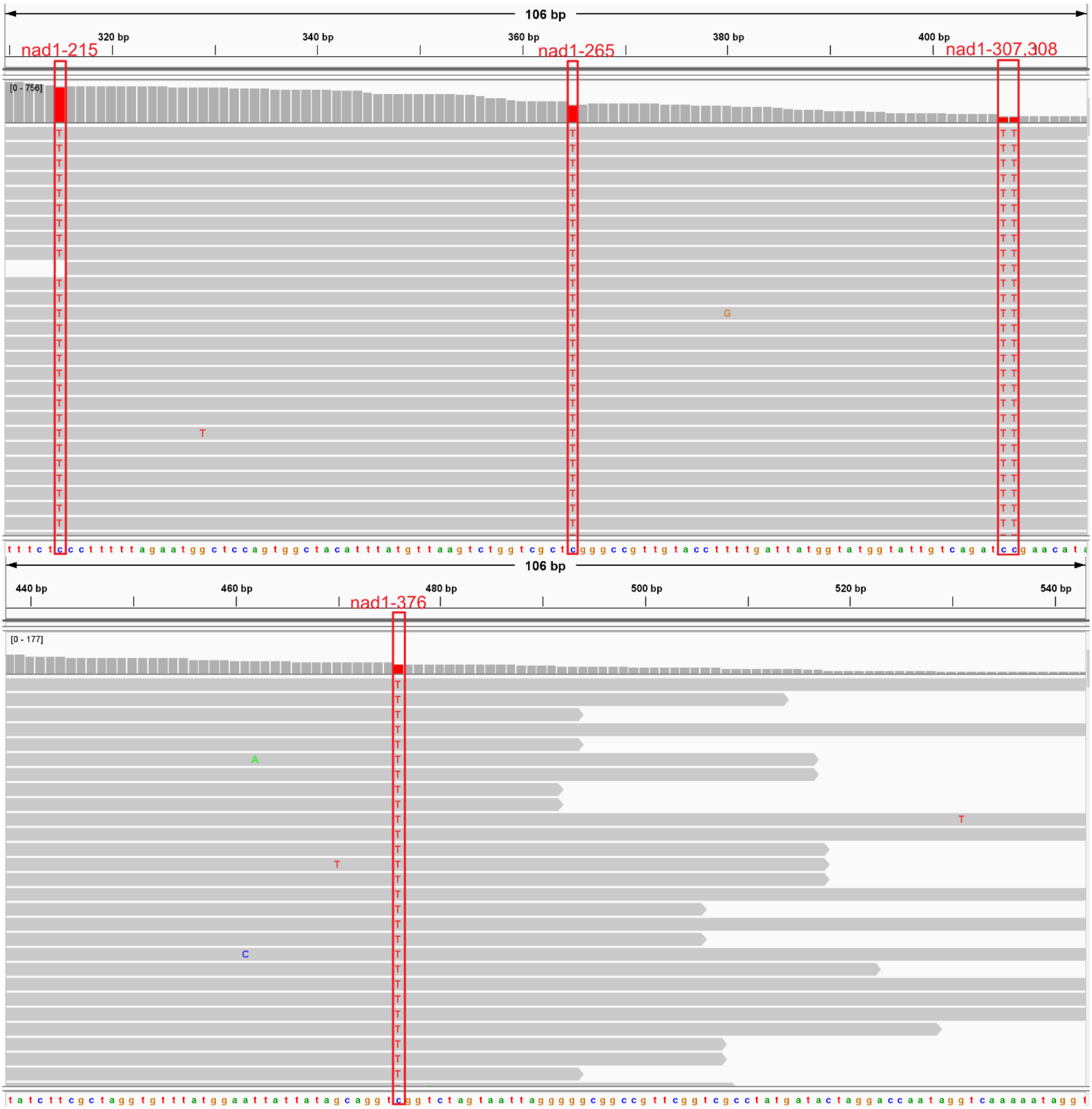

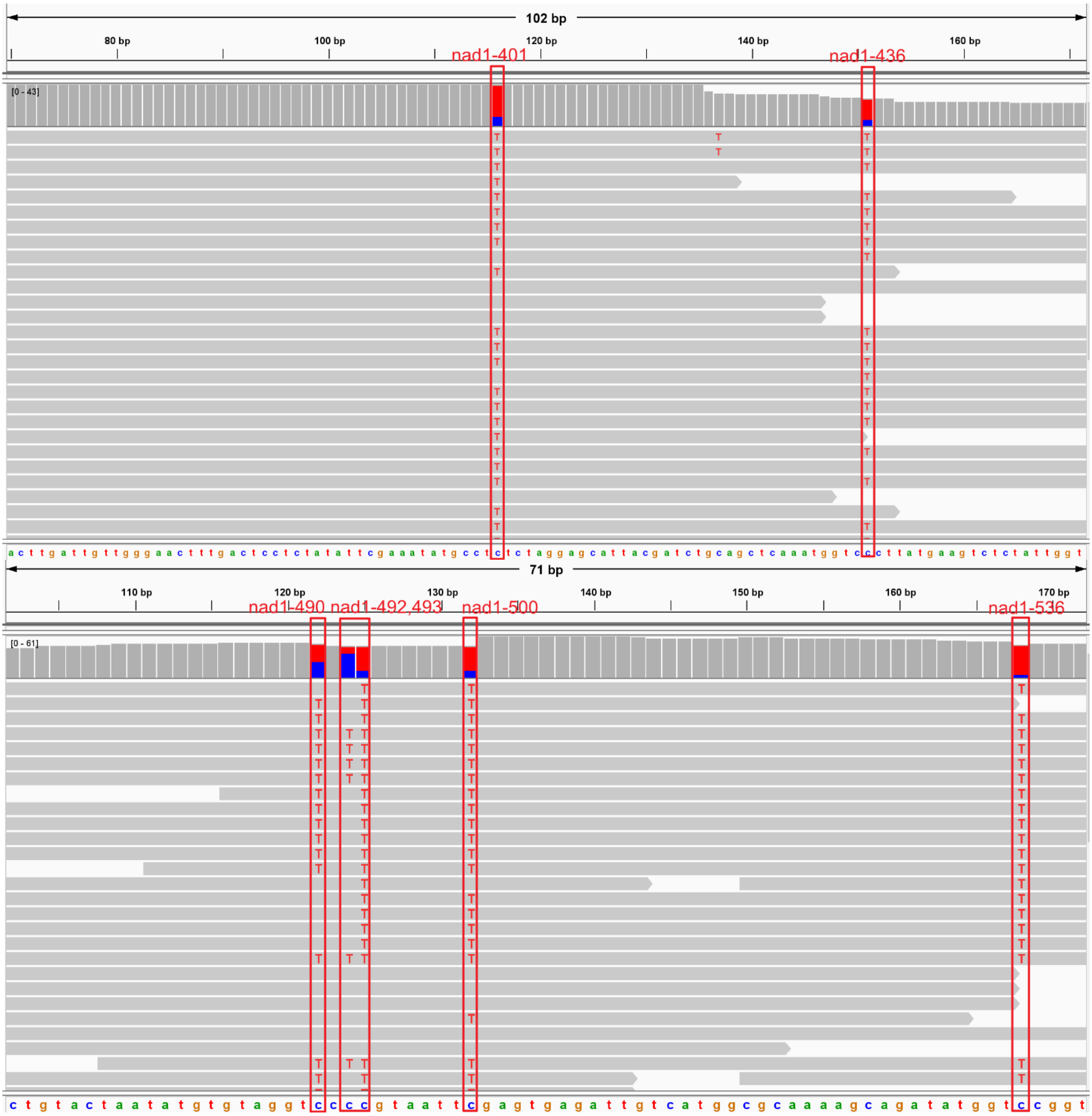
102 bp
80 bp
100 bp
120 bp
140 bp
160 bp
nad1-401
nad1-436
[0 - 43]
71 bp
110 bp
120 bp
130 bp
140 bp
150 bp
160 bp
170 bp
nad1-490
nad1-492,493
nad1-500
nad1-536
[0 - 61]

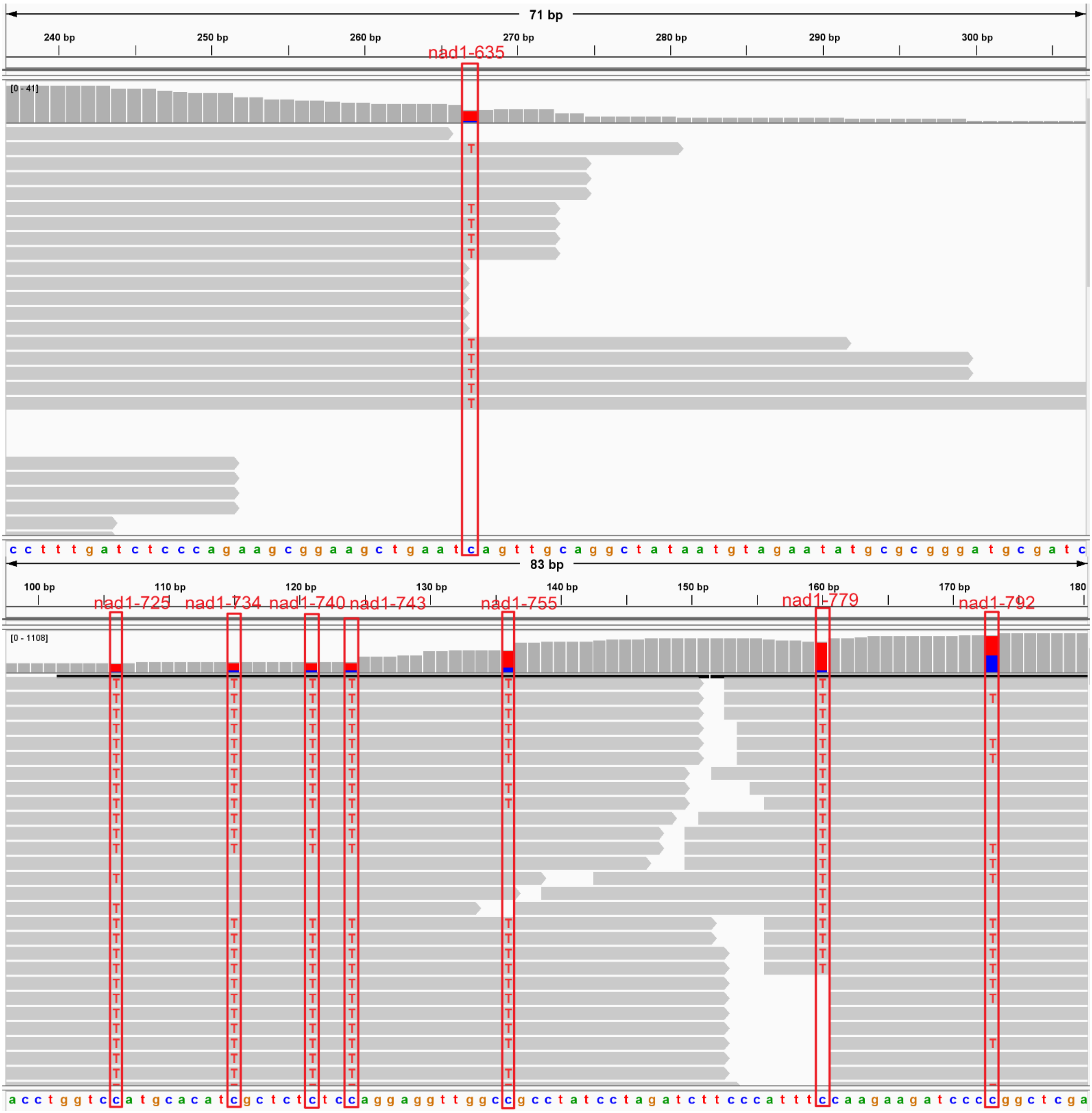

71 bp
240 bp
250 bp
260 bp
270 bp
280 bp
290 bp
300 bp
nad1-635
[0 - 41]
83 bp
100 bp
110 bp
120 bp
130 bp
140 bp
150 bp
160 bp
170 bp
180
nad1-725
nad1-734
nad1-740
nad1-743
nad1-755
nad1-779
nad1-792
[0 - 1108]

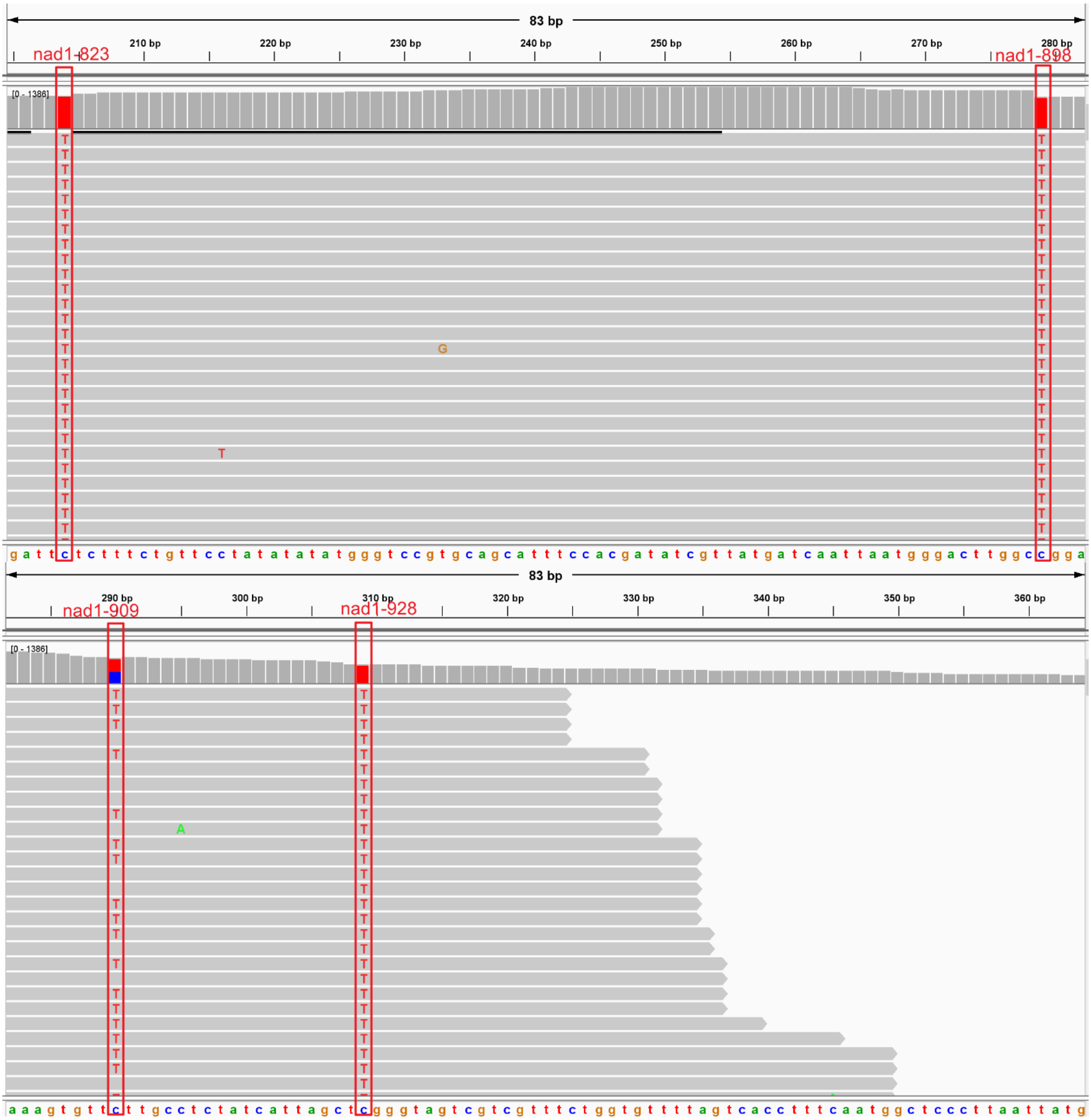
83 bp
210 bp
220 bp
230 bp
240 bp
250 bp
260 bp
270 bp
280 bp
nad1-823
nad1-898
[0 - 1386]
83 bp
290 bp
300 bp
310 bp
320 bp
330 bp
340 bp
350 bp
360 bp
nad1-909
nad1-928
[0 - 1386]

p alignment of RNA-seq reads to the coding sequence of *nad2.* 25 RNA-seq editing sites: *nad2*-26, 223, 252, 303, 308, 311, 356, 361, 367, 401, 428, 497, 788, 800, 809, 928, 958, 1028, 1058, 1298, 1400, 1408, 1409, 1416, 1457 were highlighted in red squares.

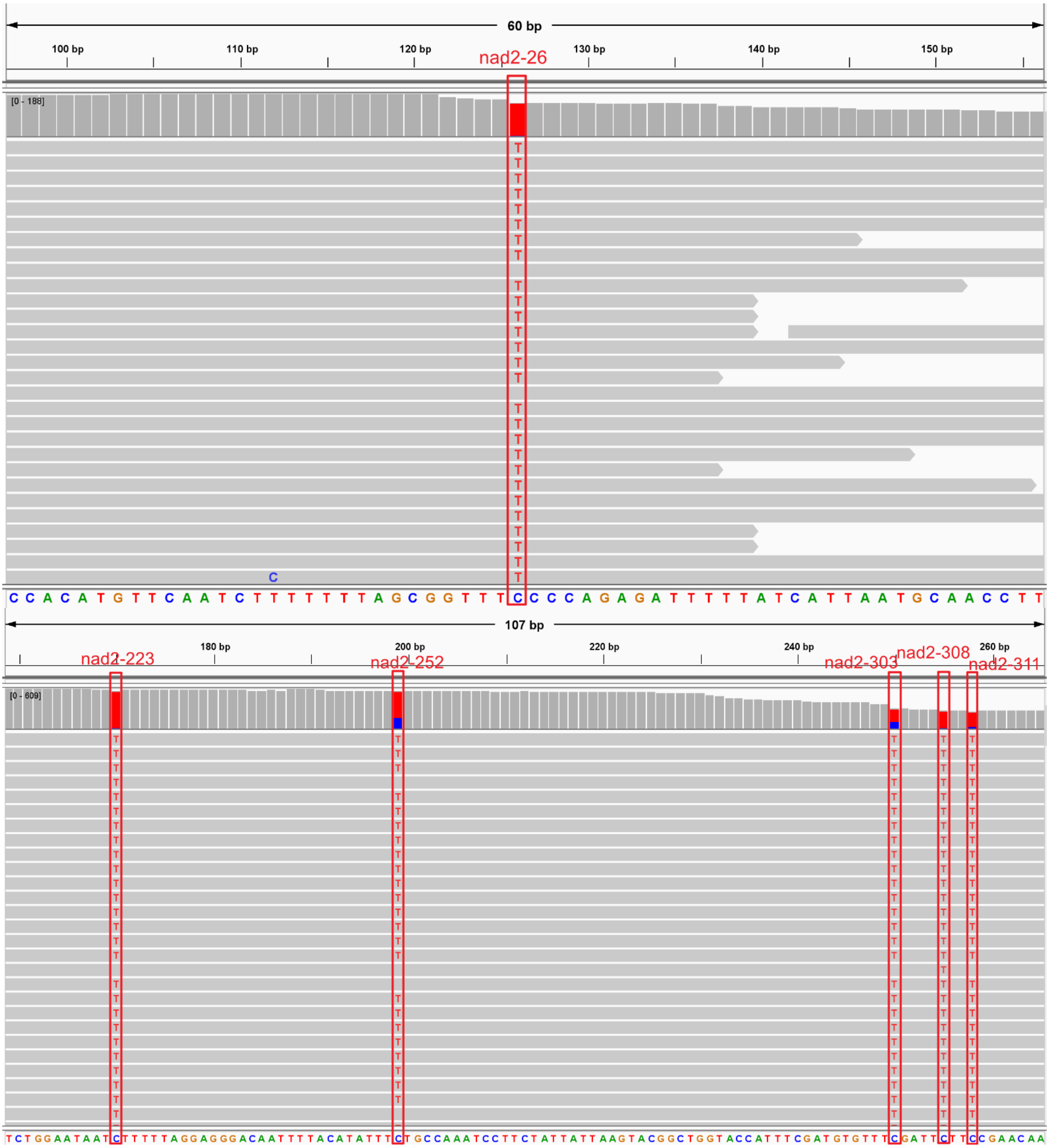

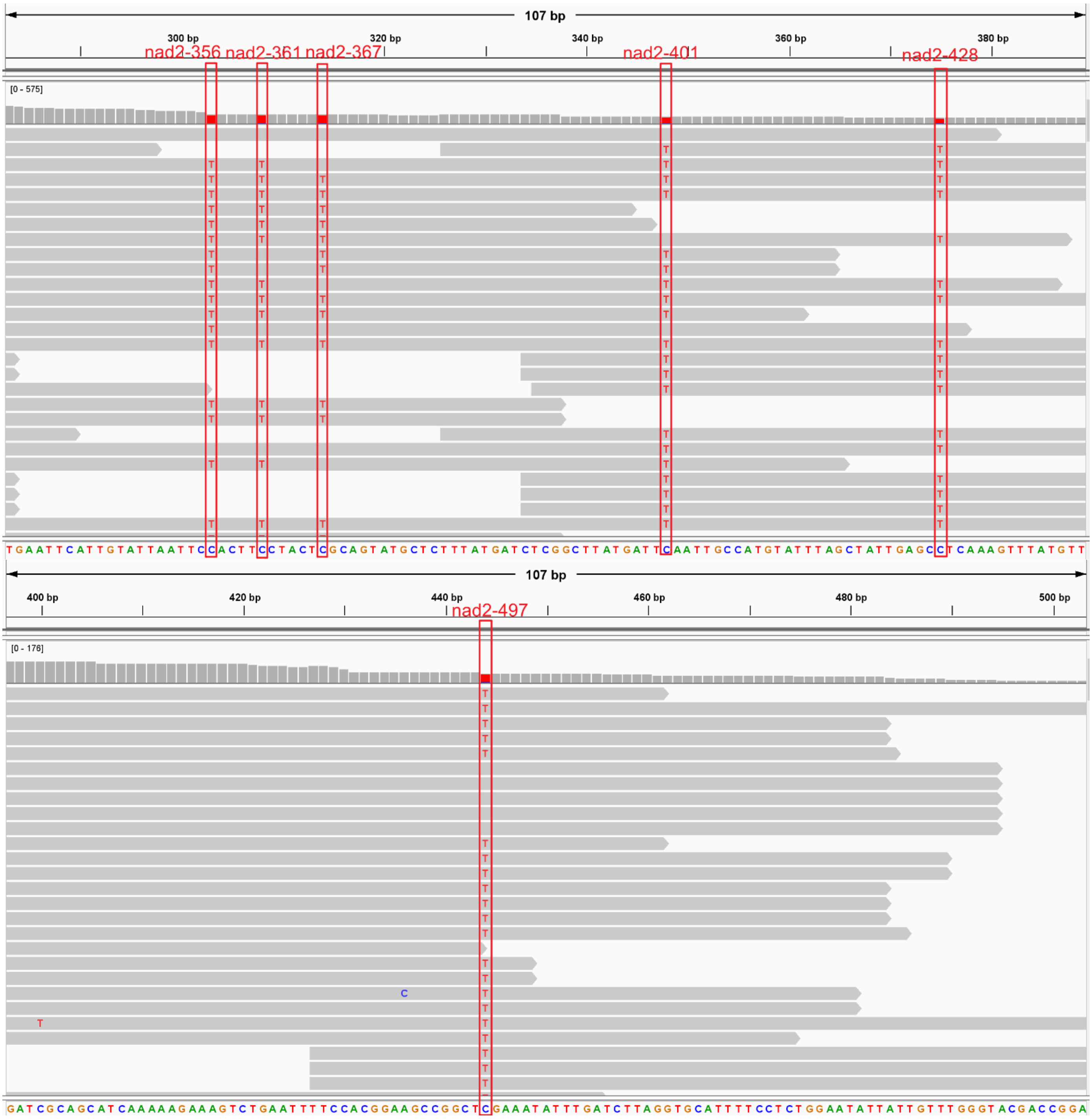

107 bp
300 bp
320 bp
340 bp
360 bp
380 bp
nad2-356
nad2-361
nad2-367
nad2-401
nad2-428
[0 - 575]
107 bp
400 bp
420 bp
440 bp
460 bp
480 bp
500 bp
nad2-497
[0 - 176]

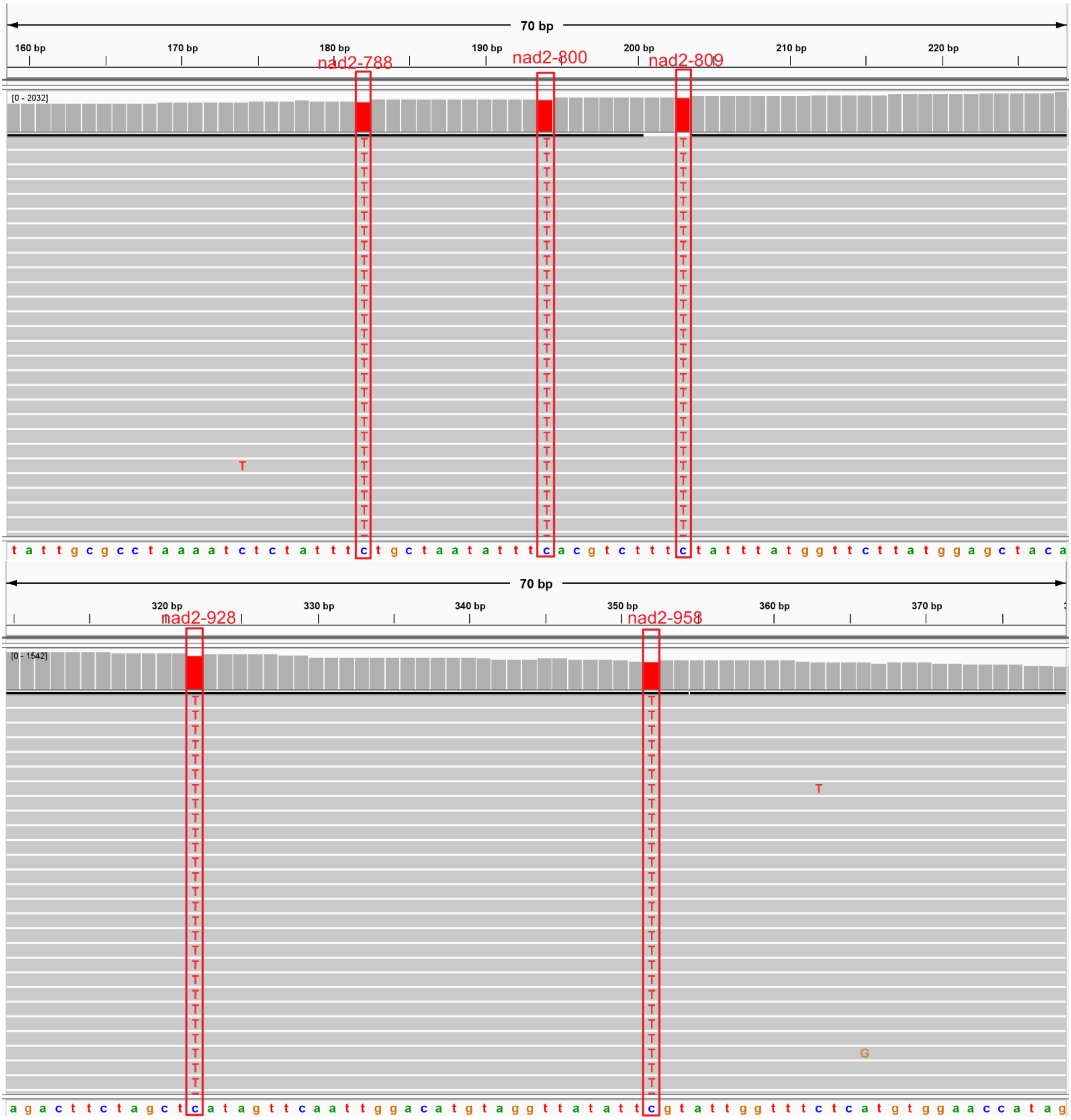
70 bp
160 bp
170 bp
180 bp
190 bp
200 bp
210 bp
220 bp
nad2-788
nad2-800
nad2-809
[0 - 2032]
70 bp
320 bp
330 bp
340 bp
350 bp
360 bp
370 bp
nad2-928
nad2-958
[0 - 1542]

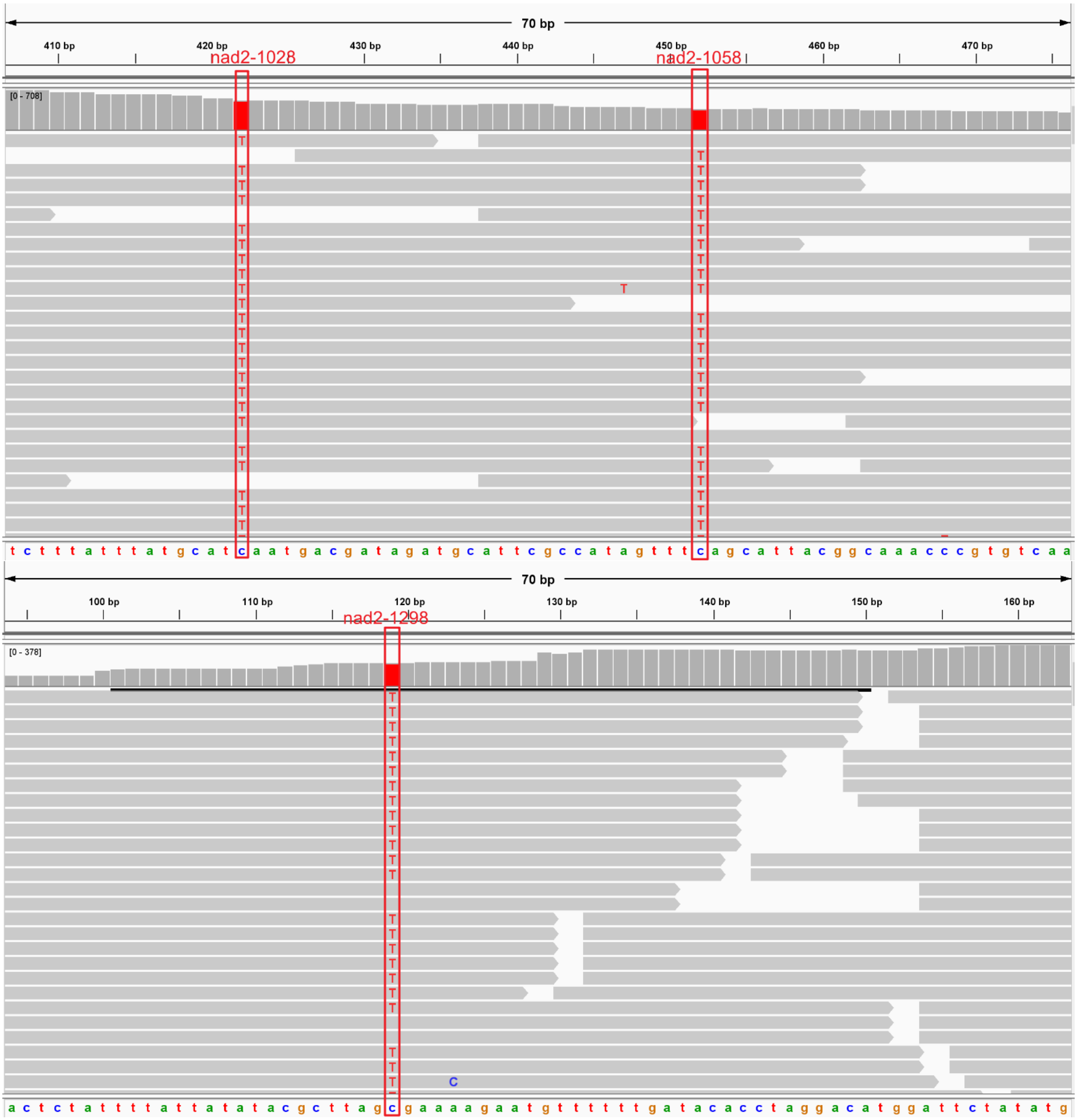

70 bp
410 bp
420 bp
430 bp
440 bp
450 bp
460 bp
470 bp
nad2-1028
nad2-1058
[0 - 708]
70 bp
100 bp
110 bp
120 bp
130 bp
140 bp
150 bp
160 bp
nad2-1298
[0 - 378]

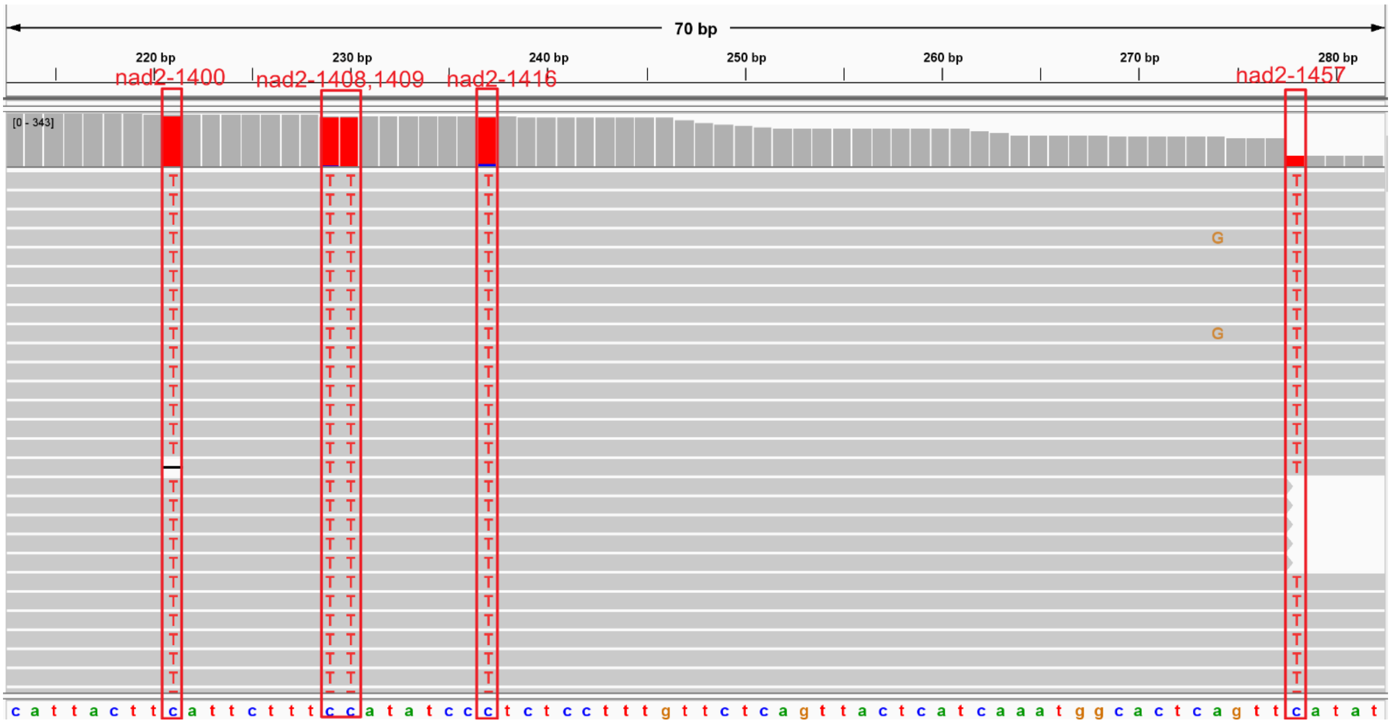

70 bp
220 bp
230 bp
240 bp
250 bp
260 bp
270 bp
280 bp
nad2-1400
nad2-1408,1409
nad2-1416
nad2-1457
[0 - 343]

q alignment of RNA-seq reads to the coding sequence of *nad3.* 17 RNA-seq editing sites: *nad3*-44, 62, 79, 80, 124, 146, 208, 209, 215, 230, 247, 251, 266, 275, 317, 344, 349 were highlighted in red squares.

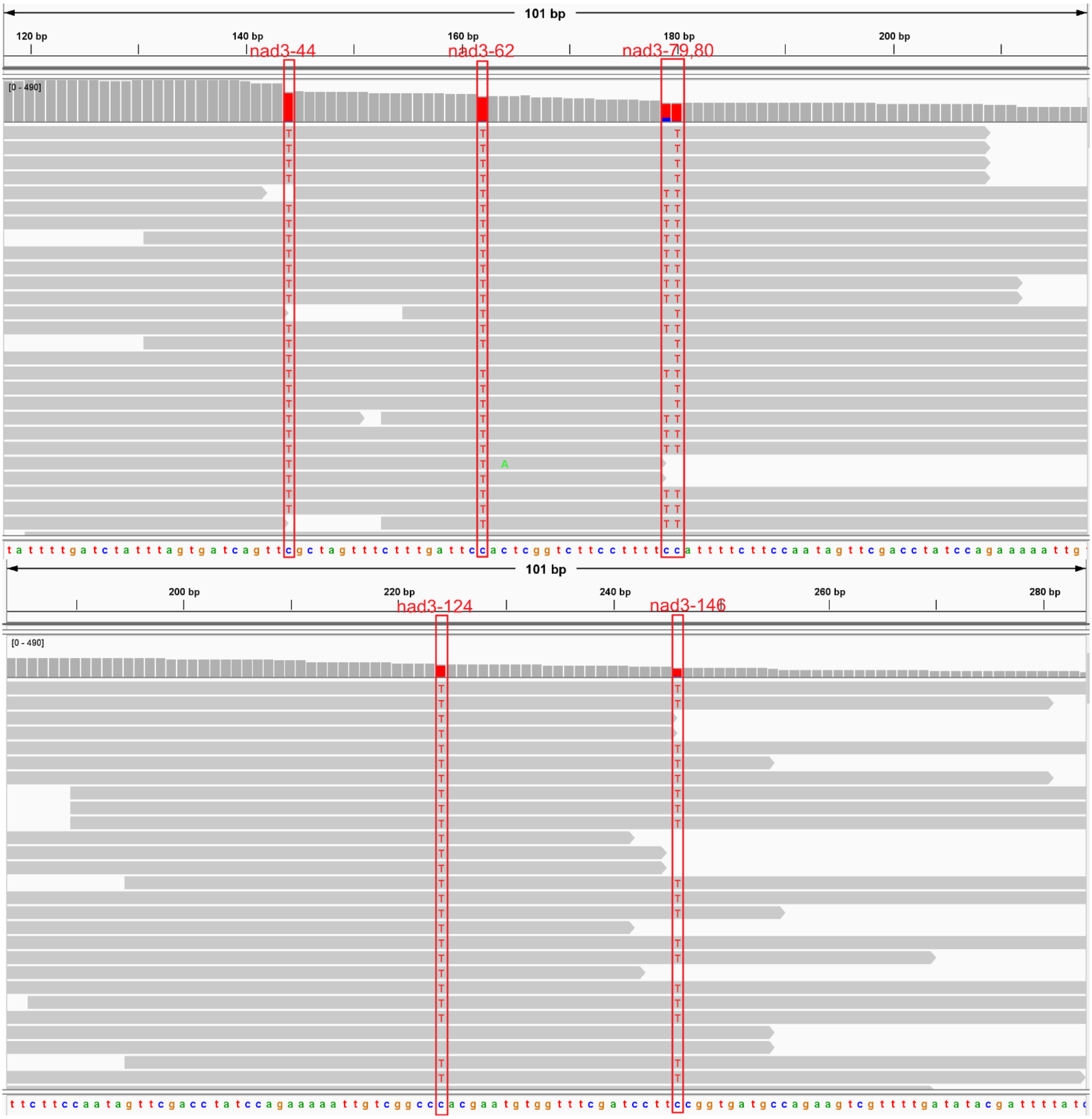

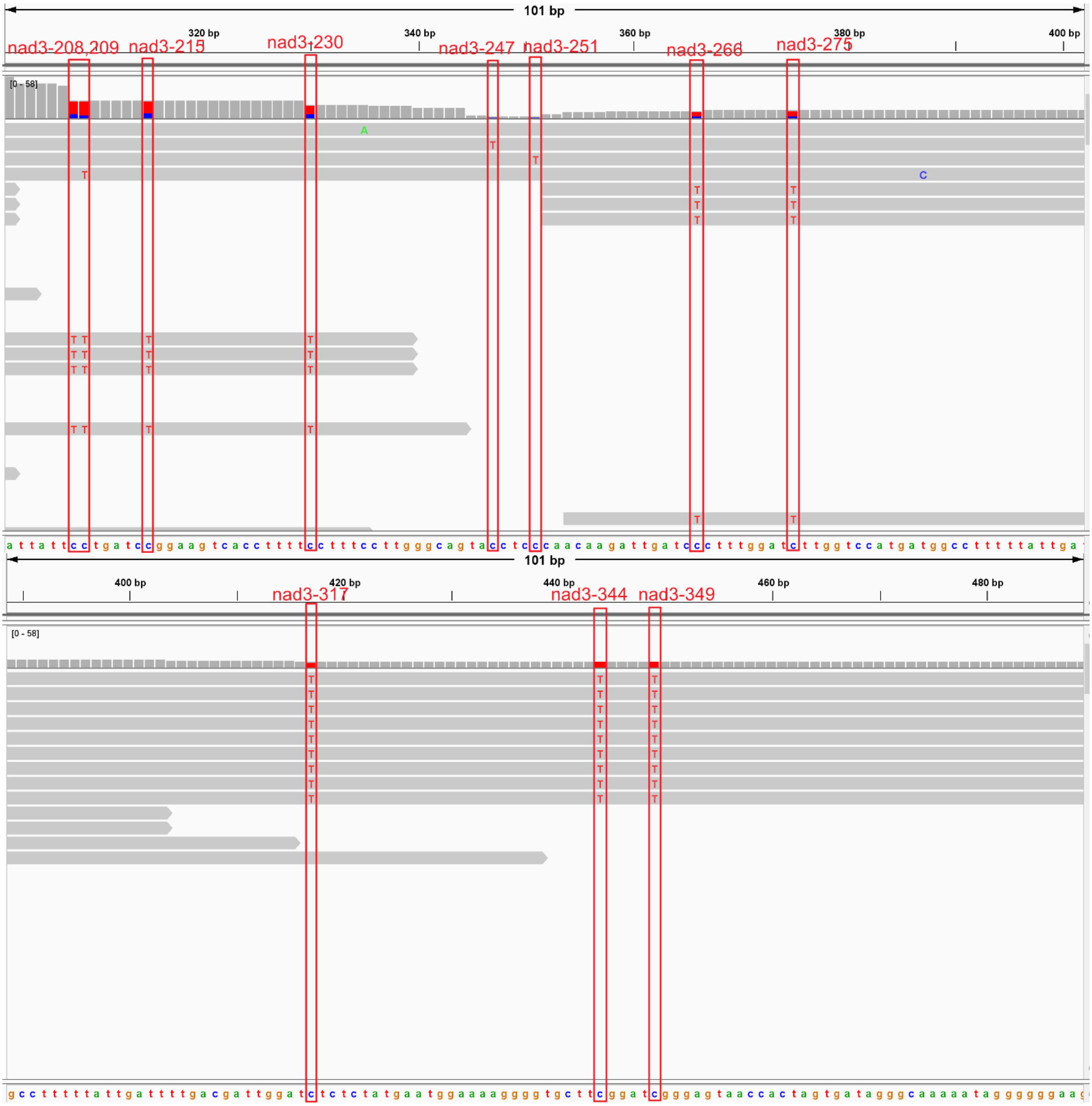

101 bp
320 bp
340 bp
360 bp
380 bp
400 bp
[0 - 58]
nad3-208,209
nad3-215
nad3-230
nad3-247
nad3-251
nad3-266
nad3-275
101 bp
400 bp
420 bp
440 bp
460 bp
480 bp
[0 - 58]
nad3-317
nad3-344
nad3-349

r alignment of RNA-seq reads to the coding sequence of *nad4.* 36 RNA-seq editing sites: *nad4*-65, 68, 75, 98, 149, 157, 188, 353, 359, 367, 407, 424, 427, 428, 440, 599, 637, 650, 758, 810, 847, 848, 878, 997, 1001, 1007, 1100, 1120, 1123, 1142, 1163, 1298, 1346, 1364, 1408, 1424 were highlighted in red squares.

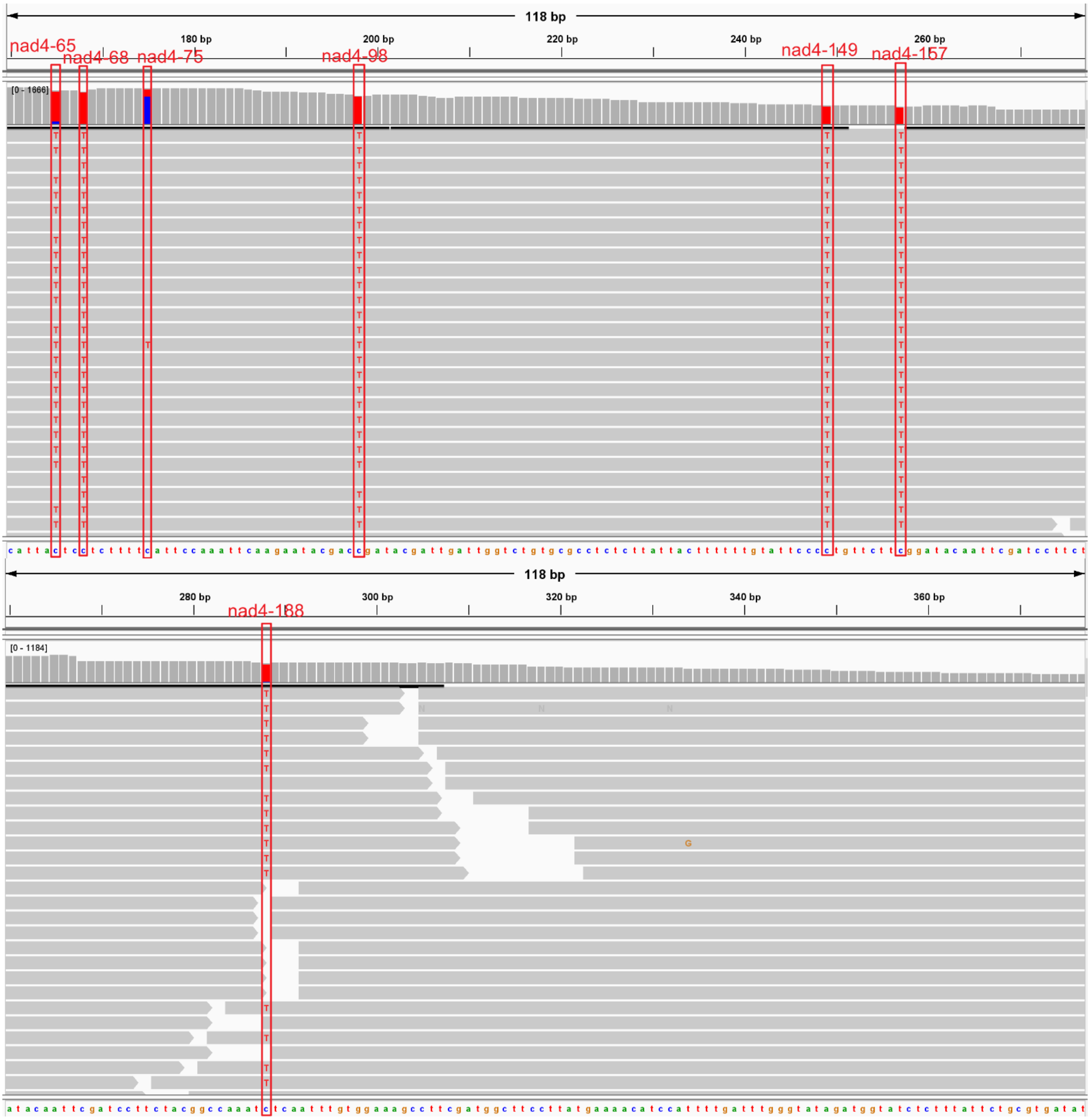

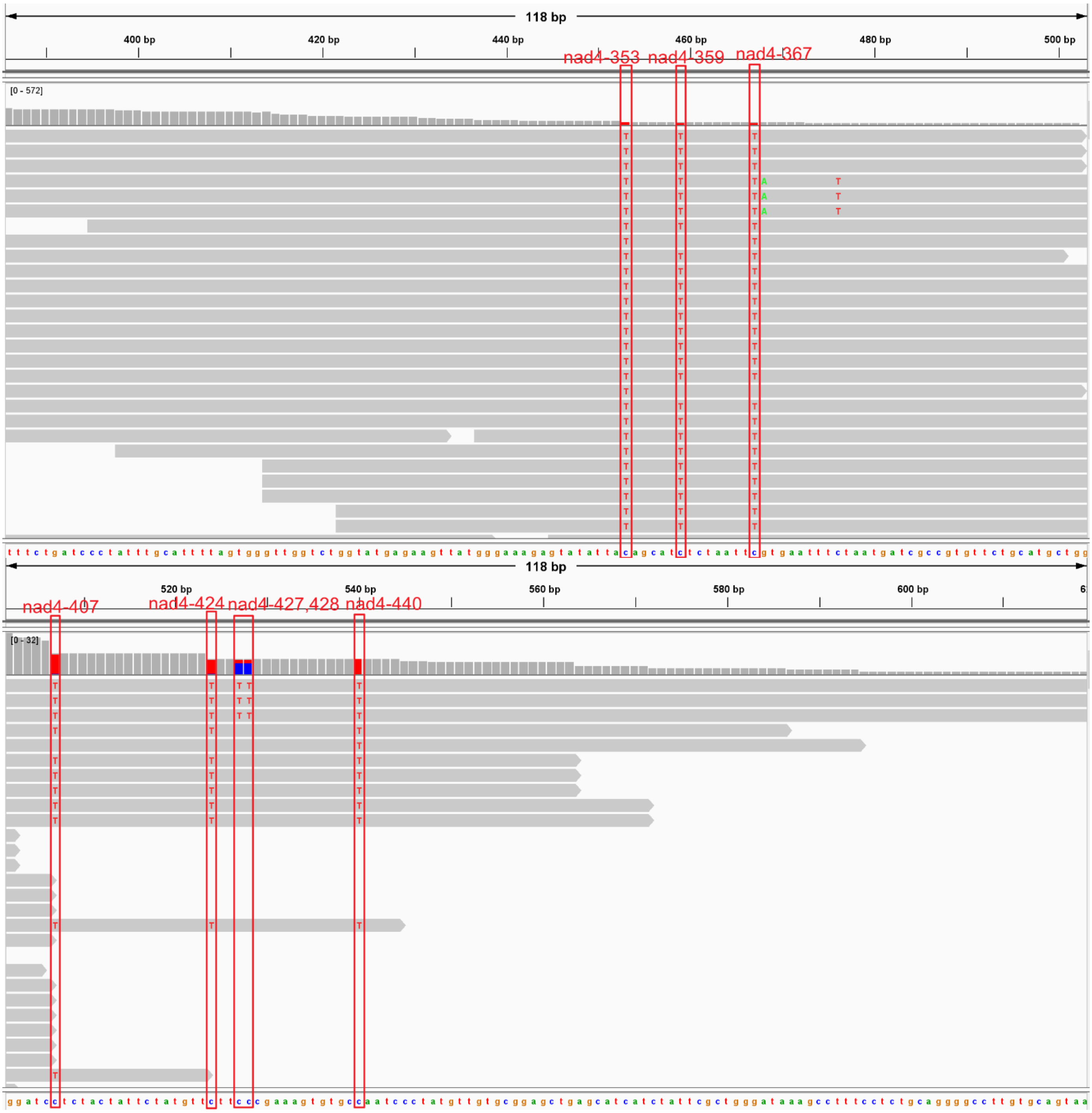

118 bp
400 bp
420 bp
440 bp
460 bp
480 bp
500 bp
nad4-353
nad4-359
nad4-367
[0 - 572]
118 bp
520 bp
540 bp
560 bp
580 bp
600 bp
nad4-407
nad4-424
nad4-427,428
nad4-440
[0 - 32]

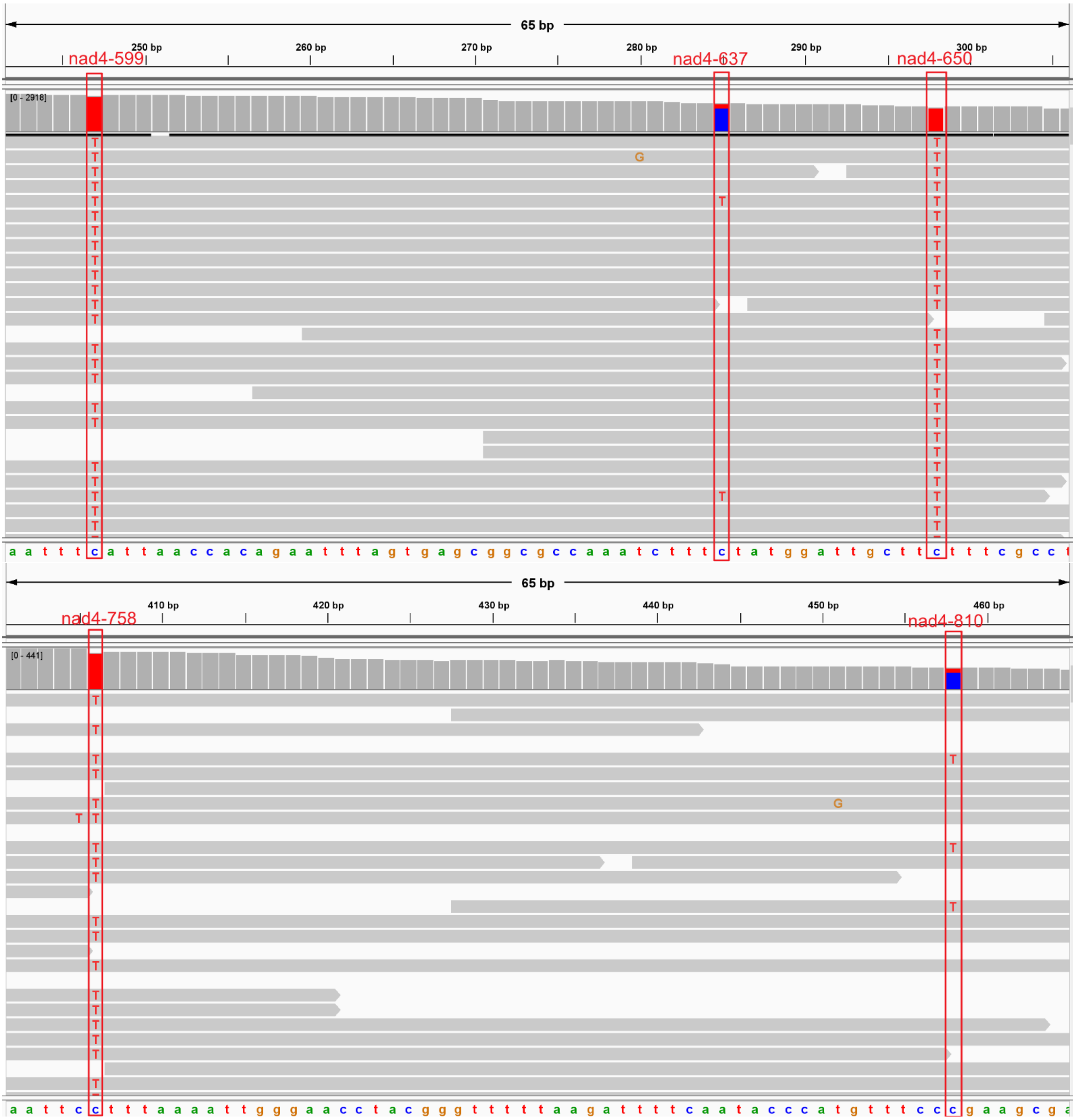
65 bp
250 bp
260 bp
270 bp
280 bp
290 bp
300 bp
nad4-599
nad4-637
nad4-650
[0 - 2918]
65 bp
410 bp
420 bp
430 bp
440 bp
450 bp
460 bp
nad4-758
nad4-810
[0 - 441]

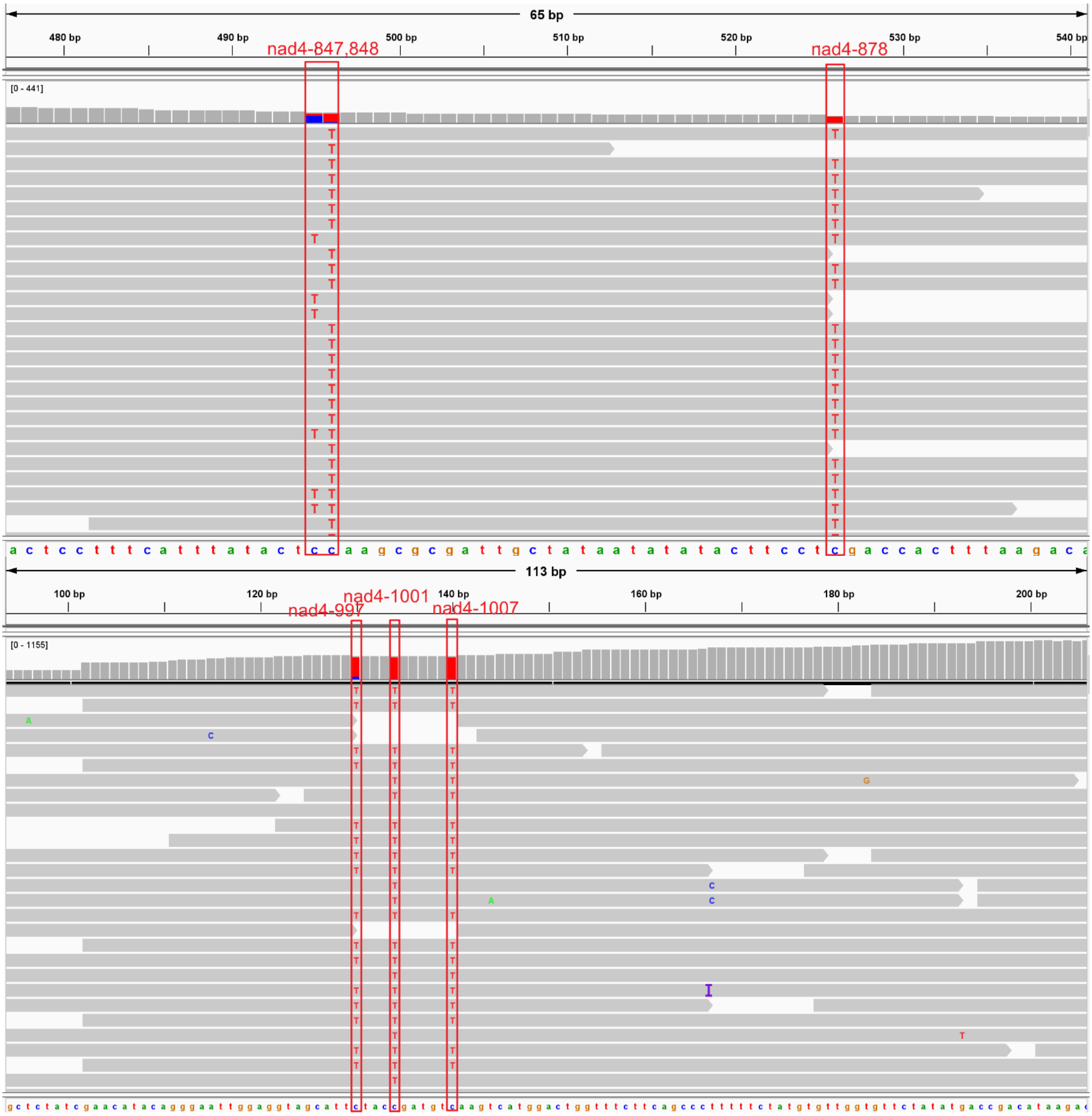
65 bp
480 bp
490 bp
500 bp
510 bp
520 bp
530 bp
540 bp
nad4-847,848
nad4-878
[0 - 441]
113 bp
100 bp
120 bp
140 bp
160 bp
180 bp
200 bp
nad4-997
nad4-1001
nad4-1007
[0 - 1155]

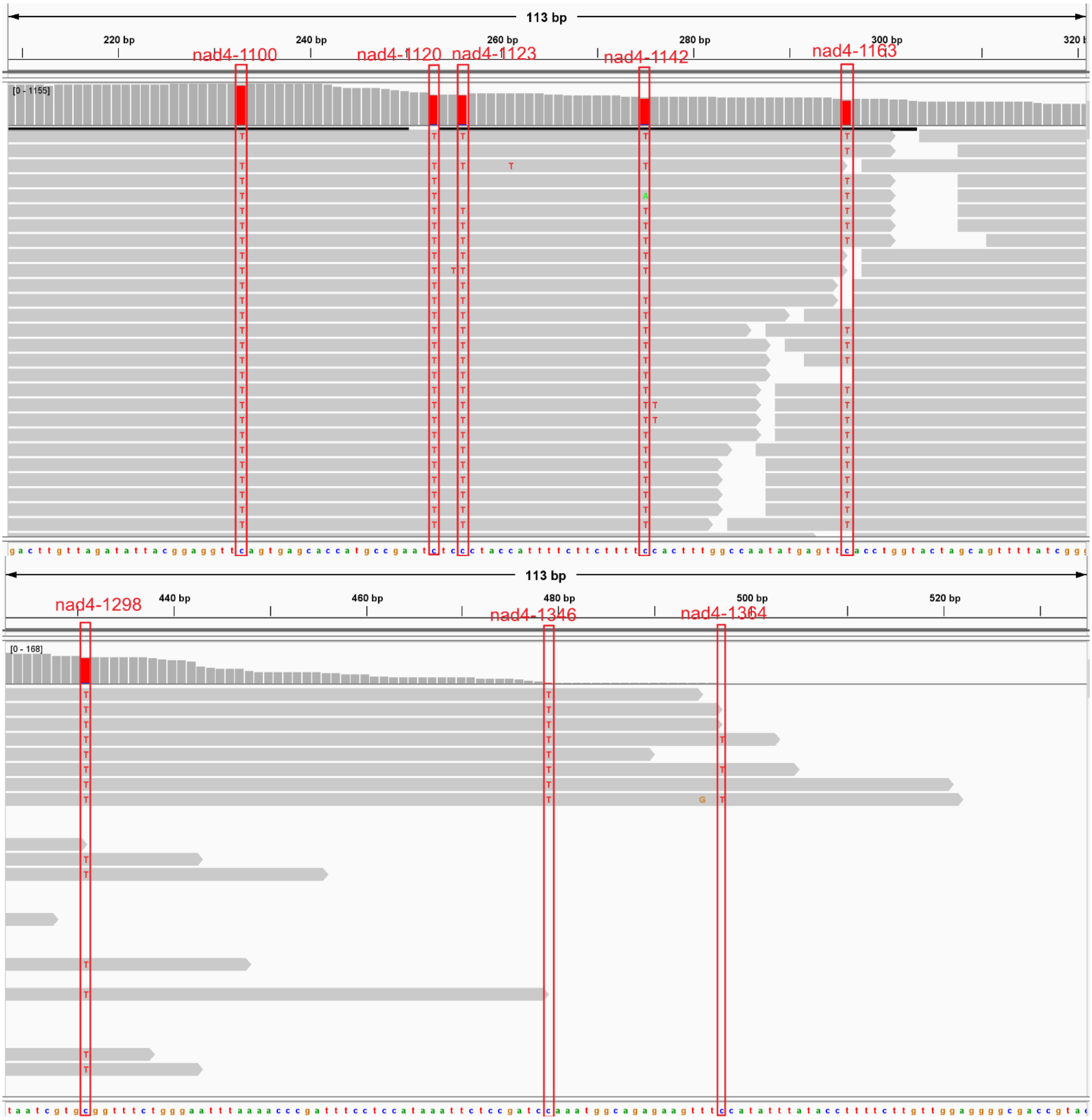
113 bp
220 bp
240 bp
260 bp
280 bp
300 bp
nad4-1100
nad4-1120
nad4-1123
nad4-1142
nad4-1163
[0 - 1155]
113 bp
440 bp
460 bp
480 bp
500 bp
520 bp
nad4-1298
nad4-1346
nad4-1364
[0 - 168]

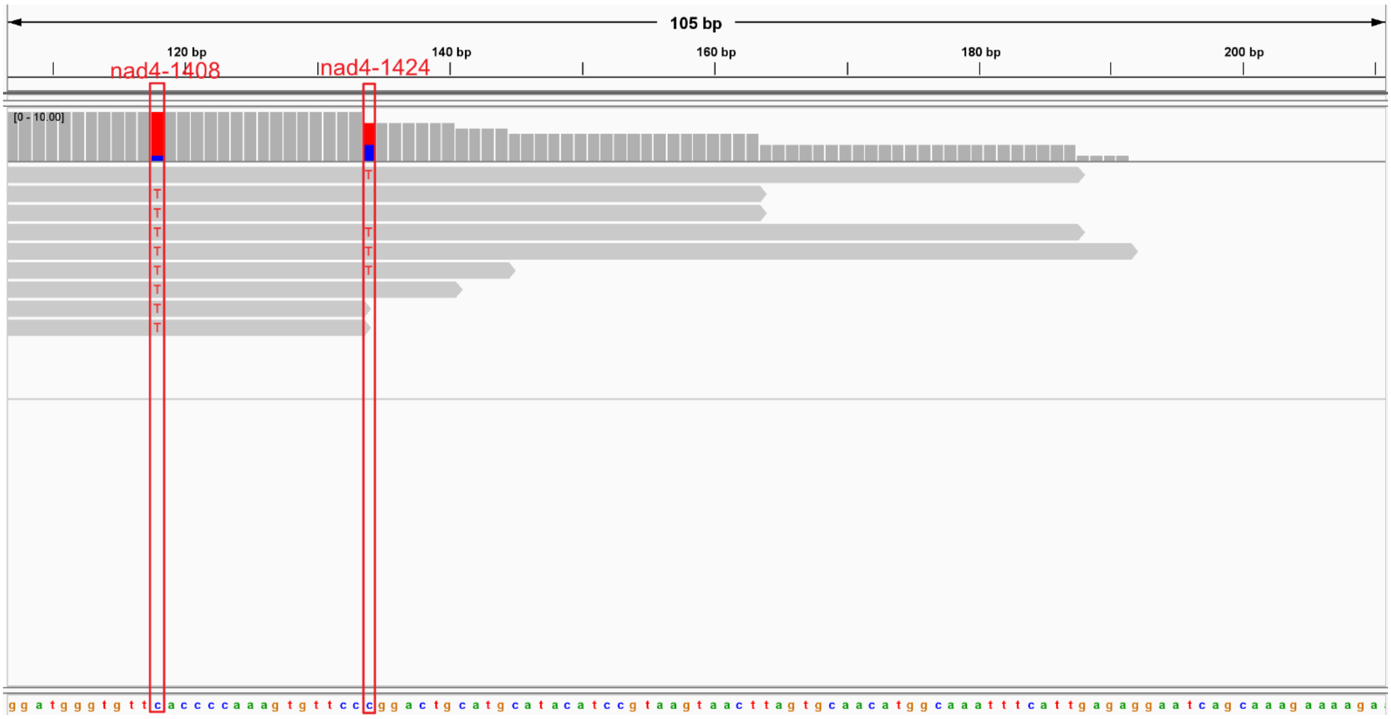
105 bp
120 bp
140 bp
160 bp
180 bp
200 bp
nad4-1408
nad4-1424
[0 - 10.00]

s alignment of RNA-seq reads to the coding sequence of *nad4L*. 14 RNA-seq editing sites: *nad4L*-11, 17, 25, 56, 65, 70, 80, 101, 128, 149, 158, 167, 222, 251 were highlighted in red squares.

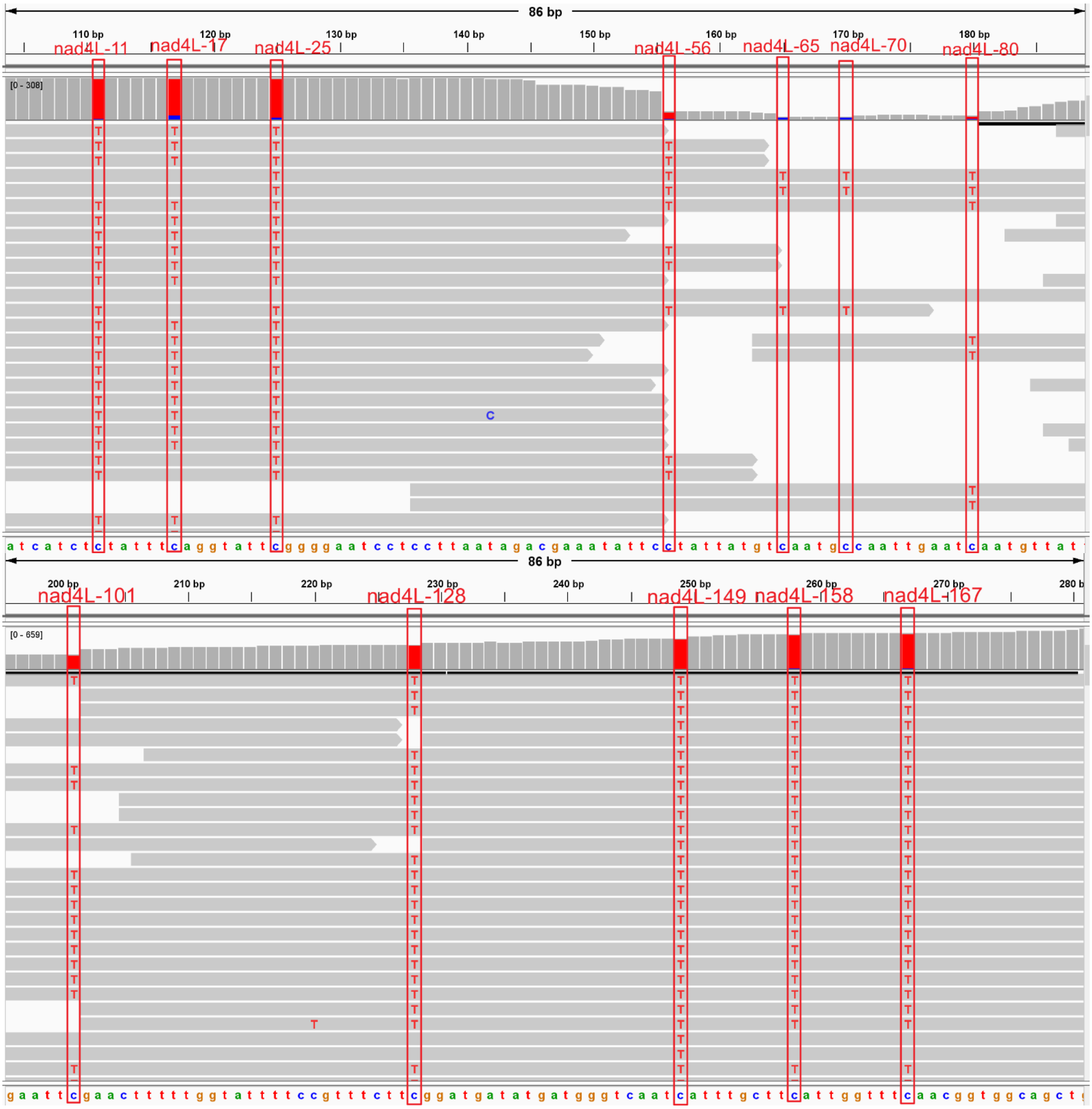

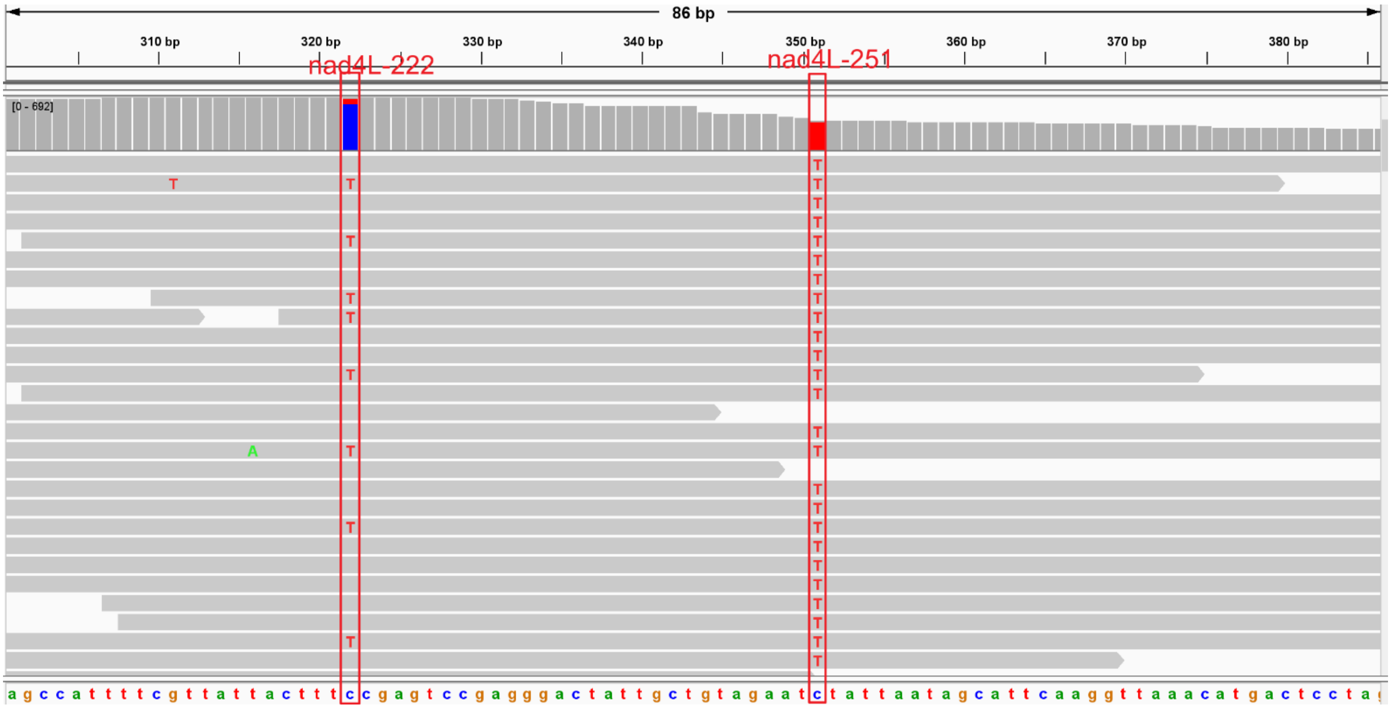
86 bp
310 bp
320 bp
330 bp
340 bp
350 bp
360 bp
370 bp
380 bp
nad4L-222
nad4L-251
[0 - 692]

t alignment of RNA-seq reads to the coding sequence of *nad5.* 27 RNA-seq editing sites: *nad5*-155, 242, 359, 374, 398, 539, 548, 608, 609, 629, 676, 713, 725, 835, 1310, 1490, 1550, 1568, 1580, 1589, 1610, 1695, 1895, 1916, 1918, 1958, 1981 were highlighted in red squares.

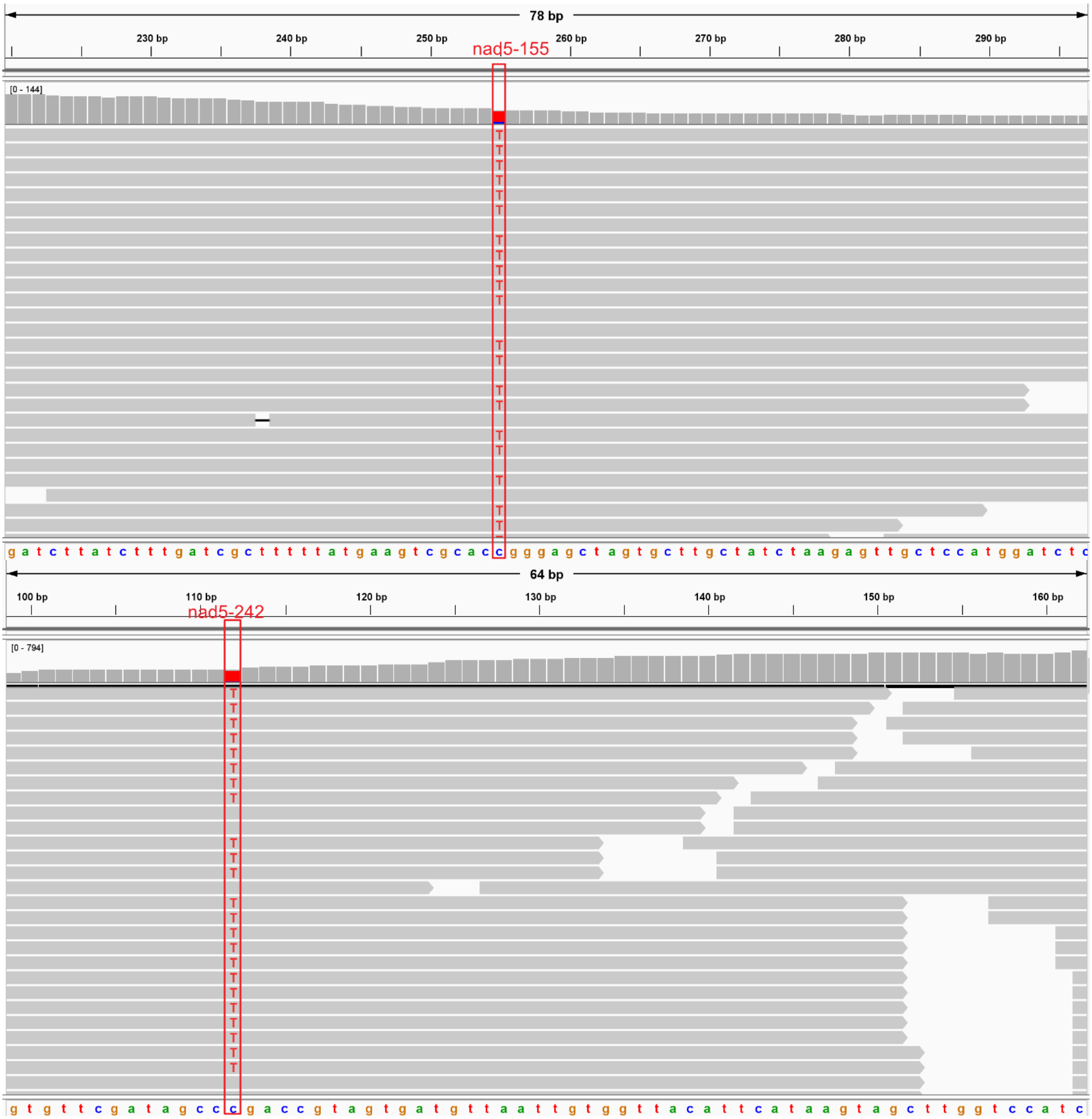

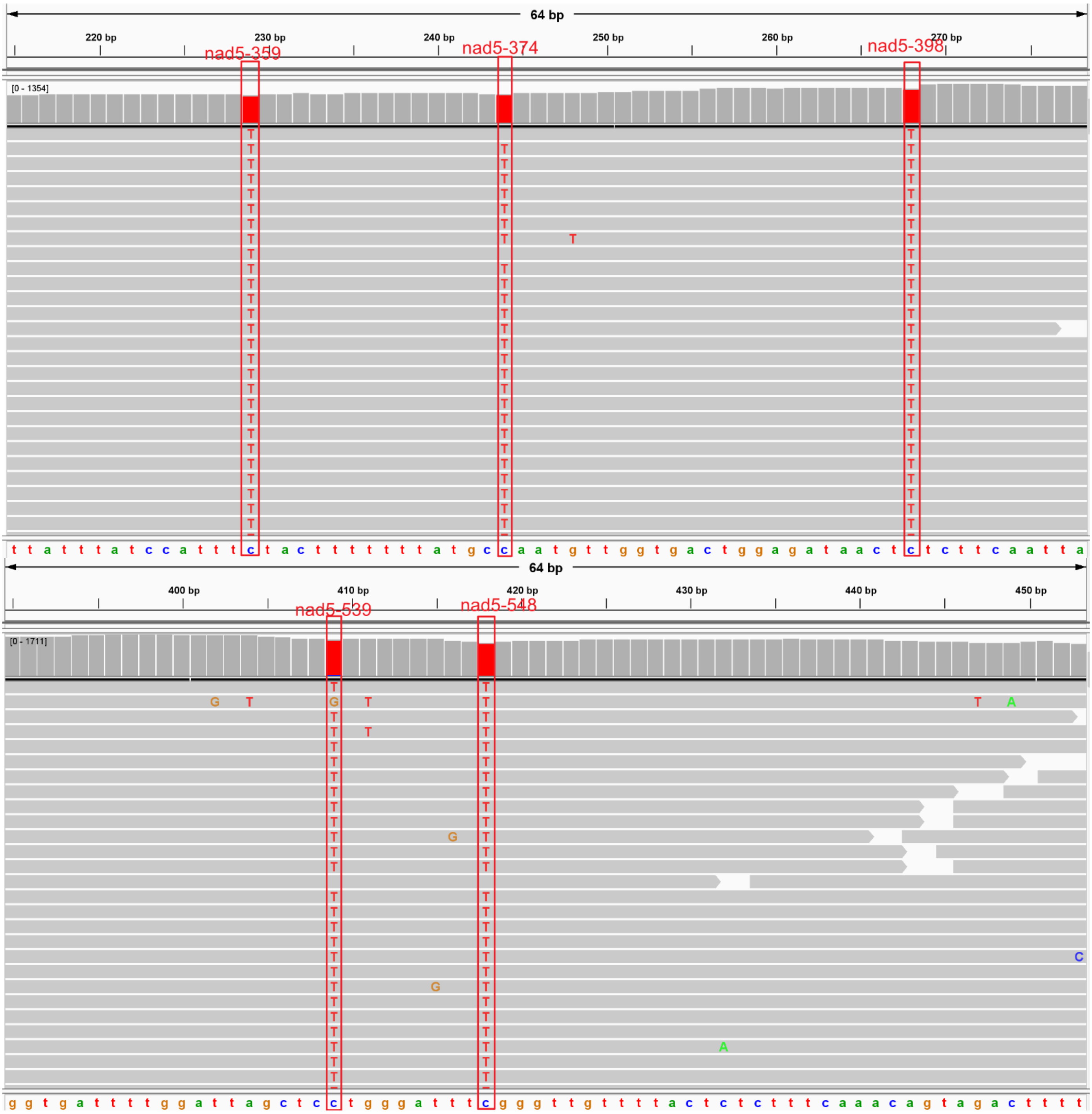
64 bp
220 bp
230 bp
240 bp
250 bp
260 bp
270 bp
nad5-359
nad5-374
nad5-398
[0 - 1354]
t t a t t t a t c c a t t t c t a c t t t t t t t t a t g c c a a t g t t g g t g a c t g g a g a t a a c t c t c t t c a a t t a
64 bp
400 bp
410 bp
420 bp
430 bp
440 bp
450 bp
nad5-539
nad5-548
[0 - 1711]
g g t g a t t t t g g a t t a g c t c c t g g g a t t t c g g g t t g t t t t a c t c t c t t t c a a a c a g t a g a c t t t t

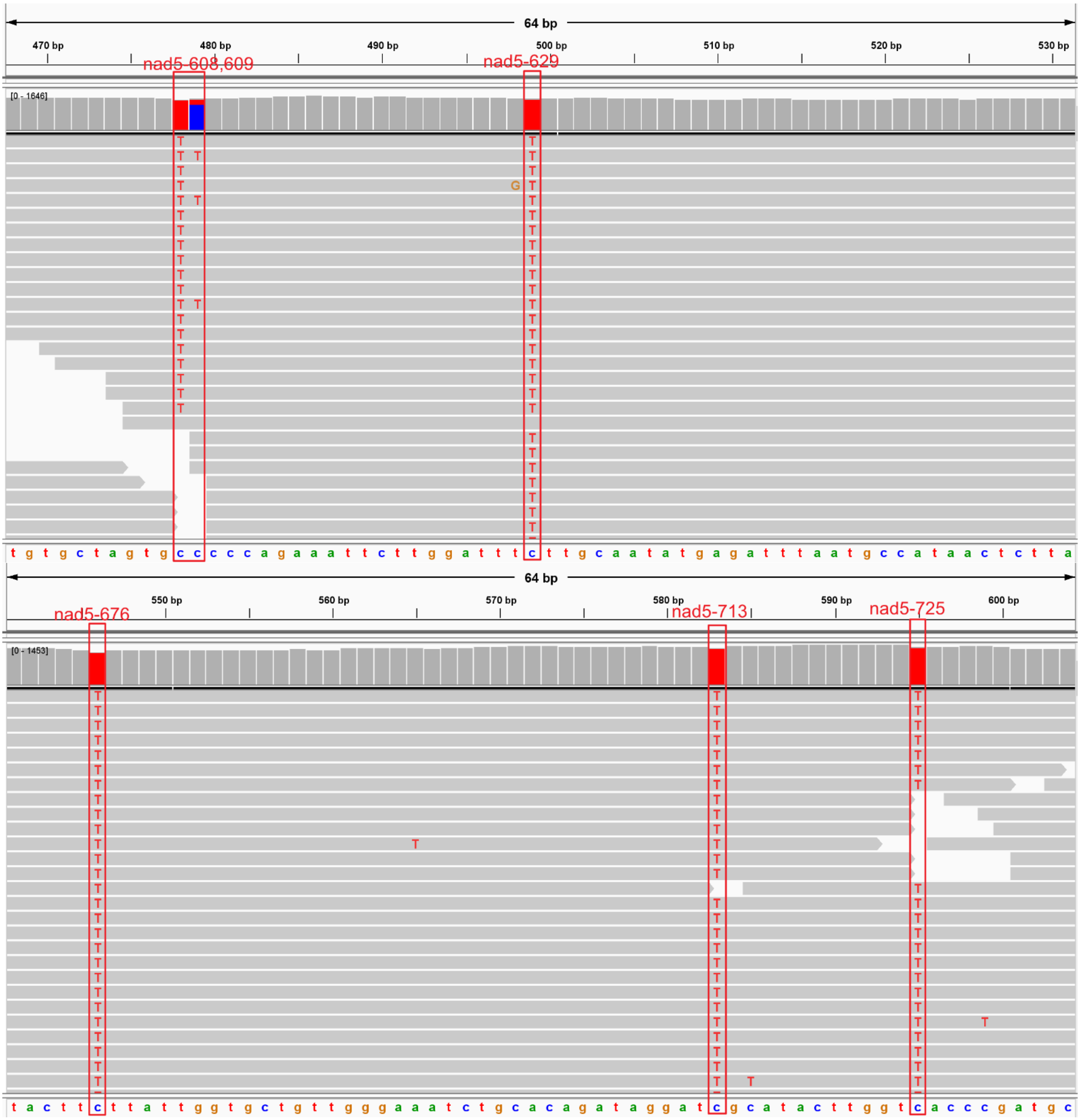
64 bp
470 bp
480 bp
490 bp
500 bp
510 bp
520 bp
530 bp
nad5-608,609
nad5-629
[0 - 1646]
64 bp
550 bp
560 bp
570 bp
580 bp
590 bp
600 bp
nad5-676
nad5-713
nad5-725
[0 - 1453]

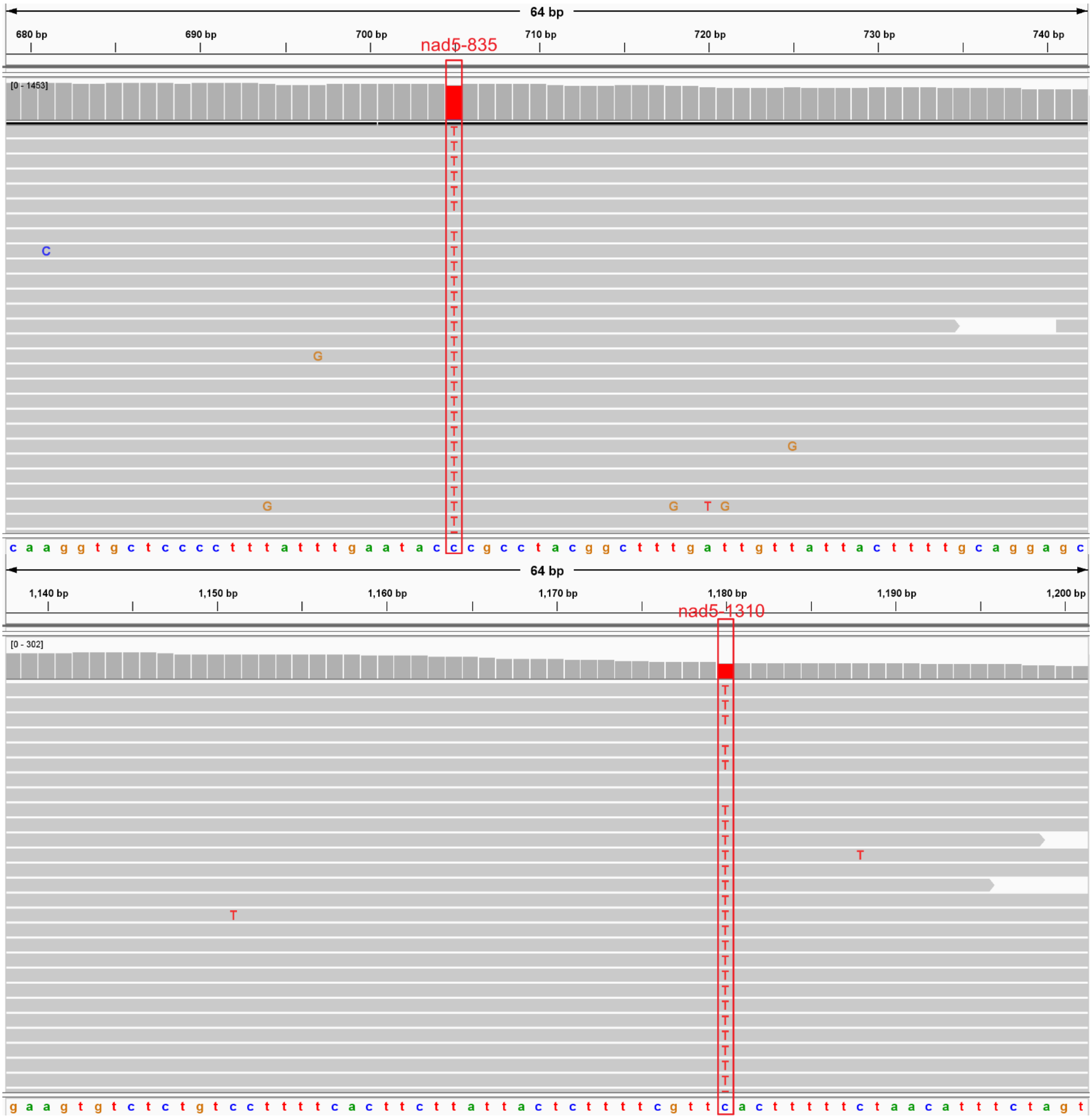
64 bp
680 bp
690 bp
700 bp
710 bp
720 bp
730 bp
740 bp
nad5-835
[0 - 1453]
c a a g g t g c t c c c c t t t a t t t g a a t a c c c g c c t a c g g c t t t g a t t g t t a t t a c t t t t g c a g g a g c
64 bp
1,140 bp
1,150 bp
1,160 bp
1,170 bp
1,180 bp
1,190 bp
1,200 bp
nad5-1310
[0 - 302]
g a a g t g t c t c t g t c c t t t t c a c t t c t t a t t a c t c t t t t c g t t c a c t t t t t t c t a a c a t t t c t a g t

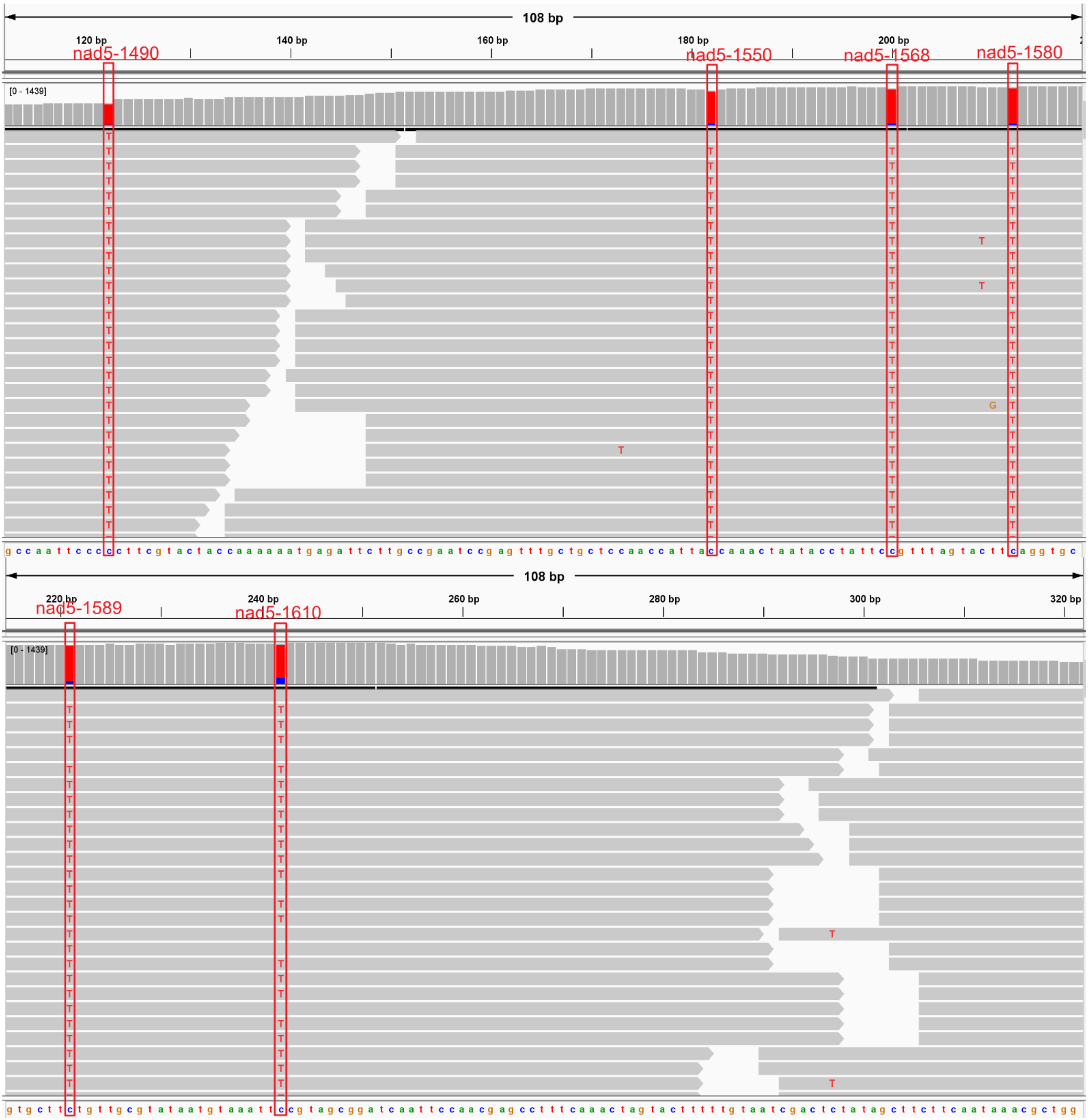
108 bp
120 bp
140 bp
160 bp
180 bp
200 bp
nad5-1490
nad5-1550
nad5-1568
nad5-1580
[0 - 1439]
108 bp
220 bp
240 bp
260 bp
280 bp
300 bp
320 bp
nad5-1589
nad5-1610
[0 - 1439]

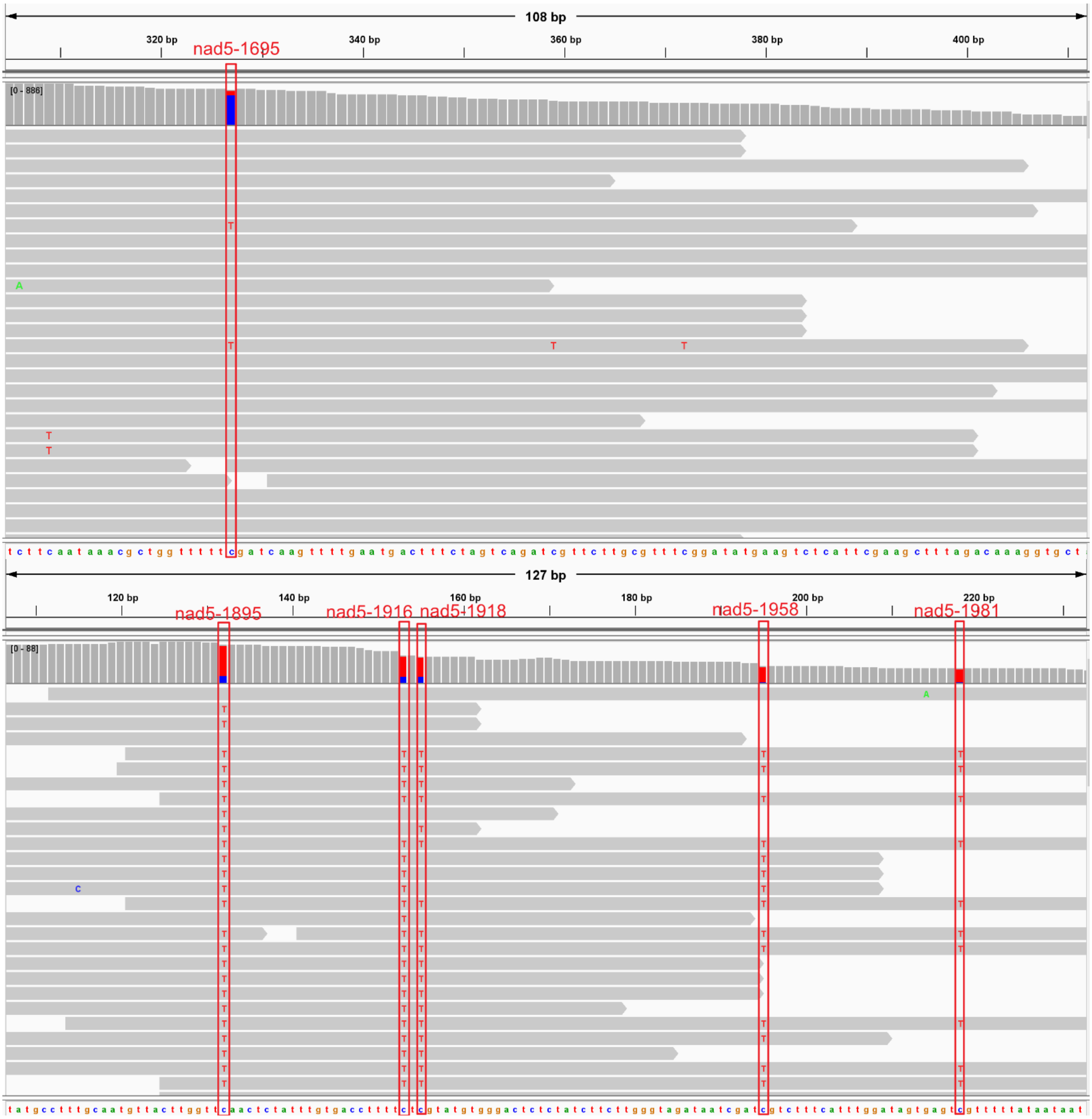
108 bp
320 bp
340 bp
360 bp
380 bp
400 bp
nad5-1695
[0 - 886]
127 bp
120 bp
140 bp
160 bp
180 bp
200 bp
220 bp
nad5-1895
nad5-1916
nad5-1918
nad5-1958
nad5-1981
[0 - 88]

u alignment of RNA-seq reads to the coding sequence of *nad6.* 11 RNA-seq editing sites: *nad6*-26, 88, 89, 95, 103, 161, 169, 191, 306, 463, 569 were highlighted in red squares.

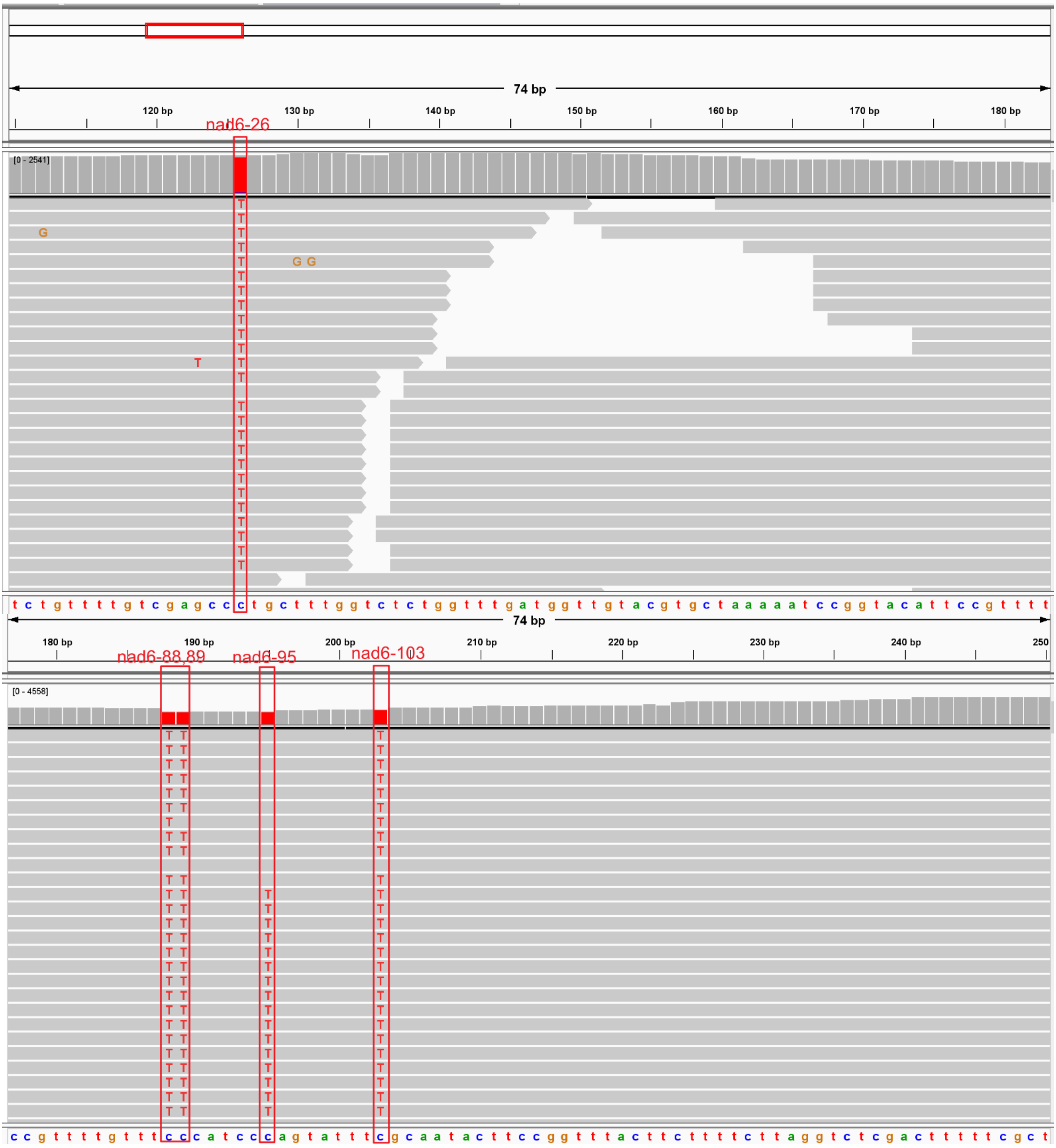

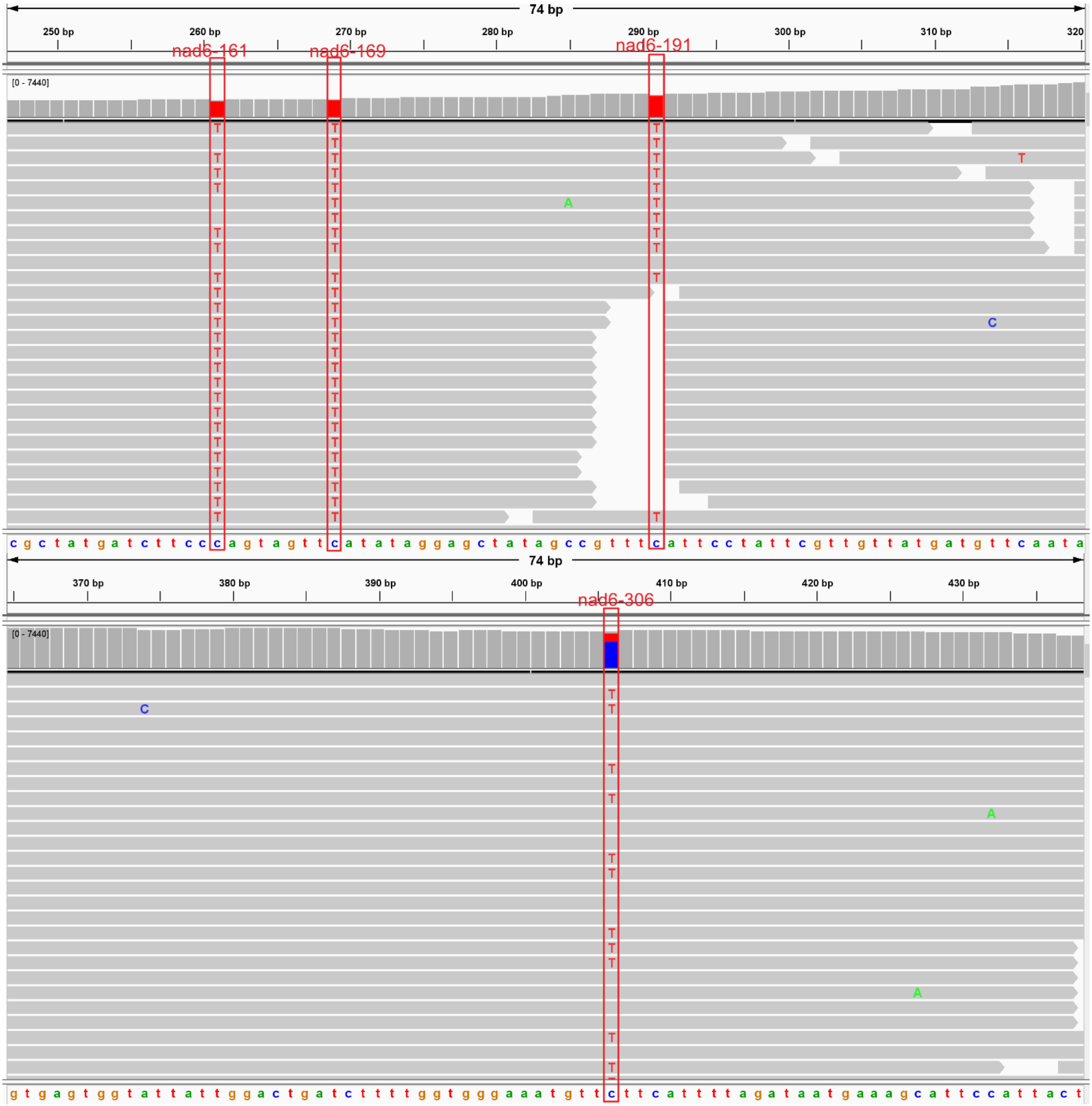
74 bp
250 bp
260 bp
270 bp
280 bp
290 bp
300 bp
310 bp
320
nad6-161
nad6-169
nad6-191
[0 - 7440]
74 bp
370 bp
380 bp
390 bp
400 bp
410 bp
420 bp
430 bp
nad6-306
[0 - 7440]

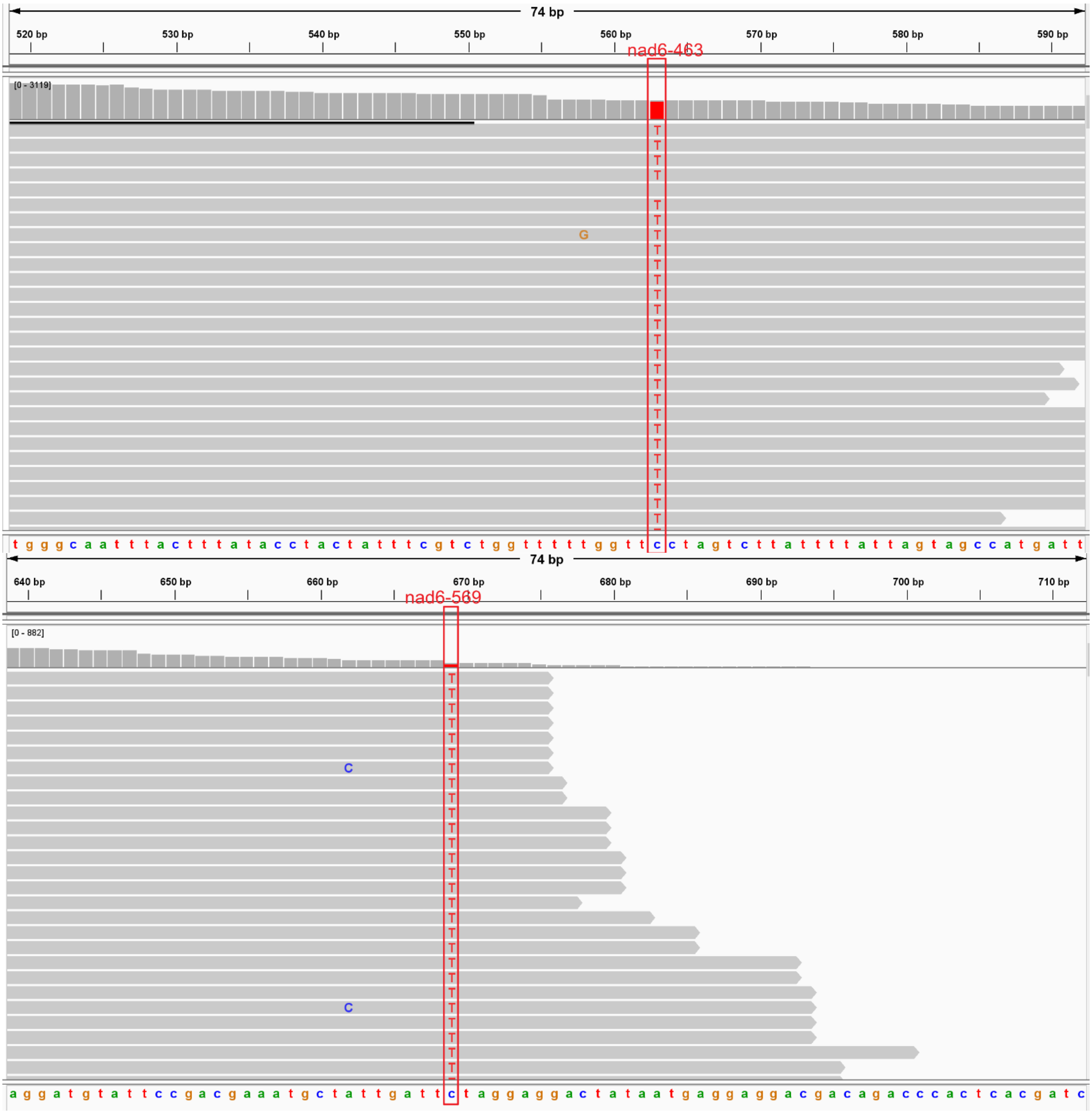

74 bp
520 bp
530 bp
540 bp
550 bp
560 bp
570 bp
580 bp
590 bp
nad6-463
[0 - 3119]
74 bp
640 bp
650 bp
660 bp
670 bp
680 bp
690 bp
700 bp
710 bp
nad6-569
[0 - 882]

v alignment of RNA-seq reads to the coding sequence of *nad7*. 26 RNA-seq editing sites: *nad7*-45, 77, 137, 200, 209, 244, 251, 316, 335, 344, 383, 531, 534, 578, 724, 739, 740, 769, 926, 944, 1050, 1057, 1103, 1124, 1137, 1166 were highlighted in red squares.

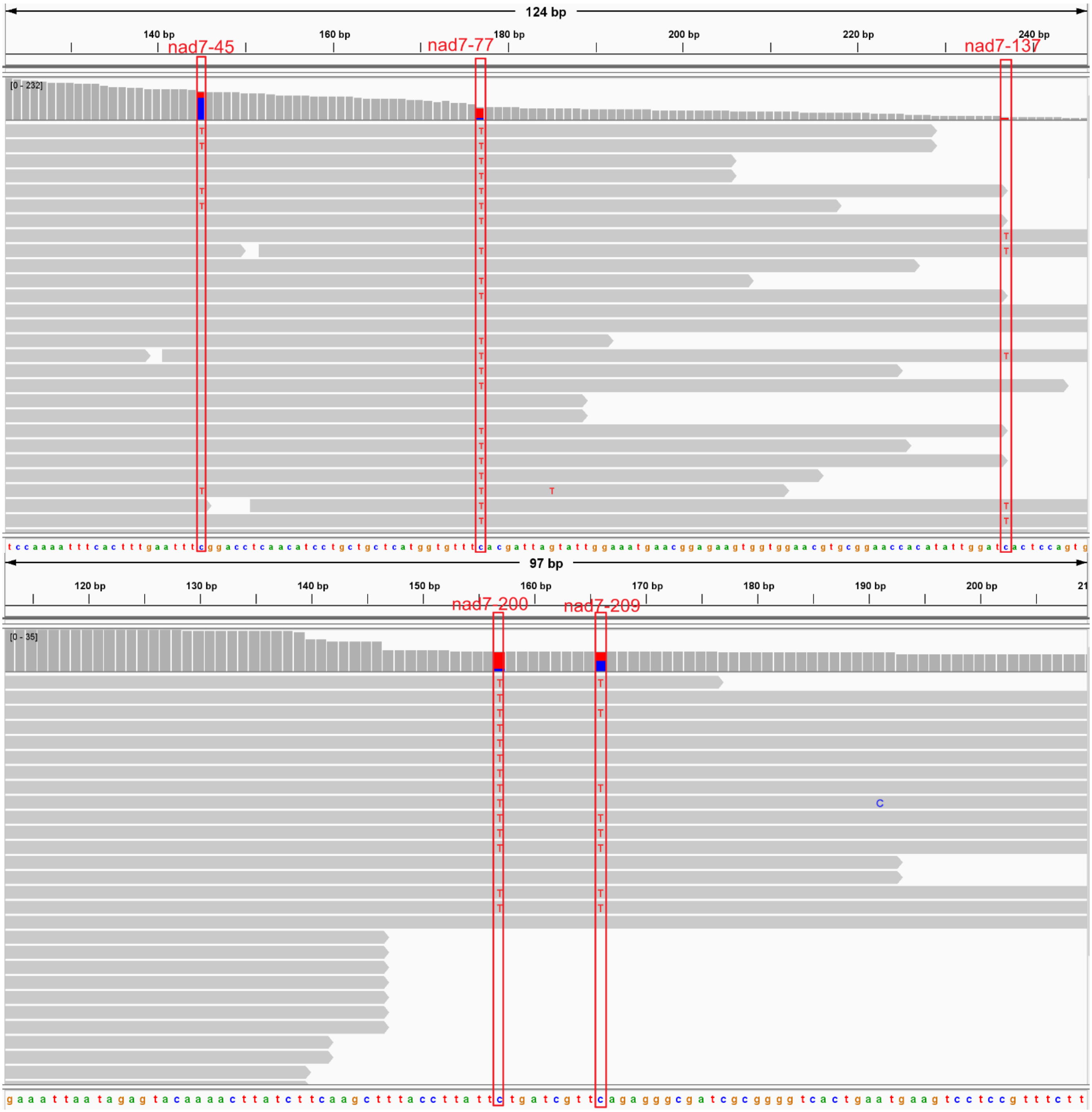

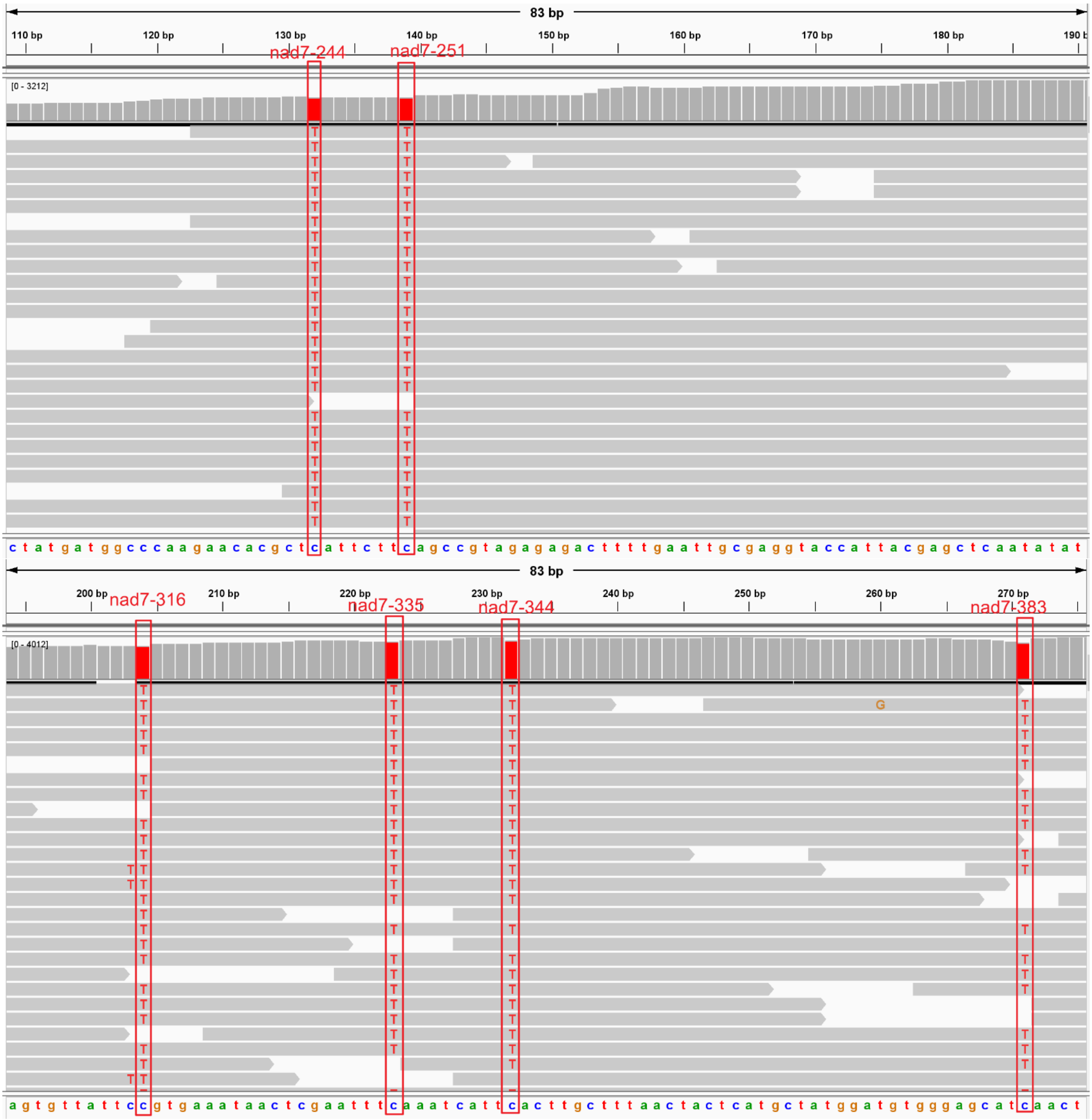
83 bp
110 bp
120 bp
130 bp
140 bp
150 bp
160 bp
170 bp
180 bp
nad7-244
nad7-251
[0 - 3212]
83 bp
200 bp
210 bp
220 bp
230 bp
240 bp
250 bp
260 bp
270 bp
nad7-316
nad7-335
nad7-344
nad7-383
[0 - 4012]

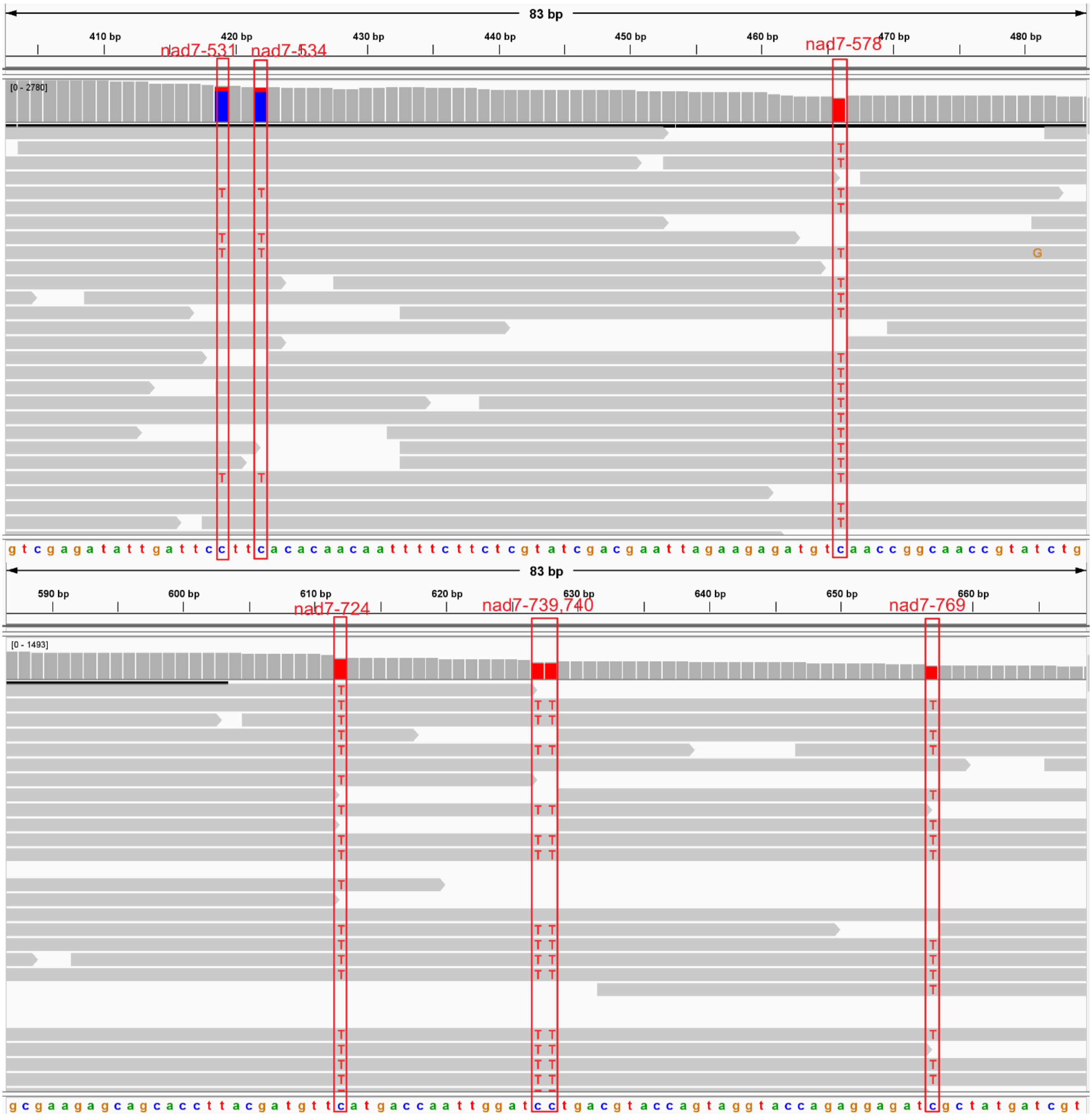
83 bp
410 bp
420 bp
430 bp
440 bp
450 bp
460 bp
470 bp
480 bp
nad7-531
nad7-534
nad7-578
[0 - 2780]
g t c g a g a t a t t g a t t c c t t c a c a c a a c a a t t t t c t t c t c g t a t c g a c g a a t t a g a a g a g a t g t c a a c c g g c a a c c g t a t c t g
83 bp
590 bp
600 bp
610 bp
620 bp
630 bp
640 bp
650 bp
660 bp
nad7-724
nad7-739,740
nad7-769
[0 - 1493]
g c g a a g a g c a g c a c c t t a c g a t g t t c a t g a c c a a t t g g a t c c t g a c g t a c c a g t a g g t a c c a g a g g a g a t c g c t a t g a t c g t

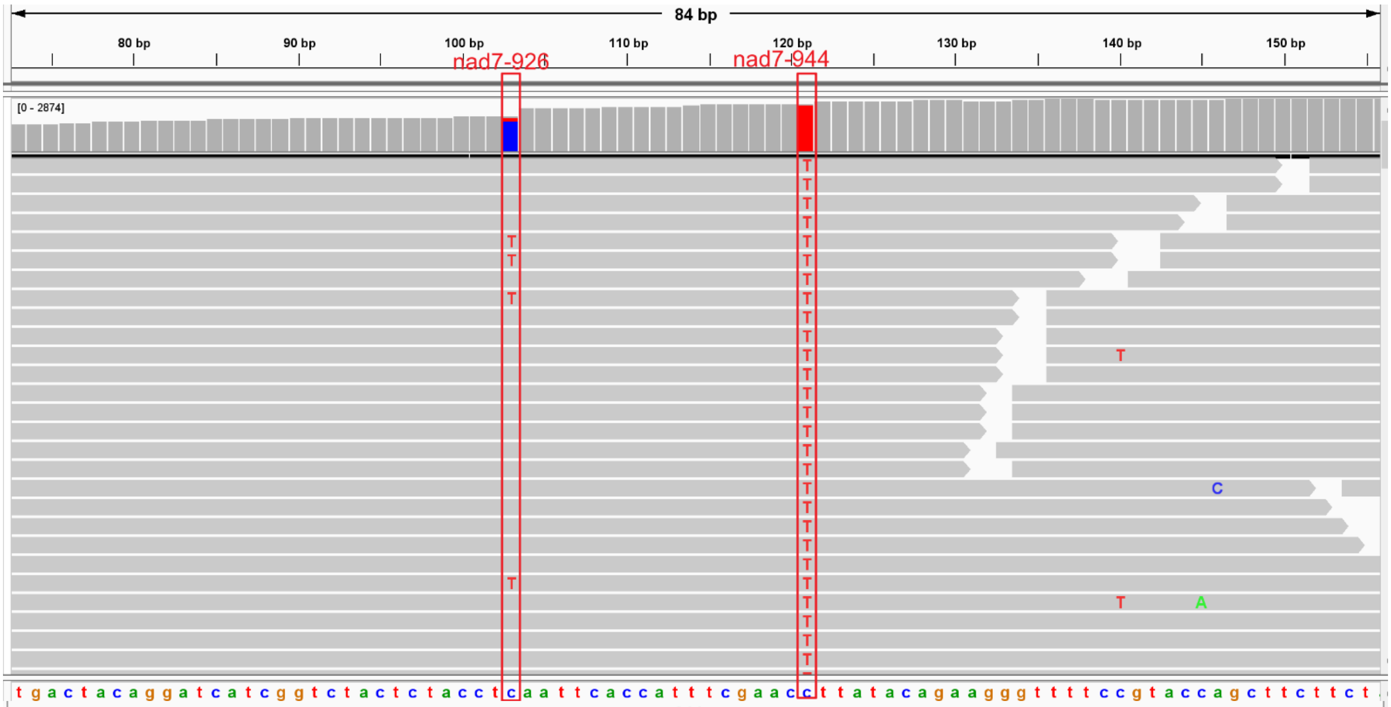
84 bp
80 bp
90 bp
100 bp
110 bp
120 bp
130 bp
140 bp
150 bp
nad7-926
nad7-944
[0 - 2874]

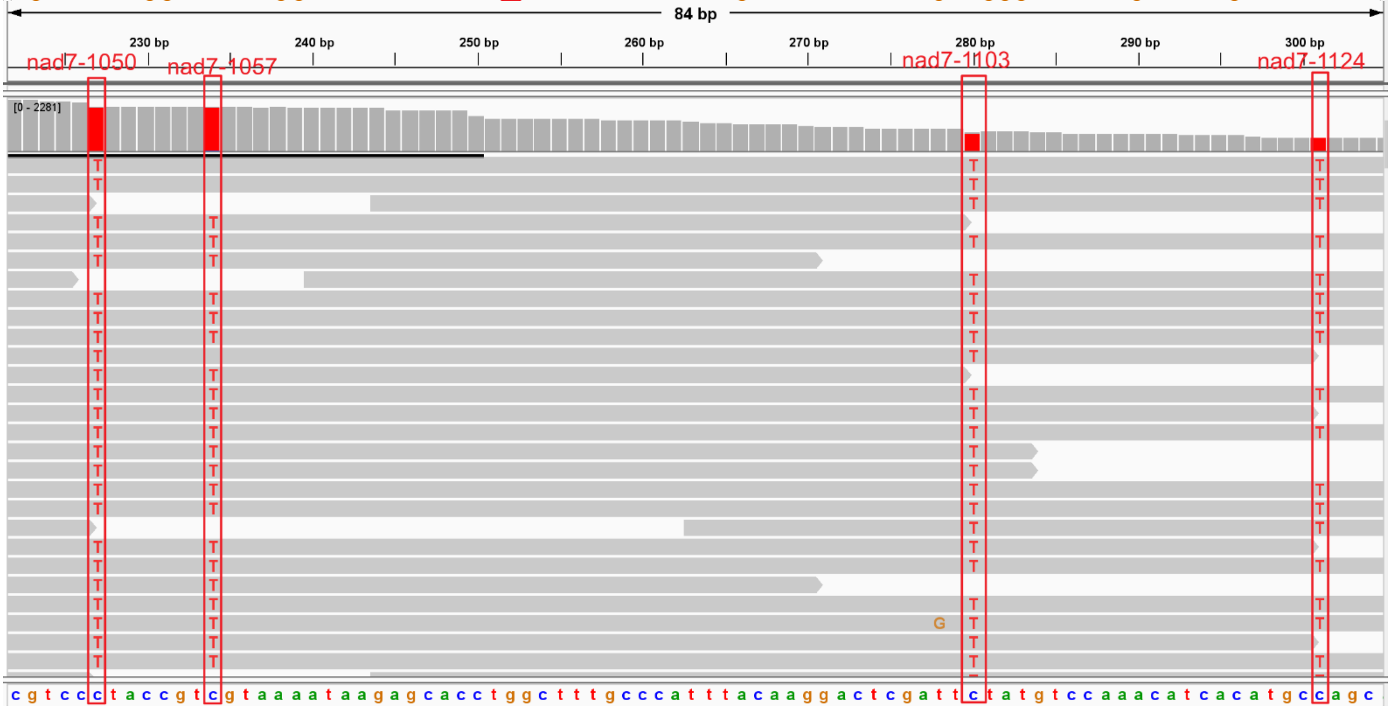
84 bp
230 bp
240 bp
250 bp
260 bp
270 bp
280 bp
290 bp
300 bp
nad7-1050
nad7-1057
nad7-1103
nad7-1124
[0 - 2281]

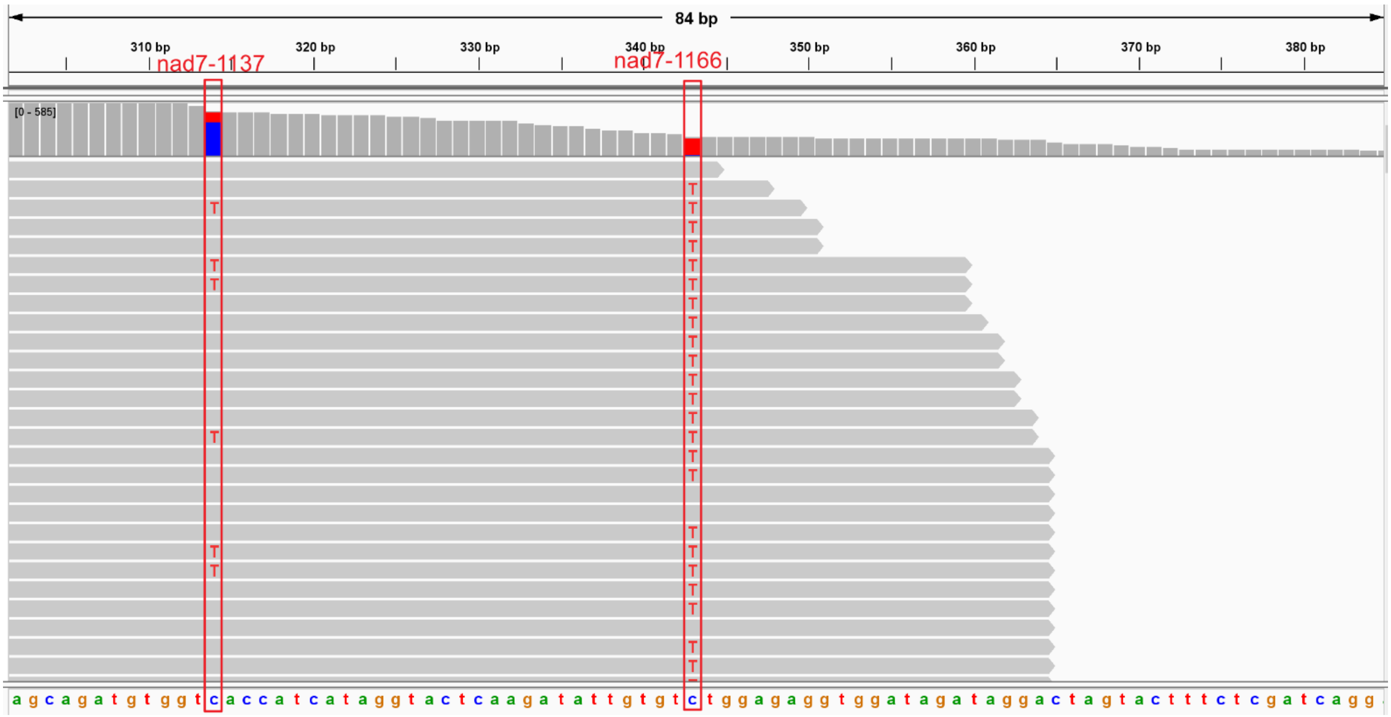
84 bp
310 bp
320 bp
330 bp
340 bp
350 bp
360 bp
370 bp
380 bp
nad7-1137
nad7-1166
[0 - 585]

w alignment of RNA-seq reads to the coding sequence of *nad9.* 9 RNA-seq editing sites: *nad9*-15, 92, 113, 167, 298, 328, 368, 398, 439 were highlighted in red squares.

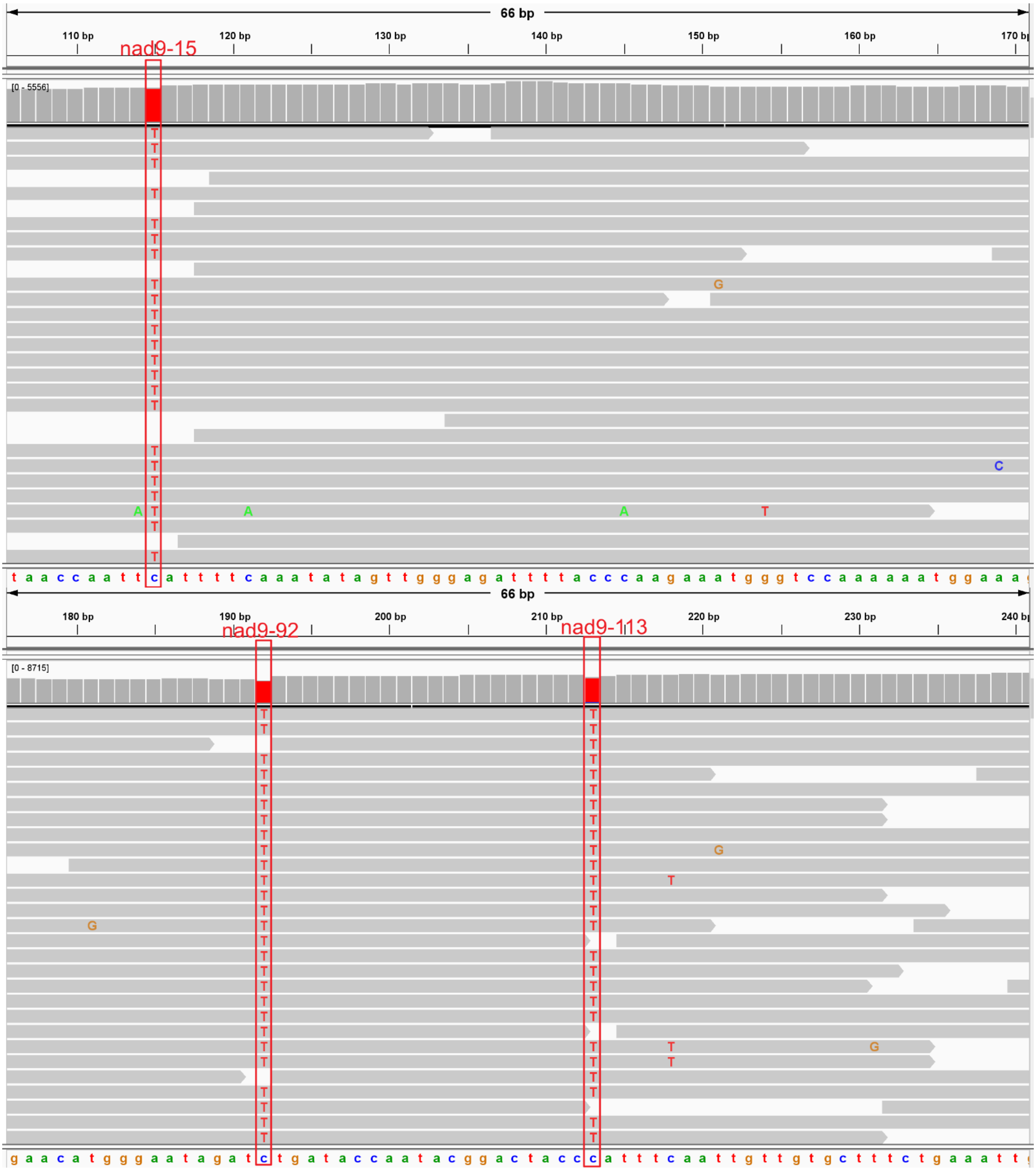

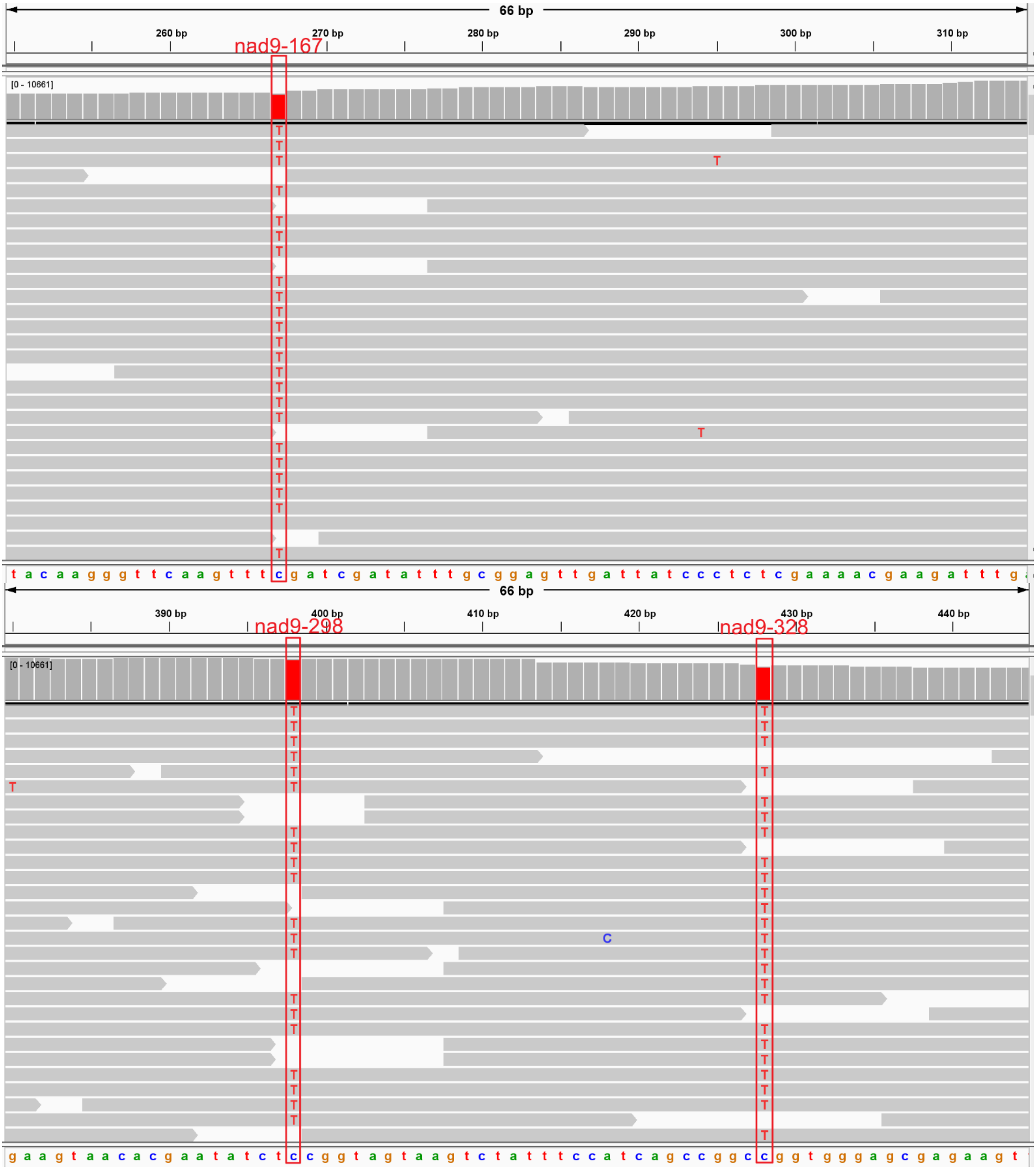
66 bp
260 bp
270 bp
280 bp
290 bp
300 bp
310 bp
nad9-167
[0 - 10661]
66 bp
390 bp
400 bp
410 bp
420 bp
430 bp
440 bp
nad9-298
nad9-328
[0 - 10661]

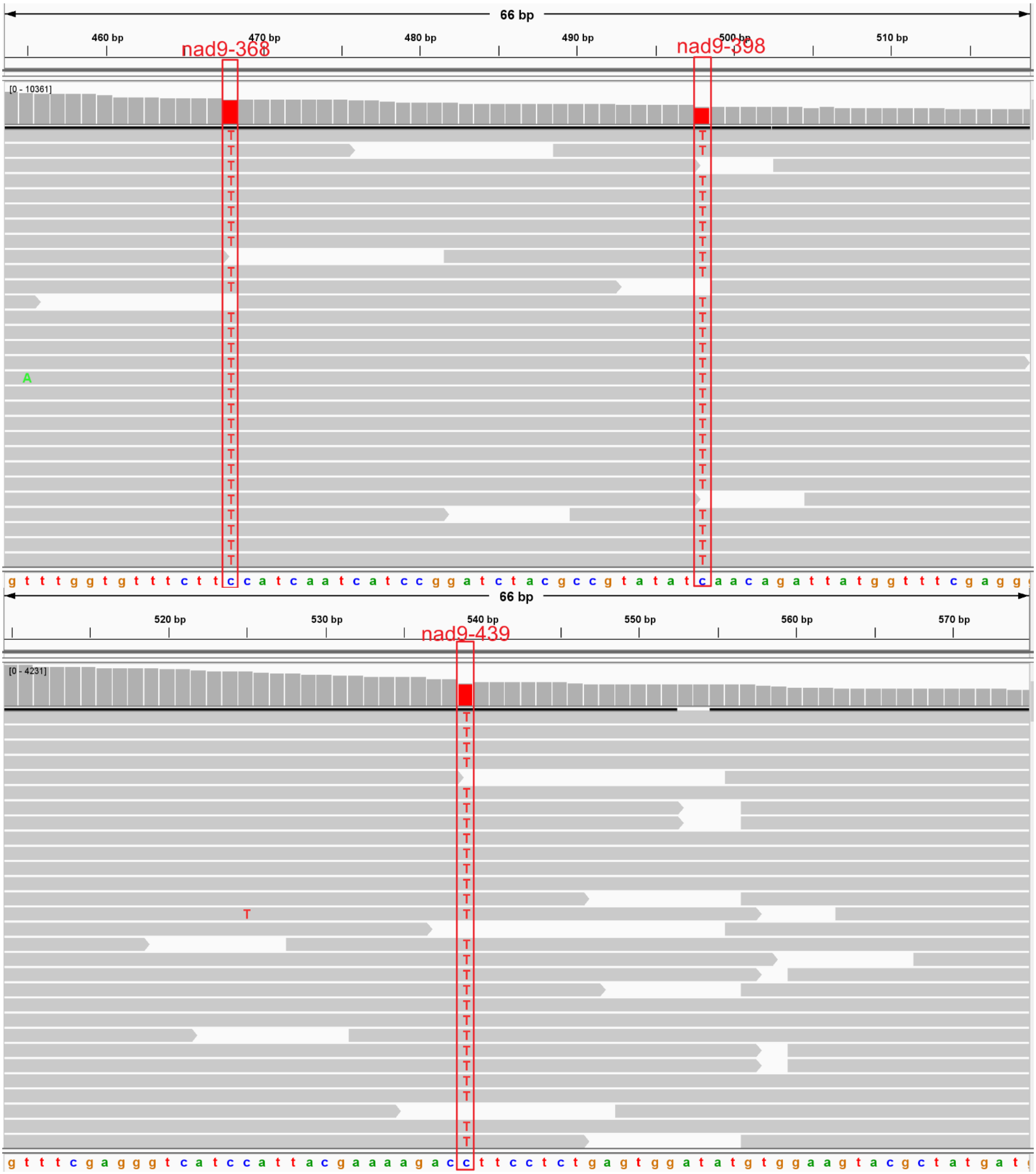
66 bp
460 bp
470 bp
480 bp
490 bp
500 bp
510 bp
nad9-368
nad9-398
[0 - 10361]
66 bp
520 bp
530 bp
540 bp
550 bp
560 bp
570 bp
nad9-439
[0 - 4231]

x alignment of RNA-seq reads to the coding sequence of *rpl5.* 11 RNA-seq editing sites: *rpl5*-8, 35, 47, 64, 160, 161, 441, 509, 512, 529, 666 were highlighted in red squares.

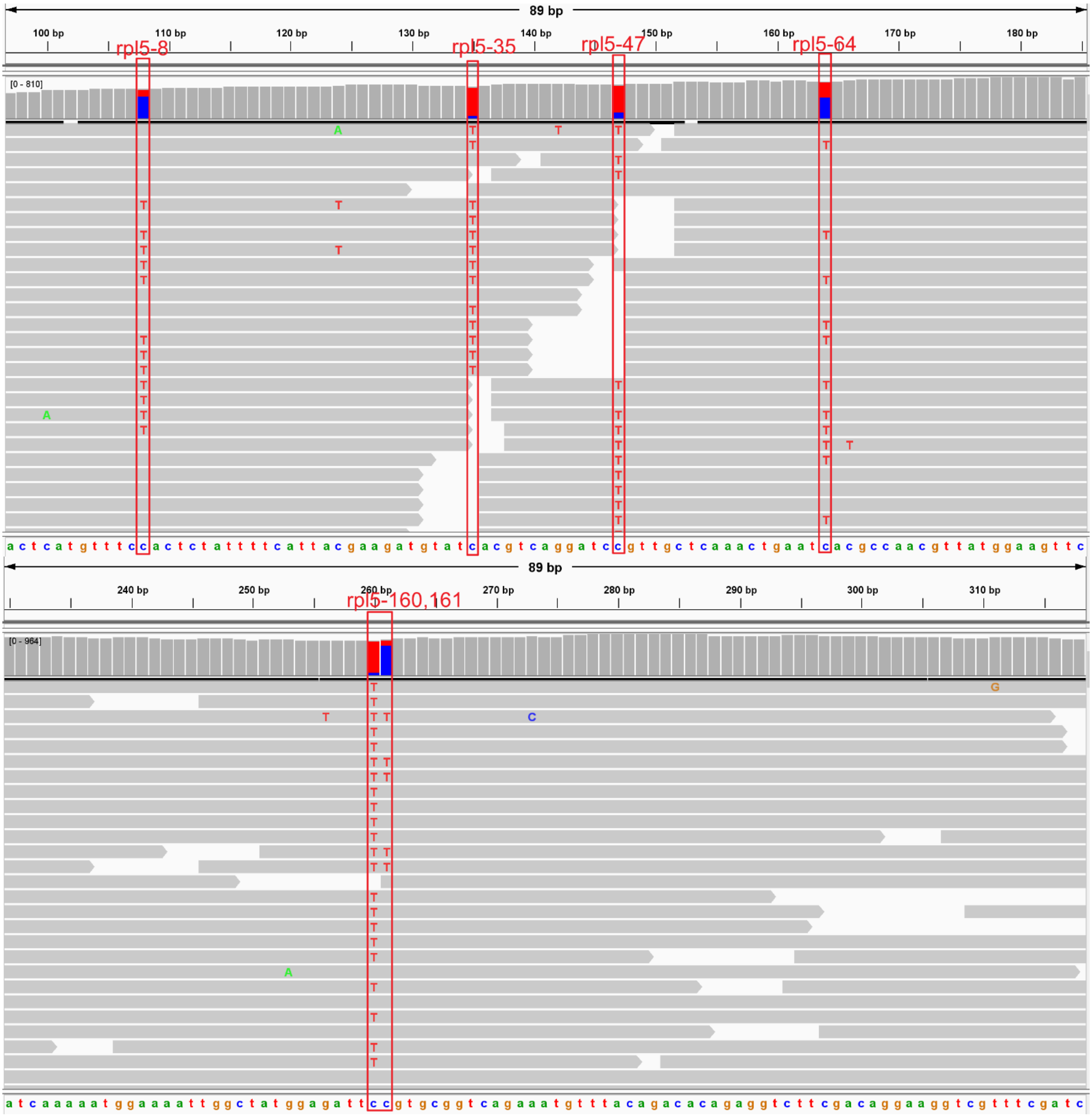

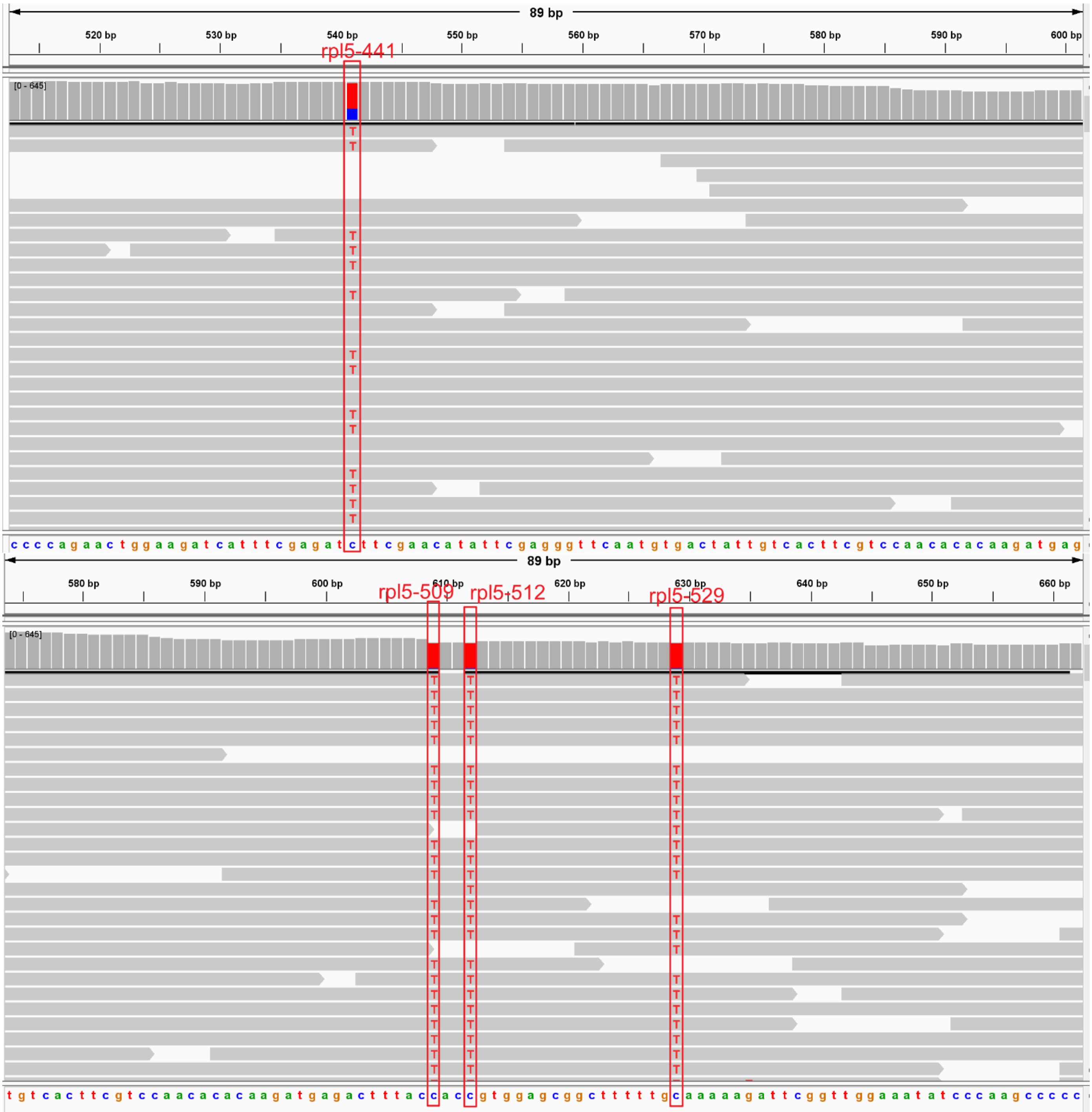
89 bp
520 bp
530 bp
540 bp
550 bp
560 bp
570 bp
580 bp
590 bp
600 bp
rpl5-441
[0 - 645]
89 bp
580 bp
590 bp
600 bp
610 bp
620 bp
630 bp
640 bp
650 bp
660 bp
rpl5-509
rpl5-512
rpl5-529
[0 - 645]

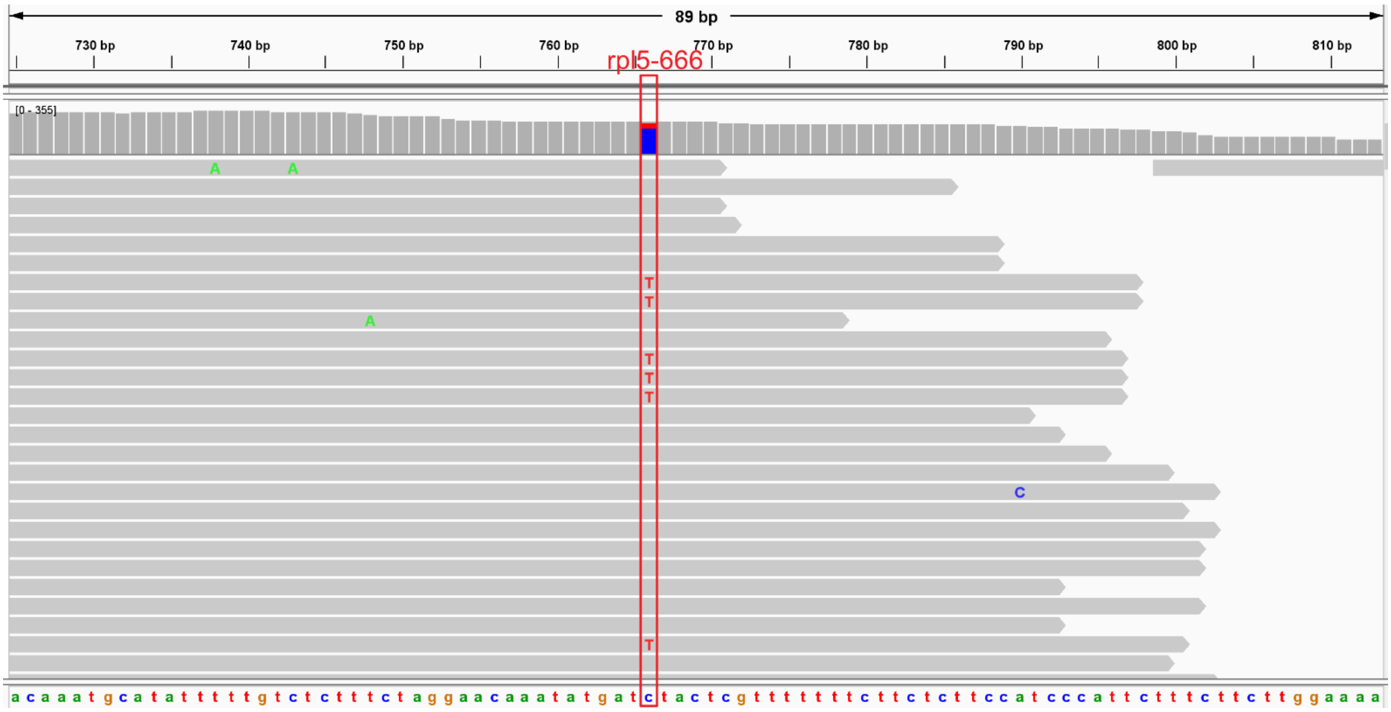

89 bp
730 bp
740 bp
750 bp
760 bp
770 bp
780 bp
790 bp
800 bp
810 bp
rpl5-666
[0 - 355]
a c a a a t g c a t a t t t t t g t c t c t t t c t a g g a a c a a a t a t g a t c t a c t c g t t t t t t t c t t c t c t t c c a t c c c a t t c t t t c t t c t t g g a a a a

y alignment of RNA-seq reads to the coding sequence of *rpl10.* Two RNA-seq editing sites: *rpl10*-101 and 134 were highlighted in red squares.

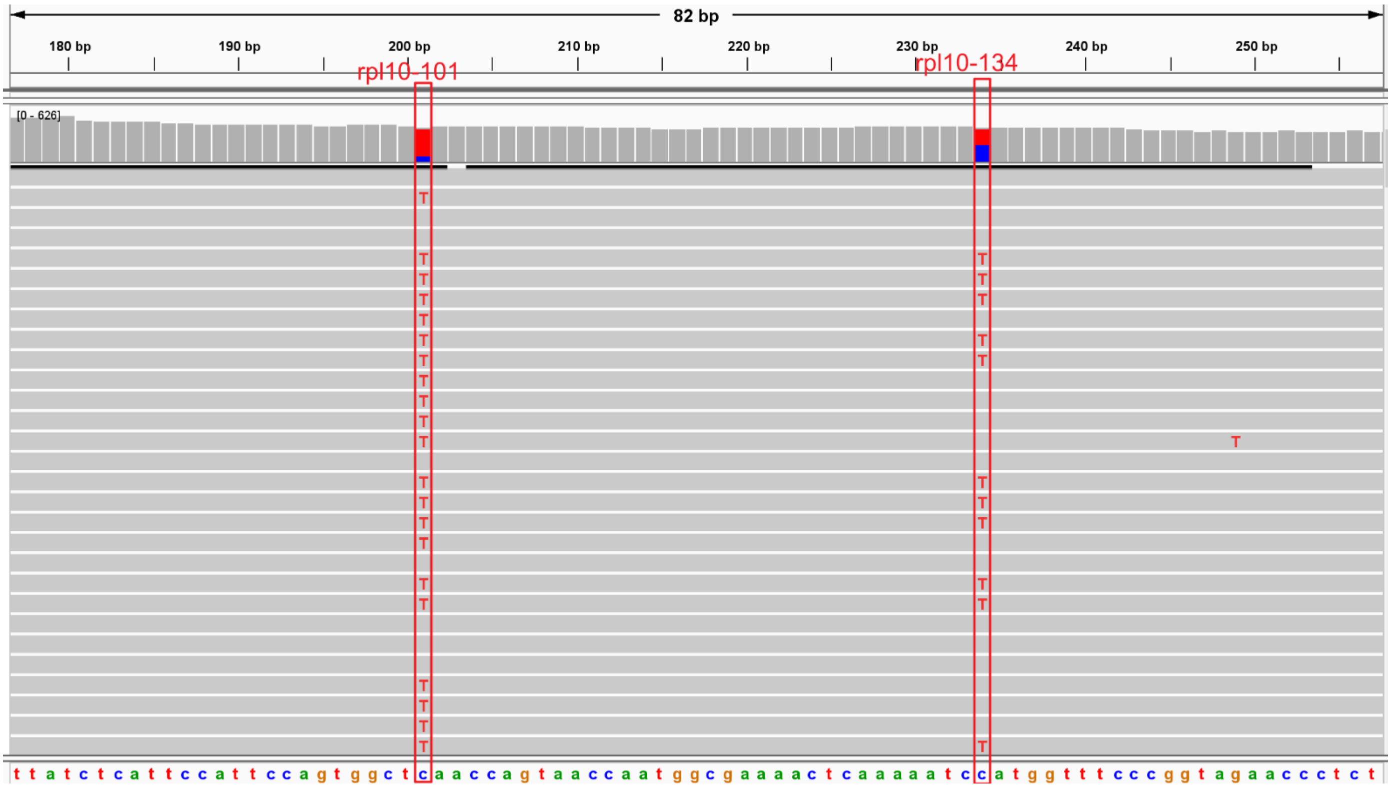

z alignment of RNA-seq reads to the coding sequence of *rpl16.* One RNA-seq editing sites: *rpl16*-221 was highlighted in red squares.

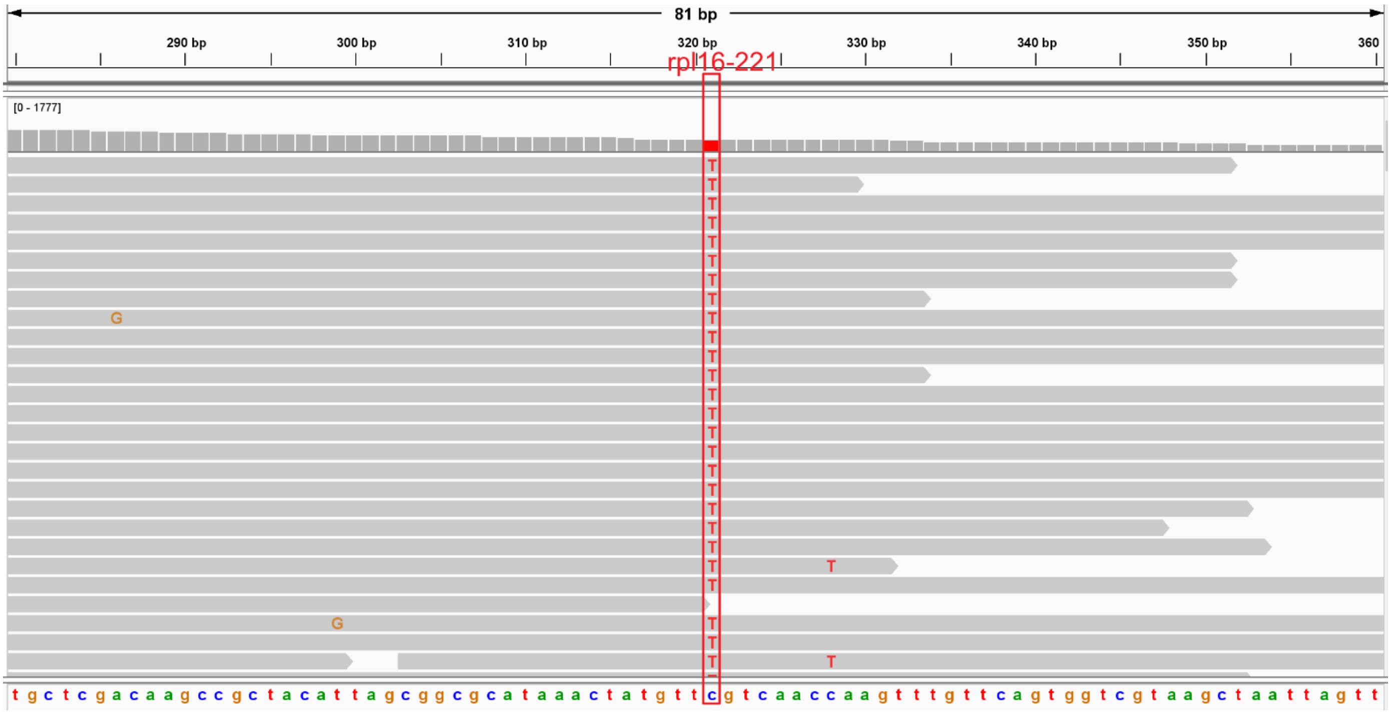

A alignment of RNA-seq reads to the coding sequence of *rps3.* Eight RNA-seq editing sites: *rps3*-92, 512, 713, 986, 1022, 1355, 1496, and 1582 were highlighted in red squares.

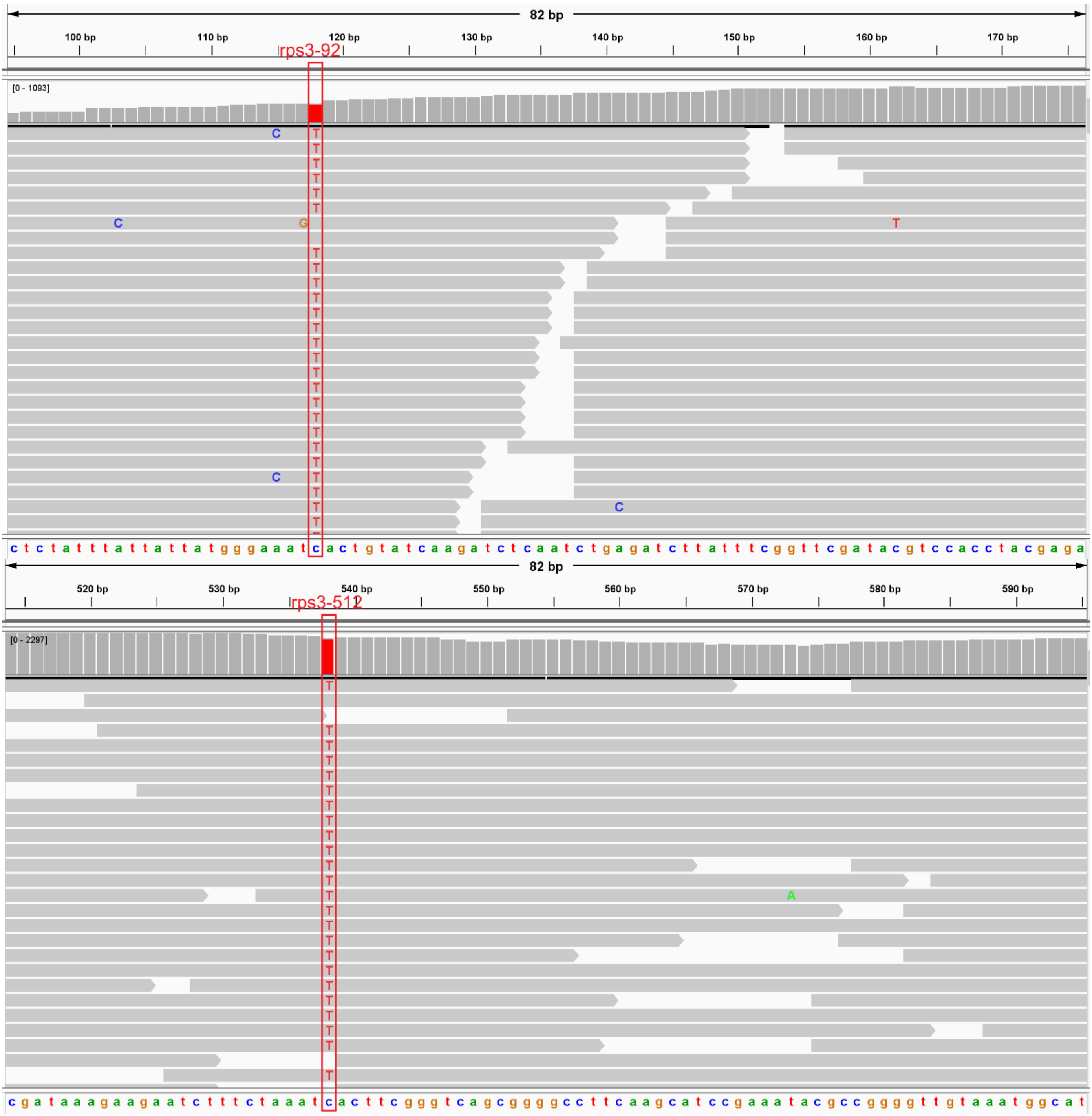

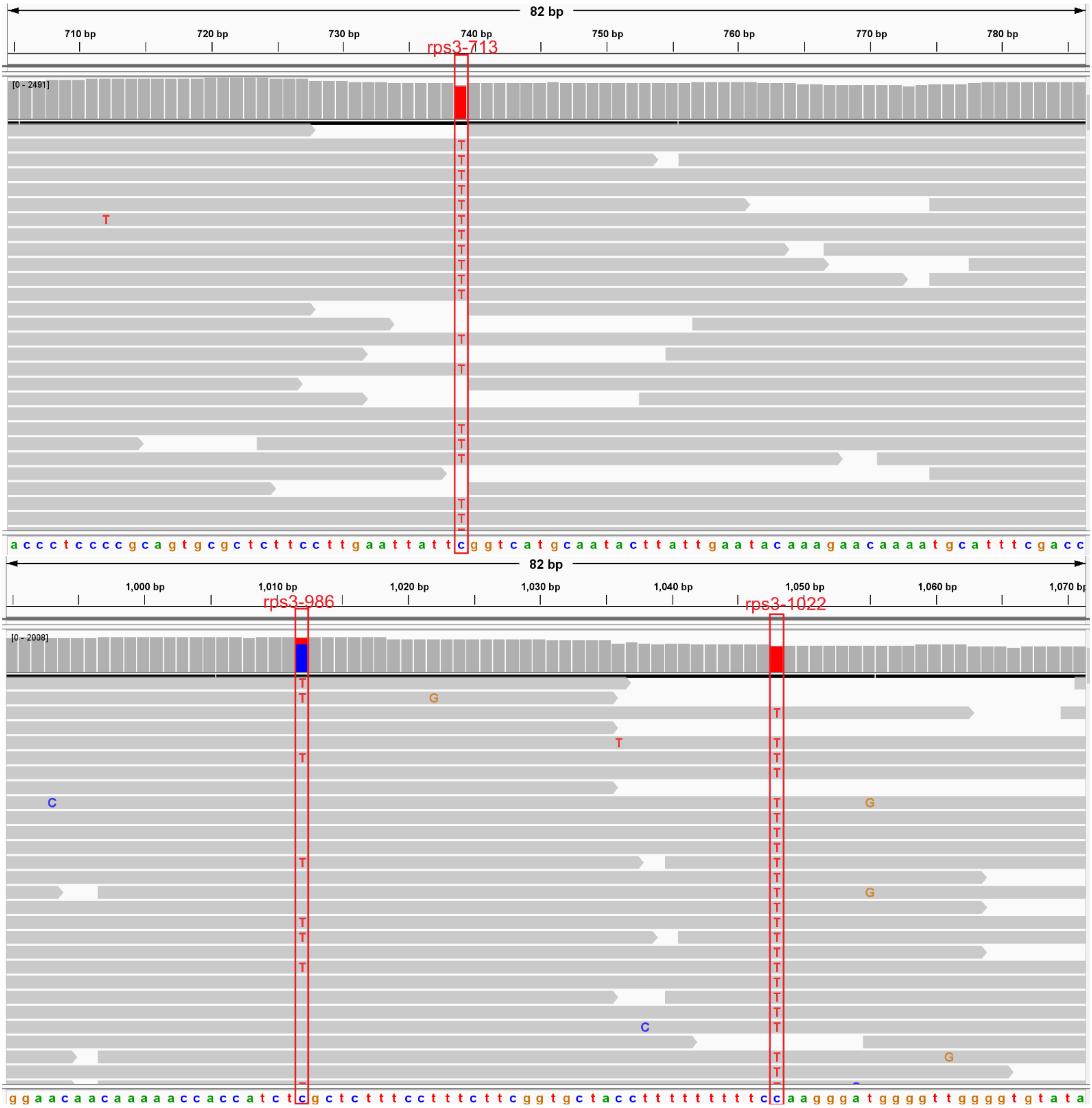
82 bp
710 bp
720 bp
730 bp
740 bp
750 bp
760 bp
770 bp
780 bp
rps3-713
[0 - 2491]
82 bp
1,000 bp
1,010 bp
1,020 bp
1,030 bp
1,040 bp
1,050 bp
1,060 bp
rps3-986
rps3-1022
[0 - 2008]

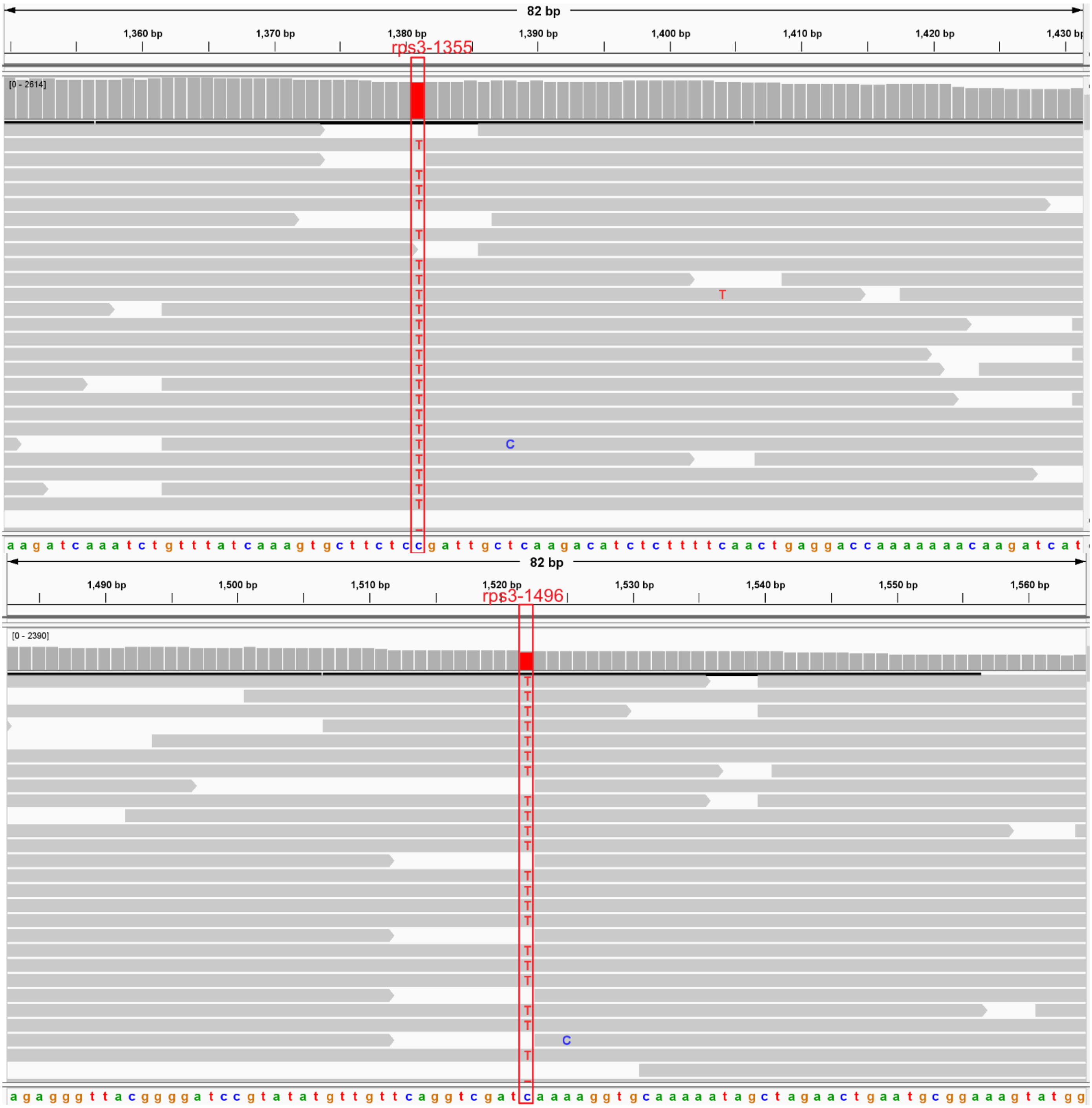
82 bp
1,360 bp
1,370 bp
1,380 bp
1,390 bp
1,400 bp
1,410 bp
1,420 bp
rps3-1355
[0 - 2614]
a a g a t c a a a t c t g t t t a t c a a a g t g c t t c t c c g a t t g c t c a a g a c a t c t c t t t t c a a c t g a g g a c c a a a a a a a c a a g a t c a t
82 bp
1,490 bp
1,500 bp
1,510 bp
1,520 bp
1,530 bp
1,540 bp
1,550 bp
1,560 bp
rps3-1496
[0 - 2390]
a g a g g g t t a c g g g g a t c c g t a t a t g t t g t t c a g g t c g a t c a a a a g g t g c a a a a a t a g c t a g a a c t g a a t g c g g a a a g t a t g g

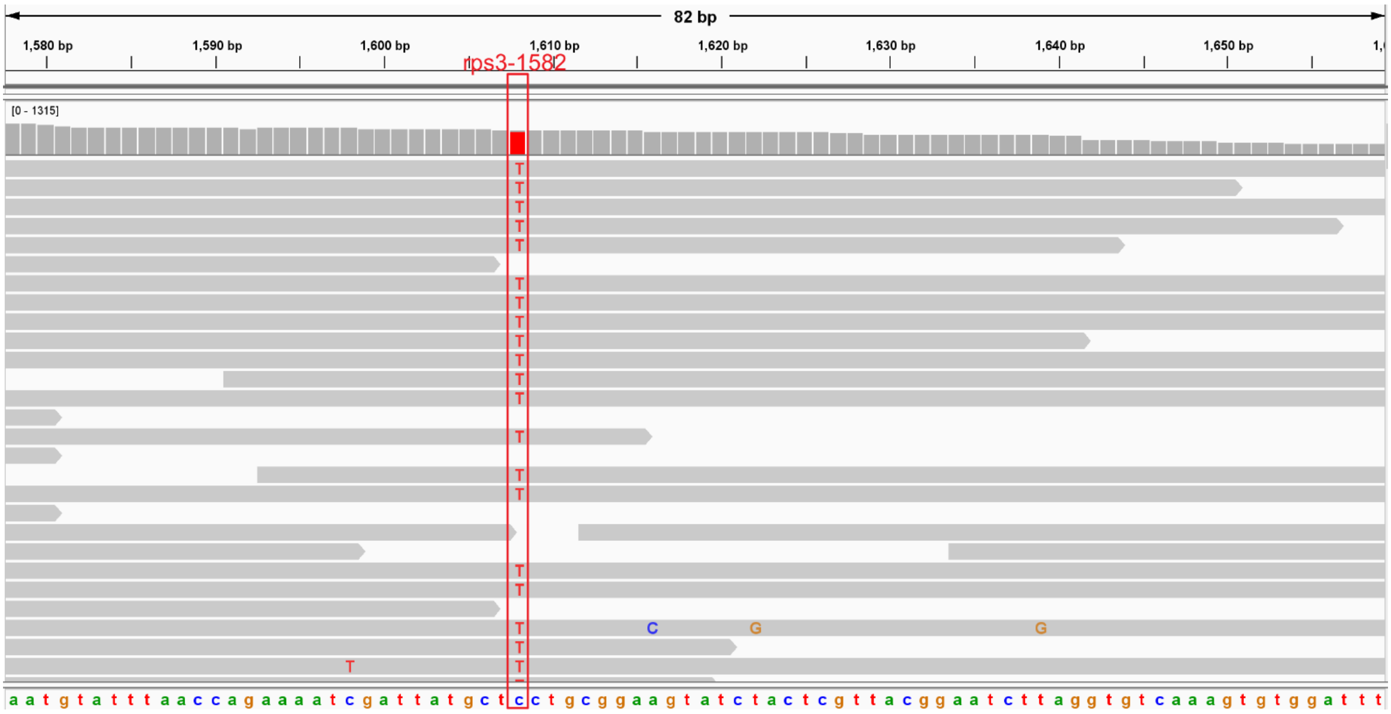
82 bp
1,580 bp
1,590 bp
1,600 bp
1,610 bp
1,620 bp
1,630 bp
1,640 bp
1,650 bp
rps3-1582
[0 - 1315]

B alignment of RNA-seq reads to the coding sequence of *rps4.* 20 RNA-seq editing sites: *rps4*-176, 205, 219, 275, 287, 299, 316, 344, 443, 483, 491, 592, 848, 852, 941, 952, 962, 977, 1028, 1042 were highlighted in red squares.

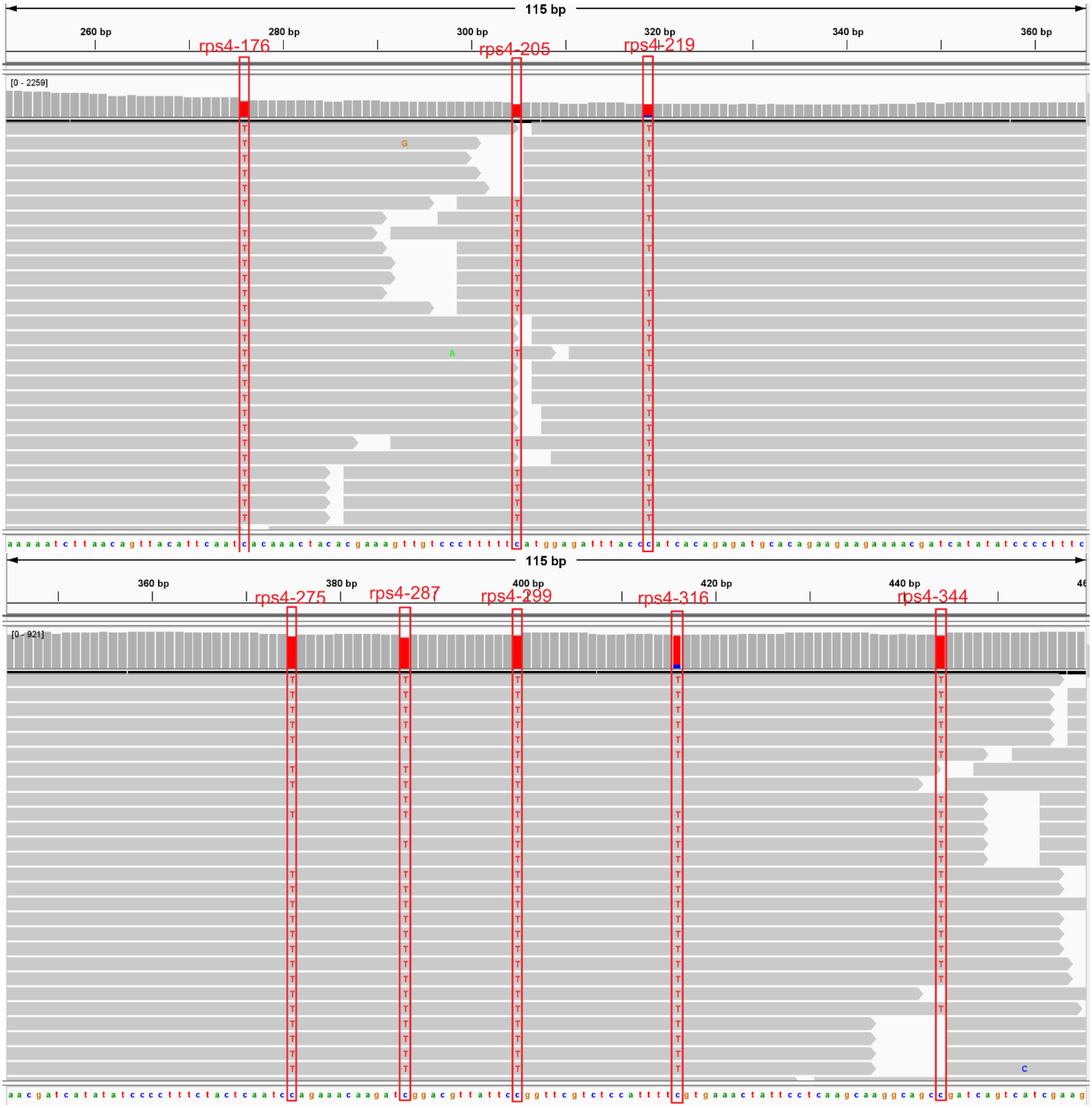

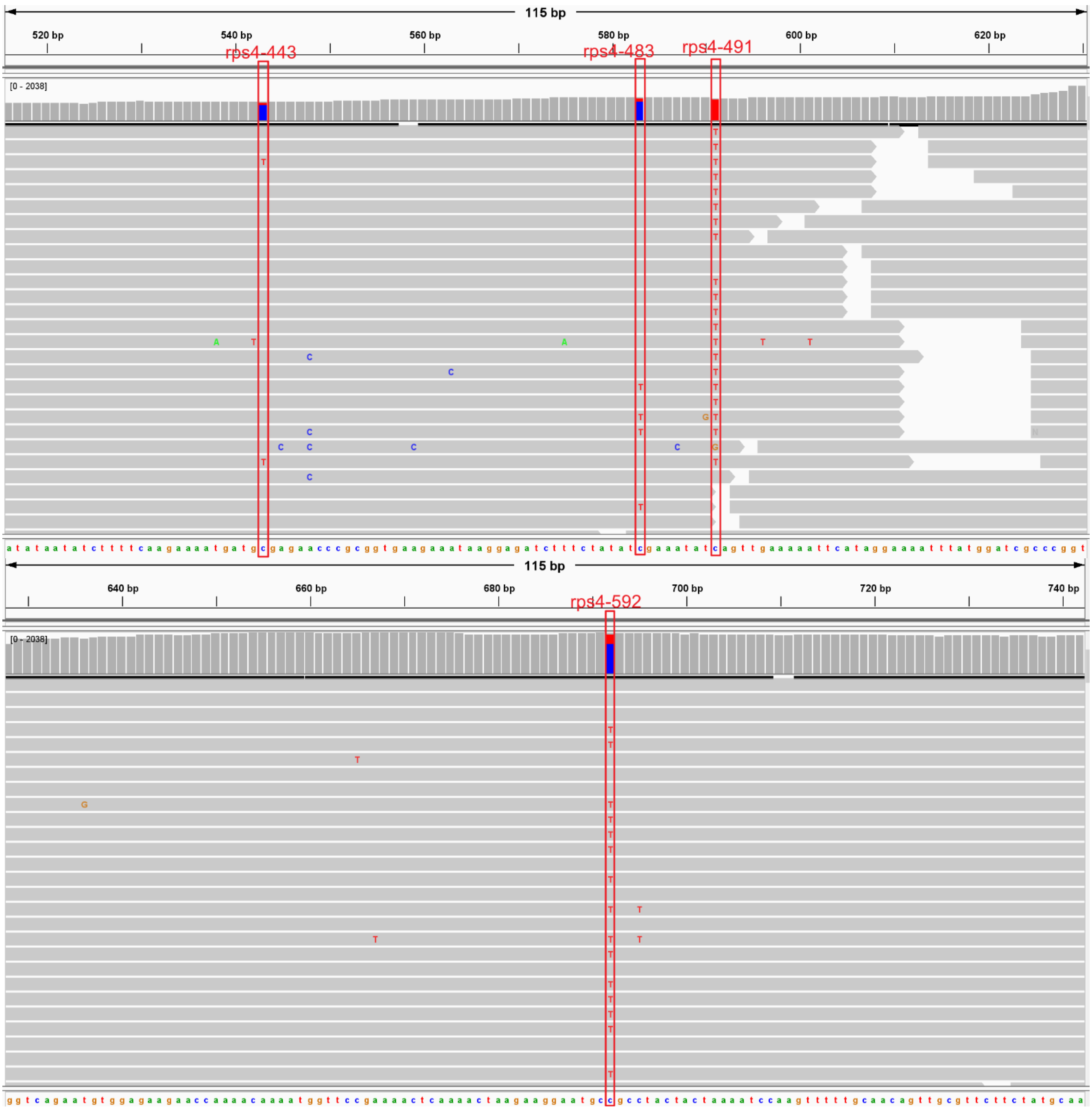
115 bp
520 bp
540 bp
560 bp
580 bp
600 bp
620 bp
rps4-443
rps4-483
rps4-491
[0 - 2038]
115 bp
640 bp
660 bp
680 bp
700 bp
720 bp
740 bp
rps4-592
[0 - 2038]

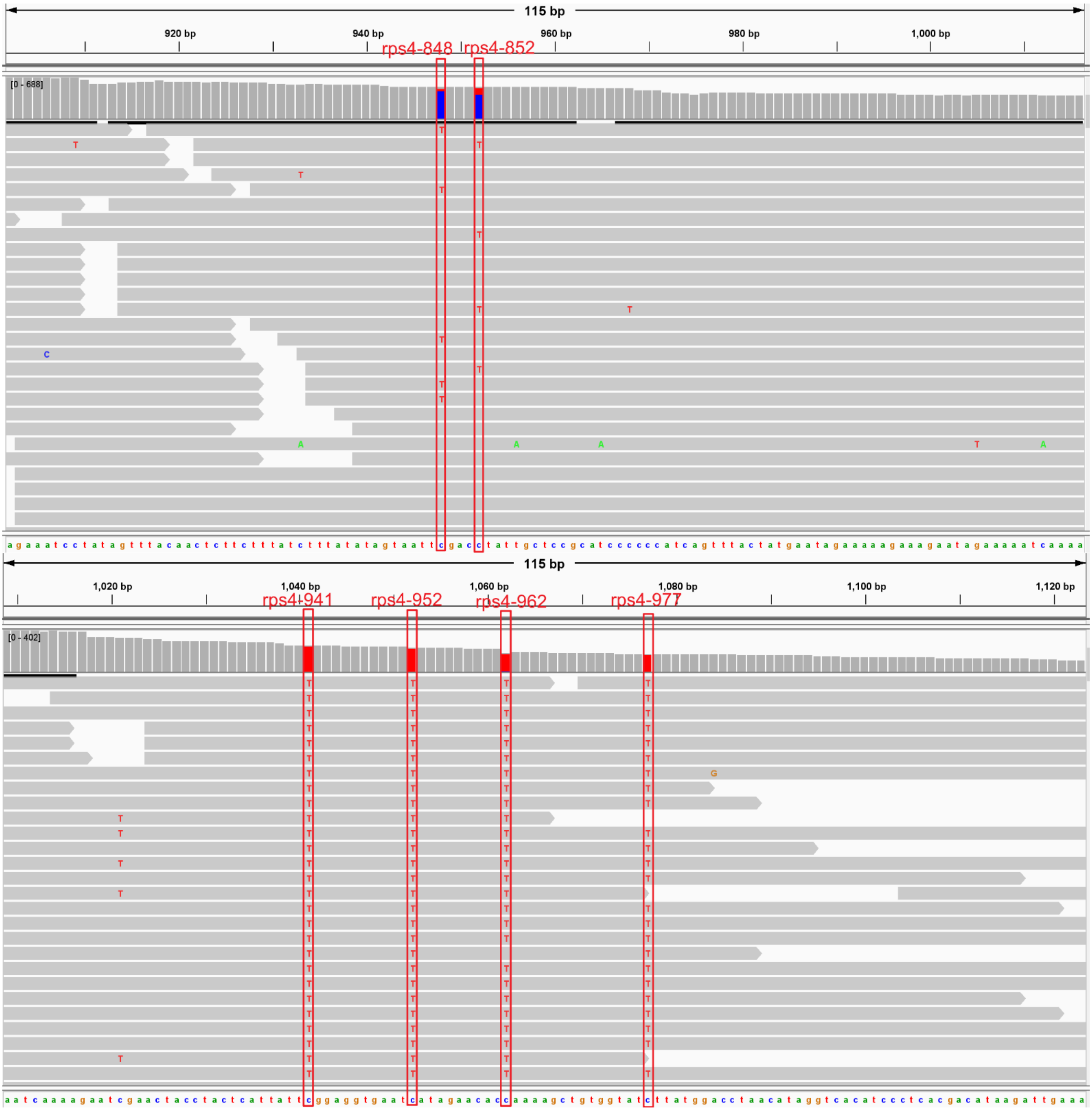
115 bp
920 bp
940 bp
960 bp
980 bp
1,000 bp
rps4-848
rps4-852
[0 - 688]
115 bp
1,020 bp
1,040 bp
1,060 bp
1,080 bp
1,100 bp
1,120 bp
rps4-941
rps4-952
rps4-962
rps4-977
[0 - 402]

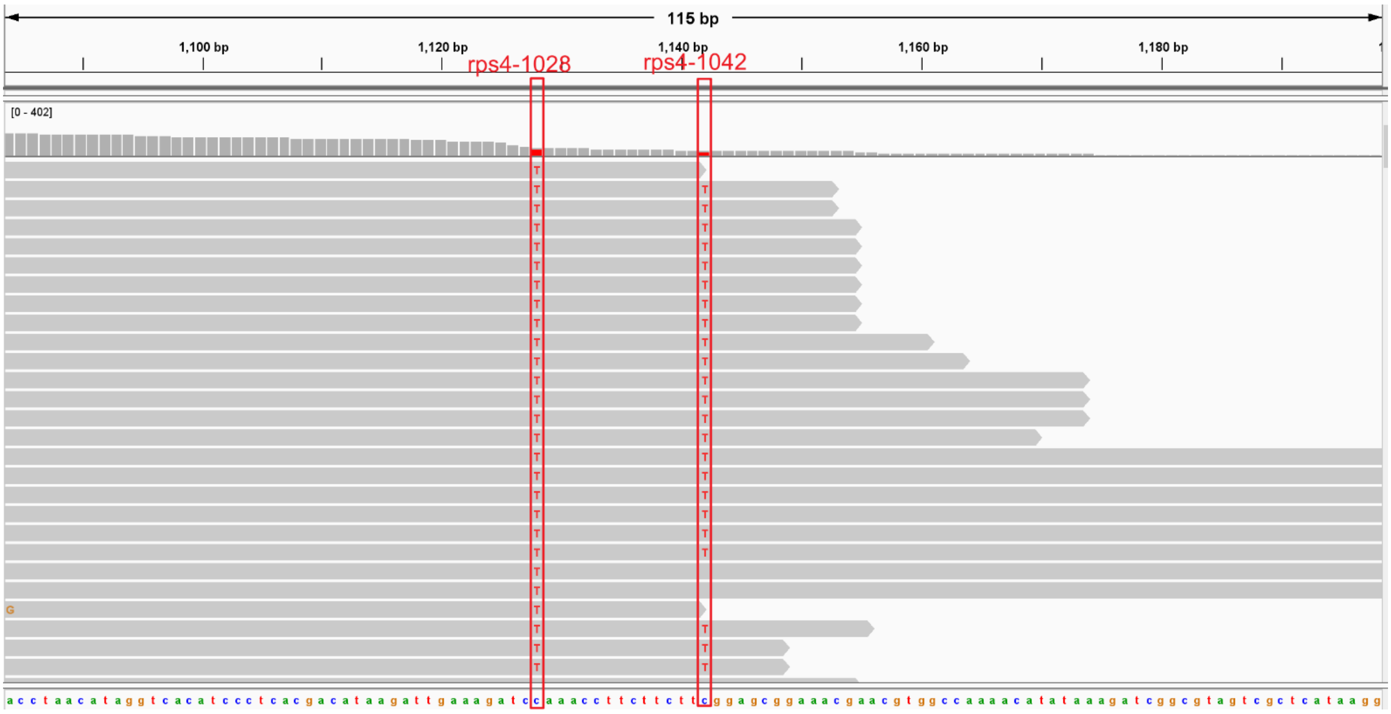
115 bp
1,100 bp
1,120 bp
1,140 bp
1,160 bp
1,180 bp
rps4-1028
rps4-1042
[0 - 402]

C alignment of RNA-seq reads to the coding sequence of *rps10.* Six RNA-seq editing sites: *rps10*-102, 132, 210, 214, 278, 307 were highlighted in red squares.

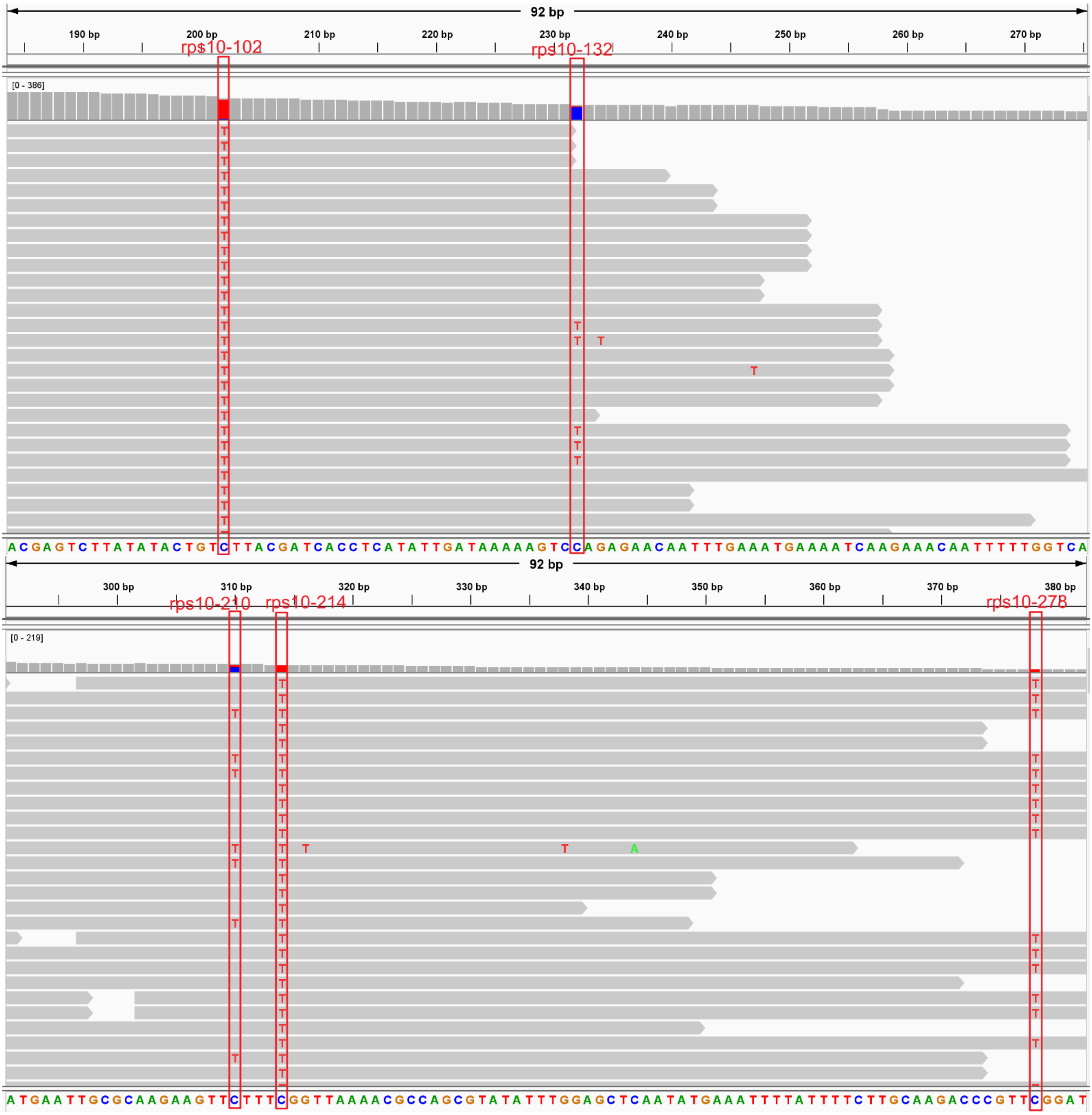

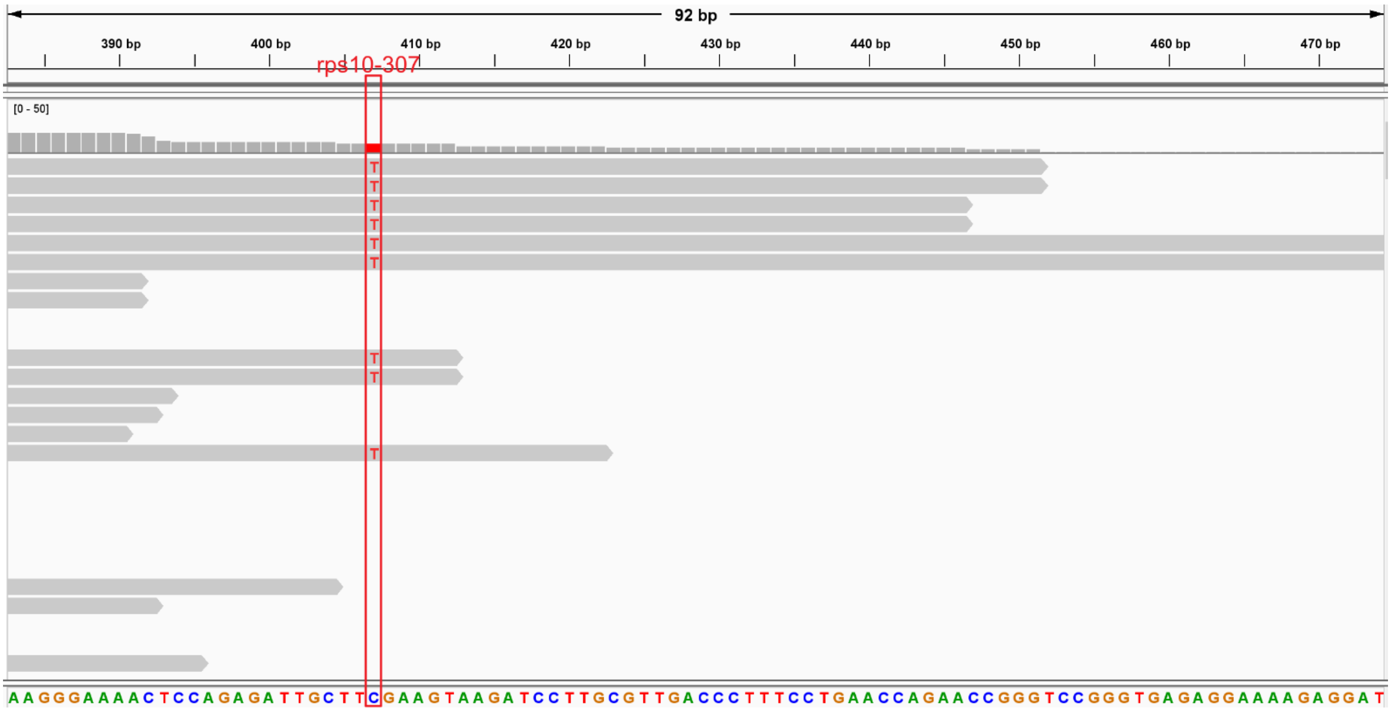
92 bp
390 bp
400 bp
410 bp
420 bp
430 bp
440 bp
450 bp
460 bp
470 bp
rps10-307
[0 - 50]

D alignment of RNA-seq reads to the coding sequence of *rps12.* Five RNA-seq editing sites: *rps12*-104, 159, 196, 221, 284 were highlighted in red squares.

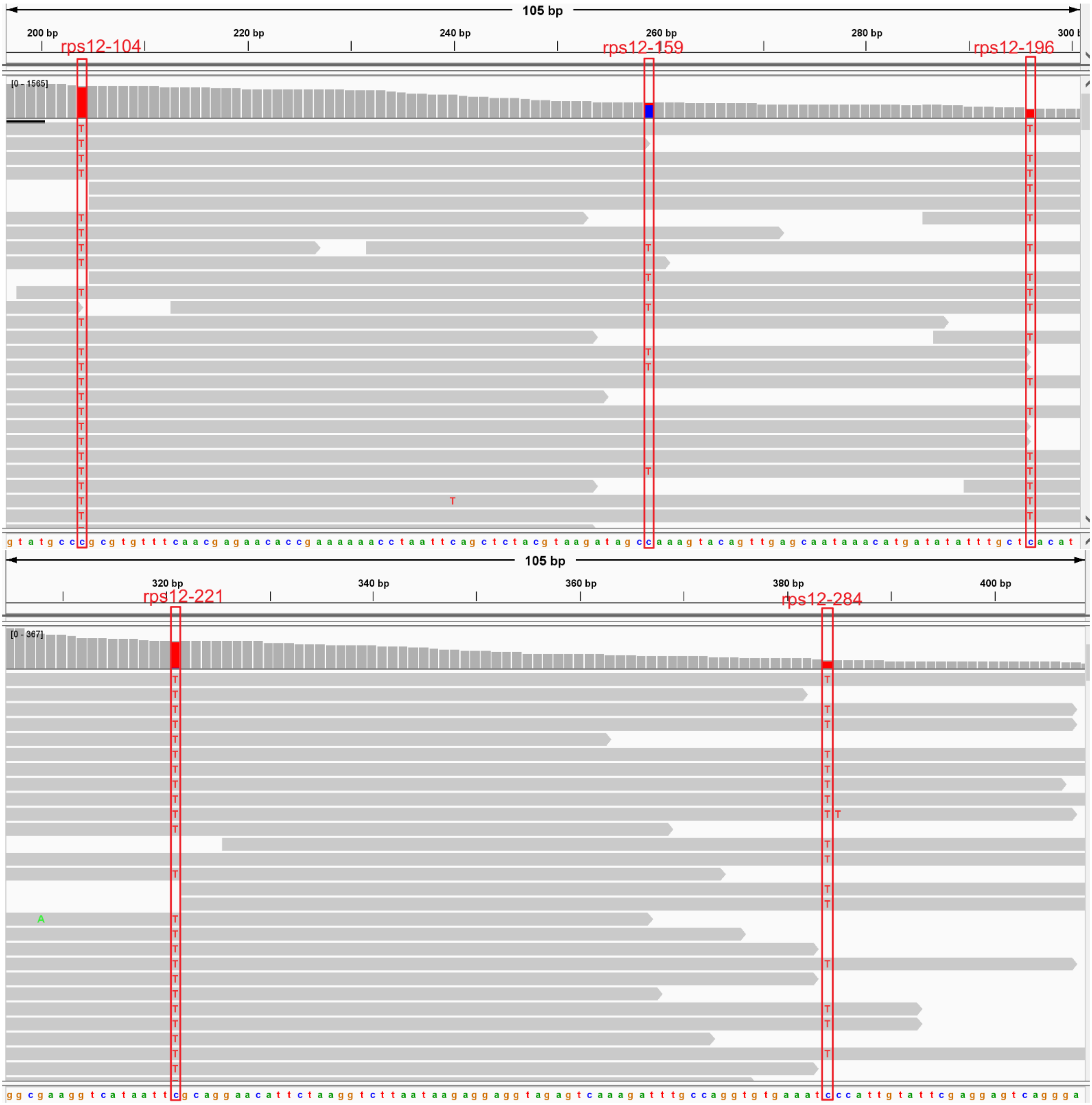

E alignment of RNA-seq reads to the coding sequence of *rps13.* Three RNA-seq editing sites: *rps13*-56, 100, 287 were highlighted in red squares.

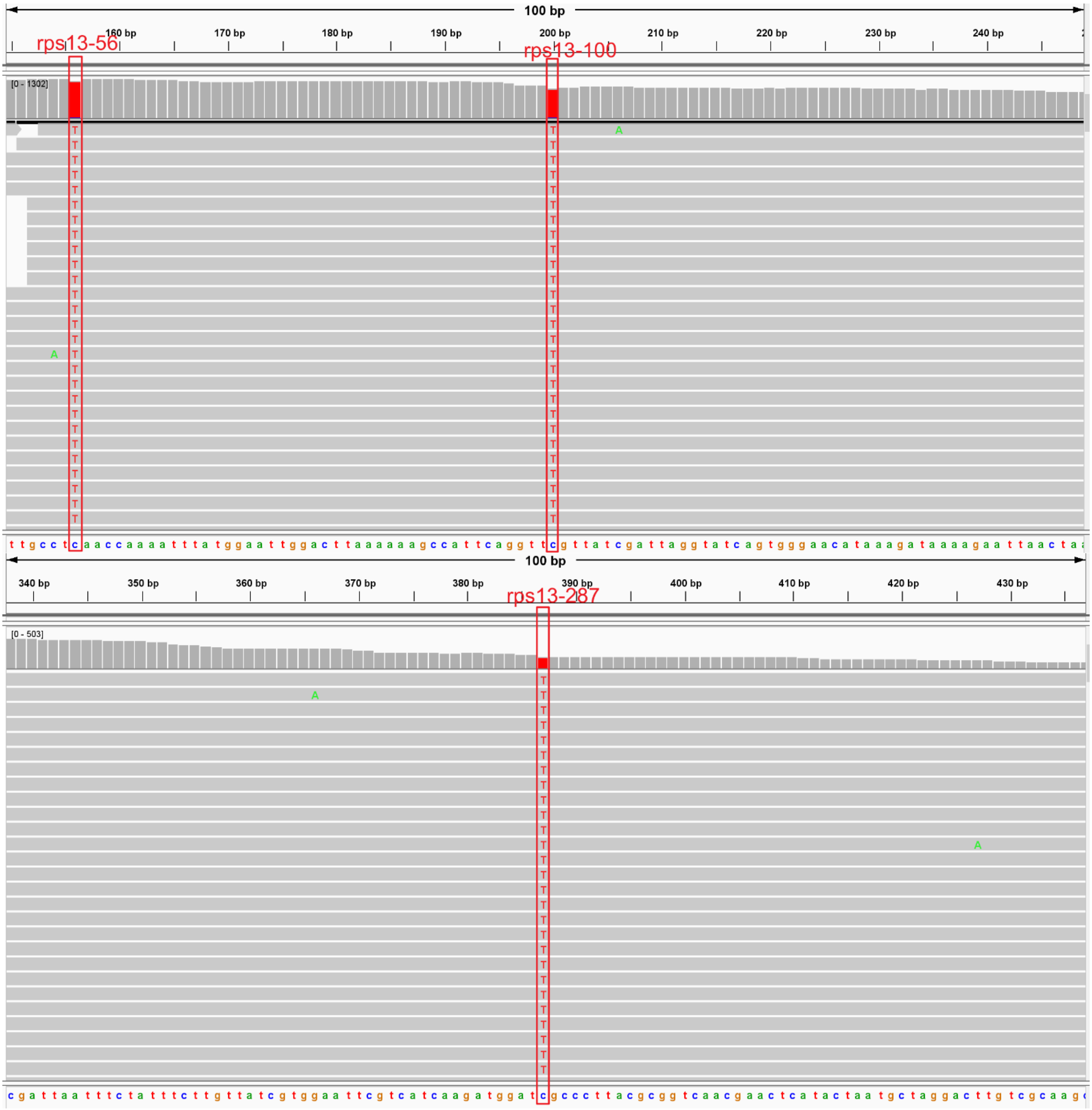

Figure S7 Validation of the RNA editing sites within eleven PCGs using PCR and Sanger sequencing. The top panel shows the sequences and chromatographs from the PCR products ampli-fied using the genomic DNA (gDNA) and complementary DNA (cDNA) as templates. The bottom panel shows the sequences from the PCR products amplified using the gDNA and cDNA. The gene names and the RNA editing sites' positions on the CDs are shown at the bottom of the figure, separated with “-”. The red squares highlight the focal RNA ed-iting site.

a validation of the *atp4*-59, *atp4*-71, *atp4*-89, *atp4*-118, *atp4*-215, *atp4*-227, *atp4*-248, *atp4*-251, *atp4*-395, *atp4*-407, and *atp4*-416 using PCR amplification and Sanger sequencing experiments.

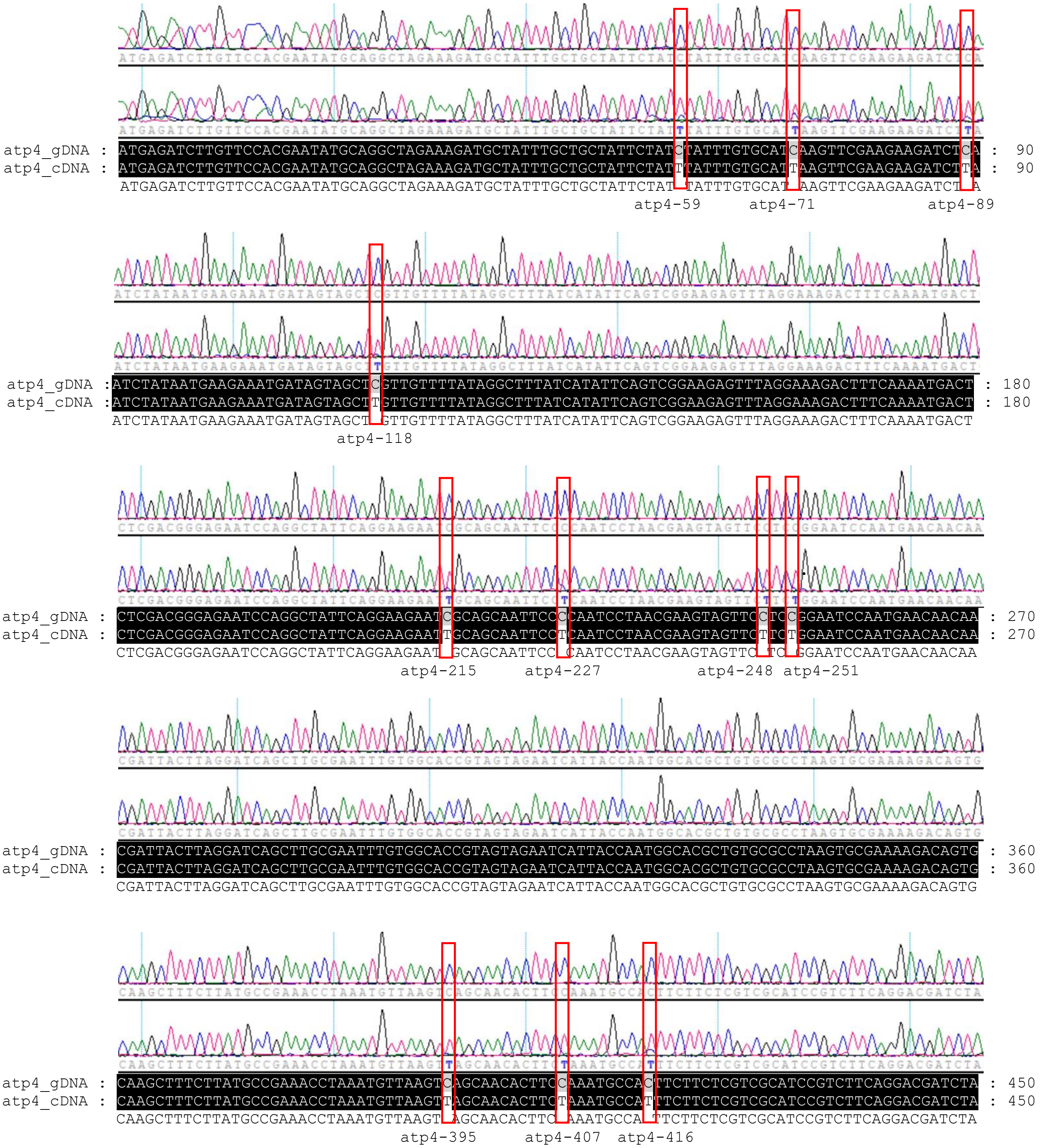

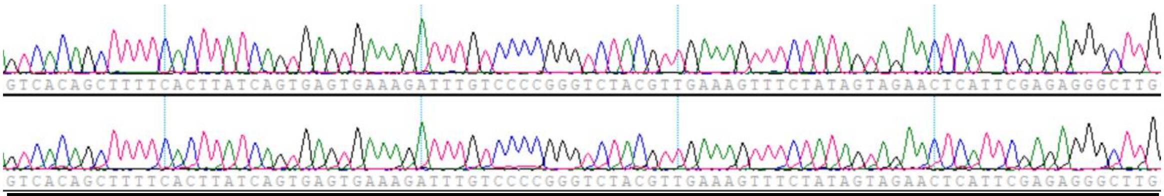

atp4_gDNA : GTCACAGCTTTTCACTTATCAGTGAGTGAAAGATTTGTCCCCGGGTCTACGTTGAAAGTTTCTATAGTAGAACTCATTCGAGAGGGCTTG : 540
atp4_cDNA : GTCACAGCTTTTCACTTATCAGTGAGTGAAAGATTTGTCCCCGGGTCTACGTTGAAAGTTTCTATAGTAGAACTCATTCGAGAGGGCTTG : 540
GTCACAGCTTTTCACTTATCAGTGAGTGAAAGATTTGTCCCCGGGTCTACGTTGAAAGTTTCTATAGTAGAACTCATTCGAGAGGGCTTG

b validation of the *atp8*-58 using PCR amplification and Sanger sequencing experiments.

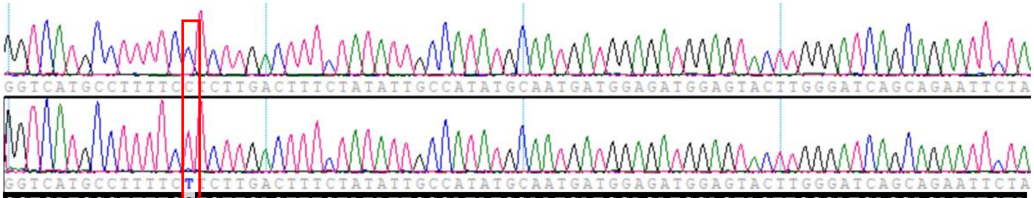

atp8_gDNA : GGTCATGCCTTTTCCTCTTGACTTTCTATATTGCCATATGCAATGATGGAGATGGAGTACTTGGGATCAGCAGAATTCTA : 80
atp8_cDNA : GGTCATGCCTTTTCTTCTTGACTTTCTATATTGCCATATGCAATGATGGAGATGGAGTACTTGGGATCAGCAGAATTCTA : 80
GGTCATGCCTTTTC TCTTGACTTTCTATATTGCCATATGCAATGATGGAGATGGAG**T**ACTTGGGATCAGCAGAATTCTA
atp8-58

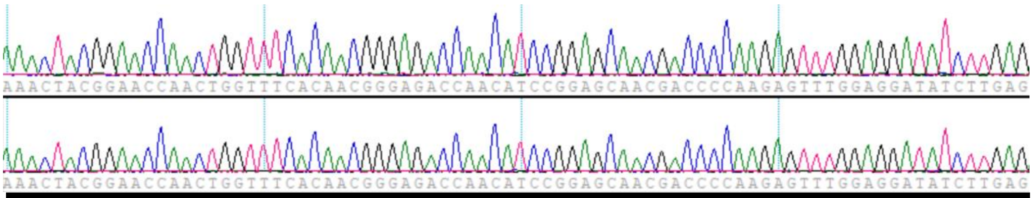

atp8_gDNA : AAACTACGGAACCAACTGGTTTCACAACGGGAGACCAACATCCGGAGCAACGACCCCAAGAGTTTGGAGGATATCTTGAG : 160
atp8_cDNA : AAACTACGGAACCAACTGGTTTCACAACGGGAGACCAACATCCGGAGCAACGACCCCAAGAGTTTGGAGGATATCTTGAG : 160
AAACTACGGAACCAACTGGTTTCACAACGGGAGACCAACATCCGGAGCAACGACCCCAAGAGTTTGGAGGATATCTTGAG

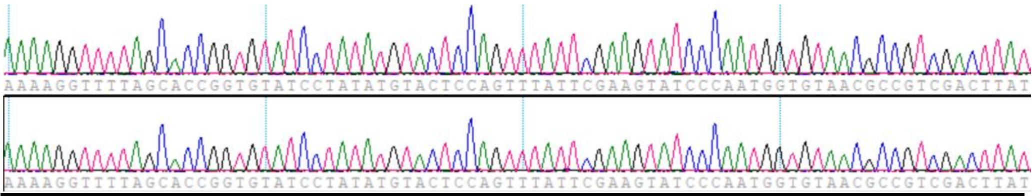

atp8_gDNA : AAAAGGTTTAGCACCGGTGTATCCTATATGTACTCCAGTTTATTCGAAGTATCCCAATGGTGTAACGCCGTCGACTTAT : 240
atp8_cDNA : AAAAGGTTTAGCACCGGTGTATCCTATATGTACTCCAGTTTATTCGAAGTATCCCAATGGTGTAACGCCGTCGACTTAT : 240
AAAAGGTTTAGCACCGGTGTATCCTATATGTACTCCAGTTTATTCGAAGTATCCCAATGGTGTAACGCCGTCGACTTAT

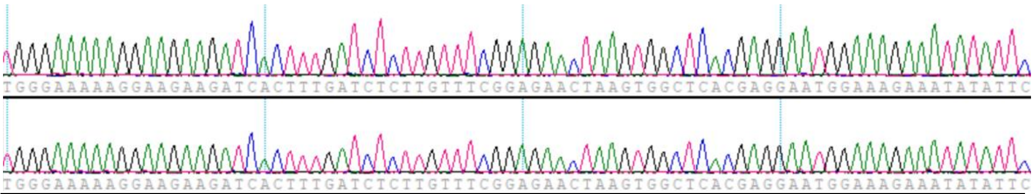

atp8_gDNA : TGGGAAAAAGGAAGAAGATCACTTTGATCTCTTGTTTCGGAGAACTAAGTGGCTCACGAGGAATGGAAAGAAATATATTC : 320
atp8_cDNA : TGGGAAAAAGGAAGAAGATCACTTTGATCTCTTGTTTCGGAGAACTAAGTGGCTCACGAGGAATGGAAAGAAATATATTC : 320
TGGGAAAAAGGAAGAAGATCACTTTGATCTCTTGTTTCGGAGAACTAAGTGGCTCACGAGGAATGGAAAGAAATATATTC

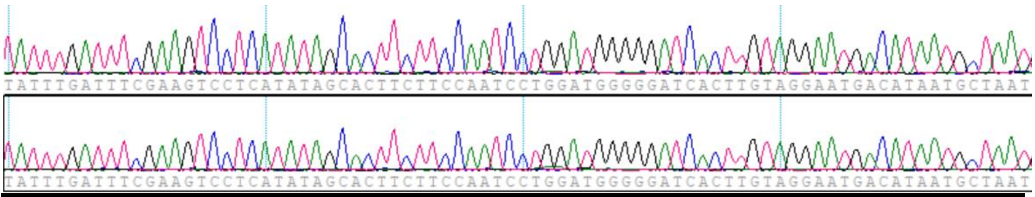

atp8_gDNA : TATTTGATTTCGAAGTCCTCATATAGCACTTCTTCCAATCCTGGATGGGGGATCACTTGTAGGAATGACATAATGCTAAT : 400
atp8_cDNA : TATTTGATTTCGAAGTCCTCATATAGCACTTCTTCCAATCCTGGATGGGGGATCACTTGTAGGAATGACATAATGCTAAT : 400
TATTTGATTTCGAAGTCCTCATATAGCACTTCTTCCAATCCTGGATGGGGGATCACTTGTAGGAATGACATAATGCTAAT

c validation of the *cox1*-242, *cox1*-254, *cox1*-452, *cox1*-515, *cox1*-551, *cox1*-590, *cox1*-715, *cox1*-1186, *cox1*-1405, *cox1*-1433, and *cox1*-1499 using PCR amplification and Sanger sequencing experiments.

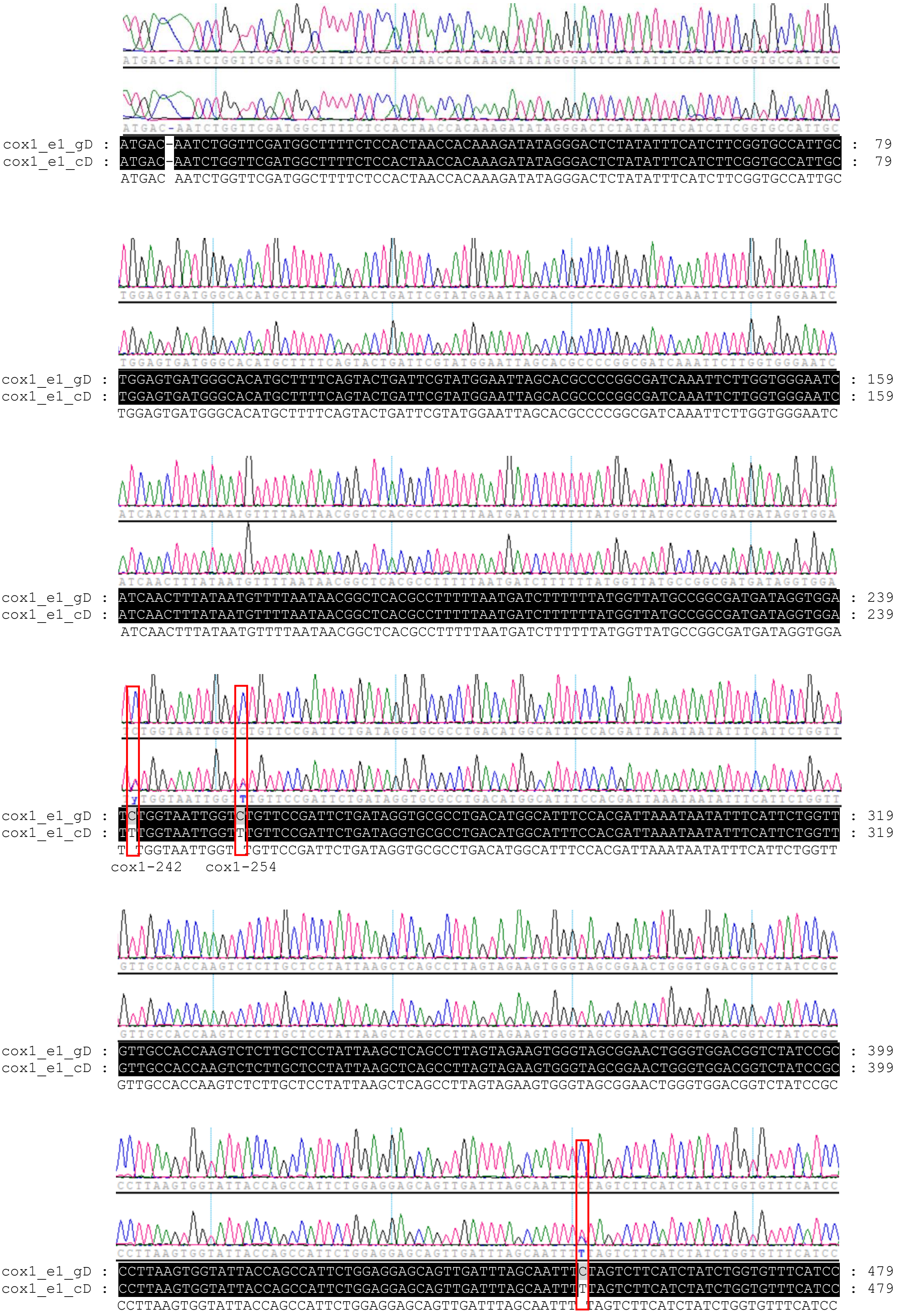

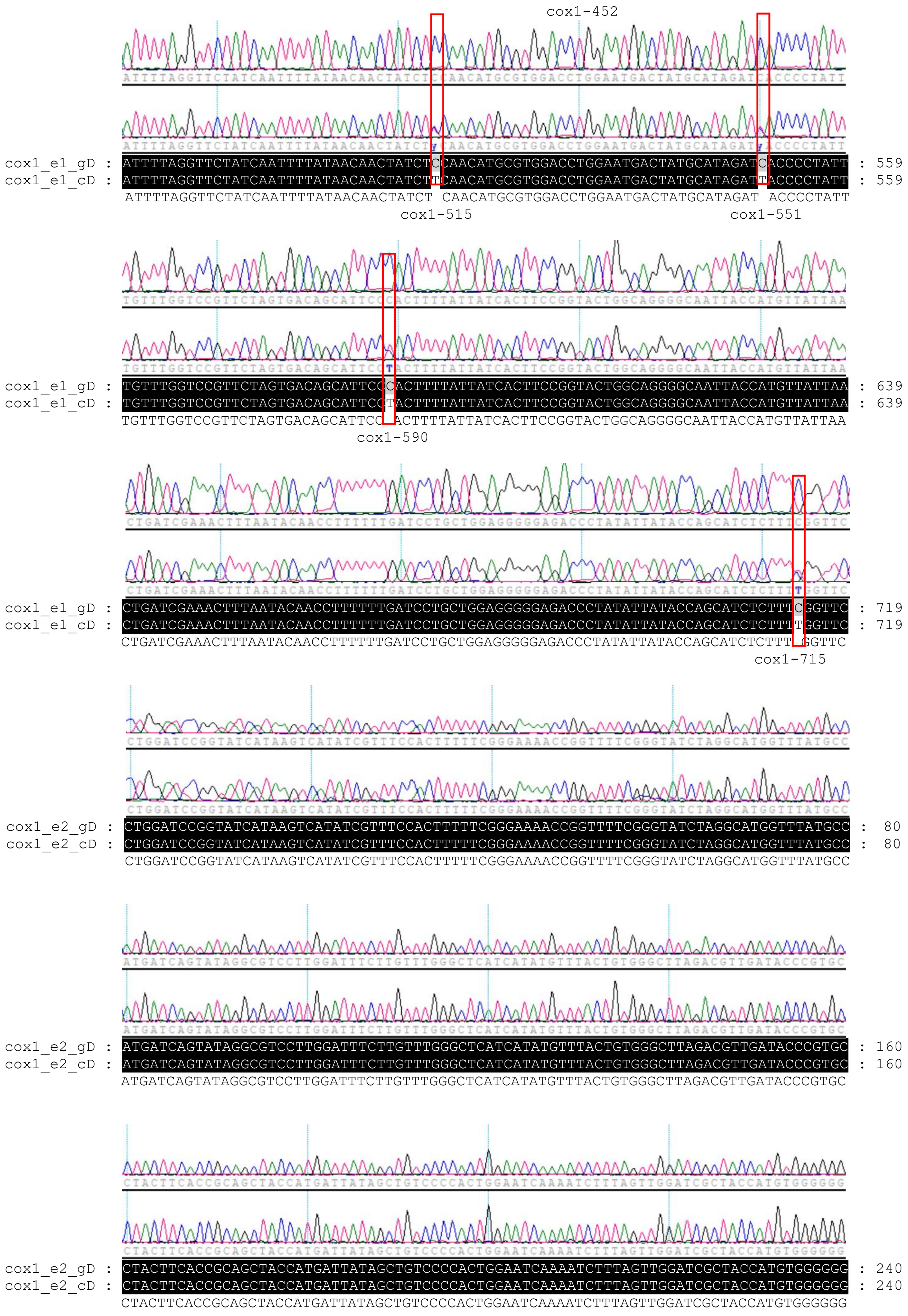

cox1-452
cox1_e1_gD : ATTTTAGGTTCTATCAATTTTATAACAACTATCTCCAACATGCGTGGACCTGGAATGACTATGCATAGATCACCCCTATT : 559
cox1_e1_cD : ATTTTAGGTTCTATCAATTTTATAACAACTATCTTCAACATGCGTGGACCTGGAATGACTATGCATAGATTACCCCTATT : 559
ATTTTAGGTTCTATCAATTTTATAACAACTATCT CAACATGCGTGGACCTGGAATGACTATGCATAGAT ACCCCTATT
cox1-515
cox1-551
cox1_e1_gD : TGTTTGGTCCGTTCTAGTGACAGCATTCCCACTTTTATTATCACTTCCGGTACTGGCAGGGGCAATTACCATGTTATTAA : 639
cox1_e1_cD : TGTTTGGTCCGTTCTAGTGACAGCATTCCTACTTTTATTATCACTTCCGGTACTGGCAGGGGCAATTACCATGTTATTAA : 639
TGTTTGGTCCGTTCTAGTGACAGCATTCC ACTTTTATTATCACTTCCGGTACTGGCAGGGGCAATTACCATGTTATTAA
cox1-590
cox1_e1_gD : CTGATCGAAACTTTAATACAACCTTTTTTGATCCTGCTGGAGGGGGAGACCCTATATTATACCAGCATCTCTTTCGGTTC : 719
cox1_e1_cD : CTGATCGAAACTTTAATACAACCTTTTTTGATCCTGCTGGAGGGGGAGACCCTATATTATACCAGCATCTCTTTTGGTTC : 719
CTGATCGAAACTTTAATACAACCTTTTTTGATCCTGCTGGAGGGGGAGACCCTATATTATACCAGCATCTCTTT GGTTC
cox1-715
cox1_e2_gD : CTGGATCCGGTATCATAAGTCATATCGTTTCCACTTTTTCGGGAAAACCGGTTTTCGGGTATCTAGGCATGGTTTATGCC : 80
cox1_e2_cD : CTGGATCCGGTATCATAAGTCATATCGTTTCCACTTTTTCGGGAAAACCGGTTTTCGGGTATCTAGGCATGGTTTATGCC : 80
CTGGATCCGGTATCATAAGTCATATCGTTTCCACTTTTTCGGGAAAACCGGTTTTCGGGTATCTAGGCATGGTTTATGCC
cox1_e2_gD : ATGATCAGTATAGGCGTCCTTGGATTTCTTGTTTGGGCTCATCATATGTTTACTGTGGGCTTAGACGTTGATACCCGTGC : 160
cox1_e2_cD : ATGATCAGTATAGGCGTCCTTGGATTTCTTGTTTGGGCTCATCATATGTTTACTGTGGGCTTAGACGTTGATACCCGTGC : 160
ATGATCAGTATAGGCGTCCTTGGATTTCTTGTTTGGGCTCATCATATGTTTACTGTGGGCTTAGACGTTGATACCCGTGC
cox1_e2_gD : CTACTTCACCGCAGCTACCATGATTATAGCTGTCCCCACTGGAATCAAAATCTTTAGTTGGATCGCTACCATGTGGGGGG : 240
cox1_e2_cD : CTACTTCACCGCAGCTACCATGATTATAGCTGTCCCCACTGGAATCAAAATCTTTAGTTGGATCGCTACCATGTGGGGGG : 240
CTACTTCACCGCAGCTACCATGATTATAGCTGTCCCCACTGGAATCAAAATCTTTAGTTGGATCGCTACCATGTGGGGGG

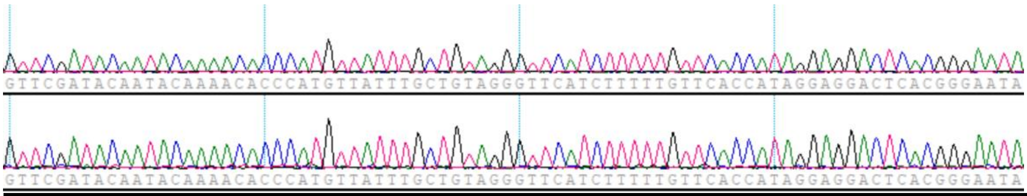

```
cox1_e2_gD : GTTCGATACAATACAAAACACCCATGTTATTTGCTGTAGGGTTCATCTTTTTGTTCACCATAGGAGGACTCACGGGAATA : 320
cox1_e2_cD : GTTCGATACAATACAAAACACCCATGTTATTTGCTGTAGGGTTCATCTTTTTGTTCACCATAGGAGGACTCACGGGAATA : 320
             GTTCGATACAATACAAAACACCCATGTTATTTGCTGTAGGGTTCATCTTTTTGTTCACCATAGGAGGACTCACGGGAATA
```

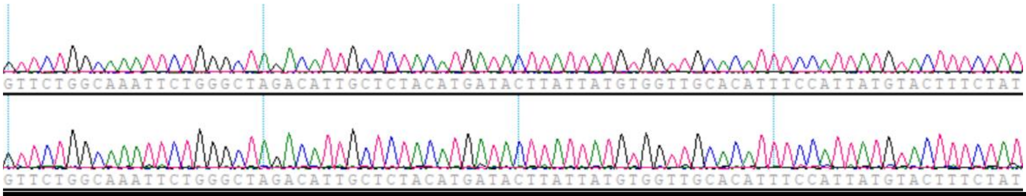

```
cox1_e2_gD : GTTCTGGCAAATTCTGGGCTAGACATTGCTCTACATGATACTTATTATGTGGTTGCACATTTCCATTATGTACTTTCTAT : 400
cox1_e2_cD : GTTCTGGCAAATTCTGGGCTAGACATTGCTCTACATGATACTTATTATGTGGTTGCACATTTCCATTATGTACTTTCTAT : 400
             GTTCTGGCAAATTCTGGGCTAGACATTGCTCTACATGATACTTATTATGTGGTTGCACATTTCCATTATGTACTTTCTAT
```

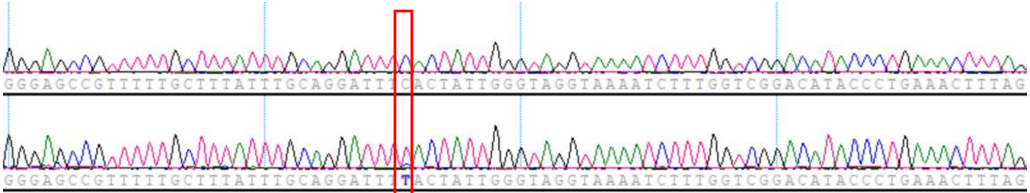

```
cox1_e2_gD : GGGAGCCGTTTTTGCTTTATTTGCAGGATTTCACTATTGGGTAGGTAAAATCTTTGGTCGGACATACCCTGAAACTTTAG : 480
cox1_e2_cD : GGGAGCCGTTTTTGCTTTATTTGCAGGATTTTACTATTGGGTAGGTAAAATCTTTGGTCGGACATACCCTGAAACTTTAG : 480
             GGGAGCCGTTTTTGCTTTATTTGCAGGATTT ACTATTGGGTAGGTAAAATCTTTGGTCGGACATACCCTGAAACTTTAG
                                           cox1-1186
```

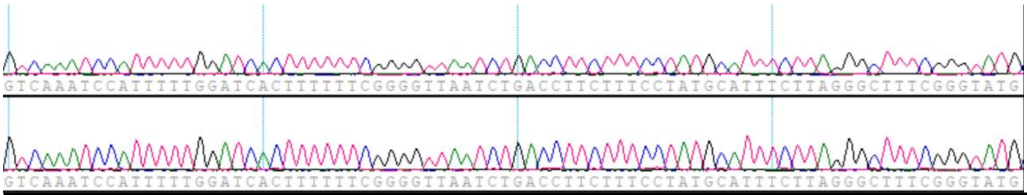

```
cox1_e2_gD : GTCAAATCCATTTTTGGATCACTTTTTTCGGGGTTAATCTGACCTTCTTTCCTATGCATTTCTTAGGGCTTTCGGGTATG : 560
cox1_e2_cD : GTCAAATCCATTTTTGGATCACTTTTTTCGGGGTTAATCTGACCTTCTTTCCTATGCATTTCTTAGGGCTTTCGGGTATG : 560
             GTCAAATCCATTTTTGGATCACTTTTTTCGGGGTTAATCTGACCTTCTTTCCTATGCATTTCTTAGGGCTTTCGGGTATG
```

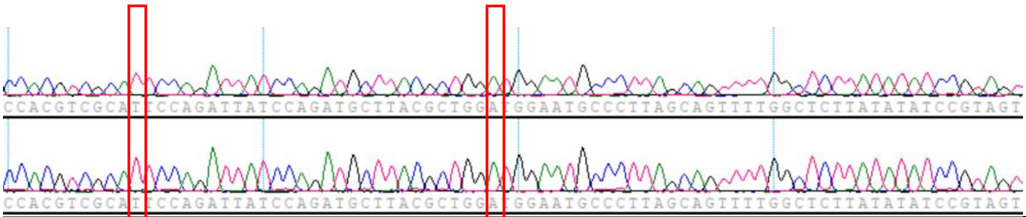

```
cox1_e2_gD : CCACGTCGCATTCCAGATTATCCAGATGCTTACGCTGGATGGAATGCCCTTAGCAGTTTTGGCTCTTATATATCCGTAGT : 640
cox1_e2_cD : CCACGTCGCATTCCAGATTATCCAGATGCTTACGCTGGATGGAATGCCCTTAGCAGTTTTGGCTCTTATATATCCGTAGT : 640
             CCACGTCGCATTCCAGATTATCCAGATGCTTACGCTGGATGGAATGCCCTTAGCAGTTTTGGCTCTTATATATCCGTAGT
```

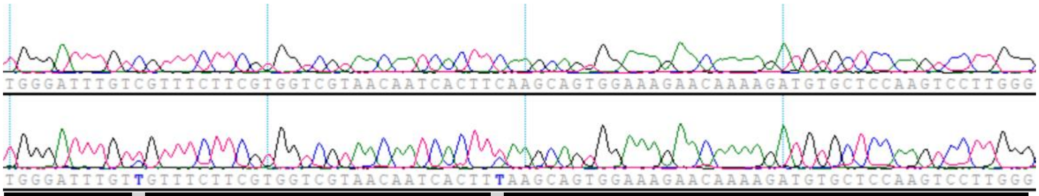

```
cox1_e2_gD : TGGGATTTGTCGTTTCTTCGTGGTCGTAACAATCACTTCAAGCAGTGGAAAGAACAAAAGATGTGCTCCAAGTCCTTGGG : 720
cox1_e2_cD : TGGGATTTGTTGTTTCTTCGTGGTCGTAACAATCACTTTAAGCAGTGGAAAGAACAAAAGATGTGCTCCAAGTCCTTGGG : 720
             TGGGATTTGT GTTTCTTCGTGGTCGTAACAATCACTT AAGCAGTGGAAAGAACAAAAGATGTGCTCCAAGTCCTTGGG
                 cox1-1405                 cox1-1433
```

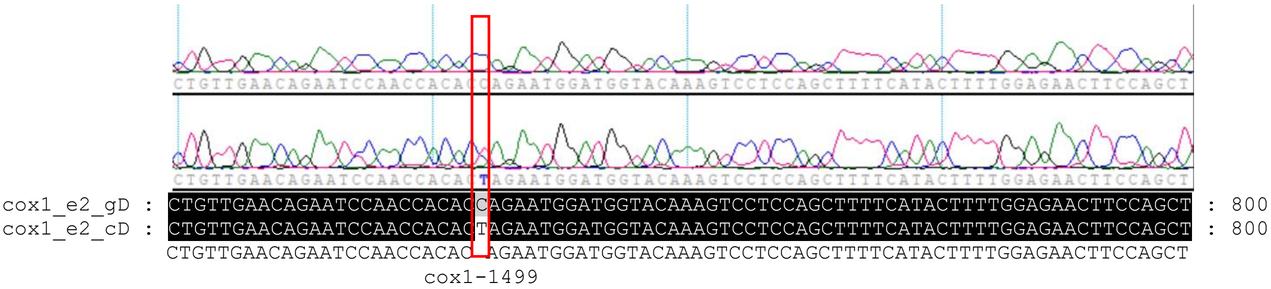

d validation of the *cox3*-245, *cox3*-304, *cox3*-311, *cox3*-314, *cox3*-419, *cox3*-422, *cox3*-566, *cox3*-754, and *cox3*-764 using PCR amplification and Sanger sequencing experiments.

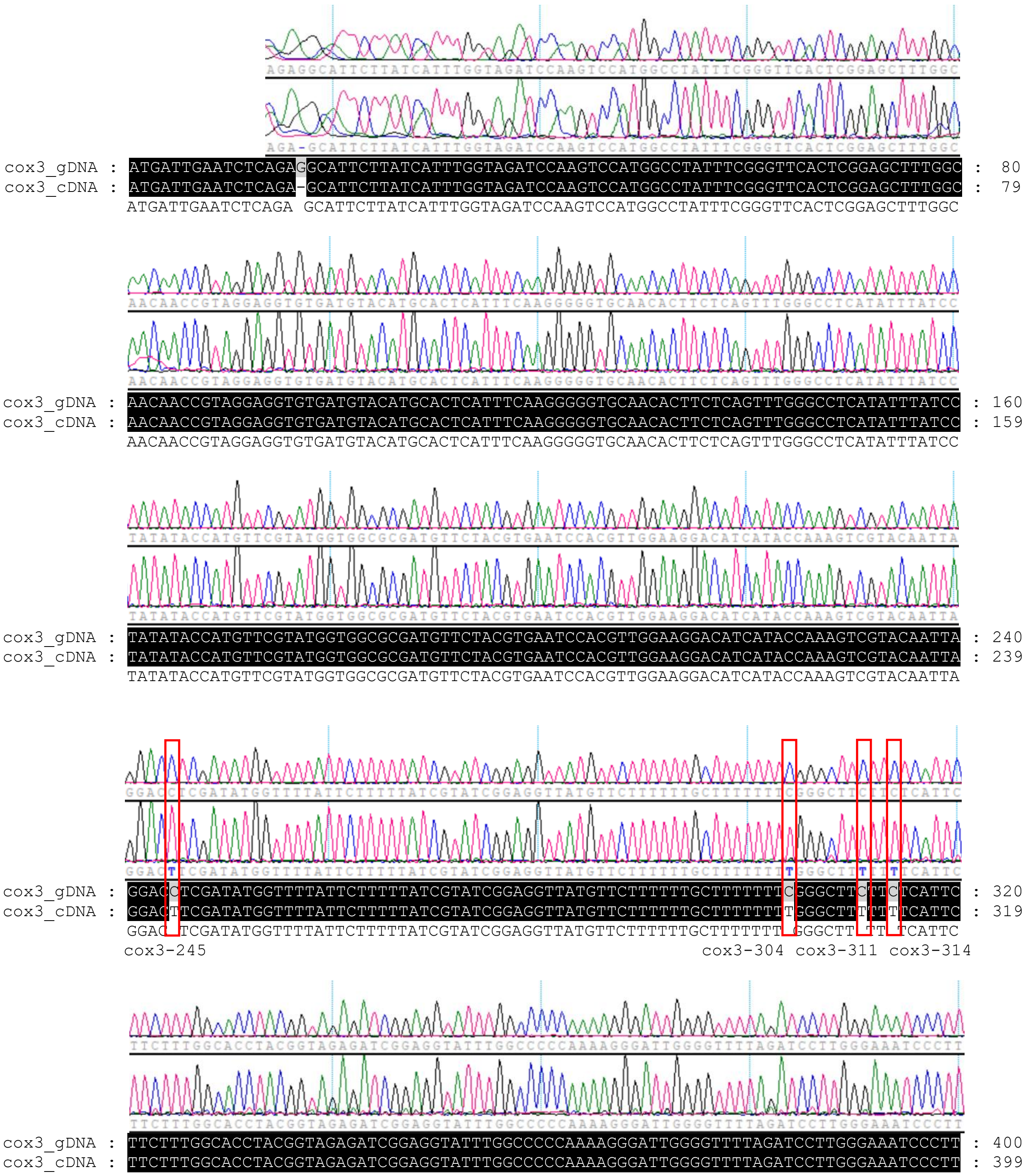

TTCTTTGGCACCTACGGTAGAGATCGGAGGTATTTGGCCCCCAAAAGGGATTGGGGTTTTAGATCCTTGGGAAATCCCTT

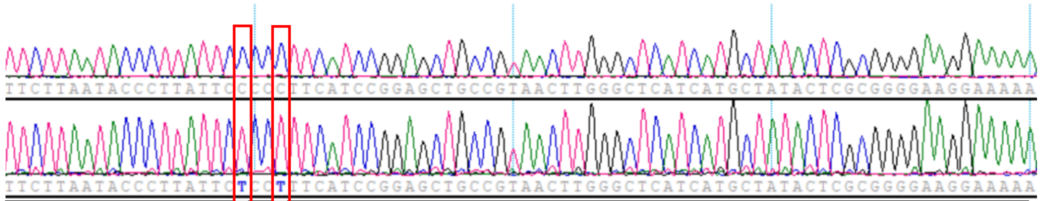

cox3_gDNA : TTCTTAATACCCTTATTGCCCCTTCATCCGGAGCTGCCGTAACTTGGGCTCATCATGCTATACTCGCGGGGAAGGAAAAA : 480
cox3_cDNA : TTCTTAATACCCTTATTGTCCTTTCATCCGGAGCTGCCGTAACTTGGGCTCATCATGCTATACTCGCGGGGAAGGAAAAA : 479
TTCTTAATACCCTTATTG CC TTCATCCGGAGCTGCCGTAACTTGGGCTCATCATGCTATACTCGCGGGGAAGGAAAAA
cox3-419 cox3-422

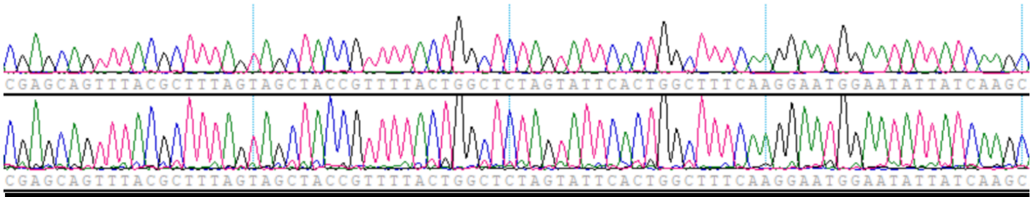

cox3_gDNA : CGAGCAGTTTACGCTTTAGTAGCTACCGTTTTACTGGCTCTAGTATTCACTGGCTTTCAAGGAATGGAATATTATCAAGC : 560
cox3_cDNA : CGAGCAGTTTACGCTTTAGTAGCTACCGTTTTACTGGCTCTAGTATTCACTGGCTTTCAAGGAATGGAATATTATCAAGC : 559
CGAGCAGTTTACGCTTTAGTAGCTACCGTTTTACTGGCTCTAGTATTCACTGGCTTTCAAGGAATGGAATATTATCAAGC

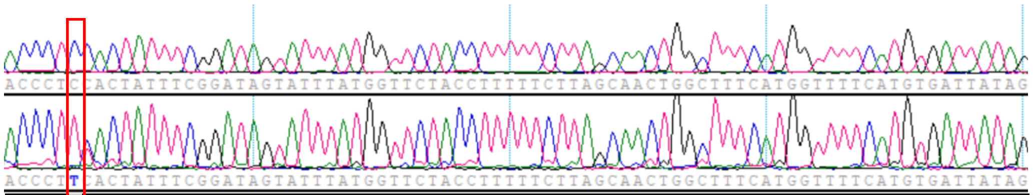

cox3_gDNA : ACCCTCCACTATTTCGGATAGTATTTATGGTTCTACCTTTTTCTTAGCAACTGGCTTTCATGGTTTTCATGTGATTATAG : 640
cox3_cDNA : ACCCTTCACTATTTCGGATAGTATTTATGGTTCTACCTTTTTCTTAGCAACTGGCTTTCATGGTTTTCATGTGATTATAG : 639
ACCCT CACTATTTCGGATAGTATTTATGGTTCTACCTTTTTCTTAGCAACTGGCTTTCATGGTTTTCATGTGATTATAG
cox3-566

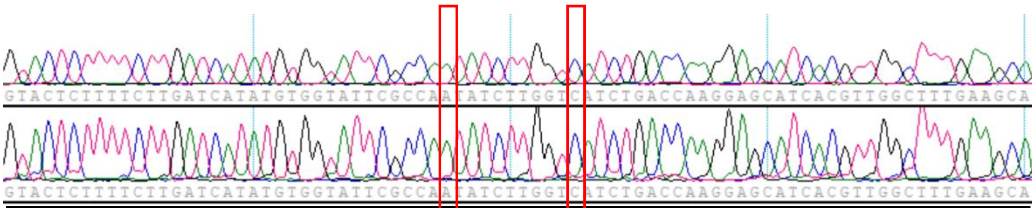

cox3_gDNA : GTACTCTTTTCTTGATCATATGTGGTATTCGCCAATATCTTGGTCATCTGACCAAGGAGCATCACGTTGGCTTTGAAGCA : 720
cox3_cDNA : GTACTCTTTTCTTGATCATATGTGGTATTCGCCAATATCTTGGTCATCTGACCAAGGAGCATCACGTTGGCTTTGAAGCA : 719
GTACTCTTTTCTTGATCATATGTGGTATTCGCCAATATCTTGGTCATCTGACCAAGGAGCATCACGTTGGCTTTGAAGCA

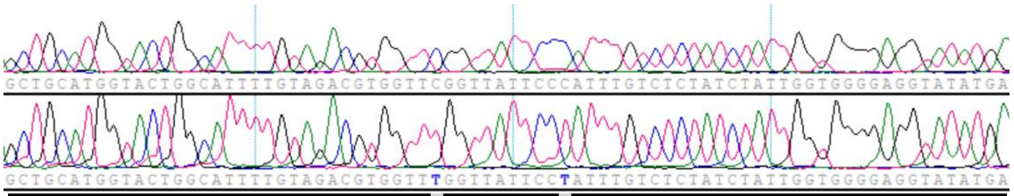

cox3_gDNA : GCTGCATGGTACTGGCATTTTGTAGACGTGGTTCGGTTATTCCCATTTGTCTCTATCTATTGGTGGGGAGGTATATGA : 798
cox3_cDNA : GCTGCATGGTACTGGCATTTTGTAGACGTGGTTTGGTTATTCCTATTTGTCTCTATCTATTGGTGGGGAGGTATATGA : 797
GCTGCATGGTACTGGCATTTTGTAGACGTGGTT GGTTATTCC ATTTGTCTCTATCTATTGGTGGGGAGGTATATGA
cox3-754 cox3-764

e validation of the *mttB*-16, *mttB*-26, *mttB*-64, *mttB*-100, *mttB*-112, *mttB*-128, *mttB*-131, *mttB*-178, *mttB*-188, *mttB*-202 , *mttB*-236, *mttB*-262, *mttB*-328, *mttB*-331, *mttB*-344, *mttB*-346, *mttB*-373, *mttB*-376, *mttB*-379, *mttB*-407, *mttB*-472, *mttB*-497, *mttB*-505, *mttB*-541, *mttB*- 554, *mttB*-578, *mttB*-610, *mttB*-616, *mttB*-667, and *mttB*-713 using PCR amplification and Sanger sequencing experiments.

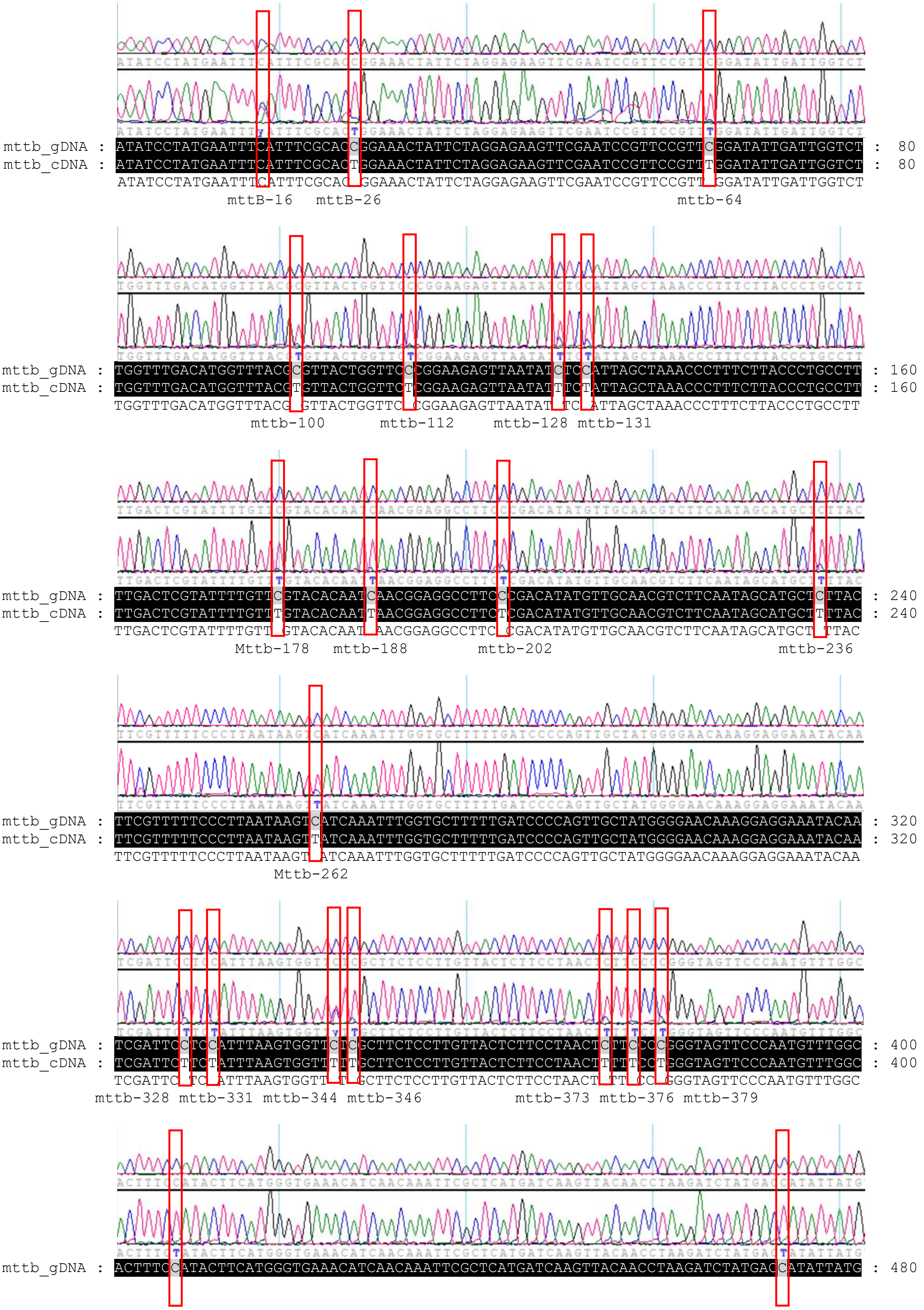

```
mttb_cDNA : ACTTTCTATACTTCATGGGTGAAACATCAACAAATTCGCTCATGATCAAGTTACAACCTAAGATCTATGACTATATTATG : 480
            ACTTTC ATACTTCATGGGTGAAACATCAACAAATTCGCTCATGATCAAGTTACAACCTAAGATCTATGAC ATATTATG
              mttb-407                                                            mttb-472
```

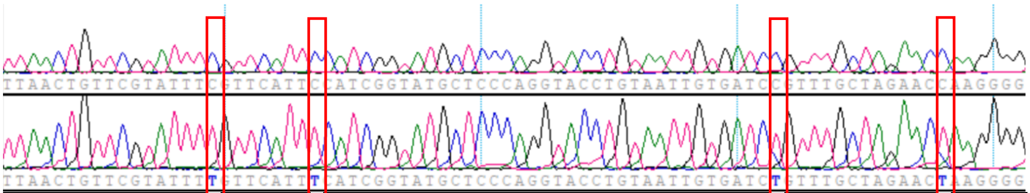

```
mttb_gDNA : TTAACTGTTCGTATTTCGTTCATTCCATCGGTATGCTCCCAGGTACCTGTAATTGTGATCCGTTTGCTAGAACCAAGGGG : 560
mttb_cDNA : TTAACTGTTCGTATTTTGTTCATTTCATCGGTATGCTCCCAGGTACCTGTAATTGTGATCTGTTTGCTAGAACTAAGGGG : 560
            TTAACTGTTCGTATTT GTTCATT CATCGGTATGCTCCCAGGTACCTGTAATTGTGATC GTTTGCTAGAAC AAGGGG
                      mttb-497 mttb-505                             mttb-541   mttb-554
```

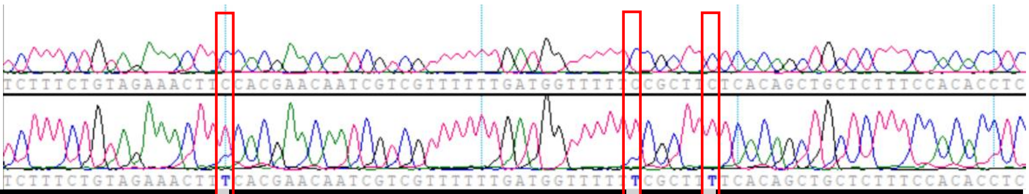

```
mttb_gDNA : TCTTTCTGTAGAAACTTCCACGAACAATCGTCGTTTTTTGATGGTTTTTCCGCTTCTCACAGCTGCTCTTTCCACACCTC : 640
mttb_cDNA : TCTTTCTGTAGAAACTTTCACGAACAATCGTCGTTTTTTGATGGTTTTTTCGCTTTTCACAGCTGCTCTTTCCACACCTC : 640
            TCTTTCTGTAGAAACTT CACGAACAATCGTCGTTTTTTGATGGTTTTT CGCTT TCACAGCTGCTCTTTCCACACCTC
                       mttb-578                             mttb-610 mttb-616
```

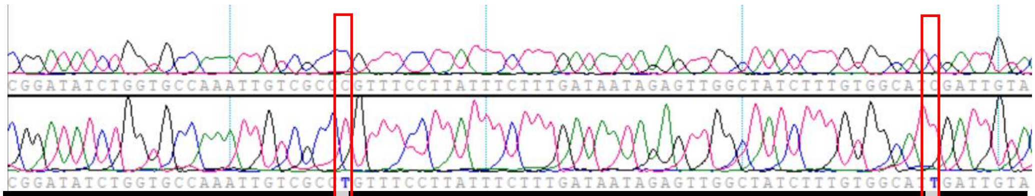

```
mttb_gDNA : CGGATATCTGGTGCCAAATTGTCGCCCGTTTCCTTATTTCTTTGATAATAGAGTTGGCTATCTTTGTGGCATCGATTGTA : 720
mttb_cDNA : CGGATATCTGGTGCCAAATTGTCGCCTGTTTCCTTATTTCTTTGATAATAGAGTTGGCTATCTTTGTGGCATTGATTGTA : 720
            CGGATATCTGGTGCCAAATTGTCGCC GTTTCCTTATTTCTTTGATAATAGAGTTGGCTATCTTTGTGGCAT GATTGTA
                                mttb-667                                        mttb-713
```

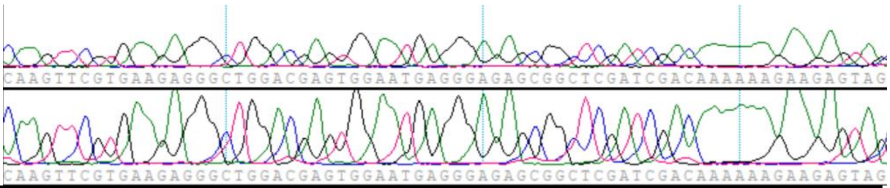

```
mttb_gDNA : CAAGTTCGTGAAGAGGGCTGGACGAGTGGAATGAGGGAGAGCGGCTCGATCGACAAAAAAGAAGAGTAG : 789
mttb_cDNA : CAAGTTCGTGAAGAGGGCTGGACGAGTGGAATGAGGGAGAGCGGCTCGATCGACAAAAAAGAAGAGTAG : 789
            CAAGTTCGTGAAGAGGGCTGGACGAGTGGAATGAGGGAGAGCGGCTCGATCGACAAAAAAGAAGAGTAG
```

f validation of the *nad3*-44, *nad3*-62, *nad3*-79, *nad3*-80, *nad3*-124, *nad3*-146, *nad3*-208, *nad3*-209, *nad3*-215, *nad3*-230, *nad3*-247, *nad3*-251, *nad3*-266, *nad3*-275, *nad3*-317, *nad3*-344, and *nad3*-349 using PCR amplification and Sanger sequencing experiments.

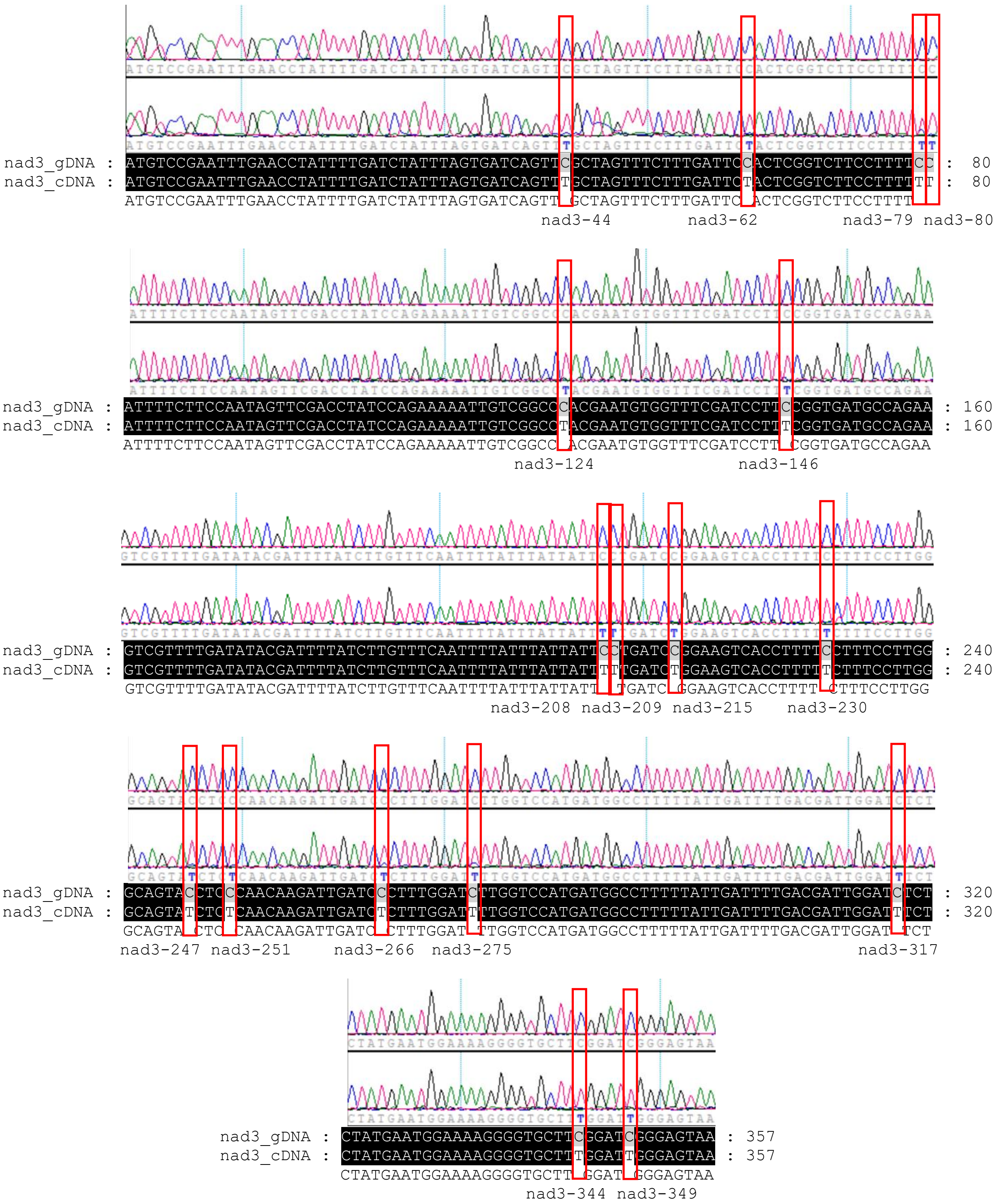

g validation of the *nad4L*-11, *nad4L*-17, *nad4L*-25, *nad4L*-56, *nad4L*-65, *nad4L*-70, *nad4L*-80, *nad4L*-101, *nad4L*-128, *nad4L*-149, *nad4L*-158, *nad4L*-167, *nad4L*-222, and *nad4L*-251 using PCR amplification and Sanger sequencing experiments.

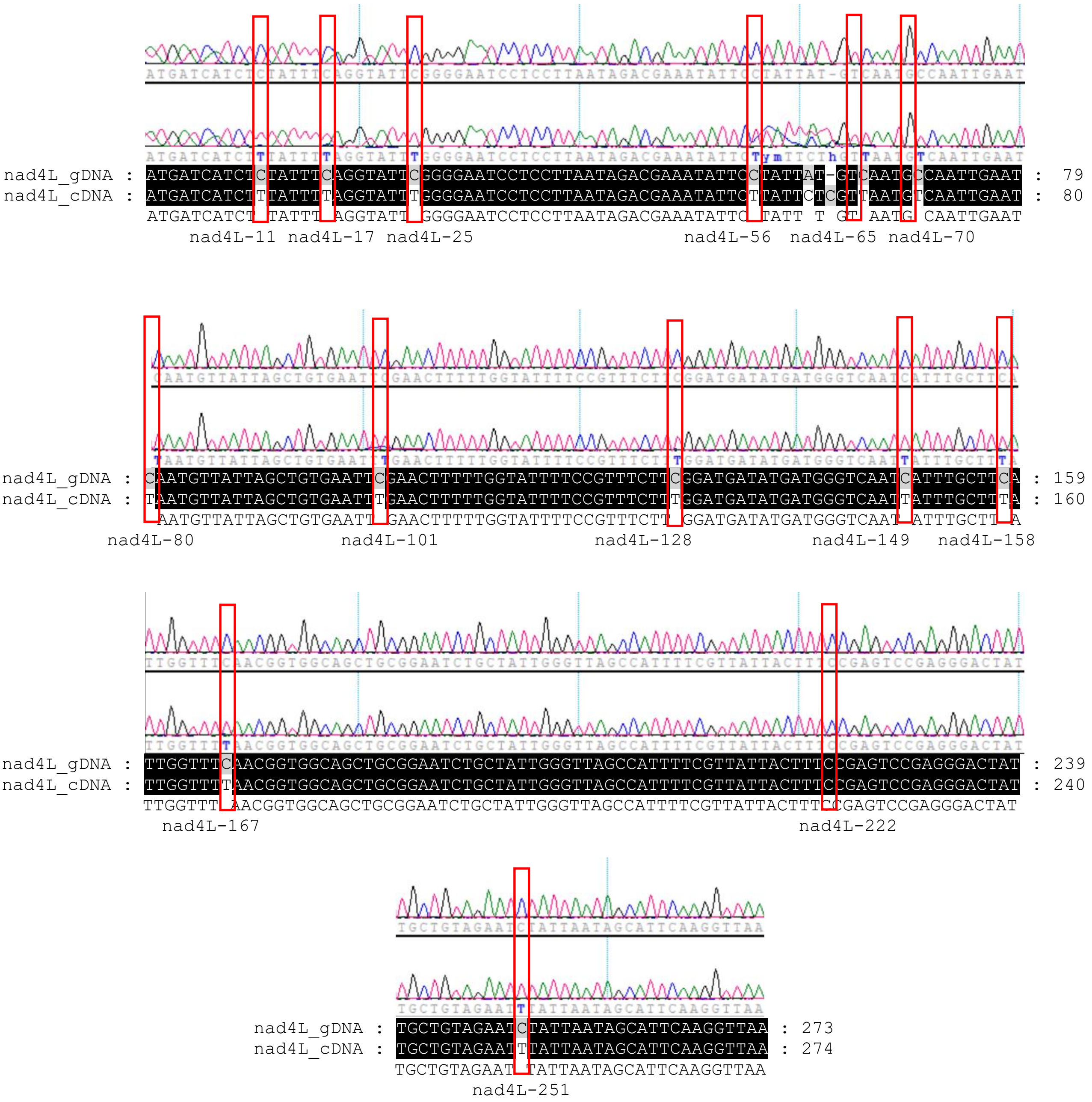

h validation of the *nad9*-15, *nad9*-92, *nad9*-113, *nad9*-167, *nad9*-298, *nad9*-328, *nad9*-368, *nad9*-398, and *nad9*-439 using PCR amplification and Sanger sequencing experiments.

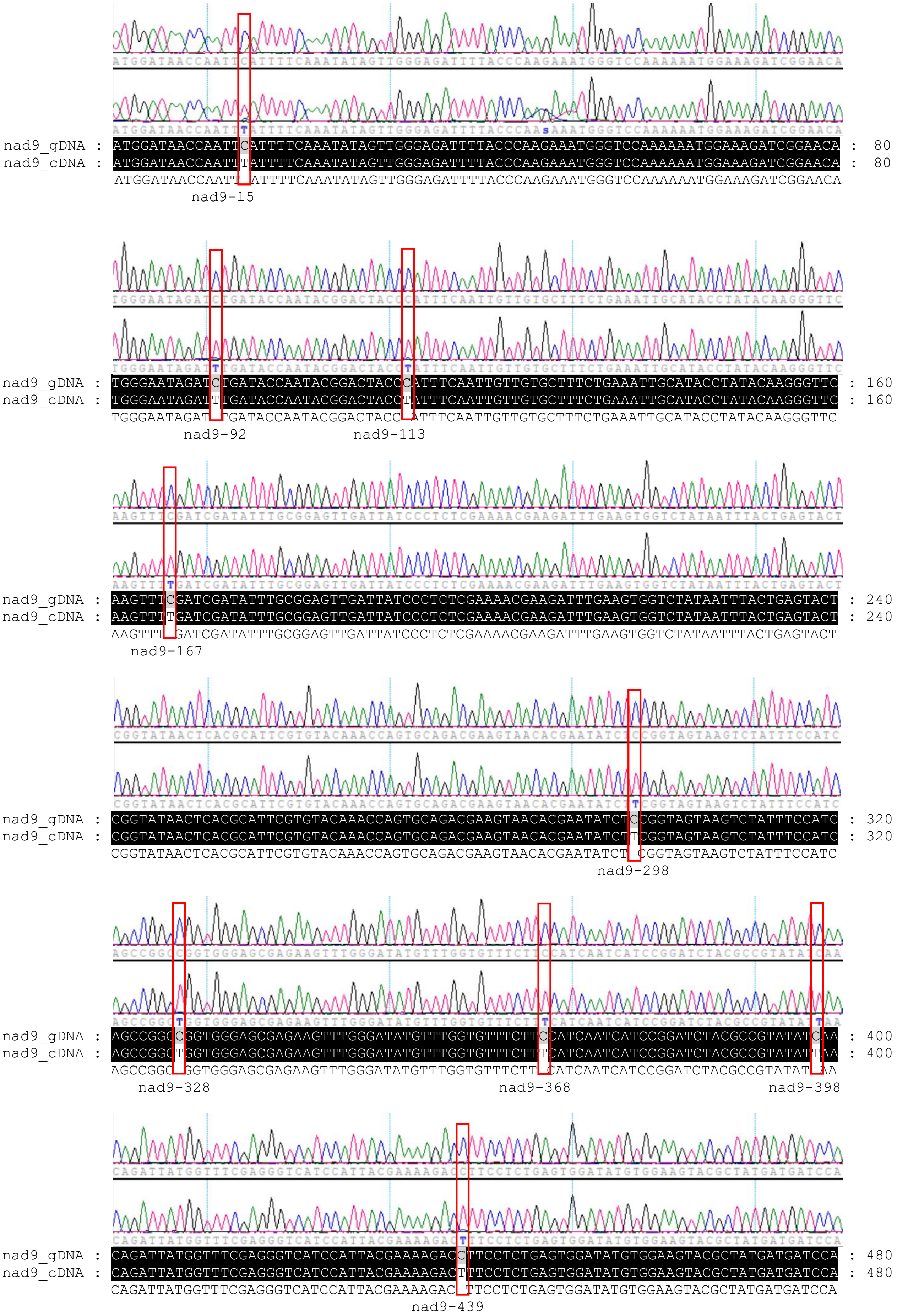

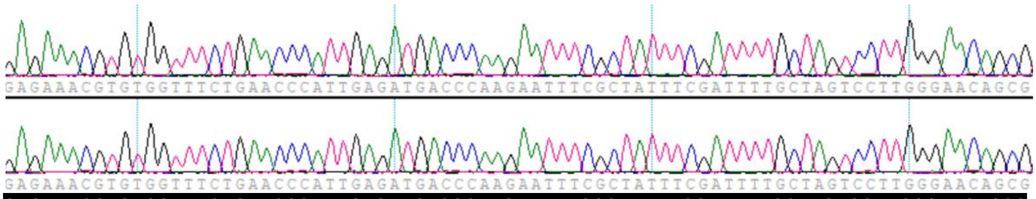

nad9_gDNA : GAGAAACGTGTGGTTTCTGAACCCATTGAGATGACCCAAGAATTTCGCTATTTCGATTTTGCTAGTCCTTGGGAACAGCG : 560
nad9_cDNA : GAGAAACGTGTGGTTTCTGAACCCATTGAGATGACCCAAGAATTTCGCTATTTCGATTTTGCTAGTCCTTGGGAACAGCG : 560
GAGAAACGTGTGGTTTCTGAACCCATTGAGATGACCCAAGAATTTCGCTATTTCGATTTTGCTAGTCCTTGGGAACAGCG

i validation of the *rpl5*-35, *rpl5*-47, *rpl5*-160, *rpl5*-509, *rpl5*-512, and *rpl5*-529 using PCR amplification and Sanger sequencing experiments.

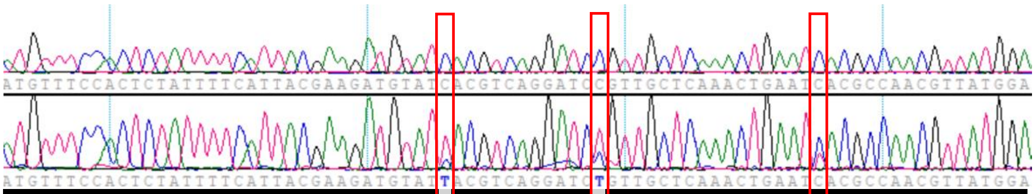

rpl5_gDNA : ATGTTTCCACTCTATTTTCATTACGAAGATGTATCACGTCAGGATCCGTTGCTCAAACTGAATCACGCCAACGTTATGGA : 80
rpl5_cDNA : ATGTTTCCACTCTATTTTCATTACGAAGATGTATTACGTCAGGATCTGTTGCTCAAACTGAATCACGCCAACGTTATGGA : 80
ATGTTTCCACTCTATTTTCATTACGAAGATGTAT ACGTCAGGATC GTTGCTCAAACTGAATCACGCCAACGTTATGGA

rpl5-35 rpl5-47 rpl5-64

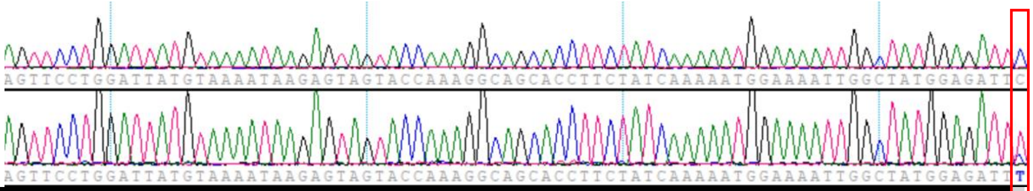

rpl5_gDNA : AGTTCCTGGATTATGTAAAATAAGAGTAGTACCAAAGGCAGCACCTTCTATCAAAAATGGAAAATTGGCTATGGAGATTC : 160
rpl5_cDNA : AGTTCCTGGATTATGTAAAATAAGAGTAGTACCAAAGGCAGCACCTTCTATCAAAAATGGAAAATTGGCTATGGAGATTT : 160
AGTTCCTGGATTATGTAAAATAAGAGTAGTACCAAAGGCAGCACCTTCTATCAAAAATGGAAAATTGGCTATGGAGATT

rpl5-160

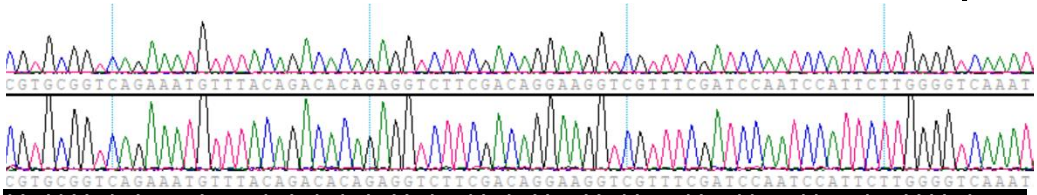

rpl5_gDNA : CGTGCGGTCAGAAATGTTTACAGACACAGAGGTCTTCGACAGGAAGGTCGTTTCGATCCAATCCATTCTTGGGGTCAAAT : 240
rpl5_cDNA : CGTGCGGTCAGAAATGTTTACAGACACAGAGGTCTTCGACAGGAAGGTCGTTTCGATCCAATCCATTCTTGGGGTCAAAT : 240
CGTGCGGTCAGAAATGTTTACAGACACAGAGGTCTTCGACAGGAAGGTCGTTTCGATCCAATCCATTCTTGGGGTCAAAT

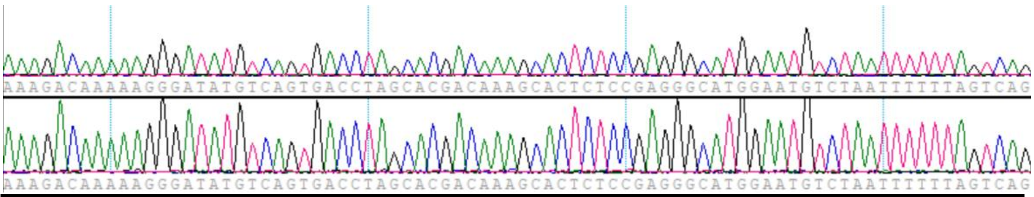

rpl5_gDNA : AAAGACAAAAAGGGATATGTCAGTGACCTAGCACGACAAAGCACTCTCCGAGGGCATGGAATGTCTAATTTTTTAGTCAG : 320
rpl5_cDNA : AAAGACAAAAAGGGATATGTCAGTGACCTAGCACGACAAAGCACTCTCCGAGGGCATGGAATGTCTAATTTTTTAGTCAG : 320
AAAGACAAAAAGGGATATGTCAGTGACCTAGCACGACAAAGCACTCTCCGAGGGCATGGAATGTCTAATTTTTTAGTCAG

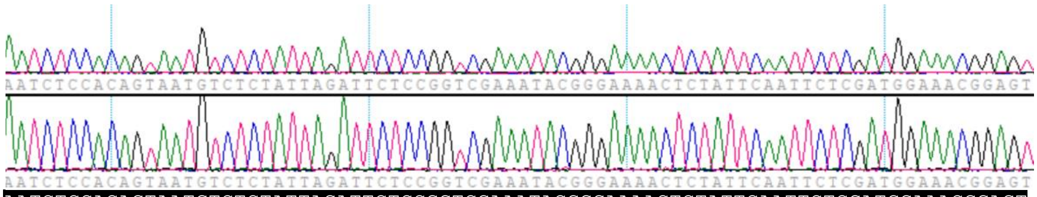

rpl5_gDNA : AATCTCCACAGTAATGTCTCTATTAGATTCTCCGGTCGAAATACGGGAAAACTCTATTCAATTCTCGATGGAAACGGAGT : 400
rpl5_cDNA : AATCTCCACAGTAATGTCTCTATTAGATTCTCCGGTCGAAATACGGGAAAACTCTATTCAATTCTCGATGGAAACGGAGT : 400
AATCTCCACAGTAATGTCTCTATTAGATTCTCCGGTCGAAATACGGGAAAACTCTATTCAATTCTCGATGGAAACGGAGT

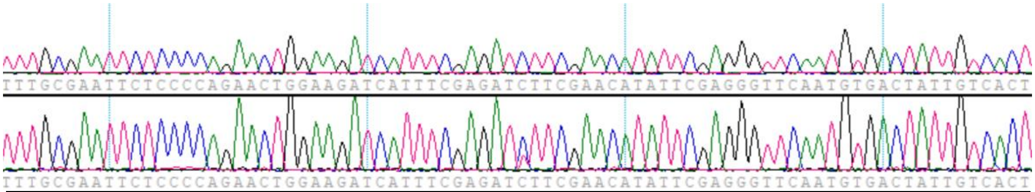

```
rpl5_gDNA : TTTGCGAATTCTCCCCAGAACTGGAAGATCATTTCGAGATCTTCGAACATATTCGAGGGTTCAATGTGACTATTGTCACT : 480
rpl5_cDNA : TTTGCGAATTCTCCCCAGAACTGGAAGATCATTTCGAGATCTTCGAACATATTCGAGGGTTCAATGTGACTATTGTCACT : 480
            TTTGCGAATTCTCCCCAGAACTGGAAGATCATTTCGAGATCTTCGAACATATTCGAGGGTTCAATGTGACTATTGTCACT
```

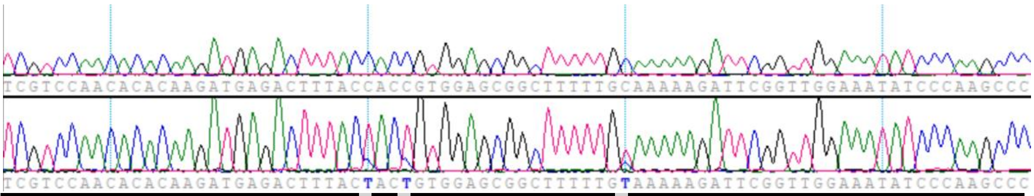

```
rpl5_gDNA : TCGTCCAACACACAAGATGAGACTTTACCACCGTGGAGCGGCTTTTTGCAAAAAGATTCGGTTGGAAATATCCCAAGCCC : 560
rpl5_cDNA : TCGTCCAACACACAAGATGAGACTTTACTACTGTGGAGCGGCTTTTTGTAAAAAGATTCGGTTGGAAATATCCCAAGCCC : 560
            TCGTCCAACACACAAGATGAGACTTTAC AC GTGGAGCGGCTTTTTG AAAAAGATTCGGTTGGAAATATCCCAAGCCC
                                    rpl5-509 rpl5-512       rpl5-529
```

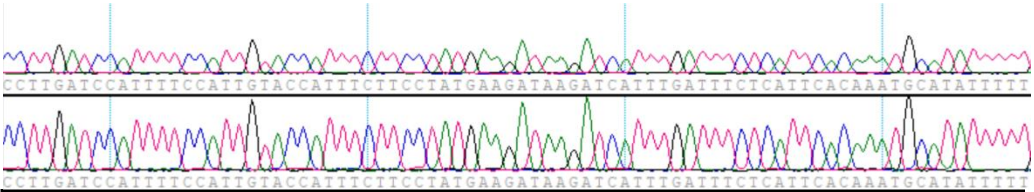

```
rpl5_gDNA : CCTTGATCCATTTTCCATTGTACCATTTCTTCCTATGAAGATAAGATCATTTGATTTCTCATTCACAAATGCATATTTTT : 640
rpl5_cDNA : CCTTGATCCATTTTCCATTGTACCATTTCTTCCTATGAAGATAAGATCATTTGATTTCTCATTCACAAATGCATATTTTT : 640
            CCTTGATCCATTTTCCATTGTACCATTTCTTCCTATGAAGATAAGATCATTTGATTTCTCATTCACAAATGCATATTTTT
```

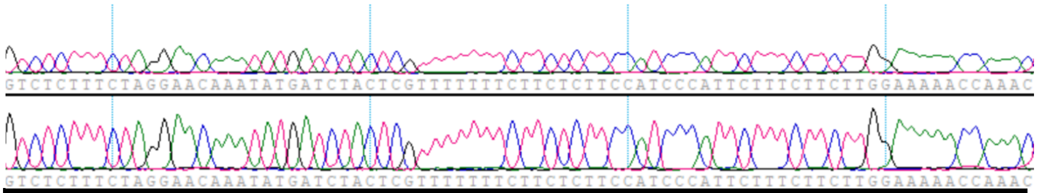

```
rpl5_gDNA : GTCTCTTTCTAGGAACAAATATGATCTACTCGTTTTTTTCTTCTCTTCCATCCCATTCTTTCTTCTTGGAAAAACCAAAC : 720
rpl5_cDNA : GTCTCTTTCTAGGAACAAATATGATCTACTCGTTTTTTTCTTCTCTTCCATCCCATTCTTTCTTCTTGGAAAAACCAAAC : 720
            GTCTCTTTCTAGGAACAAATATGATCTACTCGTTTTTTTCTTCTCTTCCATCCCATTCTTTCTTCTTGGAAAAACCAAAC
```

j validation of the *rpl16*-221 using PCR amplification and Sanger sequencing experiments.

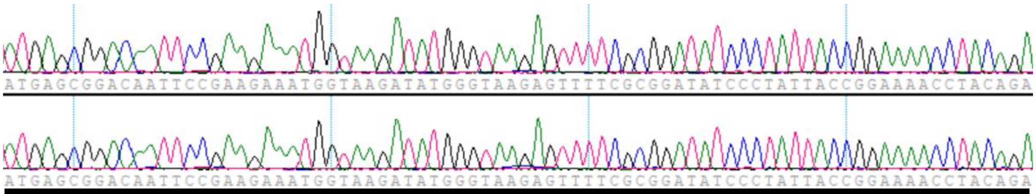

```
rpl16_gDNA : ATGAGCGGACAATTCCGAAGAAATGGTAAGATATGGGTAAGAGTTTTCGCGGATATCCCTATTACCGGAAAACCTACAGA :  80
rpl16_cDNA : ATGAGCGGACAATTCCGAAGAAATGGTAAGATATGGGTAAGAGTTTTCGCGGATATCCCTATTACCGGAAAACCTACAGA :  80
             ATGAGCGGACAATTCCGAAGAAATGGTAAGATATGGGTAAGAGTTTTCGCGGATATCCCTATTACCGGAAAACCTACAGA
```

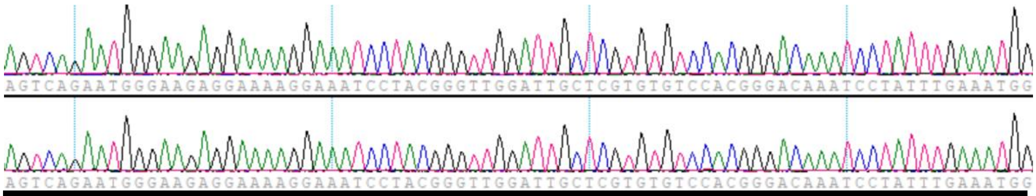

```
rpl16_gDNA : AGTCAGAATGGGAAGAGGAAAAGGAAATCCTACGGGTTGGATTGCTCGTGTGTCCACGGGACAAATCCTATTTGAAATGG : 160
rpl16_cDNA : AGTCAGAATGGGAAGAGGAAAAGGAAATCCTACGGGTTGGATTGCTCGTGTGTCCACGGGACAAATCCTATTTGAAATGG : 160
             AGTCAGAATGGGAAGAGGAAAAGGAAATCCTACGGGTTGGATTGCTCGTGTGTCCACGGGACAAATCCTATTTGAAATGG
```

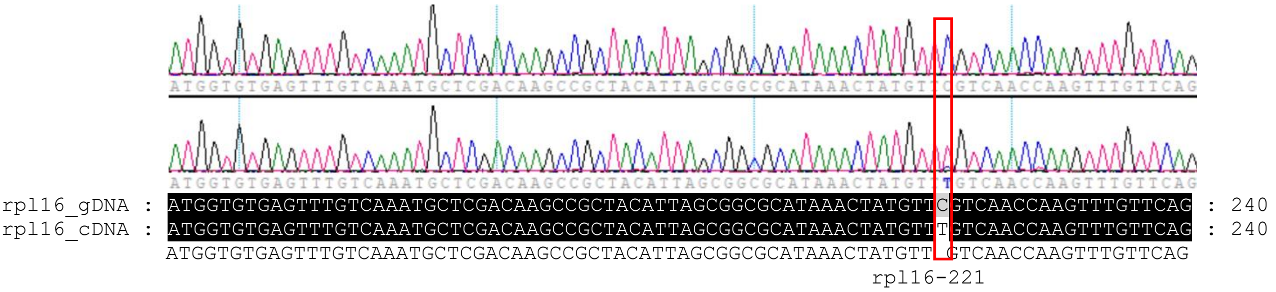

k validation of the *rps13*-56, *rps13*-100, *rps13*-287 using PCR amplification and Sanger sequencing experiments.

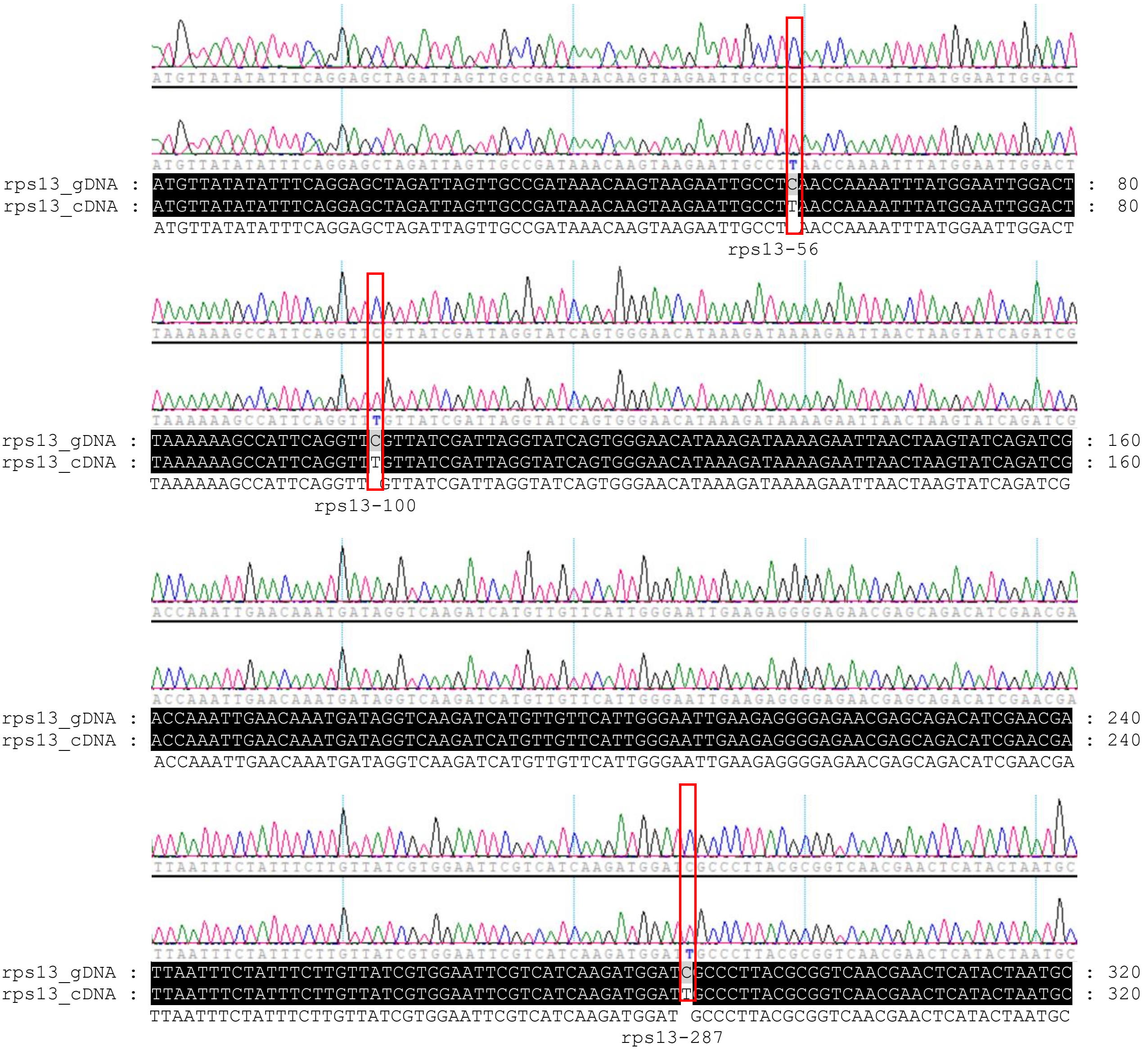

Supplement: Supplementary file 1 [file ijms-24-05372-s001.zip › ijms-2148654-supplementary.pdf]
